# A tautomerized ligand enabled meta selective C–H borylation of phenol

**DOI:** 10.1038/s41467-023-42310-6

**Published:** 2023-10-30

**Authors:** Saikat Guria, Mirja Md Mahamudul Hassan, Jiawei Ma, Sayan Dey, Yong Liang, Buddhadeb Chattopadhyay

**Affiliations:** 1 Department of Biological & Synthetic Chemistry, Centre of Biomedical Research, SGPGIMS Campus, Raebareli Road, Lucknow, 226014 Uttar Pradesh India; 2 State Key Laboratory of Coordination Chemistry, Jiangsu Key Laboratory of Advanced Organic Materials, Chemistry and Biomedicine Innovation Center, School of Chemistry and Chemical Engineering, Nanjing University, 210023 Nanjing, China

**Keywords:** Synthetic chemistry methodology, Catalytic mechanisms, Homogeneous catalysis

## Abstract

Remote meta selective C–H functionalization of aromatic compounds remains a challenging problem in chemical synthesis. Here, we report an iridium catalyst bearing a bidentate pyridine-pyridone (PY-PYRI) ligand framework that efficiently catalyzes this meta selective borylation reaction. We demonstrate that the developed concept can be employed to introduce a boron functionality at the remote meta position of phenols, phenol containing bioactive and drug molecules, which was an extraordinary challenge. Moreover, we have demonstrated that the method can also be applied for the remote C6 borylation of indole derivatives including tryptophan that was the key synthetic precursor for the total synthesis of Verruculogen and Fumitremorgin A alkaloids. The inspiration of this catalytic concept was started from the O–Si secondary interaction, which by means of several more detailed control experiments and detailed computational investigations revealed that an unprecedented Bpin shift occurs during the transformation of iridium bis(boryl) complex to iridium tris(boryl) complex, which eventually control the remote meta selectivity by means of the dispersion between the designed ligand and steering silane group.

## Abstract (web summary)

Remote meta selective C–H Functionalization of aromatic compounds remains challenging in chemical synthesis. Here, the authors report an iridium catalyst bearing a bidentate pyridine-pyridone ligand framework that efficiently catalyzes this meta selective borylation reaction.

## Introduction

Transition metal-catalyzed C–H bond activation and functionalization^1–10^ of aromatic compounds has been branded as one of the most significant chemical transformations. This has a profound impact in modern synthetic organic chemistry, ranging from laboratory methods to industrial deployment^11,12^. However, the key underlying principles for the success of the metal catalysis lies on the two important factors, such as: (i) design and synthesis of new generation ligand framework that can produce highly reactive catalyst system^13,14^ and (ii) substrates’ structure modifications^15^ by which site selectivity could be controlled by the steric crowding^16–20^ or various weak interactions^21–24^ of the aromatic compounds among several similar type of C–H bonds via the ligand–substrate pre-organization^25,26^. In recent times, many elegant approaches^27^ have been developed for the functionalization of proximal^14,28–30^ and remote C–H bonds^1,3,31–39^ of arenes by the design of either new ligand frameworks with an extended architectures featuring a weak coordinating functional groups^40^ or templates^41^ as well as transient mediators^42^ or transient directing groups^43^ attached with the substrates. While ligand having an extended architecture or template approaches are extremely important to functionalize the remotely located C–H bonds of arenes, but requirement of multi-step preparation of the linkers of the ligands and templates of the aromatic substrates significantly limit the wide application of the methods^44^.

**Fig. 1:**
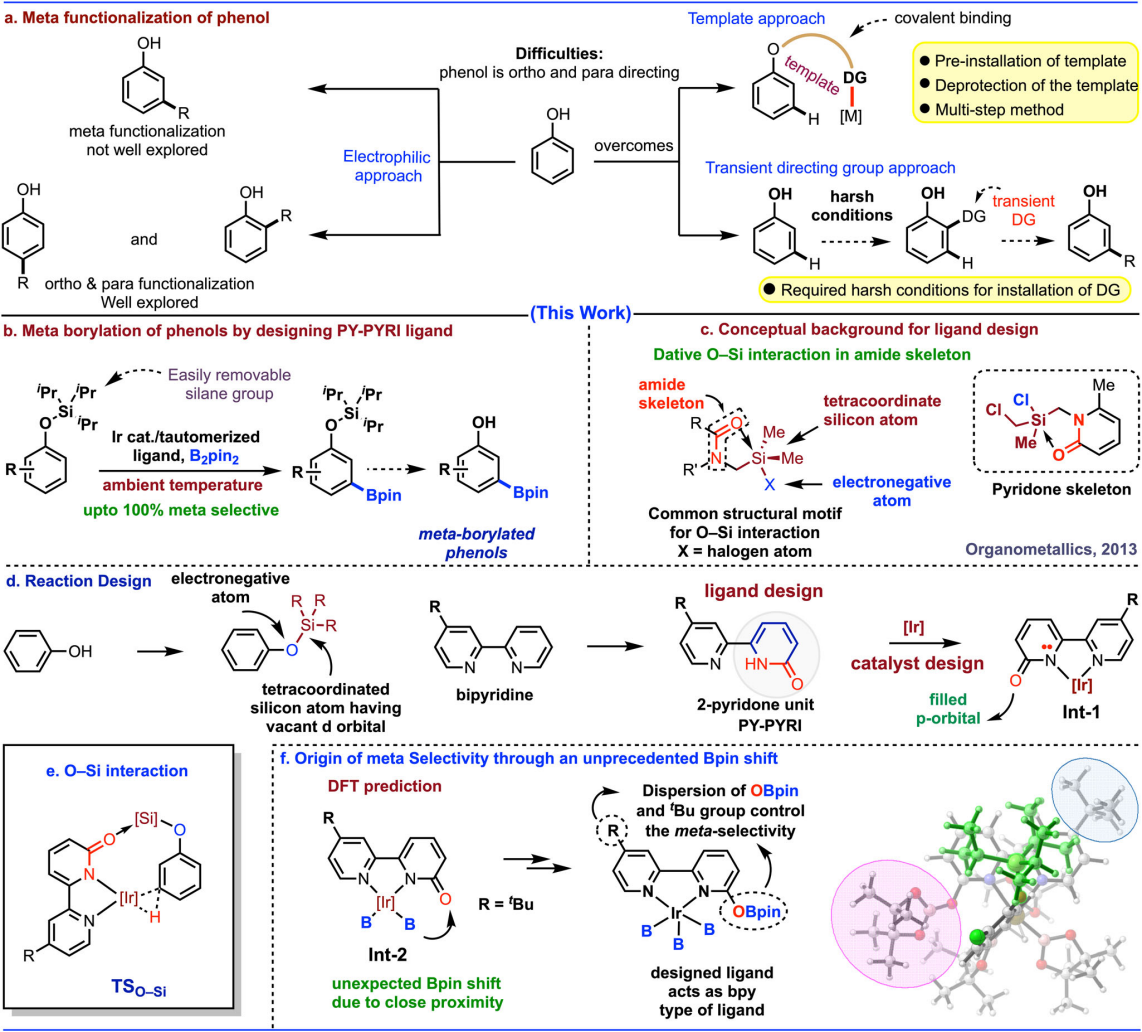
Previous limitations of meta functionalization of phenols and present state of art for the meta borylation of phenols. **a** Meta functionalization of phenol. **b** Present work of meta selective borylation of phenols at ambient temperature. **c** Conceptual background for ligand design. **d** Reaction design. **e** O–Si interaction. **f** Origin of meta selectivity on the basis of DFT calculation.

Among numerous aromatic substrates, phenols are the most widespread aromatic compounds that acquired household products including several bioactive to important drug molecules^45^. Moreover, it is well-documented that 10% of the top 200 selling pharmaceuticals contain a phenol and several others employ phenols as synthetic intermediates^46^. Furthermore, phenols are also key components of the biopolymers melanin, lignin, resins, and polyphenylene oxides^45–47^. In industry, phenol is routinely used as a raw material to make numerous important components by means of its diversification via the synthetic manipulation^45,46^. Thus, direct functionalization of phenols would be a significant development for the rapid access of numerous important products^47^. In this context, traditional electrophilic substitution is an alternative method that affords variously substituted phenols (Fig. 1a)^48^. Employing this method, one can easily access ortho and para substituted phenol derivatives, although often remain a chance to have mixture of isomers. However, functionalization of the remote meta C-H bonds of phenols and getting meta functionalized phenols^49^ is extremely difficult because of the extreme inertness of the meta C–H bonds. Several pioneering approaches have been developed by Yu and others either using template method^50–52^ or transient directing group by Larrosa^2^ (Fig. 1a). But, achieving the meta functionalized products using these methods, it is essential to have specialized substrates that limits the application of the methods.

Having tremendous importance of catalytic C-H borylation^53–58^ in organic synthesis, we report here a concept for the meta selective C-H borylation of phenols through a unique ligand design strategy that has never been utilized in the C-H functionalization chemistry (Fig. 1b). Literature reports revealed^59,60^ that the most common structural motif for the O–Si interaction can be found in the amide skeleton, where a filled p-orbital of carbonyl oxygen atom interact with the vacant d-orbital of the tetracoordinated silicon atom consisting of at least one electronegative atom (Fig. 1c). Inspired from this background reports^59,60^, we initially proposed a hypothesis where phenol is protected with an easily removable silane group that will meet all the necessary criteria for the weak O–Si interaction with 2-pyridone moiety having amide skeleton. The designed ligand (PY-PYRI) consists of two parts, one part is the simple pyridine unit (PY) and the other one is a 2-pyridone unit (PYRI)^61–64^, which was redesigned by the skeletal modification of the bipyridine core structure (Fig. 1d). Based on the above-mentioned findings, we anticipated that the designed PY-PYRI ligand may control the meta selectivity owing to the following two reasons. Firstly, in presence of [Ir(cod)OMe]_2_, the ligand (PY-PYRI) will generate a complex (**Int-1**). Secondly, the p-orbital of the oxygen atom of the 2-pyridone unit will interact with the vacant d-orbital of the tetracoordinated silicon atom of the substrate through **TS**_**O-Si**_ (Fig. 1e). While experimental observations indicated that the secondary O–Si interaction was the key to control the remote meta selectivity, surprisingly, what we found from the computational calculations is somewhat different from our anticipated O–Si interaction. It was revealed that the **Int-1** derived from the PY-PYRI ligand, in presence of di-boron reagent generated iridium bis(boryl) complex (**Int-2**), which underwent an unprecedented Bpin shift due to the close proximity of the 2-pyridone carbonyl oxygen atom and Bpin group attached with iridium atom. A dispersion force was observed by the ligand containing OBpin group and substituent (R = ^t^Bu) of the PY-PYRI ligand that creates a suitable pocket for the phenol bearing tri-isopropyl group for the meta selective borylation reactions (Fig. 1f).

## Results

**Fig. 2:**
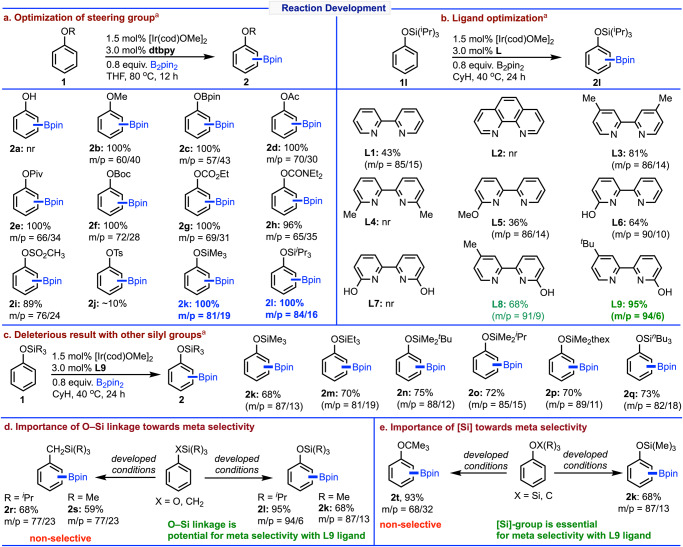
Reaction development. **a** Optimization of steering group. **b** Ligand optimization. **c** Deleterious result with other silyl groups. **d** Importance of O–Si linkage towards meta selectivity. **e** Importance of [Si] towards meta selectivity. Reactions are on 0.2 mmol scales. ^a^Conversion was reported. See SI for details.

The investigation of this meta selective C-H borylation started with phenol (**1a**) using the standard dtbpy ligand under the iridium-catalyzed conditions at 80 °C in THF solvent, where free phenol does not participate in the reaction (Fig. 2a). On the other hand, performing the borylations with several steering groups such as, OMe, OBpin, acetyl, pivalate, OBoc, carbamate and other sulfonate groups proved to be nonselective. Interestingly, when the reaction was examined using SiMe_3_ as steering group (**1k**), it gave 81% meta selectivity with dtbpy ligand. The selectivity was further enhanced to 84% upon changing the steering group from SiMe_3_ to Si^i^Pr_3_ group (**1l**).

After identifying the best choice of steering group (Si^i^Pr_3_) for meta borylation of phenol, we then studied the same reaction with several bipyridine ligands (Fig. 2b). It was observed that while bipyridine ligand (**L1**) afforded same type of selectivity similar to the dtbpy ligand with less conversion, no reaction occurred with 1,10-phenanthroline (**L2**). Testing the reaction with 4,4’-dimethyl bpy (**L3**) also resulted in 86% meta selectivity with 81% conversion. As expected, due to steric crowding ligand (**L4**) bearing 6,6’-disubtituted methyl group failed to undergo the reaction. Notably, while a monosubstituted ligand (**L5**) having a methoxy group resulted in 86% meta borylation with 36% conversion, the ligand (**L6**) containing a hydroxy group that can undergo tautomerization provided excellent meta selectivity (90%) with improved conversion (64%). Surprisingly, introducing two hydroxy groups into the ligand (**L7**), no reaction occurred. To see the electronic effect of the ligand, we performed the borylation with (**L9**) ligand that yielded minor improvement (91% meta selectivity, 68% conversion) with respect to the ligand (**L6**). Remarkably, when the reaction was conducted with the ligand (**L9**) having a *tert*-butyl group, it provided 94% meta selectivity along with excellent conversion of the meta borylation (95%).

Having established the optimized reaction conditions employing the tri-isopropyl silyl steering group, we then became interested in other silyl steering groups (**1k, 1m–1q**) which exhibited deleterious outcomes for the meta borylation (Fig. 2c). Moreover, we found that the essential criteria for achieving high level of meta selectivity is that the substrate must have an O–Si linkage, because substrates having C–Si linkage (**1r**, **1s**) afforded non-selective borylation (Fig. 2d). Furthermore, to sense the importance of silyl group [Si] was replaced with the [C] (**1t**) that also resulted in non-selective borylation reactions (Fig. 2e). Thus, it may be stated that for achieving high degree of meta selective borylation, it is essential that substrates should have silane steering group along with the most important 2-pyridone ligand (**L9**: Py-PYRI).

**Fig. 3:**
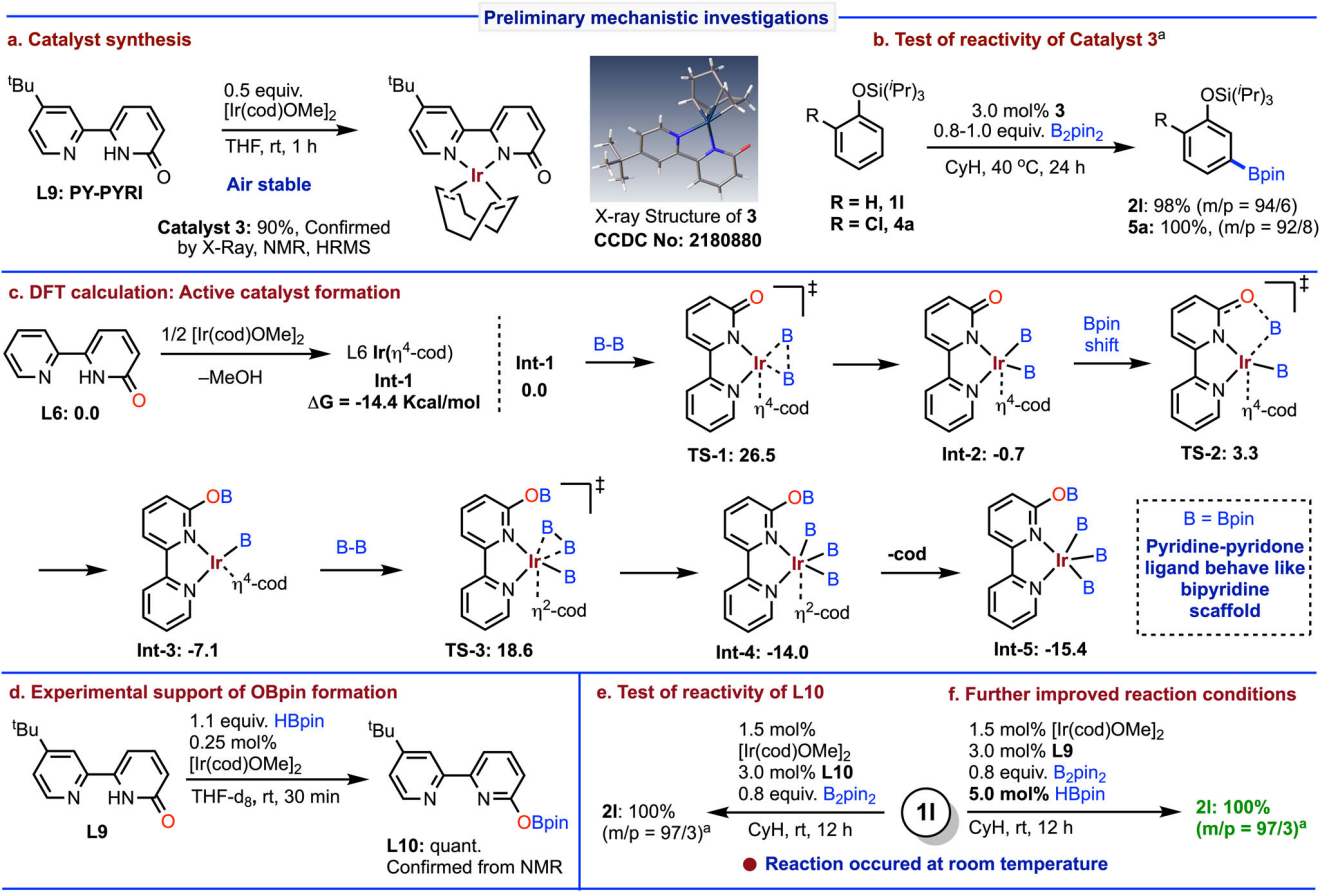
Preliminary mechanistic investigations. **a** Catalyst synthesis. **b** Test of reactivity of catalyst **3**. **c** DFT calculation: Active catalyst formation. **d** Experimental support of OBpin formation. **e** Test of reactivity of **L10**. **f** Further improved reaction conditions. Reactions are on 0.2 mmol scales. ^a^Conversion was reported. See SI for details.

To this end, we were curious about the catalyst structure with the designed tautomeric ligand (**L9: PY–PYRI**) for the meta selective borylation. Accordingly, to sense the binding behavior of the developed ligand system we performed a stoichiometric reaction between the ligand (**L9**) and [Ir(cod)OMe]_2_, which gave a complex (**3**) that have 2-pyridone type unit confirmed by X-ray and NMR spectroscopy (Fig. 3a). To know whether this complex (**3**) is catalytically active or not, it was then tested as a catalyst for the reactions of the substrates (**1l**) and (**4a**), which afforded highly meta selective borylation with excellent conversions (Fig. 3b). Moreover, testing the stability of the catalyst [**3**: Ir(cod)(PY-PYRI)] we observed that it is highly stable that can even be stored in open air for several months.

To show insights into the reaction mechanism and the origin of meta-selectivity, detailed DFT calculations were conducted. The proposed reaction pathway is shown in Fig. 3c, in which **L6** was used as the model ligand. [Ir(OMe)(cod)]_2_ firstly reacts with the ligand and forms L6Ir(η^4^-cod) (**INT1**) as a starting species. When B_2_pin_2_ was the only boron reagent, a B-B oxidative addition to **INT1** occurs via **TS1**, giving L6Ir(η^4^-cod)(Bpin)_2_ (**INT2**). Then, unexpectedly, the oxygen in the ligand attacks a Bpin group on the iridium center and eventually undergoes an intramolecular Bpin-shift via **TS2**. With a free energy barrier of only 4.0 kcal/mol, this tautomerization step proceeds very quickly and delivers a more stable (L6Bpin)Ir(η^4^-cod)(Bpin) (**INT3**). Next, **INT3** partly dissociates cod to generate an empty site for incoming B_2_pin_2_, which then undergoes another B-B oxidative addition through **TS3** to form (L6Bpin)Ir(η^2^-cod)(Bpin)_3_ (**INT4**). Finally, the dissociation of cod yields (L6Bpin)Ir(Bpin)_3_ (**INT5**), which we believe to be the active catalyst in C-H borylation of the phenol silyl ethers. To support this Bpin-shift process, we prepared the intermediate ligand **L10** from the ligand **L9** with HBpin in presence of a catalytic amount of iridium catalyst (Fig. 3d). The intermediate ligand **L10** was then employed in the reaction using the substrate (**1l**) at room temperature that resulted in an improved result of 97% meta selectivity with full conversion to the borylated product (**2l**) (Fig. 3e). From these experimental observations, we realized that if we used catalytic amount of HBpin in the reaction mixture with ligand **L9**, it could give better selectivity and outcomes. As expected, we noticed that the use of ligand **L9** in presence of 5.0 mol% HBpin afforded 97% meta selectivity with 100% conversion of **1l** at room temperature (Fig. 3f).

**Fig. 4:**
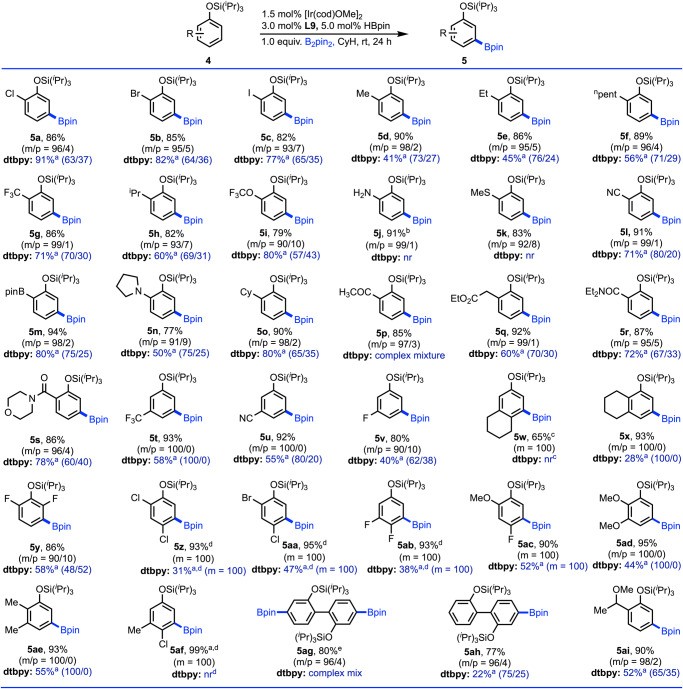
Substrates scope for substituted arenes. Reactions are in 0.5 mmol scale. ^a^Conversion was reported. ^b^1.5 equiv. B_2_pin_2_ was used. ^c^Reaction is carried out at 80 ^o^C. ^d^Reactions are carried out at 50 ^o^C. ^e^2.0 equiv. B_2_pin_2_ was used. See SI for details.

With the optimized reaction conditions of meta selective borylation using **L9** (PY-PYRI) as ligand and Si(^*i*^Pr)_3_ as steering group at room temperature, we next performed the iridium-catalyzed meta borylation of a variety of phenols that afforded excellent meta selectivity and yields of the isolated borylated products (Fig. 4). For example, we first tested 2-chlorophenol (**4a**) for the borylation reaction, while our designed ligand (**L9**) gave high meta selectivity (*m*/*p* = 96/4), traditional dtbpy ligand provided poor meta selectivity (*m*/*p* = 63/37), which clearly demonstrated the utility of the designed (**L9**: PY-PYRI) ligand. Other 2-substituted phenols, such as 2-bromo (**4b**) and 2-iodo (**4c**) afforded high meta selectivity that have great synthetic values owing to the two different types of handles on the phenols.

Likewise, phenols bearing various alkyl chain ranging from methyl to pentyl (**4d–4f**) at the 2-positions along with trifluoromethyl (**4g**), isopropyl (**4h**) and trifluoromethoxy (**4i**) smoothly underwent meta borylation irrespective of the nature of the substituent. Amino phenol (**4j**), substrate of momentous importance for the chemical and pharmaceutical industries^65^ is borylated with high meta selectivity (*m*/*p* = 99/1) without borylation next to the amino group, which is known to give ortho borylation under iridium-catalyzed borylation conditions via in situ generation of NHBpin group^66^. Thioether (**4k**) that usually directs borylation at the ortho position^67^ also underwent borylation with good meta selectivity. We observed that phenols containing functional groups such as cyano (**4l**) and Bpin (**4m**) resulted very high meta selectivity of 99% and 98% respectively without any disturbance by the electronics of these substituents. Also, cyclic amine (**4n**), cyclohexyl (**4o**), ketomethyl (**4p**) and homologous ester (**4r**) afforded high level of meta selectivity and tolerated well under the employed reaction conditions. The utility of the developed **PY-PYRI (L9)** ligand was further highlighted when substrates were employed in the reactions conditions with dtbpy ligand and all resulted in non-selective borylation reactions. Amide functionalities (**4r** and **4s**) that are known to undergo numerous synthetic transformations^68^ exhibited excellent meta selective borylation. Borylation of phenols having CF_3_ (**4g**) and CN (**4l**) substituents at the ortho position afforded exclusively meta borylation, the same substituents at the meta position of phenols (**4t** and **4u**) also gave meta selective borylation, which indicated the generality of the developed method. Moreover, fluoro-substituted arene, which typically gives borylation next to the fluorine atom under standard iridium-catalyzed conditions, in this case, 3-fluorophenol (**4v**) gave meta borylation as the major product. On the other hand, dtbpy gave non-selective borylation reaction of **4v** bearing meta fluoro substituents. Several disubstituted phenols (**4w–4af**) were also examined under the developed conditions that reacted smoothly to afford variously substituted meta borylated products in high yields. 2,2′-Biphenol, compound of paramount importance in medicinal chemistry as well as in chemical industry^69^ can selectively be mono- and diborylated (**5ah** and **5ag**) by tuning the amount of boron reagent. On the other hand, dtbpy gave complex mixture for the borylation of **4ag** and this result again highlighted the importance of **PY-PYRI** ligand. A bulky substituent at the ortho positions (**4ah**) did not hamper the reaction that gave 98% meta borylated product with 90% isolated yield.

**Fig. 5:**
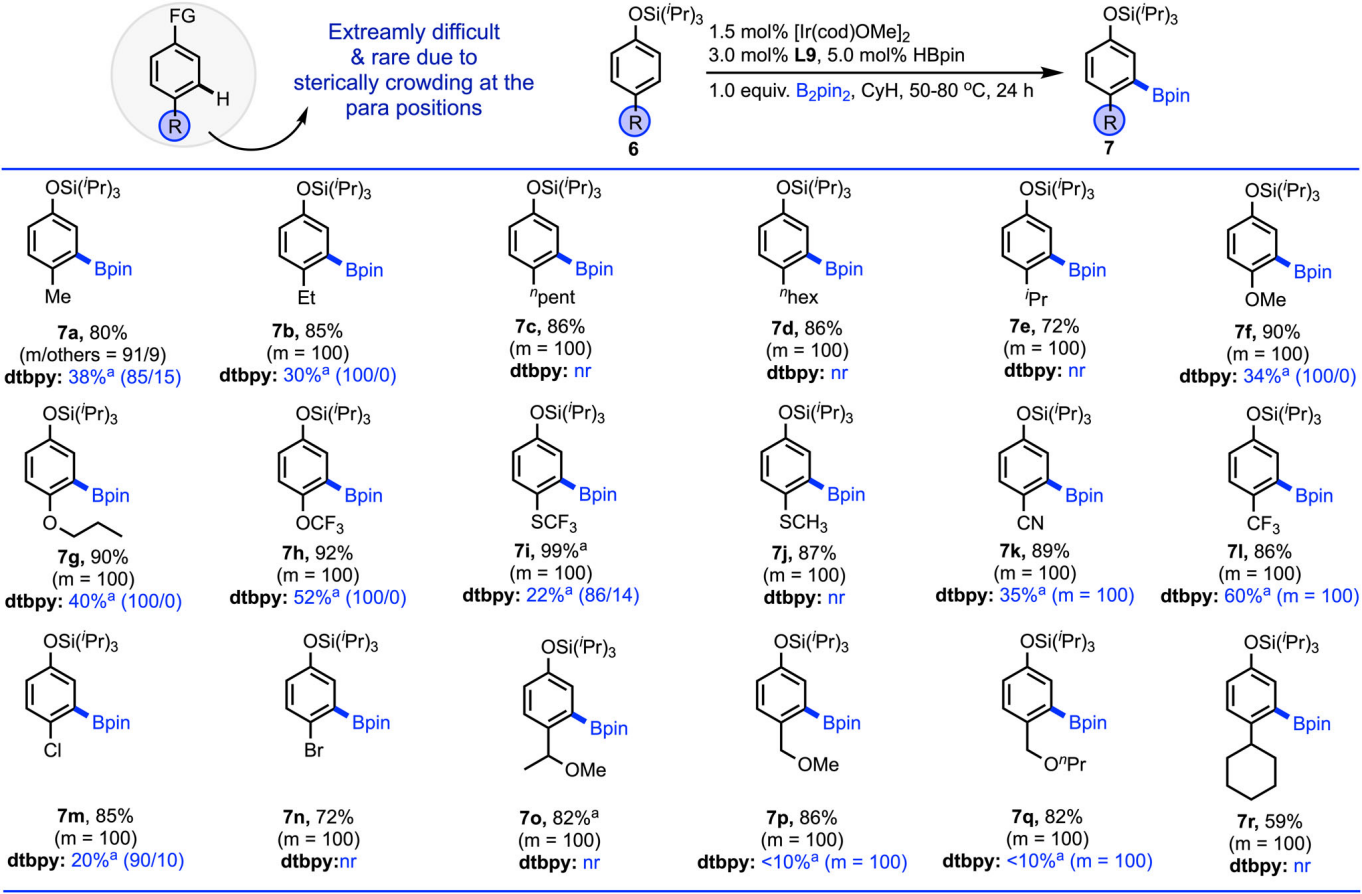
Substrates scope for the 4-substituted arenes. Reactions are in 0.5 mmol scale. ^a^Conversions were reported. See SI for details.

Next, we focused on the meta borylation of those phenols bearing a substituent at the para position (Fig. 5). Because, borylation at the remote meta position in presence of a para substituent remains an extraordinary challenge due to the steric reason. Moreover, we selected those substituents at the para position that already provided exclusive meta borylation of phenols when they were located at either ortho or meta positions. The reason for this selection is mainly to observe the overall effects of the borylation by the same substituents. For the testification, we begun with the 4-methyl phenol (**6a**) that afforded 91% meta selective borylation. Increasing the chain length from small methyl group to the relatively bulkier alkyl groups such as, ethyl (**6b**), pentyl (**6c**), hexyl (**6d**) and isopropyl (**6e**), the borylation underwent smoothly with further enhancement of the meta selectivity from 91% to 100%. Para-substituted ethers and thioethers bearing electronically different substituents (**6f–6j**) reacted with 100% meta selectivity, which revealed that the scope of the meta borylation is very general regardless of the nature of the substituents. While 2-CN, 3-CN as well as 2-CF_3_ and 3-CF_3_ bearing phenols resulted in excellent meta borylation, the same substituents at the para position reacted to yield 100% meta borylation. Likewise, we also observed that chloro (**6m**) and bromo (**6n**) containing phenols reacted to give the meta borylation products solely irrespective of their position in the arene. Moreover, it has been found that the phenols featuring bulky substituents at the para position (**6o–6r**) also gave exclusively meta borylation, although conversion was moderate in case of the cyclohexyl group. In all cases, dtbpy results were compared and gave somewhat bad result with compared to developed **PY-PYRI (L9)** ligand.

**Fig. 6:**
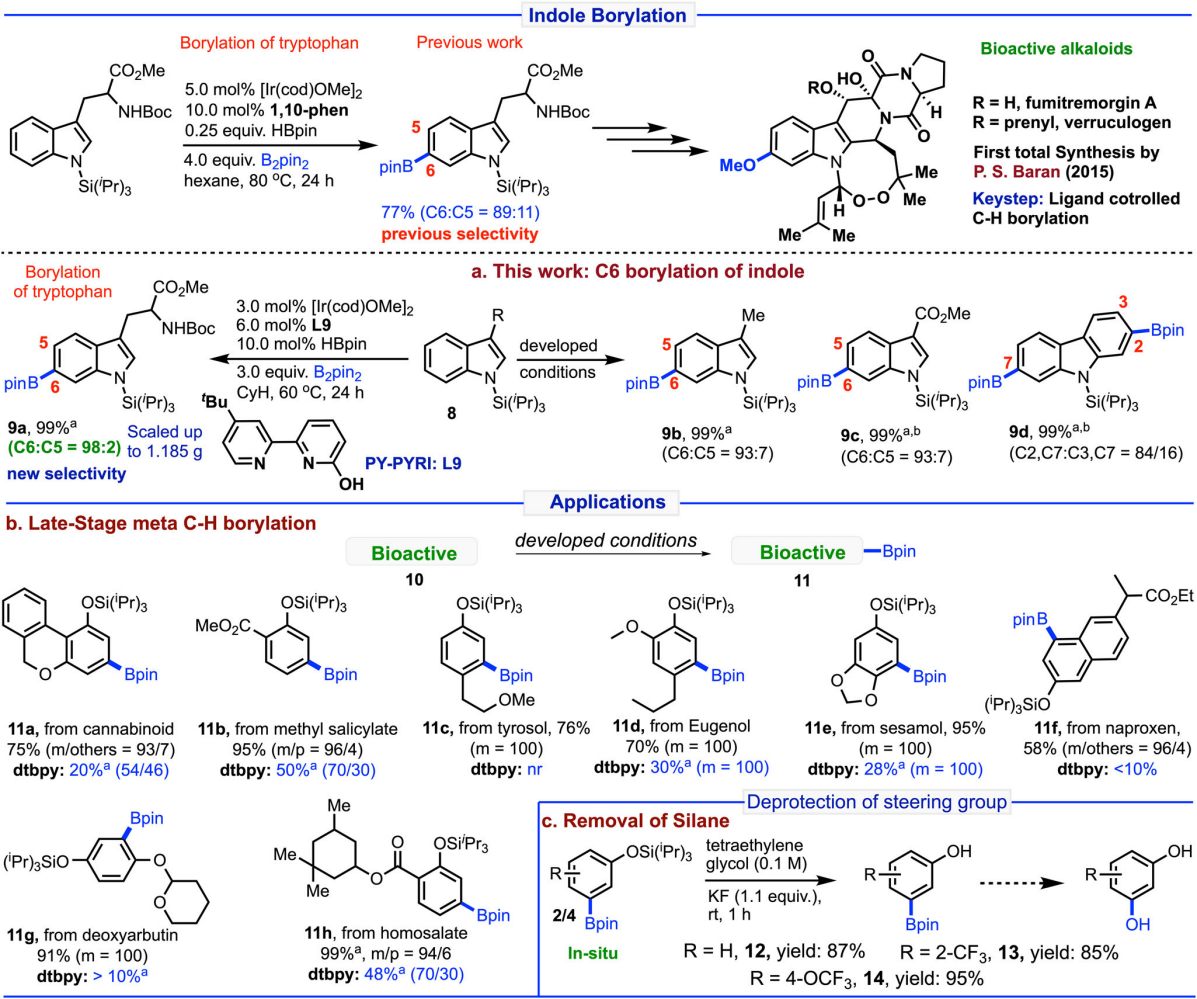
Borylation of indoles previous and present reports and applications. **a** C6 borylation of indoles. **b** Late-stage meta C–H borylation. **c** Removal of silane. Reactions are in 0.5 mmol scale. ^a^Conversions were reported. ^b^2.0 equiv. B_2_pin_2_. See SI for details.

In 2015, Baran et al. reported^70^ the first total synthesis of Verruculogen and Fumitremorgin A enabled by ligand-controlled C–H borylation as the key step of TIPS protected tryptophan. We were curious if our designed ligand system could provide the remote C6 borylation of TIPS protected indoles and tryptophan (Fig. 6a). For that reason, we performed borylation of TIPS-protected tryptophan (**8a**) (synthetic key precursor of bioactive alkaloids Verruculogen and Fumitremorgin A) which provided C6 borylation with 98% selectivity with excellent conversions at 60 °C. Moreover, large-scale synthesis of (**8a**) smoothly underwent under the developed reaction conditions without hampering the selectivity. We also found that other indole derivatives (**8b** and **8c**) and carbazole (**8d**) easily underwent remote borylation affording excellent selectivity and conversion. This developed method provided a simple way to borylate the 3-substituted indoles derivatives that might be beneficial for the total synthesis or the late-stage functionalization of several bioactive molecules.

Late-stage functionalization^71^ of complex bioactive and medicinally important molecules by the site selective C–H activation is a powerful method for the development of new drug candidates^72^. In this context, introducing a boron functionality into the bioactive and medicinally important molecules would further enhance the identification of new lead molecules not only for the enormous importance of the boron-bearing small molecules^73^ but also for the uniqueness of the boron group towards the diverse derivatization towards numerous other functional groups. Thus, we tested our developed method for several commercially available bioactive and drug molecules (Fig. 6b). For example, cannabinoid core (**10a:** used as a psychoactive drug), methyl salicylate derivatives (**10b:** an anti-inflammatory and analgesic agent), tyrosol derivatives (**10c:** an antioxidant), eugenol derivatives (**10d:** a flavoring agent), sesamol derivatives (**10e:** an antioxidant), naproxen derivatives (**10f:** a nonsteroidal anti-inflammatory drug, NSAID), deoxyarbutin derivatives (**10g:** used for treatment of hyperpigmentation disorders) and homosalate (**10h**: used as a sunscreen) were meta borylated with high yield and selectivity. Moreover, parallel reactions were carried out with the dtbpy ligand for **10a–h** substrates and resulted comparatively less conversions or non-selective borylation reactions than the developed **PY-PYRI** (**L9**) ligand. The steering silane group from the borylated phenols has been removed under a very mild reaction conditions at room temperature (ethylene glycol, KF, 1 h) that afforded the meta borylated phenols in high yields (Fig. 6c). Notably, the meta borylated phenols can further be transformed to a number of substituted phenols/resorcinols that are difficult to prepare by otherwise.

**Fig. 7:**
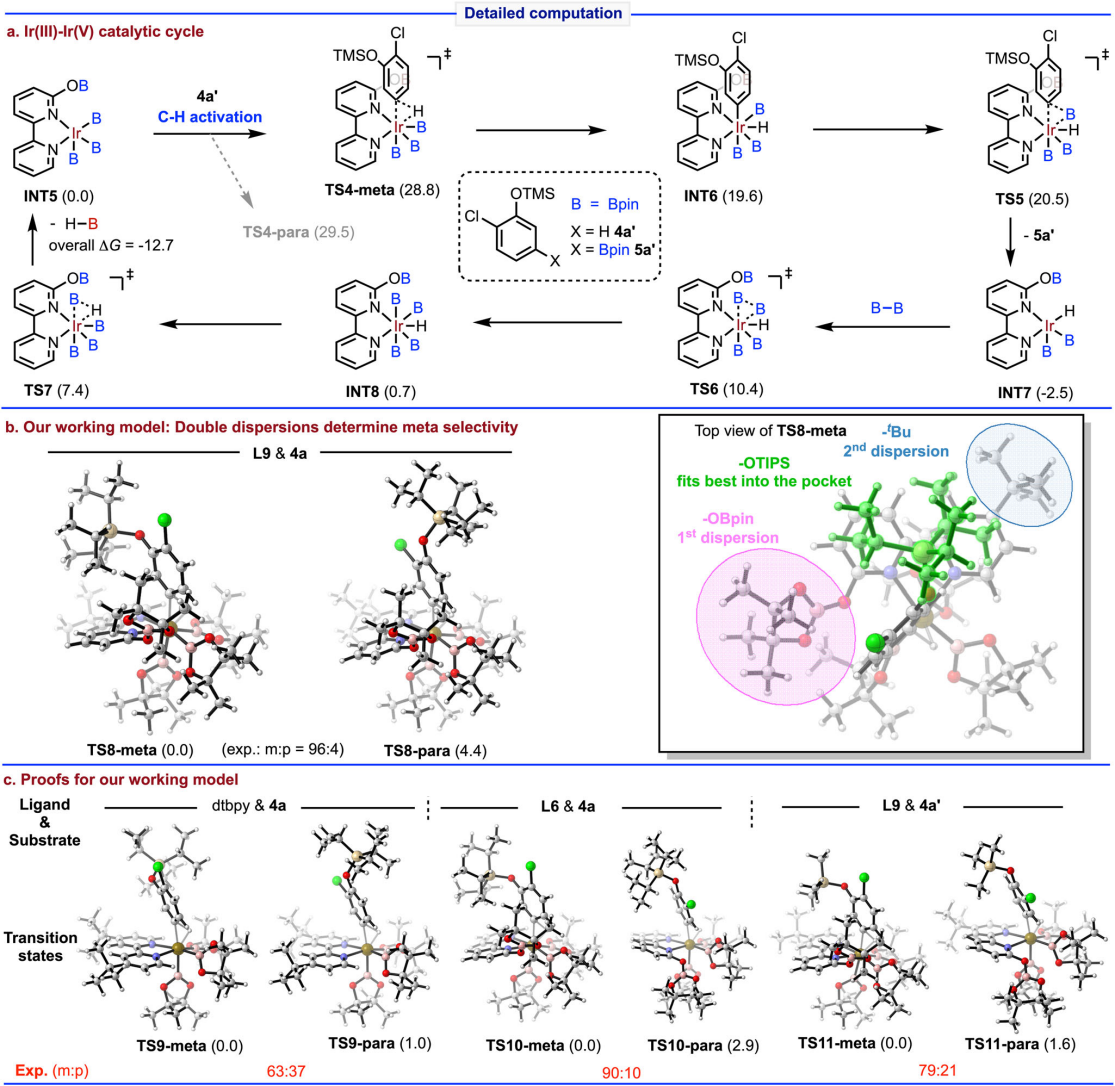
Detailed computation. **a** Ir(III)–Ir(V) catalytic cycle. **b** Our working model: Double dispersions determine meta selectivity. **c** Proofs for our working model. Computed at SMD(CyH)-M06/6-311 + G(d,p)//B3LYP/6-31 G(d). Relative free energies are given in kcal/mol. Atom colors in graphics: gray, C; white, H; red, O; blue, N; pink, B; light yellow, Si; green, Cl; brown, Ir.

At the end, we are curious about the origin of meta selectivity in the C-H borylation. Therefore, a detailed DFT calculations were carried out. It is found that the active catalyst **INT5** follows an Ir(III)-Ir(V) catalytic cycle through C-H oxidative addition (via **TS4-meta**), C-B reductive elimination (via **TS5**), and catalyst regeneration (from **INT7** to **INT5**), which is similar to those reported for bipyridine ligands (Fig. 7a)^57,74^. An evaluation of the regioselectivity-determining C-H activation process showed that the meta-C-H activation of model substrate **4a’** (2Cl-PhOSiMe_3_) through **TS4-meta** is preferred by 0.7 kcal/mol over its para-C-H activation through **TS4-para**. This tendency agrees with our experiments. The activation free energy of the rate-determining C-H activation is computed to be 28.8 kcal/mol. Since dispersions can significantly stabilize the meta-C-H activation transition state (vide infra), the barrier for the real system is expected to be lower by 4–5 kcal/mol, making the reaction smoothly occur at room temperature.

Furthermore, the real ligand and substrate (**L9** and **4a**) were evaluated. Firstly, the transition states of meta- and para-C-H activation (**TS8-meta** and **TS8-para**) were located (Fig. 7b). To our delight, a much larger energetic preference of 4.4 kcal/mol was found for meta-C-H activation, in accordance with the observed excellent meta-selectivity. Scrutinizing the structures, **TS8-para** seems not to benefit from any significant interaction. However, for **TS8-meta**, we found that a double-dispersion model may explain the meta-selectivity (Fig. 7b, right). The OBpin group (highlighted in pink) in the ligand lies near the substrate, providing the first dispersion with the silyl group. In addition, the *tert*-butyl group (highlighted in blue) exerts the second dispersion on the other side. The Si(^*i*^Pr)_3_ group (highlighted in green) has the best volume to fit into the constructed pocket, maximizing both dispersions. The multiple C-H···π interactions with the bipyridine rings may also help.

Our double-dispersion working model can be further supported by comparison with analogous ligands (Fig. 7c). The importance of the OBpin-originated first dispersion can be proved by considering the typical dtbpy as ligand. The computation showed that the silyl group lies far away from the ligand in **TS9-meta** without any dispersion mentioned above. Accordingly, an energetic preference of only 1.0 kcal/mol was predicted for meta-borylation, correlating with the poor experimental result (*m*:*p* = 63:37). The function of *tert*-butyl is verified by using **L6** as ligand. The meta-C-H activation transition state **TS10-meta** has the same structural pattern as **TS8-meta**, showing the existence of the first dispersion. However, without the *tert*-butyl group, the second dispersion is absent, and the energy difference between the meta- and para-C-H activation shrinks to 2.9 kcal/mol. Experimentally, the meta-selectivity drops to 90:10. Finally, substrate **4a’**, with a smaller silyl group, was calculated for reaction using ligand **L9**. The silyl group in **TS11-meta** is also placed as the same orientation as in **TS8-meta**, but the suboptimal size fitting results in a decreased energy difference of 1.6 kcal/mol, in agreement of the experiment (*m*:*p* = 79:21). In addition, more computational studies using different DFT methods are performed. When B3LYP (without dispersion correction) is used for single-point energy calculations, the computation contradicts with the experiments; when other functionals with dispersion terms are used, the results agree with the experiments (for details, see Supplementary Fig. 23). This further demonstrates the importance of the dispersion effect in the reaction system. Therefore, the synergistic effect of two optimal dispersions contributes to the observed excellent meta-selectivity.

## Discussion

In conclusion, we report a new class of ligand and catalyst that has demonstrated remarkable efficiency for the remote meta selective borylation of phenols featuring all types of substitutions at the arene ring. In addition, we have seen that our developed ligand system is beneficial for the remote C6-borylation of indole derivatives including tryptophan which is a synthetic precursor of bioactive alkaloids (Verruculogen and Fumitremorgin A). Several late-stage meta borylations have been showcased with bioactive and drug molecules that might be useful for repurposing medicines and identification of new lead drug candidates. Notably, while preliminary experimental findings indicated that a secondary O–Si interaction was the key element that controls the remote meta selectivity, detailed computational investigations along with more detailed experimental results revealed that an unprecedented Bpin shift was observed during the transformation of iridium bis(boryl) complex to iridium tris(boryl) complex and developed tautomeric ligand behaves like bipyridine during the borylation reaction. The governing factors for the meta selective borylation is the combined dispersion of OBpin and *tert*-butyl group of the designed ligand which make a suitable pocket for the phenol bearing tri-isopropyl silane group that resulted in meta selective borylation of phenol. We anticipate that the designed ligand and catalyst will also find wide application in the context of other C–H functionalization reactions.

## Methods

### Experimental procedure: iridium-catalyzed meta-C-H borylation

In an argon-filled glove box, a 5.0 mL Wheaton microreactor was charged with [Ir(cod)OMe]_2_ (4.97 mg, 1.5 mol%), ligand **L9** (3.4 mg, 3.0 mol%), B_2_pin_2_ (127.0 mg, 1.0 equiv.), HBpin (3.2 mg, 5.0 mol%) and dry cyclohexane (2.0 mL) were added sequentially. The reaction mixture was stirred for 2 min at room temperature and then substrate (0.5 mmol) was added. The microreactor was capped with a Teflon pressure cap and stirred for 24 h at a particular mentioned temperature. After completion (judged by GC-MS), CyH was removed under reduced pressure and chromatographic separation with silica gel gave the meta-borylated product.

### Supplementary information

Supplementary Information

Peer Review File

### Source data

Source Data

## Data Availability

All data are available from the corresponding authors upon request. X-ray dataset for catalyst 3 is freely available at the Cambridge Crystallographic Data Centre under deposition number CCDC 2180880. All relevant data are available in this article and its Supplementary Information. The experimental procedures and characterization of all new compounds are provided in Supplementary Information file. Coordinates of the optimized structures are provided as source data. Source data are provided with this paper.

## References

[1] Zhang Z, Tanaka K, Yu J-Q (2017). Remote site-selective C–H activation directed by a catalytic bifunctional template. Nature.

[2] Luo J, Preciado S, Larrosa I (2014). Overriding ortho-para selectivity via a traceless directing group relay strategy: the meta-selective arylation of phenols. J. Am. Chem. Soc..

[3] Sinha SK (2022). Toolbox for distal C–H bond functionalizations in organic molecules. Chem. Rev..

[4] Lyons TW, Sanford MS (2010). Palladium-catalyzed ligand-directed C–H functionalization reactions. Chem. Rev..

[5] Davies HML, Bois JD, Yu J-Q (2011). C–H functionalization in organic synthesis. Chem. Soc. Rev..

[6] Arockiam PB, Bruneau C, Dixneuf PH (2012). Ruthenium (II)-catalyzed C–H bond activation and functionalization. Chem. Rev..

[7] Sambiagio C (2018). A comprehensive overview of directing groups applied in metal-catalysed C–H functionalisation chemistry. Chem. Soc. Rev..

[8] Crabtree RH, Lei A (2017). Introduction: CH activation. Chem. Rev..

[9] Shilov AE, Shul’pin GB (1997). Activation of C−H bonds by metal complexes. Chem. Rev..

[10] Rogge, T. et al. C–H activation. *Nat. Rev. Methods Primers*10.1038/s43586-021-00041-2 (2021).

[11] Yamaguchi J, Yamaguchi AD, Itami K (2012). C-H bond functionalization: emerging synthetic tools for natural products and pharmaceuticals. Angew. Chem. Int. Ed..

[12] Dalton T, Faber T, Glorius F (2021). C–H activation: toward sustainability and applications. ACS Cent. Sci..

[13] Liao K (2018). Design of catalysts for site-selective and enantioselective functionalization of non-activated primary C–H bonds. Nat. Chem..

[14] Hoque ME, Hassan MMM, Chattopadhyay B (2021). Remarkably efficient iridium catalysts for directed C(sp2)–H and C(sp3)–H borylation of diverse classes of substrates. J. Am. Chem. Soc..

[15] Hoveyda AH, Evans DA, Fu GC (1993). Substrate-directable chemical reactions. Chem. Rev..

[16] Cheng C, Hartwig JF (2014). Rhodium-catalyzed intermolecular C–H silylation of arenes with high steric regiocontrol. Science.

[17] Ramadoss B, Jin Y, Asako S, Ilies L (2022). Remote steric control for undirected meta-selective C–H activation of arenes. Science.

[18] Cho JY, Tse MK, Holmes D, Maleczka RE, Smith MR (2002). Remarkably selective iridium catalysts for the elaboration of aromatic C-H bonds. Science.

[19] Saito Y, Segawa Y, Itami K (2015). para-C–H borylation of benzene derivatives by a bulky iridium catalyst. J. Am. Chem. Soc..

[20] Mondal A, Chen H, Flämig L, Wedi P, Gemmeren MV (2019). Sterically controlled late-stage C–H alkynylation of arenes. J. Am. Chem. Soc..

[21] Kuninobu Y, Ida H, Nishi M, Kanai M (2015). A meta-selective C-H borylation directed by a secondary interaction between ligand and substrate. Nat. Chem..

[22] Fanourakis A, Docherty PJ, Chuentragool P, Phipps RJ (2020). Recent developments in enantioselective transition metal catalysis featuring attractive noncovalent interactions between ligand and substrate. ACS Catal..

[23] Zhang T (2021). A directive Ni catalyst overrides conventional site selectivity in pyridine C–H alkenylation. Nat. Chem..

[24] Hoque ME, Bisht R, Haldar C, Chattopadhyay B (2017). Noncovalent interactions in Ir-catalyzed C–H activation: L-shaped ligand for para-selective borylation of aromatic esters. J. Am. Chem. Soc..

[25] Dydio P, Reek JNH (2014). Supramolecular control of selectivity in transitionmetal catalysis through substrate preorganization. Chem. Sci..

[26] Lou Y, Wei J, Li M, Zhu Y (2022). Distal ionic substrate–catalyst interactions enable long-range stereocontrol: access to remote quaternary stereocenters through a desymmetrizing Suzuki–Miyaura reaction. J. Am. Chem. Soc..

[27] Bisht R (2022). Metal-catalysed C–H bond activation and borylation. Chem. Soc. Rev..

[28] Ros A, Fernandez R, Lassaletta JM (2014). Functional group directed C–H borylation. Chem. Soc. Rev..

[29] Kawamorita S, Ohmiya H, Hara K, Fukuoka A, Sawamura M (2019). Directed ortho borylation of functionalized arenes catalyzed by a silica-supported compact phosphine−iridium system. J. Am. Chem. Soc..

[30] Boebel TA, Hartwig JF (2008). Silyl-directed, iridium-catalyzed ortho-borylation of arenes. a one-pot ortho-borylation of phenols, arylamines, and alkylarenes. J. Am. Chem. Soc..

[31] Bisht R, Chattopadhyay B (2016). Formal Ir-catalyzed ligand-enabled ortho and meta borylation of aromatic aldehydes via in situ-generated imines. J. Am. Chem. Soc..

[32] Yang L, Uemura N, Nakao Y (2019). meta-Selective C–H borylation of benzamides and pyridines by an iridium–lewis acid bifunctional catalyst. J. Am. Chem. Soc..

[33] Davis HJ, Madalina MT, Phipps RJ (2016). Ion pair-directed regiocontrol in transition-metal catalysis: a meta-selective C–H borylation of aromatic quaternary ammonium salts. J. Am. Chem. Soc..

[34] Chaturvedi J, Haldar C, Bisht R, Pandey G, Chattopadhyay B (2021). Meta selective C– H borylation of sterically biased and unbiased substrates directed by electrostatic interaction. J. Am. Chem. Soc..

[35] Mihai MT, Williams BD, Phipps RJ (2019). Para-selective C-H borylation of common arene building blocks enabled by ion-pairing with a bulky countercation. J. Am. Chem. Soc..

[36] Bastidas JRM, Oleskey TJ, Miller SL, Smith MR, Maleczka RE (2019). Para-selective, iridium-catalyzed C–H borylations of sulfated phenols, benzyl alcohols, and anilines directed by ion-pair electrostatic interactions. J. Am. Chem. Soc..

[37] Chang W (2022). Computationally designed ligands enable tunable borylation of remote C-H bonds in arenes. Chem..

[38] Wang Y (2022). Diversification of aryl sulfonyl compounds through ligand controlled meta- and para-CH borylation. Angew. Chem. Int. Ed..

[39] Lu S (2022). para-Selective C–H borylation of aromatic quaternary ammonium and phosphonium salts. Angew. Chem. Int. Ed..

[40] Engle KM, Mei T, Wasa M, Yu J-Q (2012). Weak Coordination as a powerful means for developing broadly useful C–H functionalization reactions. Acc. Chem. Res..

[41] Leow D, Li G, Mei T-S, Yu J-Q (2012). Activation of remote meta-C–H bonds assisted by an end-on template. Nature.

[42] Shi H, Herron AN, Shao Y, Shao Q, Yu J-Q (2018). Enantioselective remote meta-C–H arylation and alkylation via a chiral transient mediator. Nature.

[43] Gandeepan P, Ackermann L (2018). Transient directing groups for transformative C–H activation by synergistic metal catalysis. Chem.

[44] Meng G (2020). Achieving site-selectivity for C–H activation processes based on distance and geometry: a carpenter’s approach. J. Am. Chem. Soc..

[45] Scott KA, Cox PB, Njardarson JT (2022). Phenols in pharmaceuticals: analysis of a recurring motif. J. Med. Chem..

[46] Bartolomei B, Gentile G, Rosso C, Filippini G, Prato M (2021). Turning the light on phenols: new opportunities in organic synthesis. Chem. Eur. J..

[47] Quideau, S., Deffieux, D., Douat-Casassus, C. & Pouységu, L. Plant polyphenols: chemical properties, biological activities, and synthesis *Angew. Chem. Int. Ed*. **50**, 586–621 (2011). 10.1002/anie.201000044 21226137

[48] Huang Z, Lumb J-P (2019). Phenol-directed C–H functionalization. ACS Catal..

[49] Maraswami M, Hirao H, Loh T-P (2021). Copper-catalyzed meta-selective arylation of phenol derivatives: an easy access to m-aryl phenols. ACS Catal..

[50] Dai H-X, Li G, Zhang X-G, Stepan AF, Yu J-Q (2013). Pd(II)-catalyzed ortho- or meta-C–H olefination of phenol derivatives. J. Am. Chem. Soc..

[51] Wan L, Dastbaravardeh N, Li G, Yu J-Q (2013). Cross-coupling of remote meta-C–H bonds directed by a U-shaped template. J. Am. Chem. Soc..

[52] Xu J (2019). Sequential functionalization of meta-C–H and ipso-C–O bonds of phenols. J. Am. Chem. Soc..

[53] Iverson, C. N. & Smith, M. R. III. Stoichiometric and catalytic B-C bond formation from unactivated hydrocarbons and boranes. *J. Am. Chem. Soc*. **121**, 7696–7697 (1999).

[54] Ishiyama T (2002). Mild iridium-catalyzed borylation of arenes. High turnover numbers, room temperature reactions, and isolation of a potential intermediate. J. Am. Chem. Soc..

[55] Mkhalid IAI, Barnard JH, Marder TB, Murphy JM, Hartwig JF (2010). C–H activation for the construction of C–B bonds. Chem. Rev..

[56] Hartwig JF (2011). Regioselectivity of the borylation of alkanes and arenes. Chem. Soc. Rev..

[57] Boller TM (2005). Mechanism of the mild functionalization of arenes by diboron reagents catalyzed by iridium complexes. Intermediacy and chemistry of bipyridine-ligated iridium trisboryl complexes. J. Am. Chem. Soc..

[58] Haldar C, Hoque ME, Chaturvedi J, Hassan MMM, Chattopadhyay B (2021). Ir-catalyzed proximal and distal C–H borylation of arenes. Chem. Commun..

[59] Lazareva NF, Sterkhova IV, Vashchenko AV (2021). N-[difluoro(methyl)silyl]carboxamides: synthesis, structural features and theoretical estimating of Si←O dative bond energy. J. Mol. Struct..

[60] Sohail M (2013). Synthesis and hydrolysis–condensation study of water-soluble self-assembled pentacoordinate polysilylamides. Organometallics.

[61] Li Z (2021). A tautomeric ligand enables directed C‒H hydroxylation with molecular oxygen. Science.

[62] Chan HSS, Yang J-M, Yu J-Q (2022). Catalyst-controlled site-selective methylene C–H lactonization of dicarboxylic acids. Science.

[63] Wang Z (2021). Ligand-controlled divergent dehydrogenative reactions of carboxylic acids via C–H activation. Science.

[64] Li Z, Park HS, Qiao JX, Kap-Sun Yeung K-S, Yu J-Q (2022). Ligand-enabled C–H hydroxylation with aqueous H_2_O_2_ at room temperature. J. Am. Chem. Soc..

[65] Lajiness JP (2010). Design, synthesis, and evaluation of duocarmycin O-amino phenol prodrugs subject to tunable reductive activation. J. Med. Chem..

[66] Preshlock SM (2013). A traceless directing group for C-H borylation. Angew. Chem. Int. Ed..

[67] Li HL, Kuninobu Y, Kanai M (2017). Lewis acid-base interaction- controlled ortho-selective C-H borylation of aryl sulfides. Angew. Chem. Int. Ed..

[68] Sun W (2020). Chemodivergent transformations of amides using gem-diborylalkanes as pro-nucleophiles. Nat. Commun..

[69] Hua Z, Vassar VC, Choi H, Ojima I (2004). New biphenol-based, fine-tunable monodentate phosphoramidite ligands for catalytic asymmetric transformations. Proc. Natl Acad. Sci. USA.

[70] Feng Y (2015). Total synthesis of verruculogen and fumitremorgin A enabled by ligand-controlled C–H borylation. J. Am. Chem. Soc..

[71] Zhang L, Ritter T (2022). A perspective on late-stage aromatic C–H bond functionalization. J. Am. Chem. Soc..

[72] Guillemard L, Kaplaneris N, Ackermann L, Johansson MJ (2021). Late-stage C–H functionalization offers new opportunities in drug discovery. Nat. Rev. Chem..

[73] Thareja S, Zhu M, Ji X, Wang B (2017). Boron-based small molecules in disease detection and treatment (2013 -2016). Heterocyl. Commun..

[74] Tamura H, Yamazaki H, Sato H, Sakaki S (2003). Iridium-catalyzed borylation of benzene with diboron. Theoretical elucidation of catalytic cycle including unusual iridium(V) intermediate. J. Am. Chem. Soc..

