# Supplementary material for: A tautomerized ligand enabled meta selective C–H borylation of phenol
Source: Nat Commun. 2023 Oct 30;14:6906. doi: 10.1038/s41467-023-42310-6 (PMC10616221; doi:10.1038/s41467-023-42310-6)
Supplement: Supplementary file 1 — Supplementary Information [file 41467_2023_42310_MOESM1_ESM.pdf]

Supplementary Information

A Tautomerized Ligand Enabled Meta Selective C–H Borylation of Phenol

Saikat Guria[1], Mirja Md Mahamudul Hassan[1], Jiawei Ma[2], Sayan Dey[1], Yong Liang[2]*, Buddhadeb Chattopadhyay[1]*

[1]Department of Biological & Synthetic Chemistry, Centre of Biomedical Research, SGPGIMS Campus, Raebareli Road, Lucknow 226014, Uttar Pradesh, India

[2]State Key Laboratory of Coordination Chemistry, Jiangsu Key Laboratory of Advanced Organic Materials, Chemistry and Biomedicine Innovation Center, School of Chemistry and Chemical Engineering, Nanjing University, Nanjing 210023, China

*Correspondence to: buddhadeb.c@cbmr.res.in, yongliang@nju.edu.cn

Contents

General Information S2

Preparation of Starting Materials S3

Preparation of Ligands S65

Preparation of Catalyst **3** S88

Reactivity test of catalyst **3** S94

Reactivity of catalyst 10 after kept in air for 30 days S96

Preparation of **L10** S97

Test of reactivity of **L10** S98

Meta-Borylation of Substituted Arenes S102

Meta-Borylation of 4-substituted arenes S131

C6-Borylation of Indoles S148

Late-stage Meta C-H Borylation of Bio-active Molecules S153

Procedure of insitu triisopropylsilyl group deprotection S160

Computational Details S163

NMR spectra S169

References S416

General Information:

All commercially available chemicals were used as received unless otherwise indicated. Pinacolborane (HBpin) and bis(pinacolato)diboron (B_2pin_2) were procured from A. K. Scientific. Bis(1,5-cyclooctadiene)di-µ-methoxy-diiridium(I)([Ir(OMe)(cod)]$_2$) was procured from Sigma-Aldrich. Tetrahydrofuran (THF), *m*-xylene, toluene, hexane and cyclohexane were refluxed over sodium/benzophenone ketyl, distilled and degassed twice before reaction. Dichloromethane (DCM) and dimethylformamide (DMF) were distilled over CaH_2. Column chromatography was performed on flash silica gel (ACME). Thin layer chromatography was performed on 0.25 mm thick aluminum-backed silica gel plates purchased from Merck and visualized with ultraviolet light (λ = 254 nm). ^{1}H, ^{13}C and ^{11}B-NMR spectra were recorded on Bruker 400 MHz NMR spectrometer.

The boron bearing carbon atom was not observed due to quadrupolar relaxation. Due to relaxation problem few carbon peaks are missing from the borylated products. All coupling constants (J) are apparent, J values measured at the indicated field strengths in Hertz (s = singlet, d = doublet, t = triplet, q = quartet, dd = doublet of doublets, bs = broad singlet). High-resolution mass spectra (HRMS) were obtained at the Centre of Biomedical Research Mass Spectrometry Service Center using a Waters GCT Premier instrument run on electron ionization (EI) direct probe or a Waters QTOF Ultima instrument run on electrospray ionization (ESI). GC/MS (Agilent Technology) was obtained from Centre of Biomedical Research Institute and for the analysis RAM temperature was used 50 ^{o}C for each sample.

One X-ray crystal structures in the manuscript have been deposited in the CCDC with numbers 2180880. Single crystal X-ray structural studies were performed on a CCD Bruker SMART APEX diffractometer equipped with an Oxford instruments low–temperature attachment.

Experimental Section

A. Preparation of Starting Materials

Synthesis of phenyl acetate **(1d)***:*

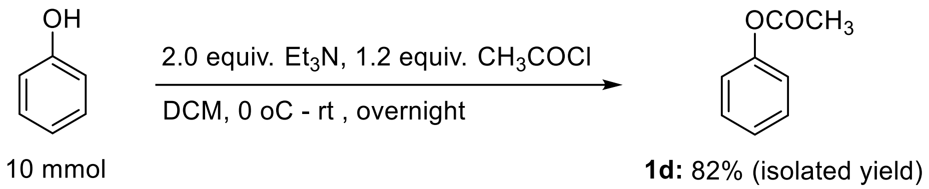

In a dry 100 mL round-bottomed flask, phenol (0.94 g, 10 mmol) was dissolved in 20 mL DCM, Et_3N (2.8 mL, 20 mmol) was added and cooled to 0 ºC. To that Acetyl chloride (0.85 mL, 12 mmol) was added dropwise via syringe and stirred at room temperature overnight. After completion (judged by TLC), quenched with water (30 mL) and extracted with DCM (50 mL x 3). The combined organic phase washed with 5% Sodium bicarbonate (50 mL), water (30 mL x 3), brine (50 mL) successively and dried over anhydrous Na_2SO_4, filtered and concentrated under reduced pressure. The resulting mixture was purified by silica gel chromatography (10% ethyl acetate in hexane as eluent) gave 1.1 g (82%) of phenyl acetate **(1d)** as a colourless liquid. Spectral data are in accordance with the reported data.[1]

Synthesis of phenyl pivalate **(1e)***:*

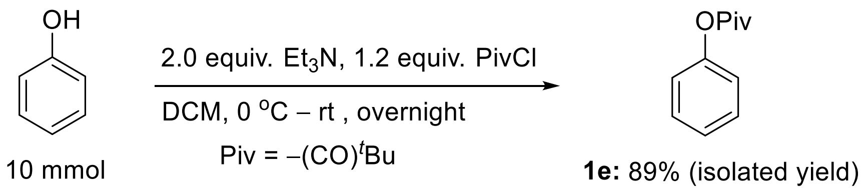

In a dry 100 mL round-bottomed flask, phenol (0.94 g, 10 mmol) was dissolved in 20 mL DCM, Et_3N (2.8 mL, 20 mmol) was added and cooled to 0 ºC. To that Pivaloyl chloride (1.5 mL, 12 mmol) was added dropwise via syringe and stirred at room temperature overnight. After completion (judged by TLC), quenched with water (30 mL) and extracted with DCM (50 mL x 3). The combined organic phase washed with 5% Sodium bicarbonate (50 mL), water (30 mL x 3), brine (50 mL) successively and dried over anhydrous Na_2SO_4, filtered and concentrated under reduced pressure. The resulting mixture was purified by silica gel chromatography (10% ethyl acetate in hexane as eluent) gave 1.58 g (89%) of phenyl pivalate **(1e)** as a colourless liquid. Spectral data are in accordance with the reported data.[2]

Synthesis of tert-butyl phenyl carbonate **(1f)**:

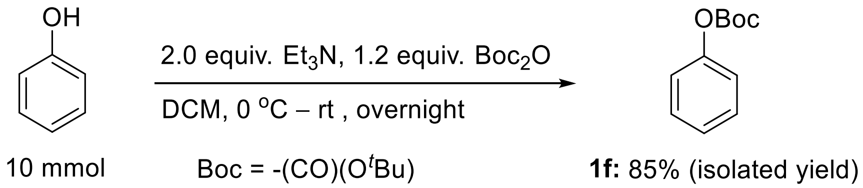

In a dry 100 mL round-bottomed flask, phenol (0.94 g, 10 mmol) was dissolved in 20 mL DCM and Et_3N (2.8 mL, 20 mmol) was added and cooled to 0 ºC. To that Di-tert-butyl dicarbonate (2.76 mL, 12 mmol) was added dropwise via syringe and stirred at room temperature overnight. After completion (judged by TLC), quenched with water (30 mL) and extracted with DCM (50 mL x 3). The combined organic phase washed with 5% Sodium bicarbonate (50 mL), water (30 mL x 3), brine (50 mL) successively and dried over anhydrous Na_2SO_4, filtered and concentrated under reduced pressure. The resulting mixture was purified by silica gel chromatography (5% ethyl acetate in hexane as eluent) gave 1.65 g (85%) of tert-butyl phenyl carbonate **(1f)** as a colourless liquid. Spectral data are in accordance with the reported data.[3]

Synthesis of ethyl phenyl carbonate **(1g)**:

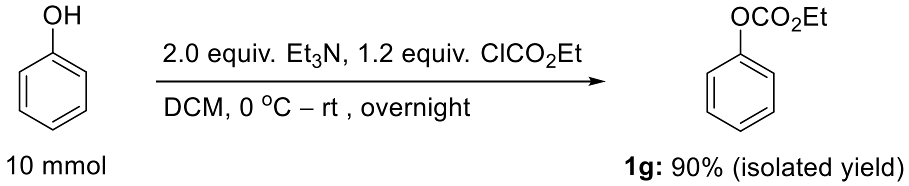

In a dry 100 mL round-bottomed flask, phenol (0.94 g, 10 mmol) was dissolved in 20 mL DCM and Et_3N (2.8 mL, 20 mmol) was added and cooled to 0 ºC. To that Ethyl chloroformate (1.2 mL, 12 mmol) was added dropwise via syringe and stirred at room temperature overnight. After completion (judged by TLC), quenched with water (30 mL) and extracted with DCM (50 mL x 3). The combined organic phase washed with 5% Sodium bicarbonate (50 mL), water (30 mL x 3), brine (50 mL) successively and dried over anhydrous Na_2SO_4, filtered and concentrated under reduced pressure. The resulting mixture was purified by silica gel chromatography (10% ethyl acetate in hexane as eluent) gave 1.49 g (90%) of ethyl phenyl carbonate **(1g)** as a colourless liquid. Spectral data are in accordance with the reported data.[4]

Synthesis of phenyl diethylcarbamate **(1h)***:*

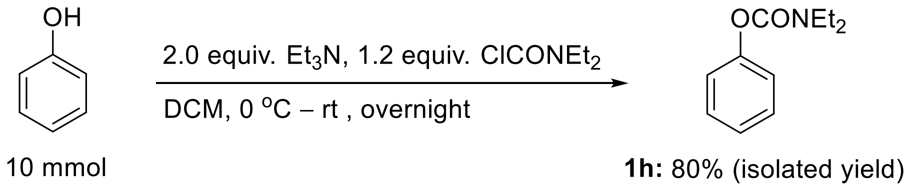

In a dry 100 mL round-bottomed flask, phenol (0.94 g, 10 mmol) was dissolved in 20 mL DCM and Et_3N (2.8 mL, 20 mmol) was added and cooled to 0 ºC. To that Diethylcarbamoyl chloride (1.5 mL, 12 mmol) was added dropwise via syringe and stirred at room temperature overnight. After completion (judged by TLC), quenched with water (30 mL) and extracted with DCM (50 mL x 3). The combined organic phase washed with 5% Sodium bicarbonate (50 mL), water (30 mL x 3), brine (50 mL) successively and dried over anhydrous Na_2SO_4, filtered and concentrated under reduced pressure. The resulting mixture was purified by silica gel chromatography (10% ethyl acetate in hexane as eluent) gave 1.5 g (80%) of phenyl diethylcarbamate **(1h)** as a colourless liquid. Spectral data are in accordance with the reported data.[5]

Synthesis of phenyl methanesulfonate **(1i)***:*

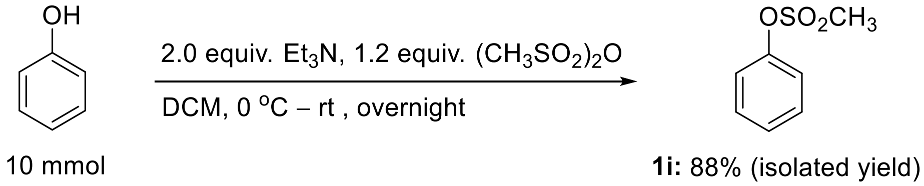

In a dry 100 mL round-bottomed flask, phenol (0.94 g, 10 mmol) was dissolved in 20 mL DCM and Et_3N (2.8 mL, 20 mmol) was added and cooled to 0 ºC. To that Methanesulfonic anhydride (2.1 g, 12 mmol) dissolved in 10 mL DCM was added dropwise via syringe and stirred at room temperature overnight. After completion (judged by TLC), quenched with water (30 mL) and extracted with DCM (50 mL x 3). The combined organic phase washed with 5% Sodium bicarbonate (50 mL), water (30 mL x 3), brine (50 mL) successively and dried over anhydrous Na_2SO_4, filtered and concentrated under reduced pressure. The resulting mixture was purified by silica gel chromatography (10% ethyl acetate in hexane as eluent) gave 1.5 g (88%) of phenyl methanesulfonate **(1i)** as a white solid. Spectral data are in accordance with the reported data.[6]

Synthesis of phenyl 4-methylbenzenesulfonate **(1j)***:*

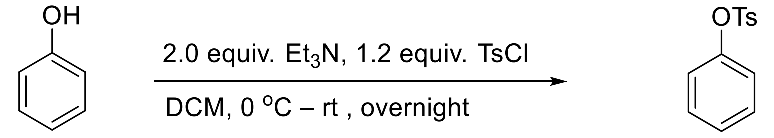

10 mmol

1j: 78% (isolated yield)

In a dry 100 mL round-bottomed flask, phenol (0.94 g, 10 mmol) was dissolved in 20 mL DCM and Et_3N (2.8 mL, 20 mmol) was added and cooled to 0 ºC. To that p-Toluenesulfonyl chloride (2.28 g, 12 mmol) dissolved in 10 mL DCM was added dropwise via syringe and stirred at room temperature overnight. After completion (judged by TLC), quenched with water (30 mL) and extracted with DCM (50 mL x 3). The combined organic phase washed with 5% Sodium bicarbonate (50 mL), water (30 mL x 3), brine (50 mL) successively and dried over anhydrous Na_2SO_4, filtered and concentrated under reduced pressure. The resulting mixture was purified by silica gel chromatography (10% ethyl acetate in hexane as eluent) gave 1.9 g (78%) of phenyl 4-methylbenzenesulfonate **(1j)** as a white solid. Spectral data are in accordance with the reported data.[7]

Synthesis of triisopropyl(phenoxy)silane **(1l)***:*

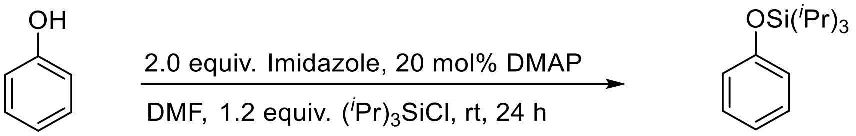

1l: 95% (isolated yield)

In a dry 100 mL round-bottomed flask phenol (0.94 g, 10 mmol), imidazole (1.36 g, 20 mmol, 2.0 equiv.), 4-Dimethylaminopyridine (DMAP) (244 mg, 2 mmol, 20 mol%) and 20 mL dry DMF was added. Then stirred for 5 minutes at room temperature followed by dropwise addition of triisopropylsilyl chloride (TIPSCl) (2.5 ml, 12 mmol, 1.2 equiv.) via syringe. The mixture was stirred at the same temperature for additional 24 h. After completion (judged by TLC), the reaction mixture was diluted with cold water (30 mL) and extracted with ethyl acetate (30 mL x 3). The combined organic layer washed with cold water (30 mL x 3), brine (50 mL) and dried over anhydrous Na_2SO_4, filtered and concentrated under reduced pressure. The resulting mixture was purified by silica gel chromatography (1% ethyl acetate in hexane as eluent) gave 2.3 g (95%) triisopropyl(phenoxy)silane **(1l)** as a colourless liquid.

^{1}H NMR (400 MHz, $CDCl_3$): δ 7.14 – 7.10 (m, 2H), 6.85 – 6.79 (m, 3H), 1.21 – 1.14 (m, 3H), 1.04 – 1.01 (m, 18H).
^{13}C NMR (100 MHz, $CDCl_3$): δ 156.2, 129.5, 121.1, 120.1, 18.1, 12.8.
HRMS (ESI) *m/z* calcd for $C_{15}H_{26}OSi$ $[M+H]^+$ 251.1831, found 251.1834.

Synthesis of triethyl(phenoxy)silane **(1m)***:*

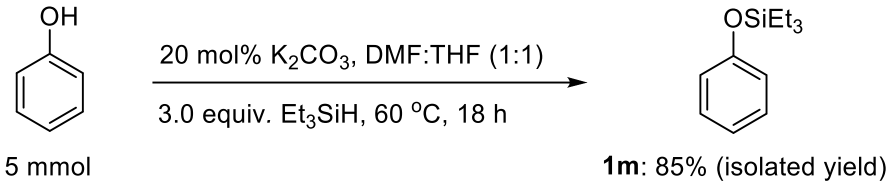

In an argon-filled glovebox, a 35 mL pressure tube was charged with phenol (470 mg, 5 mmol), K_2CO_3 (20 mol%, 120 mg), triethylsilane (2.5 g, 15 mmol, 3.0 equiv.) in DMF:THF (1:1, 10 mL). Then the pressure tube was placed into a preheated silicon oil bath and heated at 60 °C for 24 h. After completion (monitored by TLC), the reaction mixture was cooled to room temperature and filtered through a short pad of celite. Further purification by silica gel column chromatography (10% ethyl acetate in hexane as eluent) gave 884 mg (85%) triethyl(phenoxy)silane **(1m)** as a colourless liquid. Spectral data are in accordance with the reported data.[8]

Synthesis of tert-butyldimethyl(phenoxy)silane **(1n)***:*

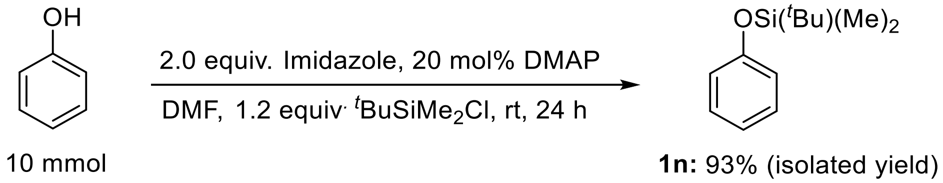

In a dry 100 mL round-bottomed flask phenol (0.94 g, 10 mmol), imidazole (1.36 g, 20 mmol, 2.0 equiv.), 4-Dimethylaminopyridine (DMAP) (244 mg, 2 mmol, 20 mol%) and 20 mL dry DMF was added. Then stirred for 5 minutes at room temperature followed by dropwise addition of tert-butylchlorodimethylsilane (TBDSCl) (2.1 g, 12 mmol, 1.2 equiv.) via syringe. The mixture was stirred at the same temperature for additional 24 h. After completion (judged by TLC), the reaction mixture was diluted with cold water (30 mL) and extracted with ethyl acetate (30 mL x 3). The combined organic layer washed with cold water (30 mL x 3), brine (50 mL) and dried over anhydrous Na_2SO_4, filtered and concentrated under reduced pressure. The resulting mixture was

purified by silica gel chromatography (1% ethyl acetate in hexane as eluent) gave 1.9 g (93%) tert-butyldimethyl(phenoxy)silane **(1n)** as a colourless liquid. Spectral data are in accordance with the reported data.[8]

Synthesis of isopropyldimethyl(phenoxy)silane **(1o)***:*

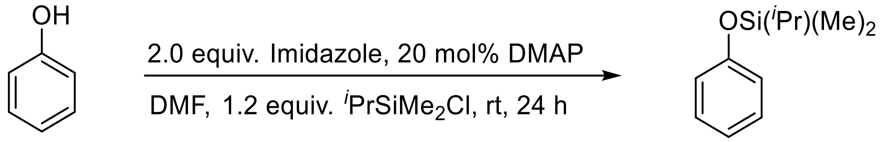

10 mmol

1o: 90% (isolated yield)

In a dry 100 mL round-bottomed flask phenol (0.94 g, 10 mmol), imidazole (1.36 g, 20 mmol, 2.0 equiv.), 4-Dimethylaminopyridine (DMAP) (244 mg, 2 mmol, 20 mol%) and 20 mL dry DMF was added. Then stirred for 5 minutes at room temperature followed by dropwise addition of chloro(isopropyl)dimethylsilane (1.8 ml, 12 mmol, 1.2 equiv.) via syringe. The mixture was stirred at the same temperature for additional 24 h. After completion (judged by TLC), the reaction mixture was diluted with cold water (30 mL) and extracted with ethyl acetate (30 mL x 3). The combined organic layer washed with cold water (30 mL x 3), brine (50 mL) and dried over anhydrous Na_2SO_4, filtered and concentrated under reduced pressure. The resulting mixture was purified by silica gel chromatography (2% ethyl acetate in hexane as eluent) gave 1.7 g (90%) isopropyldimethyl(phenoxy)silane **(1o)** as a colourless liquid.

^{1}H NMR (400 MHz, $CDCl_3$): δ 7.09 – 7.05 (m, 2H), 6.80 (t, *J* = 7.2 Hz, 1H), 6.71 – 6.69 (m, 2H), 0.91 – 0.84 (m, 7H), 0.06 (s, 6H).
^{13}C NMR (100 MHz, $CDCl_3$): δ 155.6, 129.5, 121.5, 120.2, 16.9, 14.8, -3.5.
HRMS (ESI) *m/z* calcd for $C_{11}H_{18}OSi$ $[M+H]^+$ 195.1205, found 195.1208.

Synthesis of (2,3-dimethylbutan-2-yl)dimethyl(phenoxy)silane **(1p)***:*

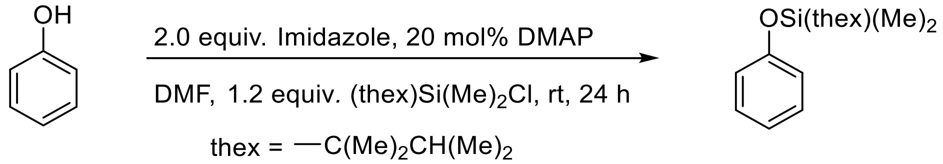

10 mmol

1p: 92% (isolated yield)

In a dry 100 mL round-bottomed flask phenol (0.94 g, 10 mmol), imidazole (1.36 g, 20 mmol, 2.0 equiv.), 4-Dimethylaminopyridine (DMAP) (244 mg, 2 mmol, 20 mol%) and 20 mL dry DMF was added. Then stirred for 5 minutes at room temperature followed by dropwise addition of Dimethylthexylsilyl chloride (TDSCl) (2.1 g, 12 mmol, 1.2 equiv.) via syringe. The mixture was stirred at the same temperature for additional 24 h. After completion (judged by TLC), the reaction mixture was diluted with cold water (30 mL) and extracted with ethyl acetate (30 mL x 3). The combined organic layer washed with cold water (30 mL x 3), brine (50 mL) and dried over anhydrous Na_2SO_4, filtered and concentrated under reduced pressure. The resulting mixture was purified by silica gel chromatography (1% ethyl acetate in hexane as eluent) gave 2.1 g (92%) (2,3-dimethylbutan-2-yl)dimethyl(phenoxy)silane **(1p)** as a colourless liquid. Spectral data are in accordance with the reported data.[9]

Synthesis of tributyl(phenoxy)silane **(1q)***:*

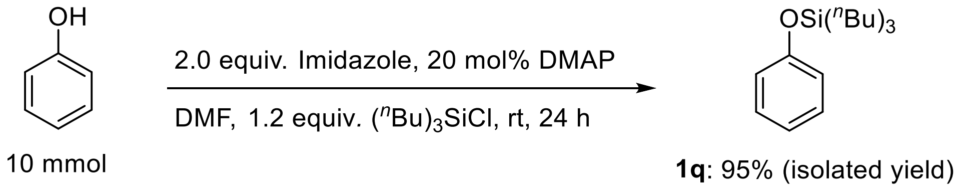

In a dry 100 mL round-bottomed flask phenol (0.94 g, 10 mmol), imidazole (1.36 g, 20 mmol, 2.0 equiv.), 4-Dimethylaminopyridine (DMAP) (244 mg, 2 mmol, 20 mol%) and 20 mL dry DMF was added. Then stirred for 5 minutes at room temperature followed by dropwise addition of tributylchlorosilane (3.2 ml, 12 mmol, 1.2 equiv.) via syringe. The mixture was stirred at the same temperature for additional 24 h. After completion (judged by TLC), the reaction mixture was diluted with cold water (30 mL) and extracted with ethyl acetate (30 mL x 3). The combined organic layer washed with cold water (30 mL x 3), brine (50 mL) and dried over anhydrous Na_2SO_4, filtered and concentrated under reduced pressure. The resulting mixture was purified by silica gel chromatography (1% ethyl acetate in hexane as eluent) gave 2.7 g (95%) tributyl(phenoxy)silane **(1q)** as a colourless liquid. Spectral data are in accordance with the reported data. [10]

Synthesis of benzyltriisopropylsilane **(1r)***:*

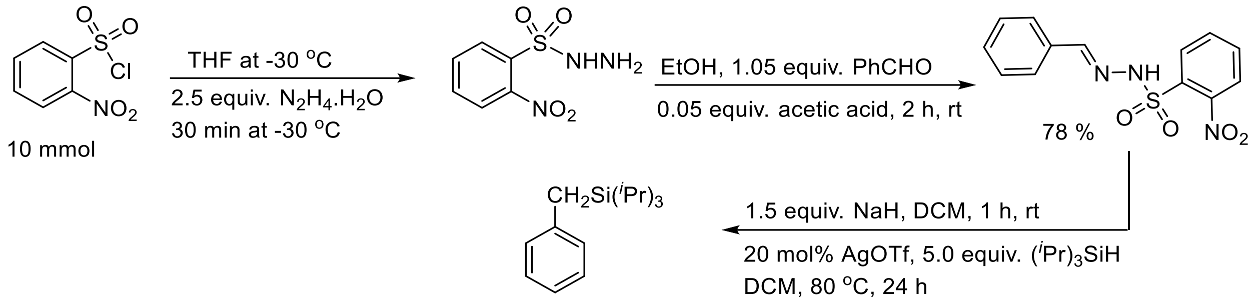

1r: 65% (isolated yield)

Step I: In a dry 250 mL two neck round-bottomed flask, hydrazine monohydrate (2.5 equiv.) was added dropwise to a vigorously stirred solution of o-nitrobenzenesulfonylchloride (2.2 g, 10 mmol) in 50 mL THF at -30 °C and continue stirring for 30 minutes at that temperature. After completion (judged by TLC) the reaction mixture was diluted with cooled EtOAc (100 mL) and organic layers were quickly washed with cooled brine solution (50 mL x 3). The combined organic phases dried over anhydrous Na_2SO_4, filtered and concentrated under reduced pressure. The resulting crude mixture was washed with dry n-pentane (10 mL x 3) and dried in high vaccum pump to get the desired 2-nitrobenzenesulfonohydrazide which was pure enough to use directly for the next step.

Step II: A dry 100 mL two neck round-bottomed flask, was charged with 2-nitrobenzenesulfonohydrazide, and benzaldehyde (1.1 g, 10.5 mmol, 1.05 equiv.), acetic acid (28 mL, 0.5 mmol,0.05 equiv.) and 30 mL of EtOH at room temperature. The resulted heterogeniious mixture was vigorously stirred for 2 h. Then the reaction mixture was concentrated under redued pressure and excess hexanes was added, causing precipitation. After that, the solid product was collected by filtration and washed thoroughly with hexane. Finally, the obtained product dried under vaccum to gave 2.3 g (78%) of (E)-N'-benzylidene-2-nitrobenzenesulfonohydrazide as a yellow solid. Spectral data are in accordance with the reported data.[11]

Step III: In an argon-filled glovebox, a 35 mL pressure tube was charged with (E)-N'-benzylidene-2-nitrobenzenesulfonohydrazide (2.3g, 7.5 mmol), NaH (517 mg, 1.5 equiv. 50 % dispersion in mineral oil) and 15 ml of dry DCM. The reaction mixture stirred for 1 h at room temperature. Then silver trifluoromethanesulfonate (385 mg, 1.5 mmol, 20 mol%), triisopropylsilane (5.9 gm, 37.5 mmol, 5 equiv.) was added and the pressure tube was placed into a preheated silicon oil bath and heated at 80 °C for 24 h. After completion (judged by TLC), the reaction mixture was cooled to room temperature and filtered through a short pad of silica using ethyl acetate as an eluent. The

filtrate was evaporated under reduced pressure to leave a crude mixture which was purified by silica gel chromatography (2% ethyl acetate in hexane as eluent) gave 1.2 g (65%) benzyltriisopropylsilane **(1r)** as a colourless liquid.

^{1}H NMR (400 MHz, $CDCl_3$): δ 7.19 (t, J = 7.2 Hz, 2H), 7.10 (d, J = 7.2 Hz, 2H), 7.05 (t, J = 7.2 Hz, 1H), 2.20 (s, 2H), 1.13 – 1.06 (m, 3H), 1.01 (d, J = 6.0 Hz, 18H).
^{13}C NMR (100 MHz, $CDCl_3$): δ 141.2, 128.7, 128.3, 124.0, 19.1, 18.7, 11.1.
HRMS (ESI) *m/z* calcd for $C_{16}H_{28}Si$ $[M+H]^+$ 249.2039, found 249.2037.

Synthesis of tert-butoxybenzene **(1t)***:*

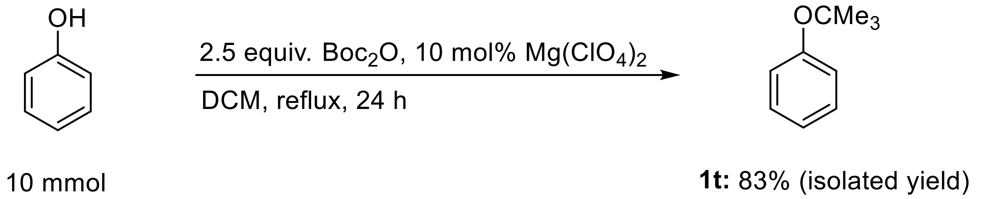

A 35 mL oven dried pressure tube was charged with phenol (0.94 g, 10 mmol), Boc-anhydride (5.4 g, 25 mmol, 2.5 equiv.), $Mg(ClO_4)_2$ (223 mg, 1 mmol, 10 mol%) and 15 mL dry DCM was added. Then the pressure tube was placed into a preheated silicon oil bath and heated at 50 °C for 24 h. After completion (judged by TLC), the reaction mixture was diluted with water (30 mL) and extracted with DCM (30 mL x 3). The combined organic layer washed with brine (50 mL) and dried over anhydrous Na_2SO_4, filtered and concentrated under reduced pressure. The resulting mixture was purified by silica gel chromatography (5% ethyl acetate in hexane as eluent) gave 1.2 g (83%) tert-butoxybenzene **(1t)** as a colourless liquid. Spectral data are in accordance with the reported data.[12]

Synthesis of (2-chlorophenoxy)triisopropylsilane **(4a)***:*

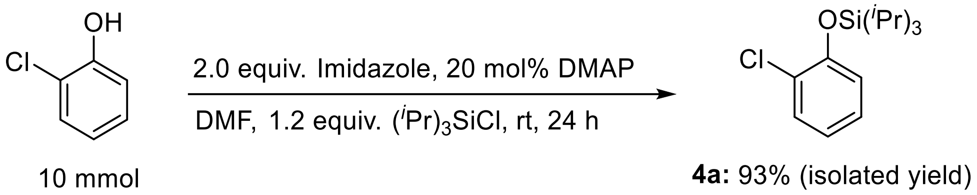

In a dry 100 mL round-bottomed flask 2-chlorophenol (1.28 g, 10 mmol), imidazole (1.36 g, 20 mmol, 2.0 equiv.), 4-Dimethylaminopyridine (DMAP) (244 mg, 2 mmol, 20 mol%) and 20 mL dry DMF was added. Then stirred for 5 minutes at room temperature followed by dropwise

addition of triisopropylsilyl chloride (TIPSCl) (2.5 ml, 12 mmol, 1.2 equiv.) via syringe. The mixture was stirred at the same temperature for additional 24 h. After completion (judged by TLC), the reaction mixture was diluted with cold water (30 mL) and extracted with ethyl acetate (30 mL x 3). The combined organic layer washed with cold water (30 mL x 3), brine (50 mL) and dried over anhydrous Na_2SO_4, filtered and concentrated under reduced pressure. The resulting mixture was purified by silica gel chromatography (5% ethyl acetate in hexane as eluent) gave 2.6 g (93%) (2-chlorophenoxy)triisopropylsilane **(4a)** as a colourless liquid. Spectral data are in accordance with the reported data.[13]

Synthesis of (2-bromophenoxy)triisopropylsilane **(4b)***:*

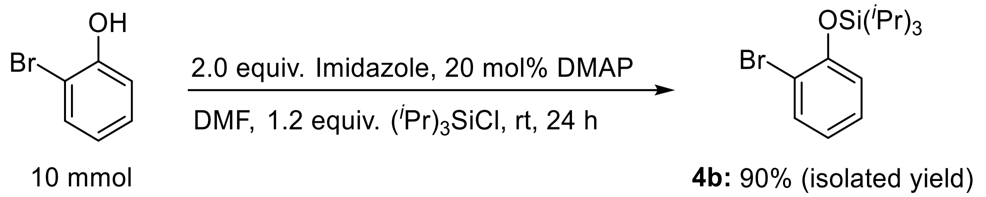

In a dry 100 mL round-bottomed flask 2-bromophenol (1.71 g, 10 mmol), imidazole (1.36 g, 20 mmol, 2.0 equiv.), 4-Dimethylaminopyridine (DMAP) (244 mg, 2 mmol, 20 mol%) and 20 mL dry DMF was added. Then stirred for 5 minutes at room temperature followed by dropwise addition of triisopropylsilyl chloride (TIPSCl) (2.5 ml, 12 mmol, 1.2 equiv.) via syringe. The mixture was stirred at the same temperature for additional 24 h. After completion (judged by TLC), the reaction mixture was diluted with cold water (30 mL) and extracted with ethyl acetate (30 mL x 3). The combined organic layer washed with cold water (30 mL x 3), brine (50 mL) and dried over anhydrous Na_2SO_4, filtered and concentrated under reduced pressure. The resulting mixture was purified by silica gel chromatography (2% ethyl acetate in hexane as eluent) gave 2.9 g (90%) (2-bromophenoxy)triisopropylsilane **(4b)** as a colourless liquid. Spectral data are in accordance with the reported data.[14]

Synthesis of (2-iodophenoxy)triisopropylsilane **(4c)***:*

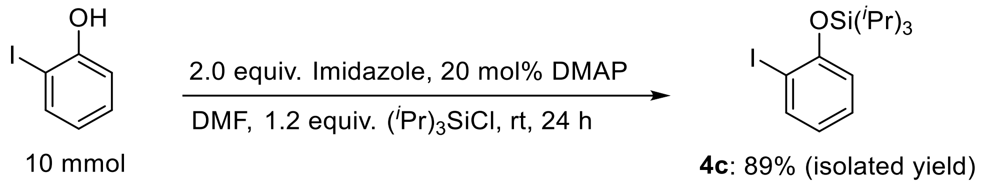

In a dry 100 mL round-bottomed flask 2-iodophenol (2.19 g, 10 mmol), imidazole (1.36 g, 20 mmol, 2.0 equiv.), 4-Dimethylaminopyridine (DMAP) (244 mg, 2 mmol, 20 mol%) and 20 mL dry DMF was added. Then stirred for 5 minutes at room temperature followed by dropwise addition of triisopropylsilyl chloride (TIPSCl) (2.5 ml, 12 mmol, 1.2 equiv.) via syringe. The mixture was stirred at the same temperature for additional 24 h. After completion (judged by TLC), the reaction mixture was diluted with cold water (30 mL) and extracted with ethyl acetate (30 mL x 3). The combined organic layer washed with cold water (30 mL x 3), brine (50 mL) and dried over anhydrous Na_2SO_4, filtered and concentrated under reduced pressure. The resulting mixture was purified by silica gel chromatography (2% ethyl acetate in hexane as eluent) gave 3.3 g (89%) (2-iodophenoxy)triisopropylsilane **(4c)** as a colourless liquid. Spectral data are in accordance with the reported data.[15]

Synthesis of triisopropyl(o-tolyloxy)silane **(4d)***:*

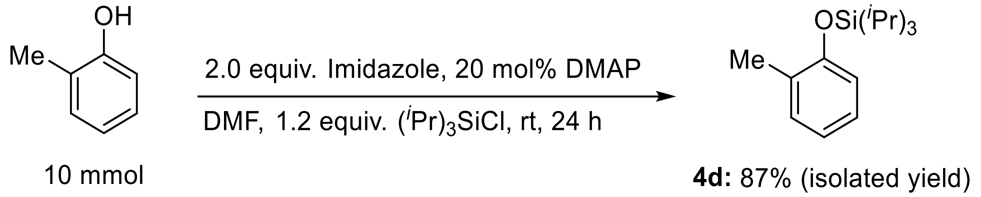

In a dry 100 mL round-bottomed flask o-cresol (1.10 g, 10 mmol), imidazole (1.36 g, 20 mmol, 2.0 equiv.), 4-Dimethylaminopyridine (DMAP) (244 mg, 2 mmol, 20 mol%) and 20 mL dry DMF was added. Then stirred for 5 minutes at room temperature followed by dropwise addition of triisopropylsilyl chloride (TIPSCl) (2.5 ml, 12 mmol, 1.2 equiv.) via syringe. The mixture was stirred at the same temperature for additional 24 h. After completion (judged by TLC), the reaction mixture was diluted with cold water (30 mL) and extracted with ethyl acetate (30 mL x 3). The combined organic layer washed with cold water (30 mL x 3), brine (50 mL) and dried over anhydrous Na_2SO_4, filtered and concentrated under reduced pressure. The resulting mixture was purified by silica gel chromatography (5% ethyl acetate in hexane as eluent) gave 2.3 g (87%)

triisopropyl(o-tolyloxy)silane **(4d)** as a colourless liquid. Spectral data are in accordance with the reported data.[16]

Synthesis of (2-ethylphenoxy)triisopropylsilane **(4e)***:*

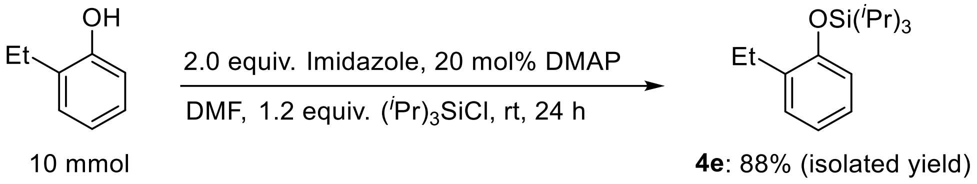

In a dry 100 mL round-bottomed flask 2-ethylphenol (1.22 g, 10 mmol), imidazole (1.36 g, 20 mmol, 2.0 equiv.), 4-Dimethylaminopyridine (DMAP) (244 mg, 2 mmol, 20 mol%) and 20 mL dry DMF was added. Then stirred for 5 minutes at room temperature followed by dropwise addition of triisopropylsilyl chloride (TIPSCl) (2.5 ml, 12 mmol, 1.2 equiv.) via syringe. The mixture was stirred at the same temperature for additional 24 h. After completion (judged by TLC), the reaction mixture was diluted with cold water (30 mL) and extracted with ethyl acetate (30 mL x 3). The combined organic layer washed with cold water (30 mL x 3), brine (50 mL) and dried over anhydrous Na_2SO_4, filtered and concentrated under reduced pressure. The resulting mixture was purified by silica gel chromatography (3% ethyl acetate in hexane as eluent) gave 2.4 g (88%) (2-ethylphenoxy)triisopropylsilane **(4e)** as a colourless liquid.

^{1}H NMR (400 MHz, $CDCl_3$): δ 7.17 (d, *J* = 7.2 Hz, 1H), 7.07 (t, *J* = 7.6 Hz, 1H), 6.89 (t, *J* = 7.2 Hz, 1H), 6.81 (d, *J* = 8.0 Hz, 1H), 2.69 (q, *J* = 7.6 Hz, 2H), 1.39 – 1.31 (m, 3H), 1.23 (t, *J* = 7.6 Hz, 3H), 1.15 (d, *J* = 7.6 Hz, 18H).

^{13}C NMR (100 MHz, $CDCl_3$): δ 153.9, 134.5, 129.3, 126.6, 120.8, 118.0, 23.8, 18.2, 14.4, 13.2.

HRMS (ESI) *m/z* calcd for $C_{17}H_{30}OSi$ $[M+H]^+$ 279.2144, found 279.2140.

Synthesis of triisopropyl(2-pentylphenoxy)silane **(4f)**:

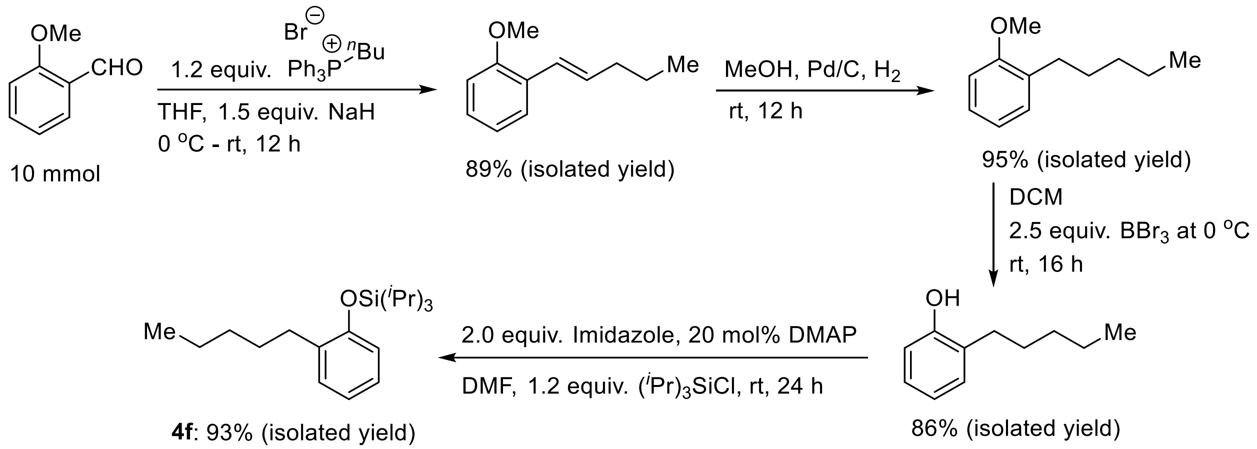

Step I: To a dry 100 mL round-bottomed flask n-butyltriphenylphosphonium bromide (4.79 g, 1.2 equiv.) was taken in dry THF (20 mL) and then NaH (720 mg, 1.5 equiv. 50 % dispersion in mineral oil) was added portion wise at 0 °C and stirred for 1 h at room temperature. After that 2-methoxybenzaldehyde (1.36 g, 10 mmol) dissolved in 10 ml THF was added dropwise at 0 °C followed by overnight stirring at room temperature. After completation the reaction mixture was quenched by H_2O and the organic layer was separated and the aqueous layer was extracted with ethyl acetate(30 mL x 3). The combined organic phase was washed with brine (50 mL), dried over Na_2SO_4, filtered and concentrated by rotary evaporation. The crude mass was purified by silica gel column chromatography (10% ethyl acetate in hexane as eluent) to give (E)-1-methoxy-2-(pent-1-en-1-yl)benzene (1.6 g, 89%) as liquid. Spectral data are in accordance with the reported data.[17]

Step II: An oven dried 100 mL round round bottom flask containing (E)-1-methoxy-2-(pent-1-en-1-yl)benzene (1.6 g, 9.1 mmol) in MeOH (10.0 mL), Pd/C (145 mg, 10 wt. %, 1.5 mol%) was added portion wise. The flask was evacuated and backfilled with H_2 three times. The suspension was stirred with a balloon of H_2 gas for 12 h at room temperature. After completion of reaction (checked by TLC), the reaction mixture was filtered through a pad of celite using ethyl acetate and concentrated under reduced pressure to afford the crude product. The crude product was purified column chromatography using silica gel (10% ethyl acetate in hexane as eluent) to afford 1-methoxy-2-pentylbenzene (1.5 g, 95%) as gummy liquid. Spectral data are in accordance with the reported data.[18]

Step III: In a 100 mL round bottom flask 1-methoxy-2-pentylbenzene (1.5 g, 8.42 mmol) was dissolved in 20 mL of anhydrous DCM. At 0 °C under N_2 atmosphere, a 1 M solution of BBr_3 in

DCM (21 mL, 15.53 mmol) was added drop-wise. The reaction mixture was slowly warmed to room temperature and stirred for 16 h. A saturated aqueous solution of $NaHCO_3$ was added carefully to quench the reaction and diethyl ether were added, and the mixture was stirred for 10-20 min. The organic phase was separated, and the aqueous phase was extracted with diethyl ether (30 mL x 3). The combined organic phase was washed with brine (50 mL), dried over Na_2SO_4, filtered and concentrated under reduced pressure. The crude product was purified by silica gel column chromatography (10% ethyl acetate in hexane as eluent) to afford 2-pentylphenol (1.2 g, 86%) as gummy liquid. Spectral data are in accordance with the reported data.[19]

Step IV: In a dry 100 mL round-bottomed flask 2-pentylphenol (1.2 g, 7.3 mmol), imidazole (0.97 g, 14.6 mmol, 2.0 equiv.), 4-Dimethylaminopyridine (DMAP) (177 mg, 1.46 mmol, 20 mol%) and 20 mL dry DMF was added. Then stirred for 5 minutes at room temperature followed by dropwise addition of triisopropylsilyl chloride (TIPSCl) (1.83 ml, 8.76 mmol, 1.2 equiv.) via syringe. The mixture was stirred at the same temperature for additional 24 h. After completion (judged by TLC), the reaction mixture was diluted with cold water (30 mL) and extracted with ethyl acetate (30 mL x 3). The combined organic layer washed with cold water (30 mL x 3), brine (50 mL) and dried over anhydrous Na_2SO_4, filtered and concentrated under reduced pressure. The resulting mixture was purified by silica gel chromatography (2% ethyl acetate in hexane as eluent) gave 2.1 g (93%) triisopropyl(2-pentylphenoxy)silane **(4f)** as a colourless liquid.

^{1}H NMR (400 MHz, $CDCl_3$): δ 7.13 (d, *J* = 7.2 Hz, 1H), 7.05 (t, *J* = 7.6 Hz, 1H), 6.86 (t, *J* = 7.2 Hz, 1H), 6.79 (d, *J* = 8.0 Hz, 1H), 2.63 (t, *J* = 8.0 Hz, 2H), 1.64 – 1.57 (m, 2H), 1.37 – 1.28 (m, 7H), 1.13 (d, *J* = 7.2 Hz, 18H), 0.91 (t, *J* = 6.8 Hz, 3H).

^{13}C NMR (100 MHz, $CDCl_3$): δ 154.0, 133.2, 130.2, 126.6, 120.6, 118.0, 32.1, 31.0, 30.0, 22.8, 18.3, 14.3, 13.3.

HRMS (ESI) *m/z* calcd for $C_{20}H_{36}OSi$ $[M+H]^+$ 321.2614, found 321.2615.

Synthesis of triisopropyl(2-(trifluoromethyl)phenoxy)silane **(4g)***:*

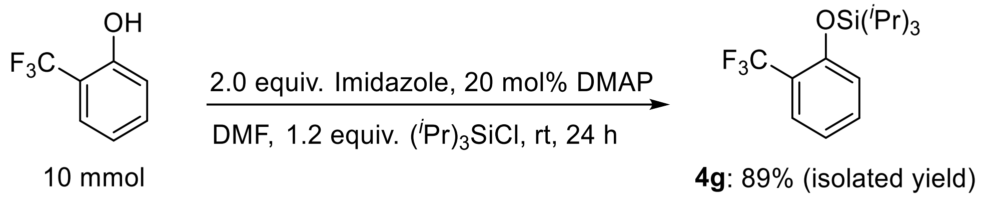

In a dry 100 mL round-bottomed flask 2-(trifluoromethyl)phenol (1.62 g, 10 mmol), imidazole (1.36 g, 20 mmol, 2.0 equiv.), 4-Dimethylaminopyridine (DMAP) (244 mg, 2 mmol, 20 mol%) and 20 mL dry DMF was added. Then stirred for 5 minutes at room temperature followed by dropwise addition of triisopropylsilyl chloride (TIPSCl) (2.5 ml, 12 mmol, 1.2 equiv.) via syringe. The mixture was stirred at the same temperature for additional 24 h. After completion (judged by TLC), the reaction mixture was diluted with cold water (30 mL) and extracted with ethyl acetate (30 mL x 3). The combined organic layer washed with cold water (30 mL x 3), brine (50 mL) and dried over anhydrous Na_2SO_4, filtered and concentrated under reduced pressure. The resulting mixture was purified by silica gel chromatography (3% ethyl acetate in hexane as eluent) gave 2.8 g (89%) triisopropyl(2-(trifluoromethyl)phenoxy)silane **(4g)** as a colourless liquid.

^{1}H NMR (400 MHz, $CDCl_3$) δ 7.61 (d, *J* = 5.6 Hz, 1H), 7.43 (t, *J* = 7.6 Hz, 1H), 7.01 – 6.99 (m, 2H), 1.45 – 1.40 (m, 3H), 1.24 – 1.21 (m, 18H).
^{13}C NMR (100 MHz, $CDCl_3$): δ 154.7, 133.1, 127.3 (d, *J* = 5.1 Hz), 124.2 (d, *J* = 270.8 Hz), 120.9 (d, *J* = 29.3 Hz), 120.2, 119.5, 18.0, 13.3.
HRMS (ESI) *m/z* calcd for $C_{16}H_{25}F_3OSi$ $[M+H]^+$ 319.1705, found 319.1707.

Synthesis of triisopropyl(2-isopropylphenoxy)silane **(4h)***:*

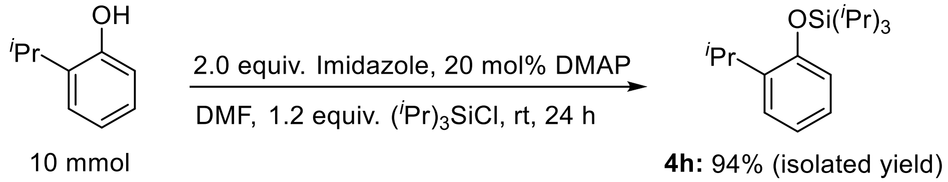

In a dry 100 mL round-bottomed flask 2-isopropylphenol (1.36 g, 10 mmol), imidazole (1.36 g, 20 mmol, 2.0 equiv.), 4-Dimethylaminopyridine (DMAP) (244 mg, 2 mmol, 20 mol%) and 20 mL dry DMF was added. Then stirred for 5 minutes at room temperature followed by dropwise addition of triisopropylsilyl chloride (TIPSCl) (2.5 ml, 12 mmol, 1.2 equiv.) via syringe. The mixture was stirred at the same temperature for additional 24 h. After completion (judged by TLC),

the reaction mixture was diluted with cold water (30 mL) and extracted with ethyl acetate (30 mL x 3). The combined organic layer washed with cold water (30 mL x 3), brine (50 mL) and dried over anhydrous Na_2SO_4, filtered and concentrated under reduced pressure. The resulting mixture was purified by silica gel chromatography (1% ethyl acetate in hexane as eluent) gave 2.7 g (94%) triisopropyl(2-isopropylphenoxy)silane **(4h)** as a colourless liquid.

^{1}H NMR (400 MHz, $CDCl_3$): δ 7.24 (d, J = 7.6 Hz, 1H), 7.08 – 7.04 (m, 1H), 6.94 (t, J = 7.6 Hz, 1H), 6.82 (d, J = 8.0 Hz, 1H), 3.46 – 3.40 (m, 1H), 1.41 – 1.31 (m, 3H), 1.25 (d, J = 7.2 Hz, 6H), 1.16 (d, J = 7.6 Hz, 18H).
^{13}C NMR (100 MHz, $CDCl_3$): δ 153.3, 138.7, 126.3, 126.2, 120.9, 118.0, 26.8, 23.0, 18.3, 13.3.
HRMS (ESI) *m/z* calcd for $C_{18}H_{32}OSi$ $[M+H]^+$ 293.2301, found 293.2298.

Synthesis of triisopropyl(2-(trifluoromethoxy)phenoxy)silane **(4i)***:*

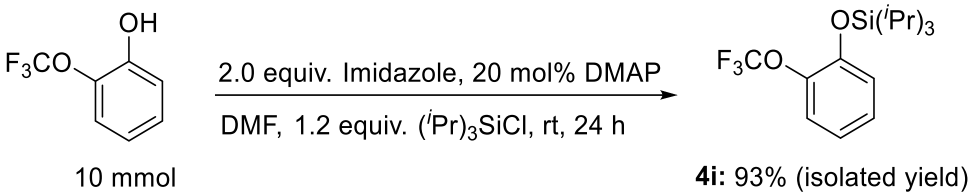

In a dry 100 mL round-bottomed flask 2-(trifluoromethoxy)phenol (1.78 g, 10 mmol), imidazole (1.36 g, 20 mmol, 2.0 equiv.), 4-Dimethylaminopyridine (DMAP) (244 mg, 2 mmol, 20 mol%) and 20 mL dry DMF was added. Then stirred for 5 minutes at room temperature followed by dropwise addition of triisopropylsilyl chloride (TIPSCl) (2.5 ml, 12 mmol, 1.2 equiv.) via syringe. The mixture was stirred at the same temperature for additional 24 h. After completion (judged by TLC), the reaction mixture was diluted with cold water (30 mL) and extracted with ethyl acetate (30 mL x 3). The combined organic layer washed with cold water (30 mL x 3), brine (50 mL) and dried over anhydrous Na_2SO_4, filtered and concentrated under reduced pressure. The resulting mixture was purified by silica gel chromatography (1% ethyl acetate in hexane as eluent) gave 3.1 g (93%) triisopropyl(2-(trifluoromethyl)phenoxy)silane **(4i)** as a colourless liquid. Spectral data are in accordance with the reported data.[13]

Synthesis of 2-((triisopropylsilyl)oxy)aniline **(4j)***:*

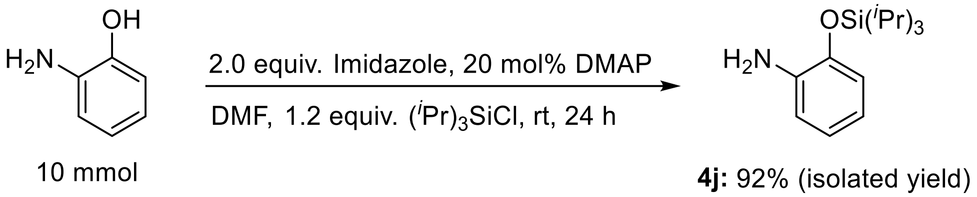

In a dry 100 mL round-bottomed flask 2-aminophenol (1.09 g, 10 mmol), imidazole (1.36 g, 20 mmol, 2.0 equiv.), 4-Dimethylaminopyridine (DMAP) (244 mg, 2 mmol, 20 mol%) and 20 mL dry DMF was added. Then stirred for 5 minutes at room temperature followed by dropwise addition of triisopropylsilyl chloride (TIPSCl) (2.5 ml, 12 mmol, 1.2 equiv.) via syringe. The mixture was stirred at the same temperature for additional 24 h. After completion (judged by TLC), the reaction mixture was diluted with cold water (30 mL) and extracted with ethyl acetate (30 mL x 3). The combined organic layer washed with cold water (30 mL x 3), brine (50 mL) and dried over anhydrous Na_2SO_4, filtered and concentrated under reduced pressure. The resulting mixture was purified by silica gel chromatography (1% ethyl acetate in hexane as eluent) gave 2.4 g (92%) 2-((triisopropylsilyl)oxy)aniline **(4j)** as a colourless liquid. Spectral data are in accordance with the reported data.[20]

Synthesis of triisopropyl(2-(methylthio)phenoxy)silane **(4k)***:*

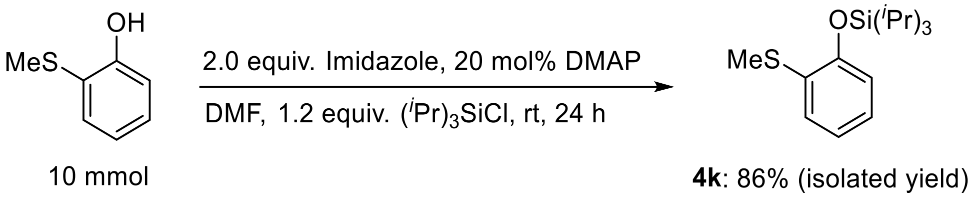

In a dry 100 mL round-bottomed flask 2-(methylthio)phenol (1.4 g, 10 mmol), imidazole (1.36 g, 20 mmol, 2.0 equiv.), 4-Dimethylaminopyridine (DMAP) (244 mg, 2 mmol, 20 mol%) and 20 mL dry DMF was added. Then stirred for 5 minutes at room temperature followed by dropwise addition of triisopropylsilyl chloride (TIPSCl) (2.5 ml, 12 mmol, 1.2 equiv.) via syringe. The mixture was stirred at the same temperature for additional 24 h. After completion (judged by TLC), the reaction mixture was diluted with cold water (30 mL) and extracted with ethyl acetate (30 mL x 3). The combined organic layer washed with cold water (30 mL x 3), brine (50 mL) and dried over anhydrous Na_2SO_4, filtered and concentrated under reduced pressure. The resulting mixture was purified by silica gel chromatography (2% ethyl acetate in hexane as eluent) gave 2.5 g (86%) triisopropyl(2-(methylthio)phenoxy)silane **(4k)** as a colourless liquid.

^{1}H NMR (400 MHz, $CDCl_3$): δ 7.12 (dd, *J* = 7.6, 1.2 Hz, 1H), 7.05 (td, *J* = 7.6, 1.6 Hz, 1H), 7.00 – 6.96 (m, 1H), 6.85 (d, *J* = 7.2 Hz, 1H), 2.43 (s, 3H), 1.44 – 1.35 (m, 3H), 1.21 (d, *J* = 7.6 Hz, 18H).

^{13}C NMR (101 MHz, $CDCl_3$): δ 152.5, 130.1, 125.0, 121.5, 117.6, 18.1, 14.2, 13.1.

HRMS (ESI) *m/z* calcd for $C_{16}H_{28}OSSi$ $[M+H]^+$ 297.1708, found 297.1711.

Synthesis of 2-((triisopropylsilyl)oxy)benzonitrile **(4l)***:*

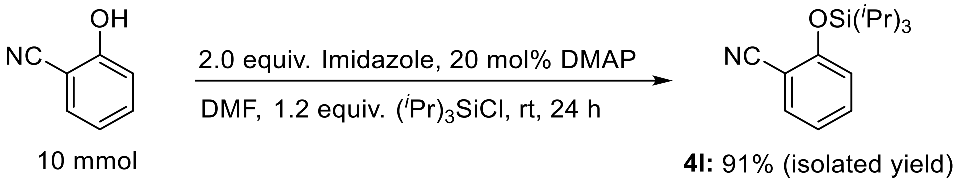

In a dry 100 mL round-bottomed flask 2-hydroxybenzonitrile (1.19 g, 10 mmol), imidazole (1.36 g, 20 mmol, 2.0 equiv.), 4-Dimethylaminopyridine (DMAP) (244 mg, 2 mmol, 20 mol%) and 20 mL dry DMF was added. Then stirred for 5 minutes at room temperature followed by dropwise addition of triisopropylsilyl chloride (TIPSCl) (2.5 ml, 12 mmol, 1.2 equiv.) via syringe. The mixture was stirred at the same temperature for additional 24 h. After completion (judged by TLC), the reaction mixture was diluted with cold water (30 mL) and extracted with ethyl acetate (30 mL x 3). The combined organic layer washed with cold water (30 mL x 3), brine (50 mL) and dried over anhydrous Na_2SO_4, filtered and concentrated under reduced pressure. The resulting mixture was purified by silica gel chromatography (1% ethyl acetate in hexane as eluent) gave 2.5 g (91%) 2-((triisopropylsilyl)oxy)benzonitrile **(4l)** as a colourless liquid. Spectral data are in accordance with the reported data.[21]

Synthesis of triisopropyl(2-(4,4,5,5-tetramethyl-1,3,2-dioxaborolan-2-yl)phenoxy)silane **(4m)***:*

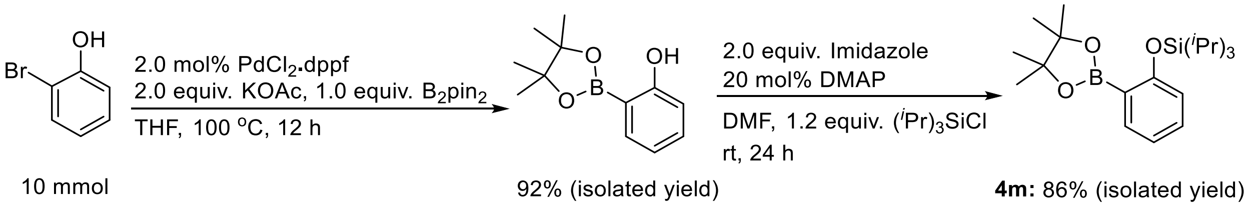

Step I: In an argon-filled glovebox, a 35 mL pressure tube was charged with $PdCl_2$•dppf (146.3 mg, 2.0 mol%), KOAc (1.96 g, 2.0 equiv.), B_2pin_2 (2.54 g, 1.0 equiv.), 2-bromophenol (1.72 g, 10 mmol) and dry THF (15.0 mL). The pressure tube was placed into a preheated silicon oil bath and heated at 100 °C for 12 h. After completion (monitored by GC/MS), the reaction mixture was

cooled to room temperature and filtered through a short pad of celite and evaporated under reduced pressure to afford the crude product. Chromatographic separation was performed with silica gel (10% ethyl acetate in hexane as eluent) gave 2.0 g (92%) 2-(4,4,5,5-tetramethyl-1,3,2-dioxaborolan-2-yl)phenol as gummy liquid. Spectral data are in accordance with the reported data.[22]

Step II: In a dry 100 mL round-bottomed flask 2-(4,4,5,5-tetramethyl-1,3,2-dioxaborolan-2-yl)phenol (2 g, 9.0 mmol), imidazole (1.22 g, 18 mmol, 2.0 equiv.), 4-Dimethylaminopyridine (DMAP) (219 mg, 1.8 mmol, 20 mol%) and 20 mL dry DMF was added. Then stirred for 5 minutes at room temperature followed by dropwise addition of triisopropylsilyl chloride (TIPSCl) (2.25 ml, 10.8 mmol, 1.2 equiv.) via syringe. The mixture was stirred at the same temperature for additional 24 h. After completion (judged by TLC), the reaction mixture was diluted with cold water (30 mL) and extracted with ethyl acetate (30 mL x 3). The combined organic layer washed with cold water (30 mL x 3), brine (50 mL) and dried over anhydrous Na_2SO_4, filtered and concentrated under reduced pressure. The resulting mixture was purified by silica gel chromatography (2% ethyl acetate in hexane as eluent) gave 2.9 g (86%) triisopropyl(2-(4,4,5,5-tetramethyl-1,3,2-dioxaborolan-2-yl)phenoxy)silane **(4m)** as a colourless liquid.

^{1}H NMR (400 MHz, $CDCl_3$): δ 7.60 (dd, *J* = 7.2, 1.2 Hz, 1H), 7.18 (dt, *J* = 8.0, 1.6 Hz, 1H), 6.81 (t, *J* = 7.2 Hz, 1H), 6.71 (d, *J* = 8.0 Hz, 1H), 1.27 – 1.21 (m, 15H), 1.04 (d, *J* = 7.2 Hz, 18H).
^{13}C NMR (100 MHz, $CDCl_3$): δ 161.3, 137.2, 132.3, 120.4, 119.0, 83.2, 25.0, 18.2, 13.4.
^{11}B NMR (128 MHz, $CDCl_3$): δ 30.7.
HRMS (ESI) *m/z* calcd for $C_{21}H_{37}BO_3Si$ $[M+H]^+$ 377.2683, found 377.2685.

Synthesis of 1-(2-((triisopropylsilyl)oxy)phenyl)pyrrolidine **(4n)***:*

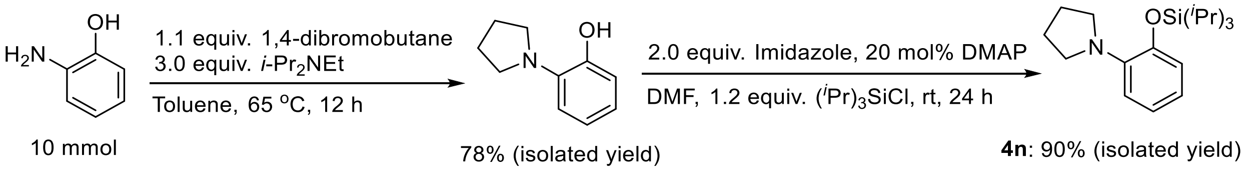

Step I: A dry 100 mL round-bottomed flask was charged with 2-aminophenol (1.09 g, 10 mmol), 1,4-dibromobutane (1.3 mL, 11 mmol, 1.1 equiv.), and *i*-Pr_2NEt (5.2 mL, 30 mmol, 3 equiv.) sequentially. Then dry toluene was added (20 mL) and stirred for 12 h at 65 ^oC. After completation the reaction mixture (judged by TLC), 2N aqueous HCl (50 ml) solution was added. and the

reaction mixture was extracted with ethyl acetate (30 mL x 3). The combined organic phase was washed with brine (50 mL), dried over Na_2SO_4, filtered and concentrated under reduced pressure. The crude mass was purified by silica gel column chromatography (30% ethyl acetate in hexane as eluent) to give 2-(pyrrolidin-1-yl)phenol (1.2 g, 78%) as a light brown solid. Spectral data are in accordance with the reported data.

Step II: In a dry 100 mL round-bottomed flask 2-(pyrrolidin-1-yl)phenol (1.2 g, 7.3 mmol), imidazole (0.97 g, 14.6 mmol, 2.0 equiv.), 4-Dimethylaminopyridine (DMAP) (177 mg, 1.46 mmol, 20 mol%) and 20 mL dry DMF was added. Then stirred for 5 minutes at room temperature followed by dropwise addition of triisopropylsilyl chloride (TIPSCl) (1.83 ml, 8.76 mmol, 1.2 equiv.) via syringe. The mixture was stirred at the same temperature for additional 24 h. After completion (judged by TLC), the reaction mixture was diluted with cold water (30 mL) and extracted with ethyl acetate (30 mL x 3). The combined organic layer washed with cold water (30 mL x 3), brine (50 mL) and dried over anhydrous Na_2SO_4, filtered and concentrated under reduced pressure. The resulting mixture was purified by silica gel chromatography (3% ethyl acetate in hexane as eluent) gave 2.1 g (90%) 1-(2-((triisopropylsilyl)oxy)phenyl)pyrrolidine **(4n)** as a colourless liquid.

^{1}H NMR (400 MHz, $CDCl_3$): δ 6.86 (t, J = 7.2 Hz, 1H), 6.78 (dd, J = 13.2, 7.6 Hz, 2H), 6.70 (t, J = 7.6 Hz, 1H), 3.28 (t, J = 6.4 Hz, 4H), 1.93 – 1.86 (m, 4H), 1.34 – 1.28 (m, 3H), 1.12 (d, J = 7.2 Hz, 18H).
^{13}C NMR (100 MHz, $CDCl_3$): δ 146.9, 141.8, 121.4, 119.4, 119.3, 116.1, 50.3, 24.8, 18.1, 13.4.
HRMS (ESI) *m/z* calcd for $C_{19}H_{33}NOSi$ $[M+H]^+$ 320.2410, found 320.2411.

Synthesis of (2-cyclohexylphenoxy)triisopropylsilane **(4o)***:*

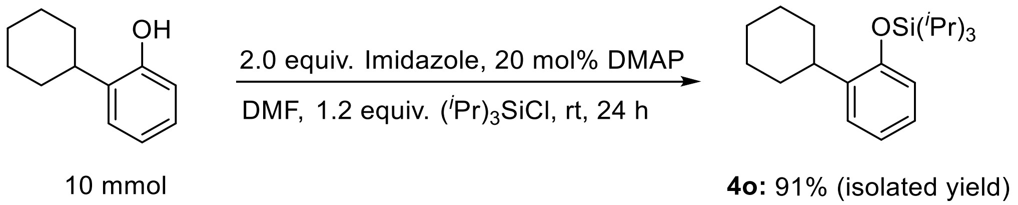

In a dry 100 mL round-bottomed flask 2-cyclohexylphenol (1.76 g, 10 mmol), imidazole (1.36 g, 20 mmol, 2.0 equiv.), 4-Dimethylaminopyridine (DMAP) (244 mg, 2 mmol, 20 mol%) and 20 mL dry DMF was added. Then stirred for 5 minutes at room temperature followed by dropwise addition of triisopropylsilyl chloride (TIPSCl) (2.5 ml, 12 mmol, 1.2 equiv.) via syringe. The

mixture was stirred at the same temperature for additional 24 h. After completion (judged by TLC), the reaction mixture was diluted with cold water (30 mL) and extracted with ethyl acetate (30 mL x 3). The combined organic layer washed with cold water (30 mL x 3), brine (50 mL) and dried over anhydrous Na_2SO_4, filtered and concentrated under reduced pressure. The resulting mixture was purified by silica gel chromatography (1% ethyl acetate in hexane as eluent) gave 3.0 g (91%) (2-cyclohexylphenoxy)triisopropylsilane **(4o)** as a colourless liquid.

^{1}H NMR (400 MHz, $CDCl_3$): δ 7.19 (dd, *J* = 7.6, 1.2 Hz, 1H), 7.07 – 7.02 (m, 1H), 6.93 – 6.90 (m, 1H), 6.81 – 6.79 (m, 1H), 3.05 (t, *J* = 8.8 Hz, 1H), 1.91 – 1.83 (m, 4H), 1.78 (d, *J* = 12.8 Hz, 1H), 1.45 – 1.30 (m, 8H), 1.15 (d, *J* = 7.6 Hz, 18H).
^{13}C NMR (100 MHz, $CDCl_3$): δ 153.3, 137.9, 126.8, 126.2, 120.9, 118.0, 37.1, 33.4, 27.3, 26.6, 18.3, 13.3.
HRMS (ESI) *m/z* calcd for $C_{21}H_{36}OSi$ $[M+H]^+$ 333.2614, found 333.2616.

Synthesis of 1-(2-((triisopropylsilyl)oxy)phenyl)ethan-1-one **(4p)***:*

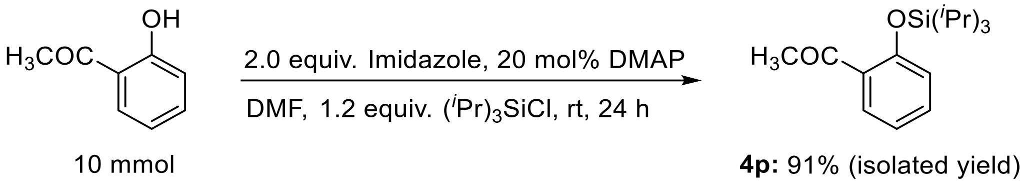

In a dry 100 mL round-bottomed flask 1-(2-hydroxyphenyl)ethan-1-one (1.36 g, 10 mmol), imidazole (1.36 g, 20 mmol, 2.0 equiv.), 4-Dimethylaminopyridine (DMAP) (244 mg, 2 mmol, 20 mol%) and 20 mL dry DMF was added. Then stirred for 5 minutes at room temperature followed by dropwise addition of triisopropylsilyl chloride (TIPSCl) (2.5 ml, 12 mmol, 1.2 equiv.) via syringe. The mixture was stirred at the same temperature for additional 24 h. After completion (judged by TLC), the reaction mixture was diluted with cold water (30 mL) and extracted with ethyl acetate (30 mL x 3). The combined organic layer washed with cold water (30 mL x 3), brine (50 mL) and dried over anhydrous Na_2SO_4, filtered and concentrated under reduced pressure. The resulting mixture was purified by silica gel chromatography (2% ethyl acetate in hexane as eluent) gave 2.6 g (91%) 1-(2-((triisopropylsilyl)oxy)phenyl)ethan-1-one **(4p)** as a colourless liquid. Spectral data are in accordance with the reported data.[23]

Synthesis of methyl ethyl 2-(2-((triisopropylsilyl)oxy)phenyl)acetate **(4q)***:*

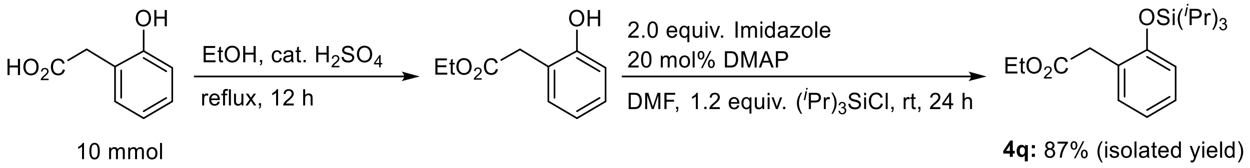

Step I: A 35 mL pressure tube was charged with a mixture of 2-(2-hydroxyphenyl)acetic acid (1.52 g, 10 mmol), EtOH (15 mL) and catalytic amount of H_2SO_4. Then it was refluxed for 12 h with vigorous stirring. After that, the reaction mixture was cooled to room temperature and neutralize with aqueous Na_2CO_3. The whole mixture was then extracted with dichloromethane (30 mL x 3) and washed with water. The combined organic layer dried over anhydrous Na_2SO_4, filtered and concentrated under reduced pressure to afford the ethyl 2-(2-hydroxyphenyl)acetate quantitatively which was directly used for the next step without further purifications.

Step II: In a dry 100 mL round-bottomed flask crude ethyl 2-(2-hydroxyphenyl)acetate, imidazole (1.36 g, 20 mmol, 2.0 equiv.), 4-Dimethylaminopyridine (DMAP) (244 mg, 2 mmol, 20 mol%) and 20 mL dry DMF was added. Then stirred for 5 minutes at room temperature followed by dropwise addition of triisopropylsilyl chloride (TIPSCl) (2.5 ml, 12 mmol, 1.2 equiv.) via syringe. The mixture was stirred at the same temperature for additional 24 h. After completion (judged by TLC), the reaction mixture was diluted with cold water (30 mL) and extracted with ethyl acetate (30 mL x 3). The combined organic layer washed with cold water (30 mL x 3), brine (50 mL) and dried over anhydrous Na_2SO_4, filtered and concentrated under reduced pressure. The resulting mixture was purified by silica gel chromatography (1% ethyl acetate in hexane as eluent) gave 2.9 g (87%) ethyl 2-(2-((triisopropylsilyl)oxy)phenyl)acetate **(4q)** as a colourless liquid.

^{1}H NMR (400 MHz, $CDCl_3$): δ 7.19 (d, J = 7.6 Hz, 1H), 7.14 – 7.10 (m, 1H), 6.89 (t, J = 7.6 Hz, 1H), 6.82 (d, J = 8.0 Hz, 1H), 4.13 (q, J = 6.8 Hz, 2H), 3.65 (s, 2H), 1.36 – 1.30 (m, 3H), 1.23 (t, J = 7.2 Hz, 3H), 1.11 (d, J = 7.2 Hz, 18H).

^{13}C NMR (100 MHz, $CDCl_3$): δ 171.9, 154.2, 131.2, 128.2, 124.9, 120.8, 117.9, 60.7, 36.3, 31.7, 18.1, 13.2.

HRMS (ESI) *m/z* calcd for $C_{19}H_{32}O_3Si$ $[M+H]^+$ 337.2199, found 337.2202.

Synthesis of N,N-diethyl-2-((triisopropylsilyl)oxy)benzamide **(4r)**:

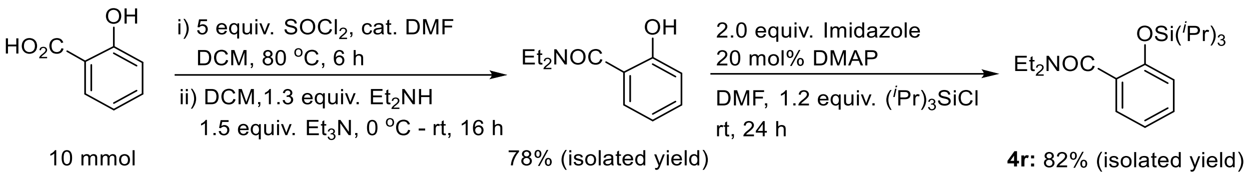

Step I: A 100 mL round-bottom flask was charged with 2-hydroxybenzoic acid (1.38 g, 10 mmol), $SOCl_2$ (3.6 mL, 50 mmol, 5 equiv.), dry DCM (20 mL) and catalytic amount of DMF. After that reaction mixture was heated at 80 ^{o}C temperature for 6 h. The resulting mixture was concentrated under reduced pressure to afford acid chloride quantitatively which was used directly for the next step without further purification.

To a solution of diethylamine (1.34 mL, 13 mmol, 1.3 equiv.) and Et_3N (2.0 mL, 15 mmol, 1.5 equiv.) in dry DCM (20 mL), acid chloride (1.0 equiv.) was added dropwise via syringe at 0 °C and the reaction mixture was stirred at room temperature for 12 h. Then water (50 mL) was added, the organic layer was separated and the aqueous layer was extracted with DCM (3 x 30 mL). The combined organic layer was washed with saturated aqueous $NaHCO_3$ (30 mL x 2) solution followed by brine solution (50 mL). After that, the organic layer was dried over Na_2SO_4, filtered and concentrated under reduced pressure. The crude mass was purified by silica gel column chromatography (60% ethyl acetate in hexane as eluent) to give N,N-diethyl-2-hydroxybenzamide (1.5 g, 78%) as oil. Spectral data are in accordance with the reported data.[24]

Step II: In a dry 100 mL round-bottomed flask N,N-diethyl-2-hydroxybenzamide (1.5 g, 7.7 mmol), imidazole (1.04 g, 15.4 mmol, 2.0 equiv.), 4-Dimethylaminopyridine (DMAP) (188 mg, 1.5 mmol, 20 mol%) and 20 mL dry DMF was added. Then stirred for 5 minutes at room temperature followed by dropwise addition of triisopropylsilyl chloride (TIPSCl) (1.9 ml, 9.24 mmol, 1.2 equiv.) via syringe. The mixture was stirred at the same temperature for additional 24 h. After completion (judged by TLC), the reaction mixture was diluted with cold water (30 mL) and extracted with ethyl acetate (30 mL x 3). The combined organic layer washed with cold water (30 mL x 3), brine (50 mL) and dried over anhydrous Na_2SO_4, filtered and concentrated under reduced pressure. The resulting mixture was purified by silica gel chromatography (15% ethyl acetate in hexane as eluent) gave 2.2 g (82%) N,N-diethyl-2-((triisopropylsilyl)oxy)benzamide **(4r)** as a colourless liquid.

^{1}H NMR (400 MHz, $CDCl_3$): δ 7.21 – 7.15 (m, 2H), 6.92 (t, J = 7.6 Hz, 1H), 6.82 (d, J = 8.0 Hz, 1H), 3.61 – 3.45 (m, 2H), 3.26 – 3.19 (m, 1H), 3.16 – 3.07 (m, 1H), 1.32 – 1.21 (m, 6H), 1.10 – 1.06 (m, 18H), 1.01 (t, J = 7.2 Hz, 3H).

^{13}C NMR (100 MHz, $CDCl_3$): δ 169.1, 151.6, 129.6, 129.4, 127.9, 121.0, 118.8, 43.0, 39.3, 18.1, 14.2, 13.3, 13.0.

HRMS (ESI) *m/z* calcd for $C_{20}H_{35}NO_2Si$ $[M+H]^+$ 350.2515, found 350.2512.

Synthesis of morpholino(2-((triisopropylsilyl)oxy)phenyl)methanone **(4s)***:*

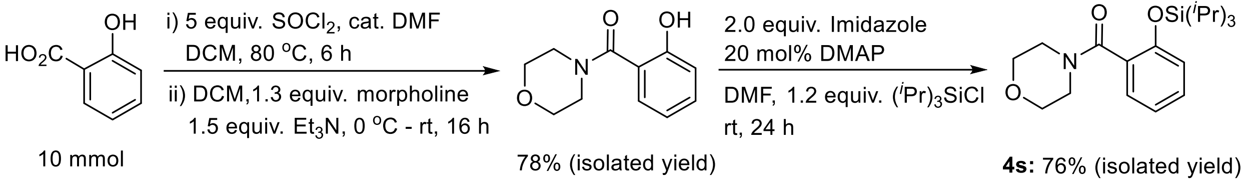

Step I: A 100 mL round-bottom flask was charged with 2-hydroxybenzoic acid (1.38 g, 10 mmol), $SOCl_2$ (3.6 mL, 50 mmol, 5 equiv.), dry DCM (20 mL) and catalytic amount of DMF. After that reaction mixture was heated at 80 °C temperature for 6 h. The resulting mixture was concentrated under reduced pressure to afford acid chloride quantitatively which was used directly for the next step without further purification.

To a solution of morpholine (1.12 mL, 13 mmol, 1.3 equiv.) and Et_3N (2.0 mL, 15 mmol, 1.5 equiv.) in dry DCM (20 mL), acid chloride (1.0 equiv.) was added dropwise via syringe at 0 °C and the reaction mixture was stirred at room temperature for 12 h. Then water (50 mL) was added, the organic layer was separated and the aqueous layer was extracted with DCM (3 x 30 mL). The combined organic layer was washed with saturated aqueous $NaHCO_3$ (30 mL x 2) solution followed by brine solution (50 mL). After that, the organic layer was dried over Na_2SO_4, filtered and concentrated under reduced pressure. The crude mass was purified by silica gel column chromatography (70% ethyl acetate in hexane as eluent) to give (2-hydroxyphenyl)(morpholino)methanone (1.6 g , 78%) as oil. Spectral data are in accordance with the reported data. [25]

Step II: In a dry 100 mL round-bottomed flask (2-hydroxyphenyl)(morpholino)methanone (1.6 g, 7.7 mmol), imidazole (1.04 g, 15.4 mmol, 2.0 equiv.), 4-Dimethylaminopyridine (DMAP) (188 mg, 1.5 mmol, 20 mol%) and 20 mL dry DMF was added. Then stirred for 5 minutes at room temperature followed by dropwise addition of triisopropylsilyl chloride (TIPSCl) (1.9 ml, 9.24

mmol, 1.2 equiv.) via syringe. The mixture was stirred at the same temperature for additional 24 h. After completion (judged by TLC), the reaction mixture was diluted with cold water (30 mL) and extracted with ethyl acetate (30 mL x 3). The combined organic layer washed with cold water (30 mL x 3), brine (50 mL) and dried over anhydrous Na_2SO_4, filtered and concentrated under reduced pressure. The resulting mixture was purified by silica gel chromatography (10% ethyl acetate in hexane as eluent) gave 2.1 g (76%) morpholino(2-((triisopropylsilyl)oxy)phenyl)methanone **(4s)** as a colourless liquid.

^{1}H NMR (400 MHz, $CDCl_3$): δ 7.23 – 7.20 (m, 2H), 6.94 (t, *J* = 7.6 Hz, 1H), 6.82 (d, *J* = 8.4 Hz, 1H), 4.10 – 4.05 (m, 1H), 3.81 – 3.77 (m, 1H), 3.68 – 3.62 (m, 2H), 3.48 – 3.42 (m, 2H), 3.3 – 3.22 (m, 2H), 1.29 – 1.23 (m, 3H), 1.03 (d, *J* = 7.2 Hz, 18H).
^{13}C NMR (100 MHz, $CDCl_3$): δ 168.4, 151.8, 130.3, 128.3, 127.7, 121.3, 118.8, 67.0, 66.8, 47.1, 41.9, 17.8, 12.9.
HRMS (ESI) *m/z* calcd for $C_{20}H_{33}NO_3Si$ $[M+H]^+$ 364.2308, found 364.2314.

Synthesis of triisopropyl(3-(trifluoromethyl)phenoxy)silane **(4t)***:*

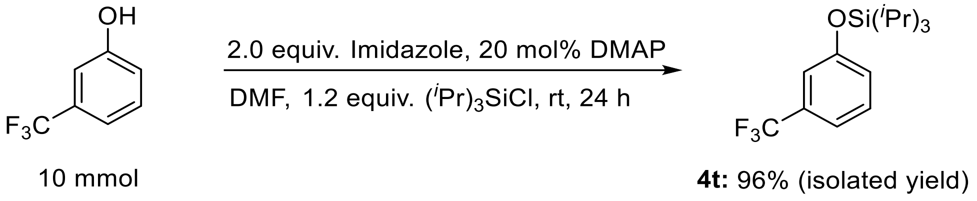

In a dry 100 mL round-bottomed flask 3-(trifluoromethyl)phenol (1.62 g, 10 mmol), imidazole (1.36 g, 20 mmol, 2.0 equiv.), 4-Dimethylaminopyridine (DMAP) (244 mg, 2 mmol, 20 mol%) and 20 mL dry DMF was added. Then stirred for 5 minutes at room temperature followed by dropwise addition of triisopropylsilyl chloride (TIPSCl) (2.5 ml, 12 mmol, 1.2 equiv.) via syringe. The mixture was stirred at the same temperature for additional 24 h. After completion (judged by TLC), the reaction mixture was diluted with cold water (30 mL) and extracted with ethyl acetate (30 mL x 3). The combined organic layer washed with cold water (30 mL x 3), brine (50 mL) and dried over anhydrous Na_2SO_4, filtered and concentrated under reduced pressure. The resulting mixture was purified by silica gel chromatography (2% ethyl acetate in hexane as eluent) gave 3.0 g (96%) triisopropyl(3-(trifluoromethyl)phenoxy)silane **(4t)** as a colourless liquid.

^{1}H NMR (400 MHz, $CDCl_3$): δ 7.33 (t, *J* = 8.0 Hz, 1H), 7.20 (d, *J* = 7.6 Hz, 1H), 7.13 (s, 1H), 7.06 (d, *J* = 8.0 Hz, 1H), 1.33 – 1.25 (m, 3H), 1.12 (d, *J* = 7.2 Hz, 18H).
^{13}C NMR (100 MHz, $CDCl_3$): δ 156.5, 132.0 (q, *J* = 31.9 Hz), 130.0, 124.1 (q, *J* = 270.6 Hz), 123.3 (d, *J* = 1.0 Hz), 117.9 (q, *J* = 3.9 Hz), 117.0 (q, *J* = 3.7 Hz), 18.0, 12.8.
HRMS (ESI) *m/z* calcd for $C_{16}H_{25}F_3OSi$ $[M+H]^+$ 319.1705, found 319.1708.

Synthesis of 3-((triisopropylsilyl)oxy)benzonitrile **(4u)***:*

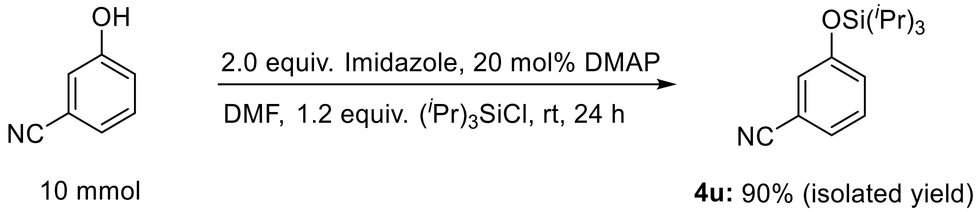

In a dry 100 mL round-bottomed flask 3-hydroxybenzonitrile (1.19 g, 10 mmol), imidazole (1.36 g, 20 mmol, 2.0 equiv.), 4-Dimethylaminopyridine (DMAP) (244 mg, 2 mmol, 20 mol%) and 20 mL dry DMF was added. Then stirred for 5 minutes at room temperature followed by dropwise addition of triisopropylsilyl chloride (TIPSCl) (2.5 ml, 12 mmol, 1.2 equiv.) via syringe. The mixture was stirred at the same temperature for additional 24 h. After completion (judged by TLC), the reaction mixture was diluted with cold water (30 mL) and extracted with ethyl acetate (30 mL x 3). The combined organic layer washed with cold water (30 mL x 3), brine (50 mL) and dried over anhydrous Na_2SO_4, filtered and concentrated under reduced pressure. The resulting mixture was purified by silica gel chromatography (3% ethyl acetate in hexane as eluent) gave 2.5 g (90%) 3-((triisopropylsilyl)oxy)benzonitrile **(4u)** as a colourless liquid.
^{1}H NMR (400 MHz, $CDCl_3$): δ 7.30 (t, *J* = 7.6 Hz, 1H), 7.21 (d, *J* = 7.6 Hz, 1H), 7.11 – 7.08 (m, 2H), 1.29 – 1.22 (m, 3H), 1.08 (d, *J* = 7.2 Hz, 18H).
^{13}C NMR (100 MHz, $CDCl_3$): δ 156.5, 130.5, 125.0, 124.9, 123.3, 118.8, 113.2, 17.9, 12.7.
HRMS (ESI) *m/z* calcd for $C_{16}H_{25}NOSi$ $[M+H]^+$ 276.1784, found 276.1782.

Synthesis of (3-fluorophenoxy)triisopropylsilane **(4v)***:*

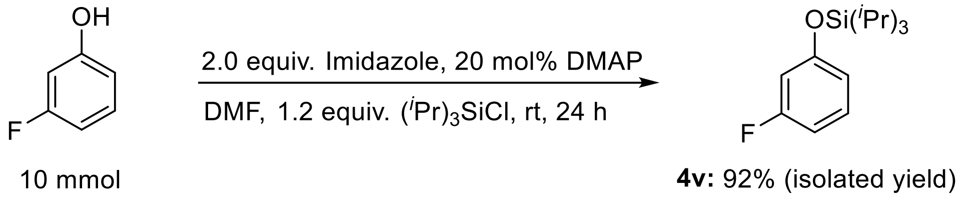

In a dry 100 mL round-bottomed flask 3-fluorophenol (1.12 g, 10 mmol), imidazole (1.36 g, 20 mmol, 2.0 equiv.), 4-Dimethylaminopyridine (DMAP) (244 mg, 2 mmol, 20 mol%) and 20 mL dry DMF was added. Then stirred for 5 minutes at room temperature followed by dropwise addition of triisopropylsilyl chloride (TIPSCl) (2.5 ml, 12 mmol, 1.2 equiv.) via syringe. The mixture was stirred at the same temperature for additional 24 h. After completion (judged by TLC), the reaction mixture was diluted with cold water (30 mL) and extracted with ethyl acetate (30 mL x 3). The combined organic layer washed with cold water (30 mL x 3), brine (50 mL) and dried over anhydrous Na_2SO_4, filtered and concentrated under reduced pressure. The resulting mixture was purified by silica gel chromatography (3% ethyl acetate in hexane as eluent) gave 2.4 g (92%) (3-fluorophenoxy)triisopropylsilane **(4v)** as a colourless liquid.

^{1}H NMR (400 MHz, $CDCl_3$): δ 7.19 – 7.13 (m, 1H), 6.70 – 6.62 (m, 3H), 1.32 – 1.25 (m, 3H), 1.15 – 1.13 (m, 18H).
^{13}C NMR (100 MHz, $CDCl_3$): δ 164.9, 162.4, 157.6 (d, J = 11.1 Hz), 130.0 (d, J = 10.2 Hz), 115.8 (d, J = 2.9 Hz), 107.87 (q, J = 22.5 Hz), 18.0, 12.8.
HRMS (ESI) *m/z* calcd for $C_{15}H_{25}FOSi$ $[M+H]^+$ 269.1737, found 269.1741.

Synthesis of triisopropyl((5,6,7,8-tetrahydronaphthalen-2-yl)oxy)silane **(4w)**:

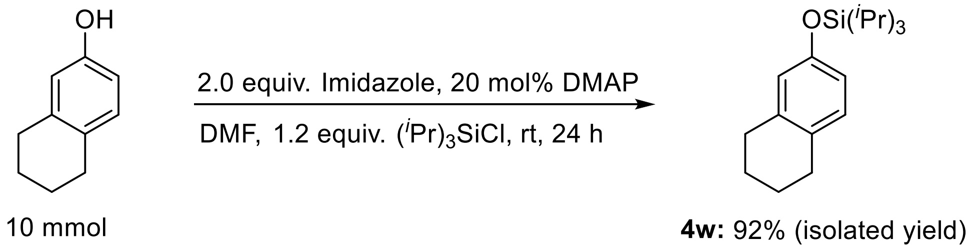

In a dry 100 mL round-bottomed flask 5,6,7,8-tetrahydronaphthalen-2-ol (1.48 g, 10 mmol), imidazole (1.36 g, 20 mmol, 2.0 equiv.), 4-Dimethylaminopyridine (DMAP) (244 mg, 2 mmol, 20 mol%) and 20 mL dry DMF was added. Then stirred for 5 minutes at room temperature followed by dropwise addition of triisopropylsilyl chloride (TIPSCl) (2.5 ml, 12 mmol, 1.2 equiv.) via syringe. The mixture was stirred at the same temperature for additional 24 h. After completion (judged by TLC), the reaction mixture was diluted with cold water (30 mL) and extracted with ethyl acetate (30 mL x 3). The combined organic layer washed with cold water (30 mL x 3), brine (50 mL) and dried over anhydrous Na_2SO_4, filtered and concentrated under reduced pressure. The

resulting mixture was purified by silica gel chromatography (2% ethyl acetate in hexane as eluent) gave 2.8 g (92%) triisopropyl((5,6,7,8-tetrahydronaphthalen-2-yl)oxy)silane **(4w)** as a colourless liquid.

^{1}H NMR (400 MHz, $CDCl_3$): δ 6.92 (d, *J* = 8.0 Hz, 1H), 6.67 – 6.62 (m, 2H), 2.73 (d, *J* = 4.4 Hz, 4H), 1.80 (s, 4H), 1.31 – 1.24 (m, 3H), 1.15 (d, *J* = 7.6 Hz, 18H).
^{13}C NMR (100 MHz, $CDCl_3$): δ 153.7, 138.1, 129.8, 129.5, 119.9, 117.2, 29.7, 28.8, 23.6, 23.3, 18.1, 12.9.
HRMS (ESI) *m/z* calcd for $C_{19}H_{32}OSi$ $[M+H]^+$ 305.2301, found 305.23.

Synthesis of triisopropyl((5,6,7,8-tetrahydronaphthalen-1-yl)oxy)silane **(4x)***:*

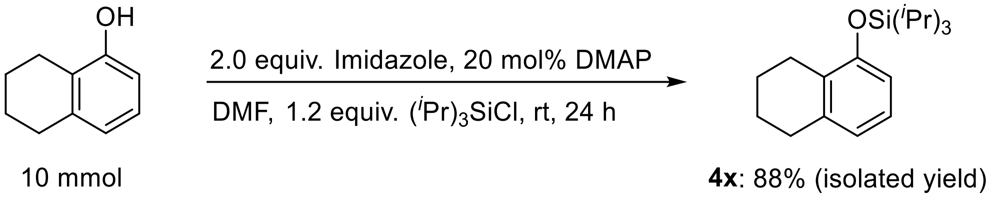

In a dry 100 mL round-bottomed flask 5,6,7,8-tetrahydronaphthalen-1-ol (1.48 g, 10 mmol), imidazole (1.36 g, 20 mmol, 2.0 equiv.), 4-Dimethylaminopyridine (DMAP) (244 mg, 2 mmol, 20 mol%) and 20 mL dry DMF was added. Then stirred for 5 minutes at room temperature followed by dropwise addition of triisopropylsilyl chloride (TIPSCl) (2.5 ml, 12 mmol, 1.2 equiv.) via syringe. The mixture was stirred at the same temperature for additional 24 h. After completion (judged by TLC), the reaction mixture was diluted with cold water (30 mL) and extracted with ethyl acetate (30 mL x 3). The combined organic layer washed with cold water (30 mL x 3), brine (50 mL) and dried over anhydrous Na_2SO_4, filtered and concentrated under reduced pressure. The resulting mixture was purified by silica gel chromatography (2% ethyl acetate in hexane as eluent) gave 2.6 g (88%) triisopropyl((5,6,7,8-tetrahydronaphthalen-1-yl)oxy)silane **(4x)** as a colourless liquid.

^{1}H NMR (400 MHz, $CDCl_3$) δ 7.02 – 6.95 (m, 1H), 6.74 – 6.64 (m, 2H), 2.79 – 2.73 (m, 4H), 1.82 (brs, 4H), 1.40 – 1.31 (m, 3H), 1.20 – 1.15 (m, 18H).
^{13}C NMR (100 MHz, $CDCl_3$): δ 154.0, 138.9, 128.1, 125.5, 121.6, 114.8, 29.9, 24.2, 23.2, 23.1, 18.3, 13.2.
HRMS (ESI) *m/z* calcd for C19H32OSi $[M+H]^+$ 305.2301, found 305.2303.

Synthesis of (2,6-difluorophenoxy)triisopropylsilane **(4y)***:*

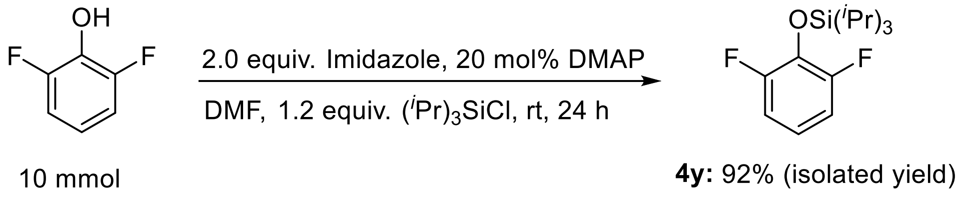

In a dry 100 mL round-bottomed flask 2,6-difluorophenol (1.30 g, 10 mmol), imidazole (1.36 g, 20 mmol, 2.0 equiv.), 4-Dimethylaminopyridine (DMAP) (244 mg, 2 mmol, 20 mol%) and 20 mL dry DMF was added. Then stirred for 5 minutes at room temperature followed by dropwise addition of triisopropylsilyl chloride (TIPSCl) (2.5 ml, 12 mmol, 1.2 equiv.) via syringe. The mixture was stirred at the same temperature for additional 24 h. After completion (judged by TLC), the reaction mixture was diluted with cold water (30 mL) and extracted with ethyl acetate (30 mL x 3). The combined organic layer washed with cold water (30 mL x 3), brine (50 mL) and dried over anhydrous Na_2SO_4, filtered and concentrated under reduced pressure. The resulting mixture was purified by silica gel chromatography (2% ethyl acetate in hexane as eluent) gave 2.3 g (92%) (2,6-difluorophenoxy)triisopropylsilane **(4y)** as a colourless liquid.

^{1}H NMR (400 MHz, $CDCl_3$) δ 6.88 – 6.78 (m, 3H), 1.35 – 1.27 (m, 3H), 1.11 (d, J = 7.6 Hz, 18H).
^{13}C NMR (100 MHz, $CDCl_3$): δ 155.3 (d, J = 243.6 Hz), 155.2 (d, J = 243.6 Hz), 133.5 (t, J = 15.0 Hz), 120.1 (t, J = 9.0 Hz), 111.8 (d, J = 22.7 Hz), 111.8 (q, J = 9.3 Hz), 17.82, 13.02.
HRMS (ESI) *m/z* calcd for $C_{15}H_{24}F_2OSi$ $[M+H]^+$ 287.1643, found 287.1645.

Synthesis of (2,4-dichlorophenoxy)triisopropylsilane **(4z)***:*

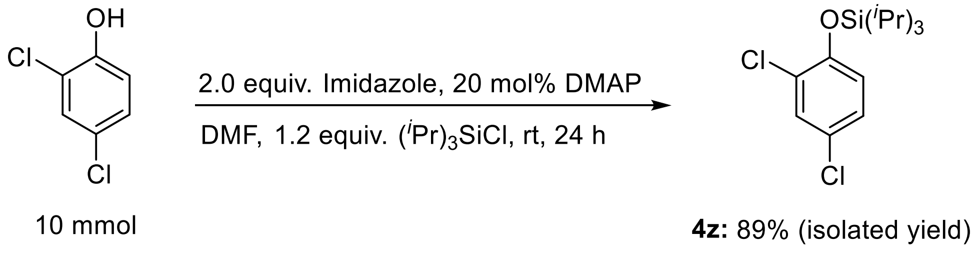

In a dry 100 mL round-bottomed flask 2,4-dichlorophenol (1.61 g, 10 mmol), imidazole (1.36 g, 20 mmol, 2.0 equiv.), 4-Dimethylaminopyridine (DMAP) (244 mg, 2 mmol, 20 mol%) and 20 mL dry DMF was added. Then stirred for 5 minutes at room temperature followed by dropwise addition of triisopropylsilyl chloride (TIPSCl) (2.5 ml, 12 mmol, 1.2 equiv.) via syringe. The mixture was stirred at the same temperature for additional 24 h. After completion (judged by TLC),

the reaction mixture was diluted with cold water (30 mL) and extracted with ethyl acetate (30 mL x 3). The combined organic layer washed with cold water (30 mL x 3), brine (50 mL) and dried over anhydrous Na_2SO_4, filtered and concentrated under reduced pressure. The resulting mixture was purified by silica gel chromatography (2% ethyl acetate in hexane as eluent) gave 2.8 g (89%) (2,4-dichlorophenoxy)triisopropylsilane **(4z)** as a colourless liquid.

^{1}H NMR (400 MHz, $CDCl_3$): δ 7.35 (d, *J* = 2.4 Hz, 1H), 7.08 (dd, *J* = 8.8, 2.8 Hz, 1H), 6.84 (d, *J* = 8.4 Hz, 1H), 1.36 – 1.28 (m, 3H), 1.13 (d, *J* = 7.2 Hz, 18H).
^{13}C NMR (100 MHz, $CDCl_3$): δ 151.0, 130.1, 127.6, 126.2, 125.9, 120.8, 18.0, 13.0.
HRMS (ESI) *m/z* calcd for $C_{15}H_{24}Cl_2OSi$ $[M+H]^+$ 319.1052, found 319.1055.

Synthesis of (2-bromo-4-chlorophenoxy)triisopropylsilane **(4aa):**

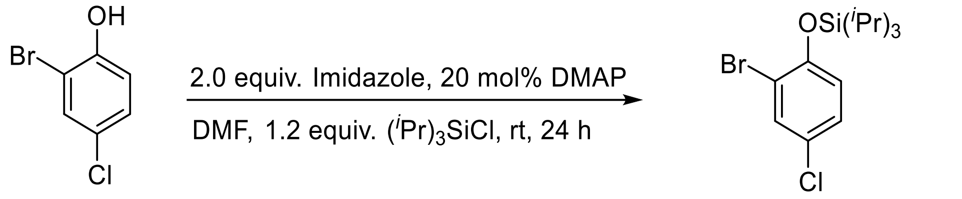

10 mmol

4aa: 87% (isolated yield)

In a dry 100 mL round-bottomed flask 2-bromo-4-chlorophenol (2.05 g, 10 mmol), imidazole (1.36 g, 20 mmol, 2.0 equiv.), 4-Dimethylaminopyridine (DMAP) (244 mg, 2 mmol, 20 mol%) and 20 mL dry DMF was added. Then stirred for 5 minutes at room temperature followed by dropwise addition of triisopropylsilyl chloride (TIPSCl) (2.5 ml, 12 mmol, 1.2 equiv.) via syringe. The mixture was stirred at the same temperature for additional 24 h. After completion (judged by TLC), the reaction mixture was diluted with cold water (30 mL) and extracted with ethyl acetate (30 mL x 3). The combined organic layer washed with cold water (30 mL x 3), brine (50 mL) and dried over anhydrous Na_2SO_4, filtered and concentrated under reduced pressure. The resulting mixture was purified by silica gel chromatography (1% ethyl acetate in hexane as eluent) gave 3.1 g (87%) (2-bromo-4-chlorophenoxy)triisopropylsilane **(4aa)** as a colourless liquid.

^{1}H NMR (400 MHz, $CDCl_3$): δ 7.52 (d, *J* = 2.4 Hz, 1H), 7.13 (dd, *J* = 8.8, 2.8 Hz, 1H), 6.83 (d, *J* = 8.8 Hz, 1H), 1.37 – 1.30 (m, 3H), 1.15 (d, *J* = 7.6 Hz, 18H).
^{13}C NMR (100 MHz, $CDCl_3$): δ 152.0, 133.0, 128.2, 126.1, 120.2, 115.7, 18.1, 13.1.
HRMS (ESI) *m/z* calcd for $C_{15}H_{24}BrClOSi$ $[M+H]^+$ 363.0547, found 363.0550.

Synthesis of (3,4-difluorophenoxy)triisopropylsilane **(4ab)***:*

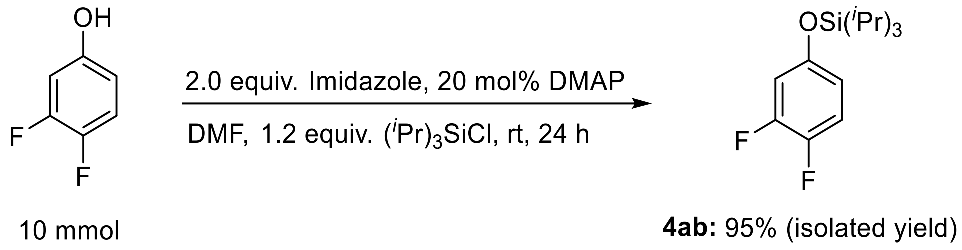

In a dry 100 mL round-bottomed flask 3,4-difluorophenol (1.30 g, 10 mmol), imidazole (1.36 g, 20 mmol, 2.0 equiv.), 4-Dimethylaminopyridine (DMAP) (244 mg, 2 mmol, 20 mol%) and 20 mL dry DMF was added. Then stirred for 5 minutes at room temperature followed by dropwise addition of triisopropylsilyl chloride (TIPSCl) (2.5 ml, 12 mmol, 1.2 equiv.) via syringe. The mixture was stirred at the same temperature for additional 24 h. After completion (judged by TLC), the reaction mixture was diluted with cold water (30 mL) and extracted with ethyl acetate (30 mL x 3). The combined organic layer washed with cold water (30 mL x 3), brine (50 mL) and dried over anhydrous Na_2SO_4, filtered and concentrated under reduced pressure. The resulting mixture was purified by silica gel chromatography (2% ethyl acetate in hexane as eluent) gave 2.7 g (95%) (3,4-difluorophenoxy)triisopropylsilane **(4ab)** as a colourless liquid.

^{1}H NMR (400 MHz, $CDCl_3$) δ 6.99 (q, *J* = 10.0 Hz, 1H), 6.73 – 6.67 (m, 1H), 6.60 – 6.57 (m, 1H), 1.30 – 1.22 (m, 3H), 1.11 (d, *J* = 7.2 Hz, 18H).

^{13}C NMR (100 MHz, $CDCl_3$): δ 152.5 (dd, *J* = 2.6, 9.1 Hz), 150.4 (dd, *J* = 13.7, 246.2 Hz), 145.5 (dd, *J* = 12.7, 238.9 Hz), 117.0 (dd, *J* = 1.8, 18.2 Hz), 115.3 (dd, *J* = 3.3, 5.6 Hz), 109.2 (d, *J* = 18.1 Hz), 17.9, 12.7.

HRMS (ESI) *m/z* calcd for $C_{15}H_{24}F_2OSi$ $[M+H]^+$ 287.1643, found 287.1645.

Synthesis of (4-fluoro-2-methoxyphenoxy)triisopropylsilane **(4ac)***:*

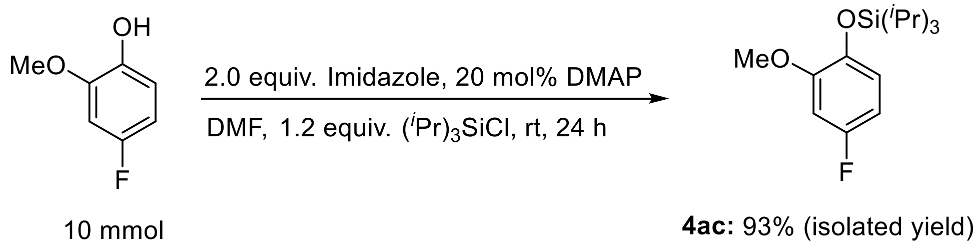

In a dry 100 mL round-bottomed flask 4-fluoro-2-methoxyphenol (1.42 g, 10 mmol), imidazole (1.36 g, 20 mmol, 2.0 equiv.), 4-Dimethylaminopyridine (DMAP) (244 mg, 2 mmol, 20 mol%)

and 20 mL dry DMF was added. Then stirred for 5 minutes at room temperature followed by dropwise addition of triisopropylsilyl chloride (TIPSCl) (2.5 ml, 12 mmol, 1.2 equiv.) via syringe. The mixture was stirred at the same temperature for additional 24 h. After completion (judged by TLC), the reaction mixture was diluted with cold water (30 mL) and extracted with ethyl acetate (30 mL x 3). The combined organic layer washed with cold water (30 mL x 3), brine (50 mL) and dried over anhydrous Na_2SO_4, filtered and concentrated under reduced pressure. The resulting mixture was purified by silica gel chromatography (1% ethyl acetate in hexane as eluent) gave 2.7 g (93%) (4-fluoro-2-methoxyphenoxy)triisopropylsilane **(4ac)** as a colourless liquid.

^{1}H NMR (400 MHz, $CDCl_3$): δ 6.80 (dd, *J* = 8.8, 5.6 Hz, 1H), 6.60 (dd, *J* = 10.0, 2.8 Hz, 1H), 6.50 (td, *J* = 8.4, 2.8 Hz, 1H), 3.79 (s, 3H), 1.29 – 1.22 (m, 3H), 1.10 (d, *J* = 7.2 Hz, 18H).
^{13}C NMR (100 MHz, $CDCl_3$): δ 157.6 (d, *J* = 237.1 Hz), 151.5 (d, *J* = 9.9 Hz), 141.7 (d, *J* = 3.0 Hz), 120.2 (d, *J* = 9.6 Hz), 106.1 (d, *J* = 22.2 Hz), 100.3 (d, *J* = 26.9 Hz), 55.6, 18.0, 12.9.
HRMS (ESI) *m/z* calcd for C16H27FO2Si $[M+H]^+$ 299.1843, found 299.1845.

Synthesis of (2,3-dimethoxyphenoxy)triisopropylsilane **(4ad)***:*

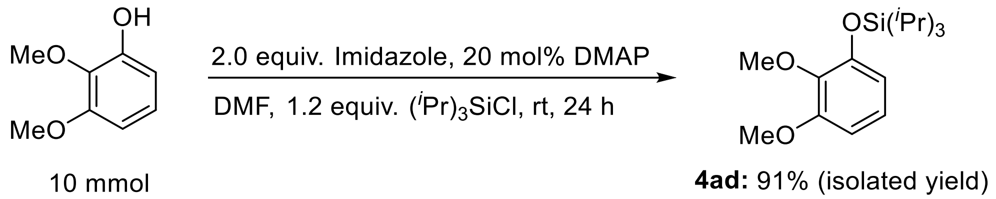

In a dry 100 mL round-bottomed flask 2,3-dimethoxyphenol (1.54 g, 10 mmol), imidazole (1.36 g, 20 mmol, 2.0 equiv.), 4-Dimethylaminopyridine (DMAP) (244 mg, 2 mmol, 20 mol%) and 20 mL dry DMF was added. Then stirred for 5 minutes at room temperature followed by dropwise addition of triisopropylsilyl chloride (TIPSCl) (2.5 ml, 12 mmol, 1.2 equiv.) via syringe. The mixture was stirred at the same temperature for additional 24 h. After completion (judged by TLC), the reaction mixture was diluted with cold water (30 mL) and extracted with ethyl acetate (30 mL x 3). The combined organic layer washed with cold water (30 mL x 3), brine (50 mL) and dried over anhydrous Na_2SO_4, filtered and concentrated under reduced pressure. The resulting mixture was purified by silica gel chromatography (1% ethyl acetate in hexane as eluent) gave 2.8 g (91%) (2,3-dimethoxyphenoxy)triisopropylsilane **(4ad)** as a colourless liquid.

^{1}H NMR (400 MHz, $CDCl_3$): δ 6.86 (t, J = 8.4 Hz, 1H), 6.53 (dd, J = 8.4, 1.6 Hz, 2H), 3.84 (s, 3H), 3.81 (s, 3H), 1.33 – 1.7 (m, 3H), 1.11 (d, J = 7.2 Hz, 18H).
^{13}C NMR (100 MHz, $CDCl_3$): δ 154.1, 150.2, 140.4, 123.3, 113.7, 105.2, 60.6, 56.0, 18.0, 12.9.
HRMS (ESI) *m/z* calcd for $C_{17}H_{30}O_3Si$ $[M+H]^+$ 311.2042, found 311.2030.

Synthesis of (2,3-dimethylphenoxy)triisopropylsilane **(4ae)***:*

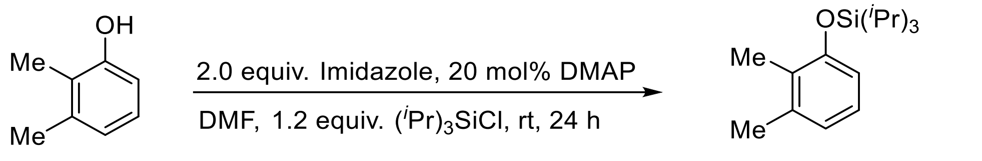

4ae: 89% (isolated yield)

In a dry 100 mL round-bottomed flask 2,3-dimethylphenol (1.22 g, 10 mmol), imidazole (1.36 g, 20 mmol, 2.0 equiv.), 4-Dimethylaminopyridine (DMAP) (244 mg, 2 mmol, 20 mol%) and 20 mL dry DMF was added. Then stirred for 5 minutes at room temperature followed by dropwise addition of triisopropylsilyl chloride (TIPSCl) (2.5 ml, 12 mmol, 1.2 equiv.) via syringe. The mixture was stirred at the same temperature for additional 24 h. After completion (judged by TLC), the reaction mixture was diluted with cold water (30 mL) and extracted with ethyl acetate (30 mL x 3). The combined organic layer washed with cold water (30 mL x 3), brine (50 mL) and dried over anhydrous Na_2SO_4, filtered and concentrated under reduced pressure. The resulting mixture was purified by silica gel chromatography (1% ethyl acetate in hexane as eluent) gave 2.4 g (89%) ((2,3-dimethylphenoxy)triisopropylsilane **(4ae)** as a colourless liquid.

^{1}H NMR (400 MHz, $CDCl_3$): δ 7.04 – 7.00 (m, 1H), 6.86 – 6.76 (m, 2H), 2.37 – 2.25 (m, 6H), 1.43 – 1.37 (m, 3H), 1.26 – 1.21 (m, 18H).
^{13}C NMR (100 MHz, $CDCl_3$): δ 154.2, 138.2, 127.1, 125.6, 122.5, 115.8, 20.5, 18.2, 13.3, 12.6.
HRMS (ESI) *m/z* calcd for $C_{17}H_{30}OSi$ $[M+H]^+$ 279.2144, found 279.2135.

Synthesis of (4-chloro-3-methylphenoxy)triisopropylsilane **(4af)***:*

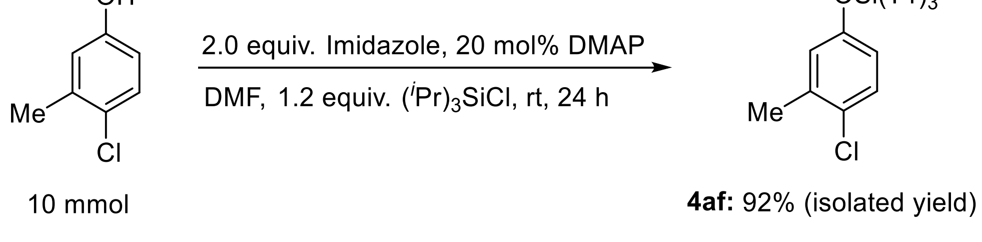

4af: 92% (isolated yield)

In a dry 100 mL round-bottomed flask 4-chloro-3-methylphenol (1.42 g, 10 mmol), imidazole (1.36 g, 20 mmol, 2.0 equiv.), 4-Dimethylaminopyridine (DMAP) (244 mg, 2 mmol, 20 mol%) and 20 mL dry DMF was added. Then stirred for 5 minutes at room temperature followed by dropwise addition of triisopropylsilyl chloride (TIPSCl) (2.5 ml, 12 mmol, 1.2 equiv.) via syringe. The mixture was stirred at the same temperature for additional 24 h. After completion (judged by TLC), the reaction mixture was diluted with cold water (30 mL) and extracted with ethyl acetate (30 mL x 3). The combined organic layer washed with cold water (30 mL x 3), brine (50 mL) and dried over anhydrous Na_2SO_4, filtered and concentrated under reduced pressure. The resulting mixture was purified by silica gel chromatography (1% ethyl acetate in hexane as eluent) gave 2.7 g (92%) (4-chloro-3-methylphenoxy)triisopropylsilane **(4af)** as a colourless liquid.

^{1}H NMR (400 MHz, $CDCl_3$): δ 7.17 (d, *J* = 8.4 Hz, 1H), 6.78 (d, *J* = 2.0 Hz, 1H), 6.67 (dd, *J* = 8.8, 2.8 Hz, 1H), 2.33 (s, 3H), 1.31 – 1.23 (m, 3H), 1.12 (d, *J* = 7.2 Hz, 18H).
^{13}C NMR (100 MHz, $CDCl_3$): δ 154.7, 137.0, 129.6, 126.2, 122.4, 118.6, 20.3, 18.0, 12.8.
HRMS (ESI) *m/z* calcd for $C_{16}H_{27}ClOSi$ $[M+H]^+$ 299.1598, found 299.1598.

Synthesis of 2,2'-bis((triisopropylsilyl)oxy)-1,1'-biphenyl **(4ag)**:

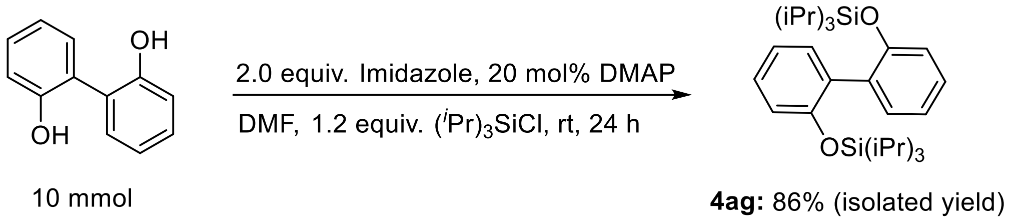

In a dry 100 mL round-bottomed flask [1,1'-biphenyl]-2,2'-diol (1.86 g, 10 mmol), imidazole (2.72 g, 40 mmol, 4.0 equiv.), 4-Dimethylaminopyridine (DMAP) (488 mg, 4 mmol, 40 mol%) and 40 mL dry DMF was added. Then stirred for 5 minutes at room temperature followed by dropwise addition of triisopropylsilyl chloride (TIPSCl) (5 ml, 24 mmol, 1.2 equiv.) via syringe. The mixture was stirred at the same temperature for additional 24 h. After completion (judged by TLC), the reaction mixture was diluted with cold water (30 mL) and extracted with ethyl acetate (30 mL x 3). The combined organic layer washed with cold water (30 mL x 3), brine (50 mL) and dried over anhydrous Na_2SO_4, filtered and concentrated under reduced pressure. The resulting mixture was purified by silica gel chromatography (1% ethyl acetate in hexane as eluent) gave 4.2 g (86%) 2,2'-bis((triisopropylsilyl)oxy)-1,1'-biphenyl **(4ag)** as a white solid.

^{1}H NMR (400 MHz, $CDCl_3$): δ 7.24 – 7.21 (m, 2H), 7.17 – 7.13 (m, 2H), 6.95 – 6.90 (m, 2H), 6.88 – 6.85 (m, 2H), 1.16 – 1.07 (m, 6H), 0.95 – 0.92 (m, 36H).

^{13}C NMR (100 MHz, $CDCl_3$): δ 153.7, 132.1, 130.7, 128.0, 120.2, 118.8, 18.0, 13.0.

HRMS (ESI) *m/z* calcd for $C_{30}H_{50}O_2Si_2$ $[M+H]^+$ 499.3428, found 499.3428.

Synthesis of triisopropyl(4-(1-methoxyethyl)phenoxy)silane **(4ah)**:

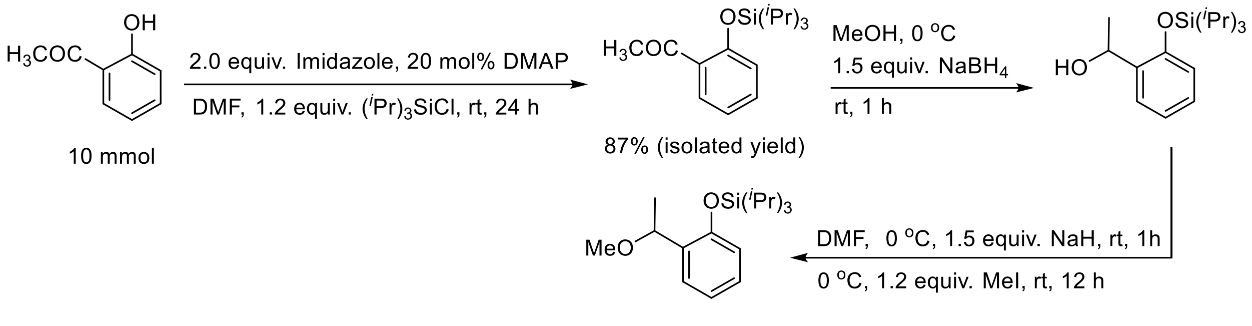

4ah: 65% (isolated yield)

Step I: In a dry 100 mL round-bottomed flask 1-(2-hydroxyphenyl)ethan-1-one (1.36g, 10 mmol), imidazole (1.36 g, 20 mmol, 2.0 equiv.), 4-Dimethylaminopyridine (DMAP) (244 mg, 2 mmol, 20 mol%) and 20 mL dry DMF was added. Then stirred for 5 minutes at room temperature followed by dropwise addition of triisopropylsilyl chloride (TIPSCl) (2.5 ml, 12 mmol, 1.2 equiv.) via syringe. The mixture was stirred at the same temperature for additional 24 h. After completion (judged by TLC), the reaction mixture was diluted with cold water (30 mL) and extracted with ethyl acetate (30 mL x 3). The combined organic layer washed with cold water (30 mL x 3), brine (50 mL) and dried over anhydrous Na_2SO_4, filtered and concentrated under reduced pressure. The resulting mixture was purified by silica gel chromatography (7% ethyl acetate in hexane as eluent) gave 2.5 g (87%) 1-(2-((triisopropylsilyl)oxy)phenyl)ethan-1-one as a colourless liquid. Spectral data are in accordance with the reported data.[23]

Step II: An oven dried 100 mL round-bottom flask was charged with 1-(2-((triisopropylsilyl)oxy)phenyl)ethan-1-one (2.5 g, 8.5 mmol), MeOH (20.0 mL) and cool to 0 ^{o}C. solid sodium borohydride (472 mg, 1.5 equiv.) was added portionwise over 30 minutes and stirred for another 1 h at room temperature. After completion (monitored by TLC), MeOH was evaporated under reduced pressure, diluted with water (20 mL) and extracted with ethyl acetate (30 mL x 3). The combined organic layer was washed with brine (50 mL), dried over anhydrous Na_2SO_4 , filterd

and concentrated under reduced pressure to get the 1-(2-((triisopropylsilyl)oxy)phenyl)ethan-1-ol as a gummy liquid which was used directly for the next step without furthur purification.

Step III: In a 100 mL round-bottom flask crude 1-(2-((triisopropylsilyl)oxy)phenyl)ethan-1-ol was dissolved in DMF (20 mL), cool to 0 °C and then NaH (586 mg, 1.5 equiv. 50 % dispersion in mineral oil) was added portion wise at 0 °C and stirred for 1 h at room temperature. Then MeI (610 µL, 1.2 equiv.) was added at 0 °C and stirred it for 12 h at room temperature. After completion (judged by TLC), the reaction mixture was diluted with cold water (30 mL) and extracted with ethyl acetate (30 mL x 3). The combined organic layer washed with cold water (30 mL x 3), brine (50 mL) and dried over anhydrous Na_2SO_4, filtered and concentrated under reduced pressure. . The resulting mixture was purified by silica gel chromatography (2% ethyl acetate in hexane as eluent) gave 1.7 g (65%) triisopropyl(2-(1-methoxyethyl)phenoxy)silane **(4ah)** as a colourless liquid.

^{1}H NMR (400 MHz, $CDCl_3$): δ 7.61 (d, *J* = 7.6 Hz, 1H), 7.23 – 7.18 (m, 1H), 6.98 (t, *J* = 7.2 Hz, 1H), 6.83 (d, *J* = 8.0 Hz, 1H), 5.34 (q, *J* = 6.0 Hz, 1H), 3.82 (s, 3H), 1.40 (d, *J* = 6.0 Hz, 3H), 1.10 – 1.01 (m, 21H).
^{13}C NMR (100 MHz, $CDCl_3$): δ 155.1, 136.0, 127.4, 126.3, 120.7, 109.9, 64.9, 55.3, 26.2, 18.2, 12.4.
HRMS (ESI) *m/z* calcd for $C_{18}H_{32}O_2Si$ $[M+H]^+$ 309.2250, found 309.2250.

Synthesis of triisopropyl(p-tolyloxy)silane **(6a)***:*

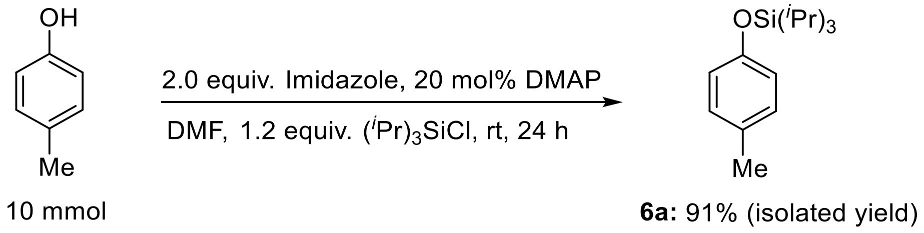

In a dry 100 mL round-bottomed flask p-cresol (1.08 g, 10 mmol), imidazole (1.36 g, 20 mmol, 2.0 equiv.), 4-Dimethylaminopyridine (DMAP) (244 mg, 2 mmol, 20 mol%) and 20 mL dry DMF was added. Then stirred for 5 minutes at room temperature followed by dropwise addition of triisopropylsilyl chloride (TIPSCl) (2.5 ml, 12 mmol, 1.2 equiv.) via syringe. The mixture was stirred at the same temperature for additional 24 h. After completion (judged by TLC), the reaction mixture was diluted with cold water (30 mL) and extracted with ethyl acetate (30 mL x 3). The

combined organic layer washed with cold water (30 mL x 3), brine (50 mL) and dried over anhydrous Na_2SO_4, filtered and concentrated under reduced pressure. The resulting mixture was purified by silica gel chromatography (2% ethyl acetate in hexane as eluent) gave 2.4 g (91%) triisopropyl(p-tolyloxy)silane **(6a)** as a colourless liquid. Spectral data are in accordance with the reported data.[26]

Synthesis of (4-ethylphenoxy)triisopropylsilane **(6b)***:*

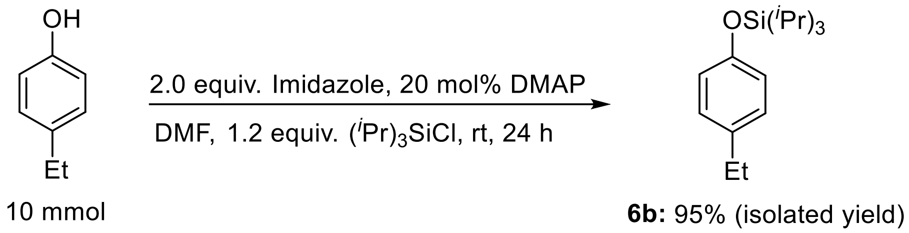

In a dry 100 mL round-bottomed flask 4-ethylphenol (1.22 g, 10 mmol), imidazole (1.36 g, 20 mmol, 2.0 equiv.), 4-Dimethylaminopyridine (DMAP) (244 mg, 2 mmol, 20 mol%) and 20 mL dry DMF was added. Then stirred for 5 minutes at room temperature followed by dropwise addition of triisopropylsilyl chloride (TIPSCl) (2.5 ml, 12 mmol, 1.2 equiv.) via syringe. The mixture was stirred at the same temperature for additional 24 h. After completion (judged by TLC), the reaction mixture was diluted with cold water (30 mL) and extracted with ethyl acetate (30 mL x 3). The combined organic layer washed with cold water (30 mL x 3), brine (50 mL) and dried over anhydrous Na_2SO_4, filtered and concentrated under reduced pressure. The resulting mixture was purified by silica gel chromatography (1% ethyl acetate in hexane as eluent) gave 2.6 g (95%) (4-ethylphenoxy)triisopropylsilane **(6b)** as a colourless liquid. Spectral data are in accordance with the reported data.[27]

Synthesis of triisopropyl(4-pentylphenoxy)silane **(6c)***:*

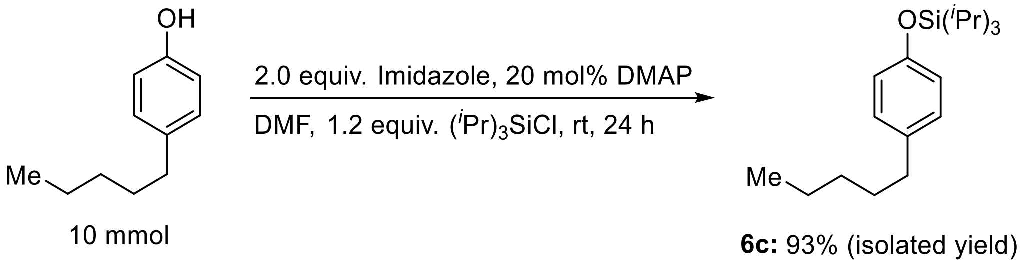

In a dry 100 mL round-bottomed flask 4-pentylphenol (1.64 g, 10 mmol), imidazole (1.36 g, 20 mmol, 2.0 equiv.), 4-Dimethylaminopyridine (DMAP) (244 mg, 2 mmol, 20 mol%) and 20 mL

dry DMF was added. Then stirred for 5 minutes at room temperature followed by dropwise addition of triisopropylsilyl chloride (TIPSCl) (2.5 ml, 12 mmol, 1.2 equiv.) via syringe. The mixture was stirred at the same temperature for additional 24 h. After completion (judged by TLC), the reaction mixture was diluted with cold water (30 mL) and extracted with ethyl acetate (30 mL x 3). The combined organic layer washed with cold water (30 mL x 3), brine (50 mL) and dried over anhydrous Na_2SO_4, filtered and concentrated under reduced pressure. The resulting mixture was purified by silica gel chromatography (1% ethyl acetate in hexane as eluent) gave 3.1 g (93%) triisopropyl(4-pentylphenoxy)silane **(6c)** as a colourless liquid.

^{1}H NMR (400 MHz, $CDCl_3$): δ 7.04 (d, *J* = 8.4 Hz, 2H), 6.82 (d, *J* = 8.4 Hz, 2H), 2.56 (t, *J* = 8.0 Hz, 2H), 1.65 – 1.57 (m, 2H), 1.37 – 1.25 (m, 7H), 1.13 (d, *J* = 7.2 Hz, 18H), 0.92 (t, *J* = 6.8 Hz, 3H).
^{13}C NMR (100 MHz, $CDCl_3$): δ 154.0, 135.5, 129.3, 119.7, 35.3, 31.7, 31.5, 22.7, 18.1, 14.2, 12.8.
HRMS (ESI) *m/z* calcd for $C_{20}H_{36}OSi$ $[M+H]^+$ 321.2614, found 321.2610.

Synthesis of (4-hexylphenoxy)triisopropylsilane **(6d)***:*

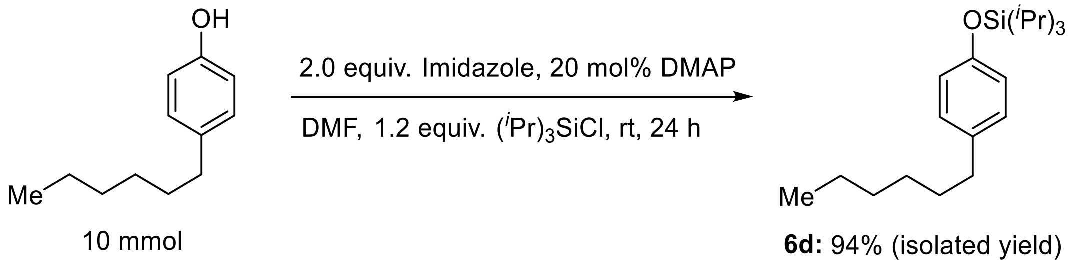

In a dry 100 mL round-bottomed flask 4-hexylphenol (1.78 g, 10 mmol), imidazole (1.36 g, 20 mmol, 2.0 equiv.), 4-Dimethylaminopyridine (DMAP) (244 mg, 2 mmol, 20 mol%) and 20 mL dry DMF was added. Then stirred for 5 minutes at room temperature followed by dropwise addition of triisopropylsilyl chloride (TIPSCl) (2.5 ml, 12 mmol, 1.2 equiv.) via syringe. The mixture was stirred at the same temperature for additional 24 h. After completion (judged by TLC), the reaction mixture was diluted with cold water (30 mL) and extracted with ethyl acetate (30 mL x 3). The combined organic layer washed with cold water (30 mL x 3), brine (50 mL) and dried over anhydrous Na_2SO_4, filtered and concentrated under reduced pressure. The resulting mixture was purified by silica gel chromatography (1% ethyl acetate in hexane as eluent) gave 3.1 g (94%) (4-hexylphenoxy)triisopropylsilane **(6d)** as a colourless liquid.

^{1}H NMR (400 MHz, $CDCl_3$): δ 7.03 (d, *J* = 8.0 Hz, 2H), 6.83 – 6.81 (m, 2H), 2.55 (t, *J* = 7.6 Hz, 2H), 1.60 (brs, 2H), 1.32 – 1.25 (m, 9H), 1.13 (d, *J* = 7.2 Hz, 18H), 0.91 (brs, 3H).
^{13}C NMR (100 MHz, $CDCl_3$): δ 154.0, 135.5, 129.3, 119.7, 35.3, 31.9, 31.8, 29.1, 22.8, 18.1, 14.2, 12.8.
HRMS (ESI) *m/z* calcd for $C_{21}H_{38}OSi$ $[M+H]^+$ 335.2770, found 335.2761.

Synthesis of triisopropyl(4-isopropylphenoxy)silane **(6e)***:*

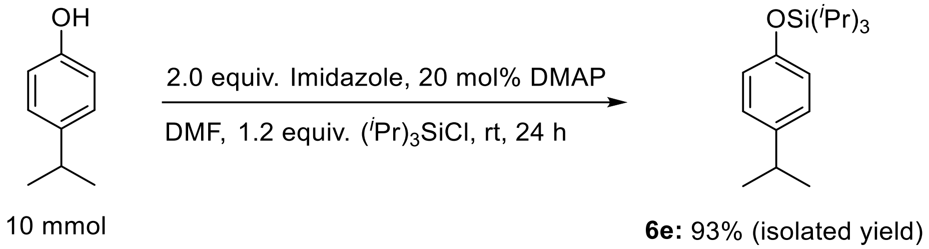

In a dry 100 mL round-bottomed flask 4-isopropylphenol (1.36 g, 10 mmol), imidazole (1.36 g, 20 mmol, 2.0 equiv.), 4-Dimethylaminopyridine (DMAP) (244 mg, 2 mmol, 20 mol%) and 20 mL dry DMF was added. Then stirred for 5 minutes at room temperature followed by dropwise addition of triisopropylsilyl chloride (TIPSCl) (2.5 ml, 12 mmol, 1.2 equiv.) via syringe. The mixture was stirred at the same temperature for additional 24 h. After completion (judged by TLC), the reaction mixture was diluted with cold water (30 mL) and extracted with ethyl acetate (30 mL x 3). The combined organic layer washed with cold water (30 mL x 3), brine (50 mL) and dried over anhydrous Na_2SO_4, filtered and concentrated under reduced pressure. The resulting mixture was purified by silica gel chromatography (1% ethyl acetate in hexane as eluent) gave 2.7 g (93%) triisopropyl(4-isopropylphenoxy)silane **(6e)** as a colourless liquid.

^{1}H NMR (400 MHz, $CDCl_3$): δ 7.09 (d, *J* = 8.4 Hz, 2H), 6.83 (d, *J* = 8.4 Hz, 2H), 2.92 – 2.82 (m, 1H), 1.32 – 1.27 (m, 3H), 1.24 (d, *J* = 7.2 Hz, 6H), 1.13 (d, *J* = 7.2 Hz, 18H).
^{13}C NMR (100 MHz, $CDCl_3$): δ 154.0, 141.4, 127.2, 119.6, 33.4, 24.4, 18.1, 12.8.
HRMS (ESI) *m/z* calcd for $C_{18}H_{32}OSi$ $[M+H]^+$ 293.2301, found 293.2301.

Synthesis of triisopropyl(4-methoxyphenoxy)silane **(6f)***:*

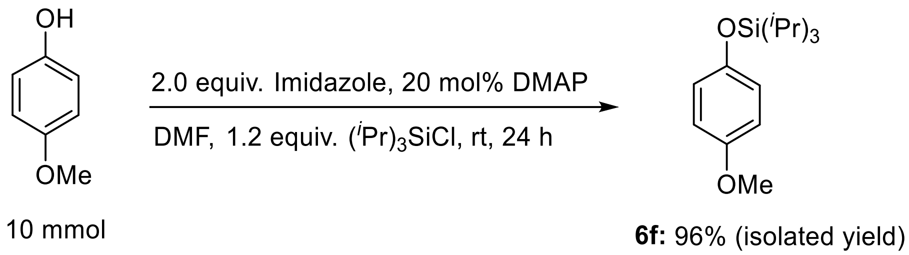

6f: 96% (isolated yield)

In a dry 100 mL round-bottomed flask 4-methoxyphenol (1.24 g, 10 mmol), imidazole (1.36 g, 20 mmol, 2.0 equiv.), 4-Dimethylaminopyridine (DMAP) (244 mg, 2 mmol, 20 mol%) and 20 mL dry DMF was added. Then stirred for 5 minutes at room temperature followed by dropwise addition of triisopropylsilyl chloride (TIPSCl) (2.5 ml, 12 mmol, 1.2 equiv.) via syringe. The mixture was stirred at the same temperature for additional 24 h. After completion (judged by TLC), the reaction mixture was diluted with cold water (30 mL) and extracted with ethyl acetate (30 mL x 3). The combined organic layer washed with cold water (30 mL x 3), brine (50 mL) and dried over anhydrous Na_2SO_4, filtered and concentrated under reduced pressure. The resulting mixture was purified by silica gel chromatography (2% ethyl acetate in hexane as eluent) gave 2.6 g (96%) triisopropyl(4-methoxyphenoxy)silane **(6f)** as a colourless liquid. Spectral data are in accordance with the reported data.[28]

Synthesis of triisopropyl(4-propoxyphenoxy)silane **(6g)***:*

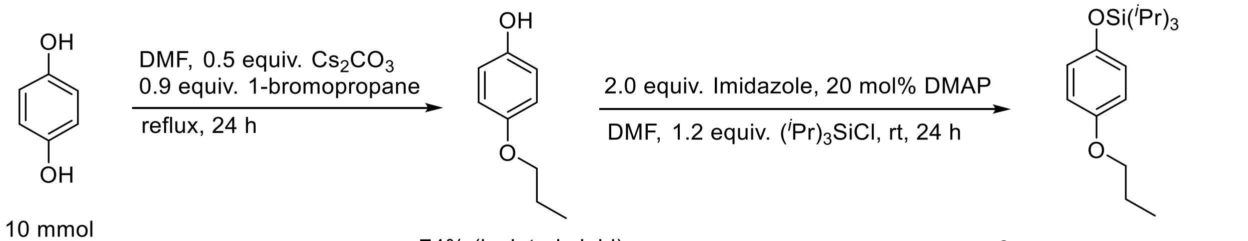

6g: 95% (isolated yield)

Step I: A dry 100 mL round-bottomed flask was charged with hydroquinone (1.1 g, 10 mmol), Cs_2CO_3 (1.6 g, 5 mmol, 0.5 equiv.) in dry DMF (20 mL). The reaction mixture was stirred for 2 h at room temperature and then 1-bromopropane (0.8 mL, 9 mmol, 0.9 equiv.) added dropwise via syringe at room temperature. Then cool to room temperature, diluted with cold water (30 mL) and extracted with ethyl acetate (30 mL x 3). The combined organic layer washed with cold water (30 mL x 3), brine (50 mL) and dried over anhydrous Na_2SO_4, filtered and concentrated under reduced

pressure. The crude mass was purified by silica gel column chromatography (10% ethyl acetate in hexane as eluent) to give 4-propoxyphenol (1.1 g, 74%) as a white solid. Spectral data are in accordance with the reported data.[29]

Step II: In a dry 100 mL round-bottomed flask 4-propoxyphenol (1.1 g, 7.2 mmol), imidazole (0.97 g, 14.6 mmol, 2.0 equiv.), 4-Dimethylaminopyridine (DMAP) (177 mg, 1.46 mmol, 20 mol%) and 20 mL dry DMF was added. Then stirred for 5 minutes at room temperature followed by dropwise addition of triisopropylsilyl chloride (TIPSCl) (1.83 ml, 8.76 mmol, 1.2 equiv.) via syringe. The mixture was stirred at the same temperature for additional 24 h. After completion (judged by TLC), the reaction mixture was diluted with cold water (30 mL) and extracted with ethyl acetate (30 mL x 3). The combined organic layer washed with cold water (30 mL x 3), brine (50 mL) and dried over anhydrous Na_2SO_4, filtered and concentrated under reduced pressure. The resulting mixture was purified by silica gel chromatography (1% ethyl acetate in hexane as eluent) gave 2.1 g (95%) triisopropyl(4-propoxyphenoxy)silane **(6g)** as a colourless liquid.

^{1}H NMR (400 MHz, $CDCl_3$): δ 6.80 (q, J = 9.2 Hz, 4H), 3.87 (t, J = 6.4 Hz, 2H), 1.85 – 1.76 (m, 2H), 1.31 – 1.23 (m, 3H), 1.13 (d, J = 7.2 Hz, 18H), 1.05 (t, J = 7.6 Hz, 3H).
^{13}C NMR (100 MHz, $CDCl_3$): δ 153.5, 149.8, 120.4, 115.2, 70.1, 22.9, 18.0, 12.7, 10.7.
HRMS (ESI) *m/z* calcd for $C_{18}H_{32}O_2Si$ $[M+H]^+$ 309.2250, found 309.2248.

Synthesis of triisopropyl(4-(trifluoromethoxy)phenoxy)silane **(6h)***:*

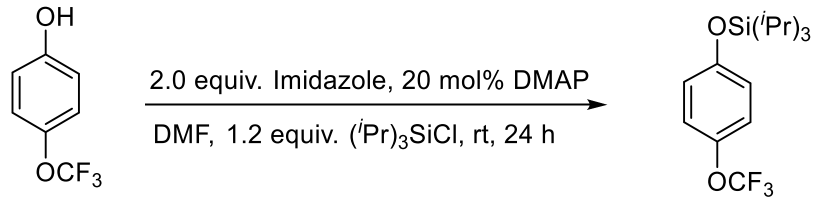

10 mmol

6h: 86% (isolated yield)

In a dry 100 mL round-bottomed flask 4-(trifluoromethoxy)phenol (1.78 g, 10 mmol), imidazole (1.36 g, 20 mmol, 2.0 equiv.), 4-Dimethylaminopyridine (DMAP) (244 mg, 2 mmol, 20 mol%) and 20 mL dry DMF was added. Then stirred for 5 minutes at room temperature followed by dropwise addition of triisopropylsilyl chloride (TIPSCl) (2.5 ml, 12 mmol, 1.2 equiv.) via syringe. The mixture was stirred at the same temperature for additional 24 h. After completion (judged by TLC), the reaction mixture was diluted with cold water (30 mL) and extracted with ethyl acetate

(30 mL x 3). The combined organic layer washed with cold water (30 mL x 3), brine (50 mL) and dried over anhydrous Na_2SO_4, filtered and concentrated under reduced pressure. The resulting mixture was purified by silica gel chromatography (2% ethyl acetate in hexane as eluent) gave 2.8 g (86%) triisopropyl(4-(trifluoromethoxy)phenoxy)silane **(6h)** as a colourless liquid. Spectral data are in accordance with the reported data.[13]

Synthesis of triisopropyl(4-((trifluoromethyl)thio)phenoxy)silane **(6i)***:*

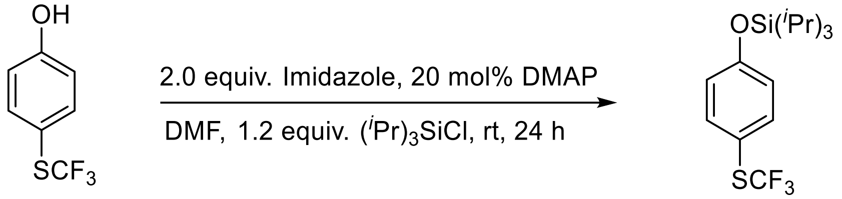

10 mmol

6i: 88% (isolated yield)

In a dry 100 mL round-bottomed flask 4-((trifluoromethyl)thio)phenol (1.94 g, 10 mmol), imidazole (1.36 g, 20 mmol, 2.0 equiv.), 4-Dimethylaminopyridine (DMAP) (244 mg, 2 mmol, 20 mol%) and 20 mL dry DMF was added. Then stirred for 5 minutes at room temperature followed by dropwise addition of triisopropylsilyl chloride (TIPSCl) (2.5 ml, 12 mmol, 1.2 equiv.) via syringe. The mixture was stirred at the same temperature for additional 24 h. After completion (judged by TLC), the reaction mixture was diluted with cold water (30 mL) and extracted with ethyl acetate (30 mL x 3). The combined organic layer washed with cold water (30 mL x 3), brine (50 mL) and dried over anhydrous Na_2SO_4, filtered and concentrated under reduced pressure. The resulting mixture was purified by silica gel chromatography (2% ethyl acetate in hexane as eluent) gave 3.1 g (88%) triisopropyl(4-((trifluoromethyl)thio)phenoxy)silane **(6i)** as a colourless liquid.

^{1}H NMR (400 MHz, $CDCl_3$): δ 7.52 (d, *J* = 8.4 Hz, 2H), 6.92 (d, *J* = 8.8 Hz, 2H), 1.33 – 1.26 (m, 3H), 1.12 (d, *J* = 7.6 Hz, 18H).

^{13}C NMR (100 MHz, $CDCl_3$): δ 159.0, 138.4, 129.8 (q, *J* = 306.1 Hz), 121.1, 115.4, 18.0, 12.8.

HRMS (ESI) *m/z* calcd for $C_{16}H_{25}F_3OSSi$ $[M+H]^+$ 351.1426, found 351.1428.

Synthesis of triisopropyl(4-(methylthio)phenoxy)silane **(6j)***:*

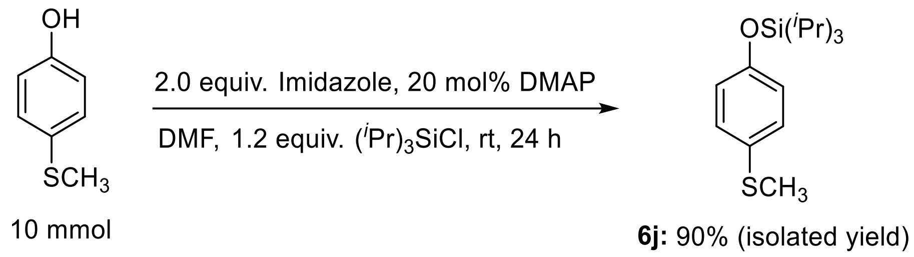

In a dry 100 mL round-bottomed flask 4-(methylthio)phenol (1.40 g, 10 mmol), imidazole (1.36 g, 20 mmol, 2.0 equiv.), 4-Dimethylaminopyridine (DMAP) (244 mg, 2 mmol, 20 mol%) and 20 mL dry DMF was added. Then stirred for 5 minutes at room temperature followed by dropwise addition of triisopropylsilyl chloride (TIPSCl) (2.5 ml, 12 mmol, 1.2 equiv.) via syringe. The mixture was stirred at the same temperature for additional 24 h. After completion (judged by TLC), the reaction mixture was diluted with cold water (30 mL) and extracted with ethyl acetate (30 mL x 3). The combined organic layer washed with cold water (30 mL x 3), brine (50 mL) and dried over anhydrous Na_2SO_4, filtered and concentrated under reduced pressure. The resulting mixture was purified by silica gel chromatography (3% ethyl acetate in hexane as eluent) gave 2.6 g (90%) triisopropyl(4-(methylthio)phenoxy)silane **(6j)** as a colourless liquid. Spectral data are in accordance with the reported data.[30]

Synthesis of 4-((triisopropylsilyl)oxy)benzonitrile **(6k):**

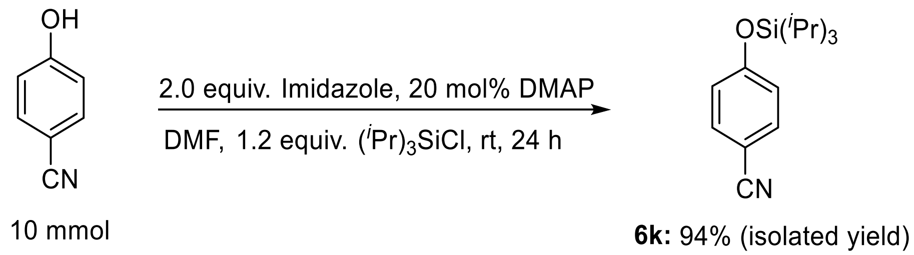

In a dry 100 mL round-bottomed flask 4-hydroxybenzonitrile (1.19 g, 10 mmol), imidazole (1.36 g, 20 mmol, 2.0 equiv.), 4-Dimethylaminopyridine (DMAP) (244 mg, 2 mmol, 20 mol%) and 20 mL dry DMF was added. Then stirred for 5 minutes at room temperature followed by dropwise addition of triisopropylsilyl chloride (TIPSCl) (2.5 ml, 12 mmol, 1.2 equiv.) via syringe. The mixture was stirred at the same temperature for additional 24 h. After completion (judged by TLC), the reaction mixture was diluted with cold water (30 mL) and extracted with ethyl acetate (30 mL x 3). The combined organic layer washed with cold water (30 mL x 3), brine (50 mL) and dried

over anhydrous Na_2SO_4, filtered and concentrated under reduced pressure. The resulting mixture was purified by silica gel chromatography (2% ethyl acetate in hexane as eluent) gave 2.6 g (94%) 4-((triisopropylsilyl)oxy)benzonitrile **(6k)** as a colourless liquid. Spectral data are in accordance with the reported data.[21]

Synthesis of triisopropyl(4-(trifluoromethyl)phenoxy)silane **(6l)***:*

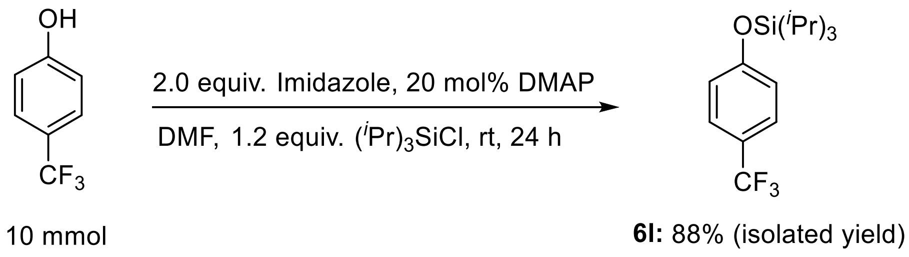

In a dry 100 mL round-bottomed flask 4-(trifluoromethyl)phenol (1.62 g, 10 mmol), imidazole (1.36 g, 20 mmol, 2.0 equiv.), 4-Dimethylaminopyridine (DMAP) (244 mg, 2 mmol, 20 mol%) and 20 mL dry DMF was added. Then stirred for 5 minutes at room temperature followed by dropwise addition of triisopropylsilyl chloride (TIPSCl) (2.5 ml, 12 mmol, 1.2 equiv.) via syringe. The mixture was stirred at the same temperature for additional 24 h. After completion (judged by TLC), the reaction mixture was diluted with cold water (30 mL) and extracted with ethyl acetate (30 mL x 3). The combined organic layer washed with cold water (30 mL x 3), brine (50 mL) and dried over anhydrous Na_2SO_4, filtered and concentrated under reduced pressure. The resulting mixture was purified by silica gel chromatography (2% ethyl acetate in hexane as eluent) gave 2.8 g (88%) triisopropyl(4-(trifluoromethyl)phenoxy)silane **(6l)** as a colourless liquid.

^{1}H NMR (400 MHz, $CDCl_3$): δ 7.54 – 7.51 (m, 2H), 7.01 – 6.97 (m, 2H), 1.37 – 1.29 (m, 3H), 1.18 – 1.14 (m, 18H).

^{13}C NMR (100 MHz, $CDCl_3$): δ 159.2, 127.0 (q, J = 3.8 Hz), 124.8 (q, J = 269.9 Hz), 123.5 (q, J = 32.5 Hz), 120.1, 18.0, 12.9.

HRMS (ESI) *m/z* calcd for $C_{16}H_{25}F_3OSi$ $[M+H]^+$ 319.1705, found 319.1711.

Synthesis of (4-chlorophenoxy)triisopropylsilane **(6m)***:*

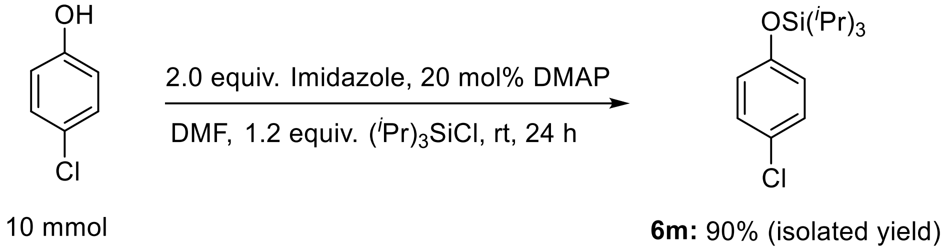

In a dry 100 mL round-bottomed flask 4-chlorophenol (1.28 g, 10 mmol), imidazole (1.36 g, 20 mmol, 2.0 equiv.), 4-Dimethylaminopyridine (DMAP) (244 mg, 2 mmol, 20 mol%) and 20 mL dry DMF was added. Then stirred for 5 minutes at room temperature followed by dropwise addition of triisopropylsilyl chloride (TIPSCl) (2.5 ml, 12 mmol, 1.2 equiv.) via syringe. The mixture was stirred at the same temperature for additional 24 h. After completion (judged by TLC), the reaction mixture was diluted with cold water (30 mL) and extracted with ethyl acetate (30 mL x 3). The combined organic layer washed with cold water (30 mL x 3), brine (50 mL) and dried over anhydrous Na_2SO_4, filtered and concentrated under reduced pressure. The resulting mixture was purified by silica gel chromatography (2% ethyl acetate in hexane as eluent) gave 2.5 g (90%) (4-chlorophenoxy)triisopropylsilane **(7m)** as a colourless liquid. Spectral data are in accordance with the reported data.[31]

Synthesis of (4-bromophenoxy)triisopropylsilane **(6n)***:*

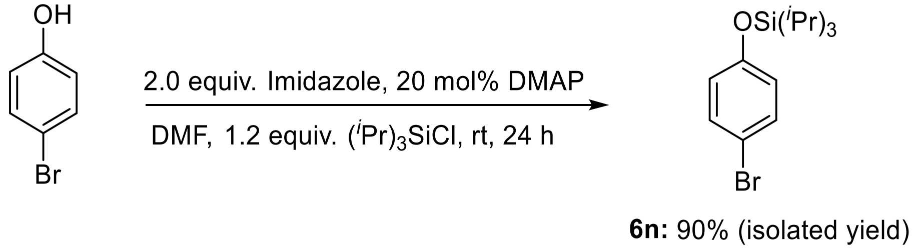

In a dry 100 mL round-bottomed flask 4-bromophenol (1.71 g, 10 mmol), imidazole (1.36 g, 20 mmol, 2.0 equiv.), 4-Dimethylaminopyridine (DMAP) (244 mg, 2 mmol, 20 mol%) and 20 mL dry DMF was added. Then stirred for 5 minutes at room temperature followed by dropwise addition of triisopropylsilyl chloride (TIPSCl) (2.5 ml, 12 mmol, 1.2 equiv.) via syringe. The mixture was stirred at the same temperature for additional 24 h. After completion (judged by TLC), the reaction mixture was diluted with cold water (30 mL) and extracted with ethyl acetate (30 mL x 3). The combined organic layer washed with cold water (30 mL x 3), brine (50 mL) and dried

over anhydrous Na_2SO_4, filtered and concentrated under reduced pressure. The resulting mixture was purified by silica gel chromatography (2% ethyl acetate in hexane as eluent) gave 3.0 g (91%) (4-bromophenoxy)triisopropylsilane **(6n)** as a colourless liquid. Spectral data are in accordance with the reported data.[32]

Synthesis of triisopropyl(4-(1-methoxyethyl)phenoxy)silane **(6o)***:*

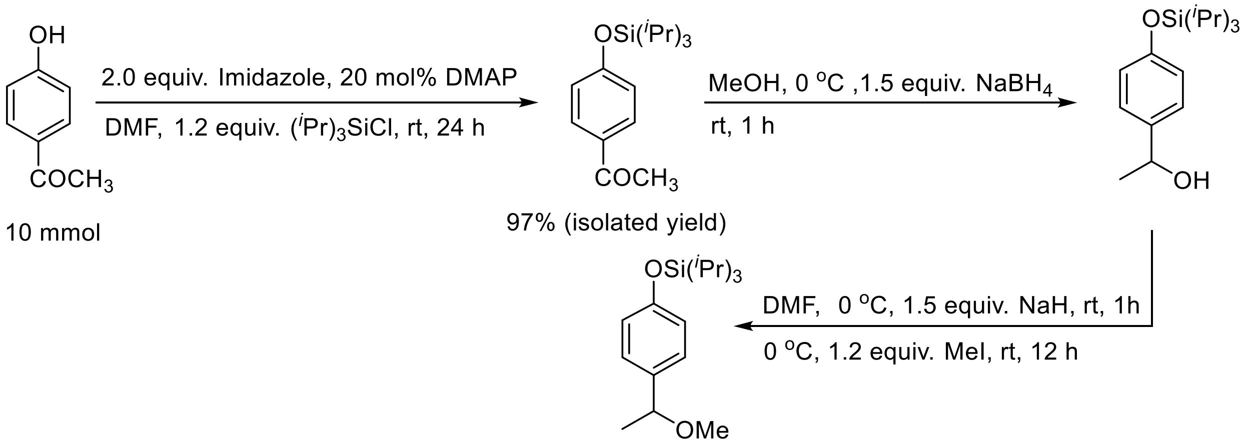

6o: 75% (isolated yield)

Step I: In a dry 100 mL round-bottomed flask 1-(4-hydroxyphenyl)ethan-1-one (1.36g, 10 mmol), imidazole (1.36 g, 20 mmol, 2.0 equiv.), 4-Dimethylaminopyridine (DMAP) (244 mg, 2 mmol, 20 mol%) and 20 mL dry DMF was added. Then stirred for 5 minutes at room temperature followed by dropwise addition of triisopropylsilyl chloride (TIPSCl) (2.5 ml, 12 mmol, 1.2 equiv.) via syringe. The mixture was stirred at the same temperature for additional 24 h. After completion (judged by TLC), the reaction mixture was diluted with cold water (30 mL) and extracted with ethyl acetate (30 mL x 3). The combined organic layer washed with cold water (30 mL x 3), brine (50 mL) and dried over anhydrous Na_2SO_4, filtered and concentrated under reduced pressure. The resulting mixture was purified by silica gel chromatography (7% ethyl acetate in hexane as eluent) gave 2.8 g (97%) 1-(4-((triisopropylsilyl)oxy)phenyl)ethan-1-one as a colourless liquid. Spectral data are in accordance with the reported data.[33]

Step II: An oven dried 100 mL round-bottom flask was charged with 1-(4-((triisopropylsilyl)oxy)phenyl)ethan-1-one (2.8 g, 9.7 mmol), MeOH (20.0 mL) and cool to 0 ^oC. solid sodium borohydride (570 mg, 1.5 equiv.) was added portionwise over 30 minutes and stirred for another 1 h at room temperature. After completion (monitored by TLC), MeOH was evaporated under reduced pressure, diluted with water (20 mL) and extracted with ethyl acetate (30 mL x 3).

The combined organic layer was washed with brine (50 mL), dried over anhydrous Na_2SO_4 , filterd and concentrated under reduced pressure to get the 1-(4-((triisopropylsilyl)oxy)phenyl)ethan-1-ol as a gummy liquid which was used directly for the next step without furthur purification.

Step III: In a 100 mL round-bottom flask crude 1-(4-((triisopropylsilyl)oxy)phenyl)ethan-1-ol was dissolved in DMF (20 mL), cool to 0 ^oC and then NaH (630 mg, 1.5 equiv. 50 % dispersion in mineral oil) was added portion wise at 0 ^oC and stirred for 1 h at room temperature. Then MeI (680 µL, 1.2 equiv.) was added at 0 ^oC and stirred it for 12 h at room temperature. After completion (judged by TLC), the reaction mixture was diluted with cold water (30 mL) and extracted with ethyl acetate (30 mL x 3). The combined organic layer washed with cold water (30 mL x 3), brine (50 mL) and dried over anhydrous Na_2SO_4, filtered and concentrated under reduced pressure. . The resulting mixture was purified by silica gel chromatography (2% ethyl acetate in hexane as eluent) gave 2.2 g (75%) triisopropyl(4-(1-methoxyethyl)phenoxy)silane **(6o)** as a colourless liquid.

^{1}H NMR (400 MHz, $CDCl_3$): δ 7.15 (d, J = 8.4 Hz, 2H), 6.86 (d, J = 8.4 Hz, 2H), 4.23 (q, J = 6.4 Hz, 1H), 3.18 (s, 3H), 1.42 (d, J = 6.4 Hz, 3H), 1.28 – 1.23 (m, 3H), 1.11 (d, J = 7.2 Hz, 18H).
^{13}C NMR (100 MHz, $CDCl_3$): δ 155.5, 135.9, 127.4, 119.9, 79.3, 56.2, 23.8, 18.0, 12.8.
HRMS (ESI) *m/z* calcd for $C_{18}H_{32}O_2Si$ $[M+Na]^+$ 331.2069, found 331.2069.

Synthesis of triisopropyl(4-(methoxymethyl)phenoxy)silane **(6p)***:*

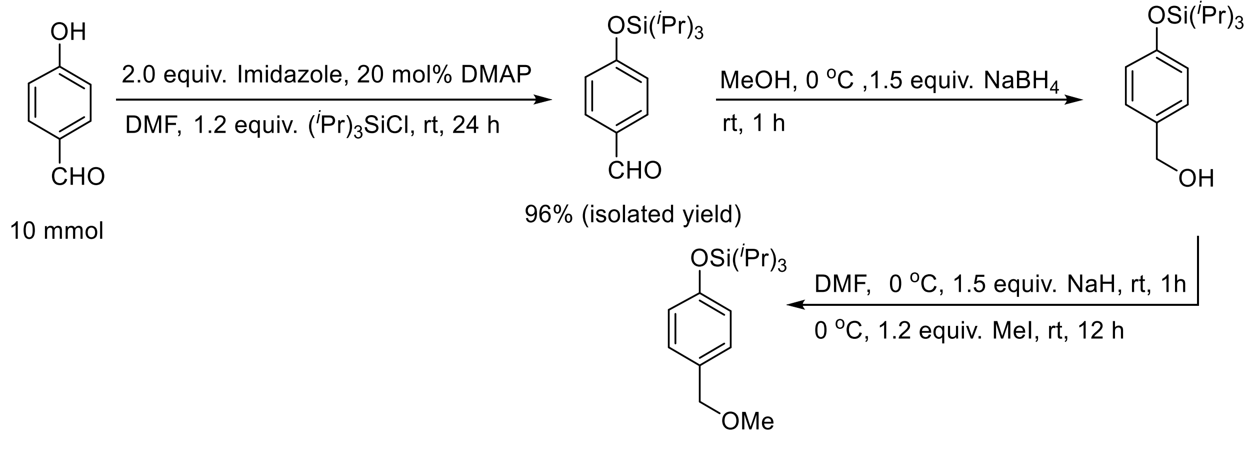

6p: 88% (isolated yield)

Step I: In a dry 100 mL round-bottomed flask 4-hydroxybenzaldehyde (1.22 g, 10 mmol), imidazole (1.36 g, 20 mmol, 2.0 equiv.), 4-Dimethylaminopyridine (DMAP) (244 mg, 2 mmol, 20 mol%) and 20 mL dry DMF was added. Then stirred for 5 minutes at room temperature followed

by dropwise addition of triisopropylsilyl chloride (TIPSCl) (2.5 ml, 12 mmol, 1.2 equiv.) via syringe. The mixture was stirred at the same temperature for additional 24 h. After completion (judged by TLC), the reaction mixture was diluted with cold water (30 mL) and extracted with ethyl acetate (30 mL x 3). The combined organic layer washed with cold water (30 mL x 3), brine (50 mL) and dried over anhydrous Na_2SO_4, filtered and concentrated under reduced pressure. The resulting mixture was purified by silica gel chromatography (5% ethyl acetate in hexane as eluent) gave 2.7 g (96%) 4-((triisopropylsilyl)oxy)benzaldehyde as a colourless liquid. Spectral data are in accordance with the reported data[34].

Step II: An oven dried 100 mL round-bottomed flask was charged with 4-((triisopropylsilyl)oxy)benzaldehyde (2.66 g, 9.7 mmol), MeOH (20.0 mL) and cool to 0 ^oC. Solid sodium borohydride (570 mg, 1.5 equiv.) was added portionwise over 30 minutes and stirred for another 1 h at room temperature. After completion (monitored by TLC), MeOH was evaporated under reduced pressure, diluted with water (20 mL) and extracted with ethyl acetate (30 mL x 3). The combined organic layer was washed with brine (50 mL), dried over anhydrous Na_2SO_4 , filterd and concentrated under reduced pressure to get the (4-((triisopropylsilyl)oxy)phenyl)methanol as a gummy liquid which was used directly for the next step without furthur purification.

Step III: In a 100 mL round-bottom flask crude (4-((triisopropylsilyl)oxy)phenyl)methanol was dissolved in DMF (20 mL), cool to 0 ^oC and then NaH (630 mg, 1.5 equiv. 50 % dispersion in mineral oil) was added portion wise at 0 ^oC and stirred for 1 h at room temperature. Then MeI (680 µL, 1.2 equiv.) was added at 0 ^oC and stirred it for 12 h at room temperature. After completion (judged by TLC), the reaction mixture was diluted with cold water (30 mL) and extracted with ethyl acetate (30 mL x 3). The combined organic layer washed with cold water (30 mL x 3), brine (50 mL) and dried over anhydrous Na_2SO_4, filtered and concentrated under reduced pressure. . The resulting mixture was purified by silica gel chromatography (2% ethyl acetate in hexane as eluent) gave 2.4 g (88%) triisopropyl(4-(methoxymethyl)phenoxy)silane **(6p)** as a colourless liquid.

^{1}H NMR (400 MHz, $CDCl_3$): δ 7.19 (d, *J* = 8.4 Hz, 2H), 6.86 (d, *J* = 8.4 Hz, 2H), 4.38 (s, 2H), 3.36 (s, 3H), 1.28 – 1.23 (m, 3H), 1.11 (d, *J* = 7.6 Hz, 18H).

^{13}C NMR (100 MHz, $CDCl_3$): δ 155.8, 130.7, 129.4, 119.9, 74.6, 57.9, 18.0, 12.8.

HRMS (ESI) *m/z* calcd for $C_{17}H_{30}O_2Si$ $[M+Na]^+$ 317.1913, found 317.1917.

Synthesis of triisopropyl(4-(propoxymethyl)phenoxy)silane **(6q)**:

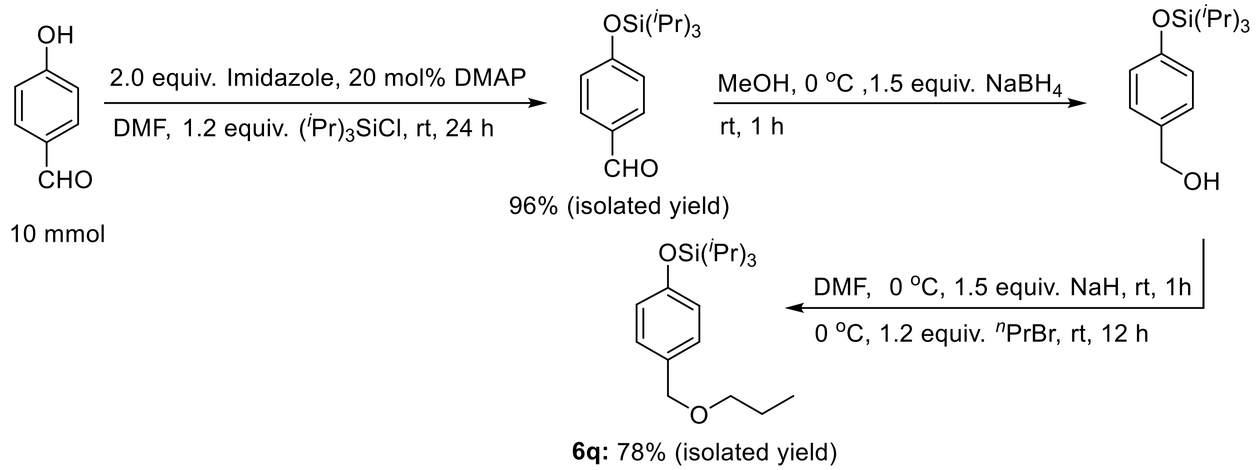

Step I: In a dry 100 mL round-bottomed flask 4-hydroxybenzaldehyde (1.22 g, 10 mmol), imidazole (1.36 g, 20 mmol, 2.0 equiv.), 4-Dimethylaminopyridine (DMAP) (244 mg, 2 mmol, 20 mol%) and 20 mL dry DMF was added. Then stirred for 5 minutes at room temperature followed by dropwise addition of triisopropylsilyl chloride (TIPSCl) (2.5 ml, 12 mmol, 1.2 equiv.) via syringe. The mixture was stirred at the same temperature for additional 24 h. After completion (judged by TLC), the reaction mixture was diluted with cold water (30 mL) and extracted with ethyl acetate (30 mL x 3). The combined organic layer washed with cold water (30 mL x 3), brine (50 mL) and dried over anhydrous Na_2SO_4, filtered and concentrated under reduced pressure. The resulting mixture was purified by silica gel chromatography (2% ethyl acetate in hexane as eluent) gave 2.7 g (96%) 4-((triisopropylsilyl)oxy)benzaldehyde as a colourless liquid. Spectral data are in accordance with the reported data.[34]

Step II: An oven dried 100 mL round-bottomed flask was charged with 4-((triisopropylsilyl)oxy)benzaldehyde (2.66 g, 9.7 mmol), MeOH (20.0 mL) and cool to 0 °C. Solid sodium borohydride (570 mg, 1.5 equiv.) was added portionwise over 30 minutes and stirred for another 1 h at room temperature. After completion (monitored by TLC), MeOH was evaporated under reduced pressure, diluted with water (20 mL) and extracted with ethyl acetate (30 mL x 3). The combined organic layer was washed with brine (50 mL), dried over anhydrous Na_2SO_4 , filterd and concentrated under reduced pressure to get the (4-((triisopropylsilyl)oxy)phenyl)methanol as a gummy liquid which was used directly for the next step without furthur purification.

Step III: In a 100 mL round-bottom flask crude (4-((triisopropylsilyl)oxy)phenyl)methanol was dissolved in DMF (20 mL), cool to 0 ^{o}C and then NaH (630 mg, 1.5 equiv. 50 % dispersion in mineral oil) was added portion wise at 0 ^{o}C and stirred for 1 h at room temperature. Then 1-bromopropane (883 µL, 1.2 equiv.) was added at 0 ^{o}C and stirred it for 12 h at room temperature. After completion (judged by TLC), the reaction mixture was diluted with cold water (30 mL) and extracted with ethyl acetate (30 mL x 3). The combined organic layer washed with cold water (30 mL x 3), brine (50 mL) and dried over anhydrous Na_2SO_4, filtered and concentrated under reduced pressure. . The resulting mixture was purified by silica gel chromatography (2% ethyl acetate in hexane as eluent) gave 2.4 g (78%) triisopropyl(4-(propoxymethyl)phenoxy)silane **(6q)** as a colourless liquid.

Synthesis of (4-cyclohexylphenoxy)triisopropylsilane **(6r)***:*

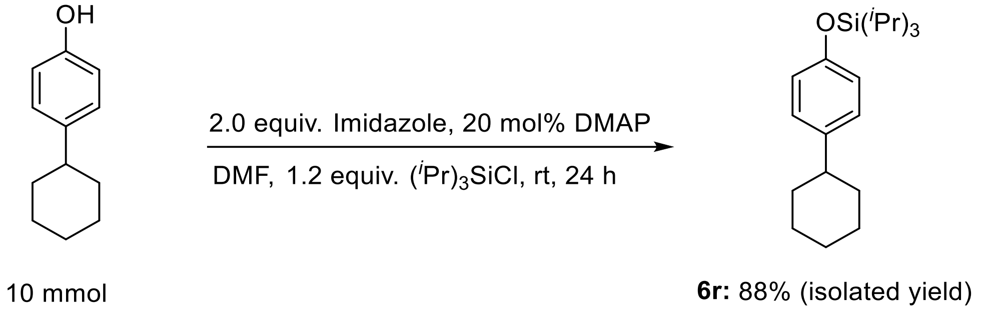

In a dry 100 mL round-bottomed flask 4-cyclohexylphenol (1.76 g, 10 mmol), imidazole (1.36 g, 20 mmol, 2.0 equiv.), 4-Dimethylaminopyridine (DMAP) (244 mg, 2 mmol, 20 mol%) and 20 mL dry DMF was added. Then stirred for 5 minutes at room temperature followed by dropwise addition of triisopropylsilyl chloride (TIPSCl) (2.5 ml, 12 mmol, 1.2 equiv.) via syringe. The mixture was stirred at the same temperature for additional 24 h. After completion (judged by TLC), the reaction mixture was diluted with cold water (30 mL) and extracted with ethyl acetate (30 mL x 3). The combined organic layer washed with cold water (30 mL x 3), brine (50 mL) and dried over anhydrous Na_2SO_4, filtered and concentrated under reduced pressure. The resulting mixture was purified by silica gel chromatography (1% ethyl acetate in hexane as eluent) gave 2.9 g (88%) (4-cyclohexylphenoxy)triisopropylsilane **(6r)** as a colourless liquid.

^{1}H NMR (400 MHz, $CDCl_3$): δ 7.07 (d, J = 8.4 Hz, 2H), 6.83 (d, J = 8.8 Hz, 2H), 2.49 – 2.43 (m, 1H), 1.90 – 1.85 (m, 4H), 1.77 (d, J = 12.4 Hz, 1H), 1.46 – 1.35 (m, 4H), 1.33 – 1.24 (m, 4H), 1.14 (d, J = 7.2 Hz, 18H).

^{13}C NMR (100 MHz, $CDCl_3$): δ 154.0, 140.7, 127.6, 119.6, 43.9, 34.9, 27.1, 26.4, 18.1, 12.8. HRMS (ESI) *m/z* calcd for $C_{21}H_{36}OSi$ $[M+H]^+$ 333.2614, found 333.2609.

Synthesis of **8a:**

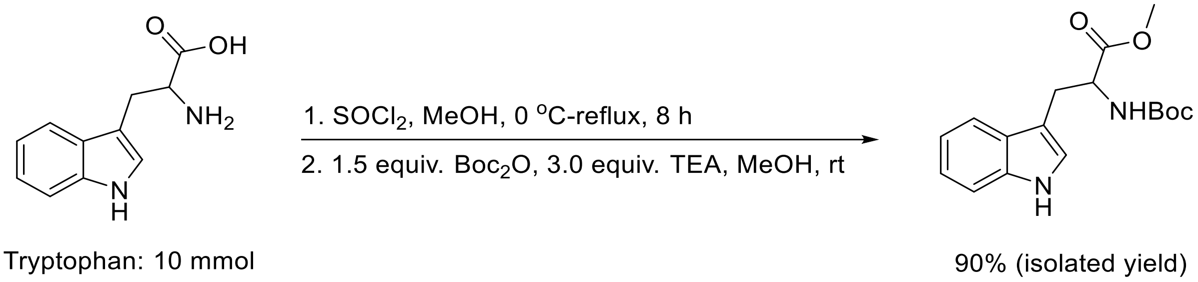

Step-I:

An oven dried 10 mL round bottom flask was charged with tryptophan (2.04 g, 10.0 mmol), and MeOH (20.0 mL). Then the reaction mixture was placed in an ice bath and $SOCl_2$ (5.0 mL) was added at 0 ^oC. After that the reaction mixture was refluxed for 8 h. After completion solvents and volatiles are removed via rotary evaporators and dried under vacuum to gave solid ester of tryptophane. The crude solid was for the next step without further purifications.

Step-II:

An oven dried 100 mL round bottom flask was charged with crude solid from the previous step and MeOH (20.0 mL). Then in the reaction mixture was added triethyl amine (4.2 mL, 3.0 equiv.) and stirred for the 15 minutes at the room temperature. After that Boc_2O (3.3 g, 3.5 mL, 1.5 equiv.) was added dropwise into the reaction mixture and stirred for 12 h at the room temperature. After completion (judged by TLC), the reaction mixture was diluted with cold water (30 mL) and extracted with ethyl acetate (30 mL x 3). The combined organic layer washed with cold water (30 mL x 3), brine (50 mL) and dried over anhydrous Na_2SO_4, filtered and concentrated under reduced pressure. The resulting mixture was purified by silica gel chromatography (10% ethyl acetate in hexane as eluent) gave 2.86 g (90%) methyl (tert-butoxycarbonyl)tryptophanate (5f) as a white solid. The spectral data are in accordance with the reported data.[35]

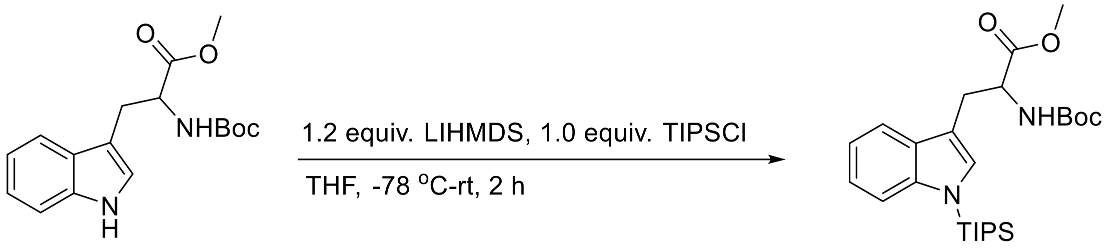

7.86 mmol

8a: 85% (isolated yield)

An oven dried 100 mL round bottom flask was charged with methyl (tert-butoxycarbonyl)tryptophanate (2.5 g, 7.86 mmol) and anhydrous THF (20.0 mL). The reaction mixture was then taken down to -78 °C and LiHMDS (1M in THF, 9.9 mL, 9.9 mmol, 1.26 equiv.) was added. After that it was stirred for 1 h at -78 °C and TIPSCl (1.7 mL, 1.0 equiv.) was added to it. Then the reaction mixture was warmed to room temperature and stirred at room temperature for 1 h. After completion (judged by TLC), the reaction mixture was diluted with cold water (30 mL) and extracted with ethyl acetate (30 mL x 3). The combined organic layer washed with cold water (30 mL x 3), brine (50 mL) and dried over anhydrous Na_2SO_4, filtered and concentrated under reduced pressure. The resulting mixture was purified by silica gel chromatography (5% ethyl acetate in hexane as eluent) gave 3.16 g (85%) **8a** as a yellow gummy liquid. The spectral data are in accordance with the reported data.[35]

Synthesis of **8b:**

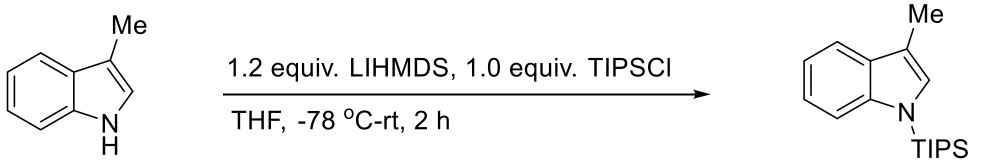

10 mmol

8b: 80% (isolated yield)

An oven dried 100 mL round bottom flask was charged with 3-methyl indole (1.31 g, 10.0 mmol) and anhydrous THF (20.0 mL). The reaction mixture was then taken down to -78 °C and LiHMDS (1M in THF, 12.6 mL, 12.6 mmol, 1.26 equiv.) was added. After that it was stirred for 1 h at -78 °C and TIPSCl (2.16 mL, 1.0 equiv.) was added to it. Then the reaction mixture was warmed to room temperature and stirred at room temperature for 1 h. After completion (judged by TLC), the reaction mixture was diluted with cold water (30 mL) and extracted with ethyl acetate (30 mL x 3). The combined organic layer washed with cold water (30 mL x 3), brine (50 mL) and dried over anhydrous Na_2SO_4, filtered and concentrated under reduced pressure. The resulting mixture was

purified by silica gel chromatography (5% ethyl acetate in hexane as eluent) gave 2.3 g (80%) **8b** as a yellow gummy liquid. The spectral data are in accordance with the reported data.[35]

Synthesis of **8c:**

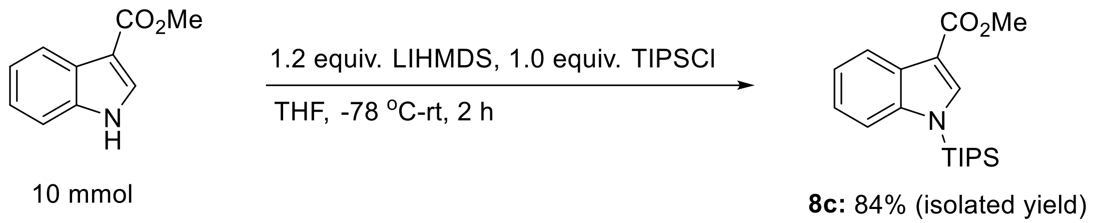

An oven dried 100 mL round bottom flask was charged with methyl 1H-indole-3-carboxylate (1.75 g, 10.0 mmol) and anhydrous THF (20.0 mL). The reaction mixture was then taken down to -78 °C and LiHMDS (1M in THF, 12.6 mL, 12.6 mmol, 1.26 equiv.) was added. After that it was stirred for 1 h at -78 °C and TIPSCl (2.16 mL, 1.0 equiv.) was added to it. Then the reaction mixture was warmed to room temperature and stirred at room temperature for 1 h. After completion (judged by TLC), the reaction mixture was diluted with cold water (30 mL) and extracted with ethyl acetate (30 mL x 3). The combined organic layer washed with cold water (30 mL x 3), brine (50 mL) and dried over anhydrous Na_2SO_4, filtered and concentrated under reduced pressure. The resulting mixture was purified by silica gel chromatography (5% ethyl acetate in hexane as eluent) gave 2.78 g (84%) **8c** as a yellow gummy liquid. The spectral data are in accordance with the reported data.[35]

Synthesis of **8d**:

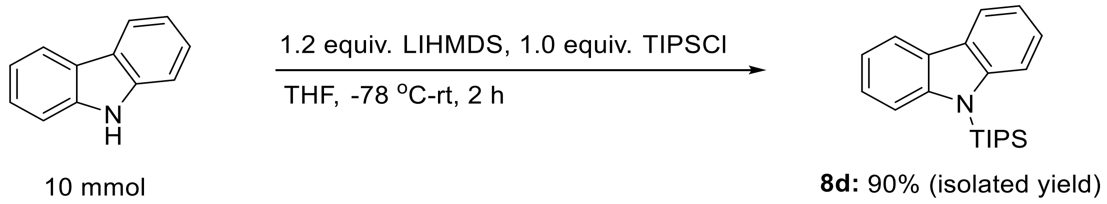

An oven dried 100 mL round bottom flask was charged with carbazole (1.67 g, 10.0 mmol) and anhydrous THF (20.0 mL). The reaction mixture was then taken down to -78 °C and LiHMDS (1M in THF, 12.6 mL, 12.6 mmol, 1.26 equiv.) was added. After that it was stirred for 1 h at -78 °C and TIPSCl (2.16 mL, 1.0 equiv.) was added to it. Then the reaction mixture was warmed to room temperature and stirred at room temperature for 1 h. After completion (judged by TLC), the reaction mixture was diluted with cold water (30 mL) and extracted with ethyl acetate (30 mL x 3). The combined organic layer washed with cold water (30 mL x 3), brine (50 mL) and dried over

anhydrous Na_2SO_4, filtered and concentrated under reduced pressure. The resulting mixture was purified by silica gel chromatography (5% ethyl acetate in hexane as eluent) gave 2.9 g (90%) **8d** as a yellow gummy liquid. The spectral data are in accordance with the reported data.[35]

Synthesis of ((6H-benzo[c]chromen-1-yl)oxy)triisopropylsilane **(10a)***:*

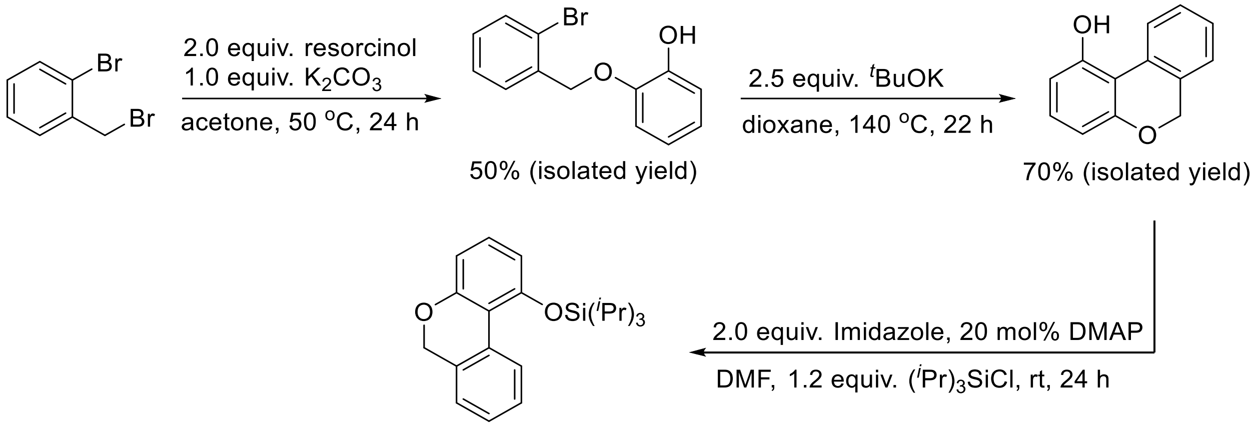

10a: 91% (isolated yield)

Step I: An oven dried 100 mL round bottom flask was charged with resorcinol (4.95 g, 30.0 mmol), K_2CO_3 (2.07 g, 1.0 equiv.) and acetone (75.0 mL). This mixture was stirred at room temperature for 30 minutes. 2-Bromobenzyl bromide (3.75 g, 15.0 mmol) was added to the mixture and placed into preheated oil bath at 50 ^{o}C for 23 h 30 minutes. Upon total consumption of 2-bromobenzyl bromide (checked by TLC), the reaction mixture was cooled to room temperature, poured into aqueous 2N NaOH (75 mL) and extracted with ethyl acetate (50 mL x 3). The combined organic layer was washed with brine (40 mL) and dried over anhydrous Na_2SO_4. The organic extract was concentrated under reduced pressure and chromatographic separation with silica gel (10% ethyl acetate in hexane as eluent) gave 2.09 g (50%) of 3-((2-bromobenzyl)oxy)phenol as white solid. Spectral data are in accordance with the reported data.[36]

Step II: In an argon-filled glove box, an oven dried 15 mL pressure tube was charged with 3-((2-bromobenzyl)oxy)phenol (2.09g, 7.5 mmol), ^{t}BuOK (2.1 g, 2.5 equiv.), sealed with a rubber septum and taken out from the glove box. Dioxane (10.0 mL) was added to the mixture, capped with a teflon pressure cap and stirred at room temperature for 5 minutes. The pressure tube was placed in a preheated silicon oil bath at 140 °C and stirred for 22 h. After cooling to room temperature, the reaction mixture was quenched with 2N HCl (50 mL) and extracted with ethyl acetate (30 mL x 3). The combined organic layer was washed with brine (40 mL) and dried over

anhydrous Na_2SO_4. The organic extract was concentrated under reduced pressure and chromatographic separation with silica gel (40% dichloromethane in hexane as eluent) gave 1.05 g (70%) of 6H-benzo[c]chromen-1-ol as white solid. Spectral data are in accordance with the reported data.[36]

Step III: In a dry 100 mL round-bottomed flask 6H-benzo[c]chromen-1-ol (960 mg, 5 mmol), imidazole (680 mg, 10 mmol, 2.0 equiv.), 4-Dimethylaminopyridine (DMAP) (122 mg, 1 mmol, 20 mol%) and 20 mL dry DMF was added. Then stirred for 5 minutes at room temperature followed by dropwise addition of triisopropylsilyl chloride (TIPSCl) (1.25 mL, 6 mmol, 1.2 equiv.) via syringe. The mixture was stirred at the same temperature for additional 24 h. After completion (judged by TLC), the reaction mixture was diluted with cold water (30 mL) and extracted with ethyl acetate (30 mL x 3). The combined organic layer washed with cold water (30 mL x 3), brine (50 mL) and dried over anhydrous Na_2SO_4, filtered and concentrated under reduced pressure. The resulting mixture was purified by silica gel chromatography (2% ethyl acetate in hexane as eluent) gave 1.6 g (91%) ((6H-benzo[c]chromen-1-yl)oxy)triisopropylsilane **(10a)** as a colourless liquid.

^{1}H NMR (400 MHz, $CDCl_3$): δ 8.32 (d, J = 8.0 Hz, 1H), 7.22 (t, J = 7.6 Hz, 1H), 7.14 (t, J = 7.6 Hz, 1H), 7.05 (d, J = 7.2 Hz, 1H), 6.94 (t, J = 8. Hz, 1H), 6.56 (d, J = 8.0 Hz, 1H), 6.50 (d, J = 8.0 Hz, 1H), 4.87 (s, 2H), 1.28 – 1.21 (m, 3H), 1.01 (d, J = 7.6 Hz, 18H).
^{13}C NMR (100 MHz, $CDCl_3$): δ 157.1, 154.2, 132.1, 129.4, 128.6, 127.7, 126.9, 126.9, 124.3, 115.2, 113.5, 110.2, 69.0, 18.1, 13.4.
HRMS (ESI) *m/z* calcd for $C_{22}H_{30}O_2Si$ $[M+Na]^+$ 377.1913, found 377.1920.

Synthesis of methyl 2-((triisopropylsilyl)oxy)benzoate **(10b)***:*

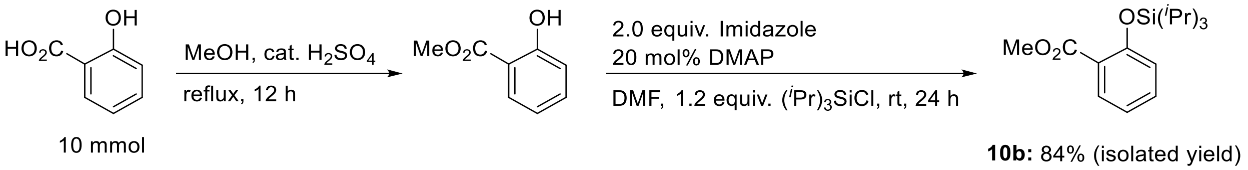

Step I: A 35 mL pressure tube was charged with a mixture of 2-hydroxybenzoic acid (1.38 g, 10 mmol), MeOH (15 mL) and catalytic amount of H_2SO_4. Then it was refluxed for 12 h with vigorous stirring. After that, the reaction mixture was cooled to room temperature and neutralize with aqueous Na_2CO_3. The whole mixture was then extracted with dichloromethane (30 mL x 3) and washed with water. The combined organic layer dried over anhydrous Na_2SO_4, filtered and

concentrated under reduced pressure to afford the methyl 2-hydroxybenzoate quantitatively which was directly used for the next step without further purifications.

Step II: In a dry 100 mL round-bottomed flask crude methyl 2-hydroxybenzoate, imidazole (1.36 g, 20 mmol, 2.0 equiv.), 4-Dimethylaminopyridine (DMAP) (244 mg, 2 mmol, 20 mol%) and 20 mL dry DMF was added. Then stirred for 5 minutes at room temperature followed by dropwise addition of triisopropylsilyl chloride (TIPSCl) (2.5 ml, 12 mmol, 1.2 equiv.) via syringe. The mixture was stirred at the same temperature for additional 24 h. After completion (judged by TLC), the reaction mixture was diluted with cold water (30 mL) and extracted with ethyl acetate (30 mL x 3). The combined organic layer washed with cold water (30 mL x 3), brine (50 mL) and dried over anhydrous Na_2SO_4, filtered and concentrated under reduced pressure. The resulting mixture was purified by silica gel chromatography (3% ethyl acetate in hexane as eluent) gave 2.6 g (84%) methyl 2-((triisopropylsilyl)oxy)benzoate **(10b)** as a colourless liquid.

^{1}H NMR (400 MHz, $CDCl_3$): δ 7.73 (dd, *J* = 8.0, 2.0 Hz, 1H), 7.32 (dt, *J* = 8.0, 1.6 Hz, 1H), 6.94 (t, *J* = 7.2 Hz, 1H), 6.87 (d, *J* = 8.4 Hz, 1H), 3.85 (s, 3H), 1.35 – 1.27 (m, 3H), 1.11 (d, *J* = 7.6 Hz, 18H).
^{13}C NMR (100 MHz, $CDCl_3$): δ 167.6, 155.6, 132.9, 131.6, 122.7, 120.5, 120.4, 51.9, 18.0, 13.2.
HRMS (ESI) *m/z* calcd for $C_{17}H_{28}O_3Si$ $[M+H]^+$ 309.1886, found 309.1886.

Synthesis of triisopropyl(4-(2-methoxyethyl)phenoxy)silane **(10c)***:*

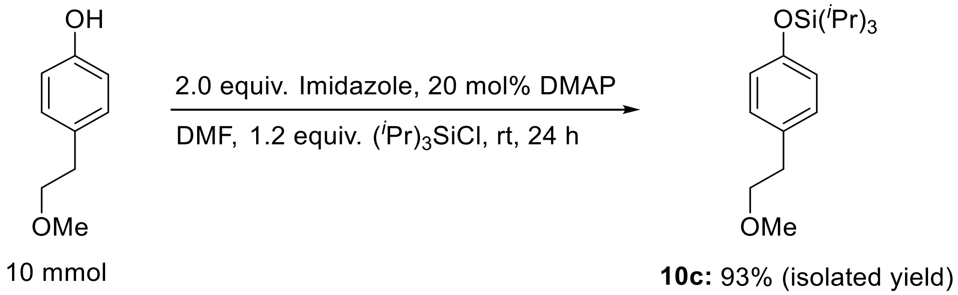

In a dry 100 mL round-bottomed flask 4-(2-methoxyethyl)phenol (1.52 g, 10 mmol), imidazole (1.36 g, 20 mmol, 2.0 equiv.), 4-Dimethylaminopyridine (DMAP) (244 mg, 2 mmol, 20 mol%) and 20 mL dry DMF was added. Then stirred for 5 minutes at room temperature followed by dropwise addition of triisopropylsilyl chloride (TIPSCl) (2.5 ml, 12 mmol, 1.2 equiv.) via syringe. The mixture was stirred at the same temperature for additional 24 h. After completion (judged by TLC), the reaction mixture was diluted with cold water (30 mL) and extracted with ethyl acetate

(30 mL x 3). The combined organic layer washed with cold water (30 mL x 3), brine (50 mL) and dried over anhydrous Na_2SO_4, filtered and concentrated under reduced pressure. The resulting mixture was purified by silica gel chromatography (1% ethyl acetate in hexane as eluent) gave 3.0 g (93%) triisopropyl(4-(2-methoxyethyl)phenoxy)silane **(10c)** as a colourless liquid.

^{1}H NMR (400 MHz, $CDCl_3$): δ 7.07 (d, *J* = 8.4 Hz, 2H), 6.82 (d, *J* = 8.4 Hz, 2H), 3.57 (t, *J* = 7.2 Hz, 2H), 3.36 (s, 3H), 2.83 (t, *J* = 7.2 Hz, 2H), 1.31 – 1.23 (m, 3H), 1.12 (d, *J* = 7.2 Hz, 18H).
^{13}C NMR (100 MHz, $CDCl_3$): δ 154.5, 131.2, 129.7, 119.8, 74.1, 58.7, 35.5, 18.0, 12.8.
HRMS (ESI) *m/z* calcd for $C_{18}H_{32}O_2Si$ $[M+H]^+$ 309.2250, found 309.2248.

Synthesis of triisopropyl(2-methoxy-4-propylphenoxy)silane **(10d)***:*

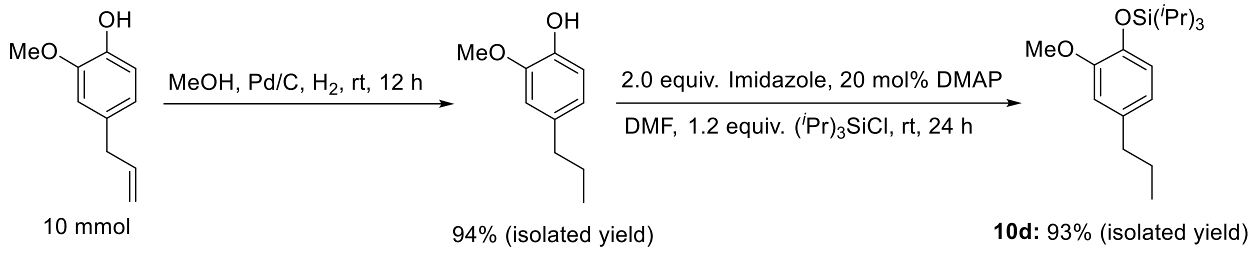

Step I: An oven dried 100 mL round-bottomed flask containing 4-allyl-2-methoxyphenol (1.64 g, 10 mmol) in MeOH (10.0 mL), Pd/C (160 mg, 10 wt.%, 1.5 mol%) was added portion wise. The flask was evacuated and backfilled with H_2 three times. The suspension was stirred with a balloon of H_2 gas for 12 h at room temperature. After completion of reaction (checked by TLC), the reaction mixture was filtered through a pad of celite using ethyl acetate and concentrated under reduced pressure to afford the crude product. The crude product was purified column chromatography using silica gel (10% ethyl acetate in hexane as eluent) to afford 1.5 g (93%) of 2-methoxy-4-propylphenol as gummy liquid. Spectral data are in accordance with the reported data.[37]

Step II: In a dry 100 mL round-bottomed flask 2-methoxy-4-propylphenol (1.5 g, 9 mmol), imidazole (1.22 g, 18 mmol, 2.0 equiv.), 4-Dimethylaminopyridine (DMAP) (219 mg, 1.8 mmol, 20 mol%) and 20 mL dry DMF was added. Then stirred for 5 minutes at room temperature followed by dropwise addition of triisopropylsilyl chloride (TIPSCl) (2.25 ml, 10.8 mmol, 1.2 equiv.) via syringe. The mixture was stirred at the same temperature for additional 24 h. After completion (judged by TLC), the reaction mixture was diluted with cold water (30 mL) and extracted with ethyl acetate (30 mL x 3). The combined organic layer washed with cold water (30

mL x 3), brine (50 mL) and dried over anhydrous Na_2SO_4, filtered and concentrated under reduced pressure. The resulting mixture was purified by silica gel chromatography (2% ethyl acetate in hexane as eluent) gave 2.7 g (93%) triisopropyl(2-methoxy-4-propylphenoxy)silane **(10d)** as a colourless liquid.

^{1}H NMR (400 MHz, $CDCl_3$): δ 6.81 (d, *J* = 7.6 Hz, 1H), 6.69 (d, *J* = 0.8 Hz, 1H), 6.63 (d, *J* = 7.6 Hz, 1H), 3.81 (s, 3H), 2.54 (t, *J* = 8.0 Hz, 2H), 1.69 – 1.60 (m, 2H), 1.33 – 1.24 (m, 3H), 1.13 (d, *J* = 7.6 Hz, 18H), 0.95 (t, *J* = 7.6 Hz, 3H).
^{13}C NMR (100 MHz, $CDCl_3$): δ 150.6, 143.5, 136.0, 120.5, 120.2, 112.7, 55.5, 37.9, 24.9, 18.1, 13.9, 13.0.
HRMS (ESI) *m/z* calcd for $C_{19}H_{34}O_2Si$ $[M+H]^+$ 323.2406, found 323.2411.

Synthesis of (benzo[d][1,3]dioxol-5-yloxy)triisopropylsilane **(10e)***:*

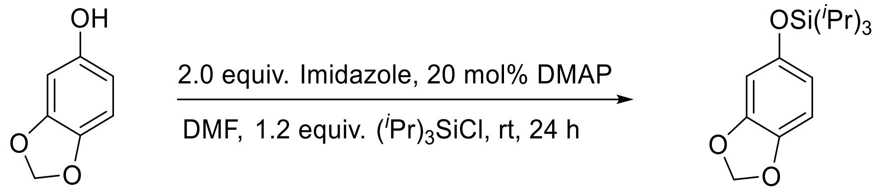

10 mmol

10e: 89% (isolated yield)

In a dry 100 mL round-bottomed flask benzo[d][1,3]dioxol-5-ol (1.38 g, 10 mmol), imidazole (1.36 g, 20 mmol, 2.0 equiv.), 4-Dimethylaminopyridine (DMAP) (244 mg, 2 mmol, 20 mol%) and 20 mL dry DMF was added. Then stirred for 5 minutes at room temperature followed by dropwise addition of triisopropylsilyl chloride (TIPSCl) (2.5 ml, 12 mmol, 1.2 equiv.) via syringe. The mixture was stirred at the same temperature for additional 24 h. After completion (judged by TLC), the reaction mixture was diluted with cold water (30 mL) and extracted with ethyl acetate (30 mL x 3). The combined organic layer washed with cold water (30 mL x 3), brine (50 mL) and dried over anhydrous Na_2SO_4, filtered and concentrated under reduced pressure. The resulting mixture was purified by silica gel chromatography (2% ethyl acetate in hexane as eluent) gave 2.6 g (89%) (benzo[d][1,3]dioxol-5-yloxy)triisopropylsilane **(5e)** as a colourless liquid.

^{1}H NMR (400 MHz, $CDCl_3$): δ 6.65 (d, *J* = 8.4 Hz, 1H), 6.47 (d, *J* = 2.4 Hz, 1H), 6.34 (dd, *J* = 8.4, 2.4 Hz, 1H), 5.90 (s, 2H), 1.30 – 1.22 (m, 3H), 1.12 (d, *J* = 7.6 Hz, 18H).
^{13}C NMR (100 MHz, $CDCl_3$): δ 151.1, 148.1, 141.7, 111.5, 108.0, 102.4, 101.2, 18.0, 12.7.
HRMS (ESI) *m/z* calcd for $C_{16}H_{26}O_3Si$ $[M+H]^+$ 295.1729, found 295.1725.

Synthesis of ethyl 2-(6-((triisopropylsilyl)oxy)naphthalen-2-yl)propanoate **(10f)***:*

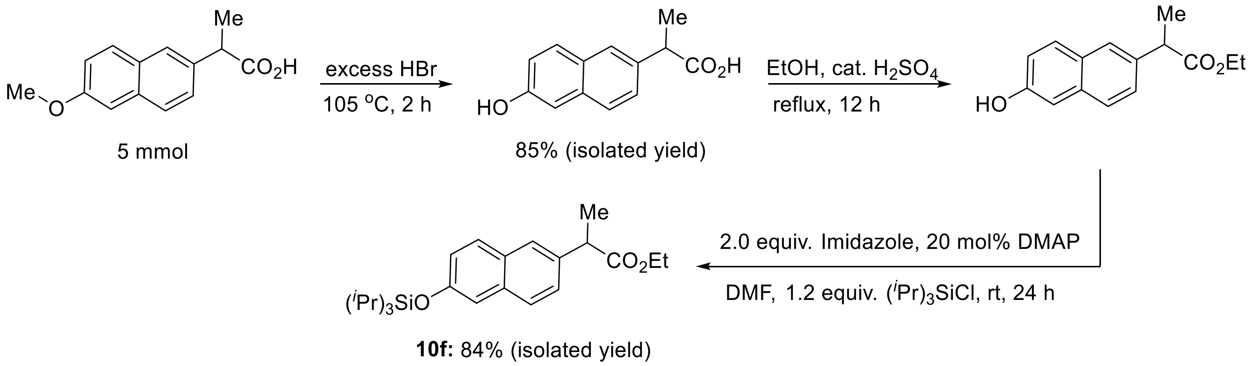

Step I: An oven dried 100 mL round-bottomed flask was charged with naproxen (1.15 g, 5 mmol), HBr (48% w/w in water, 50.0 mL) and heated to reflux for 2 h. After 2 h, the mixture was allowed to cool to room temperature and filtered. The solid was washed with ice cold water and dried under vacuum and gave 918 mg (85%) 2-(6-hydroxynaphthalen-2-yl)propanoic acid as off white solid. Spectral data are in accordance with the reported data.[38]

Step II: A 35 mL pressure tube was charged with a mixture of 2-(6-hydroxynaphthalen-2-yl)propanoic acid (918 mg, 4.2 mmol), EtOH (15 mL) and catalytic amount of H_2SO_4. Then it was refluxed for 12 h with vigorous stirring. After that, the reaction mixture was cooled to room temperature and neutralize with aqueous Na_2CO_3. The whole mixture was then extracted with dichloromethane (30 mL x 3) and washed with water. The combined organic layer dried over anhydrous Na_2SO_4, filtered and concentrated under reduced pressure to afford the ethyl 2-(6-hydroxynaphthalen-2-yl)propanoate quantitatively which was directly used for the next step without further purifications.

Step III: In a dry 100 mL round-bottomed flask crude ethyl 2-(6-hydroxynaphthalen-2-yl)propanoate, imidazole (572 mg, 8.4 mmol, 2.0 equiv.), 4-Dimethylaminopyridine (DMAP) (102 mg, 0.84 mmol, 20 mol%) and 20 mL dry DMF was added. Then stirred for 5 minutes at room temperature followed by dropwise addition of triisopropylsilyl chloride (TIPSCl) (1.04 ml, 5.04 mmol, 1.2 equiv.) via syringe. The mixture was stirred at the same temperature for additional 24 h. After completion (judged by TLC), the reaction mixture was diluted with cold water (30 mL) and extracted with ethyl acetate (30 mL x 3). The combined organic layer washed with cold water (30 mL x 3), brine (50 mL) and dried over anhydrous Na_2SO_4, filtered and concentrated under reduced pressure. The resulting mixture was purified by silica gel chromatography (2% ethyl

acetate in hexane as eluent) gave 1.4 g (84%) ethyl 2-(6-((triisopropylsilyl)oxy)naphthalen-2-yl)propanoate **(10f)** as a colourless liquid.

^{1}H NMR (400 MHz, $CDCl_3$): δ 7.72 – 7.67 (m, 3H), 7.42 (dd, *J* = 8.4, 1.6 Hz, 1H), 7.23 (d, *J* = 2.0 Hz, 1H), 7.15 (dd, *J* = 8.8, 2.4 Hz, 1H), 4.23 – 4.09 (m, 2H), 3.86 (q, *J* = 7.2 Hz, 1H), 1.59 (d, *J* = 7.2 Hz, 3H), 1.37 – 1.31 (m, 3H), 1.23 (t, *J* = 6.8 Hz, 3H), 1.16 (d, *J* = 7.2 Hz, 18H).
^{13}C NMR (100 MHz, $CDCl_3$): δ 174.8, 154.0, 135.9, 133.9, 129.2, 129.2, 127.1, 126.1, 125.9, 122.4, 114.3, 60.9, 45.6, 18.7, 18.1, 14.2, 12.8.
HRMS (ESI) *m/z* calcd for $C_{24}H_{36}O_3Si$ $[M+H]^+$ 401.2512, found 401.2513.

Synthesis of triisopropyl(4-((tetrahydro-2H-pyran-2-yl)oxy)phenoxy)silane **(10g)***:*

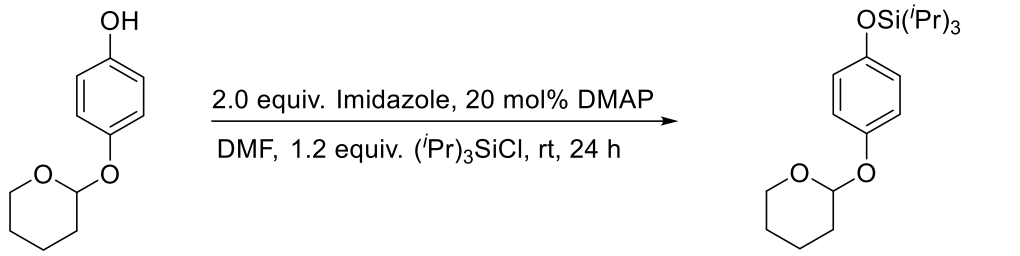

In a dry 100 mL round-bottomed flask 4-((tetrahydro-2H-pyran-2-yl)oxy)phenol (1.94 g, 10 mmol), imidazole (1.36 g, 20 mmol, 2.0 equiv.), 4-Dimethylaminopyridine (DMAP) (244 mg, 2 mmol, 20 mol%) and 20 mL dry DMF was added. Then stirred for 5 minutes at room temperature followed by dropwise addition of triisopropylsilyl chloride (TIPSCl) (2.5 ml, 12 mmol, 1.2 equiv.) via syringe. The mixture was stirred at the same temperature for additional 24 h. After completion (judged by TLC), the reaction mixture was diluted with cold water (30 mL) and extracted with ethyl acetate (30 mL x 3). The combined organic layer washed with cold water (30 mL x 3), brine (50 mL) and dried over anhydrous Na_2SO_4, filtered and concentrated under reduced pressure. The resulting mixture was purified by silica gel chromatography (2% ethyl acetate in hexane as eluent) gave 3.0 g (86%) triisopropyl(4-((tetrahydro-2H-pyran-2-yl)oxy)phenoxy)silane **(10g)** as a colourless liquid.

^{1}H NMR (400 MHz, $CDCl_3$): δ 6.94 – 6.90 (m, 2H), 6.81 – 6.77 (m, 2H), 5.28 (t, *J* = 3.2 Hz, 1H), 3.98 – 3.92 (m, 1H), 3.62 – 3.58 (m, 1H), 2.05 – 1.94 (m, 1H), 1.89 – 1.80 (m, 2H), 1.71 – 1.56 (m, 3H), 1.28 – 1.21 (m, 3H), 1.10 (d, *J* = 7.2 Hz, 18H).
^{13}C NMR (100 MHz, $CDCl_3$): δ 151.4, 150.7, 120.3, 117.5, 97.4, 62.2, 30.7, 25.4, 19.1, 18.0, 12.7.

HRMS (ESI) *m/z* calcd for $C_{20}H_{34}O_3Si$ $[M+H]^+$ 351.2355, found 351.2347.

Synthesis of 3,3,5-trimethylcyclohexyl 2-((triisopropylsilyl)oxy)benzoate **(10h)***:*

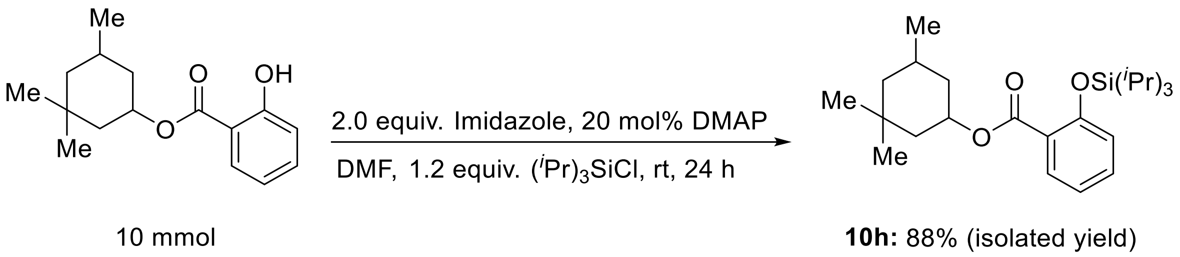

In a dry 100 mL round-bottomed flask 3,3,5-trimethylcyclohexyl 2-hydroxybenzoate (2.62 g, 10 mmol), imidazole (1.36 g, 20 mmol, 2.0 equiv.), 4-Dimethylaminopyridine (DMAP) (244 mg, 2 mmol, 20 mol%) and 20 mL dry DMF was added. Then stirred for 5 minutes at room temperature followed by dropwise addition of triisopropylsilyl chloride (TIPSCl) (2.5 ml, 12 mmol, 1.2 equiv.) via syringe. The mixture was stirred at the same temperature for additional 24 h. After completion (judged by TLC), the reaction mixture was diluted with cold water (30 mL) and extracted with ethyl acetate (30 mL x 3). The combined organic layer washed with cold water (30 mL x 3), brine (50 mL) and dried over anhydrous Na_2SO_4, filtered and concentrated under reduced pressure. The resulting mixture was purified by silica gel chromatography (2% ethyl acetate in hexane as eluent) gave 3.7 g (88%) 3,3,5-trimethylcyclohexyl 2-((triisopropylsilyl)oxy)benzoate **(10h)** as a colourless liquid.

^{1}H NMR (400 MHz, CDCl3): δ 7.62 (dd, J = 7.6, 1.2 Hz, 1H), 7.31 – 7.27 (m, 1H), 6.92 (t, J = 7.6 Hz, 1H), 6.86 (d, J = 8.0 Hz, 1H), 5.15 – 5.07 (m, 1H), 2.15 – 2.12 (m, 1H), 1.81 – 1.73 (m, 2H), 1.39 – 1.22 (m, 6H), 1.12 (d, J = 7.2 Hz, 18H), 0.98 (d, J = 8.4 Hz, 6H), 0.93 (d, J = 6.4 Hz, 3H), 0.82 (t, J = 12.8 Hz, 1H).
^{13}C NMR (100 MHz, CDCl3): δ 166.3, 155.2, 132.3, 131.0, 124.1, 120.4, 120.2, 71.5, 47.8, 44.2, 40.7, 33.2, 32.4, 27.3, 25.7, 22.5, 18.1, 13.3.
HRMS (ESI) *m/z* calcd for $C_{25}H_{42}O_3Si$ $[M+H]^+$ 419.2981, found 419.2972.

B. Preparation of Ligands

Synthesis of 6,6'-dimethyl-2,2'-bipyridine **(L4)***:*

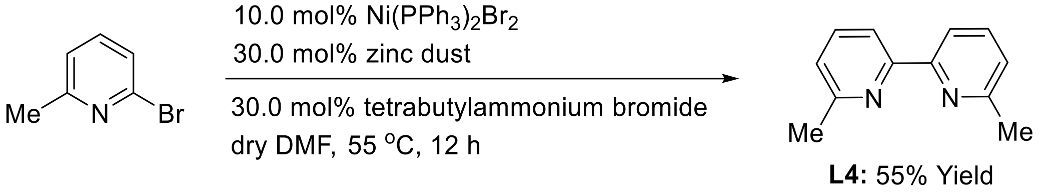

A 100 mL oven dried round bottom flask was charged with $Ni(PPh_3)_2Br_2$ (742 mg, 10.0 mol%), zinc dust (196 mg, 30.0 mol%), tetrabutylammonium bromide (965 mg, 30.0 mol%) and 2-bromo-6-methylpyridine (1.72 g, 10 mmol). After that flask was evacuated and refilled with argon, dry DMF (30 mL) was added and the suspension was stirred at 55 °C for 12 h. After completion of the reaction (monitored by TLC), the mixture was filtered through Celite and washed with Et_2O (150 mL). The combined solution was then washed with water (100 mL x 3) and brine (100 mL), dried over anhydrous Na_2SO_4, filtered and concentrated under reduced pressure. The crude material was purified by column chromatography with silica gel (10% ethyl acetate in hexane as eluent) to afford 1.01 g (55%) of the desired 6,6'-dimethyl-2,2'-bipyridine **(L4)** as a white solid. Spectral data are in accordance with the reported data.[39]

Synthesis of 6-methoxy-2,2'-bipyridine **(L5)***:*

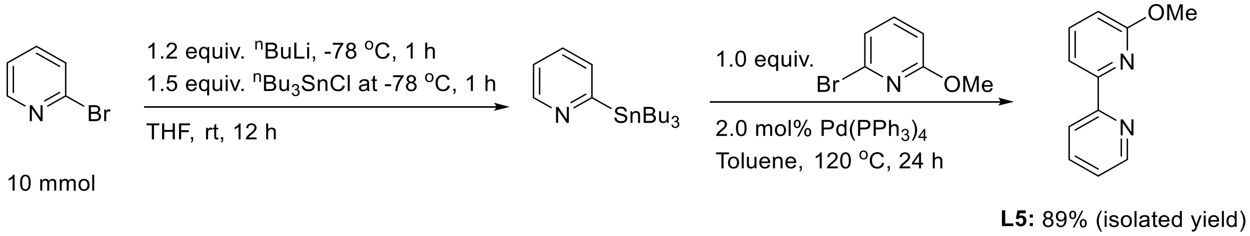

Step I: A degassed two necked round-bottom flask was charged with 2-bromopyridine (1.56 gm, 10 mmol) under argon atmosphere. Then 50 mL of dry THF was added drop wise through syringe and cooled to -78 °C followed by the dropwise addition of ^{n}BuLi (2.5 M solution in n-hexane, 1.2 equiv., 4.8 mL, 12 mmol). Then the mixture was stirred for 1 h at the same temperature. Afterward, tributyltin chloride (4 mL, 15 mmol, 1.5 equiv.) was added through the syringe, and the reaction mixture was stirred additional 1 h at -78 °C and then at room temperature for 12 h. Then a saturated solution of NH_4Cl poured into the reaction mixture and stirred for 15 minutes. The mixture was extracted with ethylacetate (3 x 50 mL) and dried over Na_2SO_4. Finally, the solvent was evaporated

under reduced pressure to get 2-(tributylstannyl)pyridine as a yellowish gummy liquid which was pure enough to use directly for the next step without further purifications.

Step II: To a degassed 250 mL two neck round-bottom flask equipped with a magnetic stirrer and a reflux condenser under an argon atmosphere were added 2-bromo-6-methoxypyridine (1.86 g, 10 mmol, 1.0 equiv.) and $Pd(PPh_3)_4$ (231 mg, 2.0 mol%). Then previously prepared crude 2-(tributylstannyl)pyridine was added using 50 mL of dry toluene under an argon atmosphere. Then the system was degassed 3 times and then heated at 120 ^oC for 24 h with continuous stirring. After that the reaction mixture was cooled to room temperature, 50 mL of aqueous NaOH (2M) was added and stirred at room temperature for 15 minute. Then the mixture was extracted with ethylacetate (3 x 50 mL), dried over Na_2SO_4 and solvent was evaporated under reduced pressure. Finally the chromatographic separation was done with silica gel (10% ethyl acetate in hexane) to gave 6-methoxy-2,2'-bipyridine **(L5)** (1.6 gm, 89% isolated yield) as a colourless gummy liquid. Spectral data are in accordance with the reported data.[40]

Synthesis of [2,2'-bipyridin]-6-ol **(L6)***:*

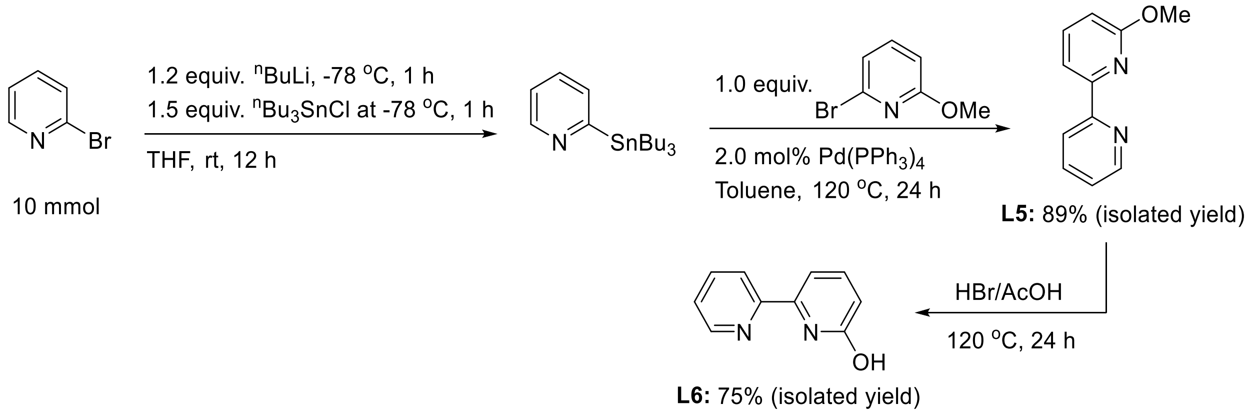

Step I: A degassed two necked round-bottom flask was charged with 2-bromopyridine (1.56 gm, 10 mmol) under argon atmosphere. Then 50 mL of dry THF was added drop wise through syringe and cooled to -78 °C followed by the dropwise addition of ^{n}BuLi (2.5 M solution in n-hexane, 1.2 equiv., 4.8 mL, 12 mmol). Then the mixture was stirred for 1 h at the same temperature. Afterward, tributyltin chloride (4 mL, 15 mmol, 1.5 equiv.) was added through the syringe, and the reaction mixture was stirred additional 1 h at -78 °C and then at room temperature for 12 h. Then a saturated solution of NH_4Cl poured into the reaction mixture and stirred for 15 minutes. The mixture was extracted with ethylacetate (3 x 50 mL) and dried over Na_2SO_4. Finally, the solvent was evaporated

under reduced pressure to get 2-(tributylstannyl)pyridine as a yellowish gummy liquid which was pure enough to use directly for the next step without further purifications.

Step II: To a degassed 250 mL two neck round-bottom flask equipped with a magnetic stirrer and a reflux condenser under an argon atmosphere were added 2-bromo-6-methoxypyridine (1.86 g, 10 mmol, 1.0 equiv.) and $Pd(PPh_3)_4$ (231 mg, 2.0 mol%). Then previously prepared crude 2-(tributylstannyl)pyridine was added using 50 mL of dry toluene under an argon atmosphere. Then the system was degassed 3 times and then heated at 120 ^oC for 24 h with continuous stirring. After that the reaction mixture was cooled to room temperature, 50 mL of aqueous NaOH (2M) was added and stirred at room temperature for 15 minute. Then the mixture was extracted with ethylacetate (3 x 50 mL), dried over Na_2SO_4 and solvent was evaporated under reduced pressure. Finally the chromatographic separation was done with silica gel (10% ethyl acetate in hexane) to gave 6-methoxy-2,2'-bipyridine (1.6 gm, 89% isolated yield) as a colourless gummy liquid which was used for the next step. Spectral data are in accordance with the reported data.[40]

Step III: To 6-methoxy-2,2'-bipyridine (1.6 gm, 8.6 mmol) was added a solution of 47% HBr (5.0 equiv., 73 mL) and glacial acetic acid (5.0 equiv., 2.7 mL) and the mixture was refluxed at 120 ^oC for 24 hours. Then the mixture was cooled at room temperature, concentrated in vacuo and in the resulting solid 20 mL water was added. Then pH was neutralized (pH ~7) with 1 M NaOH solution, after which the mixture was extracted with $CHCl_3$ (3 x 50 mL) and dried over Na_2SO_4. Finally, the solvent was evaporated under reduced pressure followed by chromatographic separation with neutral silica gel (100% EtOAc as a eluent) gave [2,2'-bipyridin]-6-ol **(L6)** (1.1 gm, 75% isolated yield) as a white solid. Spectral data are in accordance with the reported data.[41]

Synthesis of [2,2'-bipyridine]-6,6'-diol **(L7)***:*

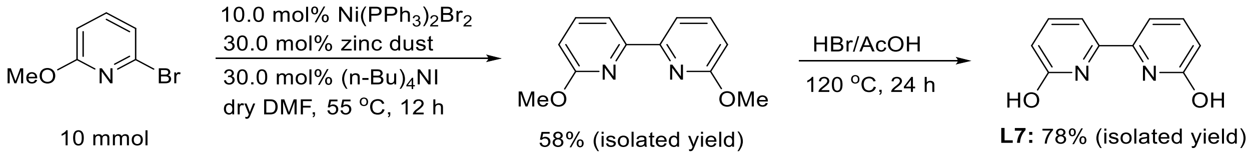

Step I: A 100 mL oven dried round bottom flask was charged with $Ni(PPh_3)_2Br_2$ (742 mg, 10.0 mol%), zinc dust (196 mg, 30.0 mol%), tetrabutylammonium bromide (965 mg, 30.0 mol%) and 2-bromo-6-methoxypyridine (1.86 g, 10 mmol). After that flask was evacuated and refilled with argon, dry DMF (30 mL) was added and the suspension was stirred at 55 °C for 12 h. After

completion of the reaction (monitored by TLC), the mixture was filtered through Celite and washed with Et_2O (150 mL). The combined solution was then washed with water (100 mL x 3) and brine (100 mL), dried over anhydrous Na_2SO_4, filtered and concentrated under reduced pressure. The crude material was purified by column chromatography with silica gel (10% ethyl acetate in hexane as eluent) to afford 1.25 (58%) of the desired 6,6'-dimethoxy-2,2'-bipyridine as a white solid. Spectral data are in accordance with the reported data.[42]

Step II: To 6,6'-dimethoxy-2,2'-bipyridine (1.25 gm, 5.78 mmol) was added a solution of 47% HBr (5.0 equiv., 50 mL) and glacial acetic acid (5.0 equiv., 1.8 mL) and the mixture was refluxed at 120 ^{o}C for 24 hours. Then the mixture was cooled at room temperature, concentrated in vacuo and in the resulting solid 20 mL water was added. Then pH was neutralized (pH ~7) with 1 M NaOH solution, after which the mixture was extracted with $CHCl_3$ (3 x 50 mL) and dried over Na_2SO_4. Finally, the solvent was evaporated under reduced pressure followed by chromatographic separation with neutral silica gel (100% EtOAc as eluent) gave [2,2'-bipyridine]-6,6'-diol **(L7)** (1.1 gm, 78% isolated yield) as a off white solid. Spectral data are in accordance with the reported data.[43]

Synthesis of 4'-methyl-[2,2'-bipyridin]-6-ol **(L8)***:*

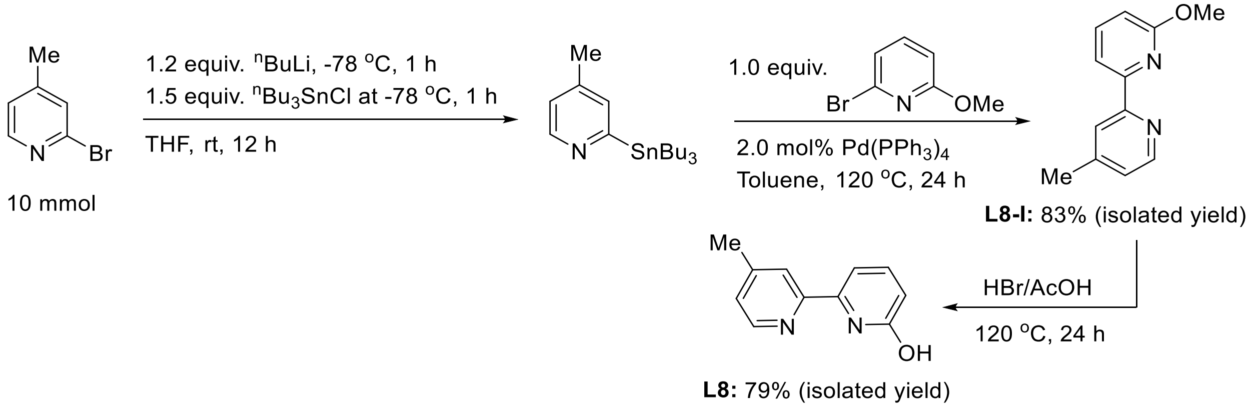

Step I: A degassed two necked round-bottom flask was charged with 2-bromo-4-methylpyridine (1.7 g, 10 mmol) under argon atmosphere. Then 50 mL of dry THF was added drop wise through syringe and cooled to -78 °C followed by the dropwise addition of ^{n}BuLi (2.5 M solution in n-hexane, 1.2 equiv., 4.8 mL, 12 mmol). Then the mixture was stirred for 1 h at the same temperature. Afterward, tributyltin chloride (4 mL, 15 mmol, 1.5 equiv.) was added through the syringe, and the reaction mixture was stirred additional 1 h at -78 °C and then at room temperature for 12 h. Then a saturated solution of NH_4Cl poured into the reaction mixture and stirred for 15 minutes.

The mixture was extracted with ethylacetate (3 x 50 mL) and dried over Na_2SO_4. Finally, the solvent was evaporated under reduced pressure to get 4-methyl-2-(tributylstannyl)pyridine as a yellowish gummy liquid which was pure enough to use directly for the next step without further purifications.

Step II: To a degassed 250 mL two neck round-bottom flask equipped with a magnetic stirrer and a reflux condenser under an argon atmosphere were added 2-bromo-6-methoxypyridine (1.8 gm, 10 mmol, 1.0 euiv.) and $Pd(PPh_3)_4$ (231 mg, 2.0 mol%). Then previously prepared crude 4-methyl-2-(tributylstannyl)pyridine was added using 50 mL of dry toluene under an argon atmosphere. Then the system was degassed 3 times and then heated at 120 ^oC for 24 h with continuous stirring. After that the reaction mixture was cooled to room temperature, 50 mL of aqueous NaOH (2M) was added and stirred at room temperature for 15 minute. Then the mixture was extracted with ethylacetate (3 x 50 mL), dried over Na_2SO_4 and solvent was evaporated under reduced pressure. Finally the chromatographic separation was done with silica gel (10% ethyl acetate in hexane as eluent) to gave 6'-methoxy-4-methyl-2,2'-bipyridine **(L8-I)** (1.6 gm, 83% isolated yield) as a colourless gummy liquid which was used for the next step.

^{1}H NMR (400 MHz, $CDCl_3$): δ 8.41 (d, J = 4.8 Hz, 1H), 8.11 (s, 1H), 7.95 (d, J = 7.6 Hz, 1H), 7.57 (t, J = 7.2 Hz, 1H), 6.96 (s, 1H), 6.66 (d, J = 8.0 Hz, 1H), 3.95 (s, 3H), 2.29 (s, 3H).
^{13}C NMR (100 MHz, $CDCl_3$): δ 163.4, 155.8, 153.6, 148.8, 147.7, 139.3, 124.4, 121.7, 113.8, 110.8, 53.2, 21.2.
HRMS (ESI) *m/z* calcd for $C_{12}H_{12}N_2O$ $[M+H]^+$ 201.1028, found 201.1024.

Step III: To 6'-methoxy-4-methyl-2,2'-bipyridine (1.6 gm, 8 mmol) was added a solution of 47% HBr (5.0 equiv., 68 mL) and glacial acetic acid (5.0 equiv., 2.5 mL) and the mixture was refluxed at 120 ^oC for 24 hours. Then the mixture was cooled at room temperature, concentrated in vacuo and in the resulting solid 20 mL water was added. Then pH was neutralized (pH ~7) with 1 M NaOH solution, after which the mixture was extracted with $CHCl_3$ (3 x 50 mL) and dried over Na_2SO_4. Finally, the solvent was evaporated under reduced pressure followed by chromatographic separation with neutral silica gel (100% EtOAc as eluent) gave 4'-methyl-[2,2'-bipyridin]-6-ol **(L8)** (1.1 gm, 79% isolated yield) as a white solid.

^{1}H NMR (400 MHz, $CDCl_3$): δ 10.71 (brs, 1H), 8.47 (d, J = 4.4 Hz, 1H), 7.61 (s, 1H), 7.46 (dd, J = 8.8, 7.2 Hz, 1H), 7.15 (d, J = 4.4 Hz, 1H), 6.77 (d, J = 6.8 Hz, 1H), 6.60 (d, J = 8.8 Hz, 1H), 2.42 (s, 3H).

^{13}C NMR (100 MHz, $CDCl_3$): δ 163.0, 149.0, 148.8, 147.8, 142.0, 140.7, 125.7, 122.0, 120.6, 102.6, 21.4.

HRMS (ESI) *m/z* calcd for $C_{11}H_{10}N_2O$ $[M+H]^+$ 187.0871, found 187.0858.

Synthesis of 4'-(tert-butyl)-[2,2'-bipyridin]-6-ol **(L9)***:*

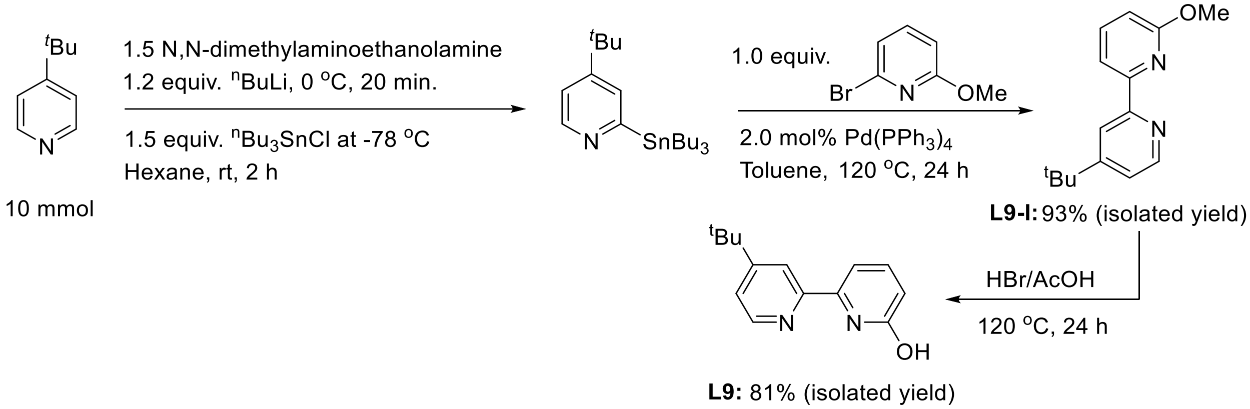

Step I: A degassed two necked round-bottom flask was charged with N,N-dimethylaminoethanolamine (1.7 g, 15 mmol) under argon atmosphere. Then 50 mL of dry hexane was added through syringe and cooled to 0 °C followed by the dropwise addition of ^{n}BuLi (2.5 M solution in n-hexane, 1.2 equiv., 4.8 mL, 12 mmol). Then the mixture was stirred for 20 min followed by addition of 4-(tert-butyl)pyridine (1.35 g, 10 mmol) at 0 °C. Afterward, the resulting mixture was cooled to -78 °C and tributyltin chloride (4 mL, 15 mmol, 1.5 equiv.) was added through the syringe. The mixture was allowed to warm at room temperature and stirred for additional 2h. Then a saturated solution of NH_4Cl poured into the reaction mixture and stirred for 15 minutes. The mixture was extracted with ethylacetate (3 x 50 mL) and dried over Na_2SO_4. Finally, the solvent was evaporated under reduced pressure to get 4-(tert-butyl)-2-(tributylstannyl)pyridine as a yellowish gummy liquid Which was pure enough to use directly for the next step without further purifications.

Step II: To a degassed 250 mL two neck round-bottom flask equipped with a magnetic stirrer and a reflux condenser under an argon atmosphere were added 2-bromo-6-methoxypyridine (1.8 gm, 10 mmol, 1.0 equiv.) and $Pd(PPh_3)_4$ (231 mg, 2.0 mol%). Then previously prepared crude 4-(tert-

butyl)-2-(tributylstannyl)pyridine was added using 50 mL of dry toluene under an argon atmosphere. Then the system was degassed 3 times and then heated at 120 °C for 24 h with continuous stirring. After that the reaction mixture was cooled to room temperature, 50 mL of aqueous NaOH (2M) was added and stirred at room temperature for 15 minute. Then the mixture was extracted with ethylacetate (3 x 50 mL), dried over Na_2SO_4 and solvent was evaporated under reduced pressure. Finally the chromatographic separation was done with silica gel (10% ethyl acetate in hexane) to gave 4-(tert-butyl)-6'-methoxy-2,2'-bipyridine **(L9-I)** (2.2 gm, 93% isolated yield) as a colourless gummy liquid which was used for the next step.

^{1}H NMR (400 MHz, $CDCl_3$): δ 8.46 (d, *J* = 5.2 Hz, 1H), 8.35 (d, *J* = 2.0 Hz, 1H), 7.91 (d, *J* = 7.2 Hz, 1H), 7.58 (t, *J* = 8.0 Hz, 1H), 7.16 (dd, *J* = 5.2, 2.0 Hz, 1H), 6.66 (d, *J* = 8.0 Hz, 1H), 3.96 (s, 3H), 1.27 (s, 9H).

^{13}C NMR (100 MHz, $CDCl_3$): δ 163.5, 160.8, 156.0, 153.9, 149.1, 139.4, 120.7, 117.9, 113.9, 110.8, 53.2, 34.9, 30.6.

HRMS (ESI) *m/z* calcd for $C_{15}H_{18}N_2O$ $[M+H]^+$ 243.1497, found 243.1497.

Step III: To 4-(tert-butyl)-6'-methoxy-2,2'-bipyridine (2.2 gm, 9.1 mmol) was added a solution of 47% HBr (5.0 equiv., 76 mL) and glacial acetic acid (5.0 equiv., 2.6 mL) and the mixture was refluxed at 120 °C for 24 hours. Then the mixture was cooled at room temperature concentrated, in vacuo and in the resulting solid 20 mL water was added. Then pH was neutralized (pH ~7) with 1 M NaOH solution, after which the mixture was extracted with $CHCl_3$ (3 x 50 mL) and dried over Na_2SO_4. Finally, the solvent was evaporated under reduced pressure followed by chromatographic separation with neutral silica gel (100% EtOAc as eluent) gave 4'-(tert-butyl)-[2,2'-bipyridin]-6-ol **(L9)** (1.6 gm, 81% isolated yield) as a white solid.

^{1}H NMR (400 MHz, $CDCl_3$): δ 10.82 (brs, 1H), 8.52 (d, *J* = 5.2 Hz, 1H), 7.79 (s, 1H), 7.47 (dd, *J* = 9.2, 6.8 Hz, 1H), 7.32 (dd, *J* = 5.6, 1.6 Hz, 1H), 6.83 (d, *J* = 6.8 Hz, 1H), 6.60 (d, *J* = 9.2 Hz, 1H), 1.35 (s, 9H).

^{13}C NMR (100 MHz, CDCl3): δ 163.1, 161.8, 149.2, 148.0, 142.4, 140.7, 122.0, 121.9, 116.7, 102.7, 35.1, 30.6.

HRMS (ESI) *m/z* calcd for $C_{14}H_{16}N_2O$ $[M+H]^+$ 229.1341, found 229.1337.

Synthesis of 6'-methyl-[2,2'-bipyridin]-6-ol **(L11)*:***

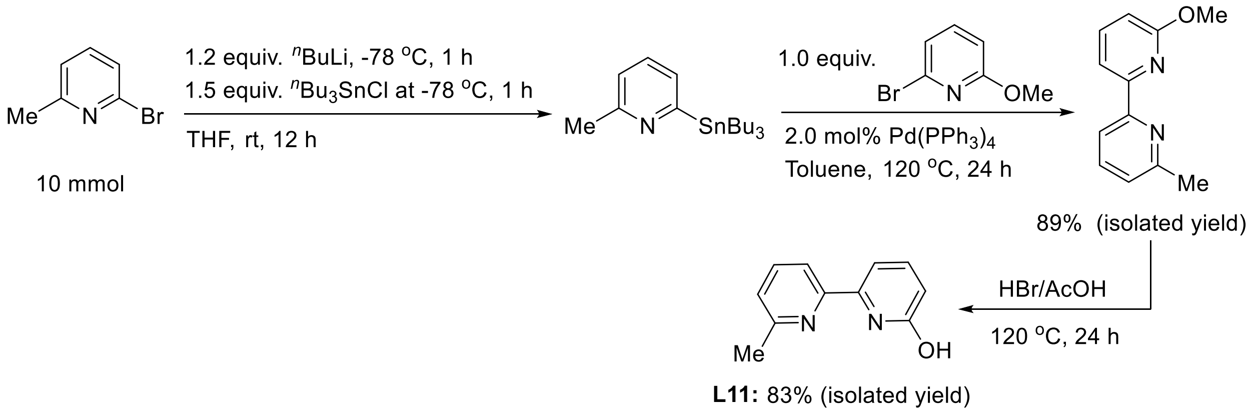

Step I: A degassed two necked round-bottom flask was charged with 2-bromo-6-methylpyridine (1.7 g, 10 mmol) under argon atmosphere. Then 50 mL of dry THF was added drop wise through syringe and cooled to -78 °C followed by the dropwise addition of ^{n}BuLi (2.5 M solution in n-hexane, 1.2 equiv., 4.8 mL, 12 mmol). Then the mixture was stirred for 1 h at the same temperature. Afterward, tributyltin chloride (4 mL, 15 mmol, 1.5 equiv.) was added through the syringe, and the reaction mixture was stirred additional 1 h at -78 °C and then at room temperature for 12 h. Then a saturated solution of NH_4Cl poured into the reaction mixture and stirred for 15 minutes. The mixture was extracted with ethylacetate (3 x 50 mL) and dried over Na_2SO_4. Finally, the solvent was evaporated under reduced pressure to get 6-methyl-2-(tributylstannyl)pyridine as a yellowish gummy liquid which was pure enough to use directly for the next step without further purifications.

Step II: To a degassed 250 mL two neck round-bottom flask equipped with a magnetic stirrer and a reflux condenser under an argon atmosphere were added 2-bromo-6-methoxypyridine (1.8 gm, 10 mmol, 1.0 euiv.) and $Pd(PPh_3)_4$ (231 mg, 2.0 mol%). Then previously prepared crude 6-methyl-2-(tributylstannyl)pyridine was added using 50 mL of dry toluene under an argon atmosphere. Then the system was degassed 3 times and then heated at 120 °C for 24 h with continuous stirring. After that the reaction mixture was cooled to room temperature, 50 mL of aqueous NaOH (2M) was added and stirred at room temperature for 15 minute. Then the mixture was extracted with ethylacetate (3 x 50 mL), dried over Na_2SO_4 and solvent was evaporated under reduced pressure. Finally the chromatographic separation was done with silica gel (10% ethyl acetate in hexane as eluent) to gave 6-methoxy-6'-methyl-2,2'-bipyridine (1.8 gm, 89% isolated yield) as a colourless

gummy liquid which was used for the next step. Spectral data are in accordance with the reported data.[44]

Step III: To 6-methoxy-6'-methyl-2,2'-bipyridine (1.8 gm, 9 mmol) was added a solution of 47% HBr (5.0 equiv., 76 mL) and glacial acetic acid (5.0 equiv., 2.8 mL) and the mixture was refluxed at 120 ^{o}C for 24 hours. Then the mixture was cooled at room temperature, concentrated in vacuo and in the resulting solid 20 mL water was added. Then pH was neutralized (pH ~7) with 1 M NaOH solution, after which the mixture was extracted with $CHCl_3$ (3 x 50 mL) and dried over Na_2SO_4. Finally, the solvent was evaporated under reduced pressure followed by chromatographic separation with neutral silica gel (100% EtOAc as eluent) gave 6'-methyl-[2,2'-bipyridin]-6-ol **(L11)** (1.4 gm, 83% isolated yield) as a reddish white solid. Spectral data are in accordance with the reported data.[41]

Synthesis of 6-methyl-2,2'-bipyridine **(L12)***:*

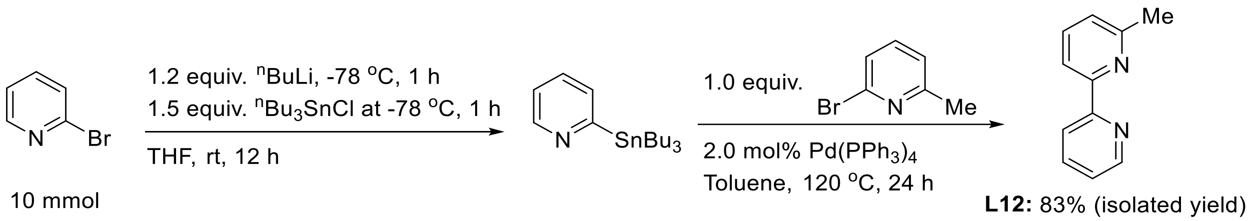

L12: 83% (isolated yield)

Step I: A degassed two necked round-bottom flask was charged with 2-bromopyridine (1.56 gm, 10 mmol) under argon atmosphere. Then 50 mL of dry THF was added drop wise through syringe and cooled to -78 °C followed by the dropwise addition of ^{n}BuLi (2.5 M solution in n-hexane, 1.2 equiv., 4.8 mL, 12 mmol). Then the mixture was stirred for 1 h at the same temperature. Afterward, tributyltin chloride (4 mL, 15 mmol, 1.5 equiv.) was added through the syringe, and the reaction mixture was stirred additional 1 h at -78 °C and then at room temperature for 12 h. Then a saturated solution of NH_4Cl poured into the reaction mixture and stirred for 15 minutes. The mixture was extracted with ethylacetate (3 x 50 mL) and dried over Na_2SO_4. Finally, the solvent was evaporated under reduced pressure to get 2-(tributylstannyl)pyridine as a yellowish gummy liquid which was pure enough to use directly for the next step without further purifications.

Step II: To a degassed 250 mL two neck round-bottom flask equipped with a magnetic stirrer and a reflux condenser under an argon atmosphere were added 2-bromo-6-methylpyridine (1.7 g, 10 mmol, 1.0 equiv.) and $Pd(PPh_3)_4$ (231 mg, 2.0 mol%). Then previously prepared crude 2-(tributylstannyl)pyridine was added using 50 mL of dry toluene under an argon atmosphere. Then

the system was degassed 3 times and then heated at 120 ^{o}C for 24 h with continuous stirring. After that the reaction mixture was cooled to room temperature, 50 mL of aqueous NaOH (2M) was added and stirred at room temperature for 15 minute. Then the mixture was extracted with ethylacetate (3 x 50 mL), dried over Na_2SO_4 and solvent was evaporated under reduced pressure. Finally the chromatographic separation was done with silica gel (10% ethyl acetate in hexane) to gave 6-methyl-2,2'-bipyridine (1.4 gm, 83% isolated yield) **(L12)** as a colourless gummy liquid. Spectral data are in accordance with the reported data.[45]

Synthesis of 6-phenyl-2,2'-bipyridine (**L13**)*:*

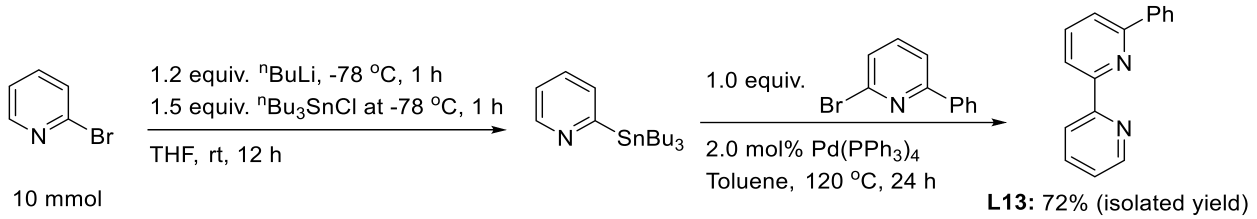

Step I: A degassed two necked round-bottom flask was charged with 2-bromopyridine (1.56 gm, 10 mmol) under argon atmosphere. Then 50 mL of dry THF was added drop wise through syringe and cooled to -78 °C followed by the dropwise addition of ^{n}BuLi (2.5 M solution in n-hexane, 1.2 equiv., 4.8 mL, 12 mmol). Then the mixture was stirred for 1 h at the same temperature. Afterward, tributyltin chloride (4 mL, 15 mmol, 1.5 equiv.) was added through the syringe, and the reaction mixture was stirred additional 1 h at -78 °C and then at room temperature for 12 h. Then a saturated solution of NH_4Cl poured into the reaction mixture and stirred for 15 minutes. The mixture was extracted with ethylacetate (3 x 50 mL) and dried over Na_2SO_4. Finally, the solvent was evaporated under reduced pressure to get 2-(tributylstannyl)pyridine as a yellowish gummy liquid which was pure enough to use directly for the next step without further purifications.

Step II: To a degassed 250 mL two neck round-bottom flask equipped with a magnetic stirrer and a reflux condenser under an argon atmosphere were added 2-bromo-6-methylpyridine (2.34 g, 10 mmol, 1.0 equiv.) and $Pd(PPh_3)_4$ (231 mg, 2.0 mol%). Then previously prepared crude 2-(tributylstannyl)pyridine was added using 50 mL of dry toluene under an argon atmosphere. Then the system was degassed 3 times and then heated at 120 ^{o}C for 24 h with continuous stirring. After that the reaction mixture was cooled to room temperature, 50 mL of aqueous NaOH (2M) was added and stirred at room temperature for 15 minute. Then the mixture was extracted with ethylacetate (3 x 50 mL), dried over Na_2SO_4 and solvent was evaporated under reduced pressure.

Finally the chromatographic separation was done with silica gel (10% ethyl acetate in hexane) to gave 6-phenyl-2,2'-bipyridine (1.42 gm, 72% isolated yield) **(L13)** as a white solid. Spectral data are in accordance with the reported data.[46]

Synthesis of 4-(tert-butyl)-6'-propoxy-2,2'-bipyridine (**L14**)*:*

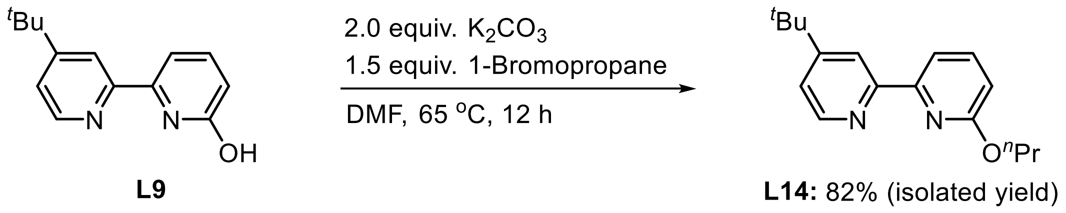

A dry 100 mL round-bottomed flask was charged with **L9** (228 mg, 1.0 mmol), K_2CO_3 (276 mg, 2.0 mmol, 2.0 equiv.), 1-Bromopropane (184 mg, 1.5 mmol, 1.5 equiv.) and dry DMF (10 mL). The reaction mixture was stirred for 12 h at 65 ^{o}C. Then the reaction mixture cool to room temperature, diluted with cold water (10 mL) and extracted with ethyl acetate (10 mL x 3). The combined organic layer washed with cold water (10 mL x 3), brine (20 mL) and dried over anhydrous Na_2SO_4, filtered and concentrated under reduced pressure. The crude mass was purified by silica gel column chromatography (10% ethyl acetate in hexane as eluent) gave **L14** (221 mg, 82%) as a colourless liquid.

^{1}H NMR (400 MHz, $CDCl_3$) δ 8.56 (d, *J* = 5.2 Hz, 1H), 8.41 (d, *J* = 1.2 Hz, 1H), 7.96 (d, *J* = 3.2 Hz, 1H), 7.68 (t, *J* = 8.0 Hz, 1H), 7.27 (dd, *J* = 5.2, 2.0 Hz, 1H), 6.75 (d, *J* = 8.4 Hz, 1H), 4.41 (t, *J* = 6.8 Hz, 2H), 1.93 – 1.84 (m, 2H), 1.38 (s, 9H), 1.07 (t, *J* = 7.6 Hz, 3H).

^{13}C NMR (100 MHz, $CDCl_3$) δ 163.3, 160.7, 156.0, 153.8, 149.0, 139.3, 120.6, 117.9, 113.6, 110.9, 67.3, 34.8, 30.5, 22.3, 10.6.

HRMS (ESI) *m/z* calcd for $C_{17}H_{22}N_2O$ $[M+H]^+$ 271.3840, found 271.2846.

Synthesis of 4-(tert-butyl)-6'-((triisopropylsilyl)oxy)-2,2'-bipyridine (**L15**)*:*

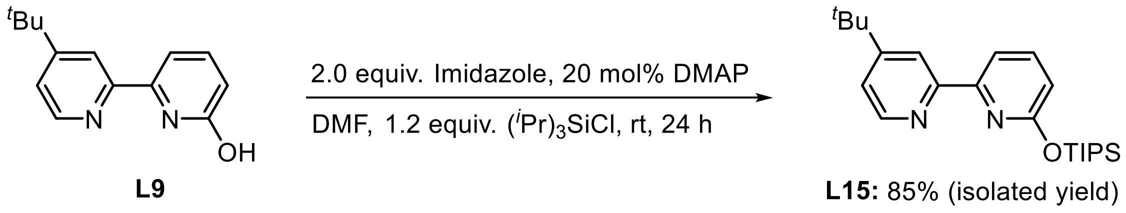

In a dry 100 mL round-bottomed flask **L9** (228 mg, 1.0 mmol), imidazole (1.36 g, 2.0 mmol, 2.0 equiv.), 4-Dimethylaminopyridine (DMAP) (24.4 mg, 0.2 mmol, 20 mol%) and 10 mL dry DMF was added. Then stirred for 5 minutes at room temperature followed by dropwise addition of triisopropylsilyl chloride (TIPSCl) (250 μl, 1.2 mmol, 1.2 equiv.) via syringe. The mixture was stirred at the same temperature for additional 24 h. After completion (judged by TLC), the reaction mixture was diluted with cold water (10 mL) and extracted with ethyl acetate (10 mL x 3). The combined organic layer washed with cold water (10 mL x 3), brine (20 mL) and dried over anhydrous Na_2SO_4, filtered and concentrated under reduced pressure. The resulting mixture was purified by silica gel chromatography (10% ethyl acetate in hexane as eluent) gave 326 mg (85%) **L15** as a colourless liquid.

^{1}H NMR (400 MHz, $CDCl_3$) δ 8.53 (d, J = 5.2 Hz, 1H), 8.36 (d, J = 1.6 Hz, 1H), 7.98 (d, J = 7.2 Hz, 1H), 7.67 (t, J = 8.0 Hz, 1H), 7.25 – 7.24 (m, 1H), 6.73 (d, J = 8.0 Hz, 1H), 1.58 – 1.50 (m, 3H), 1.35 (s, 9H), 1.14 (d, J = 7.6 Hz, 18H).
^{13}C NMR (100 MHz, $CDCl_3$) δ 162.1, 160.7, 155.9, 154.1, 148.9, 139.7, 120.5, 118.33, 114.0, 112.9, 34.8, 30.4, 18.1, 12.9, 12.3.
HRMS (ESI) *m/z* calcd for $C_{17}H_{22}N_2O$ $[M+H]^+$ 385.2675, found 385.2670.

C. Reaction development for the *Meta*-Selective Borylation

i) Screening of steering group

***General Procedure* (1a to 1l)**:

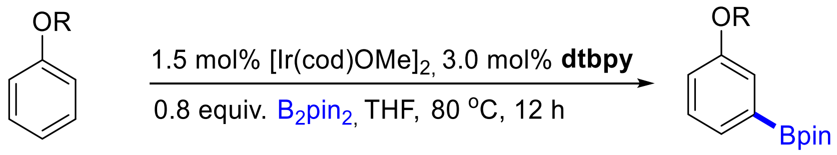

In an argon-filled glove box, a 5.0 mL Wheaton microreactor was charged with $[Ir(cod)OMe]_2$ (1.98 mg, 1.5 mol%), B_2pin_2 (40.6 mg, 0.8 equiv.), dtbpy (1.6 mg, 3.0 mol%), and dry THF (1.0 mL). The reaction mixture was stirred for 2 minutes at room temperature and then substrate (0.2 mmol) was added. The microreactor was capped with a teflon pressure cap and placed into pre-heated aluminum block at 80 °C for 12 h. After 12 h GC-MS was checked and results are shown in the above scheme.

Meta-borylation of phenol **(1a)***:*

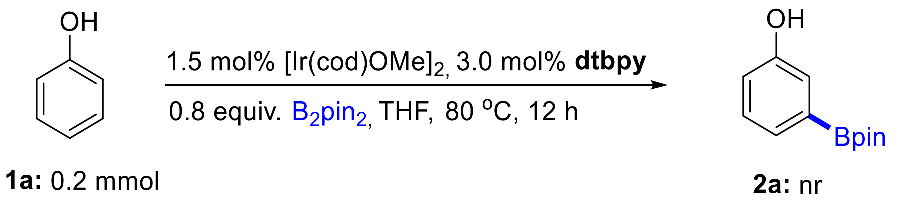

In an argon-filled glove box, a 5.0 mL Wheaton microreactor was charged with $[Ir(cod)OMe]_2$ (1.98 mg, 1.5 mol%), B_2pin_2 (40.6 mg, 0.8 equiv.), dtbpy (1.6 mg, 3.0 mol%), and dry THF (1.0 mL). The reaction mixture was stirred for 2 minutes at room temperature and then phenol (18.8 mg, 0.2 mmol) was added. The microreactor was capped with a teflon pressure cap and placed into pre-heated aluminum block at 80 ^{o}C for 12 h. After 12 h GC-MS was checked and results are shown in the above scheme.

Meta-borylation of anisole **(1b)***:*

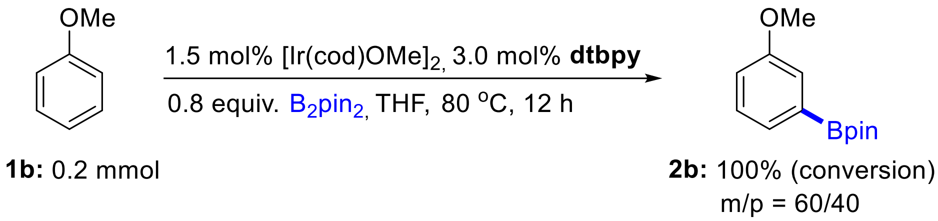

In an argon-filled glove box, a 5.0 mL Wheaton microreactor was charged with $[Ir(cod)OMe]_2$ (1.98 mg, 1.5 mol%), B_2pin_2 (40.6 mg, 0.8 equiv.), dtbpy (1.6 mg, 3.0 mol%), and dry THF (1.0 mL). The reaction mixture was stirred for 2 minutes at room temperature and then anisole (21.6 mg, 0.2 mmol) was added. The microreactor was capped with a teflon pressure cap and placed into pre-heated aluminum block at 80 ^{o}C for 12 h. After 12 h GC-MS was checked and results are shown in the above scheme.

Meta-borylation of 4,4,5,5-tetramethyl-2-phenoxy-1,3,2-dioxaborolane **(1c)***:*

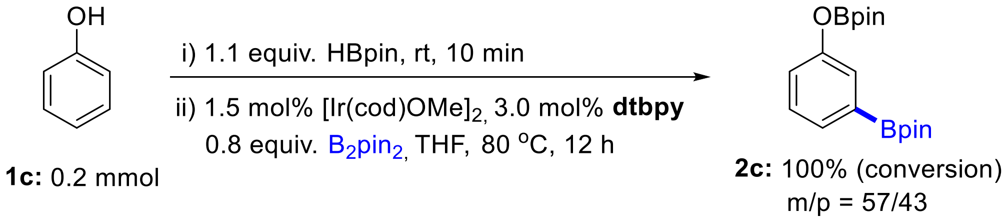

In an argon-filled glove box, a 5.0 mL Wheaton microreactor was charged with phenol (18.8 mg, 0.2 mmol) and pinacolborane (31.4 μL, 1.1 equiv) and stirred for 10 min at room temperature. To this mixture $[Ir(cod)OMe]_2$ (1.98 mg, 1.5 mol%), B_2pin_2 (40.6 mg, 0.8 equiv.), dtbpy (1.6 mg, 3.0

mol%), and dry THF (1.0 mL) was added under an inert atmosphere. Then the microreactor was capped with a teflon pressure cap and placed into a pre-heated aluminum block at 80 °C for 12 h. After 12 h GC-MS was checked and results are shown in the above scheme.

Meta-borylation of phenyl acetate **(1d)**:

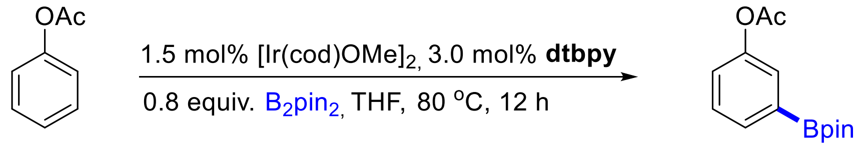

1d: 0.2 mmol

2d: 100% (conversion)
m/p = 70/30

In an argon-filled glove box, a 5.0 mL Wheaton microreactor was charged with $[Ir(cod)OMe]_2$ (1.98 mg, 1.5 mol%), B_2pin_2 (40.6 mg, 0.8 equiv.), dtbpy (1.6 mg, 3.0 mol%), and dry THF (1.0 mL). The reaction mixture was stirred for 2 minutes at room temperature and then phenyl acetate (27.2 mg, 0.2 mmol) was added. The microreactor was capped with a teflon pressure cap and placed into pre-heated aluminum block at 80 °C for 12 h. After 12 h GC-MS was checked and results are shown in the above scheme.

Meta-borylation of phenyl pivalate **(1e)**:

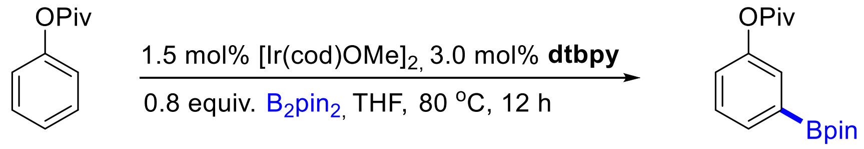

1e: 0.2 mmol

2e: 100% (conversion)
m/p = 66/34

In an argon-filled glove box, a 5.0 mL Wheaton microreactor was charged with $[Ir(cod)OMe]_2$ (1.98 mg, 1.5 mol%), B_2pin_2 (40.6 mg, 0.8 equiv.), dtbpy (1.6 mg, 3.0 mol%), and dry THF (1.0 mL). The reaction mixture was stirred for 2 minutes at room temperature and then phenyl pivalate (35.6 mg, 0.2 mmol) was added. The microreactor was capped with a teflon pressure cap and placed into pre-heated aluminum block at 80 °C for 12 h. After 12 h GC-MS was checked and results are shown in the above scheme.

Meta-borylation of tert-butyl phenyl carbonate **(1f)***:*

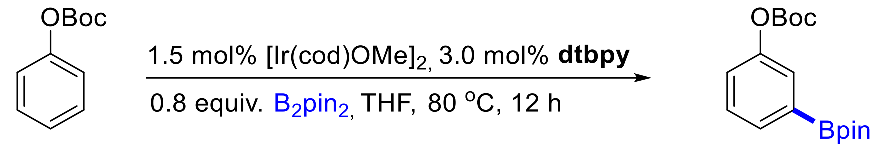

1f: 0.2 mmol

2f: 100% (conversion)
m/p = 72/28

In an argon-filled glove box, a 5.0 mL Wheaton microreactor was charged with $[Ir(cod)OMe]_2$ (1.98 mg, 1.5 mol%), B_2pin_2 (40.6 mg, 0.8 equiv.), dtbpy (1.6 mg, 3.0 mol%), and dry THF (1.0 mL). The reaction mixture was stirred for 2 minutes at room temperature and then tert-butyl phenyl carbonate (38.8 mg, 0.2 mmol) was added. The microreactor was capped with a teflon pressure cap and placed into pre-heated aluminum block at 80 °C for 12 h. After 12 h GC-MS was checked and results are shown in the above scheme.

Meta-borylation of ethyl phenyl carbonate **(1g)***:*

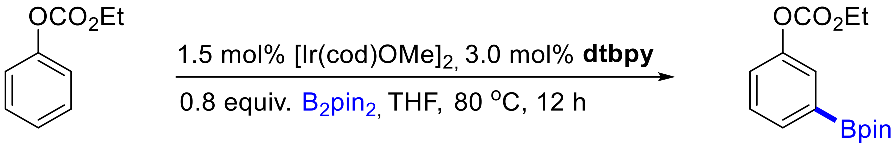

1g: 0.2 mmol

2g: 100% (conversion)
m/p = 69/31

In an argon-filled glove box, a 5.0 mL Wheaton microreactor was charged with $[Ir(cod)OMe]_2$ (1.98 mg, 1.5 mol%), B_2pin_2 (40.6 mg, 0.8 equiv.), dtbpy (1.6 mg, 3.0 mol%), and dry THF (1.0 mL). The reaction mixture was stirred for 2 minutes at room temperature and then ethyl phenyl carbonate (33.2 mg, 0.2 mmol) was added. The microreactor was capped with a teflon pressure cap and placed into pre-heated aluminum block at 80 °C for 12 h. After 12 h GC-MS was checked and results are shown in the above scheme.

Meta-borylation of phenyl diethylcarbamate **(1h)***:*

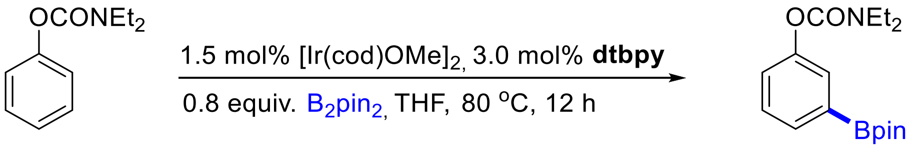

1h: 0.2 mmol

2h: 96% (conversion)
m/p = 65/35

In an argon-filled glove box, a 5.0 mL Wheaton microreactor was charged with $[Ir(cod)OMe]_2$ (1.98 mg, 1.5 mol%), B_2pin_2 (40.6 mg, 0.8 equiv.), dtbpy (1.6 mg, 3.0 mol%), and dry THF (1.0

mL). The reaction mixture was stirred for 2 minutes at room temperature and then phenyl diethylcarbamate (38.6 mg, 0.2 mmol) was added. The microreactor was capped with a teflon pressure cap and placed into pre-heated aluminum block at 80 °C for 12 h. After 12 h GC-MS was checked and results are shown in the above scheme.

Meta-borylation of phenyl methanesulfonate **(1i)**:

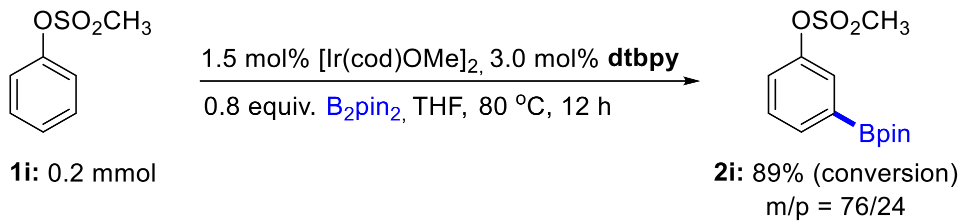

In an argon-filled glove box, a 5.0 mL Wheaton microreactor was charged with $[Ir(cod)OMe]_2$ (1.98 mg, 1.5 mol%), B_2pin_2 (40.6 mg, 0.8 equiv.), dtbpy (1.6 mg, 3.0 mol%), and dry THF (1.0 mL). The reaction mixture was stirred for 2 minutes at room temperature and then phenyl methanesulfonate (34.4 mg, 0.2 mmol) was added. The microreactor was capped with a teflon pressure cap and placed into pre-heated aluminum block at 80 °C for 12 h. After 12 h GC-MS was checked and results are shown in the above scheme.

Meta-borylation of phenyl 4-methylbenzenesulfonate **(1j)**:

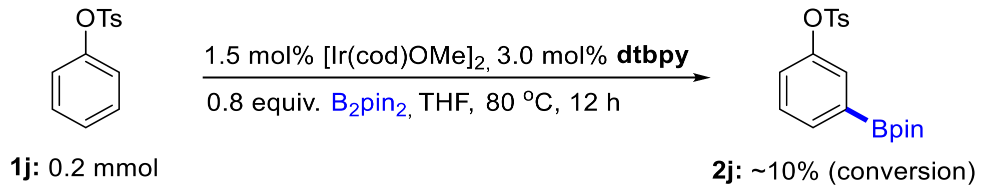

In an argon-filled glove box, a 5.0 mL Wheaton microreactor was charged with $[Ir(cod)OMe]_2$ (1.98 mg, 1.5 mol%), B_2pin_2 (40.6 mg, 0.8 equiv.), dtbpy (1.6 mg, 3.0 mol%), and dry THF (1.0 mL). The reaction mixture was stirred for 2 minutes at room temperature and then phenyl 4-methylbenzenesulfonate (49.6 mg, 0.2 mmol) was added. The microreactor was capped with a teflon pressure cap and placed into pre-heated aluminum block at 80 °C for 12 h. After 12 h GC-MS was checked and results are shown in the above scheme.

Meta-borylation of trimethyl(phenoxy)silane **(1k)**:

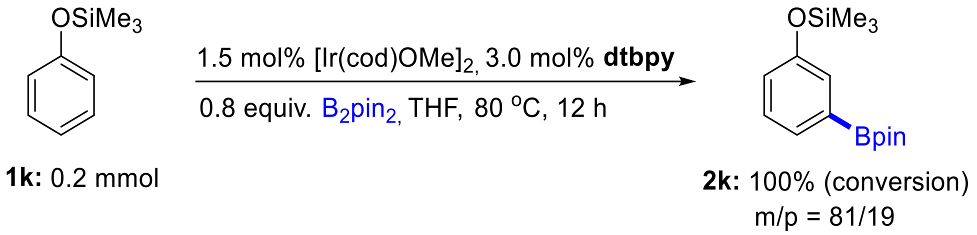

In an argon-filled glove box, a 5.0 mL Wheaton microreactor was charged with $[Ir(cod)OMe]_2$ (1.98 mg, 1.5 mol%), B_2pin_2 (40.6 mg, 0.8 equiv.), dtbpy (1.6 mg, 3.0 mol%), and dry THF (1.0 mL). The reaction mixture was stirred for 2 minutes at room temperature and then phenyl trimethyl(phenoxy)silane (33.2 mg, 0.2 mmol) was added. The microreactor was capped with a teflon pressure cap and placed into pre-heated aluminum block at 80 ^{o}C for 12 h. After 12 h GC-MS was checked and results are shown in the above scheme.

Meta-borylation of triisopropyl(phenoxy)silane **(1l)**:

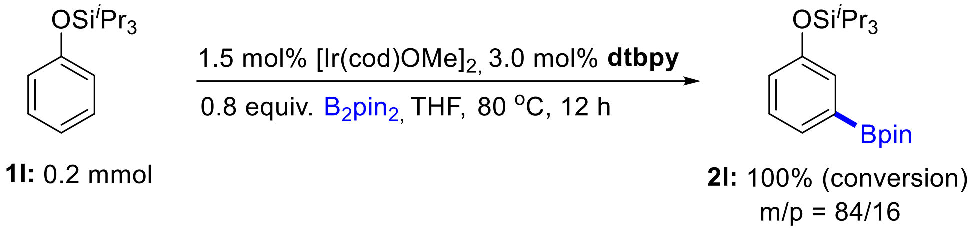

In an argon-filled glove box, a 5.0 mL Wheaton microreactor was charged with $[Ir(cod)OMe]_2$ (1.98 mg, 1.5 mol%), B_2pin_2 (40.6 mg, 0.8 equiv.), dtbpy (1.6 mg, 3.0 mol%), and dry THF (1.0 mL). The reaction mixture was stirred for 2 minutes at room temperature and then triisopropyl(phenoxy)silane (50.0 mg, 0.2 mmol) was added. The microreactor was capped with a teflon pressure cap and placed into pre-heated aluminum block at 80 ^{o}C for 12 h. After 12 h GC-MS was checked and results are shown in the above scheme.

ii) Ligand screening for borylation

General procedure[a]:

In an argon-filled glove box, a 5.0 mL Wheaton microreactor was charged with $[Ir(cod)OMe]_2$ (1.98 mg, 1.5 mol%), B_2pin_2 (40.6 mg, 0.8 equiv.), ligand **L1** (1.0 mg, 3.0 mol%), **L2** (1.1 mg, 3.0 mol%), **L3** (1.1 mg, 3.0 mol%), **L4** (1.1 mg, 3.0 mol%), **L5** (1.1 mg, 3.0 mol%), **L6** (1.1 mg, 3.0 mol%), **L7** (1.1 mg, 3.0 mol%), **L8** (1.1 mg, 3.0 mol%), **L9** (1.4 mg, 3.0 mol%), **L11** (1.1 mg, 3.0 mol%), **L12** (1.1 mg, 3.0 mol%), **L13** (1.2 mg, 3.0 mol%), **L14** (1.6 mg, 3.0 mol%), **L15** (2.3 mg,

3.0 mol%) and dry cyclohexane (1.0 mL). The reaction mixture was stirred for 2 minutes at room temperature and then triisopropyl(phenoxy)silane (50 mg, 0.2 mmol) was added. The microreactor was capped with a teflon pressure cap and placed into pre-heated aluminum block at 40 °C for 24 h. After 24 h, 10.0 µL of aliquot was withdrawn and conversion, isomeric ratio was checked by gas chromatography (GC-MS). Results are summarized in the figure.

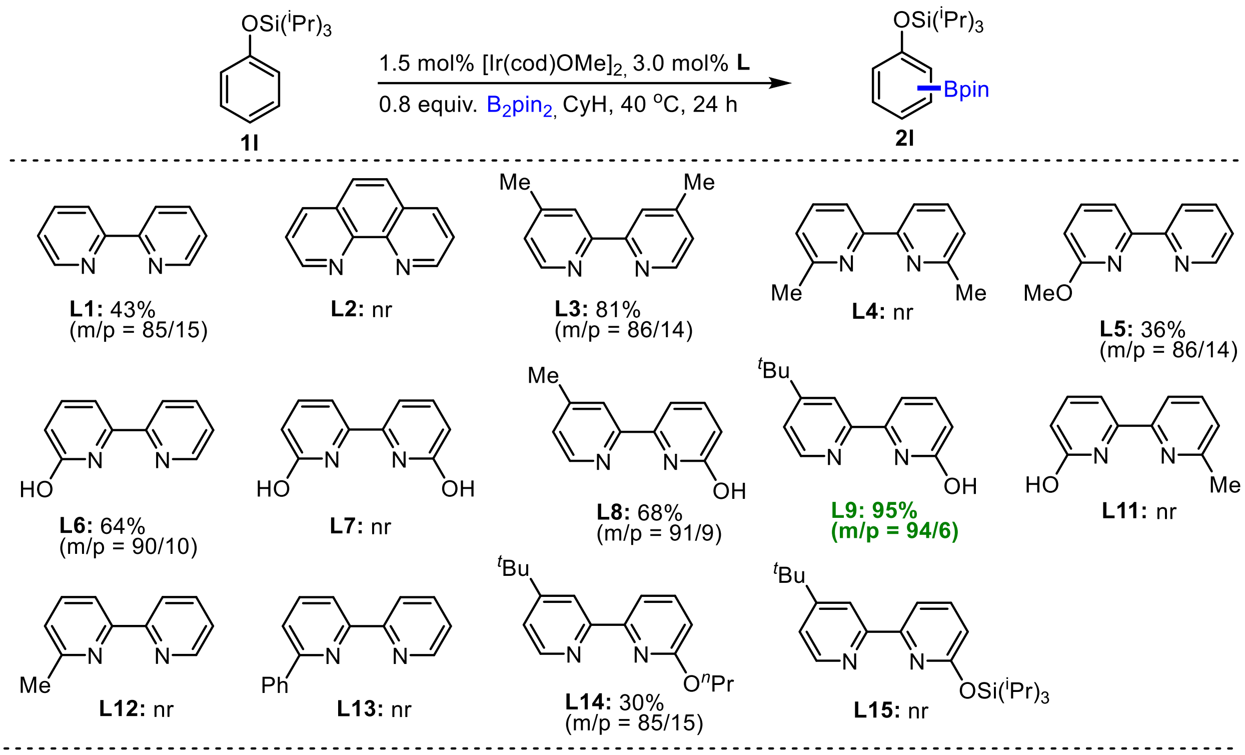

Supplementary Fig. 1: [a]Reactions were performed with 0.2 mmol substrate **1l**. Selectivity is based on the GC-MS analysis of the reaction. GC-MS conversion was measured using dodecane as an internal standard.

iii) Deleterious result with other silyl group

Meta-borylation of trimethyl(phenoxy)silane **(1k)**:

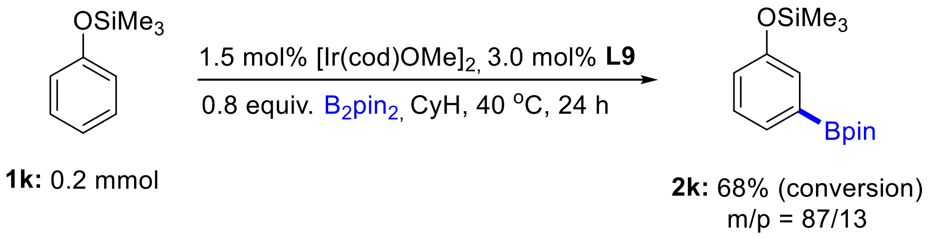

In an argon-filled glove box, a 5.0 mL Wheaton microreactor was charged with $[Ir(cod)OMe]_2$ (1.98 mg, 1.5 mol%), B_2pin_2 (40.6 mg, 0.8 equiv.), ligand **L9** (1.4 mg, 3.0 mol%), and dry

cyclohexane (1.0 mL). The reaction mixture was stirred for 2 minutes at room temperature and then trimethyl(phenoxy)silane (33.2 mg, 0.2 mmol) was added. The microreactor was capped with a teflon pressure cap and placed into pre-heated aluminum block at 40 ^{o}C for 24 h. After 24 h GC-MS was checked and results are shown in the above scheme.

Meta-borylation of triethyl(phenoxy)silane **(1m)***:*

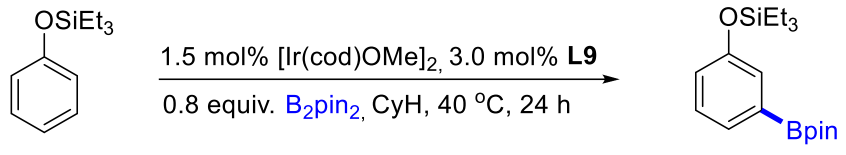

1m: 0.2 mmol

2m: 70% (conversion)
m/p = 81/19

In an argon-filled glove box, a 5.0 mL Wheaton microreactor was charged with $[Ir(cod)OMe]_2$ (1.98 mg, 1.5 mol%), B_2pin_2 (40.6 mg, 0.8 equiv.), ligand **L9** (1.4 mg, 3.0 mol%), and dry cyclohexane (1.0 mL). The reaction mixture was stirred for 2 minutes at room temperature and then triethyl(phenoxy)silane (41.6 mg, 0.2 mmol) was added. The microreactor was capped with a teflon pressure cap and placed into pre-heated aluminum block at 40 ^{o}C for 24 h. After 24 h GC-MS was checked and results are shown in the above scheme.

Meta-borylation of tert-butyldimethyl(phenoxy)silane **(1n)***:*

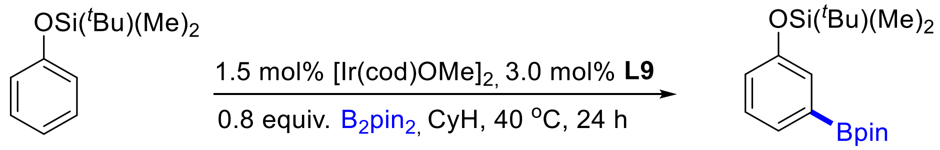

1n: 0.2 mmol

2n: 75% (conversion)
m/p = 88/12

In an argon-filled glove box, a 5.0 mL Wheaton microreactor was charged with $[Ir(cod)OMe]_2$ (1.98 mg, 1.5 mol%), B_2pin_2 (40.6 mg, 0.8 equiv.), ligand **L9** (1.4 mg, 3.0 mol%), and dry cyclohexane (1.0 mL). The reaction mixture was stirred for 2 minutes at room temperature and then tert-butyldimethyl(phenoxy)silane (41.6 mg, 0.2 mmol) was added. The microreactor was capped with a teflon pressure cap and placed into pre-heated aluminum block at 40 ^{o}C for 24 h. After 24 h GC-MS was checked and results are shown in the above scheme.

Meta-borylation of isopropyldimethyl(phenoxy)silane **(1o)***:*

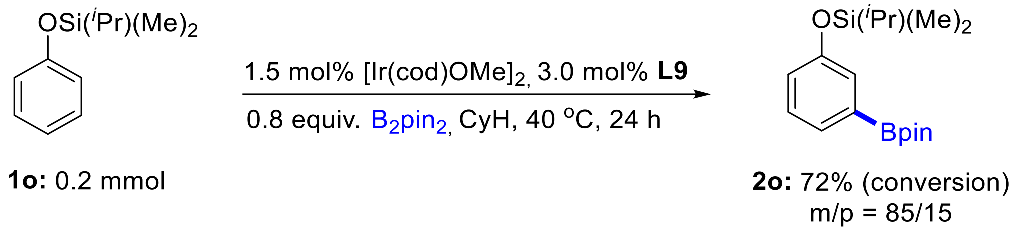

In an argon-filled glove box, a 5.0 mL Wheaton microreactor was charged with $[Ir(cod)OMe]_2$ (1.98 mg, 1.5 mol%), B_2pin_2 (40.6 mg, 0.8 equiv.), ligand **L9** (1.4 mg, 3.0 mol%), and dry cyclohexane (1.0 mL). The reaction mixture was stirred for 2 minutes at room temperature and then isopropyldimethyl(phenoxy)silane (38.8 mg, 0.2 mmol) was added. The microreactor was capped with a teflon pressure cap and placed into pre-heated aluminum block at 40 °C for 24 h. After 24 h GC-MS was checked and results are shown in the above scheme.

Meta-borylation of dimethyl(3-methylbutan-2-yl)(phenoxy)silane **(1p)***:*

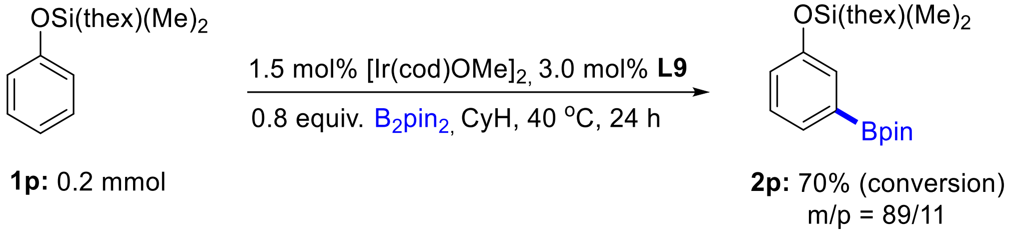

In an argon-filled glove box, a 5.0 mL Wheaton microreactor was charged with $[Ir(cod)OMe]_2$ (1.98 mg, 1.5 mol%), B_2pin_2 (40.6 mg, 0.8 equiv.), ligand **L9** (1.4 mg, 3.0 mol%), and dry cyclohexane (1.0 mL). The reaction mixture was stirred for 2 minutes at room temperature and then dimethyl(3-methylbutan-2-yl)(phenoxy)silane (44.4 mg, 0.2 mmol) was added. The microreactor was capped with a teflon pressure cap and placed into pre-heated aluminum block at 40 °C for 24 h. After 24 h GC-MS was checked and results are shown in the above scheme.

Meta-borylation of tributyl(phenoxy)silane **(1q)***:*

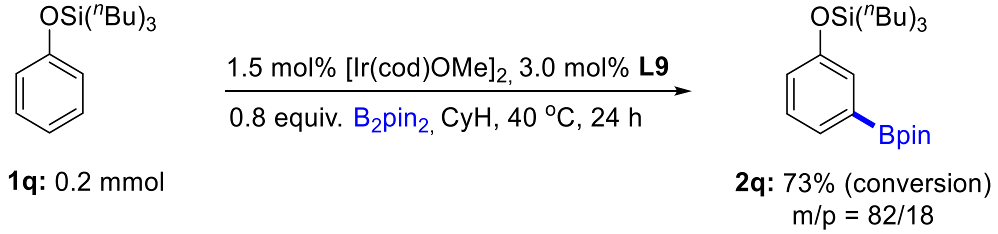

In an argon-filled glove box, a 5.0 mL Wheaton microreactor was charged with $[Ir(cod)OMe]_2$ (1.98 mg, 1.5 mol%), B_2pin_2 (40.6 mg, 0.8 equiv.), ligand **L9** (1.4 mg, 3.0 mol%), and dry cyclohexane (1.0 mL). The reaction mixture was stirred for 2 minutes at room temperature and then tributyl(phenoxy)silane (58.4 mg, 0.2 mmol) was added. The microreactor was capped with a teflon pressure cap and placed into pre-heated aluminum block at 40 ^{o}C for 24 h. After 24 h GC-MS was checked and results are shown in the above scheme.

iv) Importance of O–Si linkage towards meta selectivity :

Meta-borylation of benzyltriisopropylsilane **(1r)**:

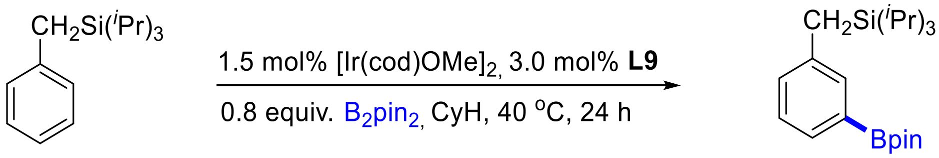

1r: 0.2 mmol

2r: 68% (conversion)
m/p = 77/23

In an argon-filled glove box, a 5.0 mL Wheaton microreactor was charged with $[Ir(cod)OMe]_2$ (1.98 mg, 1.5 mol%), B_2pin_2 (40.6 mg, 0.8 equiv.), ligand **L9** (1.4 mg, 3.0 mol%), and dry cyclohexane (1.0 mL). The reaction mixture was stirred for 2 minutes at room temperature and then benzyltriisopropylsilane (49.6 mg, 0.2 mmol) was added. The microreactor was capped with a teflon pressure cap and placed into pre-heated aluminum block at 40 ^{o}C for 24 h. After 24 h GC-MS was checked and results are shown in the above scheme. From the crude ^{1}H-NMR analysis we also calculated the isomer ratio which was in accordance with GC-MS ratio.

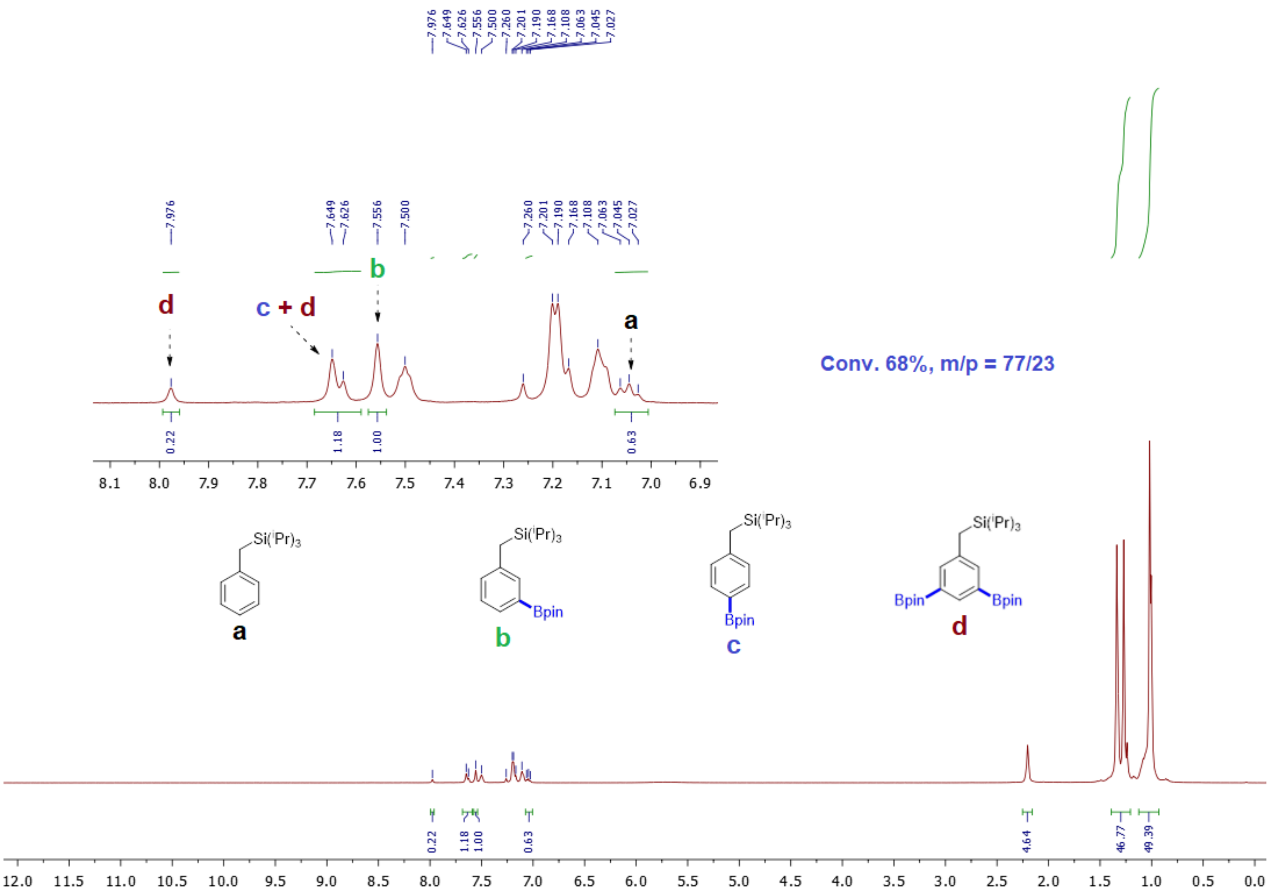

Supplementary Fig. 2: ^{1}H-NMR spectra of crude **2r** (25 °C, 400 MHz, $CDCl_3$)

Meta-borylation of benzyltrimethylsilane **(1s)***:*

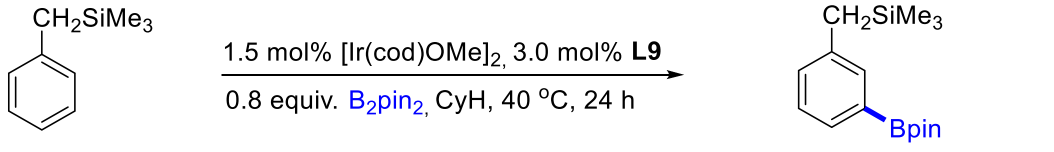

1s: 0.2 mmol

2s: 59% (GC-MS conversion)
m/p = 77/23

In an argon-filled glove box, a 5.0 mL Wheaton microreactor was charged with $[Ir(cod)OMe]_2$ (1.98 mg, 1.5 mol%), B_2pin_2 (40.6 mg, 0.8 equiv.), ligand **L9** (1.4 mg, 3.0 mol%), and dry cyclohexane (1.0 mL). The reaction mixture was stirred for 2 minutes at room temperature and then benzyltrimethylsilane (49.6 mg, 0.2 mmol) was added. The microreactor was capped with a teflon pressure cap and placed into pre-heated aluminum block at 40 °C for 24 h. After 24 h GC-MS was checked and results are shown in the above scheme. From the crude ^{1}H-NMR analysis we also calculated the isomer ratio which was in accordance with GC-MS ratio.

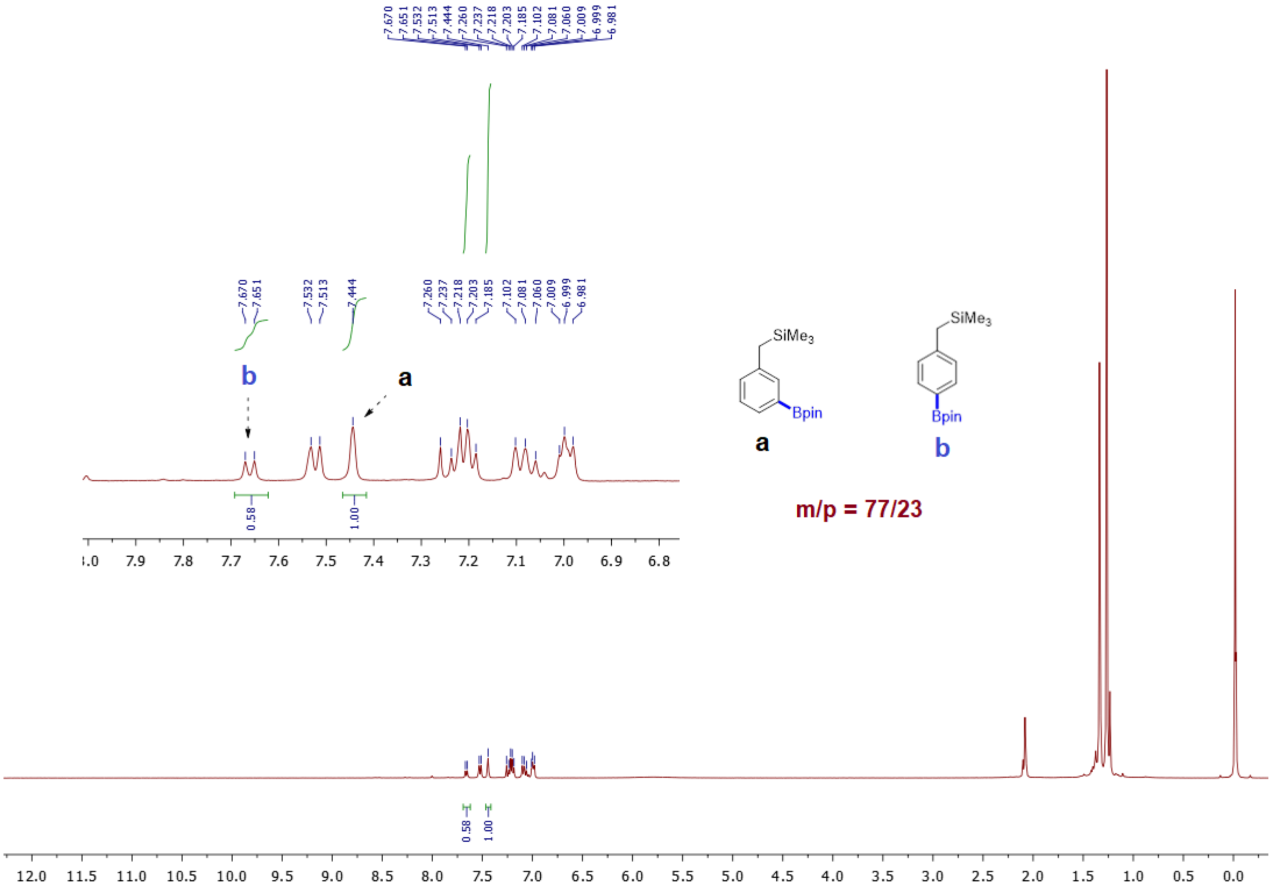

Supplementary Fig. 3: ^{1}H-NMR spectra of crude **2s** (25 °C, 400 MHz, $CDCl_3$)

v) Importance of [Si] towards meta selectivity :

Meta-borylation of tert-butoxybenzene **(1t)***:*

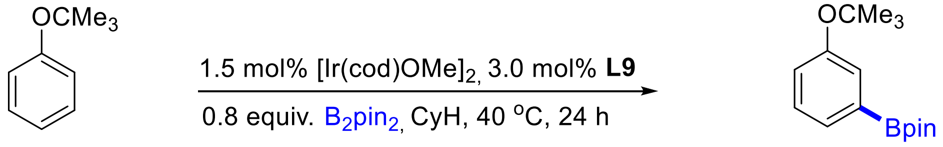

1t: 0.2 mmol

2t: 93% (GC-MS conversion)
m/p = 68/32

In an argon-filled glove box, a 5.0 mL Wheaton microreactor was charged with $[Ir(cod)OMe]_2$ (1.98 mg, 1.5 mol%), B_2pin_2 (40.6 mg, 0.8 equiv.), ligand **L9** (1.4 mg, 3.0 mol%), and dry cyclohexane (1.0 mL). The reaction mixture was stirred for 2 minutes at room temperature and then benzyltrimethylsilane (30 mg, 0.2 mmol) was added. The microreactor was capped with a

teflon pressure cap and placed into pre-heated aluminum block at 40 °C for 24 h. After 24 h GC-MS was checked and results are shown in the above scheme.

D. Preparation of Catalyst 3:

Synthesis of catalyst **3**:

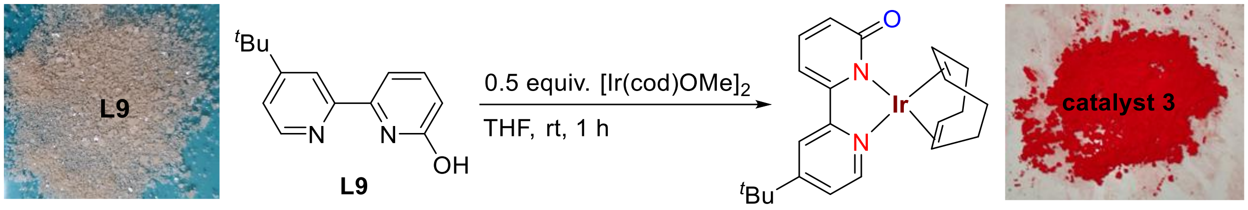

catalyst 3
90% (isolated yield)

In an argon filled glove box, a 5.0 mL wheaton microreactor was charged with **L9** ligand (114 mg, 0.5 mmol), $[Ir(cod)OMe]_2$ (165.7 mg, 0.5 equiv.), and dry THF (2.0 mL). Then the reaction mixture was stirred for 1 h at room temperature. After 1 h, 4 mL dry Hexane was used and a red colored precipitate was appeared and THF/Hexane mixture was decanted from the solid particle. After that the red precipitate was dried under vacuum to get 237 mg (90%) as powdered red colored catalyst. Crystallization was carried out in THF/Hexane solvent. The obtained product is air stable.

^{1}H NMR (400 MHz, $CDCl_3$): δ 7.82 (d, J = 1.6 Hz, 1H), 7.78 (d, J = 6.4 Hz, 1H), 7.28-7.22 (m, 2H), 6.74 (d, J = 6.8 Hz, 1H), 6.63 (d, J = 8.8 Hz, 1H), 5.60 (br, 2H), 3.61 (br, 2H), 2.41-2.38 (m, 2H), 2.27-2.23 (m, 2H), 2.03-1.97 (m, 2H), 1.71-1.65 (m, 2H), 1.36 (s, 9H).

^{13}C NMR (100 MHz, $CDCl_3$): δ 171.8, 163.7, 162.7, 154.2, 145.5, 136.6, 125.8, 121.8, 118.3, 105.2, 78.2, 62.6, 35.75, 33.7, 30.2, 30.0.

HRMS (ESI) *m/z* calcd for $C_{22}H_{28}IrN_2O$ $[M+H]^+$ 529.1831, found 529.1846.

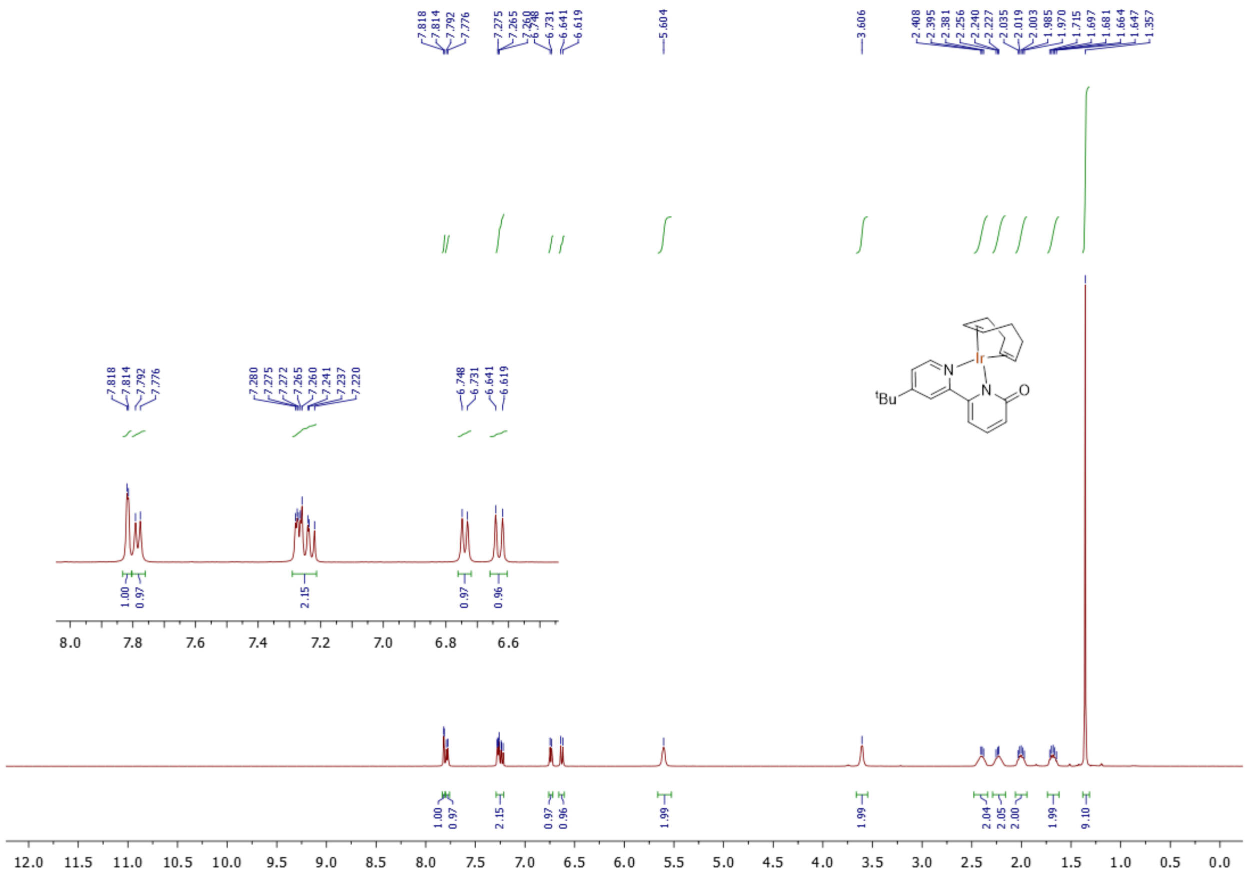

Supplementary Fig. 4: ^{1}H-NMR spectra of **catalyst 3** (25 ^{o}C, 400 MHz, $CDCl_3$)

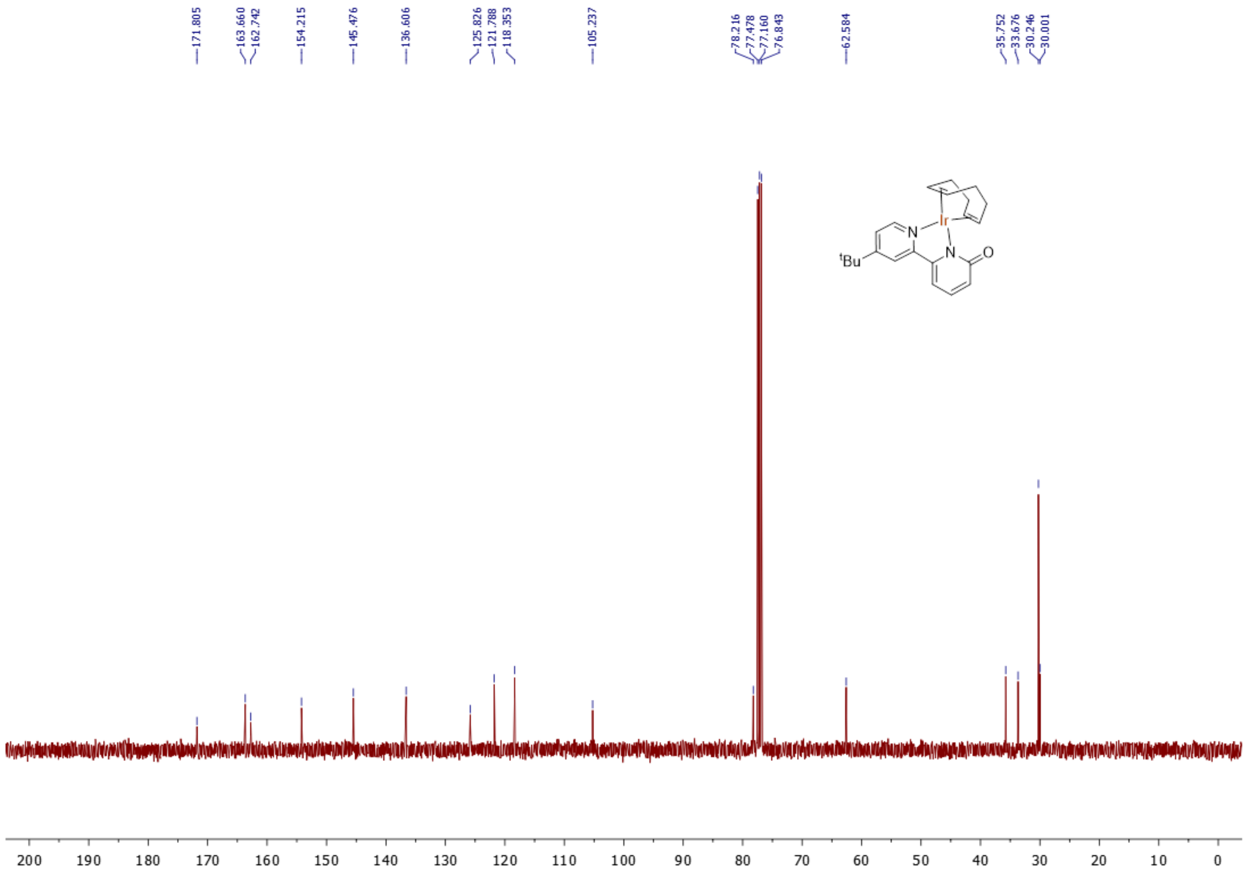

Supplementary Fig. 5: ^{13}C-NMR spectra of **catalyst 3** (25 °C, 400 MHz, $CDCl_3$)

X-Ray data of **catalyst 3**:

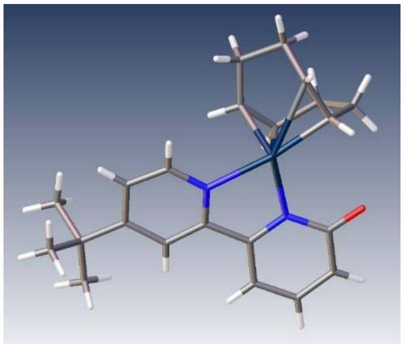

X-ray structure of **catalyst 3**

CCDC No. 2180880

checkCIF/PLATON report

Structure factors have been supplied for datablock(s) shelx

THIS REPORT IS FOR GUIDANCE ONLY. IF USED AS PART OF A REVIEW PROCEDURE FOR PUBLICATION, IT SHOULD NOT REPLACE THE EXPERTISE OF AN EXPERIENCED CRYSTALLOGRAPHIC REFEREE.

No syntax errors found. CIF dictionary Interpreting this report

Datablock: shelx

Bond precision: C-C = 0.0130 A Wavelength=0.71073

Cell:	a=10.8015(5)	b=10.8922(5)	c=11.9516(6)
	alpha=107.636(2)	beta=104.846(2)	gamma=105.135(2)
Temperature:	296 K		

	Calculated	Reported
Volume	1204.57(10)	1204.56(10)
Space group	P -1	P -1
Hall group	-P 1	-P 1
Moiety formula	C22 H27 Ir N2 O, C H2 Cl2, O	C22 H27 Ir N2 O , O , C H2 Cl2
Sum formula	C23 H29 Cl2 Ir N2 O2	C23 H29 Cl2 Ir N2 O2
Mr	628.60	628.58
Dx,g cm-3	1.733	1.733
Z	2	2
Mu (mm-1)	5.785	5.785
F000	616.0	616.0
F000'	613.99	
h,k,lmax	14,14,15	14,14,15
Nref	6039	6030
Tmin,Tmax		0.574,0.746
Tmin'		

Correction method= # Reported T Limits: Tmin=0.574 Tmax=0.746
AbsCorr = MULTI-SCAN

Data completeness= 0.999 Theta(max)= 28.374

R(reflections)= 0.0574(5536) wR2(reflections)= 0.1465(6030)

S = 1.090 Npar= 274

The following ALERTS were generated. Each ALERT has the format
test-name_ALERT_alert-type_alert-level.
Click on the hyperlinks for more details of the test.

Alert level A

```
PLAT094_ALERT_2_A Ratio of Maximum / Minimum Residual Density ....      12.93 Report
```

Author Response: This is due to heavy iridium metal and Q peak.

```
PLAT097_ALERT_2_A Large Reported Max.  (Positive) Residual Density      16.86 eA-3
```

Author Response: Residual density near the heavy iridium metal.

```
PLAT971_ALERT_2_A Check Calcd Resid. Dens.  1.39Ang From C16            17.69 eA-3
```

Author Response: Due to some disorder and residual density near heavy iridium metal.

Alert level B

```
PLAT306_ALERT_2_B Isolated Oxygen Atom (H-atoms Missing ?) .......          O Check
```

Author Response: Its a isolated atom.

```
PLAT971_ALERT_2_B Check Calcd Resid. Dens.  0.52Ang From N1              2.58 eA-3
```

Author Response: Due to some disorder and residual density near heavy iridium metal.

Alert level C

```
ABSTY02_ALERT_1_C  An _exptl_absorpt_correction_type has been given without
            a literature citation. This should be contained in the
            _exptl_absorpt_process_details field.
            Absorption correction given as multi-scan
DIFMX02_ALERT_1_C  The maximum difference density is > 0.1*ZMAX*0.75
            The relevant atom site should be identified.
PLAT042_ALERT_1_C Calc. and Reported MoietyFormula Strings  Differ     Please Check
PLAT053_ALERT_1_C Minimum Crystal Dimension Missing (or Error) ...     Please Check
PLAT054_ALERT_1_C Medium  Crystal Dimension Missing (or Error) ...     Please Check
PLAT055_ALERT_1_C Maximum Crystal Dimension Missing (or Error) ...     Please Check
PLAT342_ALERT_3_C Low Bond Precision on  C-C Bonds ..............    0.01305 Ang.
PLAT430_ALERT_2_C Short Inter D...A Contact  O        ..O1       .       2.88 Ang.
                                                  x,y,z  =      1_555 Check
PLAT430_ALERT_2_C Short Inter D...A Contact  O        ..O1       .       2.88 Ang.
                                             1-x,-y,1-z  =      2_656 Check
PLAT601_ALERT_2_C Unit Cell Contains Solvent Accessible VOIDS of .         49 Ang**3
PLAT971_ALERT_2_C Check Calcd Resid. Dens.  0.65Ang From C20             2.41 eA-3
```

Author Response: Due to some disorder and residual density near heavy iridium metal.

```
PLAT971_ALERT_2_C Check Calcd Resid. Dens.  1.24Ang From Ir            1.71 eA-3
```

Author Response: Due to some disorder and residual density near heavy iridium metal.

```
PLAT973_ALERT_2_C Check Calcd Positive Resid. Density on        Ir       1.11 eA-3
PLAT975_ALERT_2_C Check Calcd Resid. Dens.  0.97Ang From O       .       0.75 eA-3
PLAT975_ALERT_2_C Check Calcd Resid. Dens.  0.87Ang From O       .       0.67 eA-3
PLAT977_ALERT_2_C Check Negative Difference Density on H16       .      -0.95 eA-3
```

Alert level G

```
PLAT083_ALERT_2_G SHELXL Second Parameter in WGHT  Unusually Large      19.81 Why ?
PLAT154_ALERT_1_G The s.u.'s on the Cell Angles are Equal ..(Note)      0.002 Degree
PLAT432_ALERT_2_G Short Inter X...Y Contact  N2       ..C16      .       3.04 Ang.
                                        1-x,1-y,1-z  =      2_666 Check
PLAT720_ALERT_4_G Number of Unusual/Non-Standard Labels ..........          2 Note
PLAT767_ALERT_4_G INS Embedded LIST 6 Instruction Should be LIST 4     Please Check
PLAT790_ALERT_4_G Centre of Gravity not Within Unit Cell: Resd.  #          2 Note
          C H2 Cl2
PLAT793_ALERT_4_G Model has Chirality at C15         (Centro SPGR)          R Verify
PLAT883_ALERT_1_G No Info/Value for _atom_sites_solution_primary .     Please Do !
PLAT910_ALERT_3_G Missing # of FCF Reflection(s) Below Theta(Min).          1 Note
PLAT912_ALERT_4_G Missing # of FCF Reflections Above STh/L=  0.600          7 Note
PLAT941_ALERT_3_G Average HKL Measurement Multiplicity ..........         3.1 Low
PLAT965_ALERT_2_G The SHELXL WEIGHT Optimisation has not Converged     Please Check
PLAT978_ALERT_2_G Number C-C Bonds with Positive Residual Density.          2 Info
```

```
   3 ALERT level A = Most likely a serious problem - resolve or explain
   2 ALERT level B = A potentially serious problem, consider carefully
  16 ALERT level C = Check. Ensure it is not caused by an omission or oversight
  13 ALERT level G = General information/check it is not something unexpected

   8 ALERT type 1 CIF construction/syntax error, inconsistent or missing data
  18 ALERT type 2 Indicator that the structure model may be wrong or deficient
   3 ALERT type 3 Indicator that the structure quality may be low
   5 ALERT type 4 Improvement, methodology, query or suggestion
   0 ALERT type 5 Informative message, check
```

Datablock shelx - ellipsoid plot

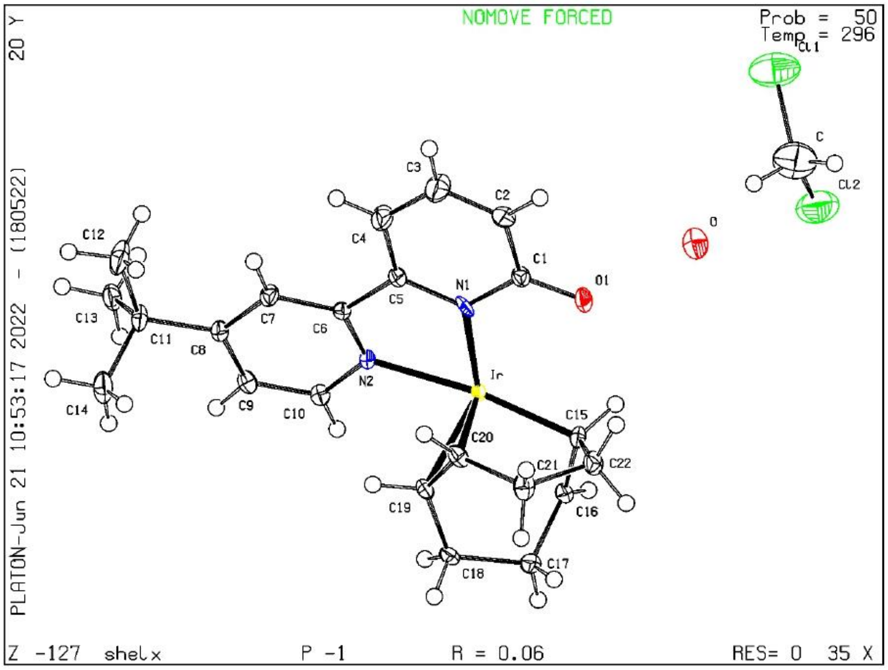

E. Reactivity test of catalyst 3:

Meta-borylation of triisopropyl(phenoxy)silane **(1l)** *with catalyst* **3***:*

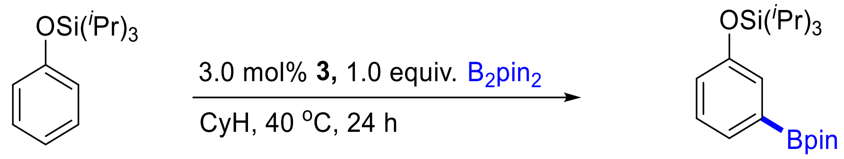

1l: 0.5 mmol

2l: 98% (GC-MS conversion)
m/p = 94/6

In an argon-filled glove box, a 5.0 mL Wheaton microreactor was charged with catalyst **3** (8 mg, 3.0 mol%), B_2pin_2 (127.0 mg, 1.0 equiv.) and dry cyclohexane (2.0 mL). The reaction mixture was stirred for 2 minutes at room temperature and then triisopropyl(phenoxy)silane (0.5 mmol, 125 mg) was added. The microreactor was capped with a teflon pressure cap and placed into pre-heated aluminum block at 40 °C and stirred for 24 h. After 24 h GC-MS was checked and results are shown in above scheme.

Meta-borylation of (2-chlorophenoxy)triisopropylsilane **(4a)** *with catalyst* **3**:

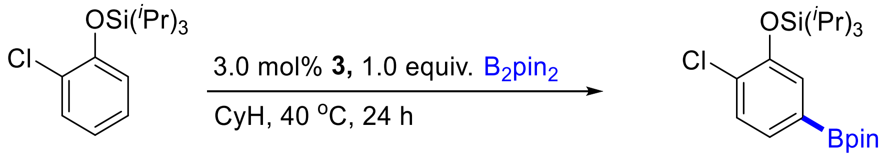

4a: 0.5 mmol

5a: 100% (GC-MS conversion)
m/p = 92/8

In an argon-filled glove box, a 5.0 mL Wheaton microreactor was charged with catalyst **3** (8 mg, 3.0 mol%), B_2pin_2 (127.0 mg, 1.0 equiv.) and dry cyclohexane (2.0 mL). The reaction mixture was stirred for 2 minutes at room temperature and then (2-chlorophenoxy)triisopropylsilane (0.5 mmol, 142 mg) was added. The microreactor was capped with a teflon pressure cap and placed into pre-heated aluminum block at 40 °C and stirred for 24 h. After 24 h GC-MS was checked and results are shown in above scheme.

Meta-borylation of (4-chlorophenoxy)triisopropylsilane **(6m)** *with catalyst* **3***:*

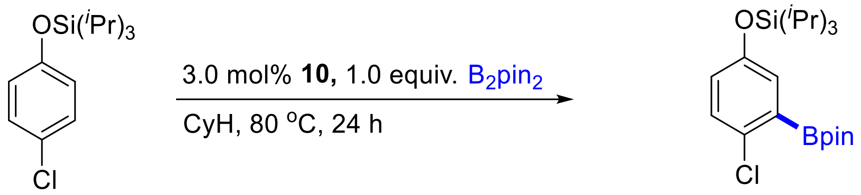

6m: 0.5 mmol

7m: 100% (GC-MS conversion)
m/others = 100/0

In an argon-filled glove box, a 5.0 mL Wheaton microreactor was charged with catalyst **3** (8 mg, 3.0 mol%), B_2pin_2 (127.0 mg, 1.0 equiv.) and dry cyclohexane (2.0 mL). The reaction mixture was stirred for 2 minutes at room temperature and then (4-chlorophenoxy)triisopropylsilane (0.5 mmol, 142 mg) was added. The microreactor was capped with a teflon pressure cap and placed into pre-heated aluminum block at 80 °C and stirred for 24 h. After 24 h GC-MS was checked and results are shown in above scheme.

F. Reactivity of catalyst 3 after kept in air for 30 days:

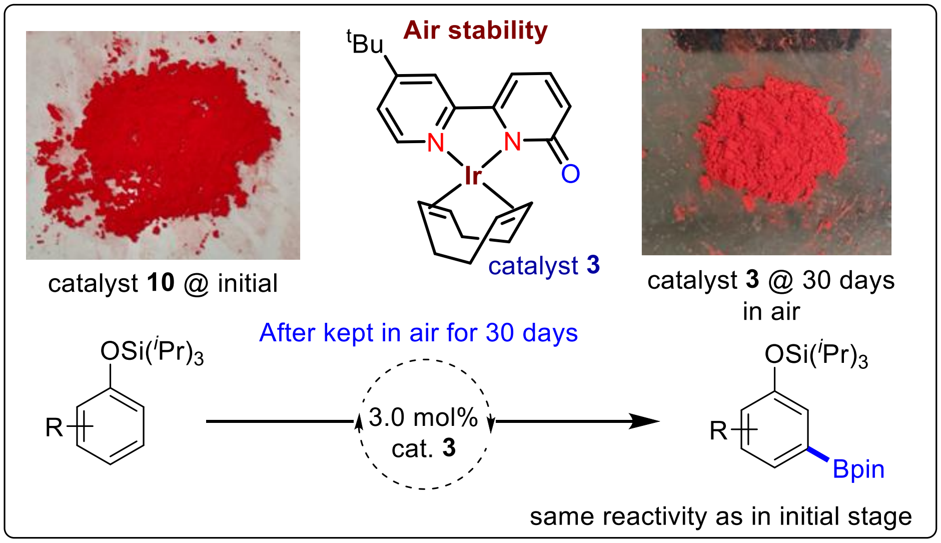

Supplementary Fig. 6 Air stability of catalyst 3

***Meta*-borylation of with catalyst 3 after kept in air for 30 days:**

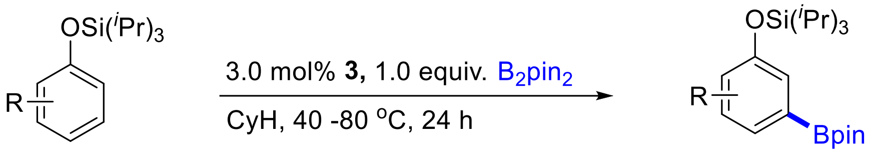

R = H, **2l:** 96%, m/p = 94/6
R = 2-Cl, **5a:** 100%, m/p 92/8
R = 4-Cl, **7m:** 100%, m = 100%
(GC-MS conversion)

In an argon-filled glove box, a 5.0 mL Wheaton microreactor was charged with catalyst **3 (**which was kept for 30 days in air**)** (8 mg, 3.0 mol%), B_2pin_2 (127.0 mg, 1.0 equiv.) and dry cyclohexane (2.0 mL). The reaction mixture was stirred for 2 minutes at room temperature and then substrates (1a or 1b or 3m) (0.5 mmol) was added. The microreactor was capped with a teflon pressure cap and placed into pre-heated aluminum block at 40-80 °C and stirred for 24 h. After 24 h GC-MS was checked and results are shown in above scheme.

G. Preparation of L10:

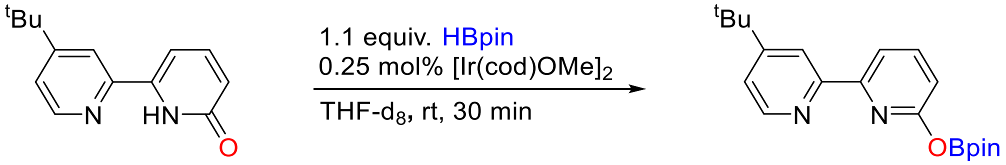

L9 **L10**

In an argon-filled glove box, a 5.0 mL Wheaton microreactor was charged with **L9** (22.8 mg, 0.1 mmol), HBpin (14.1 mg, 0.11 mmol, 1.1 equiv.), $[Ir(cod)OMe]_2$ (0.16 mg, 0.25 mol%) and 0.6 mL of THF-d8. Then, the reaction mixture was stirred for 30 minutes at room temperature under argon atmosphere and NMR was recorded. ^{1}H, ^{13}C and ^{11}B NMR analysis evidenced about the quantitative formation of **L10**.

^{1}H NMR (400 MHz, THF-d8) δ 8.51–8.49 (m, 2H), 8.23 (d, J = 7.6 Hz, 1H), 7.78 (t, J = 7.6 Hz, 1H), 7.32 (dd, J = 5.2, 1.6 Hz, 1H), 6.91 (d, J = 8.0 Hz, 1H), 1.38 (s, 9H), 1.34 (s, 12H).
^{13}C NMR (100 MHz, THF-d8) δ 161.3, 160.8, 156.5, 156.1, 150.1, 140.8, 121.6, 118.6, 117.0, 113.8, 84.3, 35.7, 31.1.
^{11}B NMR (128 MHz, THF-d8): δ 22.4.

H. Test of reactivity of L10:

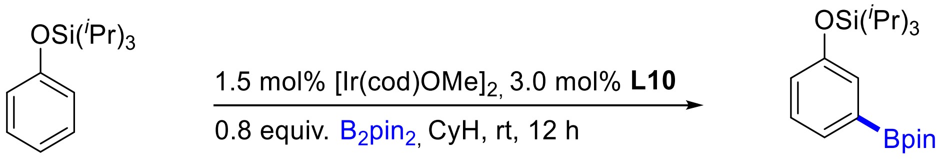

1l: 0.5 mmol

2l: 100% (conversion)
(m/p = 97/3)

In an argon-filled glove box, a 5.0 mL Wheaton microreactor was charged with $[Ir(cod)OMe]_2$ (4.97 mg, 1.5 mol%), B_2pin_2 (101.6 mg, 0.8 equiv.), **L10** (5.31 mg, 3.0 mol%), and dry cyclohexane (2.0 mL). The reaction mixture was stirred for 2 minutes at room temperature and then triisopropyl(phenoxy)silane (125 mg, 0.5 mmol) was added. The microreactor was capped with a teflon pressure cap and stirred for 12 h at room temperature. After 12 h, 10.0 µL of aliquot was withdrawn and GC-MS was checked. The GC-MS conversion and isomeric ratio are shown in the above scheme.

I. Improved reaction condition for the borylation reaction:

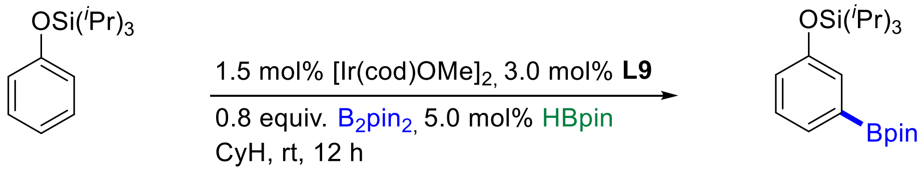

1l: 0.5 mmol

2l: conversion = 100%
(m/p = 97/3)
isolated yield = 91%

In an argon-filled glove box, a 5.0 mL Wheaton microreactor was charged with $[Ir(cod)OMe]_2$ (4.97 mg, 1.5 mol%), B_2pin_2 (101.6 mg, 0.8 equiv.), ligand **L9** (3.4 mg, 3.0 mol%), HBpin (3.2 mg, 5.0 mol%) and dry cyclohexane (2.0 mL). The reaction mixture was stirred for 2 minutes at room temperature and then triisopropyl(phenoxy)silane (125 mg, 0.5 mmol) was added. The microreactor was capped with a teflon pressure cap and stirred for 12 h at room temperature. After 12 h, 10.0 µL of aliquot was withdrawn and GC-MS was checked. The GC-MS conversion and isomeric ratio are shown in the above scheme. Then CyH was removed under reduced pressure and chromatographic separation with silica gel (2% EtOAc in hexane as eluent) gave 171 mg (91%) of the *meta*-borylated **(2l)** product as a colourless gummy liquid.

^{1}H NMR (400 MHz, $CDCl_3$): δ 7.38 (d, *J* = 7.6 Hz, 1H), 7.32 (d, *J* = 2.0 Hz, 1H), 7.22 (t, *J* = 7.6 Hz, 1H), 6.98 – 6.95 (m, 1H), 1.34 (s, 12H), 1.31 – 1.27 (m, 3H), 1.11 (d, *J* = 7.2 Hz, 18H).

^{13}C NMR (100 MHz, $CDCl_3$): δ 155.7, 128.9, 127.4, 126.1, 122.6, 83.8, 25.0, 18.1, 12.8.

^{11}B NMR (128 MHz, $CDCl_3$): δ 30.5.

HRMS (ESI) *m/z* calcd for $C_{21}H_{37}BO_3Si$ $[M+H]^+$ 377.2683, found 377.2682.

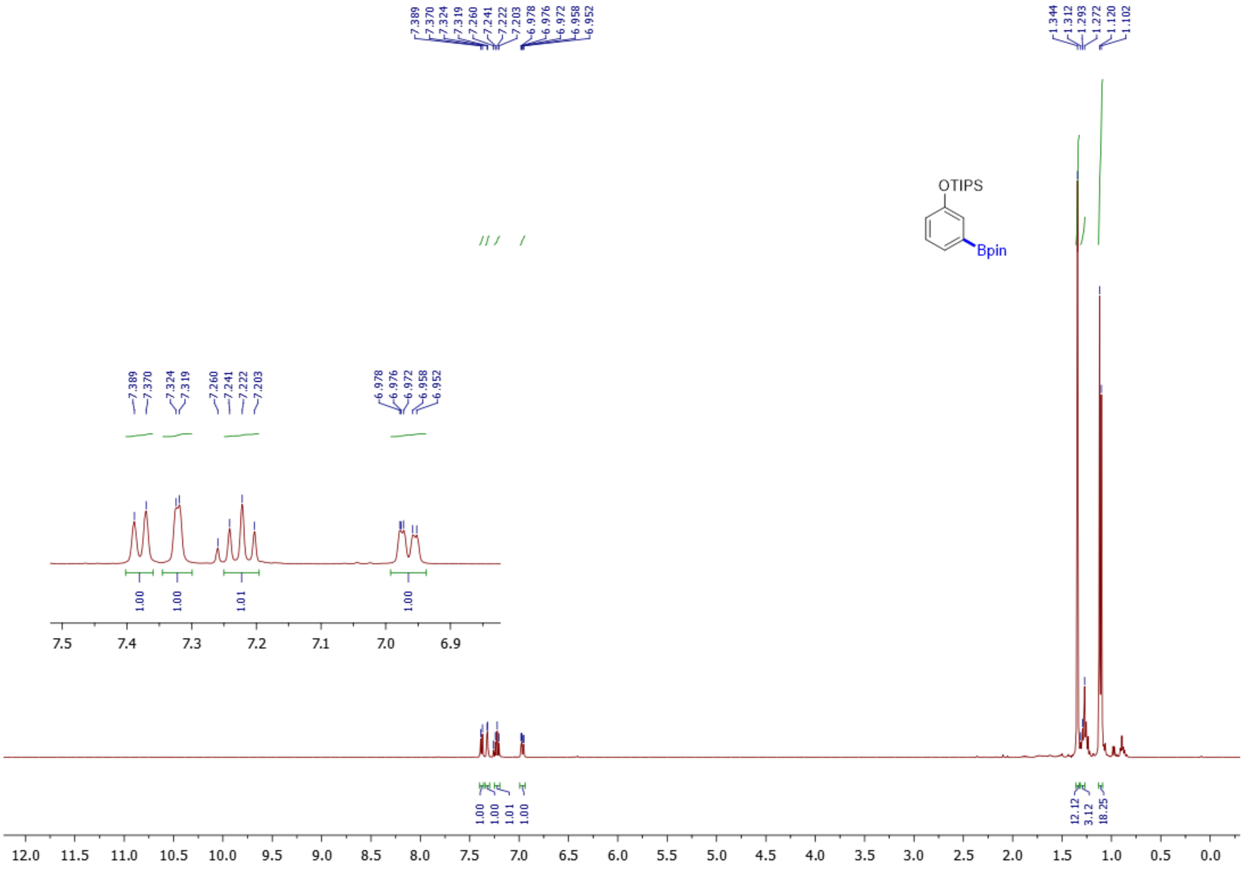

Supplementary Fig. 7: ^{1}H-NMR of meta borylated product

Authetic NMR data of ortho and para borylated products:

Ortho boylated product:

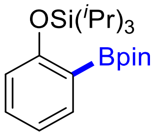

NMR data: ^{1}H NMR (400 MHz, $CDCl_3$): δ 7.60 (dd, J = 7.2, 1.2 Hz, 1H), 7.18 (dt, J = 8.0, 1.6 Hz, 1H), 6.81 (t, J = 7.2 Hz, 1H), 6.71 (d, J = 8.0 Hz, 1H), 1.27 – 1.21 (m, 15H), 1.04 (d, J = 7.2 Hz, 18H).

^{13}C NMR (100 MHz, $CDCl_3$): δ 161.3, 137.2, 132.3, 120.4, 119.0, 83.2, 25.0, 18.2, 13.4.

^{11}B NMR (128 MHz, $CDCl_3$): δ 30.7.

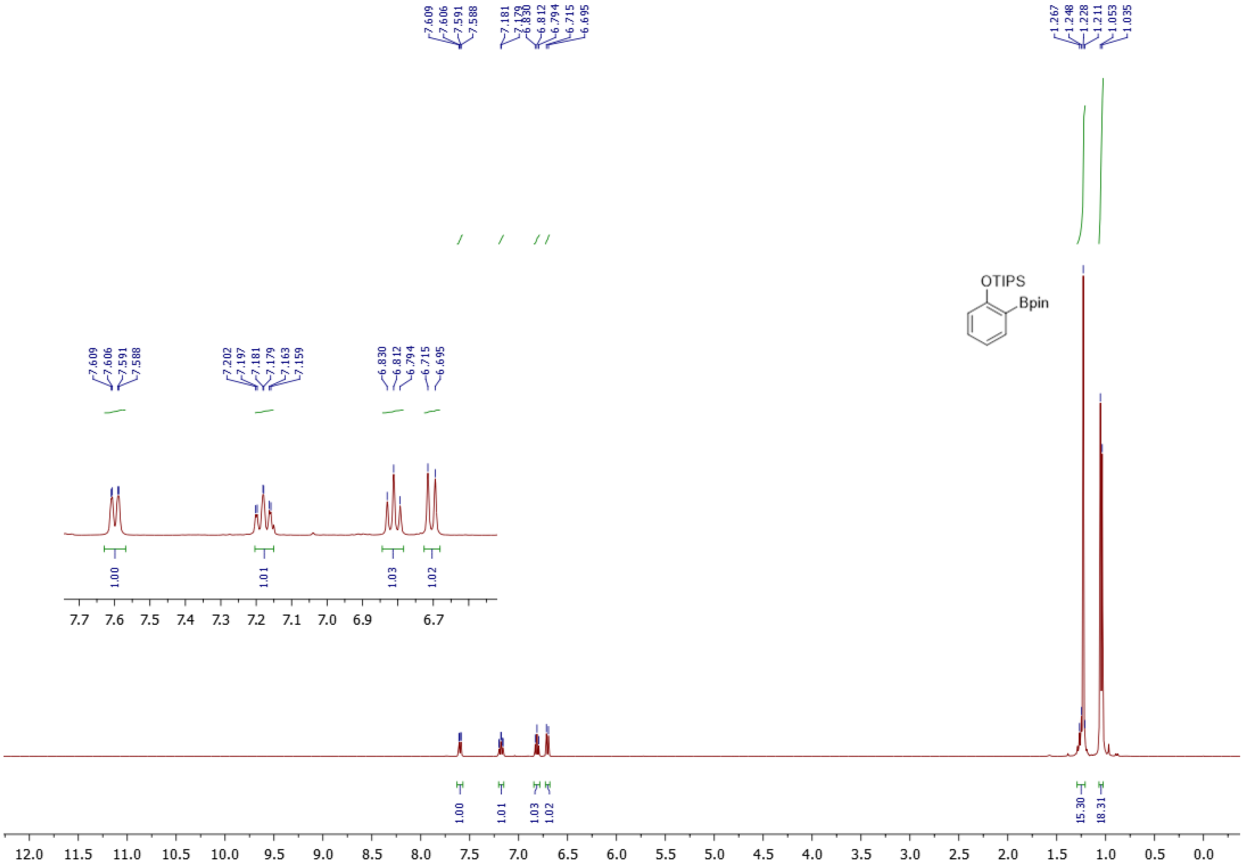

Supplementary Fig. 8: ^{1}H-NMR of ortho borylated product

Para borylated product:

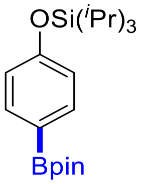

NMR data: ^{1}H NMR (400 MHz, $CDCl_3$): δ 7.69 (d, J = 8.4 Hz, 2H), 6.87 (d, J = 8.4 Hz, 2H), 1.33 (s, 12H), 1.29 – 1.23 (m, 3H), 1.10 (d, J = 7.2 Hz, 18H).

^{13}C NMR (100 MHz, $CDCl_3$): δ 159.1, 136.6, 119.5, 83.6, 25.0, 18.0, 12.8.

^{11}B NMR (128 MHz, $CDCl_3$): δ 30.7.

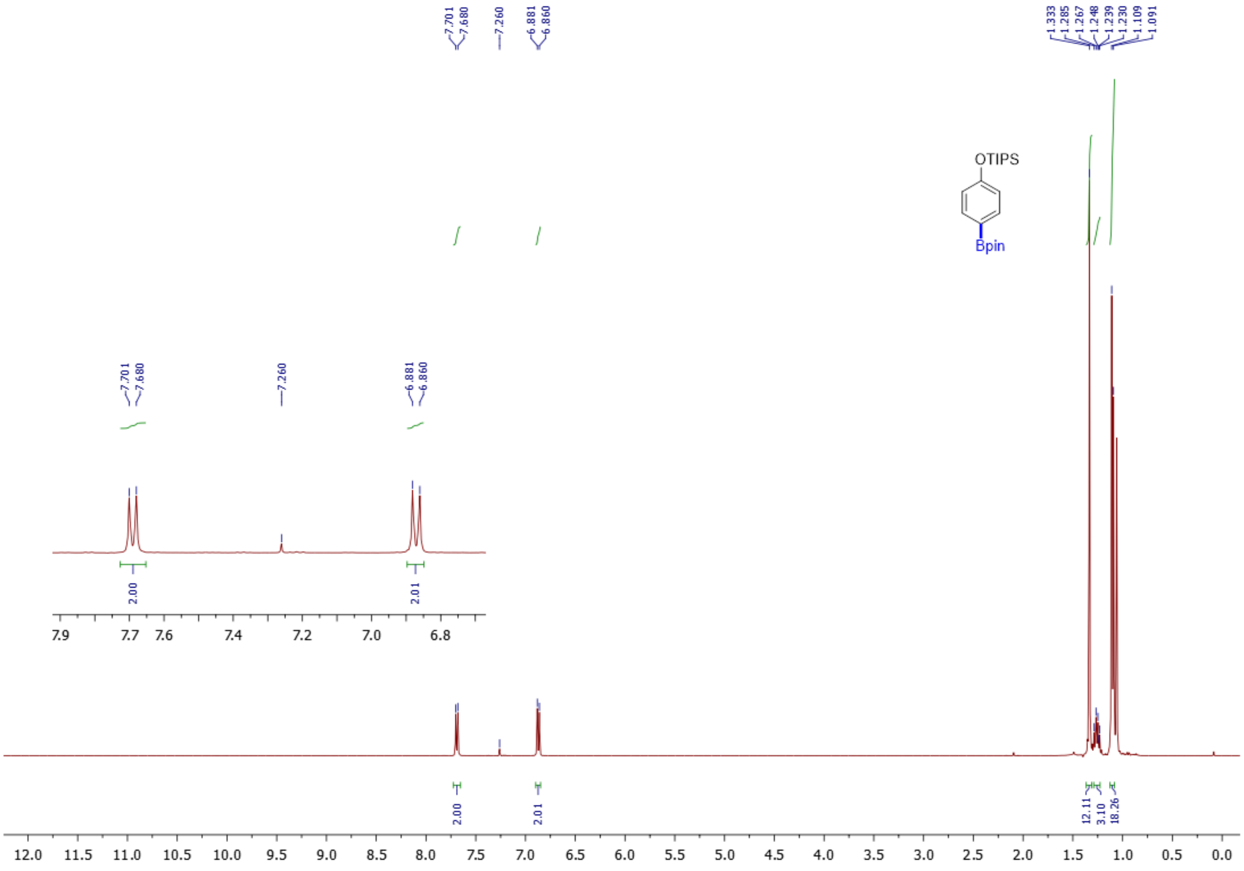

Supplementary Fig. 9: ^1H-NMR of para borylated product

J. Procedure for Meta-Borylation of Arenes(Het.):

General Procedure:

In an argon-filled glove box, a 5.0 mL Wheaton microreactor was charged with $[Ir(cod)OMe]_2$ (4.97 mg, 1.5 mol%), ligand **L9** (3.4 mg, 3.0 mol%), B_2pin_2 (127.0 mg, 1.0 equiv.), HBpin (3.2 mg, 5.0 mol%) and dry cyclohexane (2.0 mL) were added sequentially. The reaction mixture was stirred for 2 minutes at room temperature and then substrate (0.5 mmol) was added. The microreactor was capped with a Teflon pressure cap and stirred for 24 h at a particular mentioned temperature. After completion (judged by GC-MS), CyH was removed under reduced pressure and chromatographic separation with silica gel gave the *meta*-borylated product.

i) *Meta*-Borylation of Substituted Arenes:

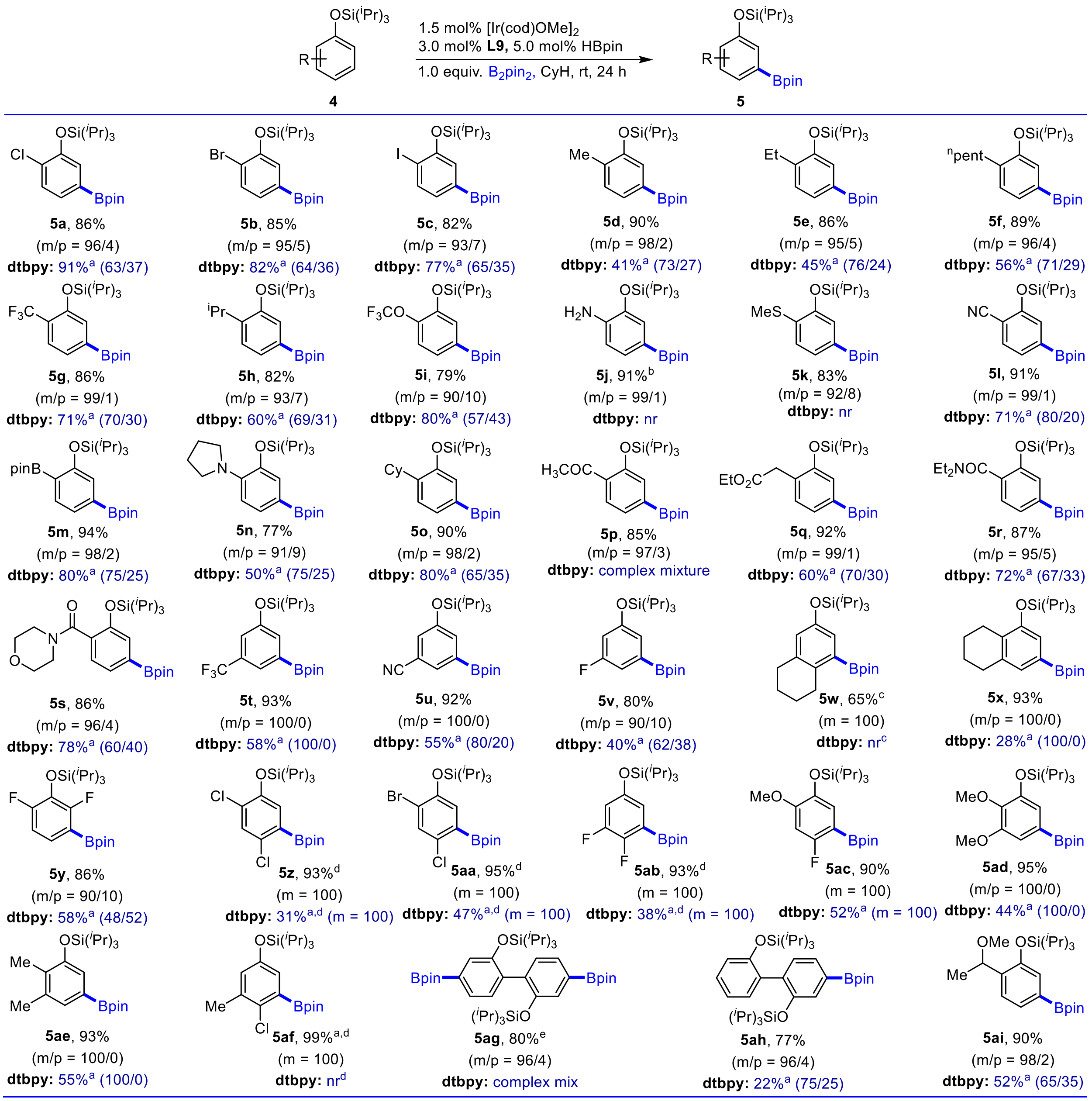

Supplementary Fig. 10: Substrates scope for substituted arenes. Reactions are in 0.5 mmol scale. [a]Conversion was reported. [b]1.5 equiv. B_2pin_2 was used. [c]Reaction is carried out at 80 °C. [d]Reactions are carried out at 50 °C. [e]2.0 equiv. B_2pin_2 was used.

Meta-borylation of (2-chlorophenoxy)triisopropylsilane **(4a)**:

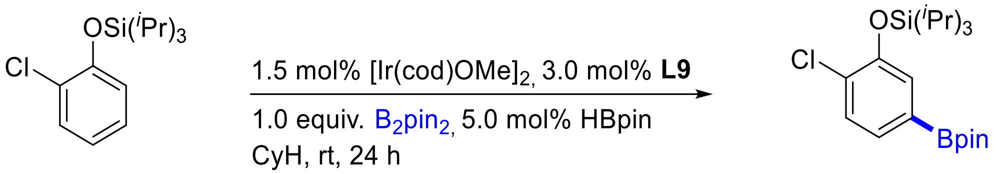

4a: 0.5 mmol

5a: 86% (isolated yield)
m/p = 96/4

In an argon-filled glove box, a 5.0 mL Wheaton microreactor was charged with $[Ir(cod)OMe]_2$ (4.97 mg, 1.5 mol%), B_2pin_2 (127.0 mg, 1.0 equiv.), ligand **L9** (3.4 mg, 3.0 mol%), HBpin (3.2 mg, 5.0 mol%) and dry cyclohexane (2.0 mL). The reaction mixture was stirred for 2 minutes at room temperature and then (2-chlorophenoxy)triisopropylsilane (0.5 mmol, 142 mg) was added. The microreactor was capped with a teflon pressure cap and stirred for 24 h at room temperature. After completion (judged by GC-MS), CyH was removed under reduced pressure and chromatographic separation with silica gel (3% EtOAc in hexane as eluent) gave 176 mg (86%) of the *meta*-borylated **(5a)** product as a colourless gummy liquid.

^{1}H NMR (400 MHz, $CDCl_3$): δ 7.30 (d, J = 8.0 Hz, 1H), 7.26 (s, 1H), 7.23 (s, 2H), 1.32 – 1.28 (m, 15H), 1.10 (d, J = 7.2 Hz, 18H).
^{13}C NMR (100 MHz, $CDCl_3$): δ 151.6, 129.9, 128.7, 128.1, 126.1, 84.0, 25.0, 18.1, 13.1.
^{11}B NMR (128 MHz, $CDCl_3$) δ 31.0.
HRMS (ESI) *m/z* calcd for $C_{21}H_{36}BClO_3Si$ $[M+H]^+$ 411.2294, found 411.2290.
Same reaction was carried out with dtbpy ligand under identical conditions.
Result: GC-MS Conversion = 91%; m/p = 63:37.
Same reaction was carried out with L6 under identical conditions.
Result: GC-MS Conversion = 88%; m/p = 90:10.

Meta-borylation of (2-chlorophenoxy)trimethylsilane **(4a')**:

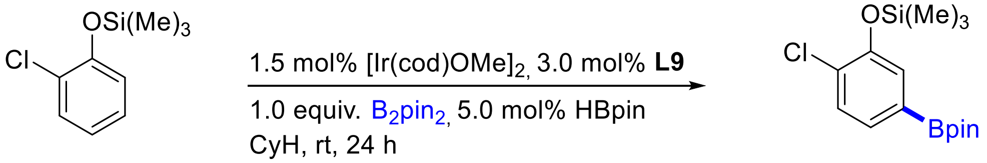

4a': 0.5 mmol

5a': 99% (GC-MS conversion)
m/p = 79/21

In an argon-filled glove box, a 5.0 mL Wheaton microreactor was charged with $[Ir(cod)OMe]_2$ (4.97 mg, 1.5 mol%), B_2pin_2 (127.0 mg, 1.0 equiv.), ligand **L9** (3.4 mg, 3.0 mol%), HBpin (3.2 mg, 5.0 mol%) and dry cyclohexane (2.0 mL). The reaction mixture was stirred for 2 minutes at room temperature and then (2-chlorophenoxy)trimethylsilane (0.5 mmol, 100 mg) was added. The microreactor was capped with a teflon pressure cap and stirred for 24 h at room temperature. After 24 h GC-MS was checked and results are shown in above scheme.

Meta-borylation of (2-bromophenoxy)triisopropylsilane **(4b)**:

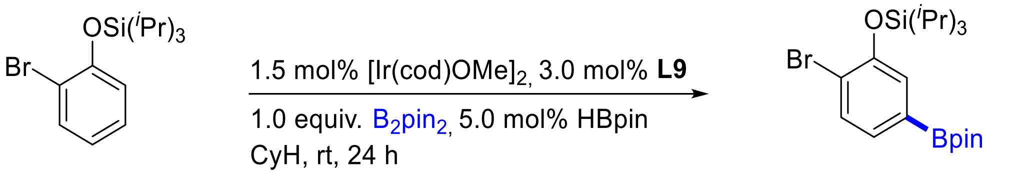

4b: 0.5 mmol

5b: 85% (isolated yield)
m/p = 95/5

In an argon-filled glove box, a 5.0 mL Wheaton microreactor was charged with $[Ir(cod)OMe]_2$ (4.97 mg, 1.5 mol%), B_2pin_2 (127.0 mg, 1.0 equiv.), ligand **L9** (3.4 mg, 3.0 mol%), HBpin (3.2 mg, 5.0 mol%) and dry cyclohexane (2.0 mL). The reaction mixture was stirred for 2 minutes at room temperature and then (2-bromophenoxy)triisopropylsilane (0.5 mmol, 164 mg) was added. The microreactor was capped with a teflon pressure cap and and stirred for 24 h at room temperature. After completion (judged by GC-MS), CyH was removed under reduced pressure and chromatographic separation with silica gel (2% EtOAc in hexane as eluent) gave 192 mg (85%) of the *meta*-borylated **(5b)** product as a colourless gummy liquid.

^{1}H NMR (400 MHz, $CDCl_3$): δ 7.50 (d, J = 8.0 Hz, 1H), 7.27 (d, J = 1.2 Hz, 1H), 7.19 (dd, J = 7.6, 1.2 Hz, 1H), 1.34 – 1.30 (m, 15H), 1.13 (d, J = 7.6 Hz, 18H).
^{13}C NMR (101 MHz, $CDCl_3$) δ 152.6, 133.1, 128.2, 125.6, 118.9, 84.0, 25.0, 18.2, 13.1.
^{11}B NMR (128 MHz, $CDCl_3$): δ 30.9.
HRMS (ESI) *m/z* calcd for $C_{21}H_{36}BBrO_3Si$ $[M+H]^+$ 455.1788, found 455.1785.
Same reaction was carried out with dtbpy ligand under identical conditions.
Result: GC-MS Conversion = 82%; m/p = 64/36.

Meta-borylation of (2-iodophenoxy)triisopropylsilane **(4c)***:*

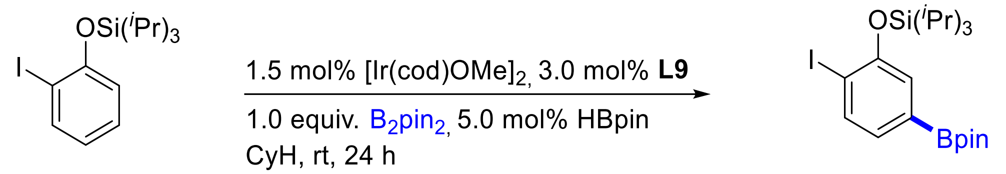

4c: 0.5 mmol

5c: 82% (isolated yield)
m/p = 93/7

In an argon-filled glove box, a 5.0 mL Wheaton microreactor was charged with $[Ir(cod)OMe]_2$ (4.97 mg, 1.5 mol%), B_2pin_2 (127.0 mg, 1.0 equiv.), ligand **L9** (3.4 mg, 3.0 mol%), HBpin (3.2 mg, 5.0 mol%) and dry cyclohexane (2.0 mL). The reaction mixture was stirred for 2 minutes at room temperature and then (2-iodophenoxy)triisopropylsilane (0.5 mmol, 188 mg) was added. The microreactor was capped with a teflon pressure cap and stirred for 24 h at room temperature. After completion (judged by GC-MS), CyH was removed under reduced pressure and chromatographic separation with silica gel (3% EtOAc in hexane as eluent) gave 205 mg (82%) of the *meta*-borylated **(5c)** product as a colourless gummy liquid.

^{1}H NMR (400 MHz, $CDCl_3$): δ 7.76 (d, J = 7.6 Hz, 1H), 7.21 (s, 1H), 7.04 (d, J = 7.6 Hz, 1H), 1.34 – 1.32 (m, 15H), 1.15 (d, J = 7.6 Hz, 18H).
^{13}C NMR (100 MHz, $CDCl_3$): δ 155.2, 139.3, 128.5, 123.9, 94.8, 84.0, 25.0, 18.3, 13.2.
^{11}B NMR (128 MHz, $CDCl_3$): δ 31.3.
HRMS (ESI) *m/z* calcd for $C_{21}H_{36}BIO_3Si$ $[M+H]^+$ 503.1650, found 503.1650.
Same reaction was carried out with dtbpy ligand under identical conditions.
Result: GC-MS Conversion = 77%; m/p = 65:35.

Meta-borylation of triisopropyl(o-tolyloxy)silane **(4d)***:*

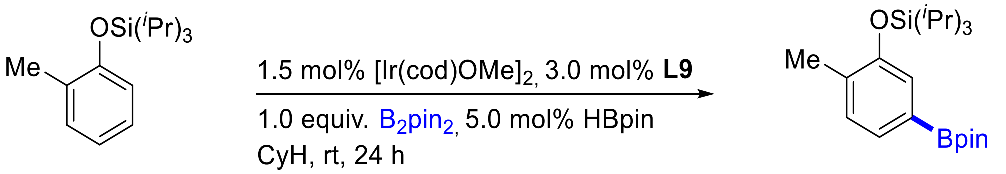

4d: 0.5 mmol

5d: 90% (isolated yield)
m/p = 98/2

In an argon-filled glove box, a 5.0 mL Wheaton microreactor was charged with $[Ir(cod)OMe]_2$ (4.97 mg, 1.5 mol%), B_2pin_2 (127.0 mg, 1.0 equiv.), ligand **L9** (3.4 mg, 3.0 mol%), HBpin (3.2

mg, 5.0 mol%) and dry cyclohexane (2.0 mL). The reaction mixture was stirred for 2 minutes at room temperature and then triisopropyl(o-tolyloxy)silane (0.5 mmol, 132 mg) was added. The microreactor was capped with a teflon pressure cap and stirred for 24 h at room temperature. After completion (judged by GC-MS), CyH was removed under reduced pressure and chromatographic separation with silica gel (3% EtOAc in hexane as eluent) gave 175 mg (90%) of the *meta*-borylated **(5d)** product as a colourless gummy liquid.

^{1}H NMR (400 MHz, CDCl$_3$): δ 7.28 (d, *J* = 7.2 Hz, 1H), 7.19 (s, 1H), 7.12 (d, *J* = 7.2 Hz, 1H), 2.26 (s, 3H), 1.34 – 1.30 (m, 15H), 1.12 (d, *J* = 7.2 Hz, 18H).
^{13}C NMR (100 MHz, CDCl$_3$): δ 154.0, 132.2, 130.6, 127.3, 124.0, 83.6, 25.0, 18.2, 17.5, 13.2.
^{11}B NMR (128 MHz, CDCl$_3$): δ 30.5.
HRMS (ESI) *m/z* calcd for $C_{22}H_{39}BO_3Si$ $[M+H]^+$ 391.2840, found 391.2834.
Same reaction was carried out with dtbpy ligand under identical conditions.
Result: GC-MS Conversion = 41%; m/p = 73:27.

Meta-borylation of (2-ethylphenoxy)triisopropylsilane **(4e)***:*

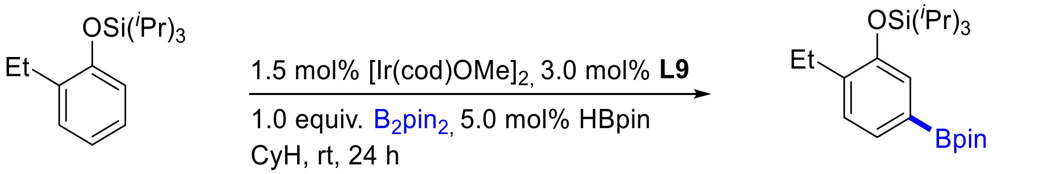

4e: 0.5 mmol

5e: 86% (isolated yield)
m/p = 95/5

In an argon-filled glove box, a 5.0 mL Wheaton microreactor was charged with $[Ir(cod)OMe]_2$ (4.97 mg, 1.5 mol%), B_2pin_2 (127.0 mg, 1.0 equiv.), ligand **L9** (3.4 mg, 3.0 mol%), HBpin (3.2 mg, 5.0 mol%) and dry cyclohexane (2.0 mL). The reaction mixture was stirred for 2 minutes at room temperature and then (2-ethylphenoxy)triisopropylsilane (0.5 mmol, 139 mg) was added. The microreactor was capped with a teflon pressure cap and stirred for 24 h at room temperature. After completion (judged by GC-MS), CyH was removed under reduced pressure and chromatographic separation with silica gel (2% EtOAc in hexane as eluent) gave 169 mg (84%) of the *meta*-borylated **(5e)** product as a colourless gummy liquid.

^{1}H NMR (400 MHz, CDCl$_3$): δ 7.32 (d, *J* = 7.6 Hz, 1H), 7.20 (s, 1H), 7.15 (d, *J* = 7.6 Hz, 1H), 2.67 (q, *J* = 7.6 Hz, 2H), 1.34 – 1.30 (m, 15H), 1.20 (d, *J* = 7.6 Hz, 3H), 1.13 (d, *J* = 7.2 Hz, 18H).

^{13}C NMR (100 MHz, $CDCl_3$): δ 153.5, 137.9, 128.9, 127.4, 123.9, 83.6, 25.0, 24.1, 18.3, 14.3, 13.2.

^{11}B NMR (128 MHz, $CDCl_3$): δ 31.0.

HRMS (ESI) *m/z* calcd for $C_{23}H_{41}BO_3Si$ $[M+Na]^+$ 427.2816, found 427.2817.

Same reaction was carried out with dtbpy ligand under identical conditions.

Result: GC-MS Conversion = 45%; m/p = 76:24.

Meta-borylation of triisopropyl(2-pentylphenoxy)silane **(4f)***:*

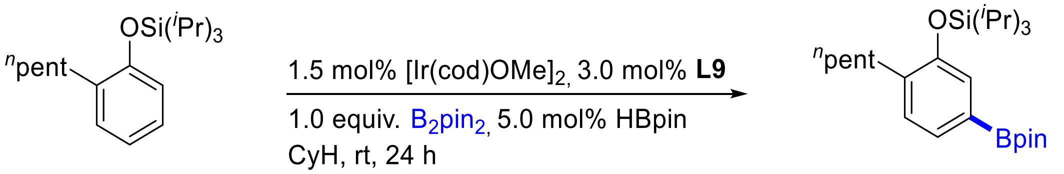

4f: 0.5 mmol

5f: 89% (isolated yield)
m/p = 96/4

In an argon-filled glove box, a 5.0 mL Wheaton microreactor was charged with $[Ir(cod)OMe]_2$ (4.97 mg, 1.5 mol%), B_2pin_2 (127.0 mg, 1.0 equiv.), ligand **L9** (3.4 mg, 3.0 mol%), HBpin (3.2 mg, 5.0 mol%) and dry cyclohexane (2.0 mL). The reaction mixture was stirred for 2 minutes at room temperature and then triisopropyl(2-pentylphenoxy)silane (0.5 mmol, 160 mg) was added. The microreactor was capped with a teflon pressure cap and stirred for 24 h at room temperature. After completion (judged by GC-MS), CyH was removed under reduced pressure and chromatographic separation with silica gel (3% EtOAc in hexane as eluent) gave 198 mg (89%) of the *meta*-borylated **(5f)** product as a colourless gummy liquid.

^1H NMR (400 MHz, $CDCl_3$): δ 7.29 (d, J = 7.2 Hz, 1H), 7.18 (s, 1H), 7.12 (d, J = 7.2 Hz, 1H), 2.62 (t, J = 7.6 Hz, 2H), 1.59 – 1.54 (m, 2H), 1.35 – 1.31 (m, 18H), 1.12 (d, J = 7.6 Hz, 18H), 0.88 (t, J = 7.2 Hz, 4H).

^{13}C NMR (100 MHz, $CDCl_3$): δ 153.5, 136.6, 129.8, 127.2, 123.9, 83.6, 32.1, 31.2, 29.8, 25.0, 22.8, 18.3, 14.2, 13.2.

^{11}B NMR (128 MHz, $CDCl_3$): δ 31.3.

HRMS (ESI) *m/z* calcd for $C_{26}H_{47}BO_3Si$ $[M+H]^+$ 447.3466, found 447.3470.

Same reaction was carried out with dtbpy ligand under identical conditions.

Result: GC-MS Conversion = 56%; m/p = 71:29.

Meta-borylation of triisopropyl(2-(trifluoromethyl)phenoxy)silane **(4g)**:

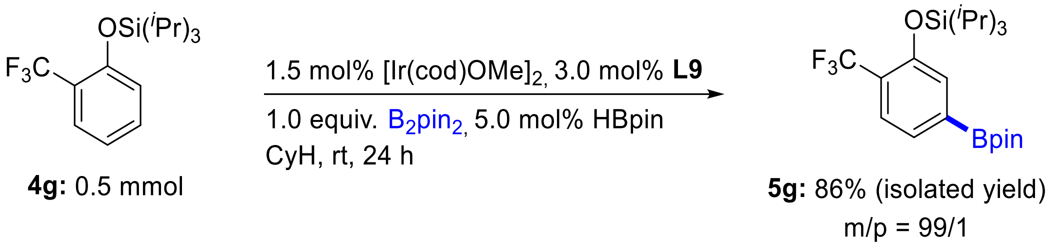

In an argon-filled glove box, a 5.0 mL Wheaton microreactor was charged with [Ir(cod)OMe]$_2$ (4.97 mg, 1.5 mol%), B$_2$pin$_2$ (127.0 mg, 1.0 equiv.), ligand **L9** (3.4 mg, 3.0 mol%), HBpin (3.2 mg, 5.0 mol%) and dry cyclohexane (2.0 mL). The reaction mixture was stirred for 2 minutes at room temperature and then triisopropyl(2-(trifluoromethyl)phenoxy)silane (0.5 mmol, 159 mg) was added. The microreactor was capped with a teflon pressure cap and stirred for 24 h at room temperature. After completion (judged by GC-MS), CyH was removed under reduced pressure and chromatographic separation with silica gel (3% EtOAc in hexane as eluent) gave 188 mg (85%) of the *meta*-borylated **(5g)** product as a colourless gummy liquid.

^{1}H NMR (400 MHz, CDCl$_3$): δ 7.52 (d, *J* = 8.0 Hz, 1H), 7.37 (d, *J* = 7.6 Hz, 1H), 7.29 (s, 1H), 1.39 – 1.31 (m, 15H), 1.13 (d, *J* = 7.2 Hz, 18H).
^{13}C NMR (100 MHz, CDCl$_3$): δ 153.8, 126.5 (q, *J* = 5.1 Hz), 126.2, 125.3, 122.8 (q, *J* = 29.6 Hz), 84.28, 25.0, 18.0, 13.1. (one peak in the aromatic region is missing)
^{11}B NMR (128 MHz, CDCl$_3$) δ 30.3.
HRMS (ESI) *m/z* calcd for $C_{22}H_{36}BF_3O_3Si$ [M+H]$^+$ 445.2557, found 445.2549.
Same reaction was carried out with dtbpy ligand under identical conditions.
Result: GC-MS Conversion = 71%; m/p = 70:30.

Meta-borylation of triisopropyl(2-isopropylphenoxy)silane **(4h)**:

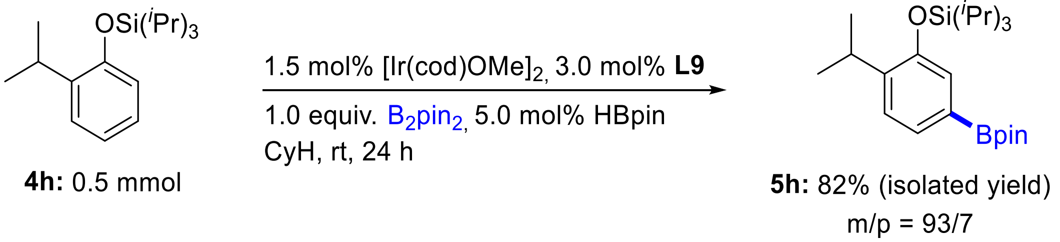

In an argon-filled glove box, a 5.0 mL Wheaton microreactor was charged with [Ir(cod)OMe]$_2$ (4.97 mg, 1.5 mol%), B_2pin_2 (127.0 mg, 1.0 equiv.), ligand **L9** (3.4 mg, 3.0 mol%), HBpin (3.2 mg, 5.0 mol%) and dry cyclohexane (2.0 mL). The reaction mixture was stirred for 2 minutes at room temperature and then triisopropyl(2-pentylphenoxy)silane (0.5 mmol, 146 mg) was added. The microreactor was capped with a teflon pressure cap and stirred for 24 h at room temperature. After completion (judged by GC-MS), CyH was removed under reduced pressure and chromatographic separation with silica gel (2% EtOAc in hexane as eluent) gave 167 mg (80%) of the *meta*-borylated **(5h)** product as a colourless gummy liquid.

^{1}H NMR (400 MHz, $CDCl_3$): δ 7.35 (d, J = 7.6 Hz, 1H), 7.20 (d, J = 6.8 Hz, 2H), 3.42 – 3.36 (m, 1H), 1.34 – 1.31 (m, 15H), 1.20 (d, J = 6.8 Hz, 6H), 1.13 (d, J = 7.6 Hz, 18H).

^{13}C NMR (100 MHz, $CDCl_3$): δ 145.2, 144.2, 128.8, 125.4, 114.9, 83.2, 50.2, 25.1, 25.0, 18.2, 13.3.

^{11}B NMR (128 MHz, $CDCl_3$): δ 31.7.

HRMS (ESI) *m/z* calcd for $C_{24}H_{43}BO_3Si$ $[M+H]^+$ 419.3153, found 419.3156.

Same reaction was carried out with dtbpy ligand under identical conditions.

Result: GC-MS Conversion = 60%; m/p = 69:31.

Meta-borylation of triisopropyl(2-(trifluoromethoxy)phenoxy)silane **(4i)***:*

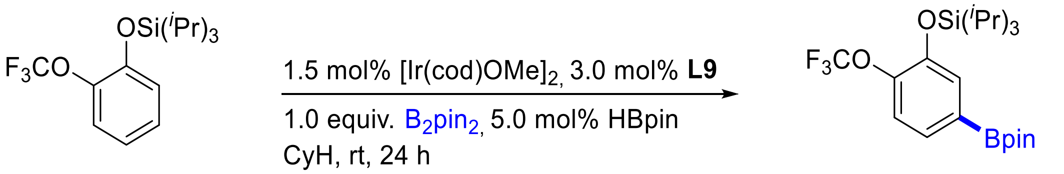

4i: 0.5 mmol

5i: 79% (isolated yield)
m/p = 90/10

In an argon-filled glove box, a 5.0 mL Wheaton microreactor was charged with [Ir(cod)OMe]$_2$ (4.97 mg, 1.5 mol%), B_2pin_2 (127.0 mg, 1.0 equiv.), ligand **L9** (3.4 mg, 3.0 mol%), HBpin (3.2 mg, 5.0 mol%) and dry cyclohexane (2.0 mL). The reaction mixture was stirred for 2 minutes at room temperature and then triisopropyl(2-(trifluoromethoxy)phenoxy)silane (0.5 mmol, 167 mg) was added. The microreactor was capped with a teflon pressure cap and stirred for 24 h at room temperature. After completion (judged by GC-MS), CyH was removed under reduced pressure and chromatographic separation with silica gel (2% EtOAc in hexane as eluent) gave 181 mg (79%) of the *meta*-borylated **(5i)** product as a colourless gummy liquid.

^{1}H NMR (400 MHz, $CDCl_3$): δ 7.36 – 7.35 (m, 2H), 7.20 (dd, *J* = 8.4, 1.2 Hz, 1H), 1.35 – 1.30 (m, 15H), 1.11 (d, *J* = 7.2 Hz, 18H).

^{13}C NMR (100 MHz, $CDCl_3$): δ 147.9, 142.5, 127.8, 127.2, 121.9, 120.8 (q, *J* = 255.9 Hz), 84.1, 25.0, 17.9, 12.9.

^{11}B NMR (128 MHz, $CDCl_3$): δ 30.9.

HRMS (ESI) *m/z* calcd for $C_{22}H_{36}BF_3O_4Si$ $[M+H]^+$ 461.2506, found 461.2506.

Same reaction was carried out with dtbpy ligand under identical conditions.

Result: GC-MS Conversion = 80%; m/p = 57:43.

Meta-borylation of 2-((triisopropylsilyl)oxy)aniline **(4j)**:

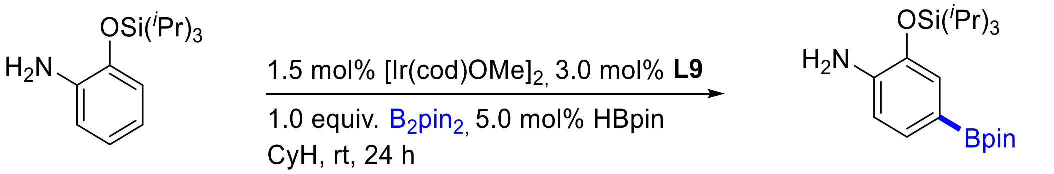

4j: 0.5 mmol

5j: 91% (isolated yield)
m/p = 99/1

In an argon-filled glove box, a 5.0 mL Wheaton microreactor was charged with $[Ir(cod)OMe]_2$ (4.97 mg, 1.5 mol%), B_2pin_2 (190.5 mg, 1.5 equiv.), ligand **L9** (3.4 mg, 3.0 mol%), HBpin (3.2 mg, 5.0 mol%) and dry cyclohexane (2.0 mL). The reaction mixture was stirred for 2 minutes at room temperature and then 2-((triisopropylsilyl)oxy)aniline (0.5 mmol, 132.5 mg) was added. The microreactor was capped with a teflon pressure cap and stirred for 24 h at room temperature. After completion (judged by GC-MS), CyH was removed under reduced pressure and chromatographic separation with silica gel (4% EtOAc in hexane as eluent) gave 178 mg (91%) of the *meta*-borylated **(5j)** product as a colourless gummy liquid.

^{1}H NMR (400 MHz, $CDCl_3$): δ 7.26 (d, *J* = 8.0 Hz,1H), 7.18 (s, 1H), 6.69 (d, *J* = 7.6 Hz,1H), 3.98 (s, 2H), 1.36 – 1.31 (m, 15H), 1.14 (d, *J* = 7.2 Hz, 18H).

^{13}C NMR (100 MHz, $CDCl_3$): δ 142.5, 141.1, 129.1, 123.9, 114.6, 83.2, 25.0, 18.2, 13.0.

^{11}B NMR (128 MHz, $CDCl_3$): δ 30.5.

HRMS (ESI) *m/z* calcd for $C_{21}H_{38}BNO_3Si$ $[M+H]^+$ 392.2792, found 392.2797.

Same reaction was carried out with dtbpy ligand under identical conditions.

Result: No reaction.

Meta-borylation of triisopropyl(2-(methylthio)phenoxy)silane **(4k)**:

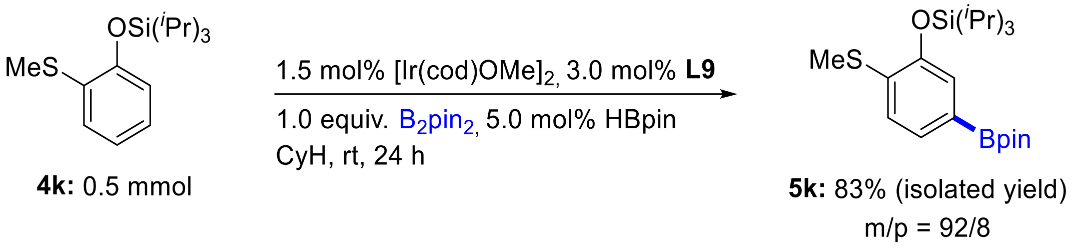

In an argon-filled glove box, a 5.0 mL Wheaton microreactor was charged with $[Ir(cod)OMe]_2$ (4.97 mg, 1.5 mol%), B_2pin_2 (127.0 mg, 1.0 equiv.), ligand **L9** (3.4 mg, 3.0 mol%), HBpin (3.2 mg, 5.0 mol%) and dry cyclohexane (2.0 mL). The reaction mixture was stirred for 2 minutes at room temperature and then triisopropyl(2-(methylthio)phenoxy)silane (0.5 mmol, 148 mg) was added. The microreactor was capped with a teflon pressure cap and stirred for 24 h at room temperature. After completion (judged by GC-MS), CyH was removed under reduced pressure and chromatographic separation with silica gel (2% EtOAc in hexane as eluent) gave 168 mg (80%) of the *meta*-borylated **(5k)** product as a colourless gummy liquid.

^{1}H NMR (400 MHz, $CDCl_3$): δ 7.36 (d, J = 2.8 Hz, 1H), 7.15 (s, 1H), 7.04 (d, J = 7.6 Hz, 1H), 2.40 (s, 3H), 1.36 – 1.32 (m, 15H), 1.13 (d, J = 7.6 Hz, 18H).

^{13}C NMR (100 MHz, $CDCl_3$): δ 151.8, 134.3, 128.0, 123.8, 123.1, 83.7, 25.0, 18.2, 14.1, 13.2.

^{11}B NMR (128 MHz, $CDCl_3$): δ 31.6.

HRMS (ESI) *m/z* calcd for $C_{22}H_{39}BO_3SSi$ $[M+H]^+$ 423.2560, found 423.2552.

Same reaction was carried out with dtbpy ligand under identical conditions.

Result: No reaction.

Meta-borylation of 2-((triisopropylsilyl)oxy)benzonitrile **(4l)**:

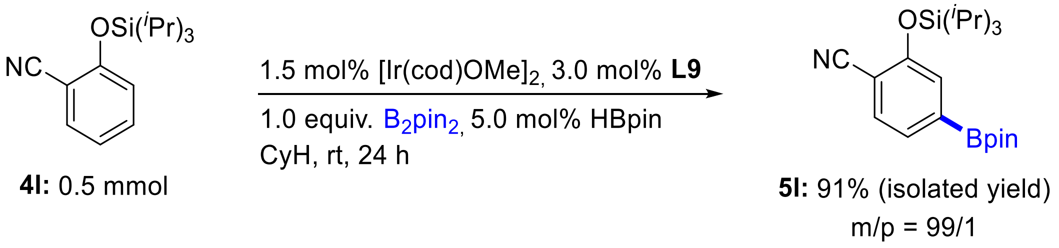

In an argon-filled glove box, a 5.0 mL Wheaton microreactor was charged with $[Ir(cod)OMe]_2$ (4.97 mg, 1.5 mol%), B_2pin_2 (127.0 mg, 1.0 equiv.), ligand **L9** (3.4 mg, 3.0 mol%), HBpin (3.2

mg, 5.0 mol%) and dry cyclohexane (2.0 mL). The reaction mixture was stirred for 2 minutes at room temperature and then 2-((triisopropylsilyl)oxy)benzonitrile (0.5 mmol, 137.5 mg) was added. The microreactor was capped with a teflon pressure cap and stirred for 24 h at room temperature. After completion (judged by GC-MS), CyH was removed under reduced pressure and chromatographic separation with silica gel (5% EtOAc in hexane as eluent) gave 182 mg (91%) of the *meta*-borylated **(5l)** product as a colourless gummy liquid.

^{1}H NMR (400 MHz, $CDCl_3$): δ 7.50 (d, J = 7.6 Hz, 1H), 7.37 (d, J = 7.6 Hz, 1H), 7.29 (s, 1H), 1.36 – 1.32 (m, 15H), 1.14 (d, J = 7.6 Hz, 18H).
^{13}C NMR (100 MHz, $CDCl_3$): δ 157.8, 132.9, 127.0, 125.2, 117.3, 107.2, 84.5, 25.0, 18.0, 13.0.
^{11}B NMR (128 MHz, $CDCl_3$): δ 31.2.
HRMS (ESI) *m/z* calcd for $C_{22}H_{36}BNO_3Si$ $[M+H]^+$ 402.2636, found 402.2636.
Same reaction was carried out with dtbpy ligand under identical conditions.
Result: GC-MS Conversion = 71%; m/p = 80:20.

Meta-borylation of triisopropyl(2-(4,4,5,5-tetramethyl-1,3,2-dioxaborolan-2-yl)phenoxy)silane **(4m)***:*

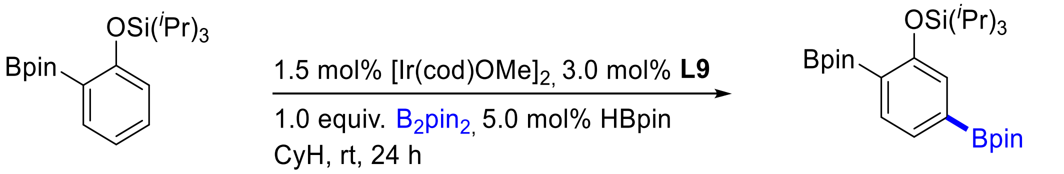

4m: 0.5 mmol

5m: 94% (isolated yield)
m/p = 98/2

In an argon-filled glove box, a 5.0 mL Wheaton microreactor was charged with $[Ir(cod)OMe]_2$ (4.97 mg, 1.5 mol%), B_2pin_2 (127.0 mg, 1.0 equiv.), ligand **L9** (3.4 mg, 3.0 mol%), HBpin (3.2 mg, 5.0 mol%) and dry cyclohexane (2.0 mL). The reaction mixture was stirred for 2 minutes at room temperature and then triisopropyl(2-(4,4,5,5-tetramethyl-1,3,2-dioxaborolan-2-yl)phenoxy)silane (0.5 mmol, 188 mg) was added. The microreactor was capped with a teflon pressure cap and stirred for 24 h at room temperature. After completion (judged by GC-MS), CyH was removed under reduced pressure and chromatographic separation with silica gel (5% EtOAc in hexane as eluent) gave 235 mg (94%) of the *meta*-borylated **(5m)** product as a colourless gummy liquid.

^{1}H NMR (400 MHz, $CDCl_3$): δ 7.65 (d, J = 7.2 Hz, 1H), 7.31 (d, J = 7.2 Hz, 1H), 7.19 (s, 1H), 1.34–1.31 (m, 27H), 1.13 (d, J = 7.6 Hz, 18H).

^{13}C NMR (100 MHz, $CDCl_3$): δ 160.6, 136.4, 126.4, 124.9, 83.8, 83.3, 25.0, 25.0, 18.3, 13.4.

^{11}B NMR (128 MHz, $CDCl_3$): δ 30.7.

HRMS (ESI) *m/z* calcd for $C_{27}H_{48}B_2O_5Si$ $[M+H]^+$ 503.3535, found 503.3544.

Same reaction was carried out with dtbpy ligand under identical conditions.

Result: GC-MS Conversion = 80%; m/p = 75:25.

Meta-borylation of 1-(2-((triisopropylsilyl)oxy)phenyl)pyrrolidine **(4n)**:

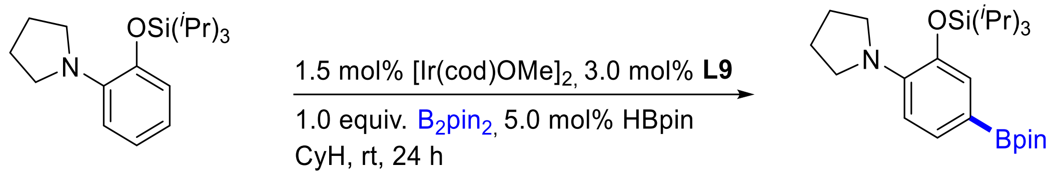

4n: 0.5 mmol

5n: 77% (isolated yield)
m/p = 91/9

In an argon-filled glove box, a 5.0 mL Wheaton microreactor was charged with $[Ir(cod)OMe]_2$ (4.97 mg, 1.5 mol%), B_2pin_2 (127.0 mg, 1.0 equiv.), ligand **L9** (3.4 mg, 3.0 mol%), HBpin (3.2 mg, 5.0 mol%) and dry cyclohexane (2.0 mL). The reaction mixture was stirred for 2 minutes at room temperature and then 1-(2-((triisopropylsilyl)oxy)phenyl)pyrrolidine (0.5 mmol, 159.5 mg) was added. The microreactor was capped with a teflon pressure cap and stirred for 24 h at room temperature. After completion (judged by GC-MS), CyH was removed under reduced pressure and chromatographic separation with silica gel (3% EtOAc in hexane as eluent) gave 167 mg (75%) of the *meta*-borylated **(5n)** product as a colourless gummy liquid.

^{1}H NMR (400 MHz, $CDCl_3$): δ 7.29 (dd, J = 8.0, 1.2 Hz, 1H), 7.18 (d, J = 1.2 Hz, 1H), 6.67 (d, J = 7.6 Hz, 1H), 3.35 (t, J = 6.4 Hz, 4H), 1.90 – 1.86 (m, 4H), 1.35 – 1.30 (m, 15H), 1.11 (d, J = 7.6 Hz, 18H).

^{13}C NMR (100 MHz, $CDCl_3$): δ 145.2, 144.2, 128.8, 125.4, 114.9, 83.2, 50.2, 25.1, 25.0, 18.2, 13.3.

^{11}B NMR (128 MHz, $CDCl_3$): δ 31.2.

HRMS (ESI) *m/z* calcd for $C_{25}H_{44}BNO_3Si$ $[M+H]^+$ 446.3262, found 446.3262.

Same reaction was carried out with dtbpy ligand under identical conditions.

Result: GC-MS Conversion = 50%; m/p = 75:25.

Meta-borylation of (2-cyclohexylphenoxy)triisopropylsilane **(4o)***:*

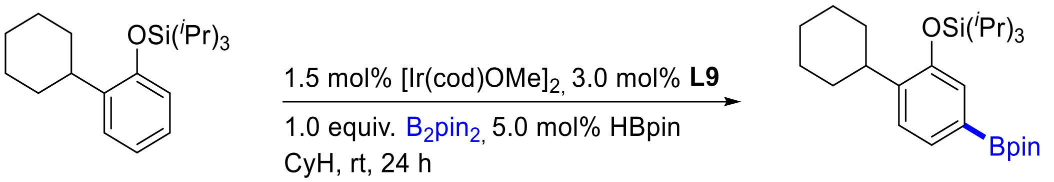

4o: 0.5 mmol

5o: 90% (isolated yield)
m/p = 98/2

In an argon-filled glove box, a 5.0 mL Wheaton microreactor was charged with $[Ir(cod)OMe]_2$ (4.97 mg, 1.5 mol%), B_2pin_2 (127.0 mg, 1.0 equiv.), ligand **L9** (3.4 mg, 3.0 mol%), HBpin (3.2 mg, 5.0 mol%) and dry cyclohexane (2.0 mL). The reaction mixture was stirred for 2 minutes at room temperature and then (2-cyclohexylphenoxy)triisopropylsilane (0.5 mmol, 166 mg) was added. The microreactor was capped with a teflon pressure cap and stirred for 24 h at room temperature. After completion (judged by GC-MS), CyH was removed under reduced pressure and chromatographic separation with silica gel (2% EtOAc in hexane as eluent) gave 206 mg (90%) of the *meta*-borylated **(5o)** product as a colourless gummy liquid.

^{1}H NMR (400 MHz, $CDCl_3$): δ 7.34 (d, *J* = 7.2 Hz, 1H), 7.19 – 7.17 (m, 2H), 3.04 (t, *J* = 10.2 Hz, 1H), 1.83 (d, *J* = 10.8 Hz, 4H), 1.75 (d, *J* = 12.4 Hz, 1H), 1.42 – 1.31 (m, 20H), 1.13 (d, *J* = 7.2 Hz, 18H).

^{13}C NMR (100 MHz, $CDCl_3$): δ 152.8, 141.2, 127.4, 126.3, 124.0, 83.6, 37.3, 33.2, 27.2, 26.5, 25.0, 18.3, 13.2.

^{11}B NMR (128 MHz, $CDCl_3$): δ 32.1.

HRMS (ESI) *m/z* calcd for $C_{27}H_{47}BO_3Si$ $[M+H]^+$ 459.3466, found 459.3469.

Same reaction was carried out with dtbpy ligand under identical conditions.

Result: GC-MS Conversion = 80%; m/p = 65:35.

Meta-borylation of 1-(2-((triisopropylsilyl)oxy)phenyl)ethan-1-one **(4p)***:*

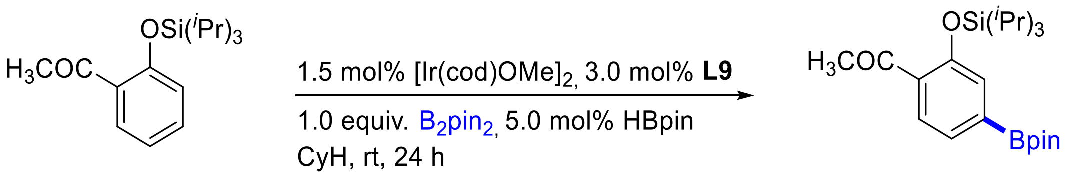

4p: 0.5 mmol

5p: 85% (isolated yield)
m/p = 97/3

In an argon-filled glove box, a 5.0 mL Wheaton microreactor was charged with [Ir(cod)OMe]$_2$ (4.97 mg, 1.5 mol%), B$_2$pin$_2$ (127.0 mg, 1.0 equiv.), ligand **L9** (3.4 mg, 3.0 mol%), HBpin (3.2 mg, 5.0 mol%) and dry cyclohexane (2.0 mL. The reaction mixture was stirred for 2 minutes at room temperature and then 1-(2-((triisopropylsilyl)oxy)phenyl)ethan-1-one (0.5 mmol, 146 mg) was added. The microreactor was capped with a teflon pressure cap and stirred for 24 h at room temperature. After completion (judged by GC-MS), CyH was removed under reduced pressure and chromatographic separation with silica gel (3% EtOAc in hexane as eluent) gave 177 mg (85%) of the *meta*-borylated **(5p)** product as a colourless gummy liquid.

^{1}H NMR (400 MHz, CDCl$_3$): δ 7.55 (d, J = 7.6 Hz, 1H), 7.36 (d, J = 7.6 Hz, 1H), 7.28 (s, 1H), 2.62 (s, 3H), 1.35–1.32 (m, 15H), 1.12 (d, J = 7.2 Hz, 18H).
^{13}C NMR (100 MHz, CDCl$_3$): δ 201.6, 154.4, 133.2, 129.1, 127.0, 125.8, 84.2, 31.5, 25.0, 18.1, 13.3.
^{11}B NMR (128 MHz, CDCl$_3$): δ 31.2.
HRMS (ESI) *m/z* calcd for $C_{23}H_{39}BO_4Si$ [M+H]$^+$ 419.2789, found 419.2787.
Same reaction was carried out with dtbpy ligand under identical conditions.
Result: Complex mixture from GC-MS analysis.

Meta-borylation of ethyl 2-(2-((triisopropylsilyl)oxy)phenyl)acetate **(4q)***:*

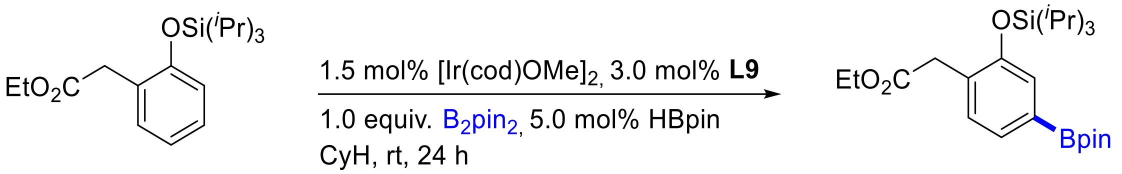

4q: 0.5 mmol

5q: 92% (isolated yield)
m/p = 99/1

In an argon-filled glove box, a 5.0 mL Wheaton microreactor was charged with [Ir(cod)OMe]$_2$ (4.97 mg, 1.5 mol%), B$_2$pin$_2$ (127.0 mg, 1.0 equiv.), ligand **L9** (3.4 mg, 3.0 mol%), HBpin (3.2 mg, 5.0 mol%) and dry cyclohexane (2.0 mL). The reaction mixture was stirred for 2 minutes at room temperature and then ethyl 2-(2-((triisopropylsilyl)oxy)phenyl)acetate (0.5 mmol, 168 mg) was added. The microreactor was capped with a teflon pressure cap and stirred for 24 h at room temperature. After completion (judged by GC-MS), CyH was removed under reduced pressure and chromatographic separation with silica gel (2% EtOAc in hexane as eluent) gave 212 mg (92%) of the *meta*-borylated **(5q)** product as a colourless gummy liquid.

^1H NMR (400 MHz, $CDCl_3$): δ 7.32 (d, J = 7.2 Hz, 1H), 7.22 (s, 1H), 7.18 (d, J = 7.2 Hz, 1H), 4.14 – 4.09 (m, 2H), 3.65 (s, 2H), 1.35 – 1.29 (m, 15H), 1.21 (t, J = 7.2 Hz, 3H), 1.11 (d, J = 7.2 Hz, 18H).

^{13}C NMR (100 MHz, $CDCl_3$): δ 171.6, 153.7, 130.7, 128.0, 127.2, 123.8, 83.8, 60.7, 36.6, 25.0, 18.2, 14.3, 13.1.

^{11}B NMR (128 MHz, $CDCl_3$): δ 31.5.

HRMS (ESI) *m/z* calcd for $C_{25}H_{43}BO_5Si$ $[M+H]^+$ 463.3051, found 463.3038.

Same reaction was carried out with dtbpy ligand under identical conditions.

Result: GC-MS Conversion = 60%; m/p = 70:30.

Meta-borylation of N,N-diethyl-2-((triisopropylsilyl)oxy)benzamide **(4r)**:

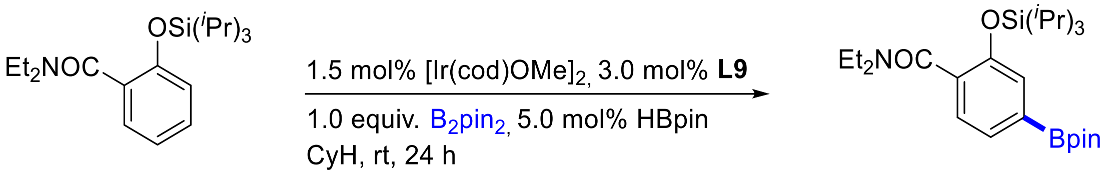

4r: 0.5 mmol

5r: 87% (isolated yield)
m/p = 95/5

In an argon-filled glove box, a 5.0 mL Wheaton microreactor was charged with $[Ir(cod)OMe]_2$ (4.97 mg, 1.5 mol%), B_2pin_2 (127.0 mg, 1.0 equiv.), ligand **L9** (3.4 mg, 3.0 mol%), HBpin (3.2 mg, 5.0 mol%) and dry cyclohexane (2.0 mL). The reaction mixture was stirred for 2 minutes at room temperature and then N,N-diethyl-2-((triisopropylsilyl)oxy)benzamide (0.5 mmol, 174.5 mg) was added. The microreactor was capped with a teflon pressure cap and stirred for 24 h at room temperature. After completion (judged by GC-MS), CyH was removed under reduced pressure and chromatographic separation with silica gel (20% EtOAc in hexane as eluent) gave 206 mg (87%) of the *meta*-borylated **(5r)** product as a colourless gummy liquid.

^1H NMR (400 MHz, $CDCl_3$): δ 7.36 (d, J = 7.2 Hz, 1H), 7.23 (s, 1H), 7.16 (d, J = 7.2 Hz, 1H), 3.52 (q, J = 7.2 Hz, 2H), 3.23–3.07 (m, 2H), 1.33 (s, 12H), 1.30–1.26 (m, 3H), 1.23 (t, J = 7.2 Hz, 3H), 1.11–1.07 (m, 18H), 0.99 (t, J = 7.2 Hz, 3H).

^{13}C NMR (100 MHz, $CDCl_3$): δ 169.1, 151.0, 132.0, 127.3, 127.3, 124.8, 84.0, 43.0, 39.3, 25.0, 18.1, 14.2, 13.3, 13.0.

^{11}B NMR (128 MHz, $CDCl_3$): δ 31.8.

HRMS (ESI) *m/z* calcd for $C_{26}H_{46}BNO_4Si$ $[M+H]^+$ 476.3367, found 476.3371.

Same reaction was carried out with dtbpy ligand under identical conditions.

Result: GC-MS Conversion = 72%; m/p = 67:33.

Meta-borylation of morpholino(2-((triisopropylsilyl)oxy)phenyl)methanone **(4s)**:

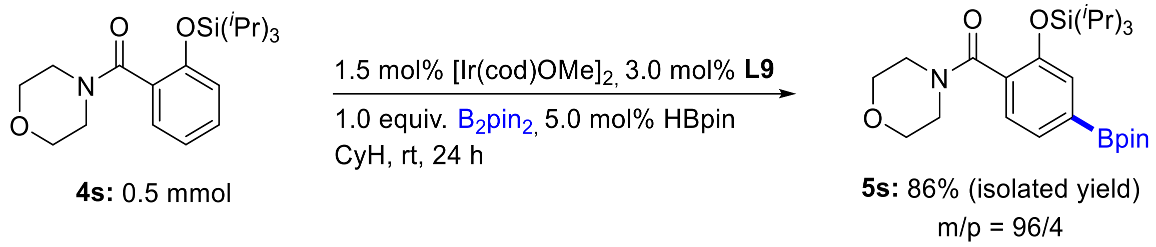

In an argon-filled glove box, a 5.0 mL Wheaton microreactor was charged with $[Ir(cod)OMe]_2$ (4.97 mg, 1.5 mol%), B_2pin_2 (127.0 mg, 1.0 equiv.), ligand **L9** (3.4 mg, 3.0 mol%), HBpin (3.2 mg, 5.0 mol%) and dry cyclohexane (2.0 mL). The reaction mixture was stirred for 2 minutes at room temperature and then morpholino(2-((triisopropylsilyl)oxy)phenyl)methanone (0.5 mmol, 181.5 mg) was added. The microreactor was capped with a teflon pressure cap and stirred for 24 h at room temperature. After completion (judged by GC-MS), CyH was removed under reduced pressure and chromatographic separation with silica gel (10% EtOAc in hexane as eluent) gave 205 mg (84%) of the *meta*-borylated **(5s)** product as a colourless gummy liquid.

^{1}H NMR (400 MHz, $CDCl_3$): δ 7.39 (d, J = 7.2 Hz, 1H), 7.24 – 7.21 (m, 2H), 4.08 – 4.03 (m, 1H), 3.83 – 3.78 (m, 1H), 3.69 – 3.62 (m, 2H), 3.51 – 3.42 (m, 2H), 3.33 – 3.19 (m, 2H), 1.33 (brs, 15H), 1.24 (s, 18H).

^{13}C NMR (100 MHz, $CDCl_3$): δ 168.3, 151.2, 130.2, 127.6, 1247, 84.0, 75.0, 66.9, 66.8, 47.0, 41.8, 24.9, 18.1, 12.8.

^{11}B NMR (128 MHz, $CDCl_3$): δ 29.7.

HRMS (ESI) *m/z* calcd for $C_{26}H_{44}BNO_5Si$ $[M+H]^+$ 490.3160, found 490.3163.

Same reaction was carried out with dtbpy ligand under identical conditions.

Result: GC-MS Conversion = 78%; m/p = 60:40.

Meta-borylation of triisopropyl(3-(trifluoromethyl)phenoxy)silane **(4t)**:

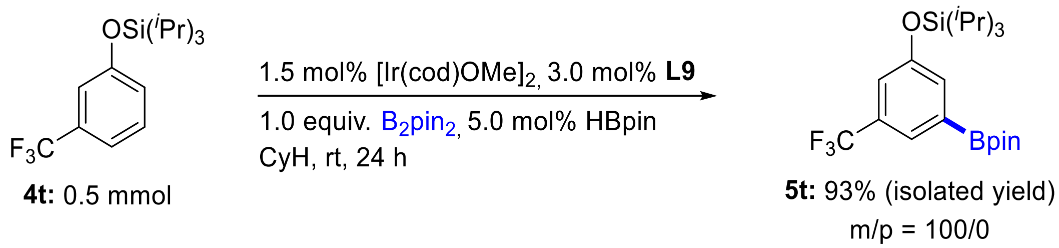

In an argon-filled glove box, a 5.0 mL Wheaton microreactor was charged with $[Ir(cod)OMe]_2$ (4.97 mg, 1.5 mol%), B_2pin_2 (127.0 mg, 1.0 equiv.), ligand **L9** (3.4 mg, 3.0 mol%), HBpin (3.2 mg, 5.0 mol%) and dry cyclohexane (2.0 mL). The reaction mixture was stirred for 2 minutes at room temperature and then triisopropyl(3-(trifluoromethyl)phenoxy)silane (0.5 mmol, 159 mg) was added. The microreactor was capped with a teflon pressure cap and stirred for 24 h at room temperature. After completion (judged by GC-MS), CyH was removed under reduced pressure and chromatographic separation with silica gel (5% EtOAc in hexane as eluent) gave 206 mg (93%) of the *meta*-borylated **(5t)** product as a colourless gummy liquid.

^{1}H NMR (400 MHz, $CDCl_3$): δ 7.63 (s, 1H), 7.45 (d, J = 2.0 Hz, 1H), 7.16 (s, 1H), 1.35 (s, 12H), 1.30 – 1.26 (m, 3H), 1.11 (d, J = 7.2 Hz, 18H).

^{13}C NMR (100 MHz, $CDCl_3$): δ 155.95, 131.4 (q, J = 31.9 Hz), 129.4, 124.2 (d, J = 270.9 Hz), 123.9 (q, J = 3.8 Hz), 119.0 (q, J = 3.6 Hz), 84.4, 25.0, 18.0, 12.8.

^{11}B NMR (128 MHz, $CDCl_3$): δ 30.2.

HRMS (ESI) *m/z* calcd for $C_{22}H_{36}BF_3O_3Si$ $[M+H]^+$ 445.2557, found 445.2562.

Same reaction was carried out with dtbpy ligand under identical conditions.

Result: GC-MS Conversion = 58%; m/p = 100:0.

Meta-borylation of 3-((triisopropylsilyl)oxy)benzonitrile **(4u)**:

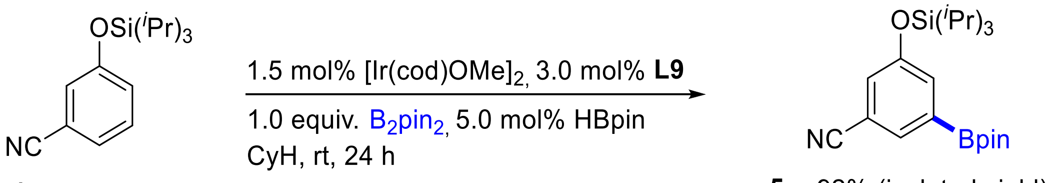

4u: 0.5 mmol

5u: 92% (isolated yield)
m/p = 100/0

In an argon-filled glove box, a 5.0 mL Wheaton microreactor was charged with $[Ir(cod)OMe]_2$ (4.97 mg, 1.5 mol%), B_2pin_2 (127.0 mg, 1.0 equiv.), ligand **L9** (3.4 mg, 3.0 mol%), HBpin (3.2 mg, 5.0 mol%) and dry cyclohexane (2.0 mL). The reaction mixture was stirred for 2 minutes at room temperature and then 3-((triisopropylsilyl)oxy)benzonitrile (0.5 mmol, 137.5 mg) was added. The microreactor was capped with a teflon pressure cap and stirred for 24 h at room temperature. After completion (judged by GC-MS), CyH was removed under reduced pressure and chromatographic separation with silica gel (5% EtOAc in hexane as eluent) gave 184 mg (92%) of the *meta*-borylated **(5u)** product as a colourless gummy liquid.

^{1}H NMR (400 MHz, $CDCl_3$): δ 7.66 (s, 1H), 7.49 (d, J = 2.0 Hz, 1H), 7.17 – 7.16 (m, 1H), 1.34 (s, 12H), 1.29 – 1.27 (m, 3H), 1.10 (d, J = 7.2 Hz, 18H).

^{13}C NMR (100 MHz, $CDCl_3$): δ 156.0, 131.1, 130.9, 125.0, 118.9, 112.9, 84.6, 25.0, 18.0, 12.8.

^{11}B NMR (128 MHz, $CDCl_3$): δ 30.8.

HRMS (ESI) *m/z* calcd for $C_{22}H_{36}BNO_3Si$ $[M+H]^+$ 402.2636, found 402.2641.

Same reaction was carried out with dtbpy ligand under identical conditions.

Result: GC-MS Conversion = 55%; m/p = 80:20.

Meta-borylation of (3-fluorophenoxy)triisopropylsilane **(4v)***:*

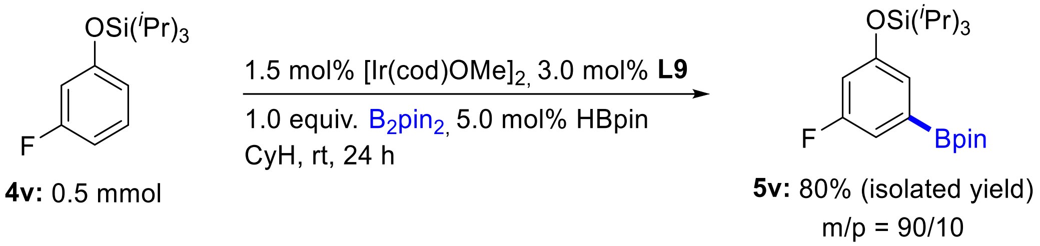

In an argon-filled glove box, a 5.0 mL Wheaton microreactor was charged with $[Ir(cod)OMe]_2$ (4.97 mg, 1.5 mol%), B_2pin_2 (127.0 mg, 1.0 equiv.), ligand **L9** (3.4 mg, 3.0 mol%), HBpin (3.2 mg, 5.0 mol%) and dry cyclohexane (2.0 mL). The reaction mixture was stirred for 2 minutes at room temperature and then (3-fluorophenoxy)triisopropylsilane (0.5 mmol, 134 mg) was added. The microreactor was capped with a teflon pressure cap and stirred for 24 h at room temperature. After completion (judged by GC-MS), CyH was removed under reduced pressure and chromatographic separation with silica gel (5% EtOAc in hexane as eluent) gave 157 mg (80%) of the *meta*-borylated **(5v)** product as a colourless gummy liquid.

^{1}H NMR (400 MHz, $CDCl_3$): δ 7.09 – 7.05 (m, 2H), 6.66 (dt, J = 10.8, 2.4 Hz, 1H), 1.33 (s, 12H), 1.29 – 1.25 (m, 3H), 1.10 (d, J = 7.2 Hz, 18H).

^{13}C NMR (100 MHz, $CDCl_3$): δ 163.2 (d, J = 245.0 Hz), 157.1 (d, J = 10.1 Hz), 121.9 (d, J = 2.6 Hz), 113.6 (d, J = 19.2 Hz), 110.0 (d, J = 22.5 Hz), 84.2, 25.0, 18.0, 12.8.

^{11}B NMR (128 MHz, $CDCl_3$) δ 30.2.

HRMS (ESI) *m/z* calcd for $C_{21}H_{36}BFO_3Si$ $[M+H]^+$ 395.2589, found 395.2589.

Same reaction was carried out with dtbpy ligand under identical conditions.

Result: GC-MS Conversion = 40%; m/p = 62:38.

Meta-borylation of triisopropyl((5,6,7,8-tetrahydronaphthalen-2-yl)oxy)silane **(4w)**:

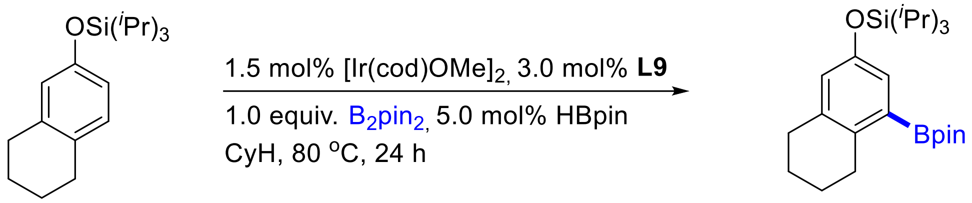

4w: 0.5 mmol

5w: 65% (isolated yield)
m = 100

In an argon-filled glove box, a 5.0 mL Wheaton microreactor was charged with $[Ir(cod)OMe]_2$ (4.97 mg, 1.5 mol%), B_2pin_2 (127.0 mg, 1.0 equiv.), ligand **L9** (3.4 mg, 3.0 mol%), HBpin (3.2 mg, 5.0 mol%) and dry cyclohexane (2.0 mL). The reaction mixture was stirred for 2 minutes at room temperature and then triisopropyl((5,6,7,8-tetrahydronaphthalen-2-yl)oxy)silane (0.5 mmol, 152 mg) was added. The microreactor was capped with a teflon pressure cap and placed into pre-heated aluminum block at 80 °C and stirred for 24 h. After completion (judged by GC-MS), CyH was removed under reduced pressure and chromatographic separation with neutral silica gel (5% EtOAc in hexane as eluent) gave 114 mg (53%) of the *meta*-borylated **(5w)** product as a colourless gummy liquid.

^{1}H NMR (400 MHz, $CDCl_3$) δ 7.11 (d, *J* = 2.0 Hz, 1H), 6.64 (d, *J* = 2.0 Hz, 1H), 2.94 (t, *J* = 6.0 Hz, 2H), 2.70 (t, *J* = 5.2 Hz, 2H), 1.75 – 1.74 (m, 4H), 1.33 (s, 12H), 1.27 – 1.23 (m, 3H), 1.11 (d, *J* = 7.2 Hz, 18H).

^{13}C NMR (100 MHz, $CDCl_3$): δ 152.9, 137.9, 135.7, 124.8, 122.6, 83.4, 30.6, 28.8, 25.0, 23.9, 23.1, 18.2, 12.9.

^{11}B NMR (128 MHz, $CDCl_3$): δ 31.4.

HRMS (ESI) *m/z* calcd for $C_{25}H_{43}BO_3Si$ $[M+H]^+$ 431.3153, found 431.3156.

Same reaction was carried out with dtbpy ligand under identical conditions.

Result: No reaction.

Meta-borylation of triisopropyl((5,6,7,8-tetrahydronaphthalen-1-yl)oxy)silane **(4x)**:

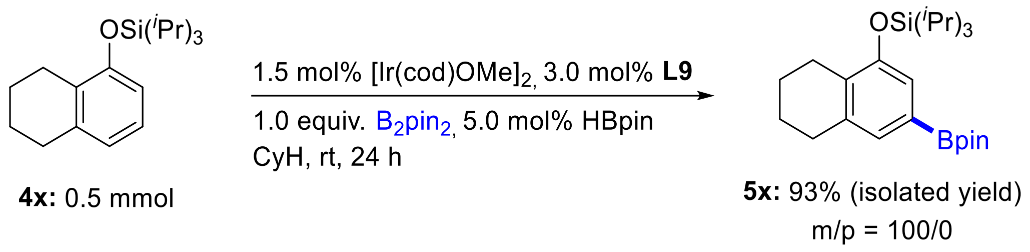

In an argon-filled glove box, a 5.0 mL Wheaton microreactor was charged with $[Ir(cod)OMe]_2$ (4.97 mg, 1.5 mol%), B_2pin_2 (127.0 mg, 1.0 equiv.), ligand **L9** (3.4 mg, 3.0 mol%), HBpin (3.2 mg, 5.0 mol%) and dry cyclohexane (2.0 mL). The reaction mixture was stirred for 2 minutes at room temperature and then triisopropyl((5,6,7,8-tetrahydronaphthalen-1-yl)oxy)silane (0.5 mmol, 152 mg) was added. The microreactor was capped with a teflon pressure cap and stirred for 24 h at room temperature. After completion (judged by GC-MS), CyH was removed under reduced pressure and chromatographic separation with silica gel (5% EtOAc in hexane as eluent) gave 199 mg (93%) of the *meta*-borylated **(5x)** product as a colourless gummy liquid.

^{1}H NMR (400 MHz, $CDCl_3$): δ 7.16 (s, 1H), 7.03 (s, 1H), 2.75 (dt, *J* = 18.0, 6.4 Hz), 1.83 – 1.74 (m, 4H), 1.38 – 1.31 (m, 15H), 1.14 (d, *J* = 7.2 Hz, 18H).

^{13}C NMR (100 MHz, $CDCl_3$): δ 153.6, 138.3, 131.7, 128.2, 120.6, 83.5, 29.7, 25.0, 24.5, 23.2, 23.1, 18.3, 13.2.

^{11}B NMR (128 MHz, $CDCl_3$): δ 30.5.

HRMS (ESI) *m/z* calcd for $C_{25}H_{43}BO_3Si$ $[M+H]^+$ 431.3153, found 431.3157.

Same reaction was carried out with dtbpy ligand under identical conditions.

Result: GC-MS Conversion = 28%; m/p = 100:0.

Meta-borylation of (2,6-difluorophenoxy)triisopropylsilane **(4y)**:

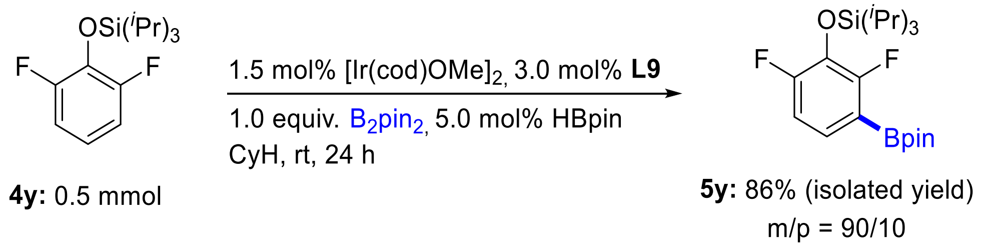

In an argon-filled glove box, a 5.0 mL Wheaton microreactor was charged with $[Ir(cod)OMe]_2$ (4.97 mg, 1.5 mol%), B_2pin_2 (127.0 mg, 1.0 equiv.), ligand **L9** (3.4 mg, 3.0 mol%), HBpin (3.2 mg, 5.0 mol%) and dry cyclohexane (2.0 mL). The reaction mixture was stirred for 2 minutes at room temperature and then (2,6-difluorophenoxy)triisopropylsilane (0.5 mmol, 143 mg) was added. The microreactor was capped with a teflon pressure cap and stirred for 24 h at room temperature. After completion (judged by GC-MS), CyH was removed under reduced pressure and chromatographic separation with silica gel (2% EtOAc in hexane as eluent) gave 177 mg (86%) of the *meta*-borylated **(5y)** product as a colourless gummy liquid.

^{1}H NMR (400 MHz, $CDCl_3$): δ 7.28 – 7.20 (m, 1H), 6.86 – 6.81 (m, 1H), 1.35 (s, 12H), 1.31 – 1.28 (m, 3H), 1.09 (d, J = 7.2 Hz, 18H).
^{11}B NMR (128 MHz, $CDCl_3$): δ 30.2.
HRMS (ESI) *m/z* calcd for $C_{21}H_{35}BF_2O_3Si$ $[M+H]^+$ 413.2495, found 413.2501.
Note: After several hour of scanning carbon NMR is not appeared properly.
Same reaction was carried out with dtbpy ligand under identical conditions.
Result: GC-MS Conversion = 58%; m/p = 48:52.

Meta-borylation of (2,4-dichlorophenoxy)triisopropylsilane **(4z)***:*

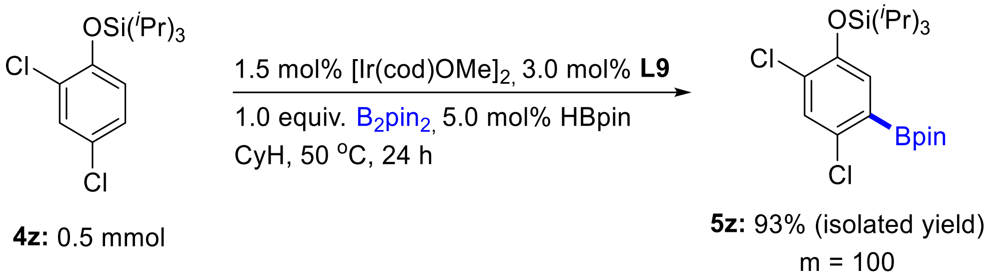

In an argon-filled glove box, a 5.0 mL Wheaton microreactor was charged with $[Ir(cod)OMe]_2$ (4.97 mg, 1.5 mol%), B_2pin_2 (127.0 mg, 1.0 equiv.), ligand **L9** (3.4 mg, 3.0 mol%), HBpin (3.2 mg, 5.0 mol%) and dry cyclohexane (2.0 mL). The reaction mixture was stirred for 2 minutes at room temperature and then (2,4-dichlorophenoxy)triisopropylsilane (0.5 mmol, 159 mg) was added. The microreactor was capped with a teflon pressure cap and placed into pre-heated aluminum block at 50 °C and stirred for 24 h. After completion (judged by GC-MS), CyH was removed under reduced pressure and chromatographic separation with neutral silica gel (5%

EtOAc in hexane as eluent) gave 206 mg (93%) of the *meta*-borylated **(5z)** product as a colourless gummy liquid.

^{1}H NMR (400 MHz, $CDCl_3$): δ 7.35 – 7.33 (m, 1H), 7.20 (s, 1H), 1.35 (s, 12H), 1.29 – 1.26 (m, 3H), 1.11 (d, *J* = 7.2 Hz, 18H).
^{13}C NMR (100 MHz, $CDCl_3$): δ 150.4, 131.2, 130.9, 128.5, 127.2, 84.4, 24.9, 18.0, 12.9.
^{11}B NMR (128 MHz, $CDCl_3$): δ 30.4.
HRMS (ESI) *m/z* calcd for $C_{21}H_{35}BCl_2O_3Si$ $[M+H]^+$ 445.1904, found 445.1907.
Same reaction was carried out with dtbpy ligand under identical conditions.
Result: GC-MS Conversion = 31%; m = 100.

Meta-borylation of (2-bromo-4-chlorophenoxy)triisopropylsilane **(4aa)***:*

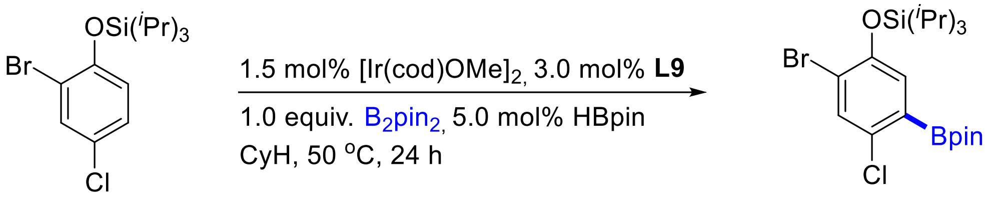

4aa: 0.5 mmol

5aa: 95% (isolated yield)
m = 100

In an argon-filled glove box, a 5.0 mL Wheaton microreactor was charged with $[Ir(cod)OMe]_2$ (4.97 mg, 1.5 mol%), B_2pin_2 (127.0 mg, 1.0 equiv.), ligand **L9** (3.4 mg, 3.0 mol%), HBpin (3.2 mg, 5.0 mol%) and dry cyclohexane (2.0 mL). The reaction mixture was stirred for 2 minutes at room temperature and then (2-bromo-4-chlorophenoxy)triisopropylsilane (0.5 mmol, 181 mg) was added. The microreactor was capped with a teflon pressure cap and placed into pre-heated aluminum block at 50 °C and stirred for 24 h. After completion (judged by GC-MS), CyH was removed under reduced pressure and chromatographic separation with neutral silica gel (4% EtOAc in hexane as eluent) gave 231 mg (95%) of the *meta*-borylated **(5aa)** product as a colourless gummy liquid.

^{1}H NMR (400 MHz, $CDCl_3$): δ 7.50 (s, 1H), 7.19 (s, 1H), 1.35 (s, 15H), 1.31 – 1.27 (m, 3H), 1.12 (d, *J* = 7.2 Hz, 18H).
^{13}C NMR (100 MHz, $CDCl_3$): δ 151.4, 133.8, 131.2, 126.6, 118.2, 84.4, 24.9, 18.1, 12.9.
^{11}B NMR (128 MHz, $CDCl_3$) δ 30.1.
HRMS (ESI) *m/z* calcd for $C_{21}H_{35}BBrClO_3Si$ $[M+H]^+$ 489.1399, found 489.1395.

Same reaction was carried out with dtbpy ligand under identical conditions.

Result: GC-MS Conversion = 47%; m = 100.

Meta-borylation of (3,4-difluorophenoxy)triisopropylsilane **(4ab)**:

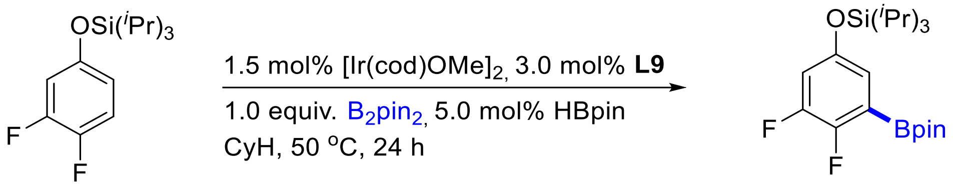

4ab: 0.5 mmol

5ab: 93% (isolated yield)
m = 100

In an argon-filled glove box, a 5.0 mL Wheaton microreactor was charged with $[Ir(cod)OMe]_2$ (4.97 mg, 1.5 mol%), B_2pin_2 (127.0 mg, 1.0 equiv.), ligand **L9** (3.4 mg, 3.0 mol%), HBpin (3.2 mg, 5.0 mol%) and dry cyclohexane (2.0 mL). The reaction mixture was stirred for 2 minutes at room temperature and then (3,4-difluorophenoxy)triisopropylsilane (0.5 mmol, 143 mg) was added. The microreactor was capped with a teflon pressure cap and placed into pre-heated aluminum block at 50 ^{o}C and stirred for 24 h. After completion (judged by GC-MS), CyH was removed under reduced pressure and chromatographic separation with neutral silica gel (5% EtOAc in hexane as eluent) gave 192 mg (93%) of the *meta*-borylated **(5ab)** product as a colourless gummy liquid.

^{1}H NMR (400 MHz, $CDCl_3$) δ 6.94 – 6.92 (m, 1H), 6.78 – 6.73 (m, 1H), 1.35 (s, 12H), 1.26 – 1.24 (m, 3H), 1.09 (d, J = 7.2 Hz, 18H).

^{13}C NMR (100 MHz, $CDCl_3$): δ 151.8 (dd, J = 2.6, 8.1 Hz), 150.2 (dd, J = 15.9, 247.2 Hz), 149.3 (dd, J = 11.8, 243.5 Hz), 121.3 (dd, J = 3.1, 6.0 Hz), 111.8 (d, J = 18.1 Hz), 84.3, 24.9, 18.0, 12.7.

^{11}B NMR (128 MHz, $CDCl_3$): δ 29.9.

HRMS (ESI) *m/z* calcd for $C_{21}H_{35}BF_2O_3Si$ $[M+H]^+$ 413.2495, found 413.2495.

Same reaction was carried out with dtbpy ligand under identical conditions.

Result: GC-MS Conversion = 38%; m = 100.

Meta-borylation of (4-fluoro-2-methoxyphenoxy)triisopropylsilane **(4ac)**:

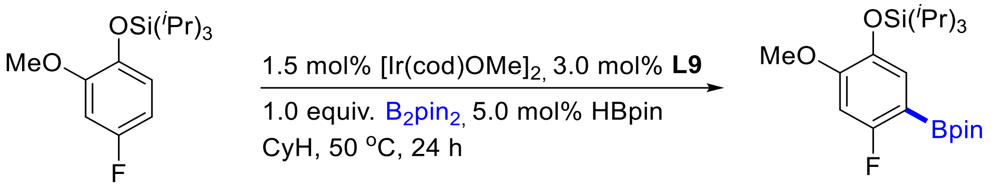

4ac: 0.5 mmol

5ac: 90% (isolated yield)
m = 100

In an argon-filled glove box, a 5.0 mL Wheaton microreactor was charged with $[Ir(cod)OMe]_2$ (4.97 mg, 1.5 mol%), B_2pin_2 (127.0 mg, 1.0 equiv.), ligand **L9** (3.4 mg, 3.0 mol%), HBpin (3.2 mg, 5.0 mol%) and dry cyclohexane (2.0 mL). The reaction mixture was stirred for 2 minutes at room temperature and then (4-fluoro-2-methoxyphenoxy)triisopropylsilane (0.5 mmol, 149 mg) was added. The microreactor was capped with a teflon pressure cap and placed into pre-heated aluminum block at 50 °C and stirred for 24 h. After completion (judged by GC-MS), CyH was removed under reduced pressure and chromatographic separation with neutral silica gel (5% EtOAc in hexane as eluent) gave 190 mg (90%) of the *meta*-borylated **(5ac)** product as a colourless gummy liquid.

^{1}H NMR (400 MHz, $CDCl_3$): δ 7.15 (d, J = 6.0 Hz, 1H), 6.53 (d, J = 10.4 Hz, 1H), 3.79 (s, 3H), 1.33 (s, 12H), 1.25 – 1.19 (m, 3H), 1.08 (d, J = 7.2 Hz, 18H).
^{13}C NMR (100 MHz, $CDCl_3$): δ 162.6 (d, J = 243.4 Hz), 154.4 (d, J = 10.8 Hz), 141.2 (d, J = 2.8 Hz), 126.3 (d, J = 10.1 Hz), 99.7 (d, J = 30.2 Hz), 83.6, 55.5, 24.9, 18.0, 13.0.
^{11}B NMR (128 MHz, $CDCl_3$): δ 29.8.
HRMS (ESI) *m/z* calcd for $C_{22}H_{38}BFO_4Si$ $[M+H]^+$ 425.2695, found 425.2699.
Same reaction was carried out with dtbpy ligand under identical conditions.
Result: GC-MS Conversion = 52%; meta = 100.

Meta-borylation of (2,3-dimethoxyphenoxy)triisopropylsilane **(4ad)**:

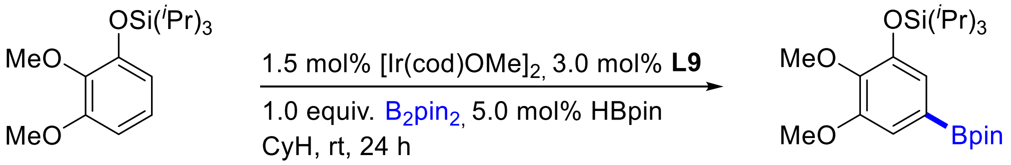

4ad: 0.5 mmol

5ad: 95% (isolated yield)
m/p = 100/0

In an argon-filled glove box, a 5.0 mL Wheaton microreactor was charged with $[Ir(cod)OMe]_2$ (4.97 mg, 1.5 mol%), B_2pin_2 (127.0 mg, 1.0 equiv.), ligand **L9** (3.4 mg, 3.0 mol%), HBpin (3.2 mg, 5.0 mol%) and dry cyclohexane (2.0 mL). The reaction mixture was stirred for 2 minutes at room temperature and then (2,3-dimethoxyphenoxy)triisopropylsilane (0.5 mmol, 155 mg) was added. The microreactor was capped with a teflon pressure cap and stirred for 24 h at room temperature. After completion (judged by GC-MS), CyH was removed under reduced pressure and chromatographic separation with silica gel (5% EtOAc in hexane as eluent) gave 207 mg (95%) of the *meta*-borylated **(5ad)** product as a colourless gummy liquid.

^{1}H NMR (400 MHz, $CDCl_3$): δ 6.98 (s, 1H), 6.96 (s, 1H), 3.88 (s, 3H), 3.82 (s, 3H), 1.32 (s, 1H), 1.29 – 1.25 (m, 3H), 1.10 (d, J = 7.6 Hz, 18H).

^{13}C NMR (100 MHz, $CDCl_3$): δ 153.5, 149.6, 143.1, 120.4, 111.0, 83.8, 60.6, 56.2, 25.0, 18.1, 13.0.

^{11}B NMR (128 MHz, $CDCl_3$): δ 31.3.

HRMS (ESI) *m/z* calcd for $C_{23}H_{41}BO_5Si$ $[M+H]^+$ 437.2895, found 437.2898.

Same reaction was carried out with dtbpy ligand under identical conditions.

Result: GC-MS Conversion = 44%; m/p = 100/0.

Meta-borylation of (2,3-dimethylphenoxy)triisopropylsilane **(4ae)**:

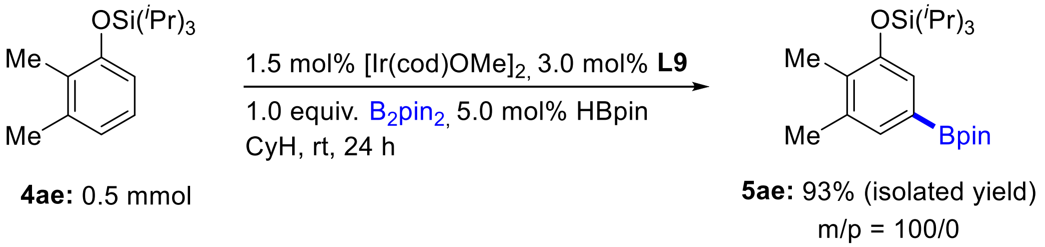

In an argon-filled glove box, a 5.0 mL Wheaton microreactor was charged with $[Ir(cod)OMe]_2$ (4.97 mg, 1.5 mol%), B_2pin_2 (127.0 mg, 1.0 equiv.), ligand **L9** (3.4 mg, 3.0 mol%), HBpin (3.2 mg, 5.0 mol%) and dry cyclohexane (2.0 mL). The reaction mixture was stirred for 2 minutes at room temperature and then (2,3-dimethylphenoxy)triisopropylsilane (0.5 mmol, 139 mg) was added. The microreactor was capped with a teflon pressure cap and stirred for 24 h at room temperature. After completion (judged by GC-MS), CyH was removed under reduced pressure and chromatographic separation with silica gel (5% EtOAc in hexane as eluent) gave 187 mg (93%) of the *meta*-borylated **(5ae)** product as a colourless gummy liquid.

^{1}H NMR (400 MHz, $CDCl_3$): δ 7.22 (s, 1H), 7.09 (s, 1H), 2.28 (s, 3H), 2.20 (s, 3H), 1.38 – 1.32 (m, 15H), 1.14 (d, J = 7.6 Hz, 18H).

^{13}C NMR (100 MHz, $CDCl_3$): δ 153.8, 137.6, 130.7, 128.8, 121.7, 83.5, 25.0, 20.2, 18.2, 13.2, 12.9.

^{11}B NMR (128 MHz, $CDCl_3$): δ 30.6.

HRMS (ESI) *m/z* calcd for $C_{23}H_{41}BO_3Si$ $[M+H]^+$ 405.2996, found 405.2999.

Same reaction was carried out with dtbpy ligand under identical conditions.

Result: GC-MS Conversion = 55%; m/p = 100/0.

Meta-borylation of (4-chloro-3-methylphenoxy)triisopropylsilane **(4af)**:

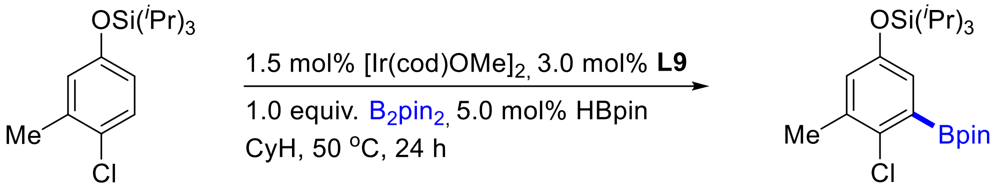

4af: 0.5 mmol

5af: 99% (isolated yield)
m = 100

In an argon-filled glove box, a 5.0 mL Wheaton microreactor was charged with $[Ir(cod)OMe]_2$ (4.97 mg, 1.5 mol%), B_2pin_2 (127.0 mg, 1.0 equiv.), ligand **L9** (3.4 mg, 3.0 mol%), HBpin (3.2 mg, 5.0 mol%) and dry cyclohexane (2.0 mL). The reaction mixture was stirred for 2 minutes at room temperature and then (4-chloro-3-methylphenoxy)triisopropylsilane (0.5 mmol, 149 mg) was added. The microreactor was capped with a teflon pressure cap and placed into pre-heated aluminum block at 50 °C and stirred for 24 h. After 24 h, the crude reaction mixture was analyzed from ^{1}H-NMR and the results are shown in the above scheme.

^{1}H NMR (400 MHz, $CDCl_3$) δ 6.97 (d, J = 2.8 Hz, 1H), 6.78 (d, J = 2.8 Hz, 1H), 2.29 (s, 3H), 1.36 (s, 12H), 1.25 – 1.23 (m, 3H), 1.09 (d, J = 7.2 Hz, 18H).

^{11}B NMR (128 MHz, $CDCl_3$): δ 31.0.

HRMS (ESI) *m/z* calcd for $C_{22}H_{38}BClO_3Si$ $[M+H]^+$ 425.2450, found 425.2444.

Same reaction was carried out with dtbpy ligand under identical conditions.

Result: No reaction.

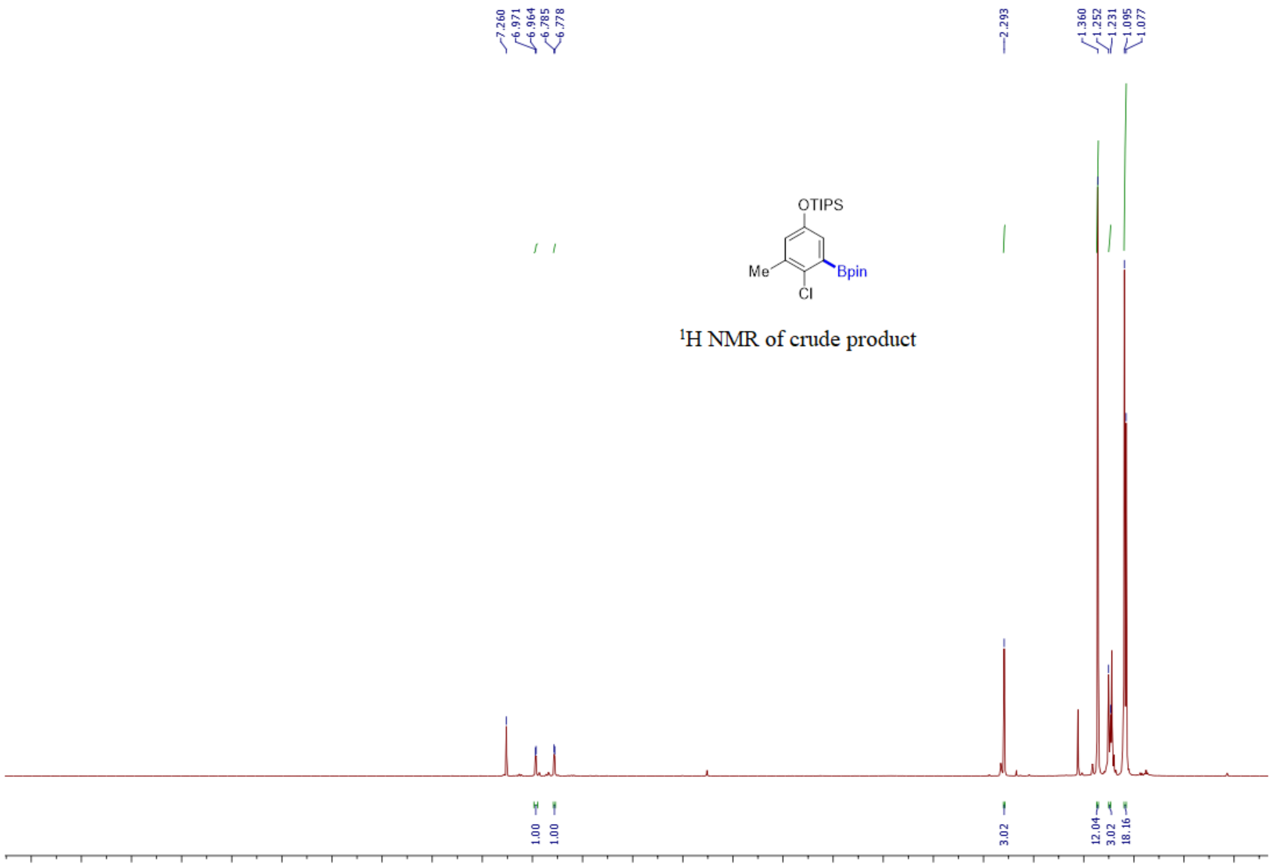

Supplementary Fig. 11: ^{1}H-NMR spectra of crude **5af** with **L9** (25 ^{o}C, 400 MHz, $CDCl_3$)

Meta-borylation of 2,2'-bis((triisopropylsilyl)oxy)-1,1'-biphenyl **(4ag)***:*

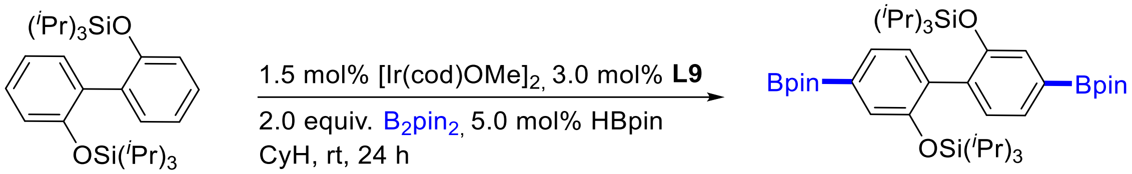

4ag: 0.5 mmol

5ag: 80% (isolated yield)
m/p = 96/4

In an argon-filled glove box, a 5.0 mL Wheaton microreactor was charged with $[Ir(cod)OMe]_2$ (9.94 mg, 3 mol%), B_2pin_2 (254.0 mg, 2.0 equiv.), ligand **L9** (6.8 mg, 6.0 mol%), HBpin (3.2 mg, 5.0 mol%) and dry cyclohexane (2.0 mL). The reaction mixture was stirred for 2 minutes at room temperature and then 2,2'-bis((triisopropylsilyl)oxy)-1,1'-biphenyl (0.5 mmol, 249 mg) was added. The microreactor was capped with a teflon pressure cap and stirred for 24 h at room temperature. After completion (judged by TLC), CyH was removed under reduced pressure and

chromatographic separation with silica gel (2% EtOAc in hexane as eluent) gave 300 mg (80%) of the *meta*-borylated **(5ag)** product as a colourless gummy liquid.

^{1}H NMR (400 MHz, $CDCl_3$): δ 7.33 (d, J = 7.6 Hz, 2H), 7.26 (s, 3H), 7.21 (d, J = 7.6 Hz, 2H), 1.34 (s, 24H), 1.11 – 1.08 (m, 6H), 0.92 (d, J = 72 Hz, 36H).

^{13}C NMR (100 MHz, $CDCl_3$): δ 153.0, 133.6, 131.5, 126.6, 125.0, 83.6, 25.0, 18.1, 13.0.

^{11}B NMR (128 MHz, $CDCl_3$): δ 31.6.

HRMS (ESI) *m/z* calcd for $C_{42}H_{72}B_2O_6Si_2$ $[M+H]^+$ 751.5132, found 751.5150.

Same reaction was carried out with dtbpy ligand under identical conditions.

Result: Complex mixture from ^{1}H-NMR analysis.

Meta-borylation of 2,2'-bis((triisopropylsilyl)oxy)-1,1'-biphenyl **(4ag)**:

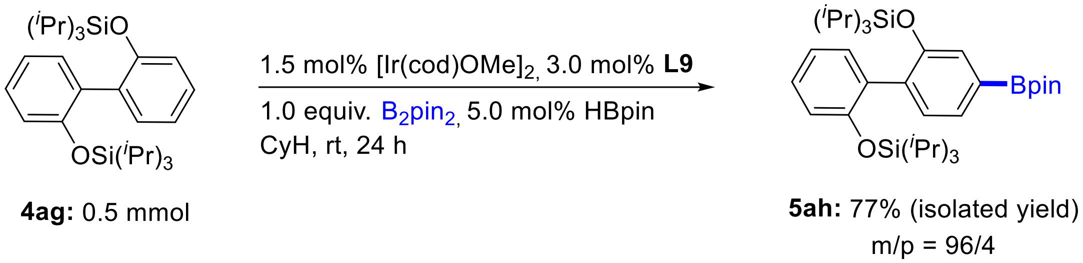

In an argon-filled glove box, a 5.0 mL Wheaton microreactor was charged with $[Ir(cod)OMe]_2$ (9.94 mg, 3 mol%), B_2pin_2 (254.0 mg, 2.0 equiv.), ligand **L9** (6.8 mg, 6.0 mol%), HBpin (3.2 mg, 5.0 mol%) and dry cyclohexane (2.0 mL). The reaction mixture was stirred for 2 minutes at room temperature and then 2,2'-bis((triisopropylsilyl)oxy)-1,1'-biphenyl (0.5 mmol, 249 mg) was added. The microreactor was capped with a teflon pressure cap and stirred for 24 h at room temperature. After completion (judged by GC-MS), CyH was removed under reduced pressure and chromatographic separation with silica gel (2% EtOAc in hexane as eluent) gave 240 mg (77%) of the *meta*-borylated **(5ah)** product as a colourless gummy liquid.

^{1}H NMR (400 MHz, $CDCl_3$): δ 7.36 – 7.33 (m, 1H), 7.27 – 7.19 (m, 4H), 7.14 (td, J = 8.0, 1.6 Hz, 1H), 6.90 (t, J = 7.2 Hz, 1H), 6.85 (d, J = 8.4 Hz, 1H), 1.35 (s, 12H), 1.13 – 1.07 (m, 6H), 0.94 – 0.91 (m, 36H).

^{13}C NMR (100 MHz, $CDCl_3$): δ 153.6, 153.1, 133.7, 132.0, 131.5, 130.6, 128.1, 126.7, 124.9, 120.2, 118.8, 83.7, 29.9, 25.0, 18.1, 18.0, 13.0.

^{11}B NMR (128 MHz, $CDCl_3$): δ 32.0.

HRMS (ESI) *m/z* calcd for $C_{36}H_{61}BO_4Si_2$ $[M+H]^+$ 625.4280, found 625.4297.

Same reaction was carried out with dtbpy ligand under identical conditions.

Result: GC-MS Conversion = 22%; m/p = 75/25.

Meta-borylation of triisopropyl(2-(1-methoxyethyl)phenoxy)silane **(4ah)***:*

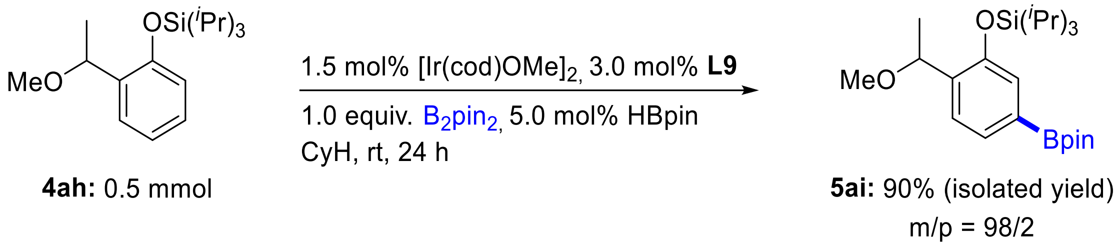

In an argon-filled glove box, a 5.0 mL Wheaton microreactor was charged with $[Ir(cod)OMe]_2$ (4.97 mg, 1.5 mol%), B_2pin_2 (127.0 mg, 1.0 equiv.), ligand **L9** (3.4 mg, 3.0 mol%), HBpin (3.2 mg, 5.0 mol%) and dry cyclohexane (2.0 mL). The reaction mixture was stirred for 2 minutes at room temperature and then triisopropyl(2-(1-methoxyethyl)phenoxy)silane (0.5 mmol, 154 mg) was added. The microreactor was capped with a teflon pressure cap and stirred for 24 h at room temperature. After completion (judged by GC-MS), CyH was removed under reduced pressure and chromatographic separation with silica gel (2% EtOAc in hexane as eluent) gave 195 mg (90%) of the *meta*-borylated **(5ai)** product as a colourless gummy liquid.

^{1}H NMR (400 MHz, $CDCl_3$): δ 7.61 (d, J = 7.2 Hz, 1H), 7.44 (d, J = 7.6 Hz, 1H), 7.23 (s, 1H), 5.32 (q, J = 5.6 Hz, 1H), 3.86 (s, 3H), 1.39 – 1.35 (m, 15H), 1.04 – 0.98 (m, 21H).

^{13}C NMR (101 MHz, $CDCl_3$): δ 154.7, 139.5, 127.7, 125.7, 115.5, 83.8, 65.1, 55.5, 25.1, 25.0, 18.2, 12.4.

^{11}B NMR (128 MHz, $CDCl_3$): δ 31.5.

HRMS (ESI) *m/z* calcd for $C_{24}H_{43}BO_4Si$ $[M+H]^+$ 435.3102, found 435.3108.

Same reaction was carried out with dtbpy ligand under identical conditions.

Result: GC-MS Conversion = 52%; m/p = 65/35.

ii) Meta-Borylation of 4-substituted arenes:

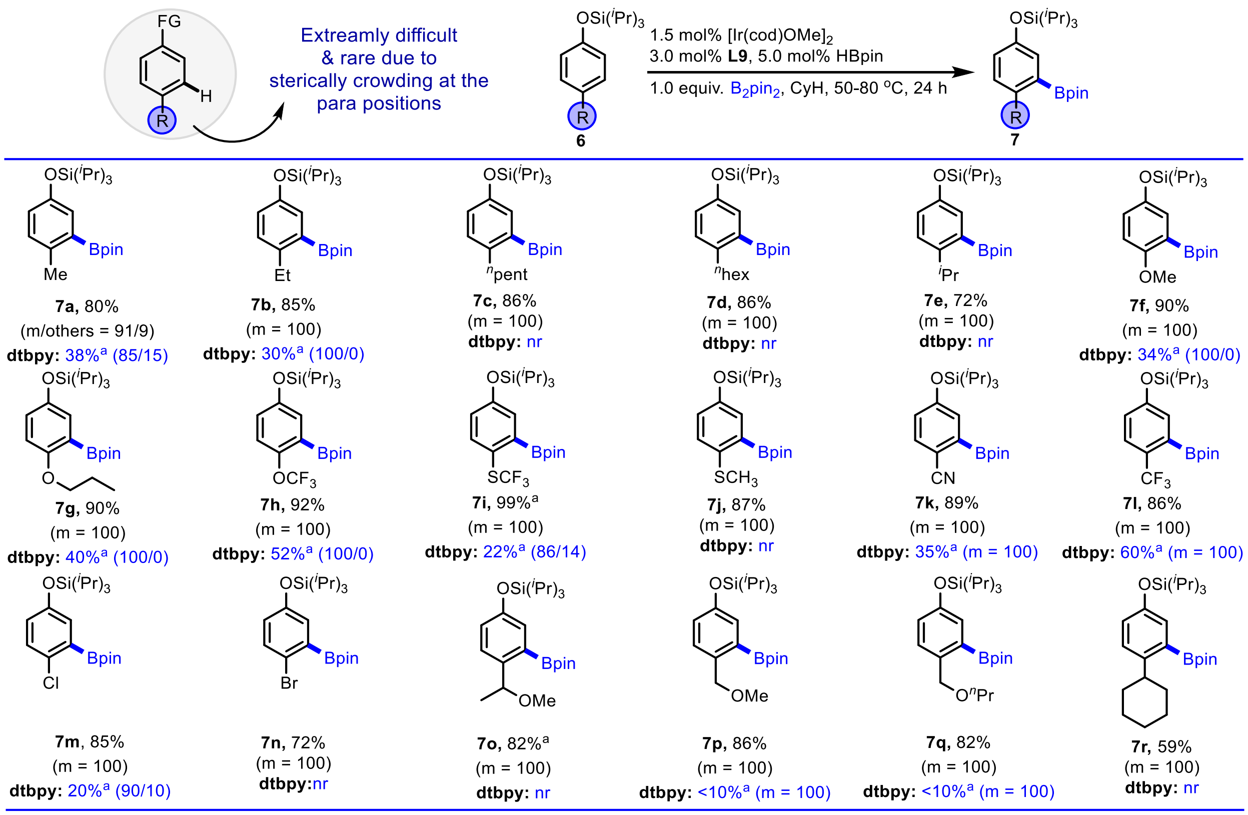

Supplementary Fig. 12: Substrates scope for 4-substituted arenes. Reactions are in 0.5 mmol scale. [a]Conversions were reported.

Meta-borylation of triisopropyl(p-tolyloxy)silane **(6a)***:*

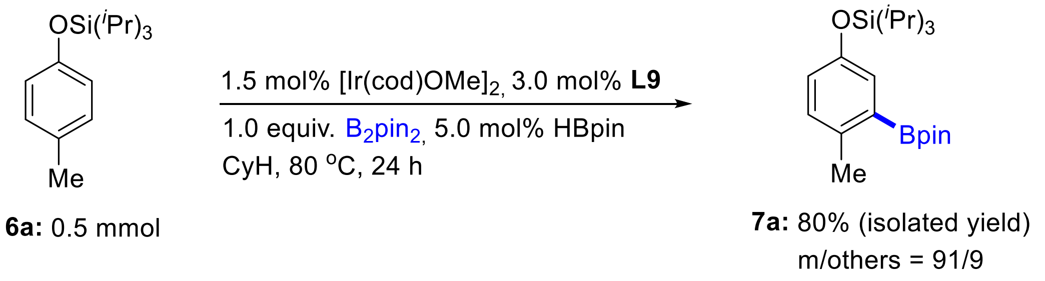

In an argon-filled glove box, a 5.0 mL Wheaton microreactor was charged with $[Ir(cod)OMe]_2$ (4.97 mg, 1.5 mol%), B_2pin_2 (127.0 mg, 1.0 equiv.), ligand **L9** (3.4 mg, 3.0 mol%), HBpin (3.2 mg, 5.0 mol%) and dry cyclohexane (2.0 mL). The reaction mixture was stirred for 2 minutes at room temperature and then triisopropyl(p-tolyloxy)silane (0.5 mmol, 132 mg) was added. The microreactor was capped with a teflon pressure cap and placed into pre-heated aluminum block at 80 °C and stirred for 24 h. After completion (judged by GC-MS), CyH was removed under reduced

pressure and chromatographic separation with neutral silica gel (2% EtOAc in hexane as eluent) gave 156 mg (80%) of the *meta*-borylated **(7a)** product as a colourless gummy liquid.

^{1}H NMR (400 MHz, $CDCl_3$): δ 7.27 (s, 1H), 7.00 (d, *J* = 8.0 Hz, 1H), 6.82 (dd, *J* = 8.4, 2.8 Hz, 1H), 2.45 (s, 3H), 1.34 (s, 12H), 1.27 – 1.24 (m, 3H), 1.10 (d, *J* = 7.2 Hz, 18H).
^{13}C NMR (100 MHz, $CDCl_3$): δ 153.3, 136.9, 130.8, 126.9, 121.9, 83.5, 25.0, 21.4, 18.1, 12.9.
^{11}B NMR (128 MHz, $CDCl_3$): δ 31.4.
HRMS (ESI) *m/z* calcd for $C_{22}H_{39}BO_3Si$ $[M+H]^+$ 391.2840, found 391.2840.
Same reaction was carried out with dtbpy ligand under identical conditions.
Result: GC-MS Conversion = 38%; m/others = 85/15.

Meta-borylation of (4-ethylphenoxy)triisopropylsilane **(6b)***:*

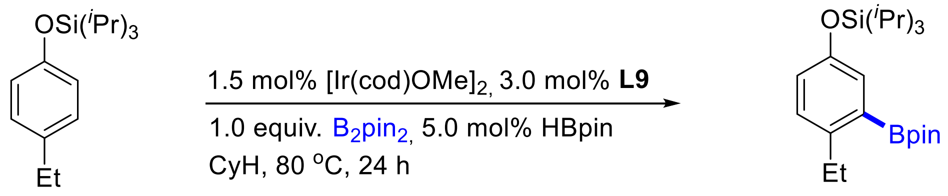

6b: 0.5 mmol

7b: 85% (isolated yield)
m = 100

In an argon-filled glove box, a 5.0 mL Wheaton microreactor was charged with $[Ir(cod)OMe]_2$ (4.97 mg, 1.5 mol%), B_2pin_2 (127.0 mg, 1.0 equiv.), ligand **L9** (3.4 mg, 3.0 mol%), HBpin (3.2 mg, 5.0 mol%) and dry cyclohexane (2.0 mL). The reaction mixture was stirred for 2 minutes at room temperature and then (4-ethylphenoxy)triisopropylsilane (0.5 mmol, 139 mg) was added. The microreactor was capped with a teflon pressure cap and placed into pre-heated aluminum block at 80 ^{o}C and stirred for 24 h. After completion (judged by GC-MS), CyH was removed under reduced pressure and chromatographic separation with neutral silica gel (3% EtOAc in hexane as eluent) gave 172 mg (85%) of the *meta*-borylated **(7b)** product as a colourless gummy liquid.

^{1}H NMR (400 MHz, $CDCl_3$) δ 7.26 (d, *J* = 2.8 Hz, 1H), 7.02 (d, *J* = 8.0 Hz, 1H), 6.84 (dd, *J* = 8.4, 2.8 Hz, 1H), 2.82 (q, *J* = 7.6 Hz, 2H), 1.33 (s, 12H), 1.25 – 1.21 (m, 3H), 1.16 (t, *J* = 7.6 Hz, 3H), 1.10 (d, *J* = 7.6 Hz, 18H).
^{13}C NMR (100 MHz, $CDCl_3$): δ 153.3, 143.7, 129.4, 127.0, 122.0, 83.4, 28.1, 25.0, 18.1, 17.5, 12.9.

^{11}B NMR (128 MHz, $CDCl_3$): δ 31.4.

HRMS (ESI) *m/z* calcd for $C_{23}H_{42}BO_3Si$ $[M+H]^+$ 405.2996, found 405.2999.

Same reaction was carried out with dtbpy ligand under identical conditions.

Result: GC-MS Conversion = 30%; meta = 100.

Meta-borylation of triisopropyl(4-pentylphenoxy)silane **(6c)**:

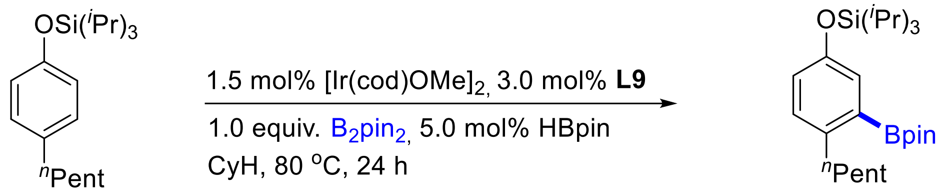

6c: 0.5 mmol

7c: 86% (isolated yield)
m = 100

In an argon-filled glove box, a 5.0 mL Wheaton microreactor was charged with $[Ir(cod)OMe]_2$ (4.97 mg, 1.5 mol%), B_2pin_2 (127.0 mg, 1.0 equiv.), ligand **L9** (3.4 mg, 3.0 mol%), HBpin (3.2 mg, 5.0 mol%) and dry cyclohexane (2.0 mL). The reaction mixture was stirred for 2 minutes at room temperature and then triisopropyl(4-pentylphenoxy)silane (0.5 mmol, 160 mg) was added. The microreactor was capped with a teflon pressure cap and placed into pre-heated aluminum block at 80 °C and stirred for 24 h. After completion (judged by GC-MS), CyH was removed under reduced pressure and chromatographic separation with neutral silica gel (5% EtOAc in hexane as eluent) gave 191 mg (86%) of the *meta*-borylated **(7c)** product as a colourless gummy liquid.

^{1}H NMR (400 MHz, $CDCl_3$) δ 7.18 (d, J = 2.8 Hz, 1H), 6.90 (d, J = 8.0 Hz, 1H), 6.74 (dd, J = 8.4, 2.8 Hz, 1H), 2.69 (t, J = 8.0 Hz, 2H), 1.46 – 1.40 (m, 2H), 1.24 (s, 15H), 1.18 – 1.13 (m, 4H), 1.01 (d, J = 7.2 Hz, 18H), 0.80 (t, J = 6.8 Hz, 3H).

^{13}C NMR (100 MHz, $CDCl_3$): δ 153.3, 142.5, 130.2, 127.0, 121.8, 83.4, 35.2, 33.5, 32.1, 25.0, 22.8, 18.1, 14.3, 12.9.

^{11}B NMR (128 MHz, $CDCl_3$): δ 31.0.

HRMS (ESI) *m/z* calcd for $C_{26}H_{47}BO_3Si$ $[M+H]^+$ 447.3466, found 447.3466.

Same reaction was carried out with dtbpy ligand under identical conditions.

Result: No reaction.

Meta-borylation of (4-hexylphenoxy)triisopropylsilane **(6d)**:

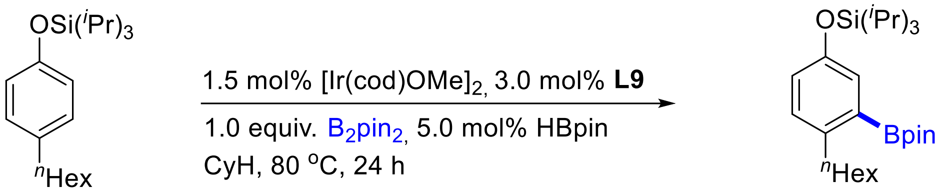

6d: 0.5 mmol

7d: 86% (isolated yield)
m = 100

In an argon-filled glove box, a 5.0 mL Wheaton microreactor was charged with $[Ir(cod)OMe]_2$ (4.97 mg, 1.5 mol%), B_2pin_2 (127.0 mg, 1.0 equiv.), ligand **L9** (3.4 mg, 3.0 mol%), HBpin (3.2 mg, 5.0 mol%) and dry cyclohexane (2.0 mL). The reaction mixture was stirred for 2 minutes at room temperature and then (4-hexylphenoxy)triisopropylsilane (0.5 mmol, 167 mg) was added. The microreactor was capped with a teflon pressure cap and placed into pre-heated aluminum block at 80 °C and stirred for 24 h. After completion (judged by GC-MS), CyH was removed under reduced pressure and chromatographic separation with neutral silica gel (2% EtOAc in hexane as eluent) gave 197 mg (86%) of the *meta*-borylated **(7d)** product as a colourless gummy liquid.

^{1}H NMR (400 MHz, $CDCl_3$): δ 7.22 (d, *J* = 2.4 Hz, 1H), 6.94 (d, *J* = 8.0 Hz, 1H), 6.78 (dd, *J* = 8.4, 2.8 Hz, 1H), 2.74 (t, *J* = 8.0 Hz, 2H), 1.48 – 1.43 (m, 2H), 1.27 (s, 15H), 1.22 – 1.17 (m, 6H), 1.06 (d, *J* = 7.2 Hz, 18H), 0.83 (t, *J* = 6.4 Hz, 3H).

^{13}C NMR (100 MHz, $CDCl_3$): δ 153.3, 142.5, 130.2, 127.0, 121.8, 83.3, 35.2, 33.8, 32.0, 29.5, 24.9, 22.8, 18.1, 14.3, 12.8.

^{11}B NMR (128 MHz, $CDCl_3$): δ 31.0.

HRMS (ESI) *m/z* calcd for $C_{27}H_{49}BO_3Si$ $[M+H]^+$ 461.3622, found 461.3630.

Same reaction was carried out with dtbpy ligand under identical conditions.

Result: No reaction.

Meta-borylation of triisopropyl(4-isopropylphenoxy)silane **(6e)**:

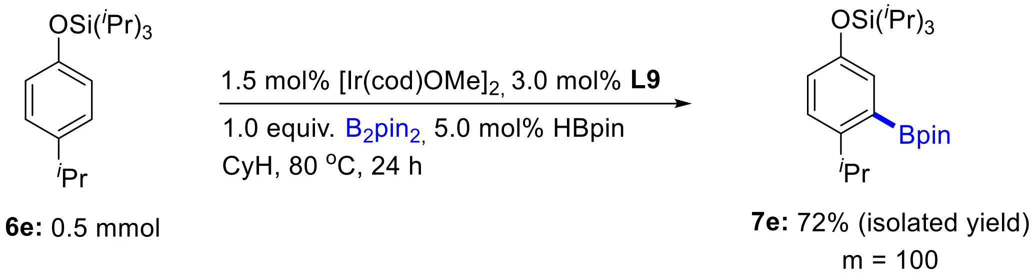

In an argon-filled glove box, a 5.0 mL Wheaton microreactor was charged with $[Ir(cod)OMe]_2$ (4.97 mg, 1.5 mol%), B_2pin_2 (127.0 mg, 1.0 equiv.), ligand **L9** (3.4 mg, 3.0 mol%), HBpin (3.2 mg, 5.0 mol%) and dry cyclohexane (2.0 mL). The reaction mixture was stirred for 2 minutes at room temperature and then triisopropyl(4-isopropylphenoxy)silane (0.5 mmol, 146 mg) was added. The microreactor was capped with a teflon pressure cap and placed into pre-heated aluminum block at 80 ^{o}C and stirred for 24 h. After completion (judged by GC-MS), CyH was removed under reduced pressure and chromatographic separation with neutral silica gel (3% EtOAc in hexane as eluent) gave 150 mg (72%) of the *meta*-borylated **(7e)** product as a colourless gummy liquid.

^{1}H NMR (400 MHz, $CDCl_3$): δ 7.22 (d, *J* = 2.4 Hz, 1H), 7.14 (d, *J* = 8.4 Hz, 1H), 6.88 (dd, *J* = 8.4, 2.4 Hz, 1H), 3.60 – 3.54 (m, 1H), 1.34 (s, 12H), 1.26 – 1.24 (m, 3H), 1.19 (d, *J* = 6.8 Hz, 6H), 1.10 (d, *J* = 7.2 Hz, 18H).

^{13}C NMR (100 MHz, $CDCl_3$): δ 153.1, 147.7, 126.5, 125.4, 121.9, 83.5, 31.0, 24.9, 24.8, 18.1, 12.8.

^{11}B NMR (128 MHz, $CDCl_3$): δ 31.7.

HRMS (ESI) *m/z* calcd for $C_{24}H_{43}BO_3Si$ $[M+H]^+$ 419.3153, found 419.3153.

Same reaction was carried out with dtbpy ligand under identical conditions.

Result: No reaction.

Meta-borylation of triisopropyl(4-methoxyphenoxy)silane **(6f)**:

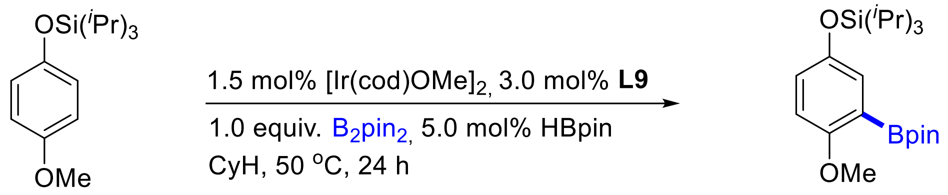

6f: 0.5 mmol

7f: 90% (isolated yield)
m = 100

In an argon-filled glove box, a 5.0 mL Wheaton microreactor was charged with [Ir(cod)OMe]$_2$ (4.97 mg, 1.5 mol%), B$_2$pin$_2$ (127.0 mg, 1.0 equiv.), ligand **L9** (3.4 mg, 3.0 mol%), HBpin (3.2 mg, 5.0 mol%) and dry cyclohexane (2.0 mL). The reaction mixture was stirred for 2 minutes at room temperature and then triisopropyl(4-methoxyphenoxy)silane (0.5 mmol, 140 mg) was added. The microreactor was capped with a teflon pressure cap and placed into pre-heated aluminum block at 50 °C and stirred for 24 h. After completion (judged by GC-MS), CyH was removed under reduced pressure and chromatographic separation with neutral silica gel (5% EtOAc in hexane as eluent) gave 183 mg (90%) of the *meta*-borylated **(7f)** product as a colourless gummy liquid.

^{1}H NMR (400 MHz, CDCl$_3$) δ 7.17 (d, *J* = 2.8 Hz, 1H), 6.87 (dd, *J* = 8.8, 3.2 Hz, 1H), 6.70 (d, *J* = 8.8 Hz, 1H), 3.77 (s, 3H), 1.34 (s, 12H), 1.25 – 1.20 (m, 3H), 1.09 (d, *J* = 7.2 Hz, 18H).
^{13}C NMR (100 MHz, CDCl$_3$): δ 158.6, 149.5, 127.5, 111.8, 83.5, 56.6, 24.9, 18.1, 12.7.
^{11}B NMR (128 MHz, CDCl$_3$): δ 30.7.
HRMS (ESI) *m/z* calcd for $C_{22}H_{39}BO_4Si$ $[M+H]^+$ 407.2789, found 407.2783.
Same reaction was carried out with dtbpy ligand under identical conditions.
Result: GC-MS Conversion = 57%; meta = 100.

Meta-borylation of triisopropyl(4-propoxyphenoxy)silane **(6g)**:

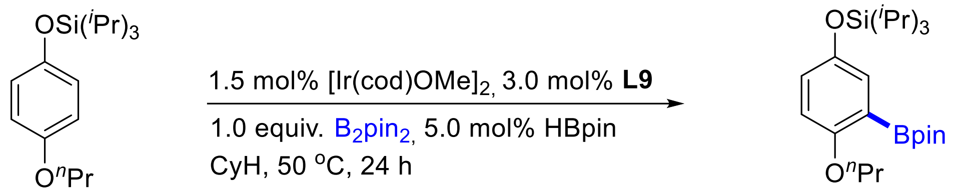

6g: 0.5 mmol

7g: 90% (isolated yield)
m = 100

In an argon-filled glove box, a 5.0 mL Wheaton microreactor was charged with [Ir(cod)OMe]$_2$ (4.97 mg, 1.5 mol%), B$_2$pin$_2$ (127.0 mg, 1.0 equiv.), ligand **L9** (3.4 mg, 3.0 mol%), HBpin (3.2

mg, 5.0 mol%) and dry cyclohexane (2.0 mL). The reaction mixture was stirred for 2 minutes at room temperature and then triisopropyl(4-propoxyphenoxy)silane (0.5 mmol, 154 mg) was added. The microreactor was capped with a teflon pressure cap and placed into pre-heated aluminum block at 50 °C and stirred for 24 h. After completion (judged by GC-MS), CyH was removed under reduced pressure and chromatographic separation with neutral silica gel (5% EtOAc in hexane as eluent) gave 195 mg (90%) of the *meta*-borylated **(7g)** product as a colourless gummy liquid.

^{1}H NMR (400 MHz, $CDCl_3$): δ 7.11 (d, *J* = 3.2 Hz, 1H), 6.84 (dd, *J* = 8.8, 3.2 Hz, 1H), 6.68 (d, *J* = 8.8 Hz, 1H), 3.85 (t, *J* = 6.4 Hz, 2H), 1.80 – 1.75 (m, 2H), 1.33 (s, 12H), 1.25 – 1.24 (m, 6H), 1.08 (d, *J* = 7.2 Hz, 18H).

^{13}C NMR (100 MHz, $CDCl_3$): δ 158.1, 149.4, 127.0, 122.7, 113.2, 83.4, 70.9, 24.9, 24.6, 18.1, 12.7,10.7.

^{11}B NMR (128 MHz, $CDCl_3$) δ 30.7.

HRMS (ESI) *m/z* calcd for $C_{24}H_{43}BO_4Si$ $[M+H]^+$ 435.3102, found 435.3093.

Same reaction was carried out with dtbpy ligand under identical conditions.

Result: GC-MS Conversion = 40%; meta = 100.

Meta-borylation of triisopropyl(4-(trifluoromethoxy)phenoxy)silane **(6h)***:*

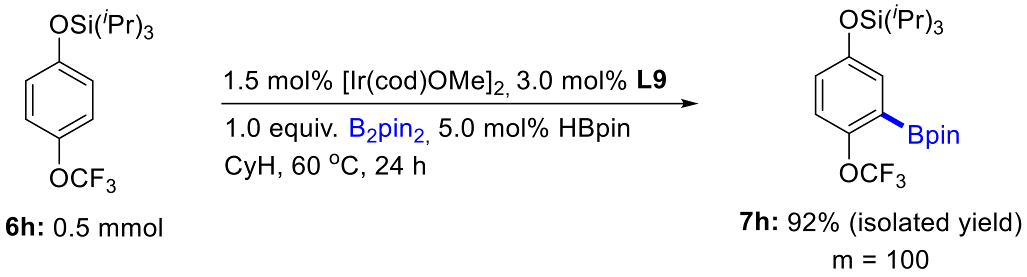

In an argon-filled glove box, a 5.0 mL Wheaton microreactor was charged with $[Ir(cod)OMe]_2$ (4.97 mg, 1.5 mol%), B_2pin_2 (127.0 mg, 1.0 equiv.), ligand **L9** (3.4 mg, 3.0 mol%), HBpin (3.2 mg, 5.0 mol%) and dry cyclohexane (2.0 mL). The reaction mixture was stirred for 2 minutes at room temperature and then triisopropyl(4-(trifluoromethoxy)phenoxy)silane (0.5 mmol, 167 mg) was added. The microreactor was capped with a teflon pressure cap and placed into pre-heated aluminum block at 60 °C and stirred for 24 h. After completion (judged by GC-MS), CyH was removed under reduced pressure and chromatographic separation with neutral silica gel (4%

EtOAc in hexane as eluent) gave 214 mg (92%) of the *meta*-borylated **(7h)** product as a colourless gummy liquid.

^{1}H NMR (400 MHz, $CDCl_3$): δ 7.21 (d, J = 3.2 Hz, 1H), 7.06 – 7.03 (m, 1H), 6.89 (dd, J = 8.8, 3.2 Hz, 1H), 1.32 (s, 12H), 1.26 – 1.22 (m, 3H), 1.08 (d, J = 7.2 Hz, 18H).

^{13}C NMR (400 MHz, $CDCl_3$) δ154.5, 147.0 (q, J = 1.9 Hz), 127.1, 122.9, 122.8, 120.7 (q, J = 253.6 Hz), 84.2, 24.8, 18.0, 12.8.

^{11}B NMR (128 MHz, $CDCl_3$): δ 30.0.

HRMS (ESI) *m/z* calcd for $C_{22}H_{36}BF_3O_4Si$ $[M+H]^+$ 461.2506, found 461.2505.

Same reaction was carried out with dtbpy ligand under identical conditions.

Result: GC-MS Conversion = 52%; meta = 100.

Meta-borylation of triisopropyl(4-((trifluoromethyl)thio)phenoxy)silane **(6i)***:*

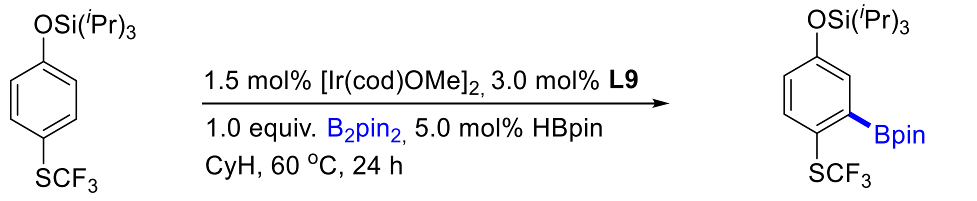

6i: 0.5 mmol

7i: 99% (conversion)
m = 100

In an argon-filled glove box, a 5.0 mL Wheaton microreactor was charged with $[Ir(cod)OMe]_2$ (4.97 mg, 1.5 mol%), B_2pin_2 (127.0 mg, 1.0 equiv.), ligand **L9** (3.4 mg, 3.0 mol%), HBpin (3.2 mg, 5.0 mol%) and dry cyclohexane (2.0 mL). The reaction mixture was stirred for 2 minutes at room temperature and then triisopropyl(4-((trifluoromethyl)thio)phenoxy)silane (0.5 mmol, 175 mg) was added. The microreactor was capped with a teflon pressure cap and placed into pre-heated aluminum block at 80 °C and stirred for 24 h. After 24 h, the crude reaction mixture was analyzed from ^{1}H-NMR and the results are shown in the above scheme.

^{1}H NMR (400 MHz, $CDCl_3$) δ 7.52 (d, J = 8.4 Hz, 1H), 7.20 (d, J = 2.8 Hz, 1H), 6.90 (dd, J = 8.4, 2.8 Hz, 1H), 1.36 (s, 12H), 1.25 – 1.23 (m, 3H), 1.09 (d, J = 7.2 Hz, 18H).

^{11}B NMR (128 MHz, $CDCl_3$): δ 31.0.

HRMS (ESI) *m/z* calcd for $C_{22}H_{36}BF_3O_3SSi$ $[M+H]^+$ 477.2278, found 477.2274.

Same reaction was carried out with dtbpy ligand under identical conditions.

Result: GC-MS Conversion = 22%; m/others = 86/14.

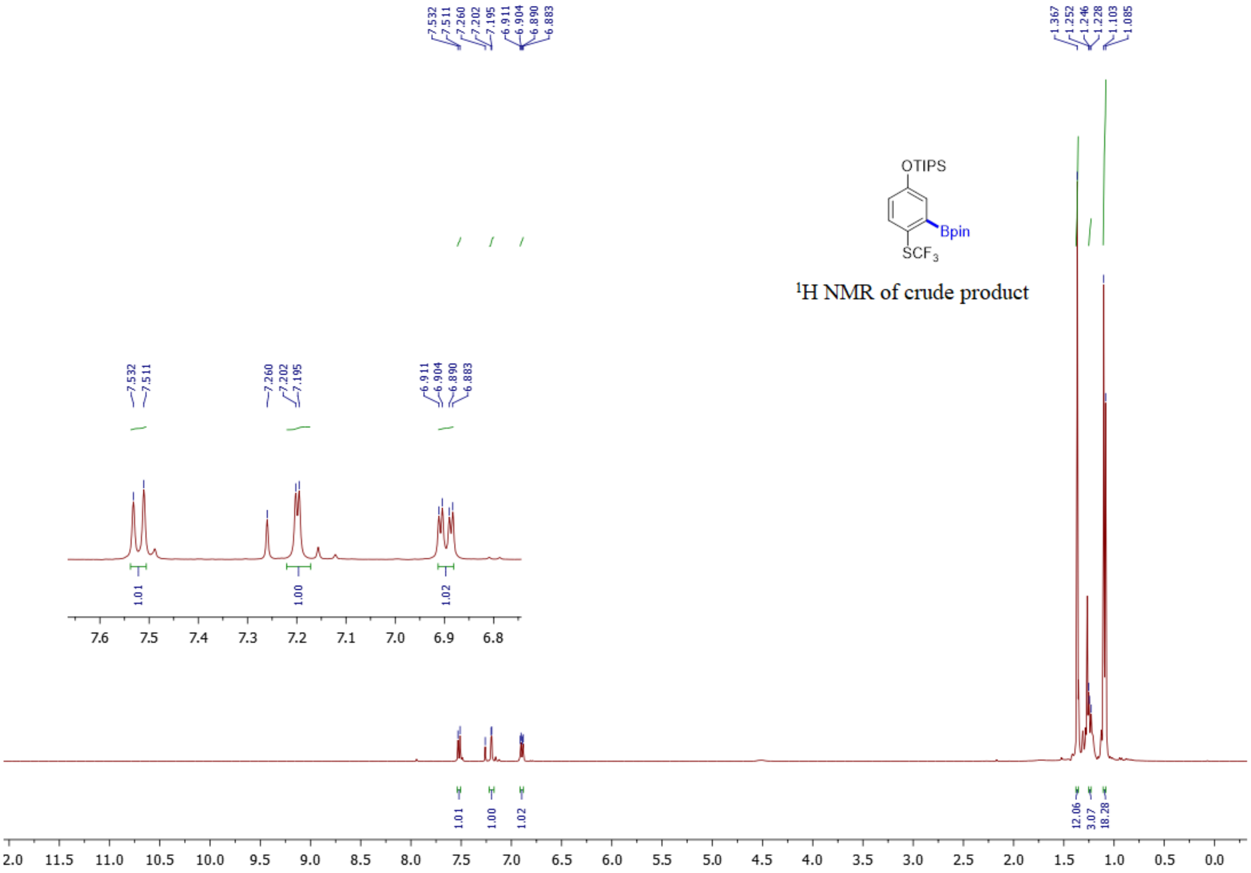

Supplementary Fig. 13: ^{1}H-NMR spectra of crude **7i** with **L9** (25 °C, 400 MHz, $CDCl_3$)

Meta-borylation of triisopropyl(4-(methylthio)phenoxy)silane **(6j)**:

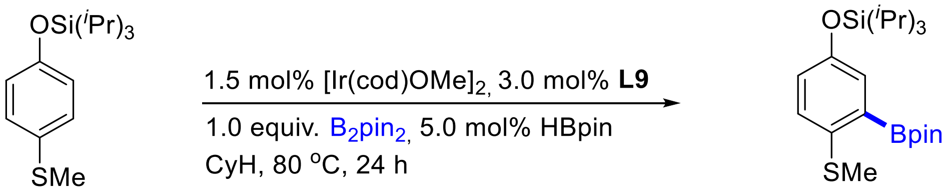

6j: 0.5 mmol

7j: 87% (isolated yield)
m = 100

In an argon-filled glove box, a 5.0 mL Wheaton microreactor was charged with $[Ir(cod)OMe]_2$ (4.97 mg, 1.5 mol%), B_2pin_2 (127.0 mg, 1.0 equiv.), ligand **L9** (3.4 mg, 3.0 mol%), HBpin (3.2 mg, 5.0 mol%) and dry cyclohexane (2.0 mL). The reaction mixture was stirred for 2 minutes at room temperature and then triisopropyl(4-(methylthio)phenoxy)silane (0.5 mmol, 148 mg) was added. The microreactor was capped with a teflon pressure cap and placed into pre-heated aluminum block at 80 °C and stirred for 24 h. After completion (judged by GC-MS), CyH was

removed under reduced pressure and chromatographic separation with neutral silica gel (5% EtOAc in hexane as eluent) gave 183 mg (87%) of the *meta*-borylated **(7j)** product as a colourless gummy liquid.

^{1}H NMR (400 MHz, CDCl$_3$): δ 7.17 (d, *J* = 2.8 Hz, 1H), 7.09 (d, *J* = 8.4 Hz, 1H), 6.87 (dd, *J* = 8.4, 2.8 Hz, 1H), 2.41 (s, 3H), 1.36 (s, 12H), 1.26 – 1.22 (m, 3H), 1.09 (d, *J* = 7.2 Hz, 18H).
^{13}C NMR (100 MHz, CDCl$_3$): δ 153.5, 135.2, 127.9, 127.0, 122.3, 84.1, 24.9, 18.1, 17.6, 12.8.
^{11}B NMR (128 MHz, CDCl$_3$): δ 30.6.
HRMS (ESI) *m/z* calcd for $C_{22}H_{39}BO_3SSi$ [M+H]$^+$ 423.2560, found 423.2543.
Same reaction was carried out with dtbpy ligand under identical conditions.
Result: GC-MS Conversion = No reaction.

Meta-borylation of 4-((triisopropylsilyl)oxy)benzonitrile **(6k)***:*

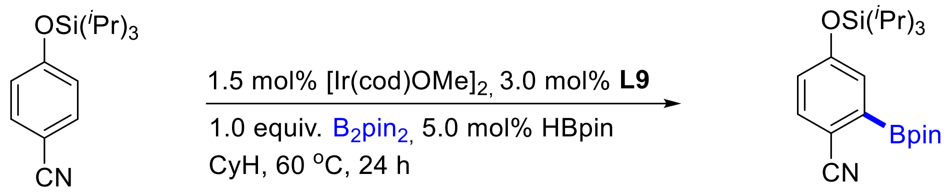

6k: 0.5 mmol

7k: 89% (isolated yield)
m = 100

In an argon-filled glove box, a 5.0 mL Wheaton microreactor was charged with [Ir(cod)OMe]$_2$ (4.97 mg, 1.5 mol%), B$_2$pin$_2$ (127.0 mg, 1.0 equiv.), ligand **L9** (3.4 mg, 3.0 mol%), HBpin (3.2 mg, 5.0 mol%) and dry cyclohexane (2.0 mL). The reaction mixture was stirred for 2 minutes at room temperature and then 4-((triisopropylsilyl)oxy)benzonitrile (0.5 mmol, 137.5 mg) was added. The microreactor was capped with a teflon pressure cap and placed into pre-heated aluminum block at 60 °C and stirred for 24 h. After completion (judged by GC-MS), CyH was removed under reduced pressure and chromatographic separation with neutral silica gel (7% EtOAc in hexane as eluent) gave 178 mg (89%) of the *meta*-borylated **(7k)** product as a colourless gummy liquid.

^{1}H NMR (400 MHz, CDCl$_3$) δ 7.57 (d, *J* = 8.4 Hz, 1H), 7.31 (d, *J* = 2.4 Hz, 1H), 6.95 (dd, *J* = 8.4, 2.8 Hz, 1H), 1.37 (s, 12H), 1.29 – 1.25 (m, 3H), 1.09 (d, *J* = 7.2 Hz, 18H).
^{13}C NMR (100 MHz, CDCl$_3$): δ 159.1, 135.5, 127.4, 122.1, 119.6, 109.0, 84.9, 24.9, 18.0, 12.8.
^{11}B NMR (128 MHz, CDCl$_3$): δ 30.5.
HRMS (ESI) *m/z* calcd for $C_{22}H_{36}BNO_3Si$ [M+H]$^+$ 402.2636, found 402.2617.

Same reaction was carried out with dtbpy ligand under identical conditions.

Result: GC-MS Conversion = 35%; meta = 100.

Meta-borylation of triisopropyl(4-(trifluoromethyl)phenoxy)silane **(6l)**:

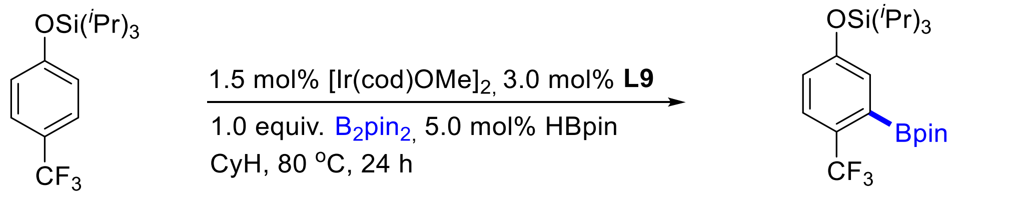

6l: 0.5 mmol

7l: 86% (isolated yield)
m = 100

In an argon-filled glove box, a 5.0 mL Wheaton microreactor was charged with $[Ir(cod)OMe]_2$ (4.97 mg, 1.5 mol%), B_2pin_2 (127.0 mg, 1.0 equiv.), ligand **L9** (3.4 mg, 3.0 mol%), HBpin (3.2 mg, 5.0 mol%) and dry cyclohexane (2.0 mL). The reaction mixture was stirred for 2 minutes at room temperature and then triisopropyl(4-(trifluoromethyl)phenoxy)silane (0.5 mmol, 159 mg) was added. The microreactor was capped with a teflon pressure cap and placed into pre-heated aluminum block at 80 °C and stirred for 24 h. After completion (judged by GC-MS), CyH was removed under reduced pressure and chromatographic separation with neutral silica gel (5% EtOAc in hexane as eluent) gave 191 mg (86%) of the *meta*-borylated **(7l)** product as a colourless gummy liquid.

^{1}H NMR (400 MHz, $CDCl_3$): δ 7.94 (s, 1H), 7.51 (d, J = 8.0 Hz, 1H), 6.83 (d, J = 8.4 Hz, 1H), 1.32 (s, 12H), 1.26 – 1.23 (m, 3H), 1.13 (d, J = 7.6 Hz, 18H).

^{13}C NMR (100 MHz, $CDCl_3$): δ 164.1, 134.6 (q, J = 3.6 Hz), 129.4 (q, J = 3.5 Hz), 124.7 (q, J = 269.5 Hz), 122.5 (q, J = 32.2 Hz), 118.9, 83.7, 25.0, 18.2, 13.4.

^{11}B NMR (128 MHz, $CDCl_3$): δ 30.1.

HRMS (ESI) *m/z* calcd for $C_{22}H_{36}BF_3O_3Si$ $[M+H]^+$ 445.2557, found 445.2538.

Same reaction was carried out with dtbpy ligand under identical conditions.

Result: GC-MS Conversion = 60%; meta = 100.

Meta-borylation of (4-chlorophenoxy)triisopropylsilane **(6m)**:

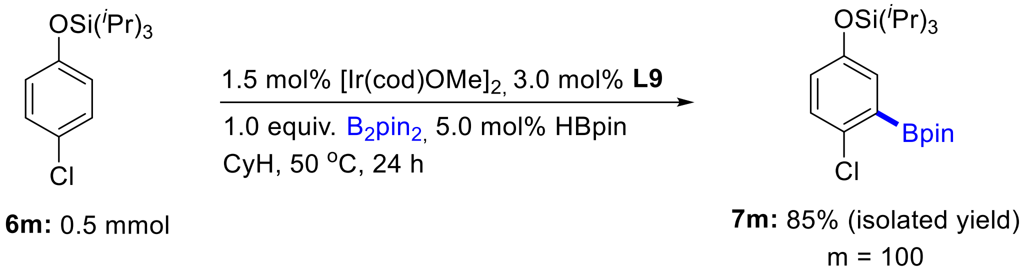

In an argon-filled glove box, a 5.0 mL Wheaton microreactor was charged with $[Ir(cod)OMe]_2$ (4.97 mg, 1.5 mol%), B_2pin_2 (127.0 mg, 1.0 equiv.), ligand **L9** (3.4 mg, 3.0 mol%), HBpin (3.2 mg, 5.0 mol%) and dry cyclohexane (2.0 mL). The reaction mixture was stirred for 2 minutes at room temperature and then (4-chlorophenoxy)triisopropylsilane (0.5 mmol, 142 mg) was added. The microreactor was capped with a teflon pressure cap and placed into pre-heated aluminum block at 50 °C and stirred for 24 h. After completion (judged by GC-MS), CyH was removed under reduced pressure and chromatographic separation with neutral silica gel (3% EtOAc in hexane as eluent) gave 174 mg (85%) of the *meta*-borylated **(7m)** product as a colourless gummy liquid.

^{1}H NMR (400 MHz, $CDCl_3$) δ 7.18 – 7.16 (m, 2H), 6.82 (dd, *J* = 8.4, 2.8 Hz, 1H), 1.36 (s, 12H), 1.26 – 1.22 (m, 3H), 1.09 (d, *J* = 7.2 Hz, 18H).
^{13}C NMR (100 MHz, $CDCl_3$): δ 154.2, 131.0, 130.3, 127.5, 123.0, 84.3, 24.9, 18.0, 12.8.
^{11}B NMR (128 MHz, $CDCl_3$): δ 30.2.
HRMS (ESI) *m/z* calcd for $C_{21}H_{36}BClO_3Si$ $[M+H]^+$ 411.2294, found 411.2295.
Same reaction was carried out with dtbpy ligand under identical conditions.
Result: GC-MS Conversion = 20%; m/others = 90/10.

Meta-borylation of (4-bromophenoxy)triisopropylsilane **(6n)**:

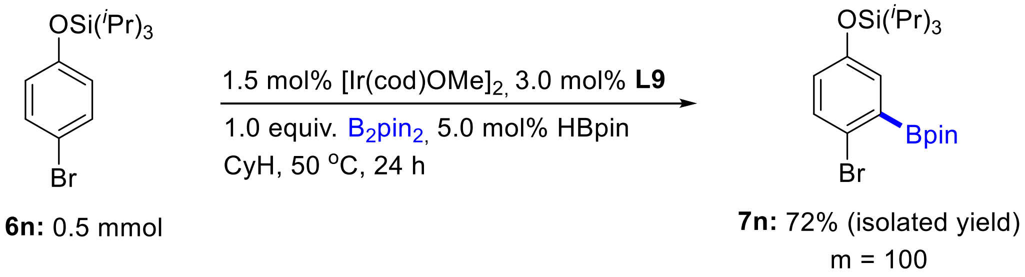

In an argon-filled glove box, a 5.0 mL Wheaton microreactor was charged with $[Ir(cod)OMe]_2$ (4.97 mg, 1.5 mol%), B_2pin_2 (127.0 mg, 1.0 equiv.), ligand **L9** (3.4 mg, 3.0 mol%), HBpin (3.2

mg, 5.0 mol%) and dry cyclohexane (2.0 mL). The reaction mixture was stirred for 2 minutes at room temperature and then (4-bromophenoxy)triisopropylsilane (0.5 mmol, 164 mg) was added. The microreactor was capped with a teflon pressure cap and placed into pre-heated aluminum block at 50 °C and stirred for 24 h. After completion (judged by GC-MS), CyH was removed under reduced pressure and chromatographic separation with neutral silica gel (3% EtOAc in hexane as eluent) gave 163 mg (72%) of the *meta*-borylated **(7n)** product as a colourless gummy liquid.

^{1}H NMR (400 MHz, $CDCl_3$): δ 7.34 (d, *J* = 8.4 Hz, 1H), 7.10 (d, *J* = 3.2 Hz, 1H), 6.75 (dd, *J* = 8.8, 3.2 Hz, 1H), 1.37 (s, 12H), 1.27 – 1.22 (m, 3H), 1.08 (d, *J* = 7.2 Hz, 18H).
^{13}C NMR (100 MHz, $CDCl_3$): δ 154.8, 133.6, 127.7, 123.3, 118.6, 84.4, 24.9, 18.0, 12.8.
^{11}B NMR (128 MHz, $CDCl_3$): δ 31.1.
HRMS (ESI) *m/z* calcd for $C_{21}H_{36}BBrO_3Si$ $[M+H]^+$ 455.1788, found 455.1789.
Same reaction was carried out with dtbpy ligand under identical conditions.
Result: GC-MS Conversion = No reaction.

Meta-borylation of triisopropyl(4-(1-methoxyethyl)phenoxy)silane **(6o)***:*

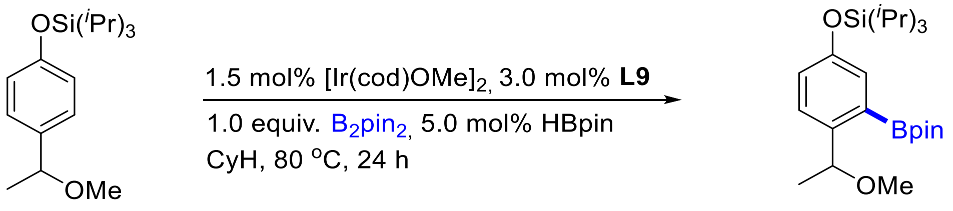

6o: 0.5 mmol

7o: 82% (conversion)
m = 100

In an argon-filled glove box, a 5.0 mL Wheaton microreactor was charged with $[Ir(cod)OMe]_2$ (4.97 mg, 1.5 mol%), B_2pin_2 (127.0 mg, 1.0 equiv.), ligand **L9** (3.4 mg, 3.0 mol%), HBpin (3.2 mg, 5.0 mol%) and dry cyclohexane (2.0 mL). The reaction mixture was stirred for 2 minutes at room temperature and then triisopropyl(4-(1-methoxyethyl)phenoxy)silane (0.5 mmol, 154 mg) was added. The microreactor was capped with a teflon pressure cap and placed into pre-heated aluminum block at 80 °C and stirred for 24 h. After 24 h, the crude reaction mixture was analyzed from ^{1}H-NMR and the results are shown in the above scheme.
^{1}H NMR (400 MHz, $CDCl_3$) δ 7.29 (d, *J* = 8.4 Hz, 1H), 7.20 (d, *J* = 2.4 Hz, 1H), 6.92 (dd, *J* = 8.4, 2.4 Hz, 1H), 4.89 (q, *J* = 6.4 Hz, 1H), 3.19 (s, 3H), 1.38 (d, *J* = 6.4 Hz, 3H), 1.34 (s, 12H), 1.24 – 1.22 (m, 3H), 1.10 (d, *J* = 7.2 Hz, 18H).

^{11}B NMR (128 MHz, $CDCl_3$): δ 30.9.

HRMS (ESI) *m/z* calcd for $C_{24}H_{43}BO_4Si$ $[M+Na]^+$ 457.2921, found 457.2919.

Same reaction was carried out with dtbpy ligand under identical conditions.

Result: No reaction.

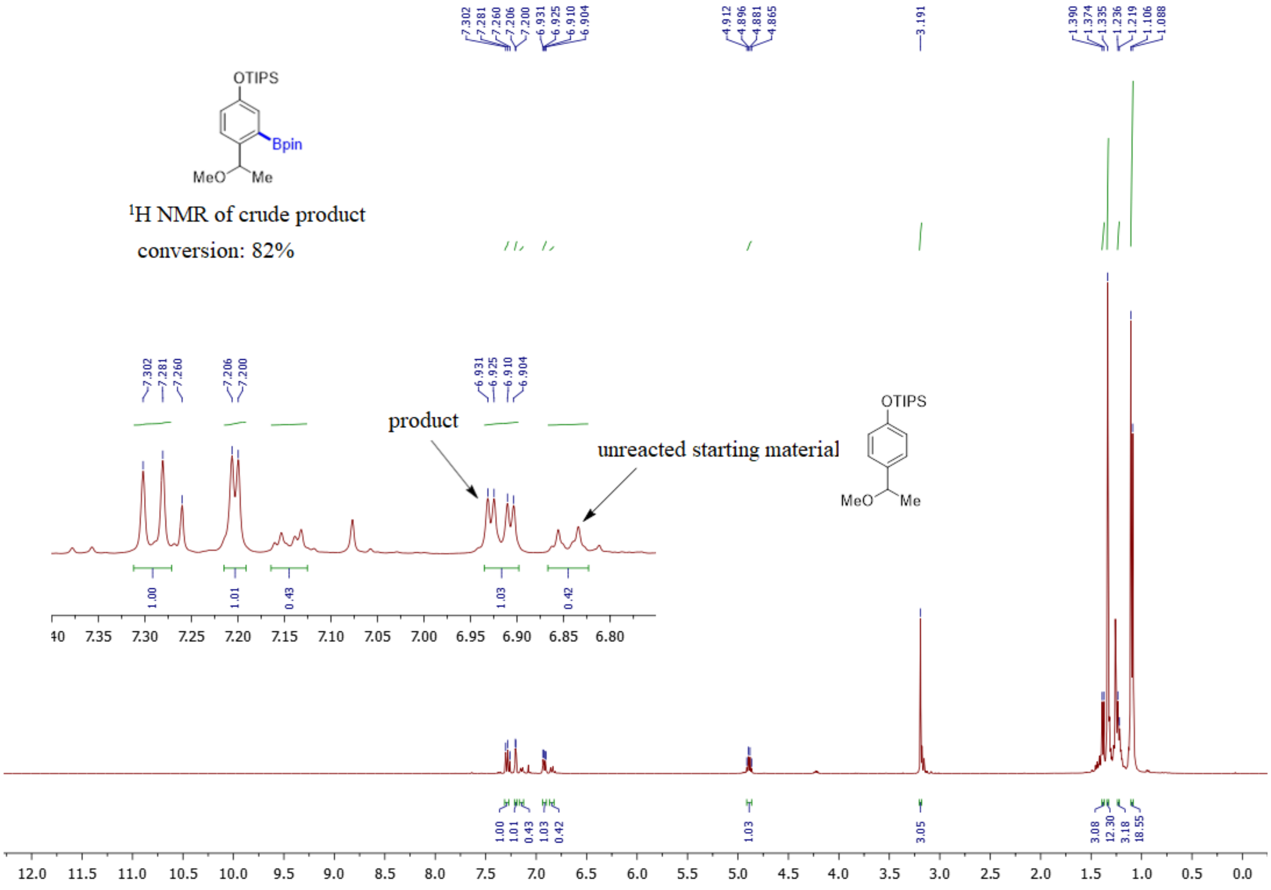

Supplementary Fig. 14: ^{1}H-NMR spectra of crude **7o** with **L9** (25 °C, 400 MHz, $CDCl_3$)

Meta-borylation of triisopropyl(4-(methoxymethyl)phenoxy)silane **(6p)***:*

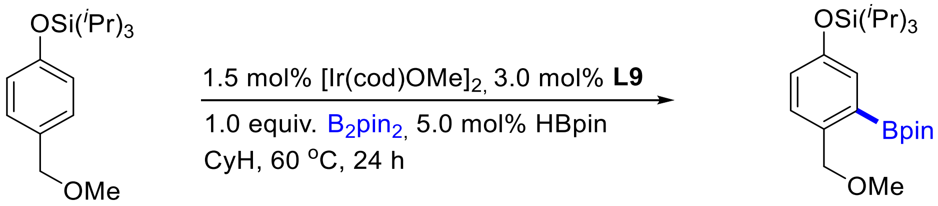

6p: 0.5 mmol

7p: 86% (isolated yield)
m = 100

In an argon-filled glove box, a 5.0 mL Wheaton microreactor was charged with $[Ir(cod)OMe]_2$ (4.97 mg, 1.5 mol%), B_2pin_2 (127.0 mg, 1.0 equiv.), ligand **L9** (3.4 mg, 3.0 mol%), HBpin (3.2 mg, 5.0 mol%) and dry cyclohexane (2.0 mL). The reaction mixture was stirred for 2 minutes at room temperature and then triisopropyl(4-(methoxymethyl)phenoxy)silane (0.5 mmol, 147 mg) was added. The microreactor was capped with a teflon pressure cap and placed into pre-heated aluminum block at 60 °C and stirred for 24 h. After completion (judged by GC-MS), CyH was removed under reduced pressure and chromatographic separation with neutral silica gel (5% EtOAc in hexane as eluent) gave 181 mg (86%) of the *meta*-borylated **(7p)** product as a colourless gummy liquid.

^1H NMR (400 MHz, $CDCl_3$): δ 7.27 (d, J = 2.8 Hz, 1H), 7.23 (d, J = 8.4 Hz, 1H), 6.89 (dd, J = 8.4, 2.8 Hz, 1H), 4.60 (s, 2H), 3.37 (s, 3H), 1.34 (s, 12H), 1.27 – 1.22 (m, 3H), 1.09 (d, J = 7.2 Hz, 18H).

^{13}C NMR (100 MHz, $CDCl_3$): δ 155.0, 136.6, 129.6, 126.8, 121.6, 83.7, 73.8, 58.0, 25.0, 18.1, 12.9.

^{11}B NMR (128 MHz, $CDCl_3$): δ 31.5.

HRMS (ESI) *m/z* calcd for $C_{23}H_{41}BO_4Si$ $[M+Na]^+$ 443.2765, found 443.2765.

Same reaction was carried out with dtbpy ligand under identical conditions.

Result: GC-MS Conversion = <10% conversion; meta = 100.

Meta-borylation of triisopropyl(4-(propoxymethyl)phenoxy)silane **(6q)***:*

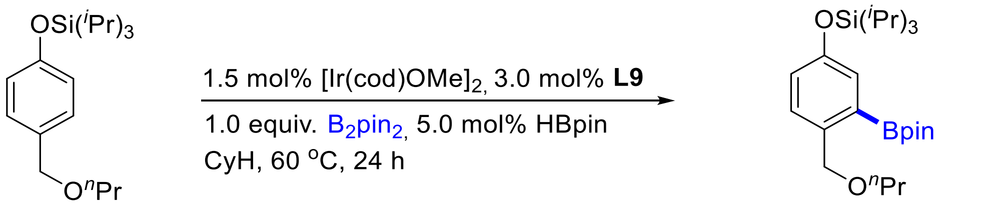

6q: 0.5 mmol

7q: 82% (isolated yield)
m = 100

In an argon-filled glove box, a 5.0 mL Wheaton microreactor was charged with $[Ir(cod)OMe]_2$ (4.97 mg, 1.5 mol%), B_2pin_2 (127.0 mg, 1.0 equiv.), ligand **L9** (3.4 mg, 3.0 mol%), HBpin (3.2 mg, 5.0 mol%) and dry cyclohexane (2.0 mL). The reaction mixture was stirred for 2 minutes at room temperature and then triisopropyl(4-(propoxymethyl)phenoxy)silane (0.5 mmol, 161 mg) was added. The microreactor was capped with a teflon pressure cap and placed into pre-heated

aluminum block at 80 °C and stirred for 24 h. After completion (judged by GC-MS), CyH was removed under reduced pressure and chromatographic separation with neutral silica gel (5% EtOAc in hexane as eluent) gave 184 mg (82%) of the *meta*-borylated **(7q)** product as a colourless gummy liquid.

^{1}H NMR (400 MHz, $CDCl_3$) δ 7.53 (d, *J* = 2.0 Hz, 1H), 7.43 (dd, *J* = 8.4, 1.6 Hz, 1H), 6.83 (d, *J* = 8.8 Hz, 1H), 4.77 (s, 2H), 3.93 (t, *J* = 6.4 Hz, 2H), 1.84 – 1.79 (m, 2H), 1.34 (s, 12H), 1.26 – 1.16 (m, 3H), 1.14 – 1.11 (m, 3H), 1.08 (d, *J* = 6.8 Hz, 18H).
^{13}C NMR (100 MHz, $CDCl_3$): δ 162.9, 134.0, 133.2, 130.1, 111.8, 83.4, 70.2, 64.8, 25.0, 22.9, 18.2, 12.2, 10.7.
^{11}B NMR (128 MHz, $CDCl_3$): δ 30.4.
HRMS (ESI) *m/z* calcd for $C_{25}H_{45}BO_4Si$ $[M+Na]^+$ 471.3078, found 471.3078.
Same reaction was carried out with dtbpy ligand under identical conditions.
Result: GC-MS Conversion = <10%; meta = 100.

Meta-borylation of (4-cyclohexylphenoxy)triisopropylsilane **(6r)***:*

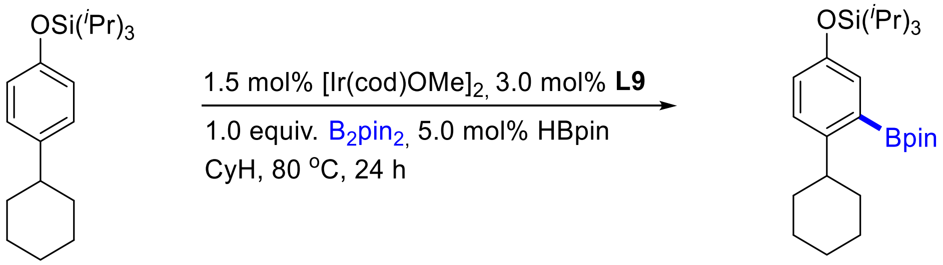

6r: 0.5 mmol

7r: 59% (isolated yield)
m = 100

In an argon-filled glove box, a 5.0 mL Wheaton microreactor was charged with $[Ir(cod)OMe]_2$ (4.97 mg, 1.5 mol%), B_2pin_2 (127.0 mg, 1.0 equiv.), ligand **L9** (3.4 mg, 3.0 mol%), HBpin (3.2 mg, 5.0 mol%) and dry cyclohexane (2.0 mL). The reaction mixture was stirred for 2 minutes at room temperature and then (4-cyclohexylphenoxy)triisopropylsilane (0.5 mmol, 166 mg) was added. The microreactor was capped with a teflon pressure cap and placed into pre-heated aluminum block at 80 °C and stirred for 24 h. After completion (judged by GC-MS), CyH was removed under reduced pressure and chromatographic separation with neutral silica gel (6% EtOAc in hexane as eluent) gave 135 mg (59%) of the *meta*-borylated **(7r)** product as a colourless gummy liquid.

^{1}H NMR (400 MHz, $CDCl_3$): δ 7.20 (s, 1H), 7.09 (d, *J* = 8.4 Hz, 1H), 6.85 (d, *J* = 6.8 Hz, 1H), 3.13 (t, *J* = 9.6 Hz, 1H), 1.82 (brs, 4H), 1.73 (d, *J* = 12.4 Hz, 1H), 1.35 (s, 12H), 1.26 – 1.24 (m, 8H), 1.10 (d, *J* = 6.8 Hz, 18H).

^{13}C NMR (100 MHz, $CDCl_3$): δ 153.1, 146.8, 126.5, 125.8, 121.6, 83.5, 41.7, 35.4, 27.4, 26.5, 24.9, 18.1, 12.8.

^{11}B NMR (128 MHz, $CDCl_3$): δ 31.5.

HRMS (ESI) *m/z* calcd for $C_{27}H_{47}BO_3Si$ $[M+H]^+$ 459.3466, found 459.3469.

Same reaction was carried out with dtbpy ligand under identical conditions.

Result: No reaction.

iii) C6-Borylation of indoles:

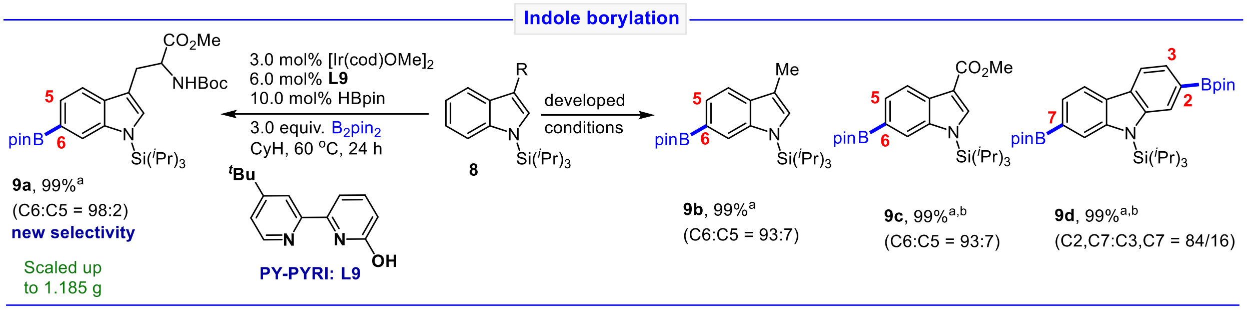

Supplementary Fig. 15: C6-Borylation of indoles. Reactions are in 0.5 mmol scale. [a]Conversions were reported. [b]2.0 equiv. B_2pin_2

C6-borylation of **8a***:*

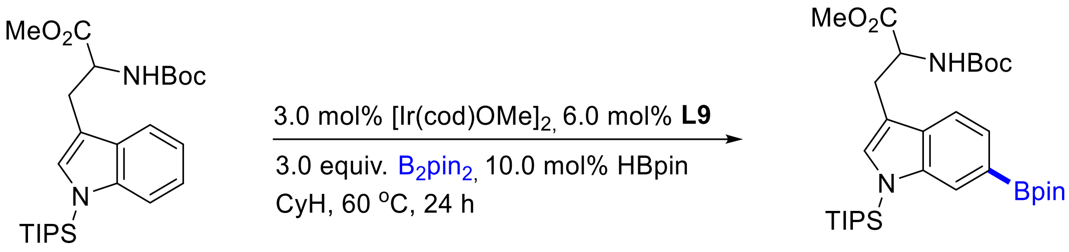

8a: 0.5 mmol

9a: 99% (conversion)
C6/C5 = 98/2

In an argon-filled glove box, a 5.0 mL Wheaton microreactor was charged with $[Ir(cod)OMe]_2$ (10 mg, 3.0 mol%), B_2pin_2 (381.0 mg, 3.0 equiv.), ligand **L9** (6.8 mg, 6.0 mol%), HBpin (6.4 mg, 10.0 mol%) and dry cyclohexane (2.0 mL). The reaction mixture was stirred for 2 minutes at room temperature and then **8a** (0.5 mmol, 237.0 mg) was added. The microreactor was capped with a

teflon pressure cap and placed into pre-heated aluminum block at 60 °C and stirred for 24 h. After 24 h, the crude reaction mixture was analyzed from ^{1}H-NMR and the results are shown in the above scheme.

<u>Gram-scale reaction</u>

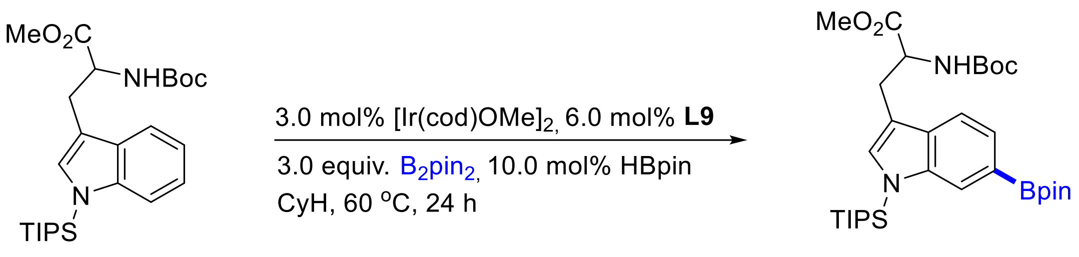

8a: 2.5 mmol

9a: 99% (conversion)
C6/C5 = 98/2

In an argon-filled glove box, a 5.0 mL Wheaton microreactor was charged with $[Ir(cod)OMe]_2$ (50.0 mg, 3.0 mol%), B_2pin_2 (1.9 g, 3.0 equiv.), ligand **L9** (34.0 mg, 6.0 mol%), HBpin (32.0 mg, 10.0 mol%) and dry cyclohexane (10.0 mL). The reaction mixture was stirred for 2 minutes at room temperature and then **8a** (0.5 mmol, 1.18 g) was added. The microreactor was capped with a teflon pressure cap and placed into pre-heated aluminum block at 60 °C and stirred for 24 h. After 24 h, the crude reaction mixture was analyzed from ^{1}H-NMR and the results are shown in the above scheme.

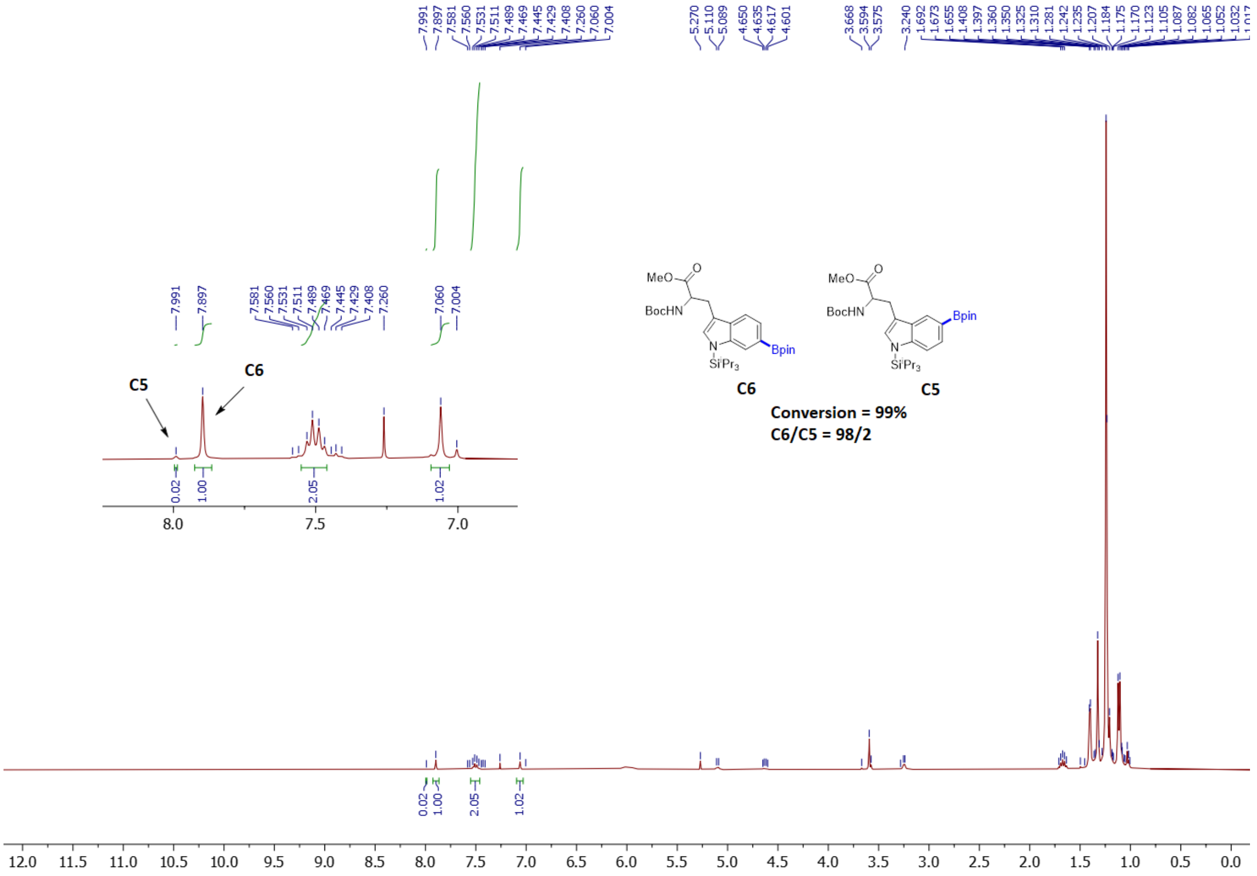

Supplementary Fig. 16: Crude ^{1}H-NMR of **9A** ($CDCl_3$, 800 MHz, 25 ^oC)

C6-borylation of **8b***:*

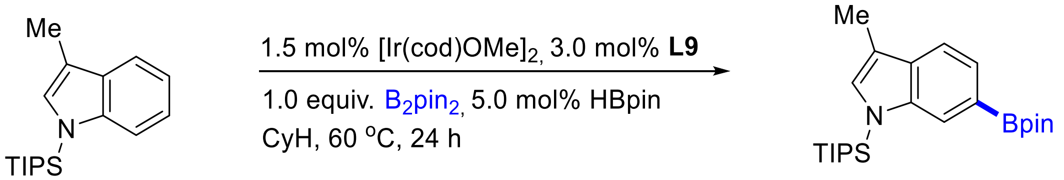

8b: 0.5 mmol

9b: 99% (conversion)
C6/C5 = 93/7

In an argon-filled glove box, a 5.0 mL Wheaton microreactor was charged with $[Ir(cod)OMe]_2$ (4.97 mg, 1.5 mol%), B_2pin_2 (127.0 mg, 1.0 equiv.), ligand **L9** (3.4 mg, 3.0 mol%), HBpin (3.2 mg, 5.0 mol%) and dry cyclohaxane (2.0 mL). The reaction mixture was stirred for 2 minutes at room temperature and then **8b** (0.5 mmol, 143.6 mg) was added. The microreactor was capped with a teflon pressure cap and placed into pre-heated aluminum block at 60 ^oC and stirred for 24 h. After 24 h, the crude reaction mixture was analyzed from ^{1}H-NMR and the results are shown in the above scheme. The spectral data are in accordance with the reported data.[35]

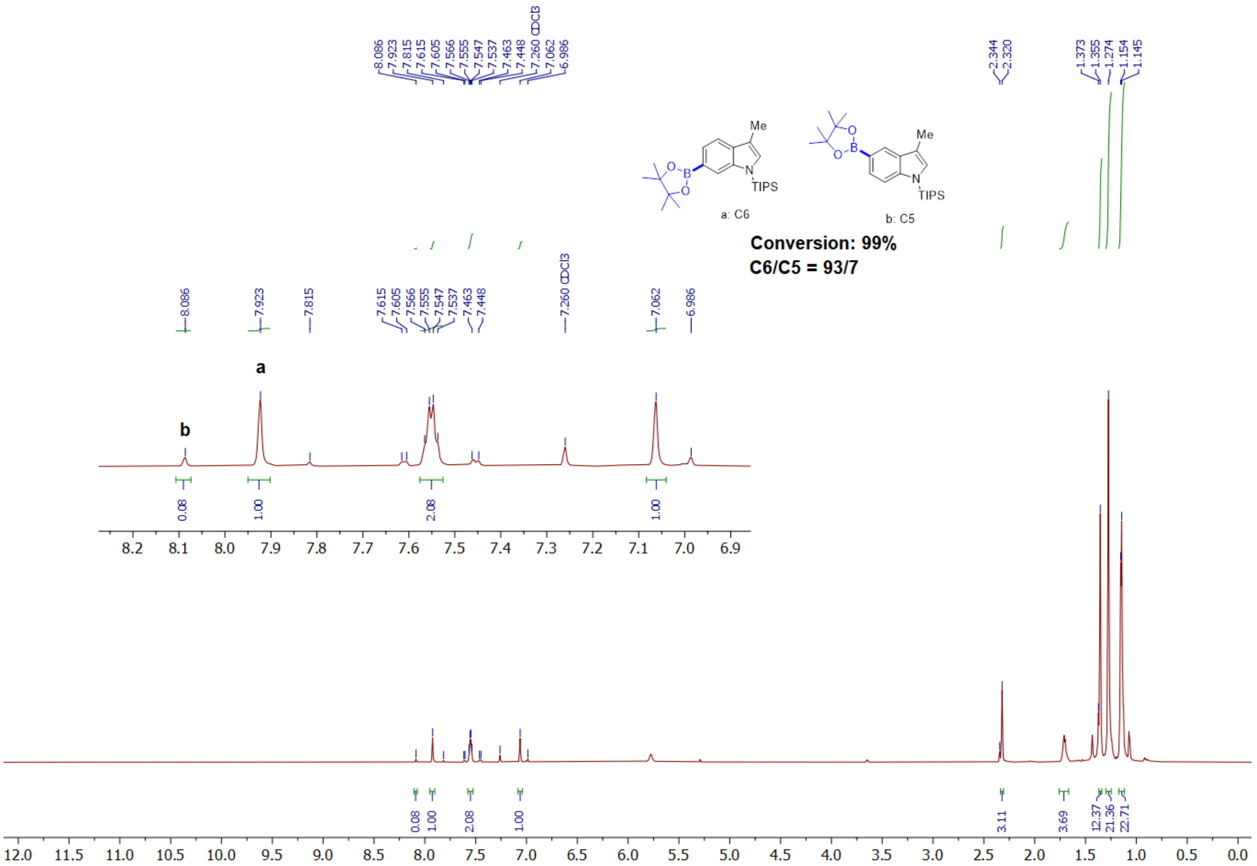

Supplementary Fig. 17: Crude ^{1}H-NMR of **9b** ($CDCl_3$, 800 MHz, 25 °C)

C6-borylation of **8c:**

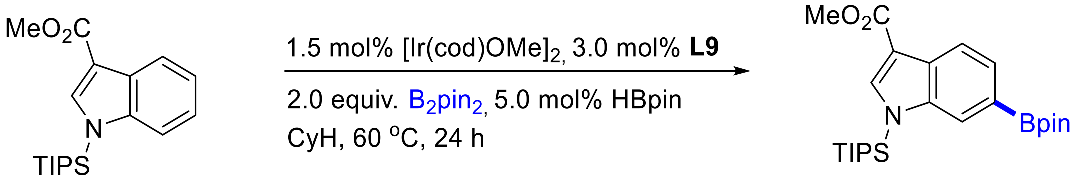

8c: 0.5 mmol

9c: 99% (conversion)
C6/C5 = 93/7

In an argon-filled glove box, a 5.0 mL Wheaton microreactor was charged with $[Ir(cod)OMe]_2$ (4.97 mg, 1.5 mol%), B_2pin_2 (254.0 mg, 2.0 equiv.), ligand **L9** (3.4 mg, 3.0 mol%), HBpin (3.2 mg, 5.0 mol%) and dry cyclohexane (2.0 mL). The reaction mixture was stirred for 2 minutes at room temperature and then **8c** (0.5 mmol, 165.6 mg) was added. The microreactor was capped with a teflon pressure cap and placed into pre-heated aluminum block at 60 °C and stirred for 24 h. After 24 h, the crude reaction mixture was analyzed from ^{1}H-NMR and the results are shown in the above scheme. The spectral data are in accordance with the reported data.[35]

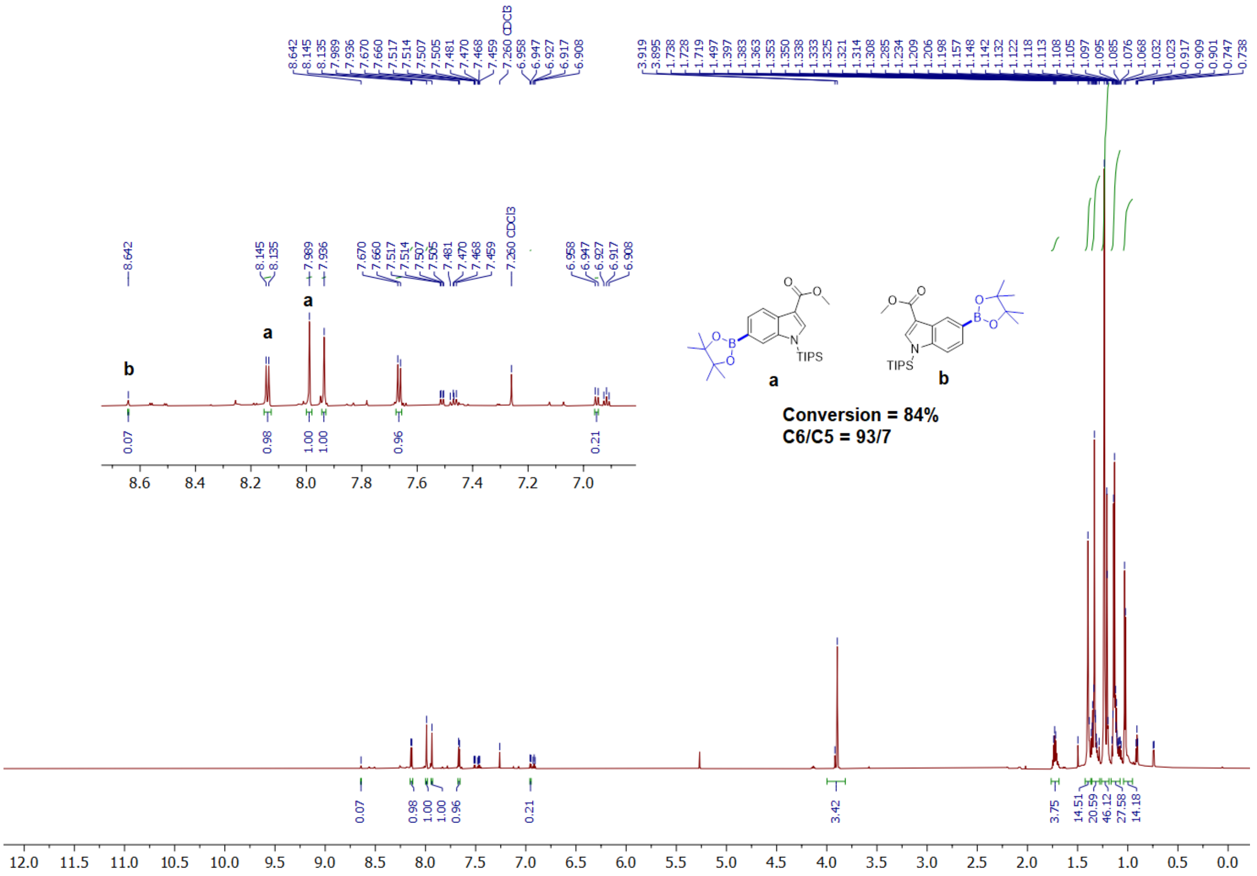

Supplementary Fig. 18: Crude ^{1}H-NMR of **9c** ($CDCl_3$, 800 MHz, 25 ^{o}C)

C2, C7-diborylation of ***8d****:*

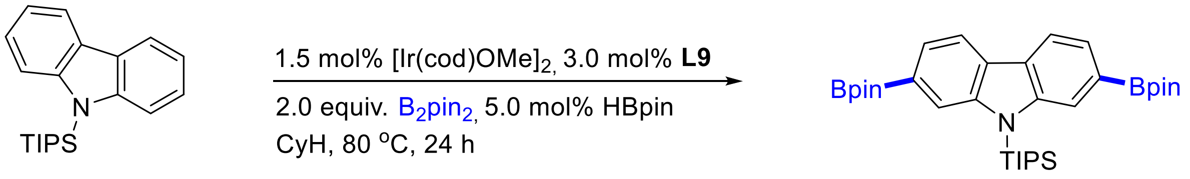

8d: 0.5 mmol

9d: 99% (conversion)
C2,C7/C3,C7 = 84/16

In an argon-filled glove box, a 5.0 mL Wheaton microreactor was charged with $[Ir(cod)OMe]_2$ (4.97 mg, 1.5 mol%), B_2pin_2 (254.0 mg, 2.0 equiv.), ligand **L9** (3.4 mg, 3.0 mol%), HBpin (3.2 mg, 5.0 mol%) and dry cyclohexane (2.0 mL). The reaction mixture was stirred for 2 minutes at room temperature and then **8d** (0.5 mmol, 161.6 mg) was added. The microreactor was capped with a teflon pressure cap and placed into pre-heated aluminum block at 80 ^{o}C and stirred for 24 h. After 24 h, the crude reaction mixture was analyzed from ^{1}H-NMR and the results are shown in the above scheme. The spectral data are in accordance with the reported data.[35]

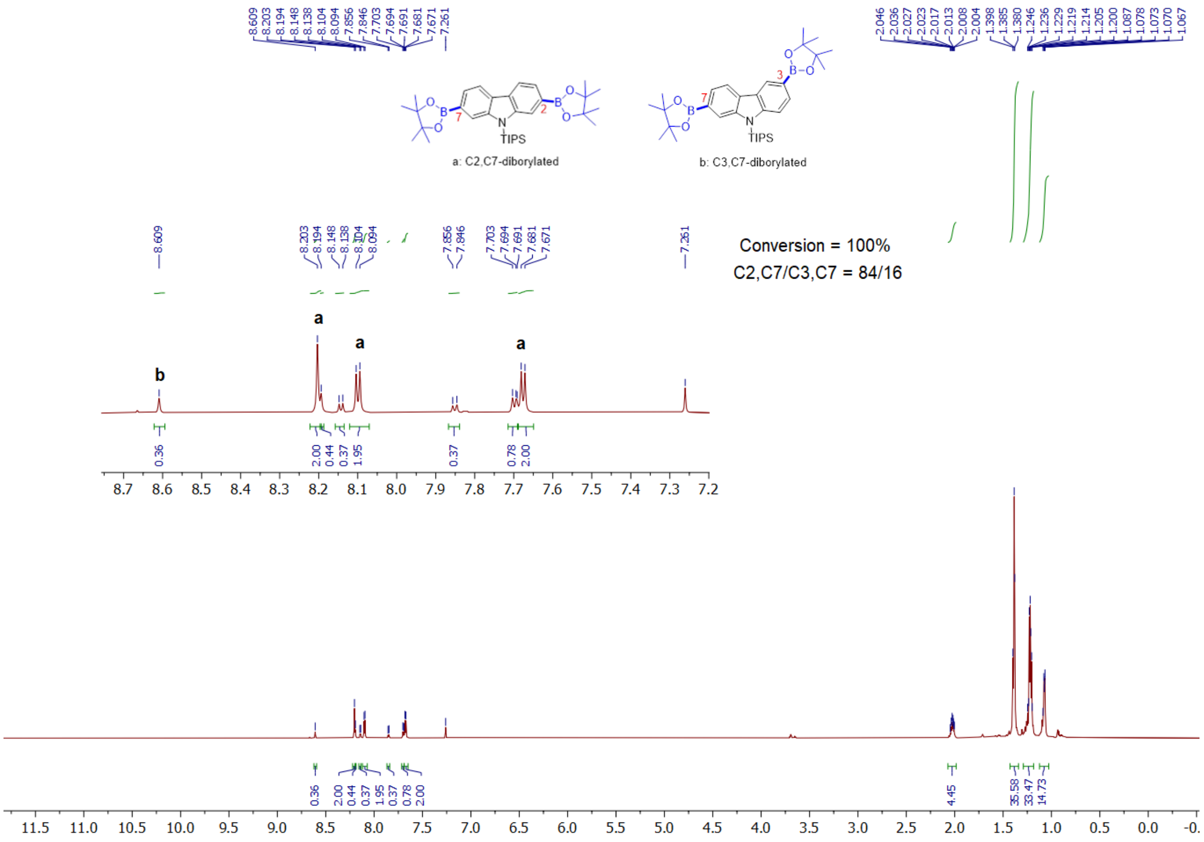

Supplementary Fig. 19: Crude ^{1}H-NMR of **9d** ($CDCl_3$, 800 MHz, 25 ^{o}C)

iv) Late-stage Meta C-H Borylation of Bio-active Molecules:

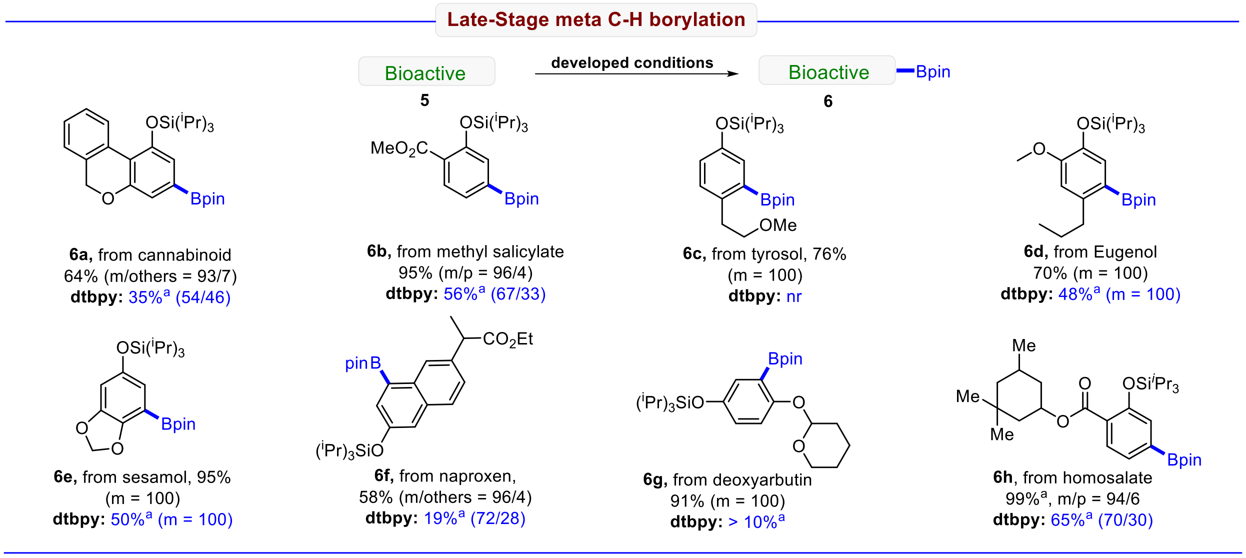

Supplementary Fig. 20: Late stage meta C-H borylation. Reactions are in 0.5 mmol scale, [a]Conversions was reported

Meta-borylation of ((6H-benzo[c]chromen-1-yl)oxy)triisopropylsilane **(10a)***:*

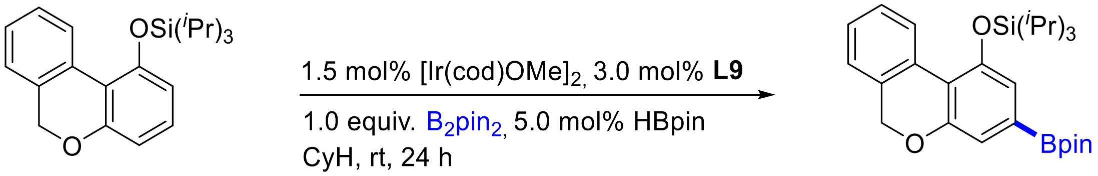

10a: 0.5 mmol

11a: 75% (isolated yield)
m/others = 93/7

In an argon-filled glove box, a 5.0 mL Wheaton microreactor was charged with $[Ir(cod)OMe]_2$ (4.97 mg, 1.5 mol%), B_2pin_2 (127.0 mg, 1.0 equiv.), ligand **L9** (3.4 mg, 3.0 mol%), HBpin (3.2 mg, 5.0 mol%) and dry cyclohexane (2.0 mL). The reaction mixture was stirred for 2 minutes at room temperature and then ((6H-benzo[c]chromen-1-yl)oxy)triisopropylsilane (0.5 mmol, 177 mg) was added. The microreactor was capped with a teflon pressure cap and placed into pre-heated aluminum block at 60 °C and stirred for 24 h. After completion (judged by GC-MS), CyH was removed under reduced pressure and chromatographic separation with silica gel (3% EtOAc in hexane as eluent) gave 177 mg (75%) of the *meta*-borylated **(11a)** product as a colourless gummy liquid.

^{1}H NMR (400 MHz, $CDCl_3$) δ 8.43 (d, *J* = 7.6 Hz, 1H), 7.32 (t, *J* = 7.2 Hz, 1H), 7.26 (t, *J* = 7.2 Hz, 1H), 7.16 (d, *J* = 7.2 Hz, 1H), 7.08 (s, 1H), 7.01 (s, 1H), 4.96 (s, 2H), 1.36 – 1.31 (m, 15H), 1.11 (d, *J* = 7.6 Hz, 18H).

^{13}C NMR (100 MHz, $CDCl_3$): δ 156.5, 153.5, 132.6, 129.4, 127.7, 127.2, 127.2, 124.4, 119.4, 117.7, 116.0, 83.9, 68.9, 25.0, 18.2, 13.3.

^{11}B NMR (128 MHz, $CDCl_3$): δ 31.7.

HRMS (ESI) *m/z* calcd for $C_{28}H_{41}BO_4Si$ $[M+H]^+$ 481.2945, found 481.2945.

Same reaction was carried out with dtbpy ligand under identical conditions.

Result: GC-MS Conversion = 35%; m/others = 54/46.

Meta-borylation of methyl 2-((triisopropylsilyl)oxy)benzoate **(10b)***:*

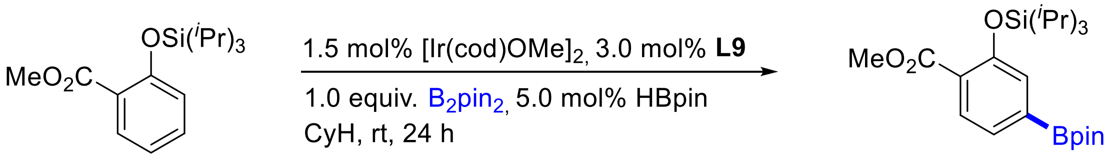

10b: 0.5 mmol

11b: 95% (isolated yield)
m/p = 96/4

In an argon-filled glove box, a 5.0 mL Wheaton microreactor was charged with $[Ir(cod)OMe]_2$ (4.97 mg, 1.5 mol%), B_2pin_2 (127.0 mg, 1.0 equiv.), ligand **L9** (3.4 mg, 3.0 mol%), HBpin (3.2 mg, 5.0 mol%) and dry cyclohexane (2.0 mL). The reaction mixture was stirred for 2 minutes at room temperature and then methyl 2-((triisopropylsilyl)oxy)benzoate (0.5 mmol, 154 mg) was added. The microreactor was capped with a teflon pressure cap and placed into pre-heated aluminum block at 40 ^{o}C and stirred for 24 h. After completion (judged by GC-MS), CyH was removed under reduced pressure and chromatographic separation with silica gel (3% EtOAc in hexane as eluent) gave 206 mg (95%) of the *meta*-borylated **(11b)** product as a colourless gummy liquid.

^{1}H NMR (400 MHz, $CDCl_3$): δ 7.68 (d, *J* = 7.6 Hz, 1H), 7.35 (d, *J* = 7.67 Hz, 1H), 7.27 (s, 1H), 3.86 (s, 3H), 1.35–1.32 (m, 15H), 1.11 (d, *J* = 7.6 Hz, 18H).

^{13}C NMR (100 MHz, $CDCl_3$): δ 167.8, 154.7, 130.7, 126.5, 126.5, 125.0, 84.1, 52.0, 25.0, 18.1, 13.2.

^{11}B NMR (128 MHz, $CDCl_3$): δ 31.3.

HRMS (ESI) *m/z* calcd for $C_{23}H_{39}BO_5Si$ $[M+H]^+$ 435.2738, found 435.2739.

Same reaction was carried out with dtbpy ligand under identical conditions.

Result: GC-MS Conversion = 56%; m/p = 67/33.

Meta-borylation of triisopropyl(4-(2-methoxyethyl)phenoxy)silane **(10c)***:*

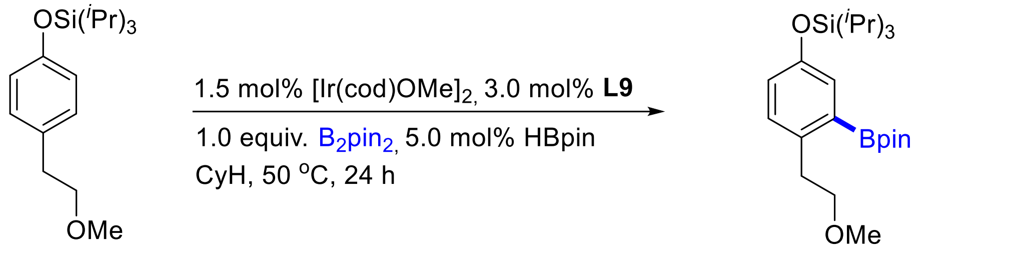

10c: 0.5 mmol

11c: 76% (isolated yield)
meta = 100

In an argon-filled glove box, a 5.0 mL Wheaton microreactor was charged with $[Ir(cod)OMe]_2$ (4.97 mg, 1.5 mol%), B_2pin_2 (127.0 mg, 1.0 equiv.), ligand **L9** (3.4 mg, 3.0 mol%), HBpin (3.2 mg, 5.0 mol%) and dry cyclohexane (2.0 mL). The reaction mixture was stirred for 2 minutes at room temperature and then triisopropyl(4-(2-methoxyethyl)phenoxy)silane (0.5 mmol, 154 mg) was added. The microreactor was capped with a teflon pressure cap and placed into pre-heated aluminum block at 80 ^{o}C and stirred for 24 h. After completion (judged by GC-MS), CyH was removed under reduced pressure and chromatographic separation with neutral silica gel (5% EtOAc in hexane as eluent) gave 165 mg (76%) of the *meta*-borylated **(11c)** product as a colourless gummy liquid.

^{1}H NMR (400 MHz, $CDCl_3$): δ 7.21 (s, 1H), 6.97 (d, J = 7.2 Hz, 1H), 6.77 (d, J = 6.4 Hz, 1H), 3.44 (br, 2H), 3.28 (s, 3H), 3.02 (br, 2H), 1.26 (s, 12H), 1.18 – 1.16 (m, 3H), 1.02 (d, J = 6.0 Hz, 18H).

^{13}C NMR (100 MHz, $CDCl_3$): δ 153.8, 137.5, 131.0, 127.2, 122.0, 83.6, 75.2, 58.6, 35.2, 25.0, 18.1, 12.8.

^{11}B NMR (128 MHz, $CDCl_3$): δ 30.7.

HRMS (ESI) *m/z* calcd for $C_{24}H_{43}BO_4Si$ $[M+H]^+$ 435.3102, found 435.3084.

Same reaction was carried out with dtbpy ligand under identical conditions.

Result: No reaction.

Meta-borylation of triisopropyl(2-methoxy-4-propylphenoxy)silane **(10d)**:

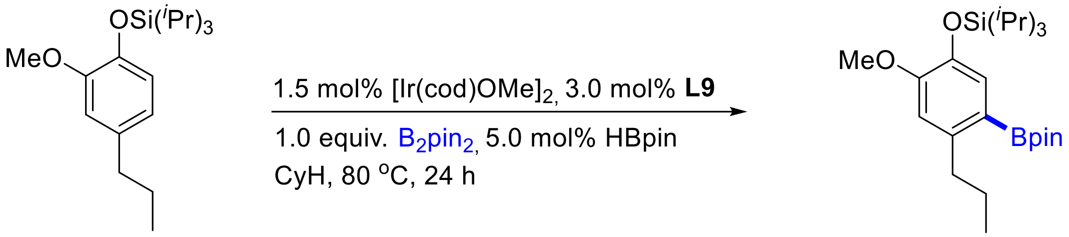

10d: 0.5 mmol

11d: 70% (isolated yield)
meta = 100

In an argon-filled glove box, a 5.0 mL Wheaton microreactor was charged with $[Ir(cod)OMe]_2$ (4.97 mg, 1.5 mol%), B_2pin_2 (127.0 mg, 1.0 equiv.), ligand **L9** (3.4 mg, 3.0 mol%), and dry cyclohexane (2.0 mL). The reaction mixture was stirred for 2 minutes at room temperature and then triisopropyl(2-methoxy-4-propylphenoxy)silane (0.5 mmol, 161 mg) was added. The microreactor was capped with a teflon pressure cap and placed into pre-heated aluminum block at 80 °C and stirred for 24 h. After completion (judged by GC-MS), CyH was removed under reduced pressure and chromatographic separation with neutral silica gel (5% EtOAc in hexane as eluent) gave 156 mg (70%) of the *meta*-borylated **(11d)** product as a colourless gummy liquid.

^{1}H NMR (400 MHz, $CDCl_3$): δ 7.26 (s, 1H), 6.62 (s, 1H), 3.79 (s, 3H), 2.78 (t, J = 7.6 Hz, 2H), 1.60 – 1.52 (m, 3H), 1.31 (s, 12H), 1.26 – 1.22 (m, 6H), 1.09 (d, J = 7.2 Hz, 18H), 0.91 (t, J = 7.2 Hz, 3H).

^{13}C NMR (100 MHz, $CDCl_3$): δ 152.6, 1443, 142.6, 127.7, 113.1, 83.1, 55.2, 37.6, 26.8, 25.0, 18.1, 14.1, 13.1.

^{11}B NMR (128 MHz, $CDCl_3$): δ 31.7.

HRMS (ESI) *m/z* calcd for $C_{25}H_{45}BO_4Si$ $[M+H]^+$ 449.3258, found 449.3250.

Same reaction was carried out with dtbpy ligand under identical conditions.

Result: GC-MS Conversion = 48%; meta = 100.

Meta-borylation of (benzo[d][1,3]dioxol-5-yloxy)triisopropylsilane **(10e)***:*

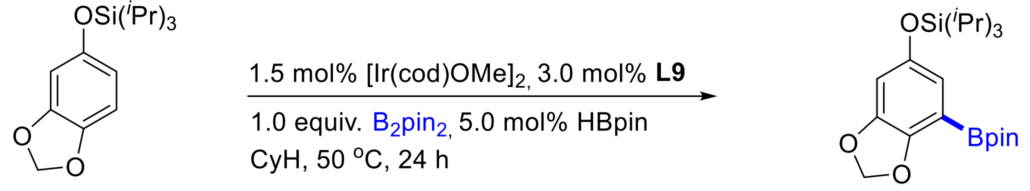

10e: 0.5 mmol

11e: 95% (isolated yield)
meta = 100

In an argon-filled glove box, a 5.0 mL Wheaton microreactor was charged with $[Ir(cod)OMe]_2$ (4.97 mg, 1.5 mol%), B_2pin_2 (127.0 mg, 1.0 equiv.), ligand **L9** (3.4 mg, 3.0 mol%), HBpin (3.2 mg, 5.0 mol%) and dry cyclohexane (2.0 mL). The reaction mixture was stirred for 2 minutes at room temperature and then (benzo[d][1,3]dioxol-5-yloxy)triisopropylsilane (0.5 mmol, 147 mg) was added. The microreactor was capped with a teflon pressure cap and placed into pre-heated aluminum block at 80 °C and stirred for 24 h. After completion (judged by GC-MS), CyH was removed under reduced pressure and chromatographic separation with neutral silica gel (5% EtOAc in hexane as eluent) gave 199 mg (95%) of the *meta*-borylated **(11e)** product as a colourless gummy liquid.

^{1}H NMR (400 MHz, $CDCl_3$): δ 6.68 (d, *J* = 2.0 Hz, 1H), 6.50 (d, *J* = 2.0 Hz, 1H), 5.97 (s, 2H), 1.35 (s, 12H), 1.25 – 1.20 (m, 3H), 1.09 (d, *J* = 7.2 Hz, 18H).

^{13}C NMR (100 MHz, $CDCl_3$): δ 150.4, 147.6, 146.9, 116.9, 104.9, 101.4, 84.0, 25.0, 18.1, 12.7.

^{11}B NMR (128 MHz, $CDCl_3$): δ 30.7.

HRMS (ESI) *m/z* calcd for $C_{22}H_{37}BO_5Si$ $[M+H]^+$ 421.2582, found 421.2573.

Same reaction was carried out with dtbpy ligand under identical conditions.

Result: GC-MS Conversion = 50%; meta = 100.

Meta-borylation of 2-(6-((triisopropylsilyl)oxy)naphthalen-2-yl)propanoate **(10f):**

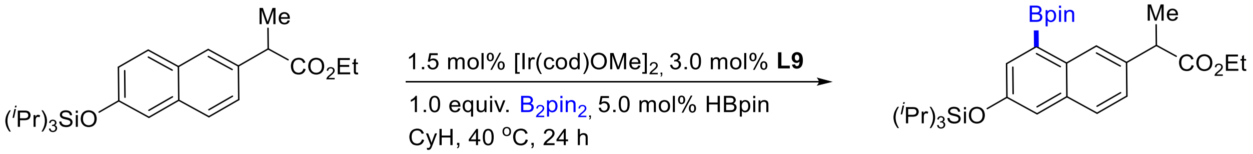

10f: 0.5 mmol

11f: 58% (isolated yield)
m/others = 96/4

In an argon-filled glove box, a 5.0 mL Wheaton microreactor was charged with $[Ir(cod)OMe]_2$ (4.97 mg, 1.5 mol%), B_2pin_2 (127.0 mg, 1.0 equiv.), ligand **L9** (3.4 mg, 3.0 mol%), HBpin (3.2

mg, 5.0 mol%) and dry cyclohexane (2.0 mL). The reaction mixture was stirred for 2 minutes at room temperature and then 2-(6-((triisopropylsilyl)oxy)naphthalen-2-yl)propanoate (0.5 mmol, 200 mg) was added. The microreactor was capped with a teflon pressure cap and placed into pre-heated aluminum block at 60 °C and stirred for 24 h. After completion (judged by GC-MS), CyH was removed under reduced pressure and chromatographic separation with silica gel (2% EtOAc in hexane as eluent) gave 152 mg (58%) of the *meta*-borylated **(11f)** product as a colourless gummy liquid.

^{1}H NMR (400 MHz, $CDCl_3$): δ 8.55 (s, 1H), 7.67 (d, *J* = 2.4 Hz, 1H), 7.63 (d, *J* = 8.4 Hz, 1H), 7.38 (d, *J* = 8.0 Hz, 1H), 7.24 (d, *J* = 2.0 Hz, 1H), 4.18 – 4.11 (m, 2H), 3.88 (q, *J* = 6.8 Hz, 1H), 1.58 (d, *J* = 7.2 Hz, 3H), 1.42 (s, 12H), 1.32 (q, *J* = 7.2 Hz, 3H), 1.26 – 1.23 (m, 3H), 1.13 (d, *J* = 7.2 Hz, 18H).

^{13}C NMR (100 MHz, $CDCl_3$): δ 175.0, 153.1, 136.2, 134.0, 132.6, 131.5, 127.4, 126.7, 125.7, 117.3, 83.9, 60.8, 45.9, 25.1, 25.1, 18.2, 14.3, 12.9.

^{11}B NMR (128 MHz, $CDCl_3$): δ 32.1.

HRMS (ESI) *m/z* calcd for $C_{30}H_{47}BO_5Si$ $[M+H]^+$ 527.3364, found 527.3364.

Same reaction was carried out with dtbpy ligand under identical conditions.

Result: GC-MS Conversion = 58%; m/others = 72/28.

Meta-borylation of triisopropyl(4-((tetrahydro-2H-pyran-2-yl)oxy)phenoxy)silane **(10g)***:*

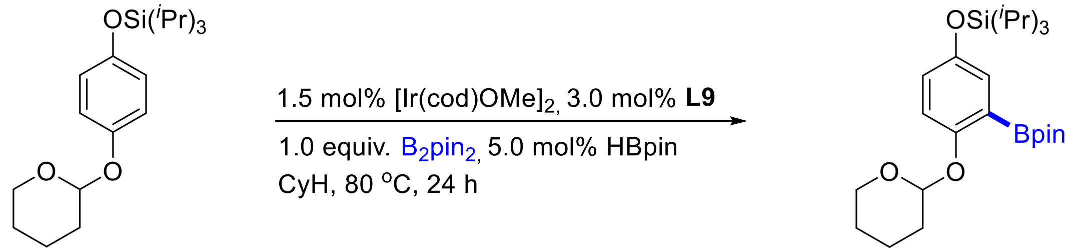

10g: 0.5 mmol

11g: 91% (isolated yield)
meta = 100

In an argon-filled glove box, a 5.0 mL Wheaton microreactor was charged with $[Ir(cod)OMe]_2$ (4.97 mg, 1.5 mol%), B_2pin_2 (127.0 mg, 1.0 equiv.), ligand **L9** (3.4 mg, 3.0 mol%), HBpin (3.2 mg, 5.0 mol%) and dry cyclohexane (2.0 mL). The reaction mixture was stirred for 2 minutes at room temperature and then triisopropyl(4-((tetrahydro-2H-pyran-2-yl)oxy)phenoxy)silane (0.5 mmol, 175 mg) was added. The microreactor was capped with a teflon pressure cap and placed into pre-heated aluminum block at 80 °C and stirred for 24 h. After completion (judged by GC-

MS), CyH was removed under reduced pressure and chromatographic separation with neutral silica gel (5% EtOAc in hexane as eluent) gave 216 mg (91%) of the *meta*-borylated **(11g)** product as a colourless gummy liquid.

^{1}H NMR (400 MHz, $CDCl_3$): δ 7.15 (d, J = 2.8 Hz, 1H), 6.94 (d, J = 8.8 Hz, 1H), 6.85 (dd, J = 8.8, 3.2 Hz, 1H), 5.30 (s, 1H), 4.04 – 3.98 (m, 1H), 3.57 – 3.54 (m, 1H), 2.17 – 2.08 (m, 1H), 1.96 – 1.92 (m, 1H), 1.86 – 1.80 (m, 1H), 1.70 – 1.60 (m, 3H), 1.33 (s, 12H), 1.25 – 1.19 (m, 3H), 1.08 (d, J = 7.2 Hz, 18H).
^{13}C NMR (100 MHz, $CDCl_3$): δ 155.8, 150.6, 126.9, 122.9, 117.9, 98.0, 83.5, 61.7, 30.5, 25.6, 25.0, 25.0, 18.1, 12.8.
^{11}B NMR (128 MHz, $CDCl_3$): δ 30.9.
HRMS (ESI) *m/z* calcd for $C_{26}H_{45}BO_5Si$ $[M+Na]^+$ 499.3027, found 499.3027.
Same reaction was carried out with dtbpy ligand under identical conditions.
Result: GC-MS Conversion = <10%.

Meta-borylation of 3,3,5-trimethylcyclohexyl 2-((triisopropylsilyl)oxy)benzoate **(10h)***:*

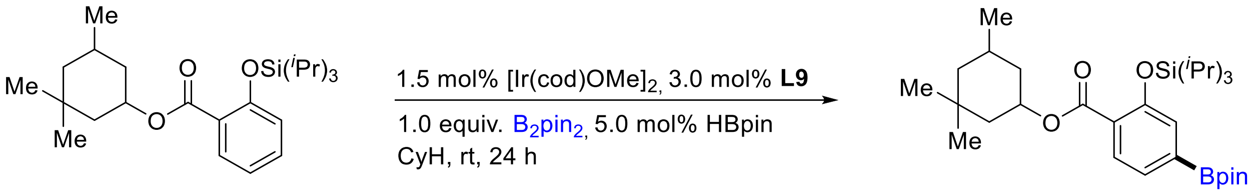

10h: 0.5 mmol

11h: 99% (conversion)
m/p = 94/6

In an argon-filled glove box, a 5.0 mL Wheaton microreactor was charged with $[Ir(cod)OMe]_2$ (4.97 mg, 1.5 mol%), B_2pin_2 (127.0 mg, 1.0 equiv.), ligand **L9** (3.4 mg, 3.0 mol%), HBpin (3.2 mg, 5.0 mol%) and dry cyclohexane (2.0 mL). The reaction mixture was stirred for 2 minutes at room temperature and then 3,3,5-trimethylcyclohexyl 2-((triisopropylsilyl)oxy)benzoate (0.5 mmol, 209 mg) was added. The microreactor was capped with a teflon pressure cap and placed into pre-heated aluminum block at 40 °C and stirred for 24 h. After 24 h, the crude reaction mixture was analyzed from ^{1}H-NMR and the results are shown in the above scheme.

^{1}H NMR (400 MHz, $CDCl_3$): 7.55 (d, J = 7.6 Hz, 1H), 7.32 (d, J = 7.6 Hz, 1H), 7.26 (s, 1H), 5.13 – 5.05 (m, J = 11.4, 5.6 Hz, 1H), 2.12 (d, J = 11.2 Hz, 1H), 1.77 (d, J = 8.8 Hz, 2H), 1.40 – 1.29

(m, 18H), 1.11 (d, *J* = 7.6 Hz, 18H), 0.97 (d, *J* = 6.8 Hz, 6H), 0.92 (d, *J* = 6.4 Hz, 3H), 0.81 (t, *J* = 12.8 Hz, 1H).

^{11}B NMR (128 MHz, $CDCl_3$): δ 30.8.

HRMS (ESI) *m/z* calcd for $C_{31}H_{53}BO_5Si$ $[M+H]^+$ 545.3834, found 545.3840.

Same reaction was carried out with dtbpy ligand under identical conditions.

Result: GC-MS Conversion = 65%; m/p = 70/30.

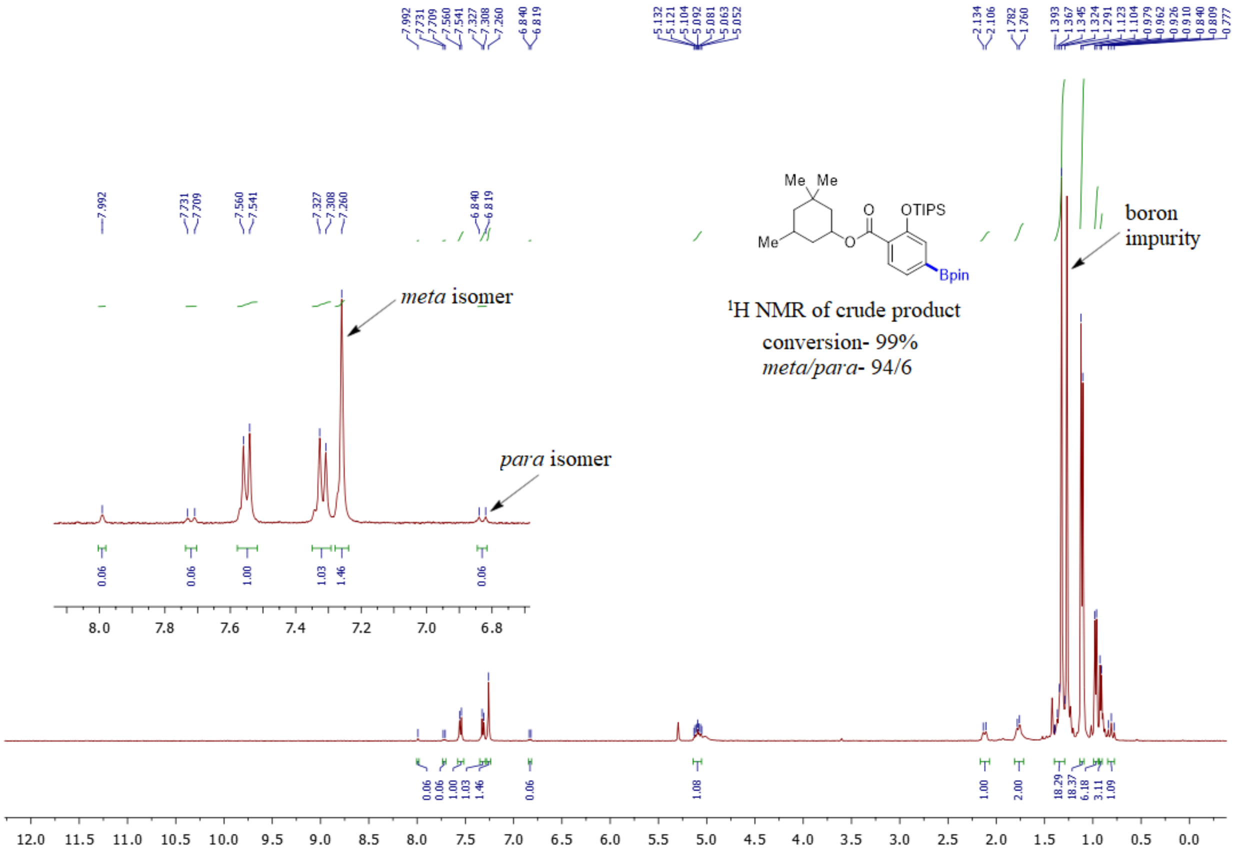

Supplementary Fig. 21: ^{1}H-NMR spectra of crude **11h** with **L1** (25 °C, 400 MHz, $CDCl_3$)

K. Procedure of insitu triisopropylsilyl group deprotection:

Meta-borylation and deprotection of triisopropyl(phenoxy)silane **(1l)***:*

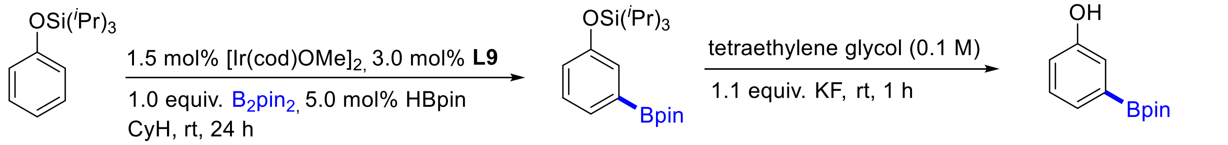

1l: 0.5 mmol

2l: meta/para = 97/3
GC-MS conv. = 100%

12: 87% (isolated yield)

In an argon-filled glove box, a 5.0 mL Wheaton microreactor was charged with $[Ir(cod)OMe]_2$ (4.97 mg, 1.5 mol%), B_2pin_2 (127.0 mg, 1.0 equiv.), ligand **L9** (3.4 mg, 3.0 mol%), HBpin (3.2 mg, 5.0 mol%) and dry cyclohexane (2.0 mL). The reaction mixture was stirred for 2 minutes at room temperature and then triisopropyl(phenoxy)silane (0.5 mmol, 125 mg) was added. The microreactor was capped with a teflon pressure cap and stirred for 24 h at room temperature. After completion (judged by GC-MS), CyH was removed under reduced pressure and the crude borylated product was used for the next step.

The crude borylated product was dissolved in tetraethylene glycol (5 ml, 0.1 M), KF (32 mg, 0.55 mmol, 1.1 equiv.) was added and stirred for 1 h at room temperature. After completion (judged by GC-MS), the reaction mixture was diluted with water (15 mL) and extracted with ethyl acetate (10 mL x 3). The combined organic layer washed with brine solution (20 mL), dried over anhydrous Na_2SO_4, filtered and concentrated under reduced pressure. The resulting mixture was purified by silica gel chromatography (10% ethyl acetate in hexane as eluent) gave 95.7 mg (87%) of 3-(4,4,5,5-tetramethyl-1,3,2-dioxaborolan-2-yl)phenol **(12)** as a colourless liquid.

^{1}H NMR (400 MHz, $CDCl_3$): δ 7.37 (d, J = 7.2 Hz, 1H), 7.29 (d, J = 2.4 Hz, 1H), 7.25 (t, J = 7.6 Hz, 1H), 6.97 (dd, J = 8.0, 2.4 Hz, 1H), 6.14 (brs, 1H), 1.34 (s, 12H).

^{13}C NMR (100 MHz, $CDCl_3$): δ 155.4, 129.4, 127.0, 121.3, 118.7, 24.9.

^{11}B NMR (128 MHz, $CDCl_3$): δ 30.6.

HRMS (ESI) *m/z* calcd for $C_{12}H_{17}BO_3$ $[M+H]^+$ 221.1349, found 221.1352

Meta-borylation and deprotection of triisopropyl(2-(trifluoromethyl)phenoxy)silane **(4g)***:*

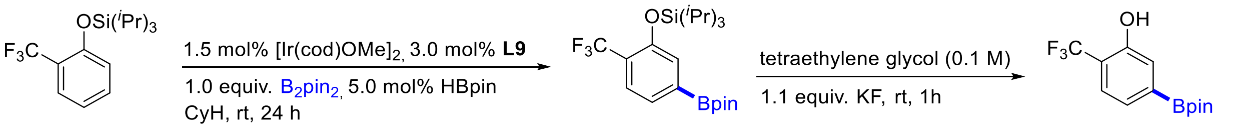

4g: 0.5 mmol

5g: meta/para = 99/1
GC-MS Conv. = 100%

13: 85% (isolated yield)

In an argon-filled glove box, a 5.0 mL Wheaton microreactor was charged with $[Ir(cod)OMe]_2$ (4.97 mg, 1.5 mol%), B_2pin_2 (127.0 mg, 1.0 equiv.), ligand **L9** (3.4 mg, 3.0 mol%), HBpin (3.2 mg, 5.0 mol%) and dry cyclohexane (2.0 mL). The reaction mixture was stirred for 2 minutes at room temperature and then triisopropyl(2-(trifluoromethyl)phenoxy)silane (0.5 mmol, 159 mg) was added. The microreactor was capped with a teflon pressure cap and and stirred for 24 h at

room temperature. After completion (judged by GC-MS), CyH was removed under reduced pressure and the crude borylated product was used for the next step.

The crude borylated product was dissolved in tetraethylene glycol (5 ml, 0.1 M), KF (32 mg, 0.55 mmol, 1.1 equiv.) was added and stirred for 1 h at room temperature. After completion (judged by GC-MS), the reaction mixture was diluted with water (15 mL) and extracted with ethyl acetate (10 mL x 3). The combined organic layer washed with brine solution (20 mL), dried over anhydrous Na_2SO_4, filtered and concentrated under reduced pressure. The resulting mixture was purified by silica gel chromatography (10% ethyl acetate in hexane as eluent) gave 122 mg (85%) of 5-(4,4,5,5-tetramethyl-1,3,2-dioxaborolan-2-yl)-2-(trifluoromethyl)phenol **(13)** as a colourless gummy liquid.

^{1}H NMR (400 MHz, $CDCl_3$): δ 7.51 (d, J = 7.6 Hz, 1H), 7.40 (d, J = 7.6 Hz, 1H), 7.35 (s, 1H), 6.05 (brs, 1H), 1.34 (s, 12H).

^{13}C NMR (100 MHz, $CDCl_3$): δ 153.1, 126.7, 126.3 (q, J = 4.8 Hz), 124.1 (q, J = 270.8 Hz), 123.5, 119.0 (q, J = 30.3 Hz), 84.6, 25.0.

^{11}B NMR (128 MHz, $CDCl_3$): δ 30.6.

HRMS (ESI) *m/z* calcd for $C_{13}H_{16}BF_3O_3$ $[M+H]^+$ 289.1223, found 289.1223

Meta-borylation and deprotection of triisopropyl(4-(trifluoromethoxy)phenoxy)silane **(6h)**:

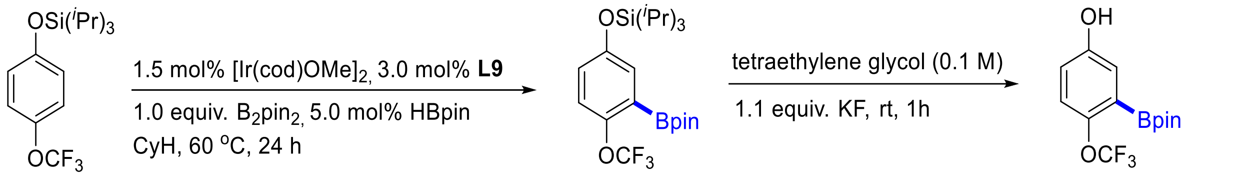

6h: 0.5 mmol | **7h:** meta = 100, GC-MS Conv. = 99% | **14:** 95% (isolated yield)

In an argon-filled glove box, a 5.0 mL Wheaton microreactor was charged with $[Ir(cod)OMe]_2$ (4.97 mg, 1.5 mol%), B_2pin_2 (127.0 mg, 1.0 equiv.), ligand **L9** (3.4 mg, 3.0 mol%), HBpin (3.2 mg, 5.0 mol%) and dry cyclohexane (2.0 mL). The reaction mixture was stirred for 2 minutes at room temperature and then triisopropyl(4-(trifluoromethoxy)phenoxy)silane (0.5 mmol, 167 mg) was added. The microreactor was capped with a teflon pressure cap and placed into pre-heated aluminum block at 40 °C and stirred for 24 h. After completion (judged by GC-MS), CyH was removed under reduced pressure and the crude borylated product was used for the next step.

The crude borylated product was dissolved in tetraethylene glycol (5 ml, 0.1 M), KF (32 mg, 0.55 mmol, 1.1 equiv.) was added and stirred for 1 h at room temperature. After completion (judged by GC-MS), the reaction mixture was diluted with water (15 mL) and extracted with ethyl acetate (10 mL x 3). The combined organic layer washed with brine solution (20 mL), dried over anhydrous Na_2SO_4, filtered and concentrated under reduced pressure. The resulting mixture was purified by silica gel chromatography (10% ethyl acetate in hexane as eluent) gave 144 mg (95%) of 3-(4,4,5,5-tetramethyl-1,3,2-dioxaborolan-2-yl)-4-(trifluoromethoxy)phenol **(14)** as a colourless liquid.

^{1}H NMR (400 MHz, $CDCl_3$): δ 7.18 (d, *J* = 3.2 Hz, 1H), 7.11 (d, *J* = 8.8 Hz, 1H), 6.91 (dd, *J* = 8.8, 3.2 Hz, 1H), 5.13 (brs, 1H), 1.34 (s, 12H).
^{13}C NMR (100 MHz, $CDCl_3$): δ 153.9, 146.9, 123.4, 122.4, 120.6 (d, *J* = 253.9 Hz), 119.1, 84.37, 24.84.
^{11}B NMR (128 MHz, $CDCl_3$): δ 30.4.
HRMS (ESI) *m/z* calcd for $C_{13}H_{16}BF_3O_4$ $[M+H]^+$ 305.1172, found 305.1172.

L. Computational Details:

All calculations were performed with Gaussian 09 program.[47] Geometry optimizations were performed using B3LYP functionals.[48,49] LANL2DZ pseudo-potential[50] was set for Ir and 6-31G(d) basis set was set for all other atoms. After optimization, frequency calculations were subsequently performed to confirm that all stationary points had correct number of imaginary frequencies (zero for minima and one for transition states) and to provide thermodynamic corrections at 298 K, 1 atm. For all transition states, Intrinsic Reaction Coordinate (IRC)[51] were calculated to confirm that they indeed connected between correct minima. Single-point energies of optimized structures were calculated using M06 functionals[52] with SDD[53] for Ir and 6-311+G(d,p) for all the other atoms. Methods as B3LYP, B3LYP-D3(BJ)[54,55] and ωB97X-D[56] were also used for single-point energy calculations to identify the property of the noncovalent interactions (see Fig. S2). SMD solvation model[57] (solvent = cyclohexane) was used in single-point energy calculations to consider solvent effect. The final Gibbs free energy was calculated as the sum of Gibbs free energy correction (from frequency calculation) and the single point energy in solution. For each species, extensive conformational search was done and the reported value is

from the conformer with the lowest Gibbs free energy. The graphics of the computed species were rendered using CYL view.[58]

i) Consideration of other possible reaction pathways

We also considered the possibilities of other possible reaction pathways, as shown as **Fig. S22.** Firstly, the substrate may directly undergo C-H oxidative addition to **INT1** via **TS12**, but it requires a high activation free energy of 37.2 kcal/mol and thus fails to compete with the B-B addition via **TS1**. **INT2** may partly dissociate its cod, forming a mildly endergonic intermediate **INT9** (+7.2 kcal/mol). However, C-H activation catalyzed by **INT9** is strongly unfavorable (**TS13**, +50.0 kcal/mol). Worthy of note is that the cod may also fully dissociate then undergoes C-H activation via **TS14**. As shown, a Si-O secondary interaction was involved, which was once suspected as the origin of the *meta*-selectivity in the early stage of research. However, this pathway from **INT2** to **TS14** still requires a barrier of 36.7 kcal/mol, not reasonable for a reaction occurring at room temperature. Also, experiments showed higher regioselectivity was obtained for substrates with bulkier trialkyl silyl groups. Neither Si-O electrostatic interaction nor orbital-orbital interaction, which should be stronger for less bulky ones, can explain. An isomeric **TS15** was also located, in which Ir-B cleavage accompanies C-H activation concertedly. This process has a lower barrier, but is still over 5 kcal/mol higher than the irreversible B-B oxidative addition via **TS3**. Therefore, we believe our proposed pathway in main text is so far most plausible.

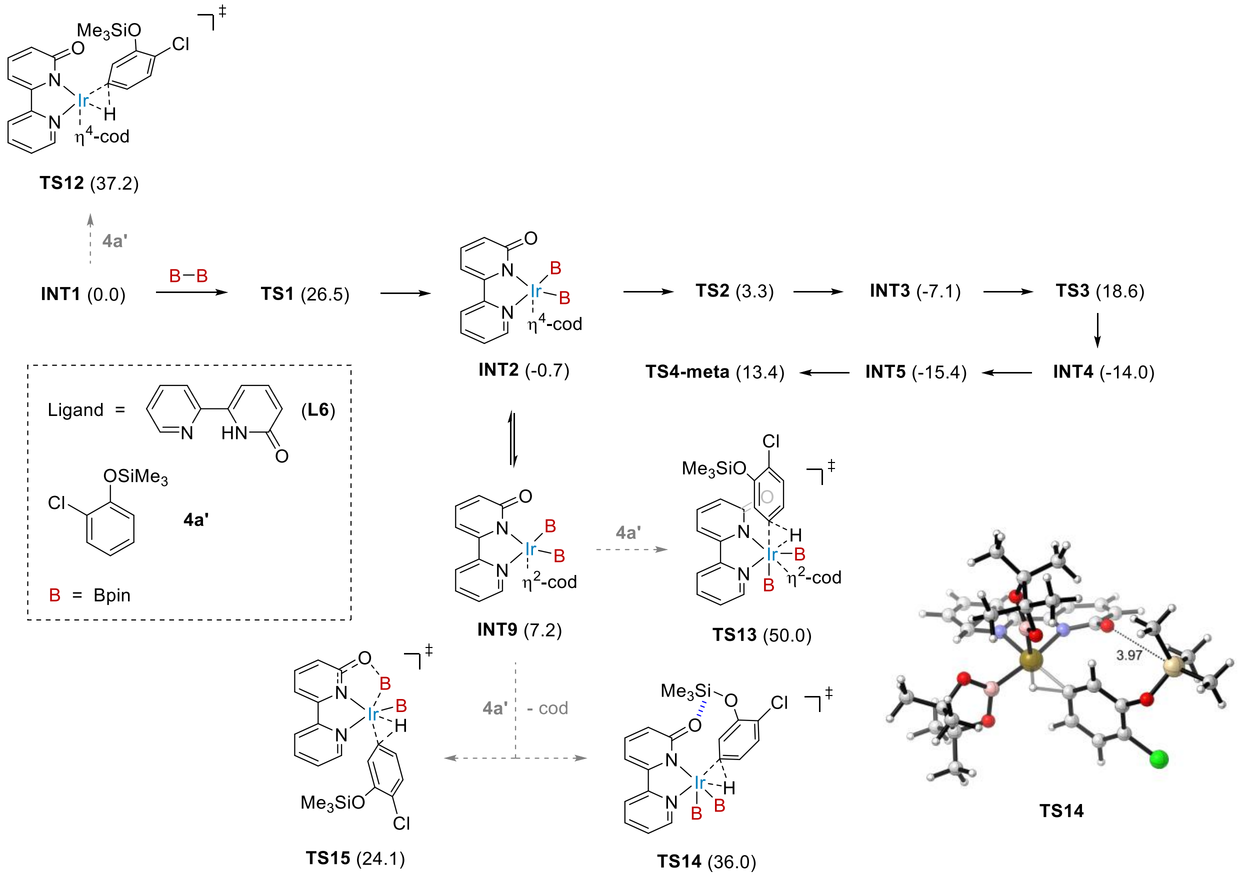

Supplementary Fig. 22: Consideration of other possible reaction pathways.

Computed at SMD(CyH)-M06/6-311+G(d,p)//B3LYP/6-31G(d). Relative free energies are given in kcal/mol. The proposed reaction pathway in main text is posed for comparison.

ii) Identification of dispersion in determining *meta*-selectivity

To get a better understanding on the property of functioning noncovalent interaction, we conducted single-point energy calculations for the C-H activation transition states using various methods as M06, B3LYP, B3LYP-D3 and ωB97X-D, as shown as **Fig. S23**. When B3LYP (without dispersion correction) is used for single-point energy calculations, the computation contradicts with the experiments; when other functionals with dispersion terms are used, the results agree with the experiments. Such tendency is more significant for the bulkier **4a** than **4a'**. Along with the structural analysis in main text, these results indicate the regioselectivity-determining noncovalent interactions are most likely to be dispersion.

		G(TS-para) - *G*(TS-meta)			
Ligand & substrate	Transition states	M06	B3LYP	B3LYP-D3	ωB97X-D
**L9** & **4a**	**TS8**	4.4	-2.4	4.1	4.7
dtbpy & **4a**	**TS9**	1.0	-1.7	1.4	2.2
**L6** & **4a**	**TS10**	2.9	0.3	4.7	4.2
**L9** & **4a'**	**TS11**	1.6	1.4	3.7	3.2

Supplementary Fig. 23: Identification of dispersion in determining *meta*-selectivity. Computed at SMD(CyH)-[Method]/6-311+G(d,p)//B3LYP/6-31G(d).

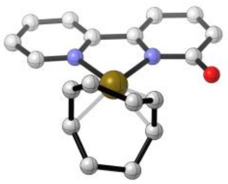

INT1

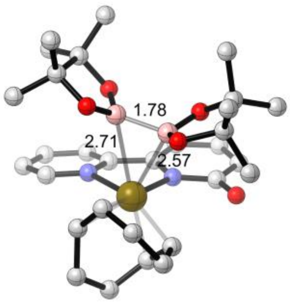

TS1

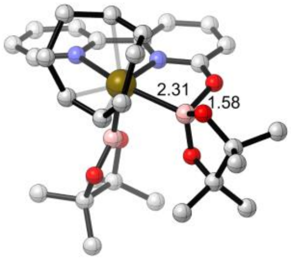

INT2

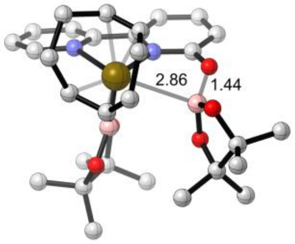

TS2

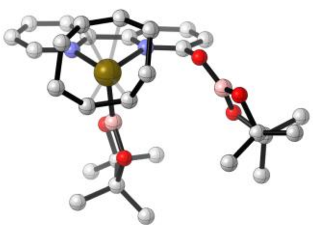

INT3

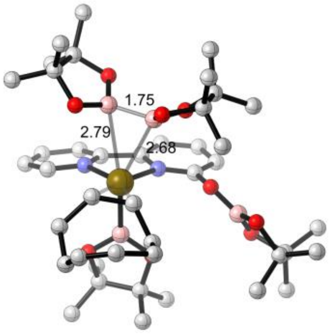

TS3

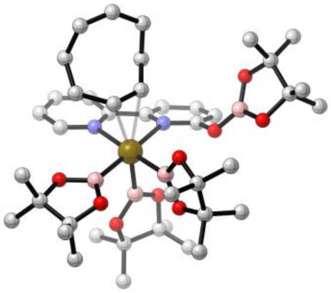

INT4

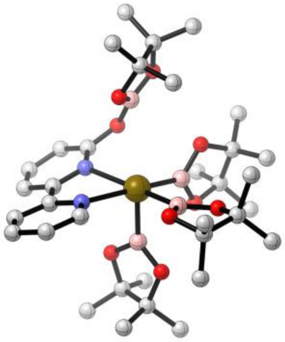

INT5

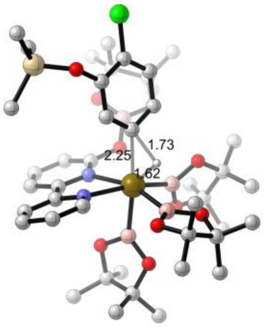

TS4-meta

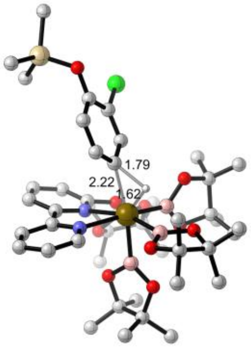

TS4-para

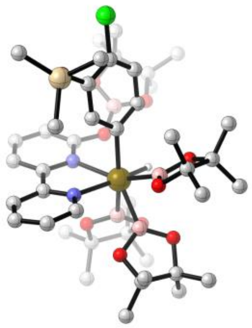

INT6

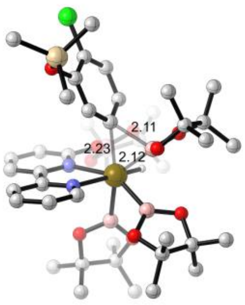

TS5

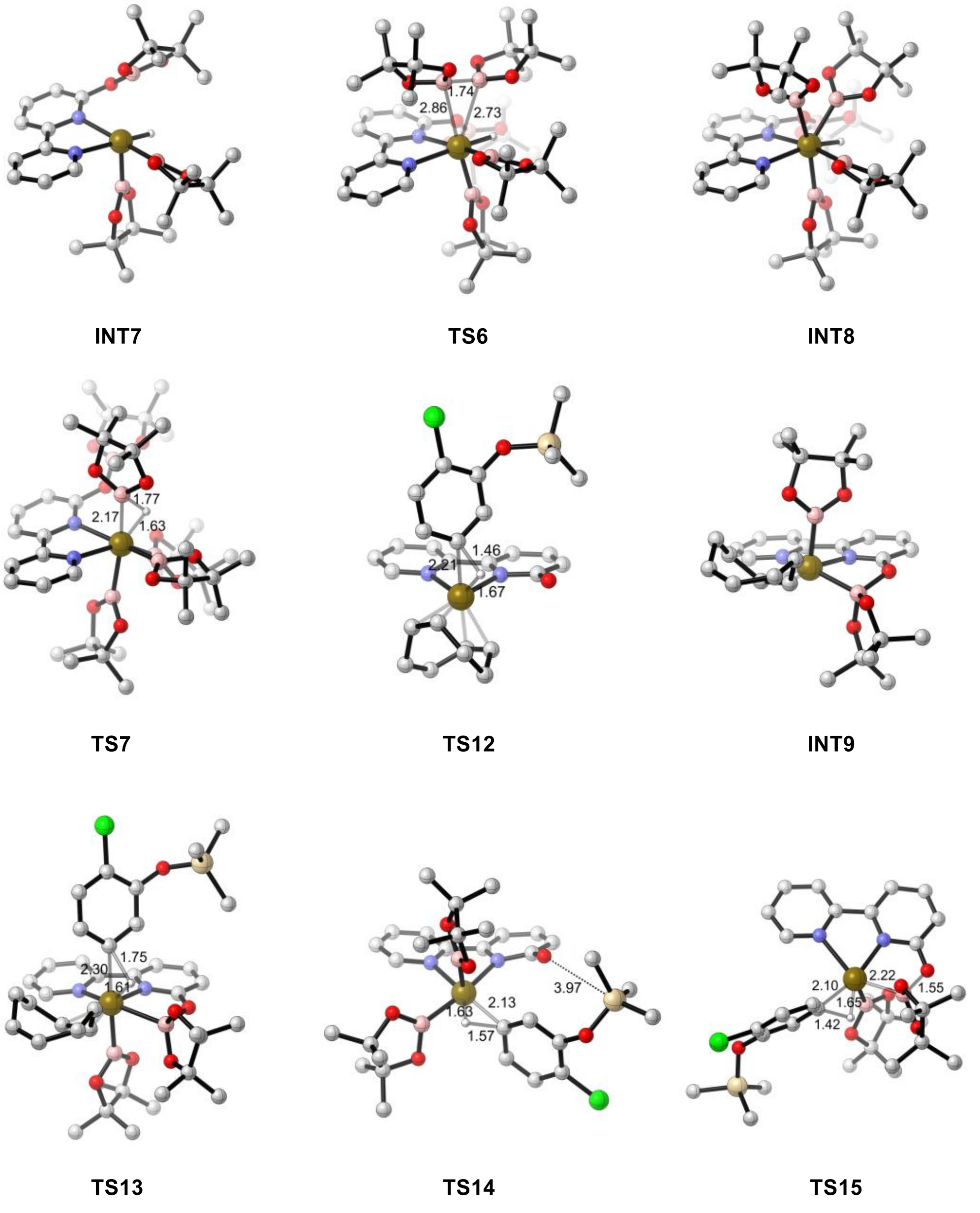

Supplementary Fig. 24: Geometries of computed species. *Atom colors:* grey, C; white, H; red, O; blue, N; pink, B; light yellow, Si; green, Cl; brown, Ir. Most hydrogen atoms are omitted for clarity. All distances are in Å.

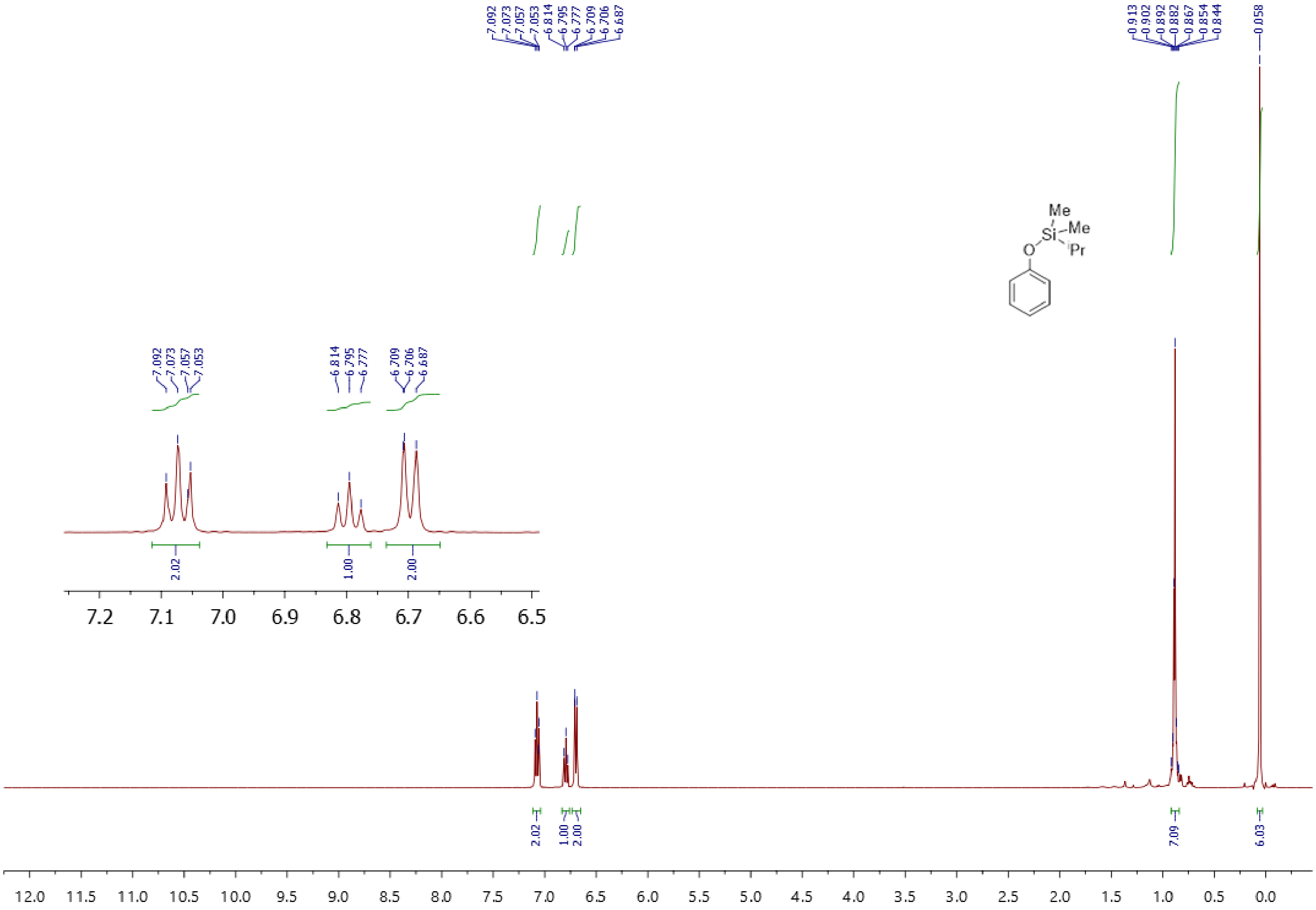

^{1}H-NMR spectra of **1o** (25 °C, 400 MHz, $CDCl_3$)

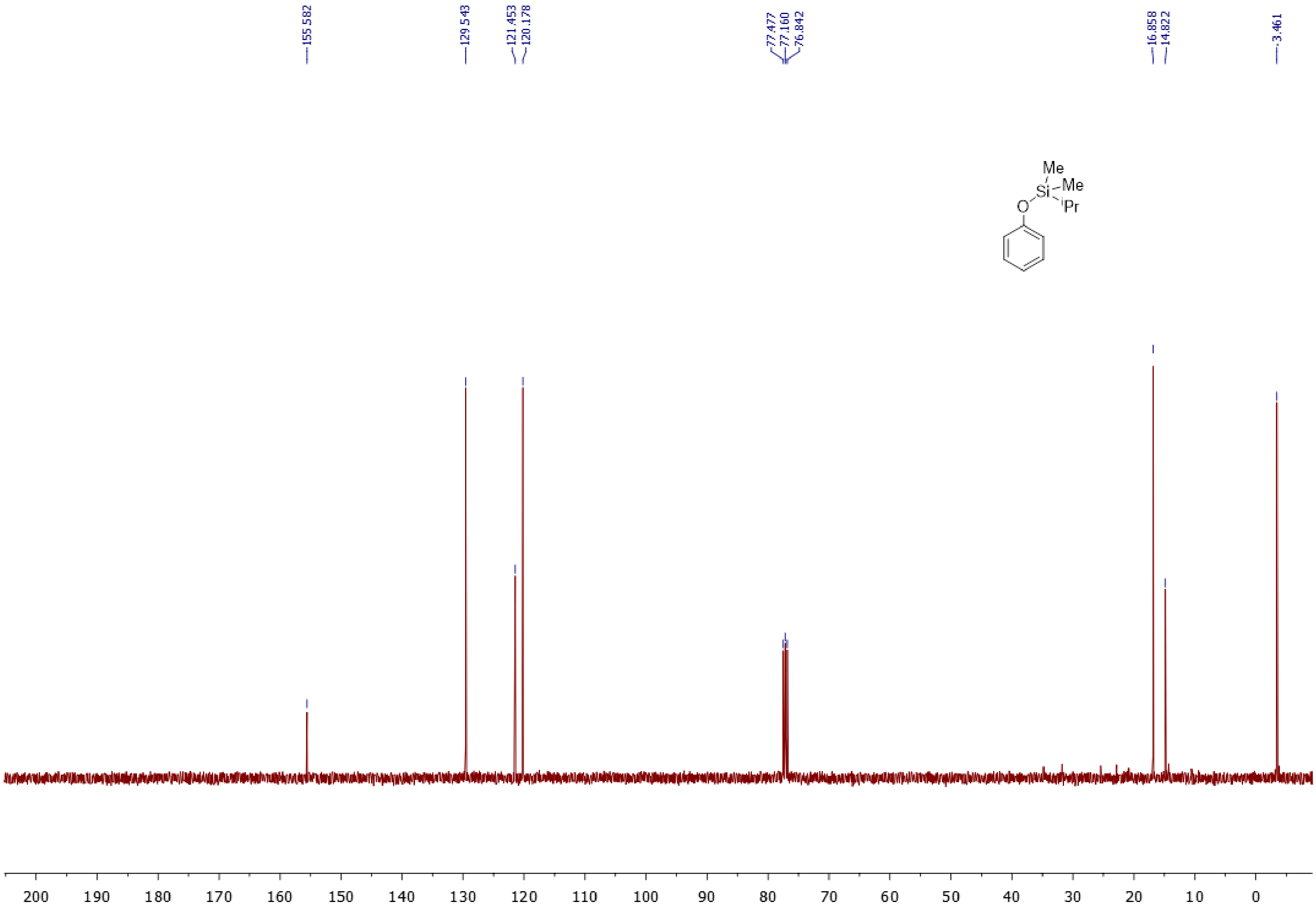

^{13}C-NMR spectra of **1o** (25 °C, 100 MHz, $CDCl_3$)

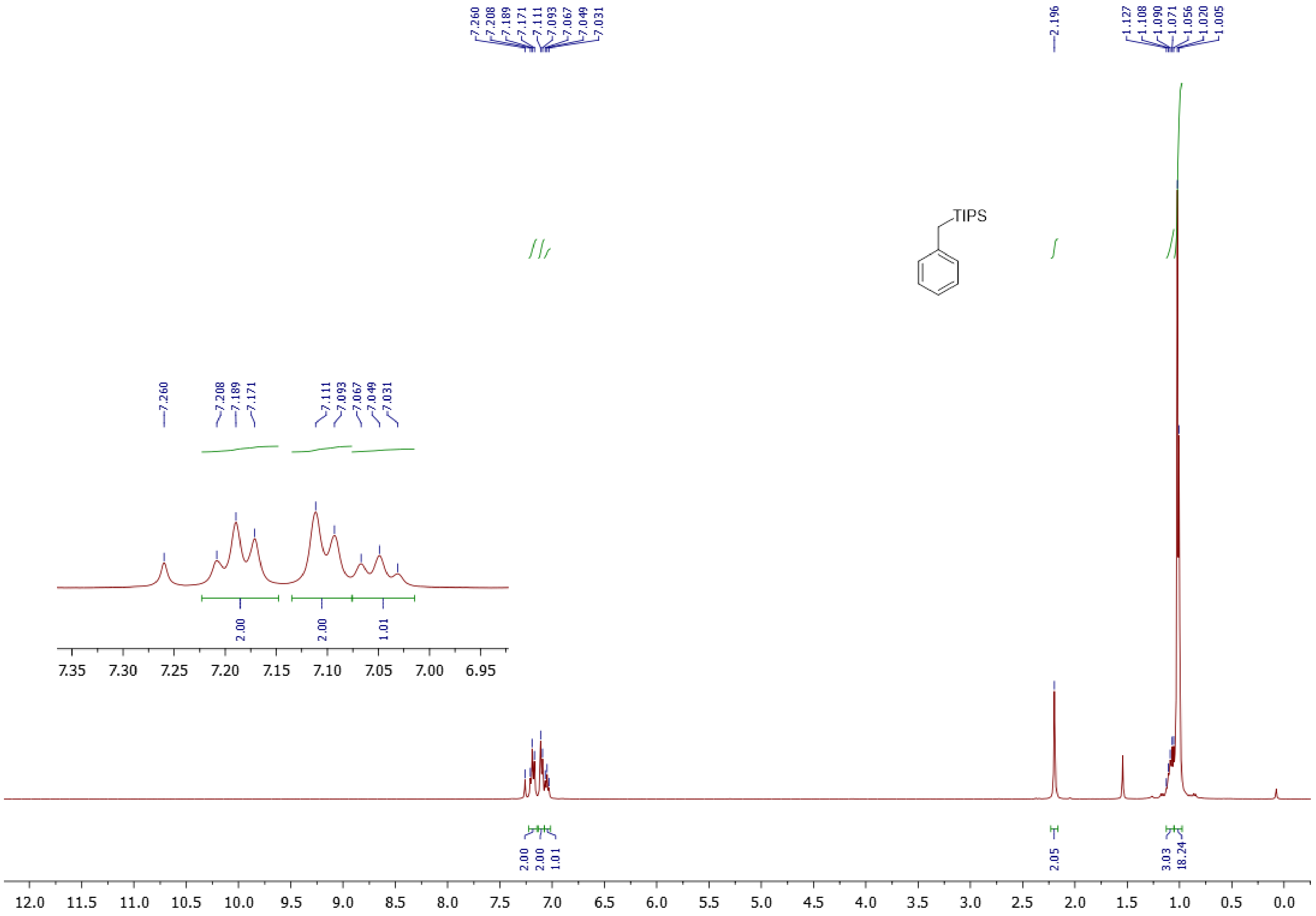

^{1}H-NMR spectra of **1q** (25 ^{o}C, 400 MHz, $CDCl_3$)

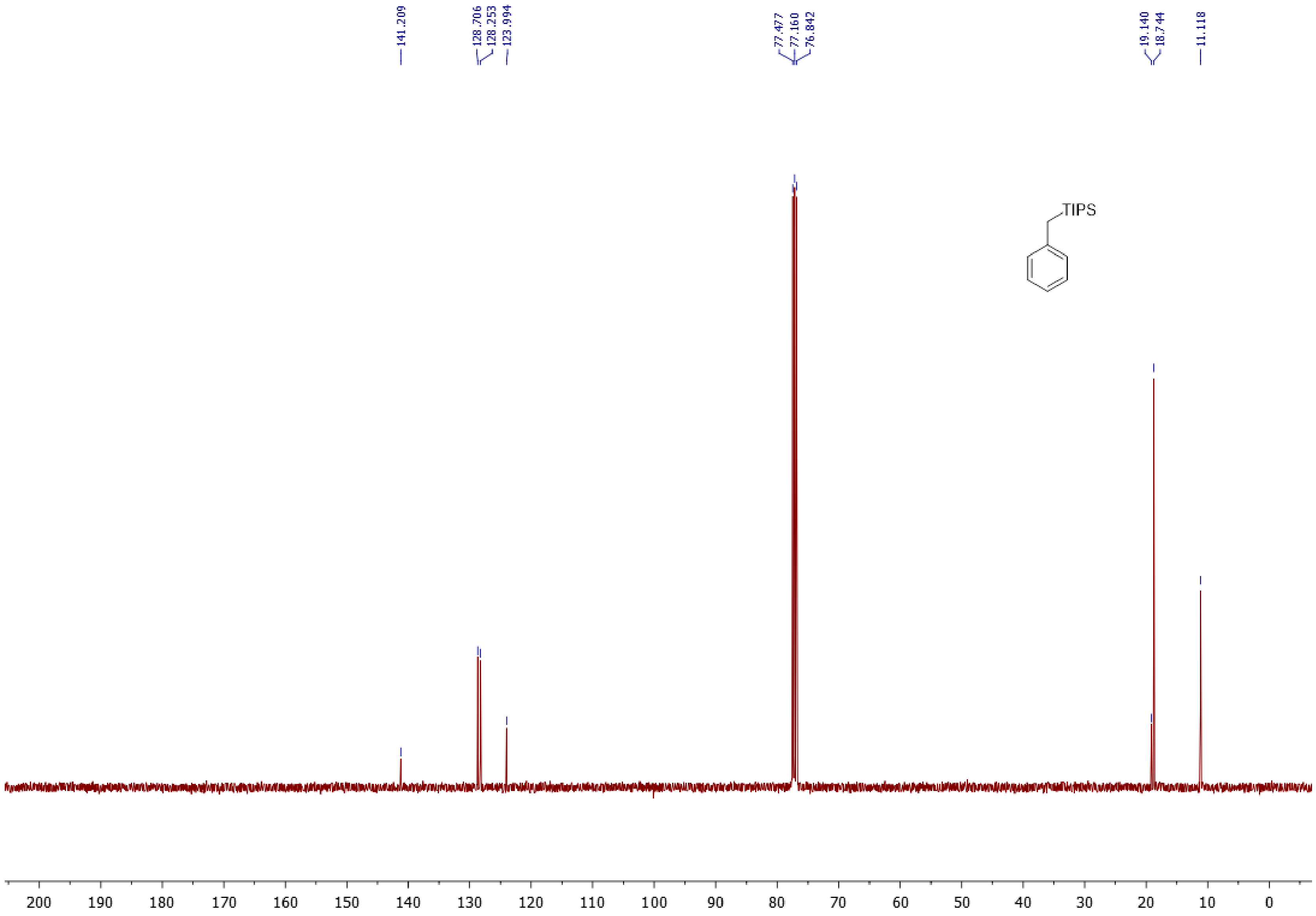

^{13}C-NMR spectra of **1q** (25 °C, 100 MHz, $CDCl_3$)

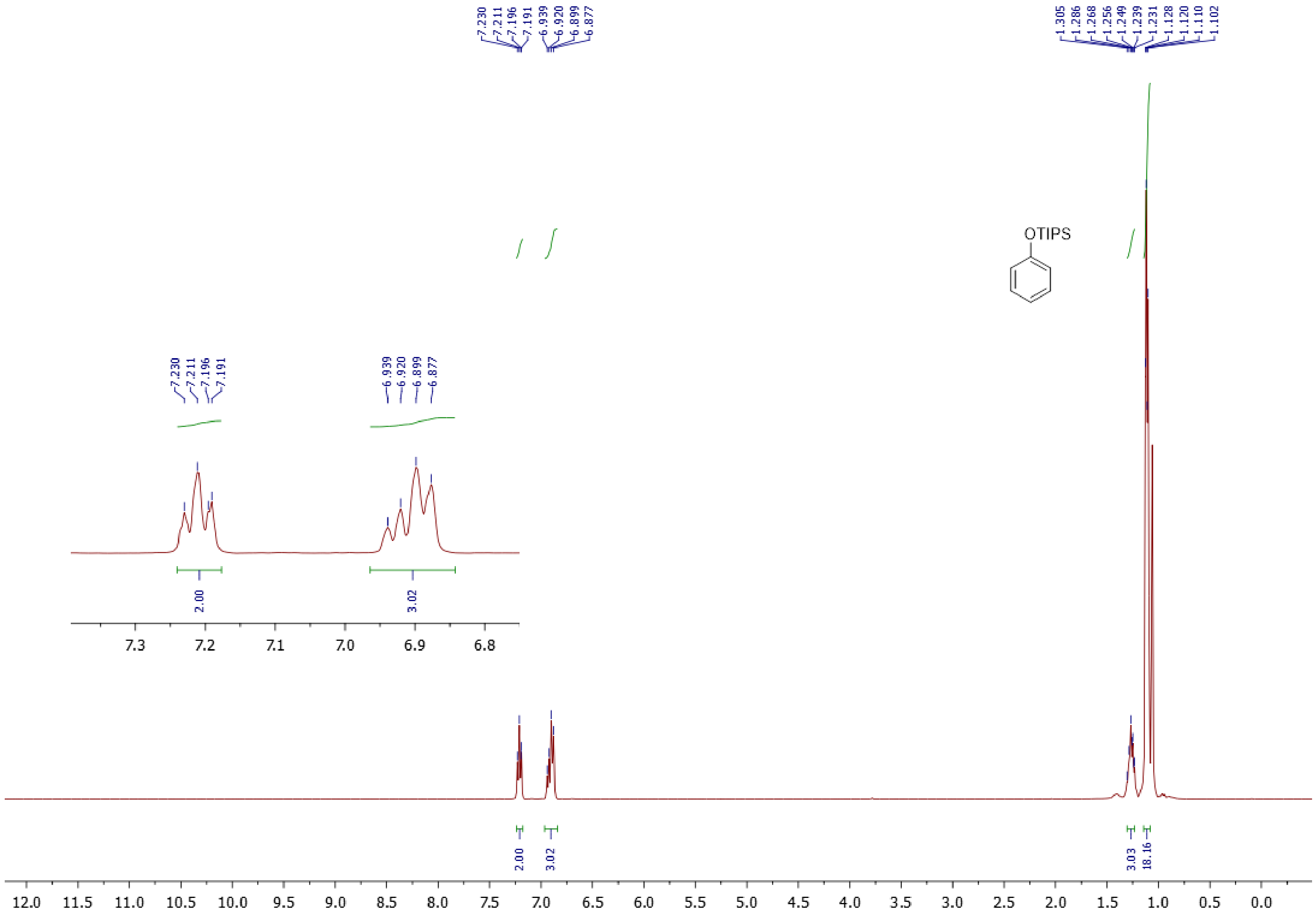

^{1}H-NMR spectra of **1l** (25 °C, 400 MHz, $CDCl_3$)

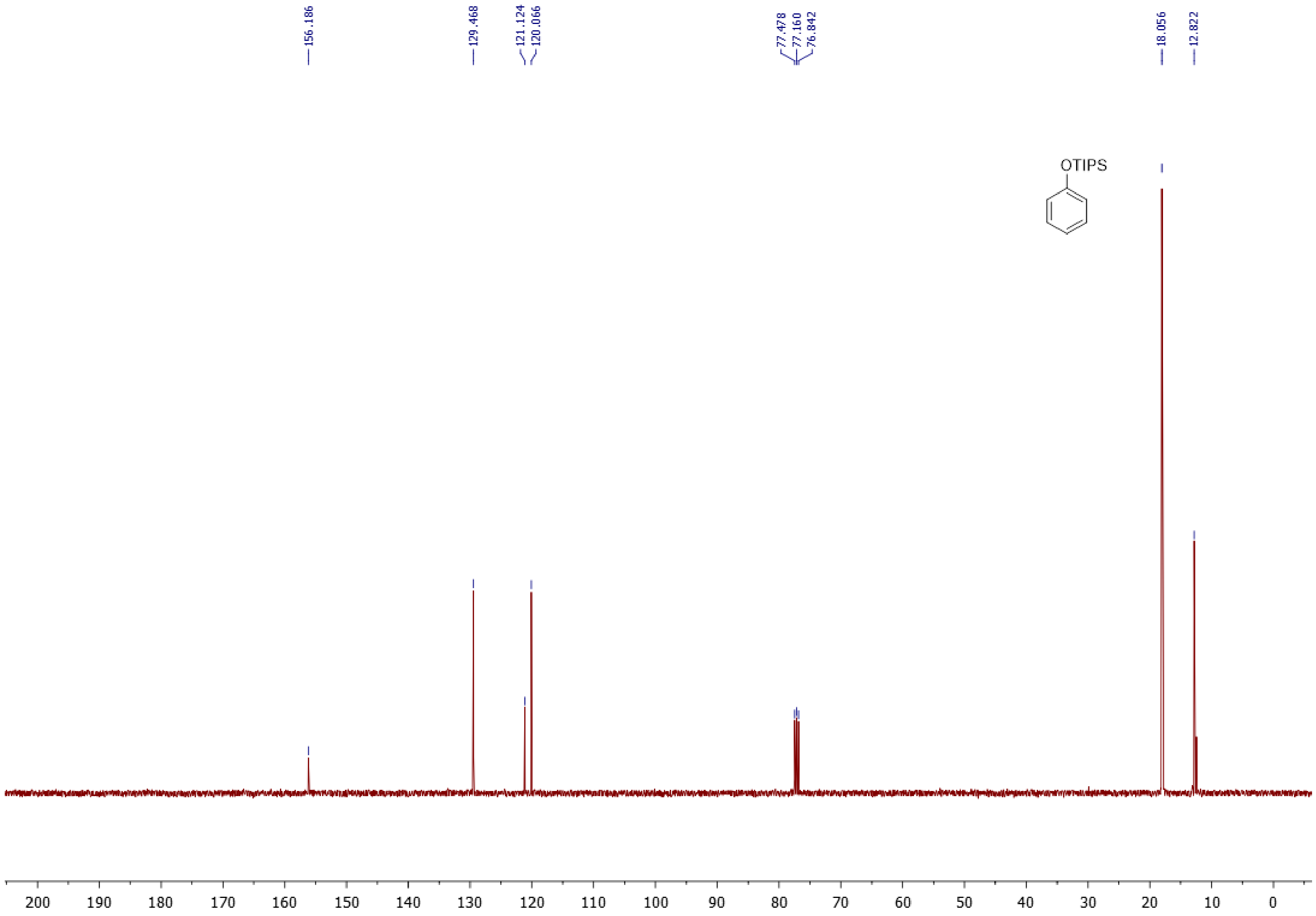

^{13}C-NMR spectra of **1l** (25 ºC, 100 MHz, $CDCl_3$)

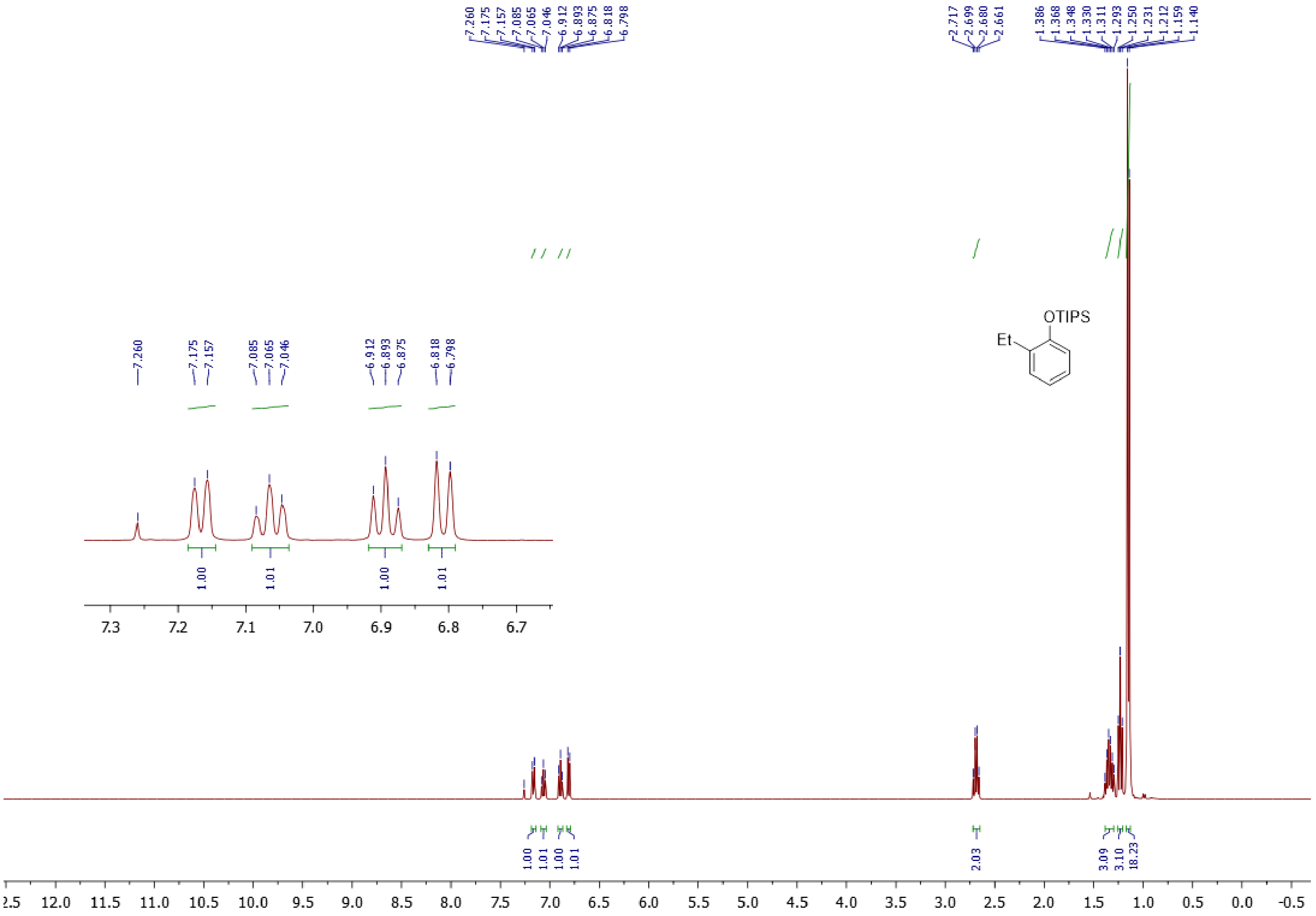

^{1}H-NMR spectra of **4e** (25 °C, 400 MHz, $CDCl_3$)

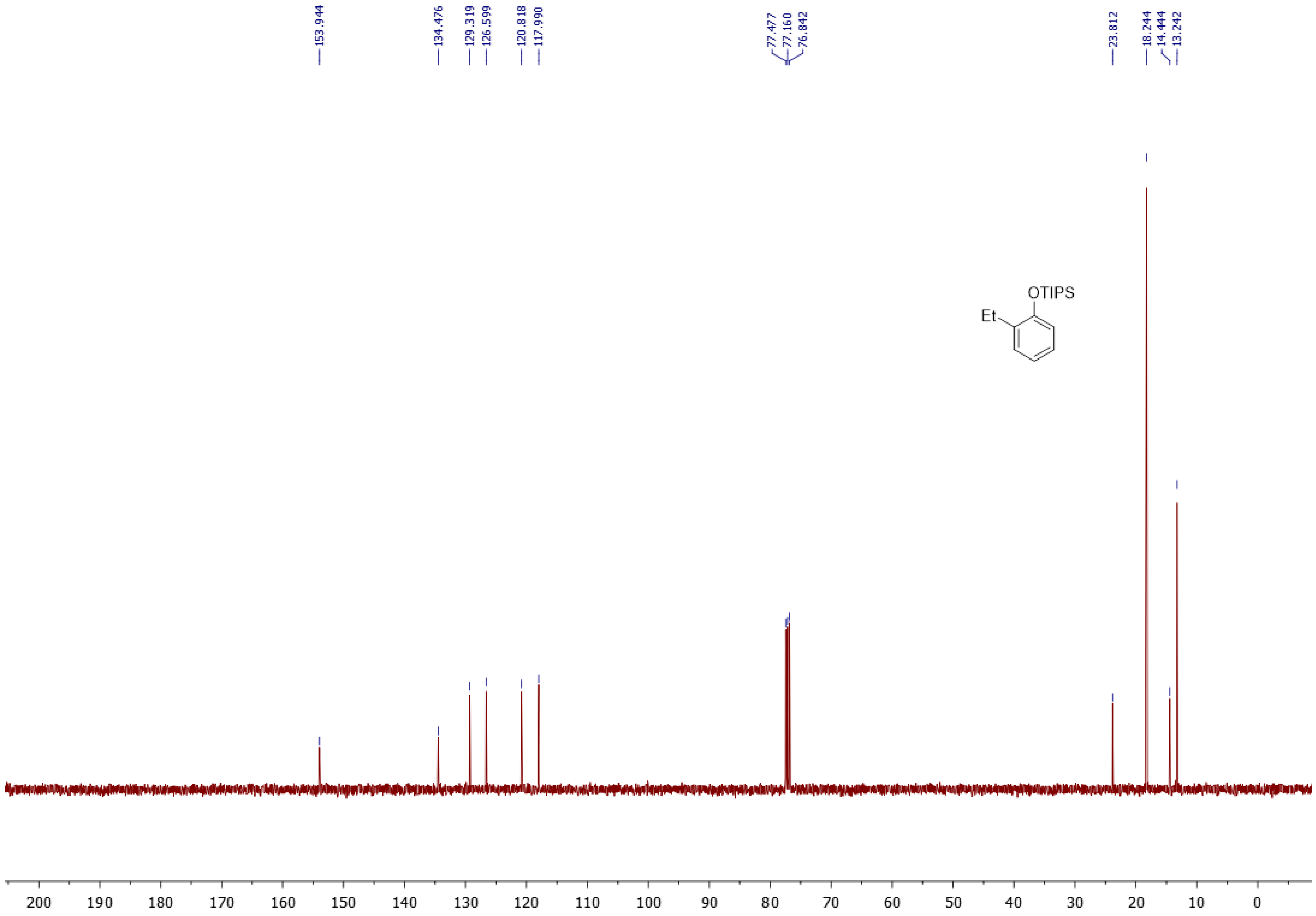

^{13}C-NMR spectra of **4e** (25 ºC, 100 MHz, $CDCl_3$)

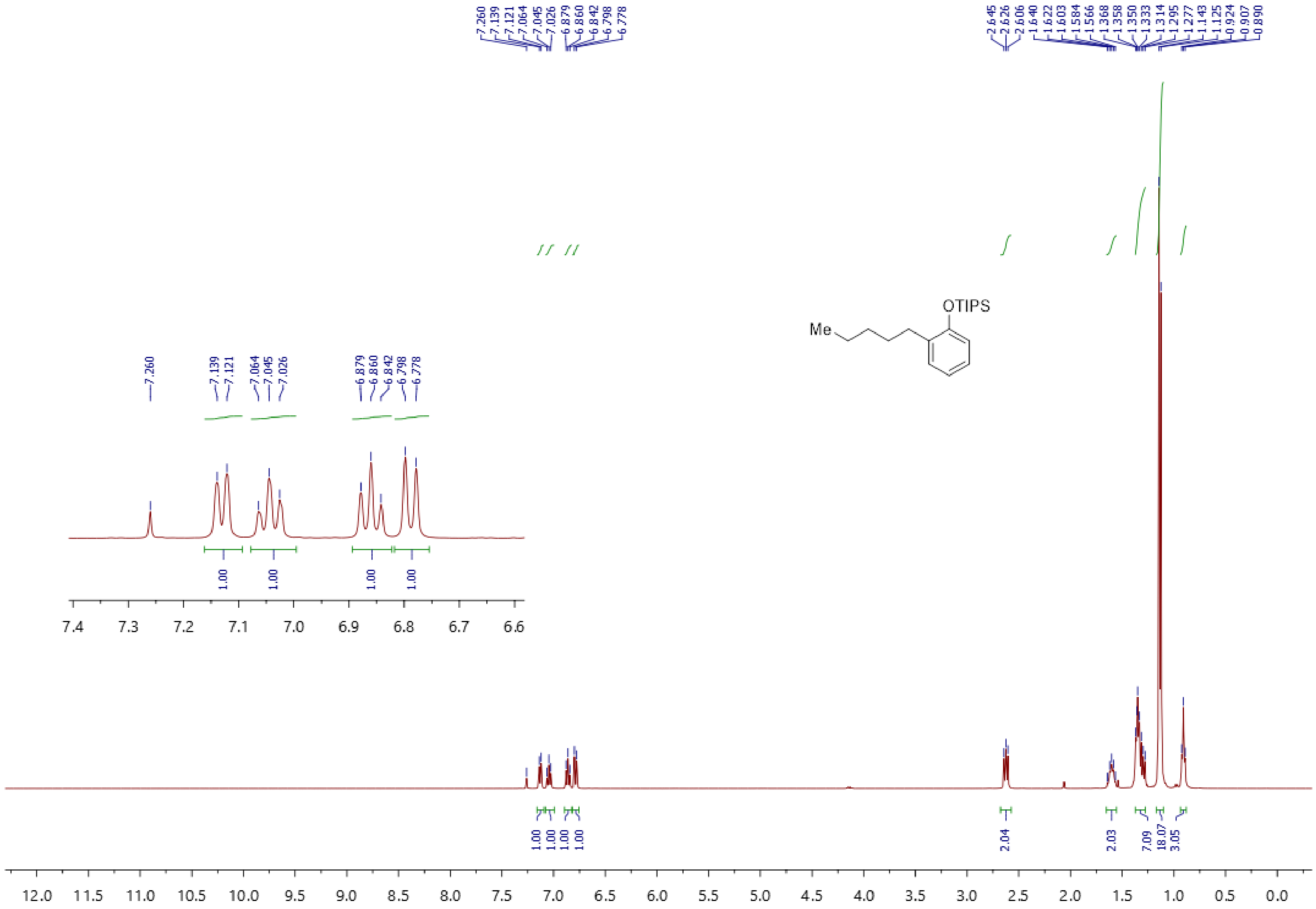

^{1}H-NMR spectra of **4f** (25 ºC, 400 MHz, $CDCl_3$)

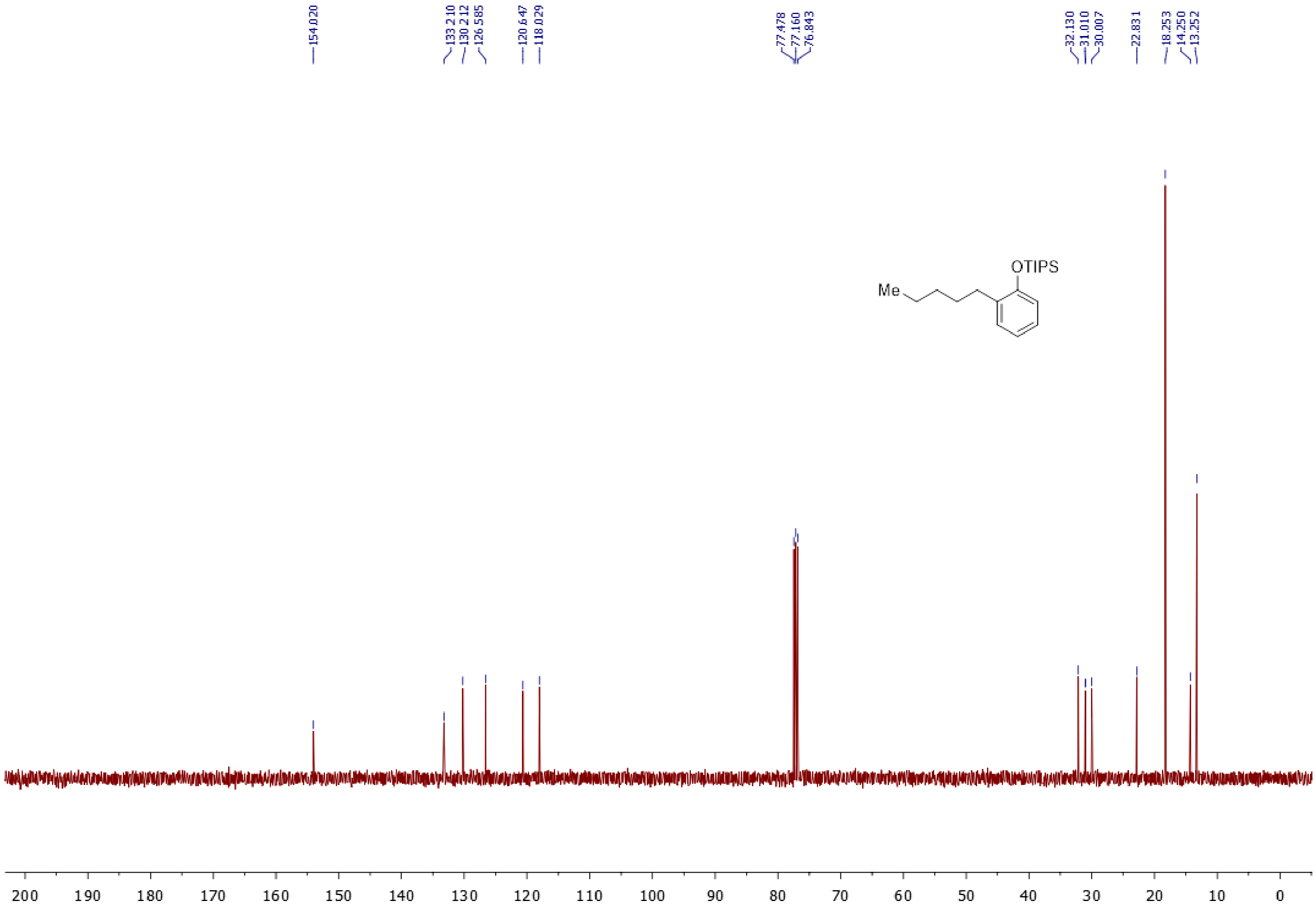

^{13}C-NMR spectra of **4f** (25 °C, 100 MHz, $CDCl_3$)

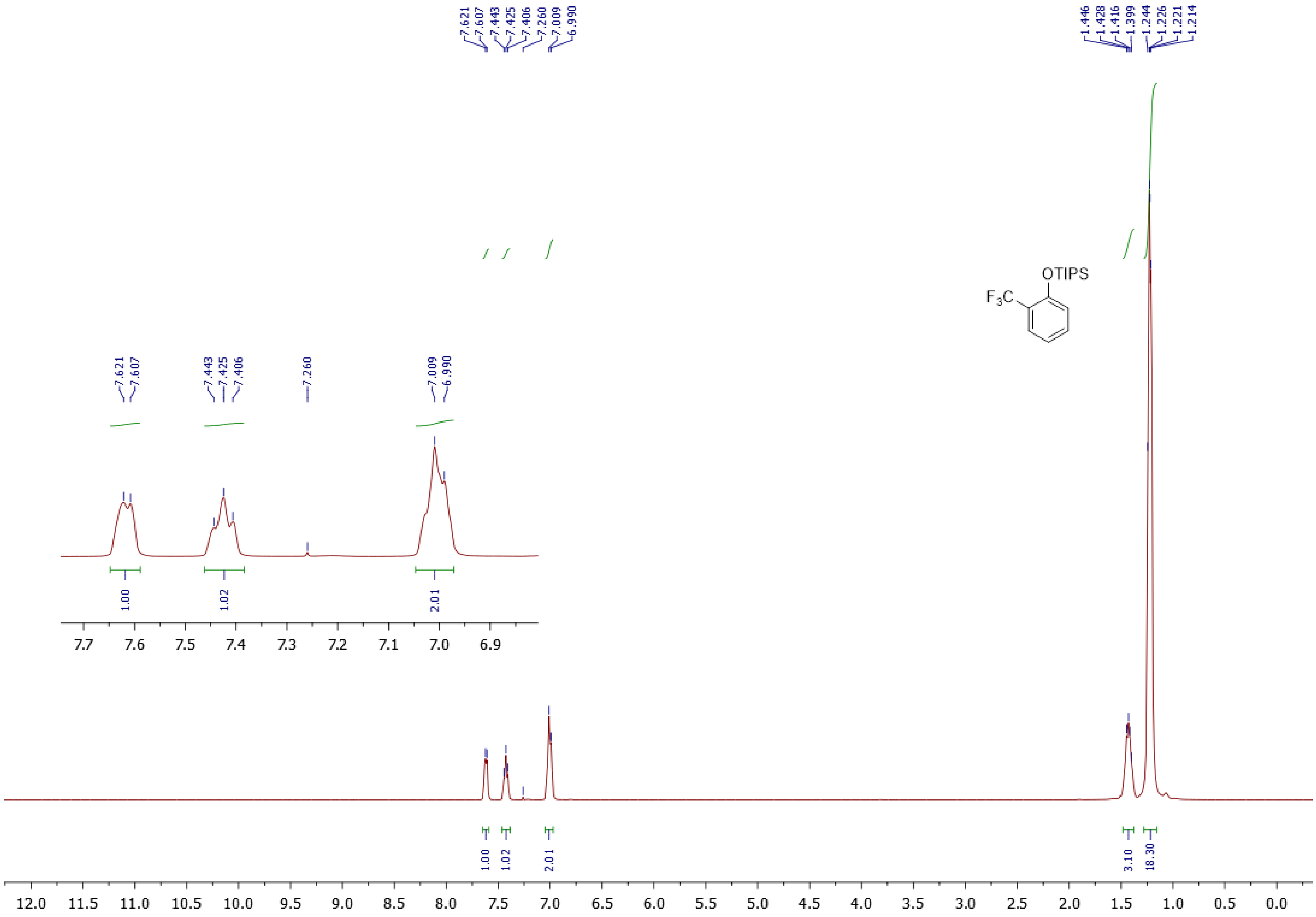

^{1}H-NMR spectra of **4g** (25 ^{o}C, 400 MHz, $CDCl_3$)

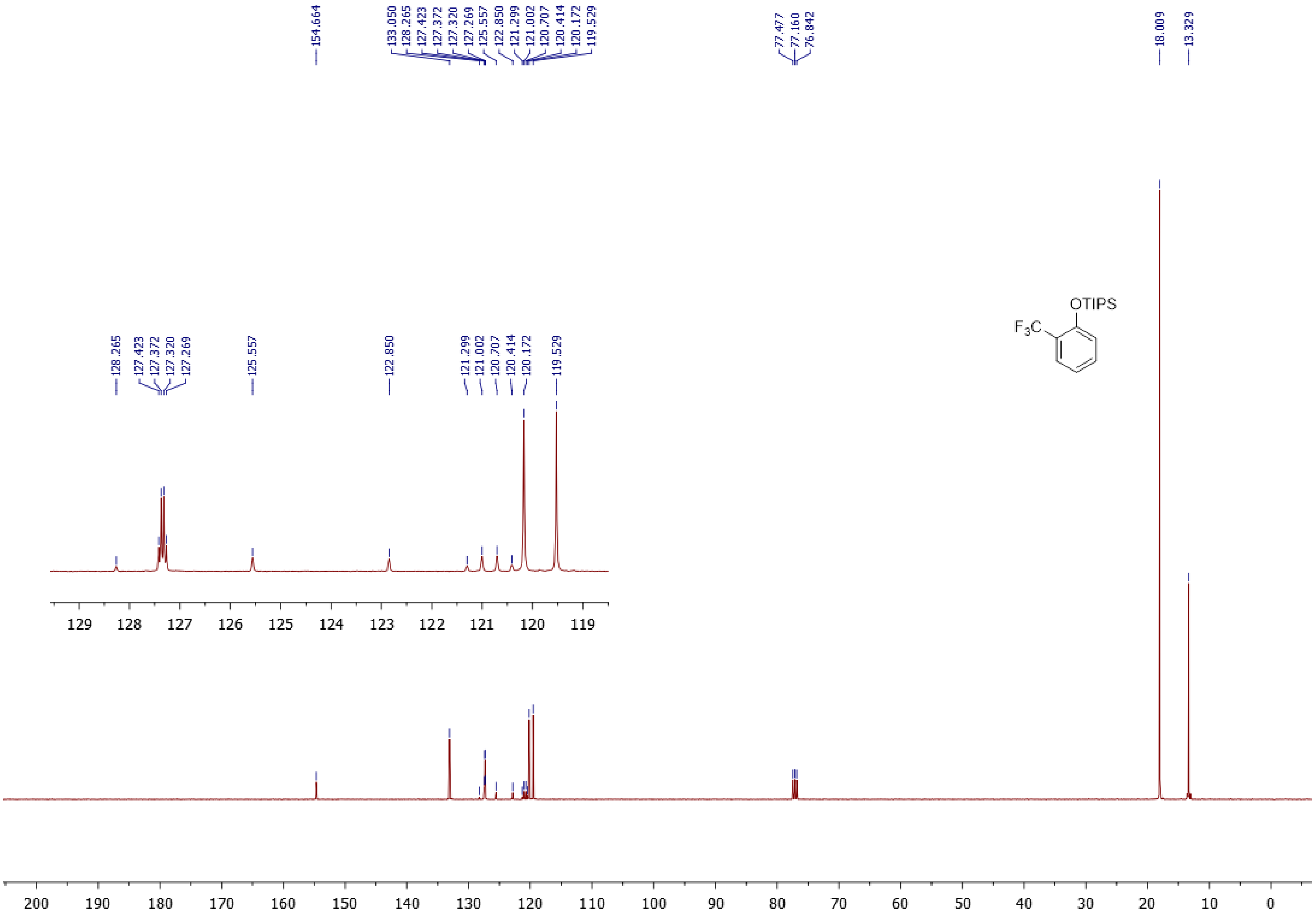

^{13}C-NMR spectra of **4g** (25 °C, 100 MHz, $CDCl_3$)

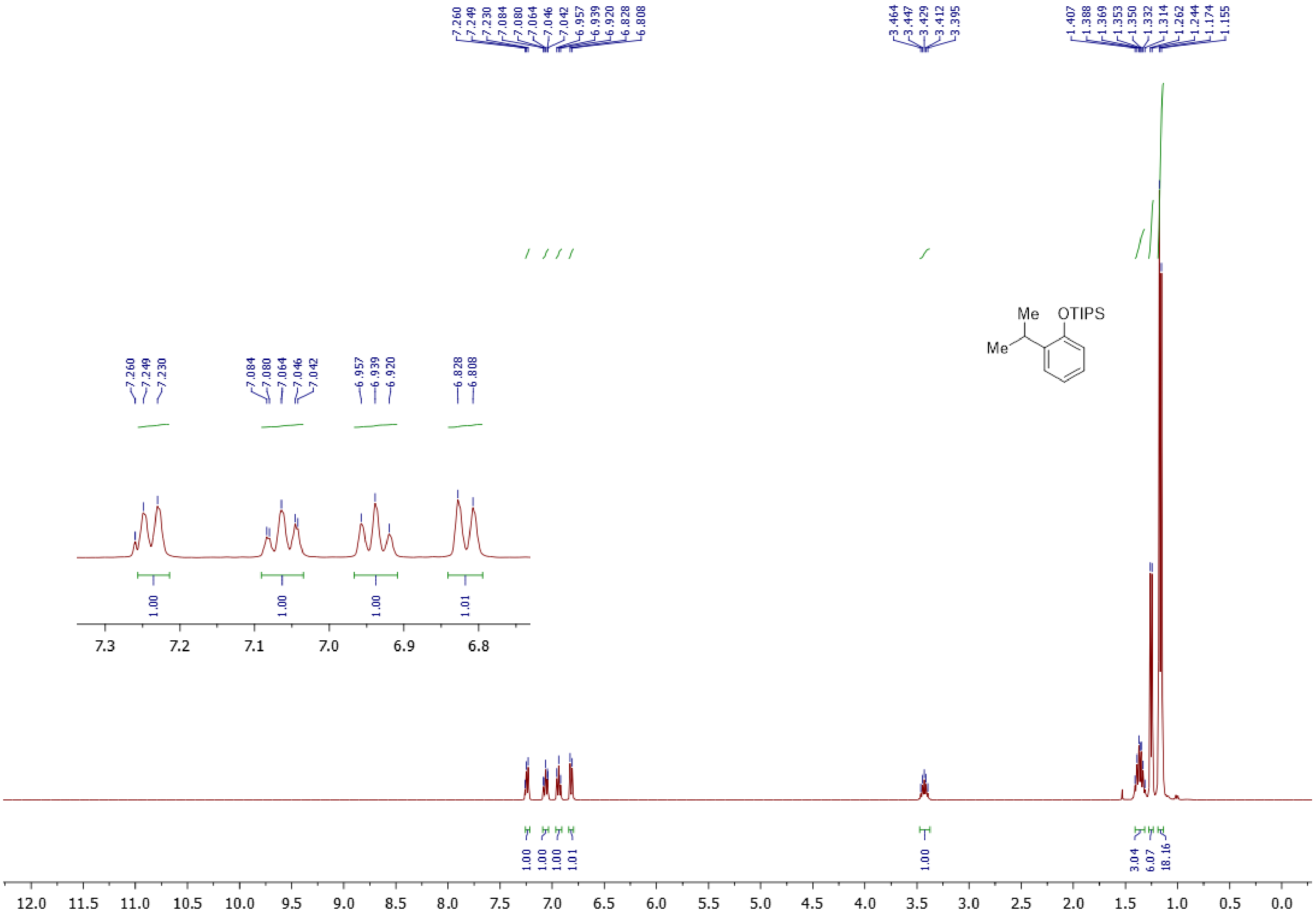

^{1}H-NMR spectra of **4h** (25 ºC, 400 MHz, $CDCl_3$)

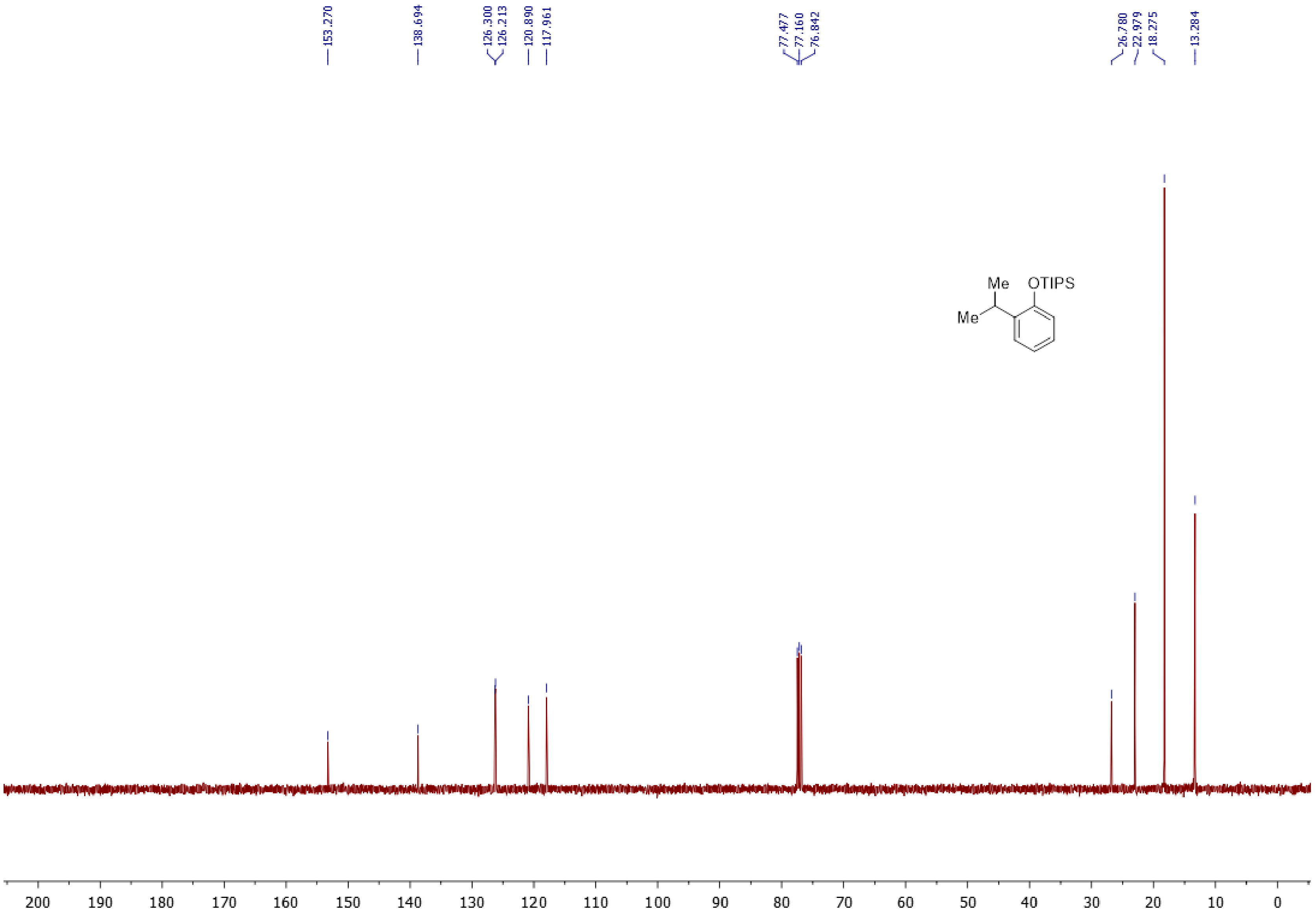

^{13}C-NMR spectra of **4h** (25 °C, 100 MHz, $CDCl_3$)

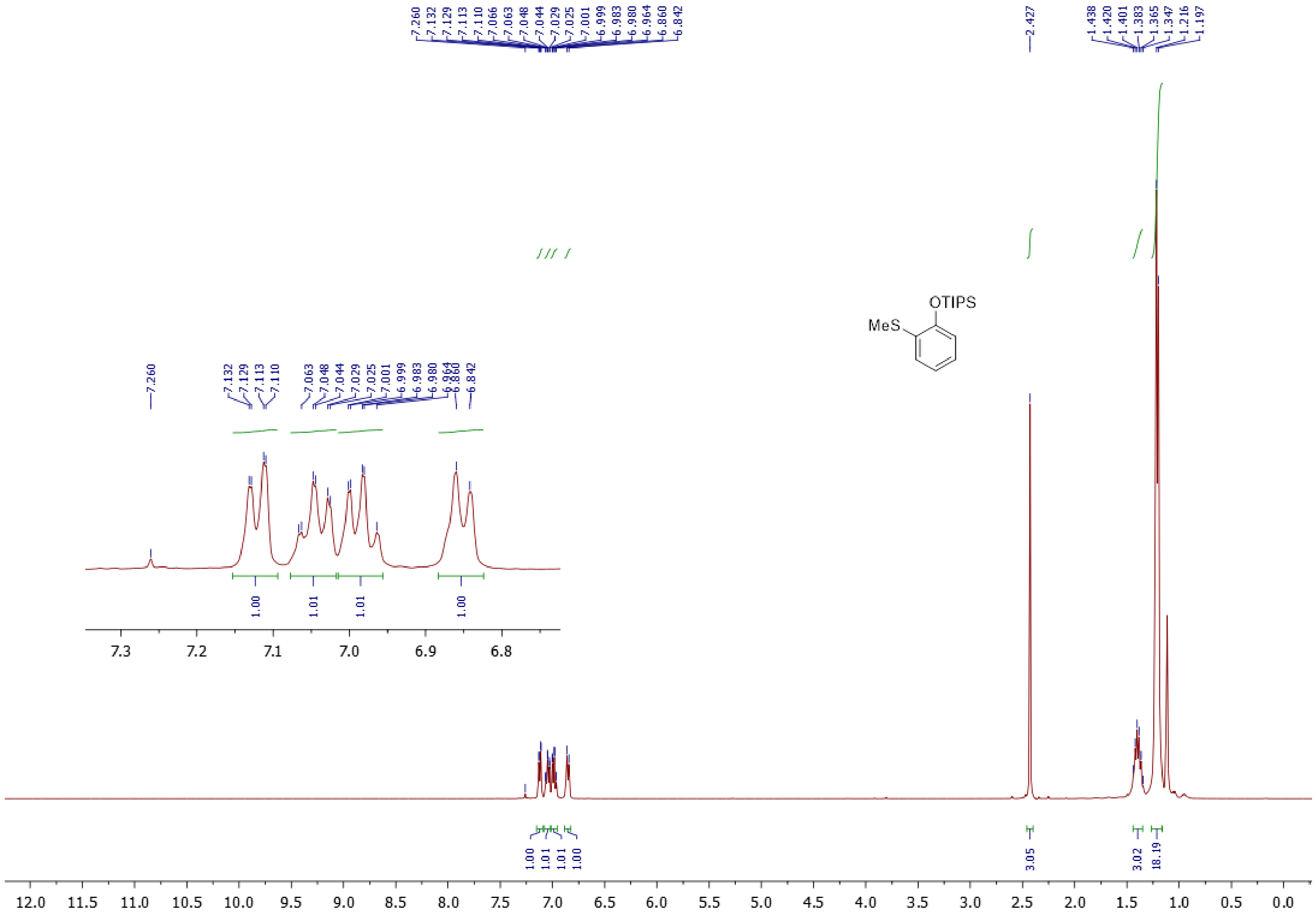

^{1}H-NMR spectra of **4k** (25 ^{o}C, 400 MHz, $CDCl_3$)

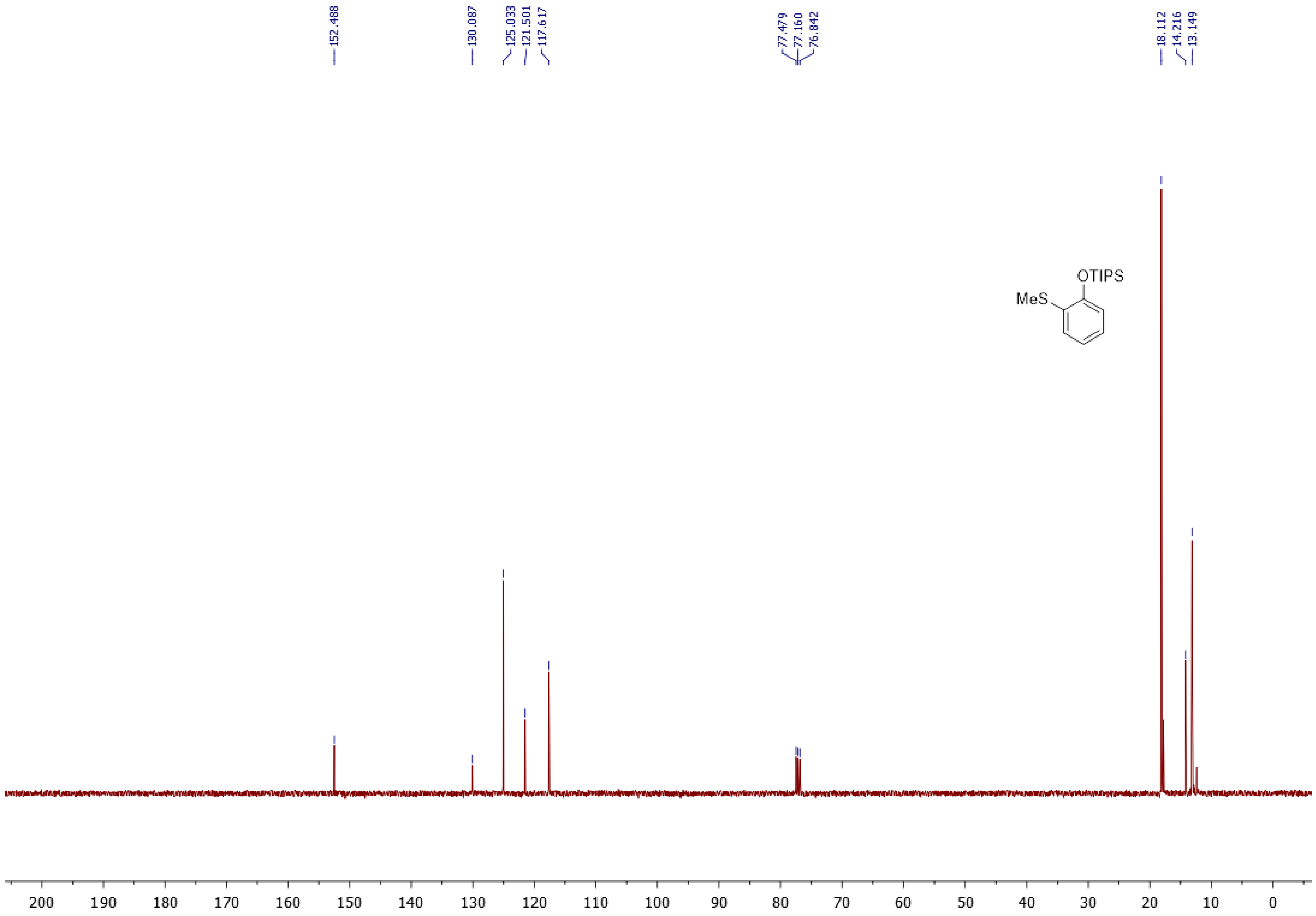

^{13}C-NMR spectra of **4k** (25 ºC, 100 MHz, $CDCl_3$)

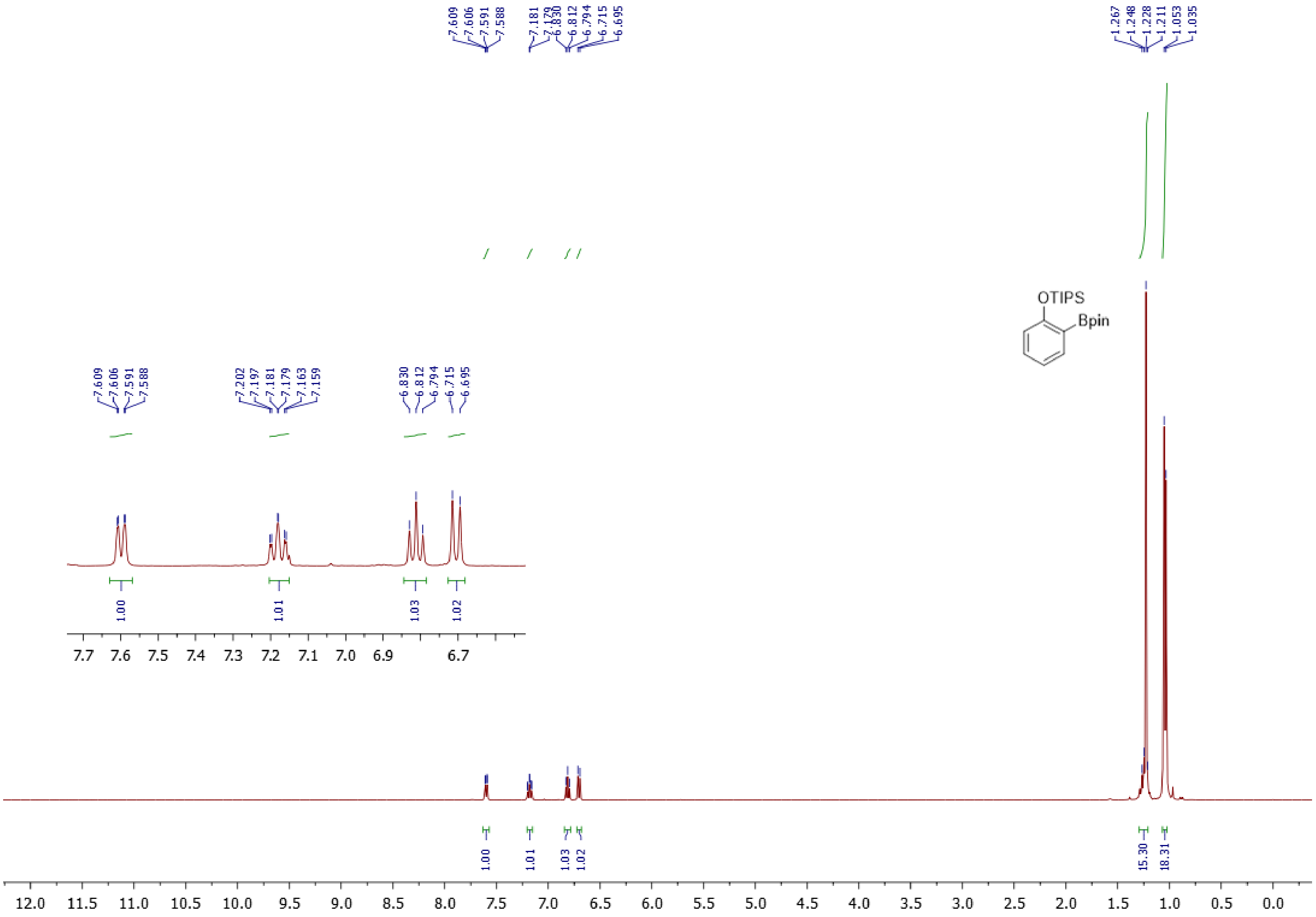

^{1}H-NMR spectra of **4m** (25 °C, 400 MHz, $CDCl_3$)

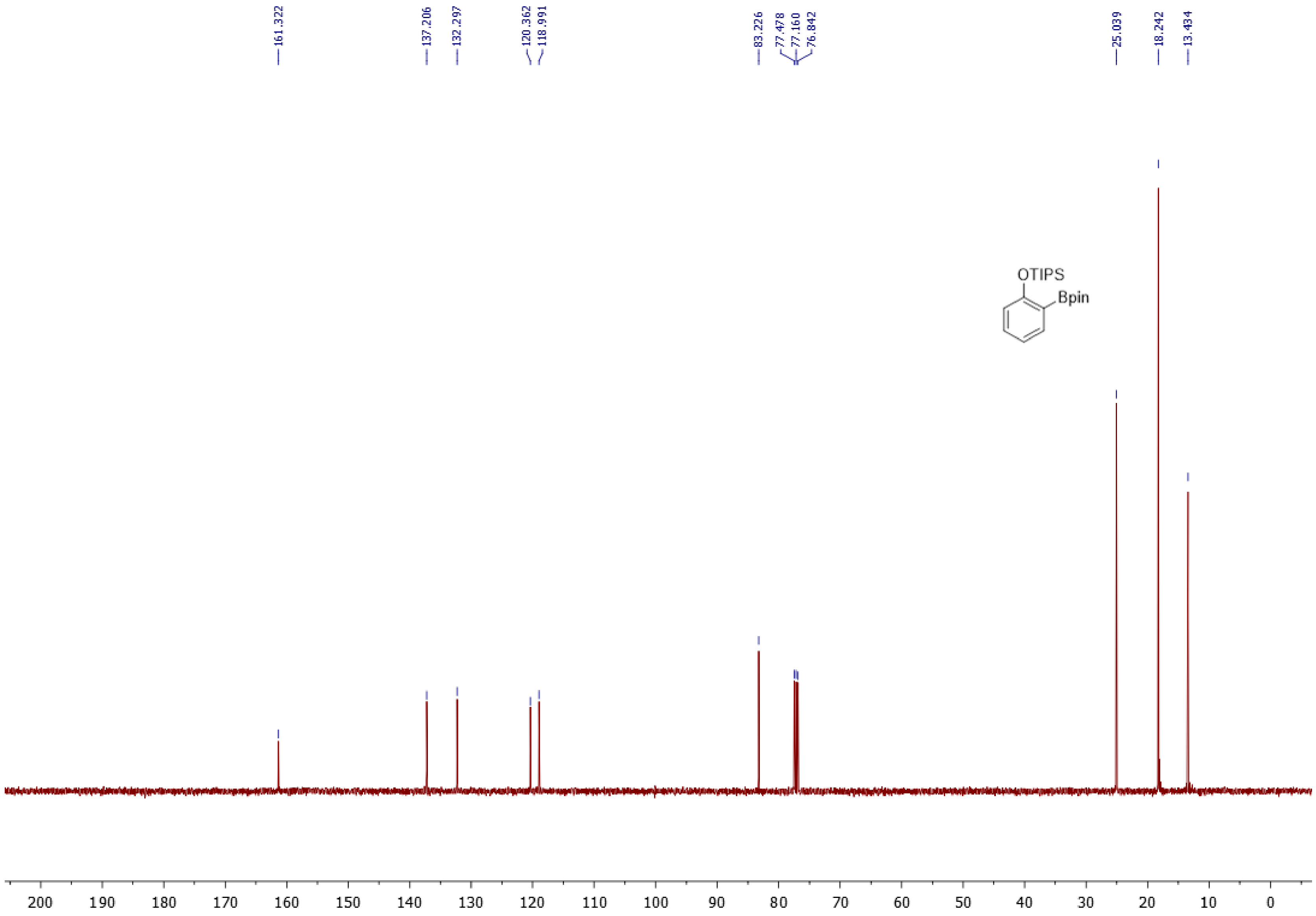

^{13}C-NMR spectra of **4m** (25 ^{o}C, 100 MHz, $CDCl_3$)

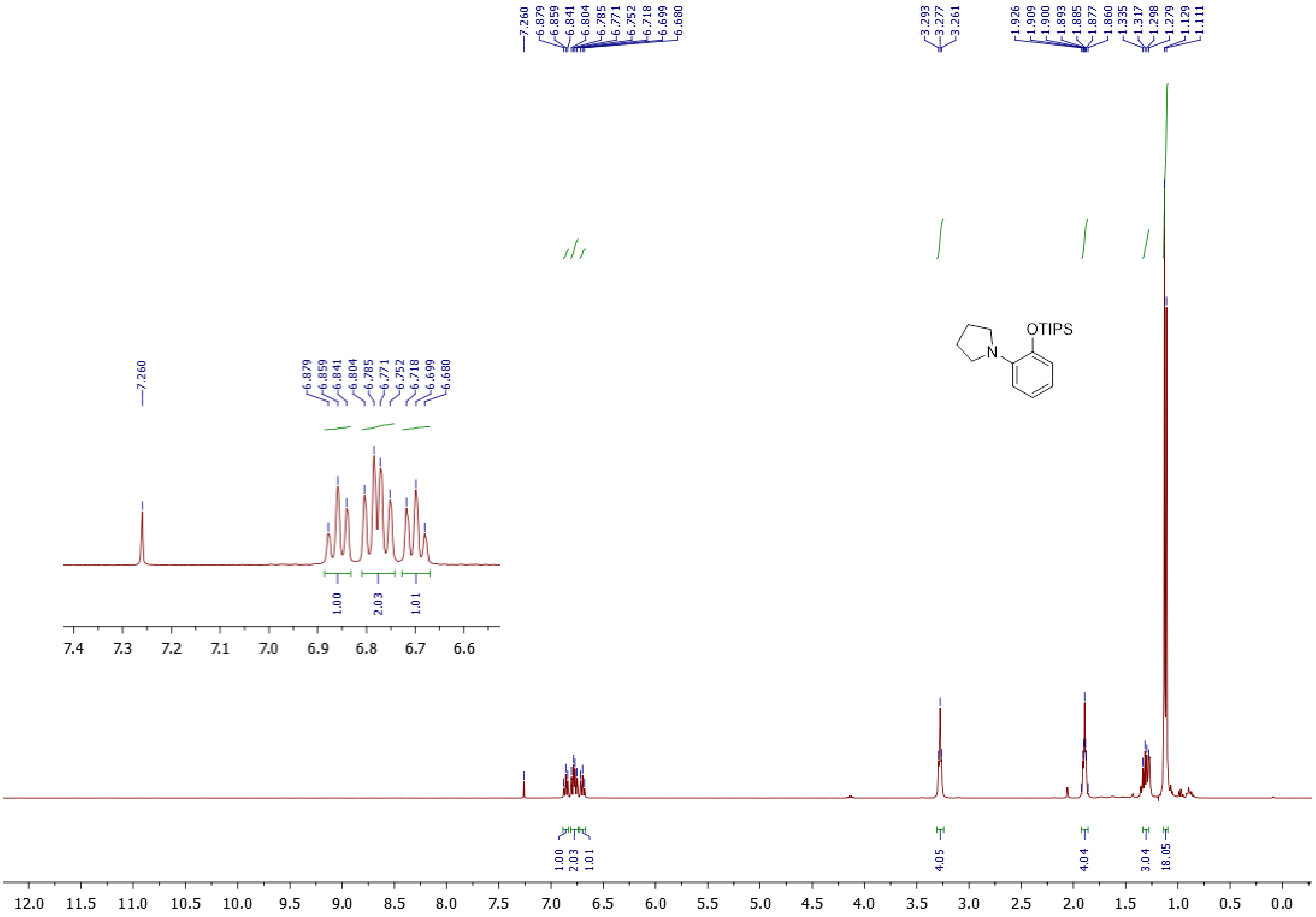

^{1}H-NMR spectra of **4n** (25 ^{o}C, 400 MHz, $CDCl_3$)

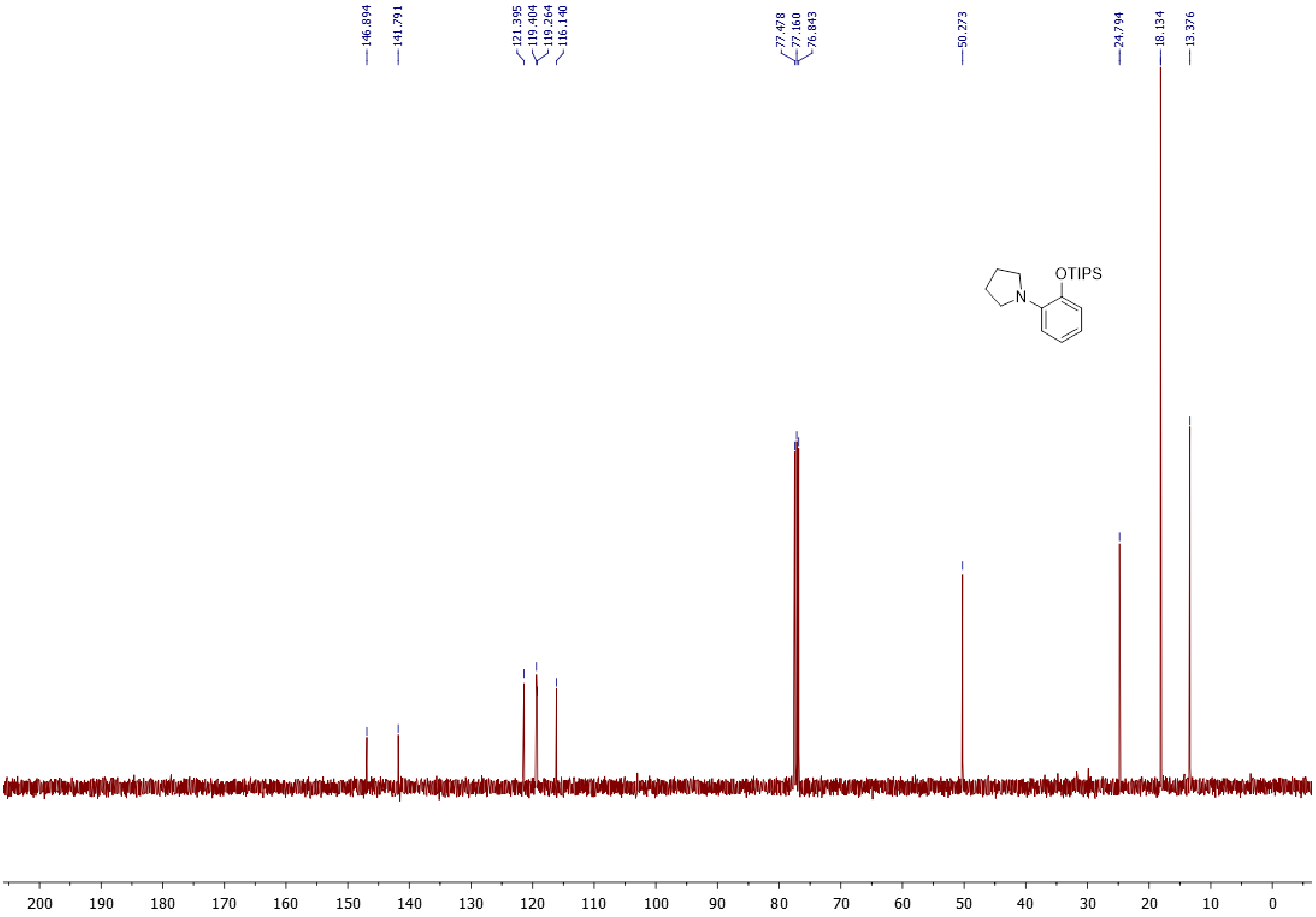

^{13}C-NMR spectra of **4n** (25 °C, 100 MHz, $CDCl_3$)

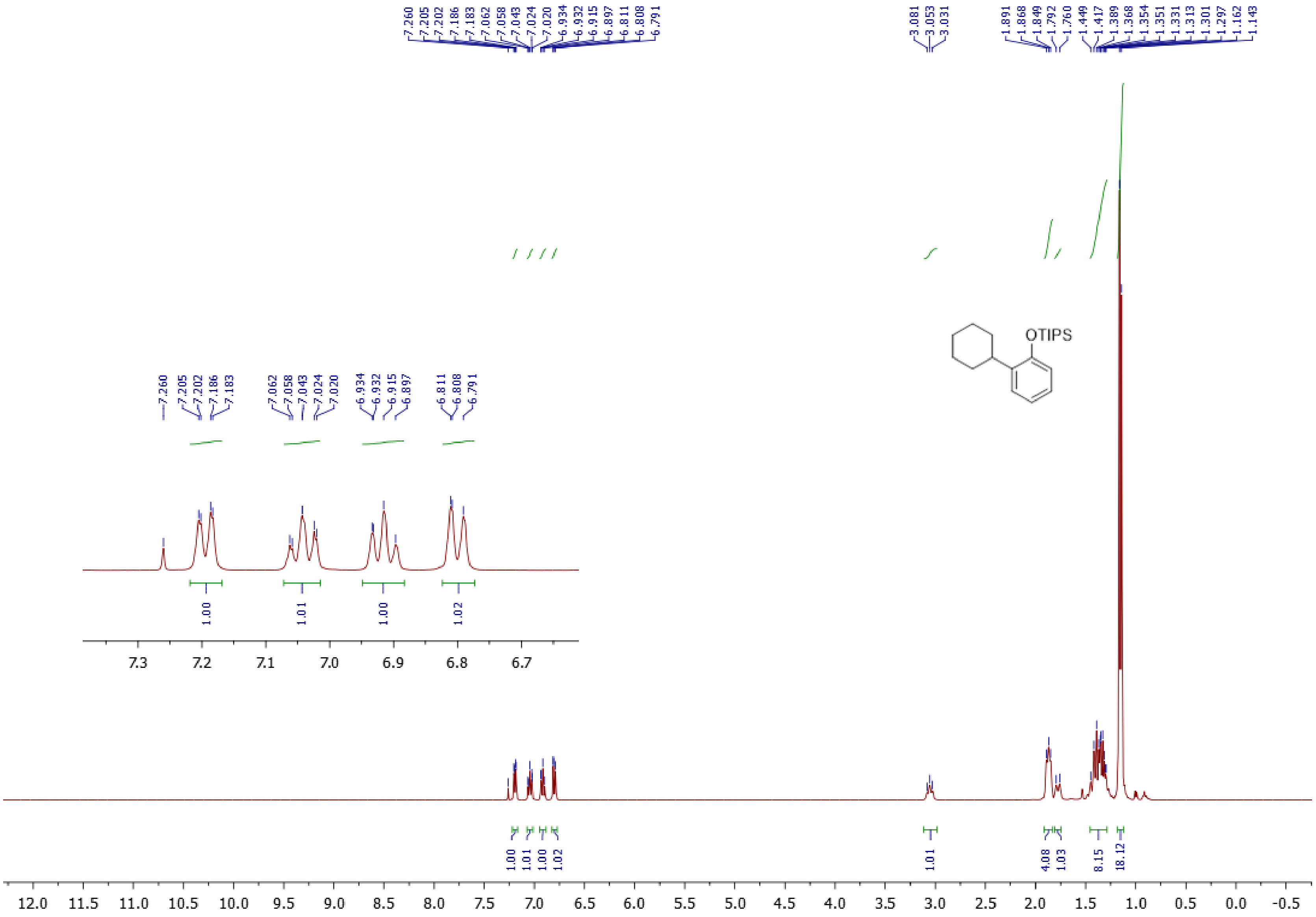

^{1}H-NMR spectra of **4o** (25 ^{o}C, 400 MHz, $CDCl_3$)

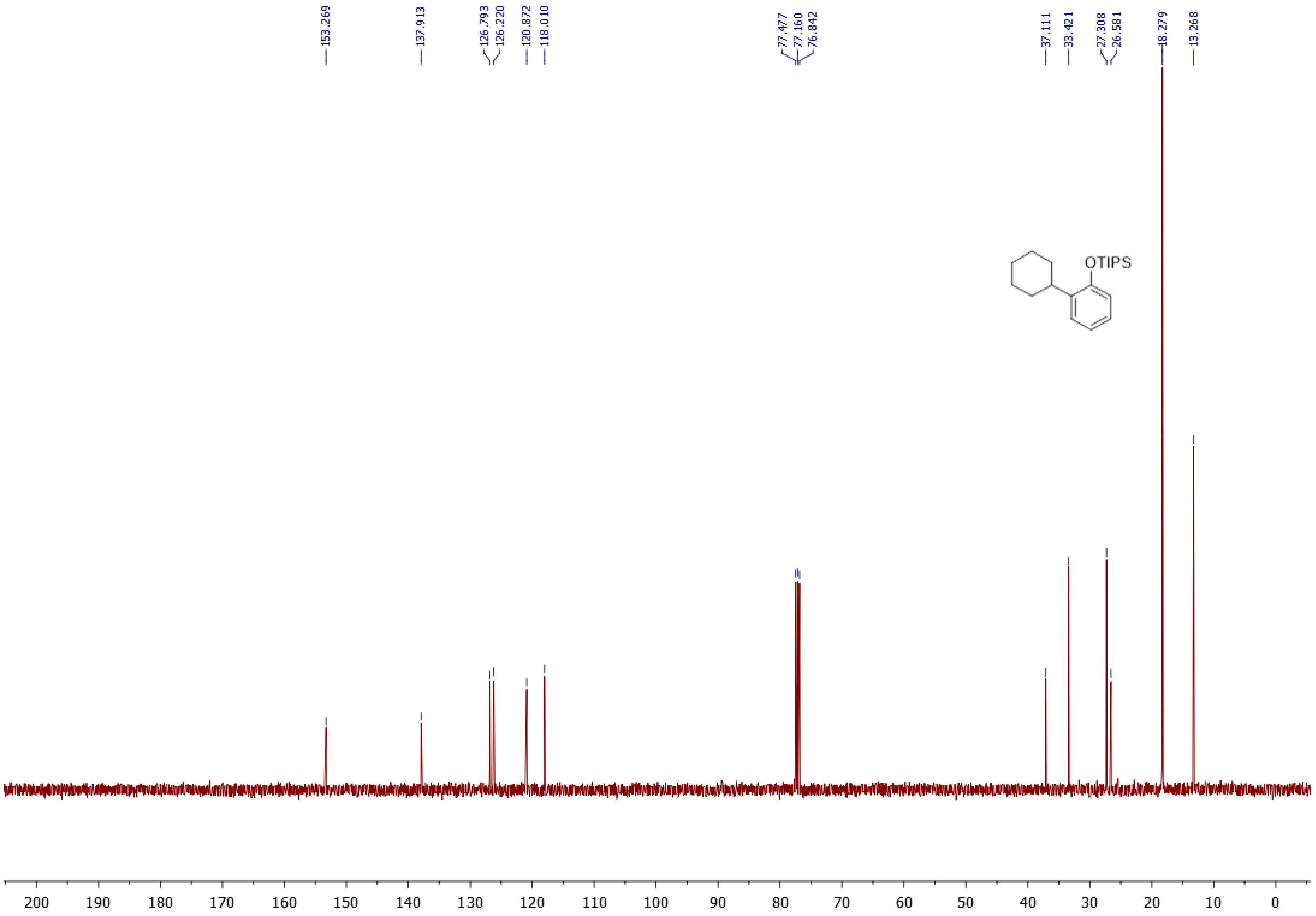

^{13}C-NMR spectra of **4o** (25 °C, 100 MHz, $CDCl_3$)

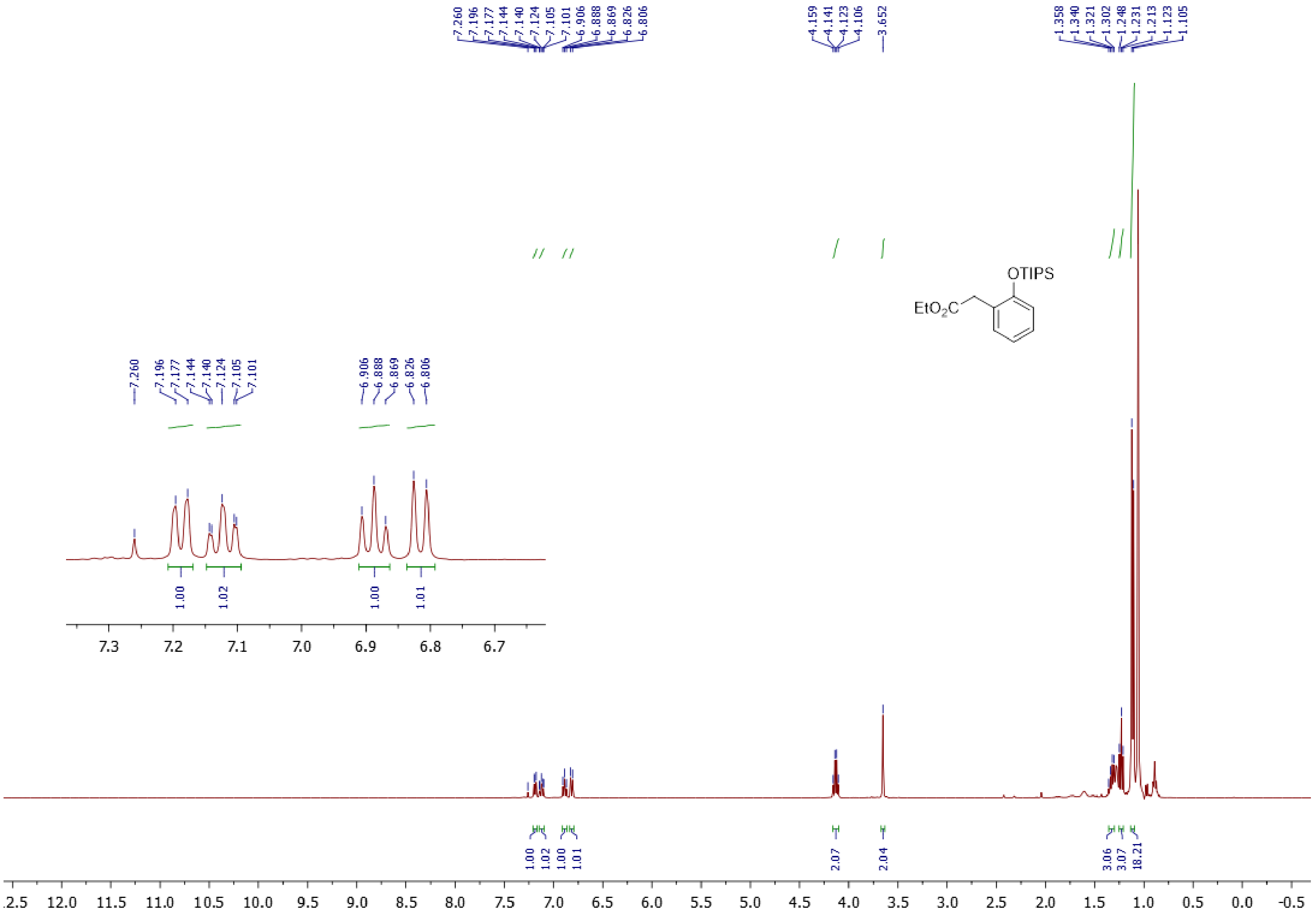

^{1}H-NMR spectra of **4q** (25 ºC, 400 MHz, $CDCl_3$)

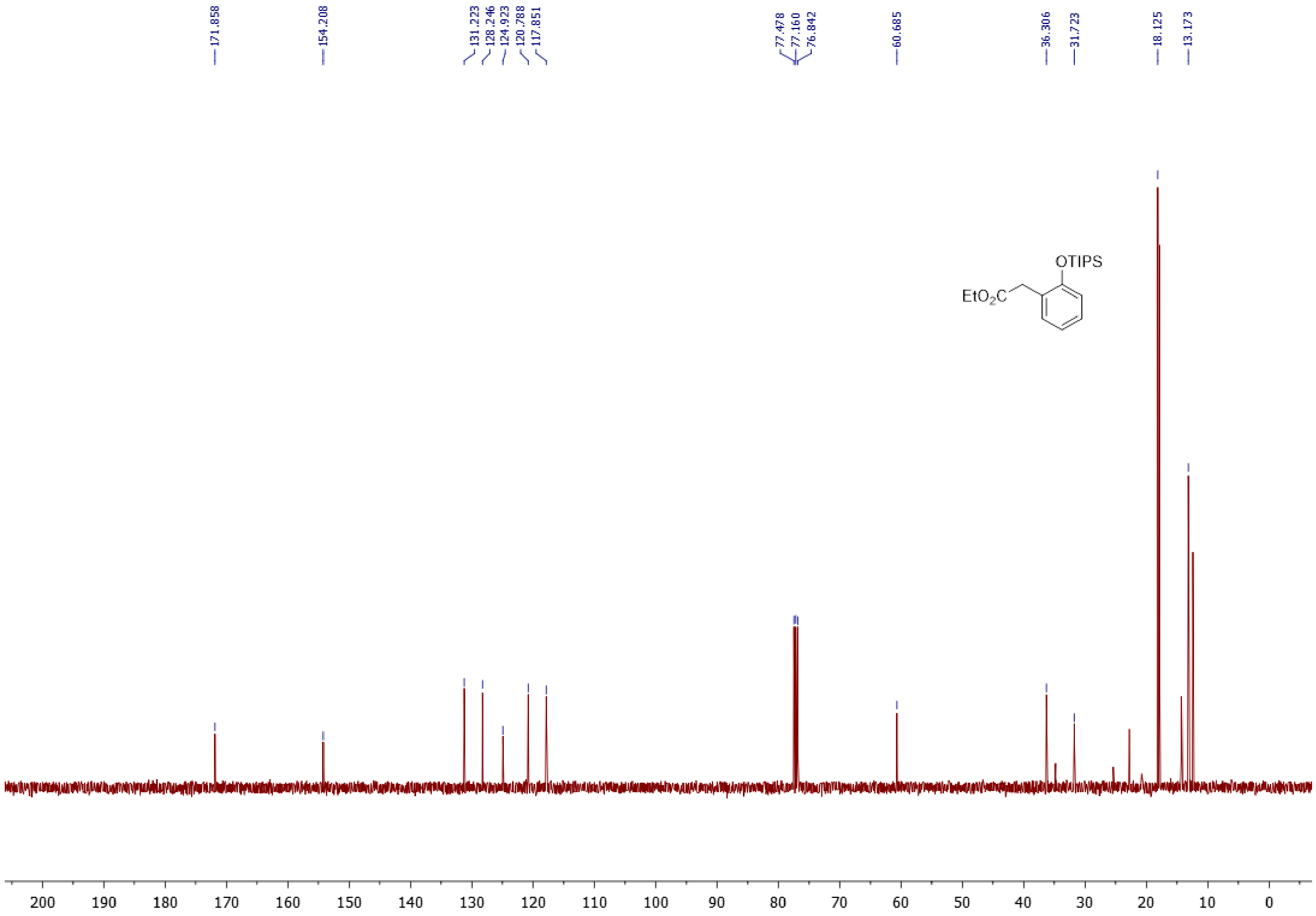

^{13}C-NMR spectra of **4q** (25 °C, 100 MHz, $CDCl_3$)

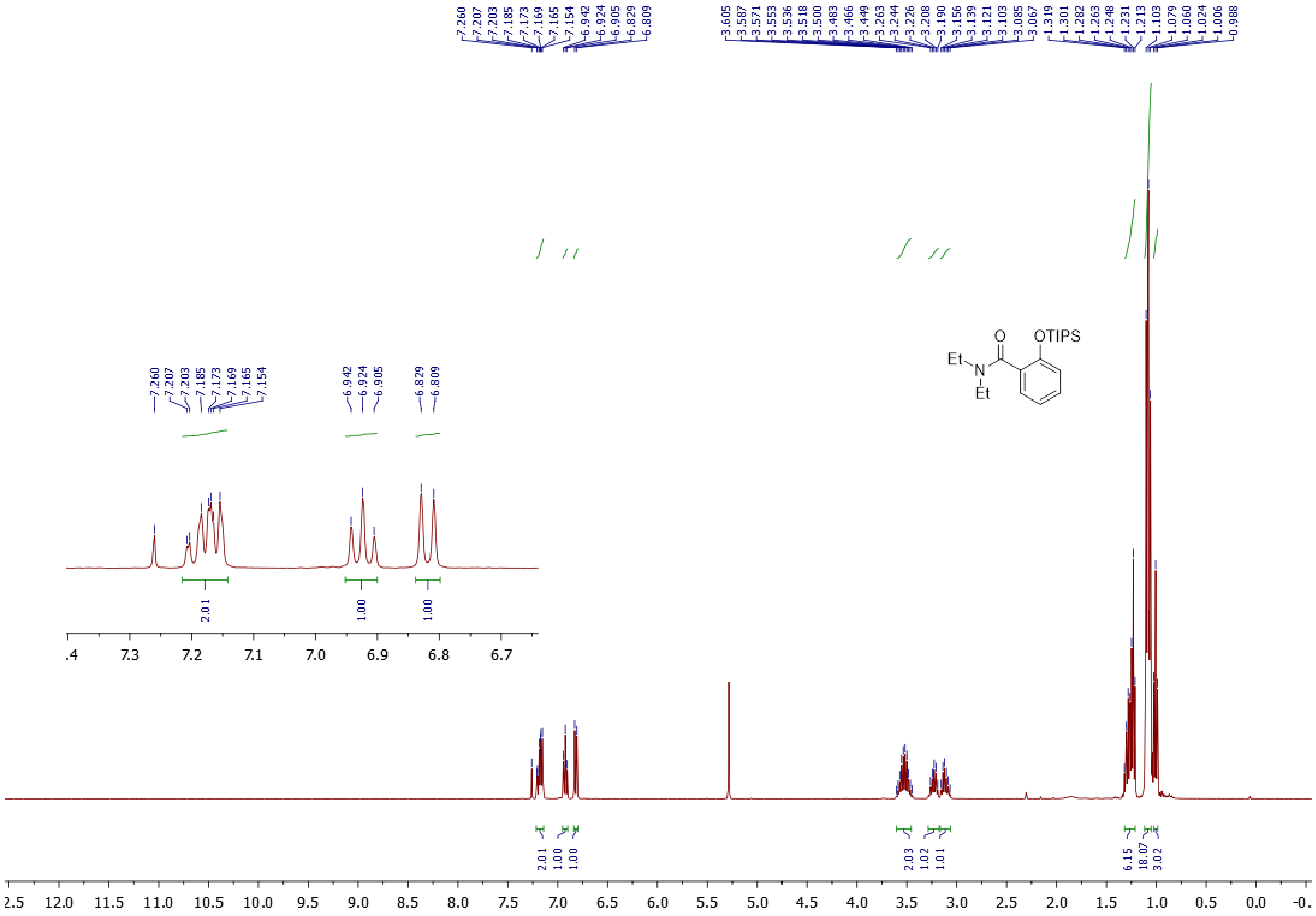

^{1}H-NMR spectra of **4r** (25 °C, 400 MHz, $CDCl_3$)

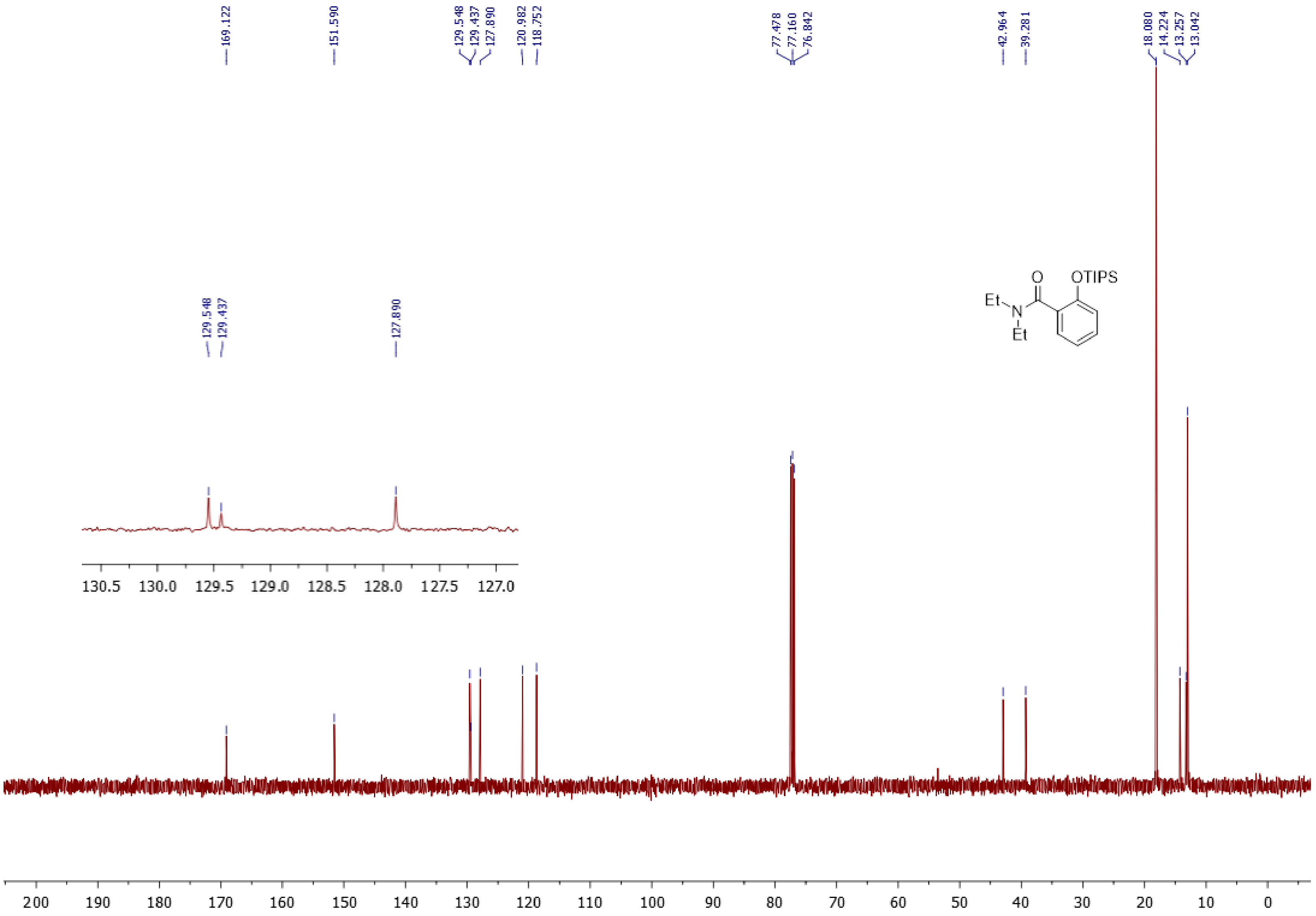

^{13}C-NMR spectra of **4r** (25 ºC, 100 MHz, $CDCl_3$)

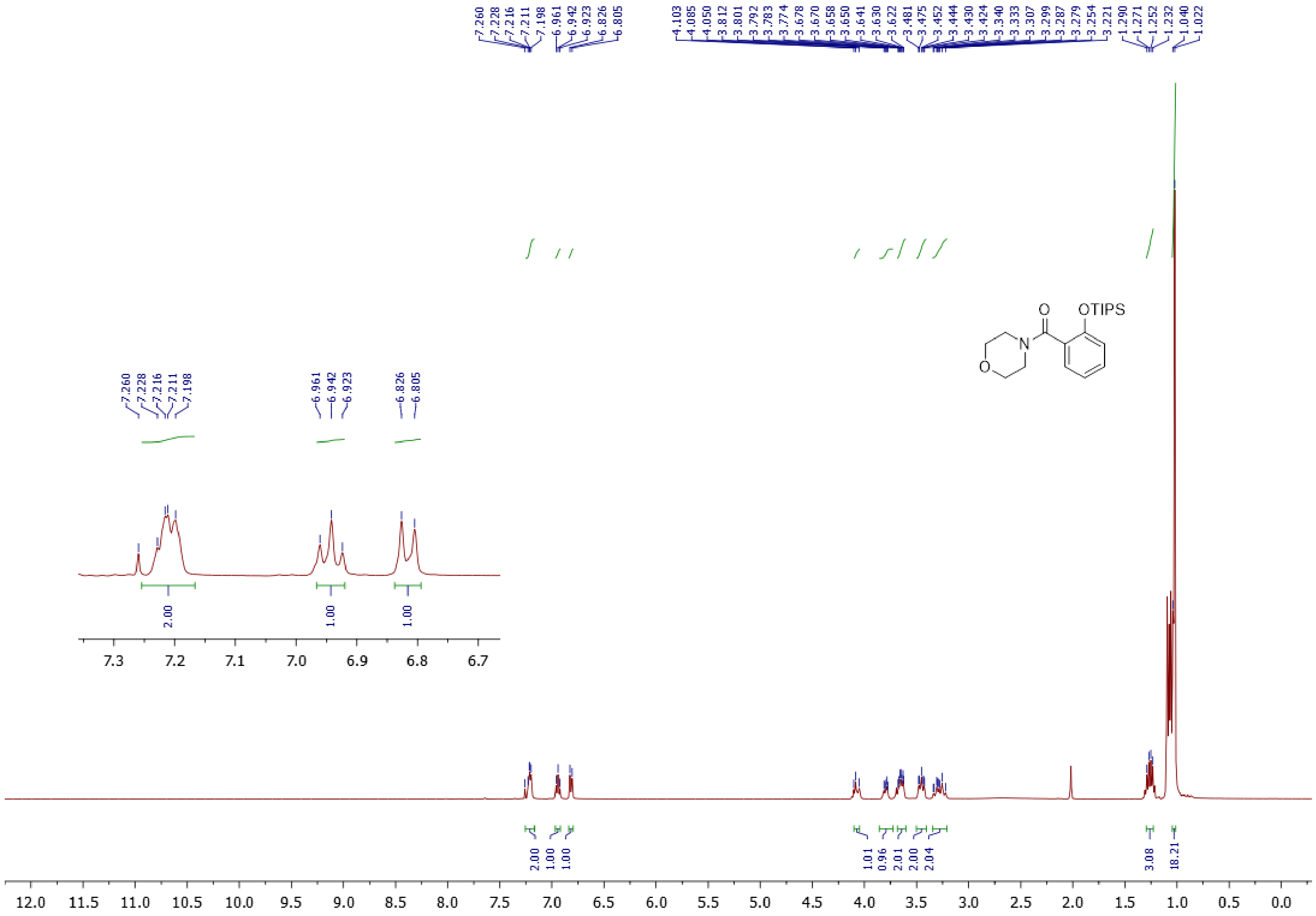

^{1}H-NMR spectra of **4s** (25 °C, 400 MHz, $CDCl_3$)

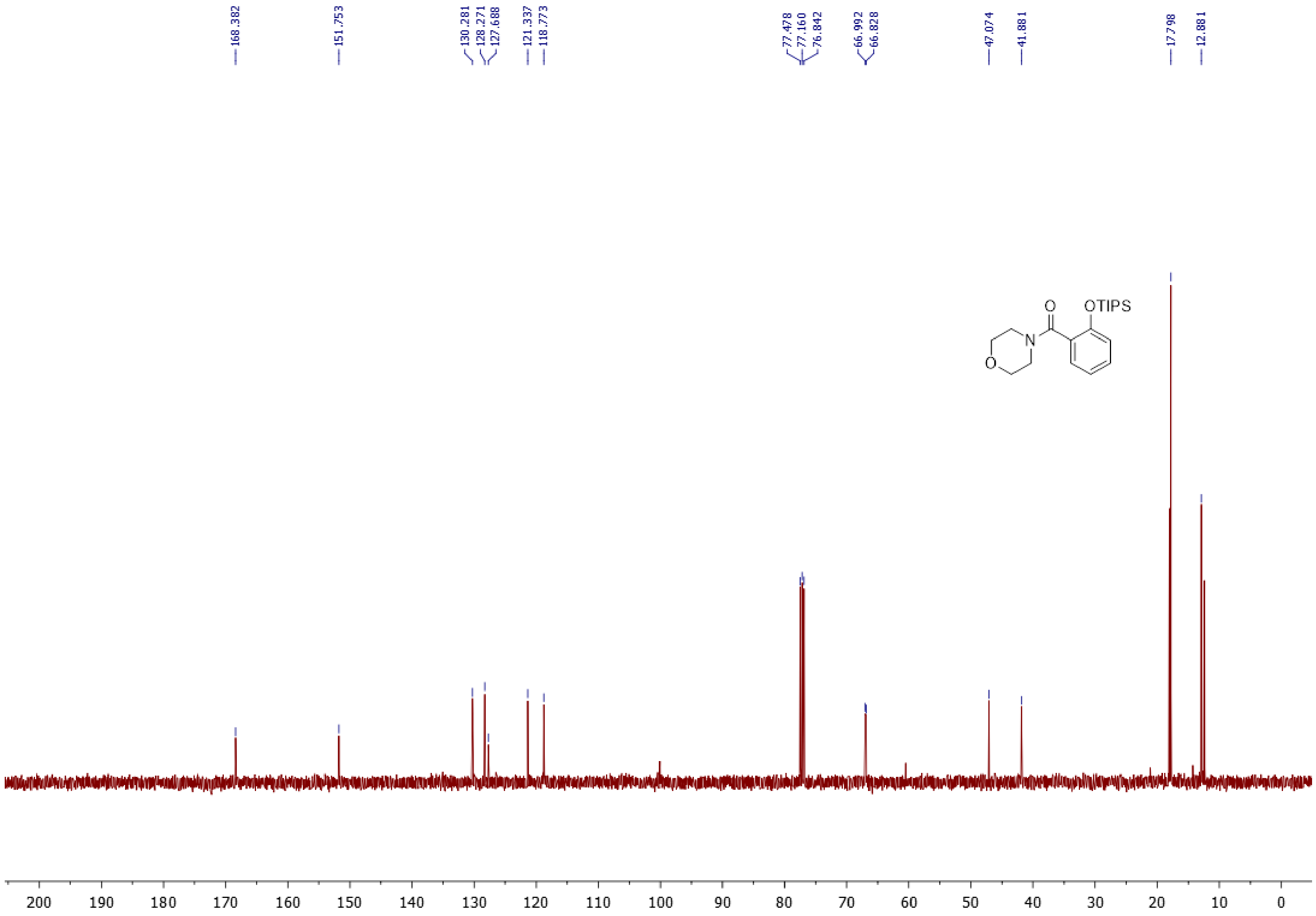

^{13}C-NMR spectra of **4s** (25 ºC, 100 MHz, $CDCl_3$)

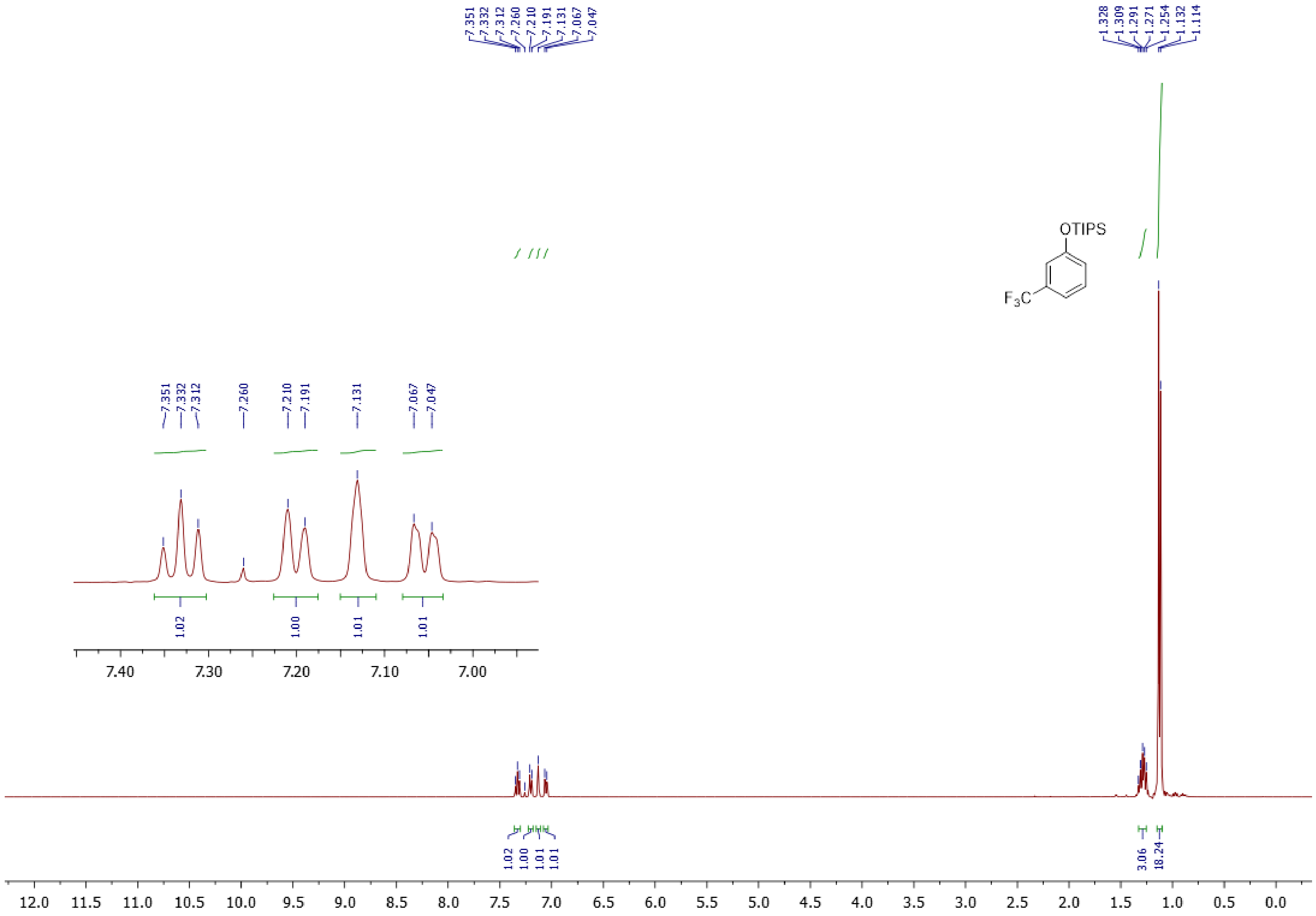

^{1}H-NMR spectra of **4t** (25 °C, 400 MHz, $CDCl_3$)

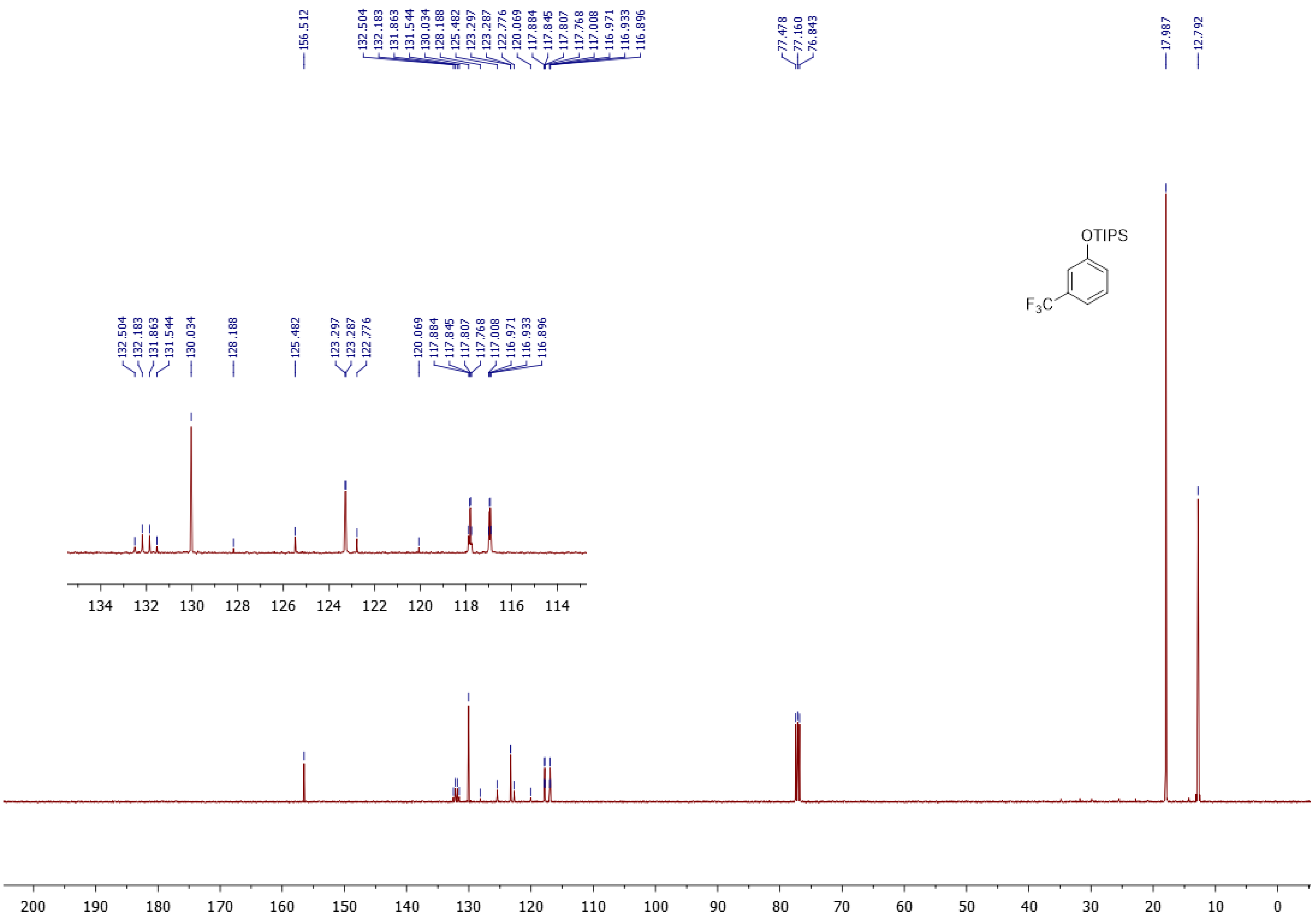

^{13}C-NMR spectra of **4t** (25 ºC, 100 MHz, $CDCl_3$)

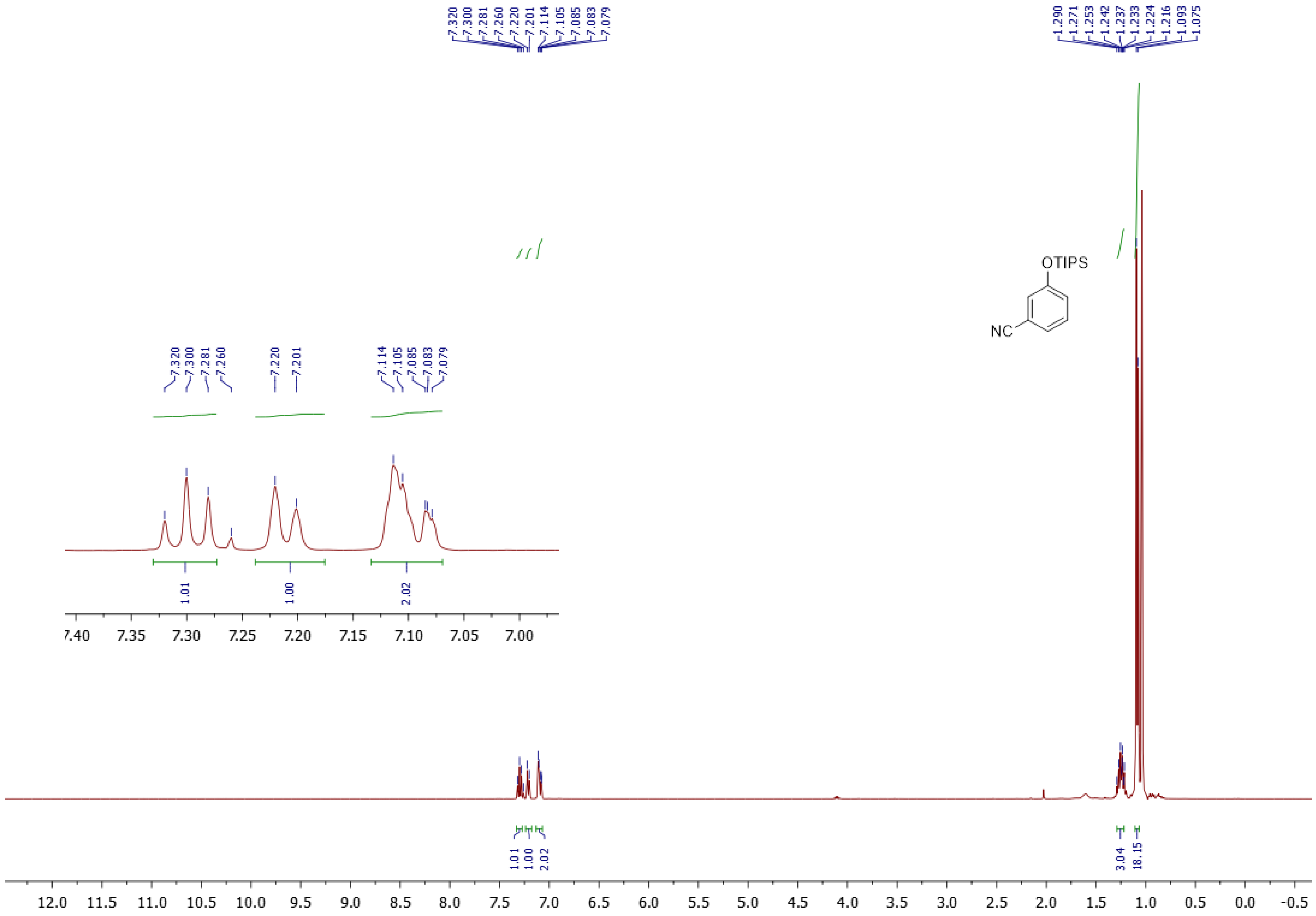

^{1}H-NMR spectra of **4u** (25 ^{o}C, 400 MHz, $CDCl_3$)

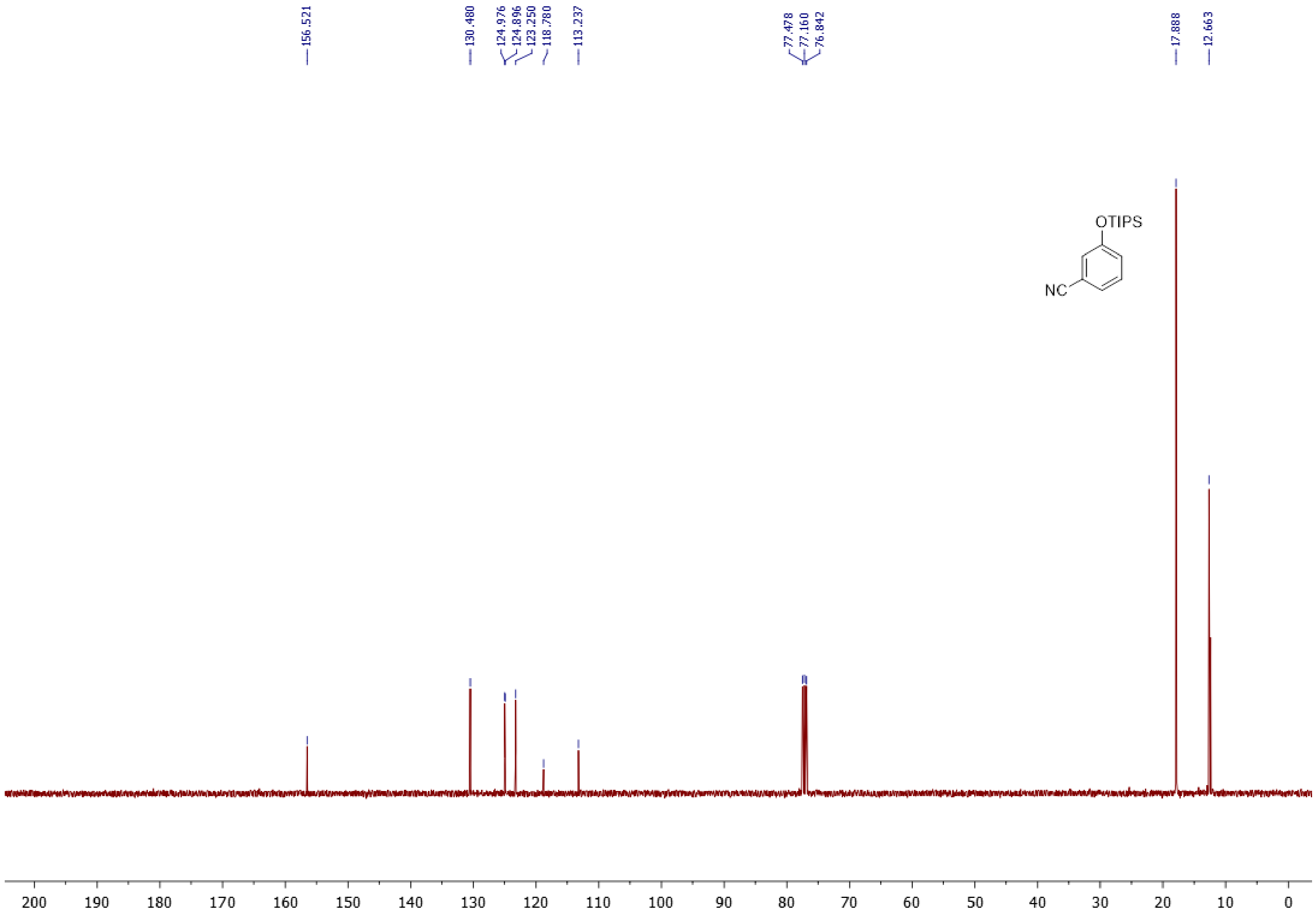

^{13}C-NMR spectra of **4u** (25 °C, 100 MHz, $CDCl_3$)

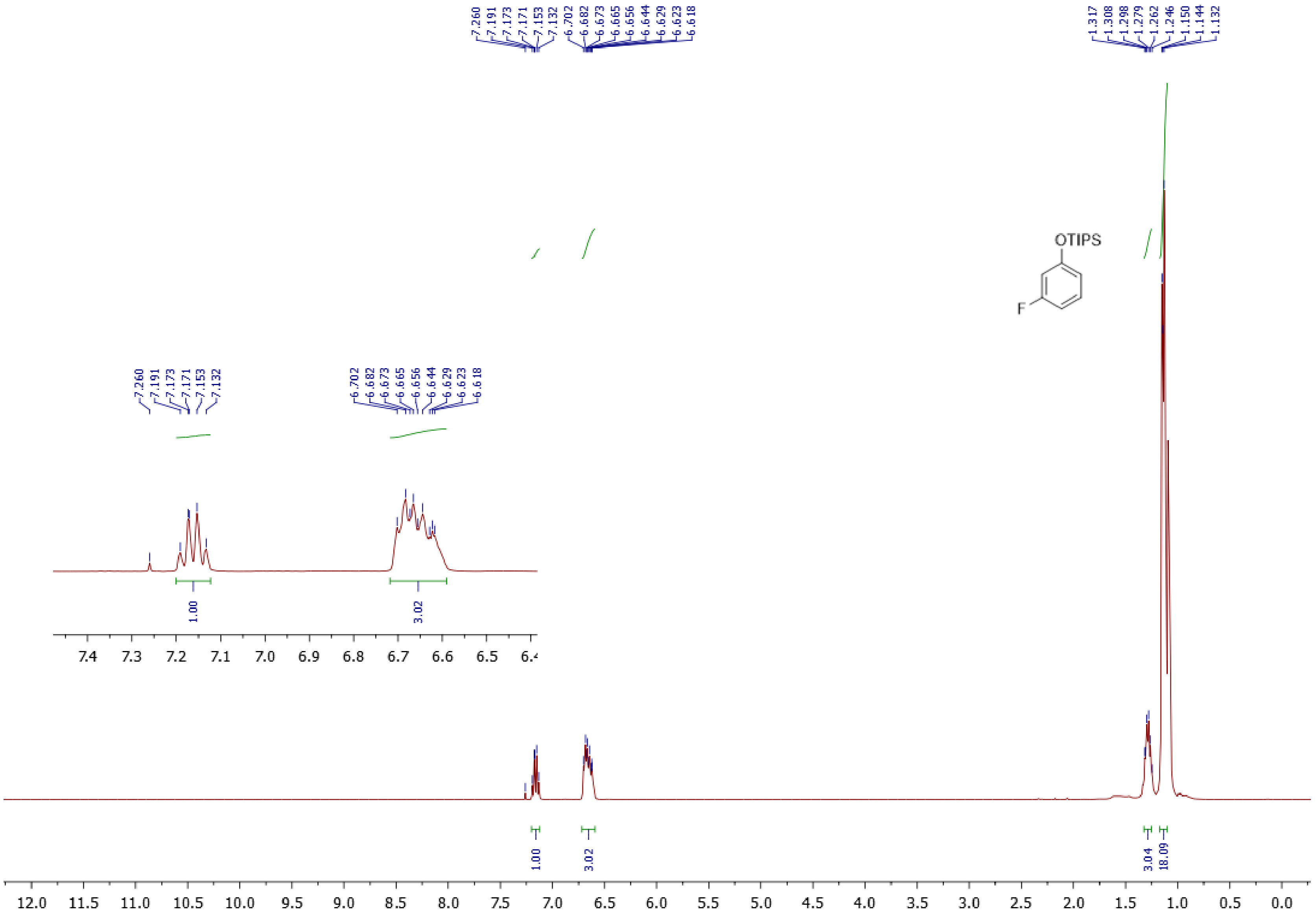

^{1}H-NMR spectra of **4v** (25 °C, 400 MHz, $CDCl_3$)

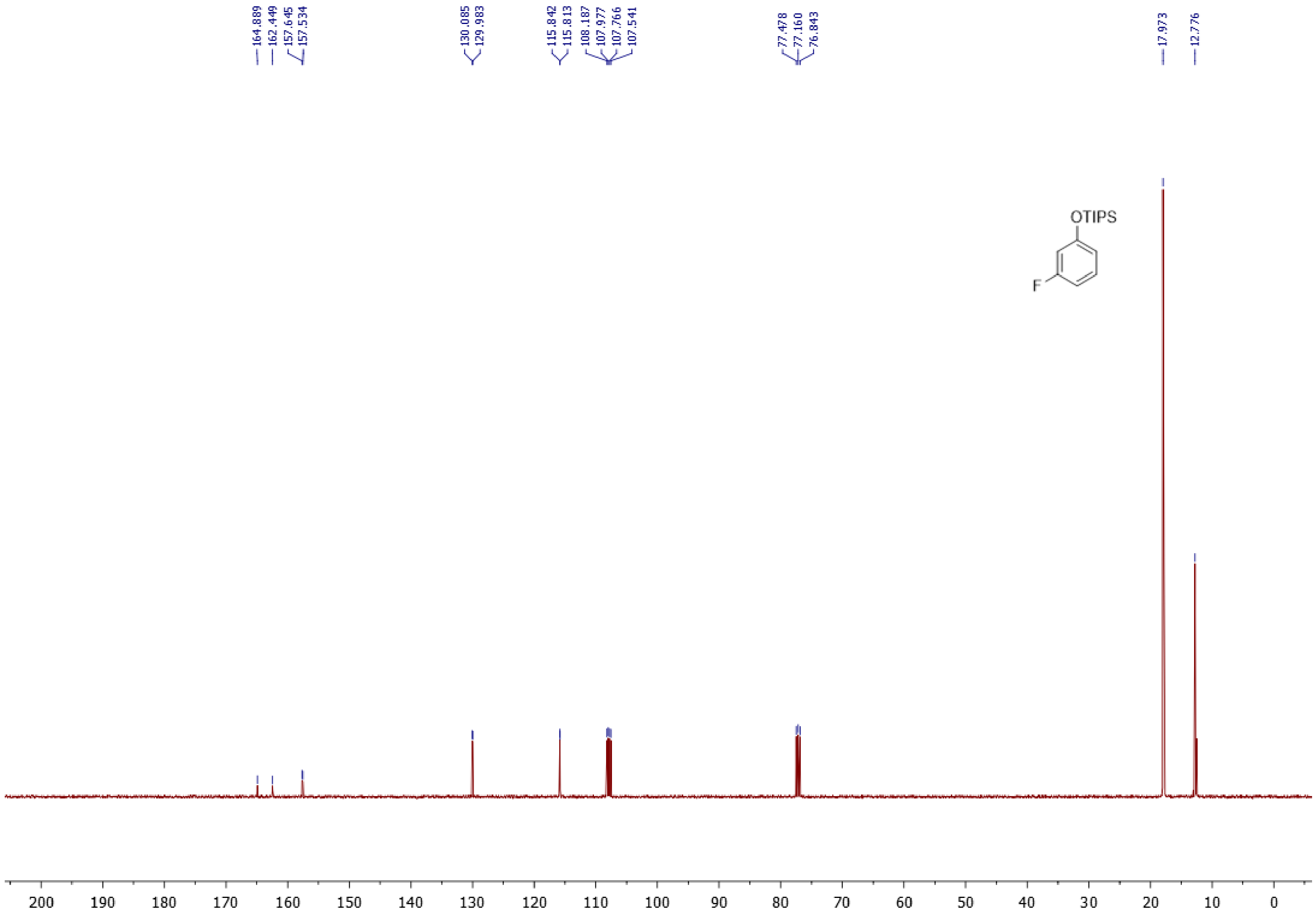

^{13}C-NMR spectra of **4v** (25 °C, 100 MHz, $CDCl_3$)

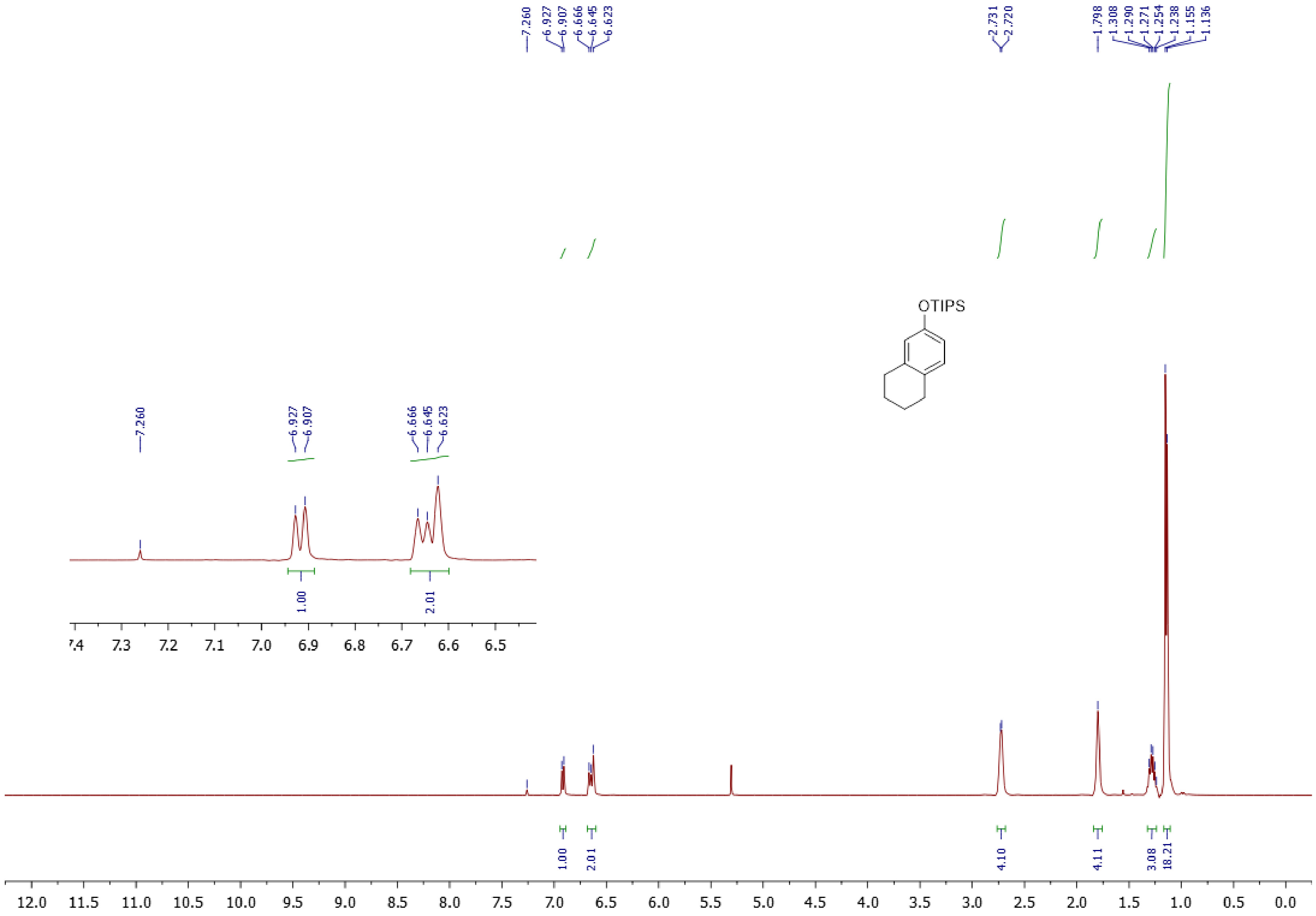

^{1}H-NMR spectra of **4w** (25 ºC, 400 MHz, $CDCl_3$)

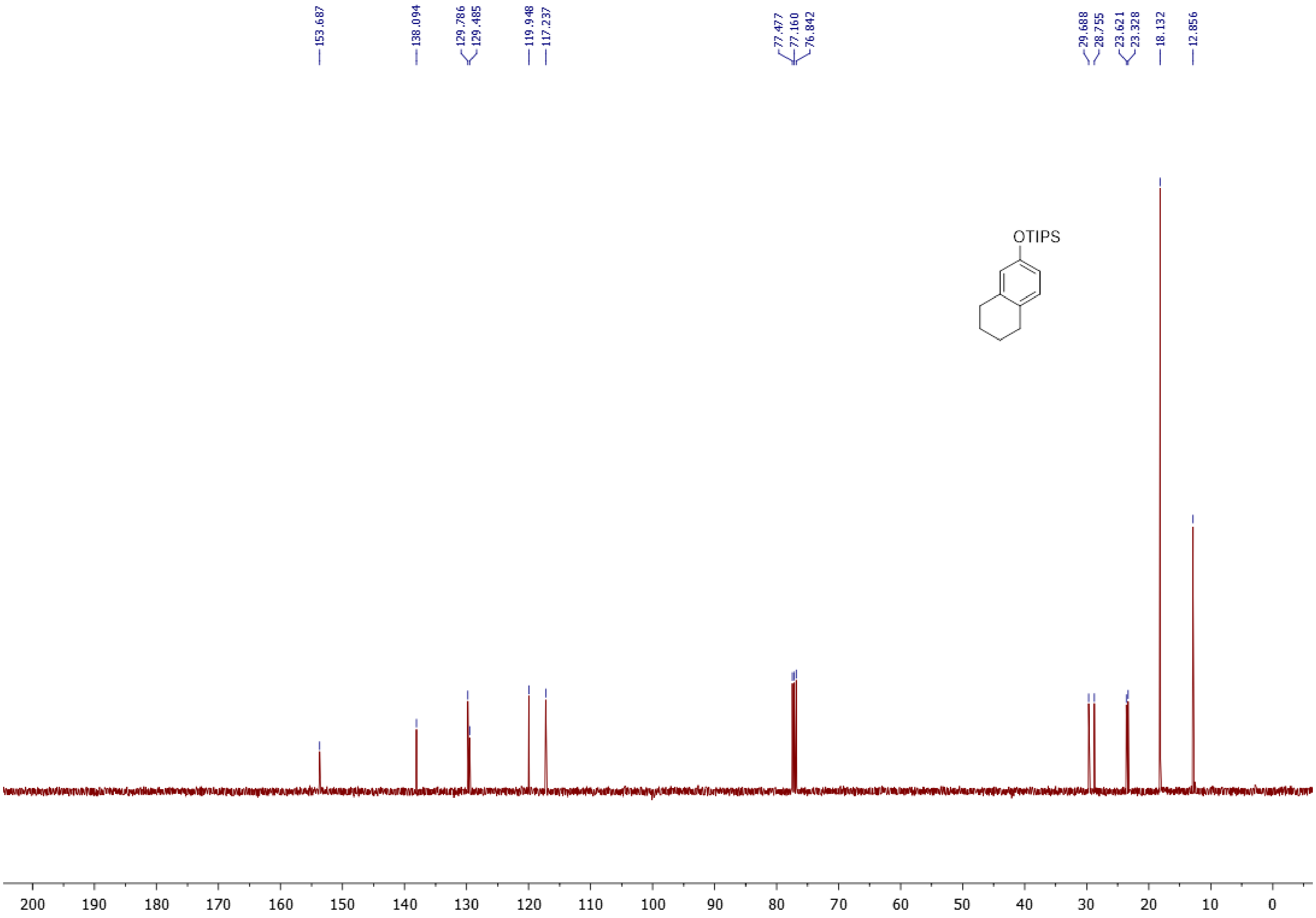

^{13}C-NMR spectra of **4w** (25 ^{o}C, 100 MHz, $CDCl_3$)

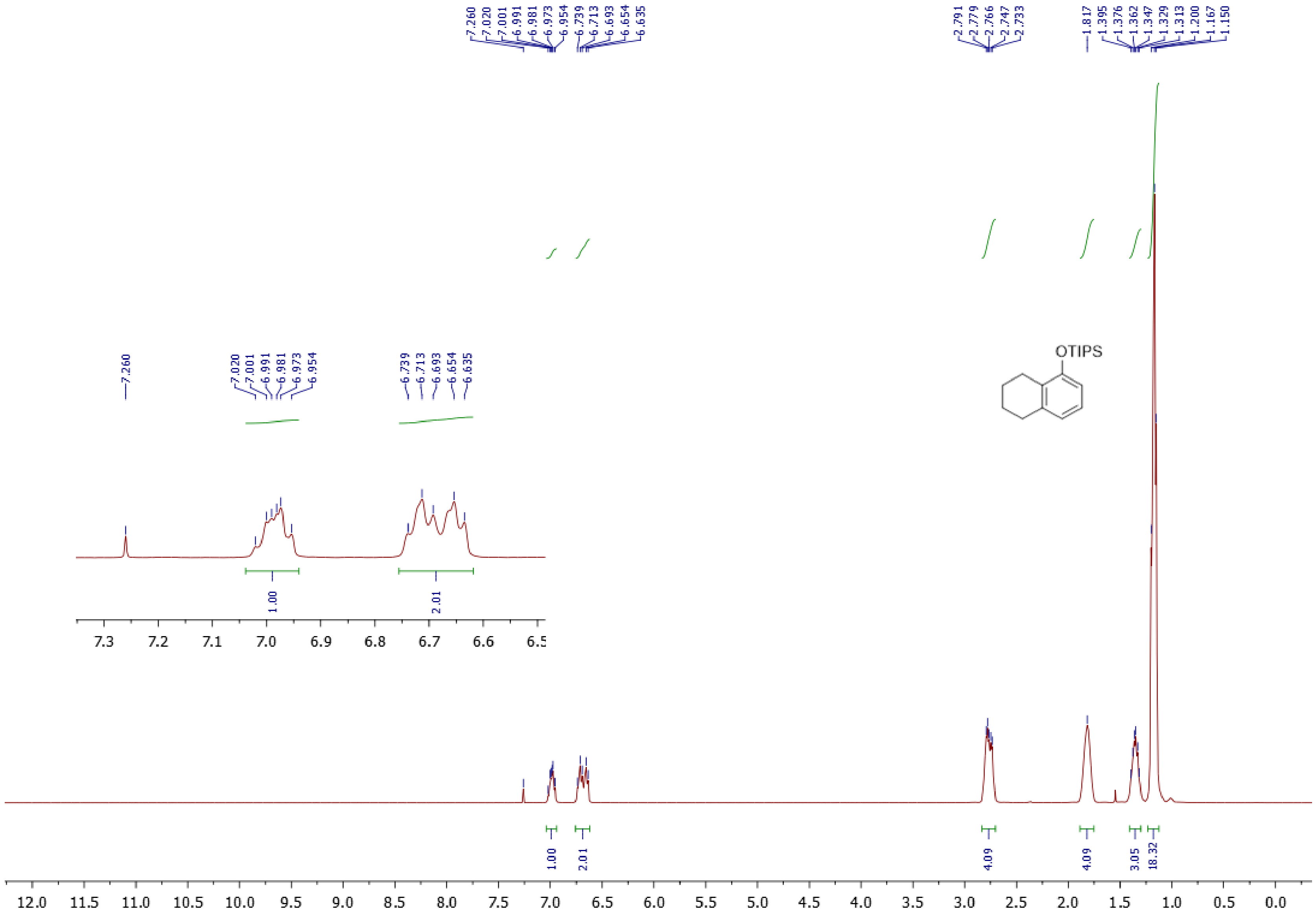

^{1}H-NMR spectra of **4x** (25 °C, 400 MHz, $CDCl_3$)

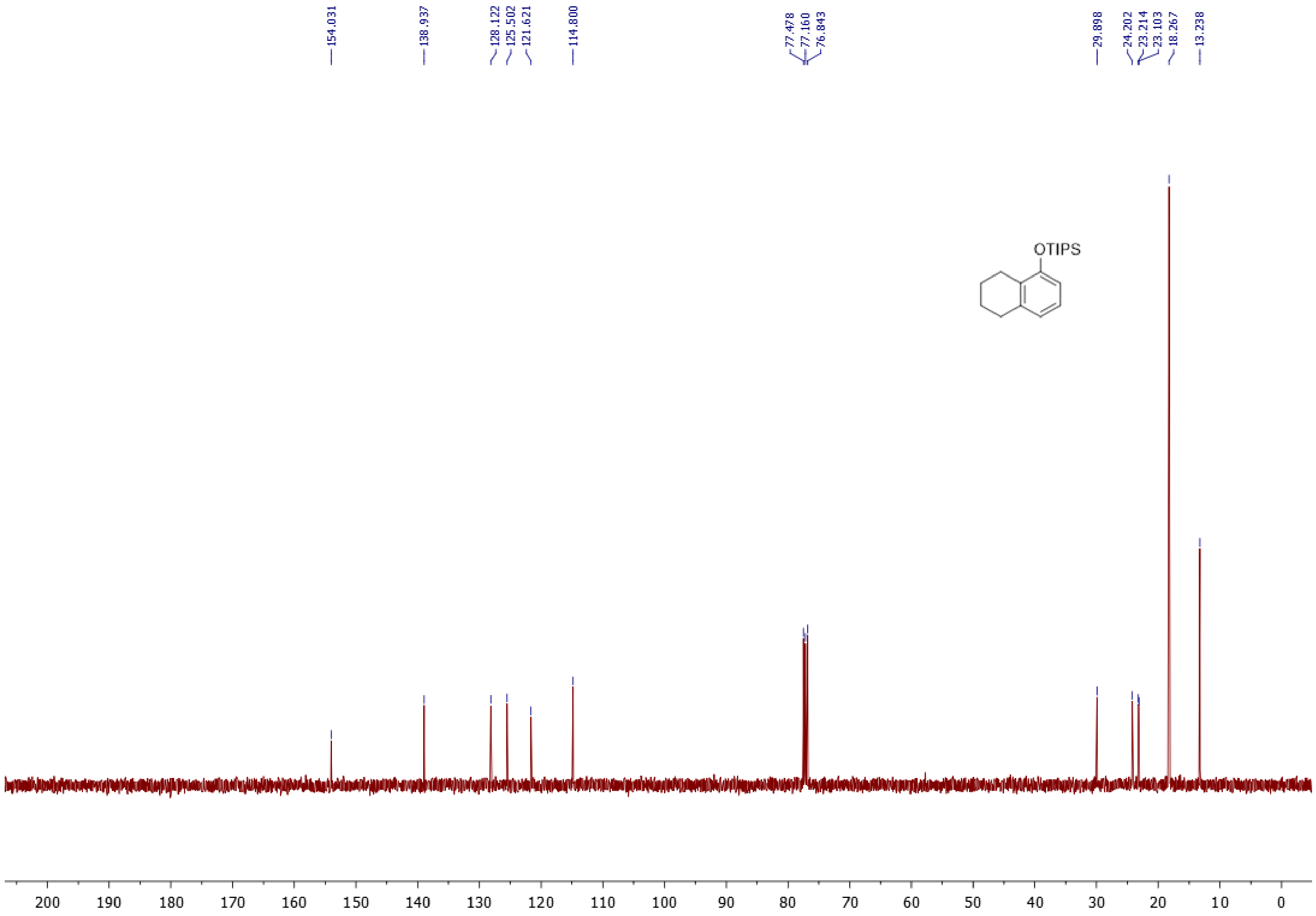

^{13}C-NMR spectra of **4x** (25 ºC, 100 MHz, $CDCl_3$)

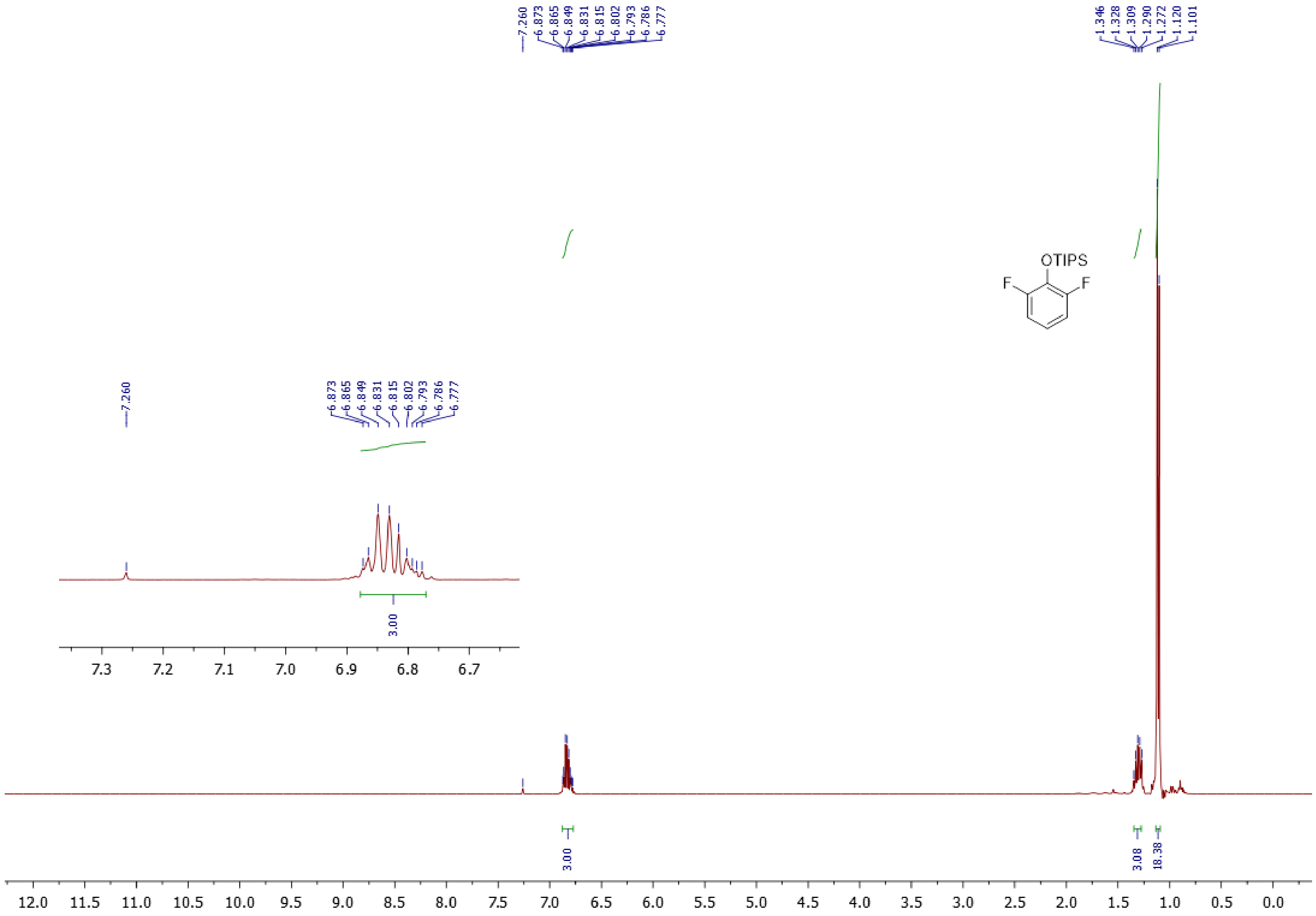

^{1}H-NMR spectra of **4y** (25 ^{o}C, 400 MHz, $CDCl_3$)

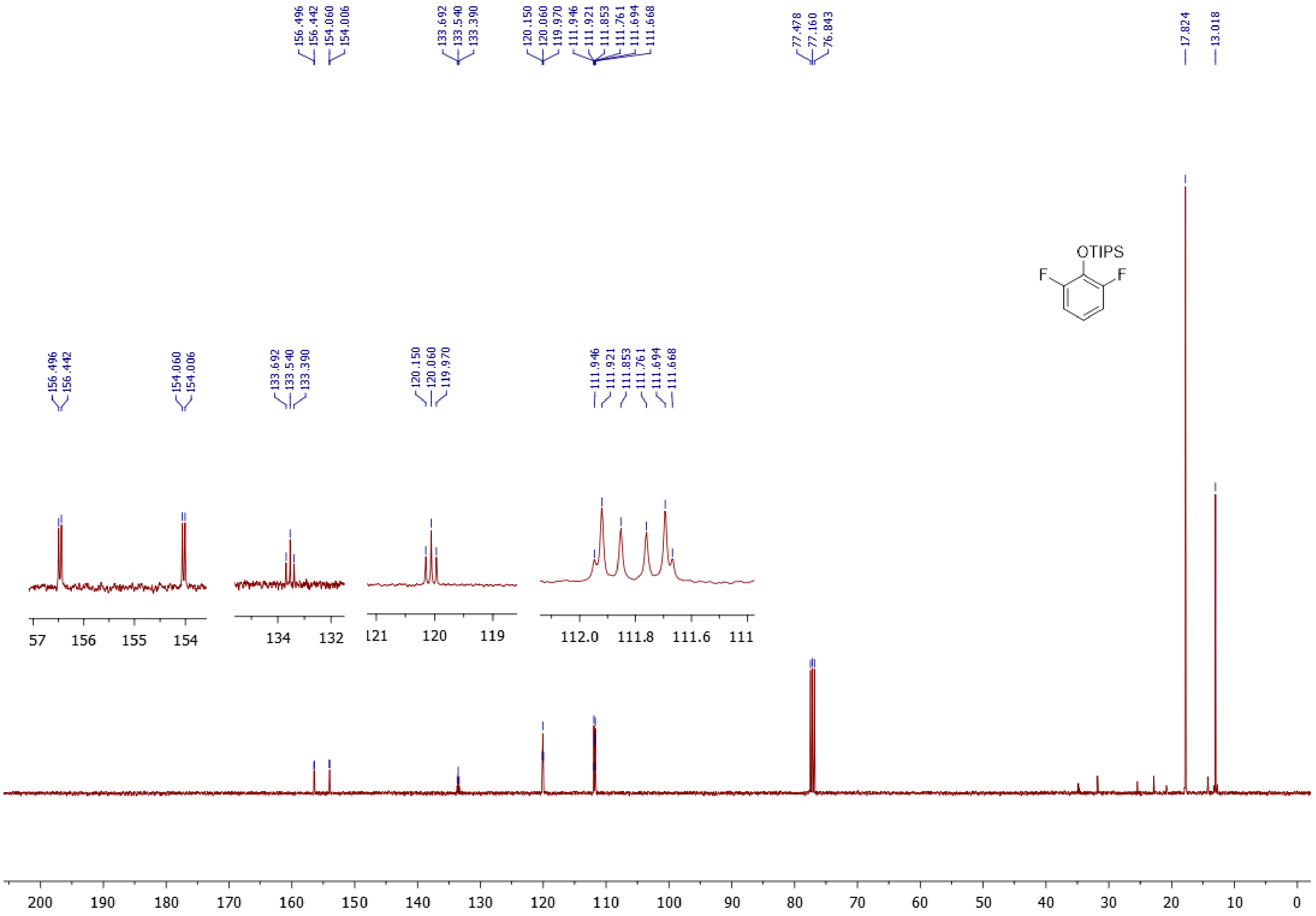

^{13}C-NMR spectra of **4y** (25 ^{o}C, 100 MHz, $CDCl_3$)

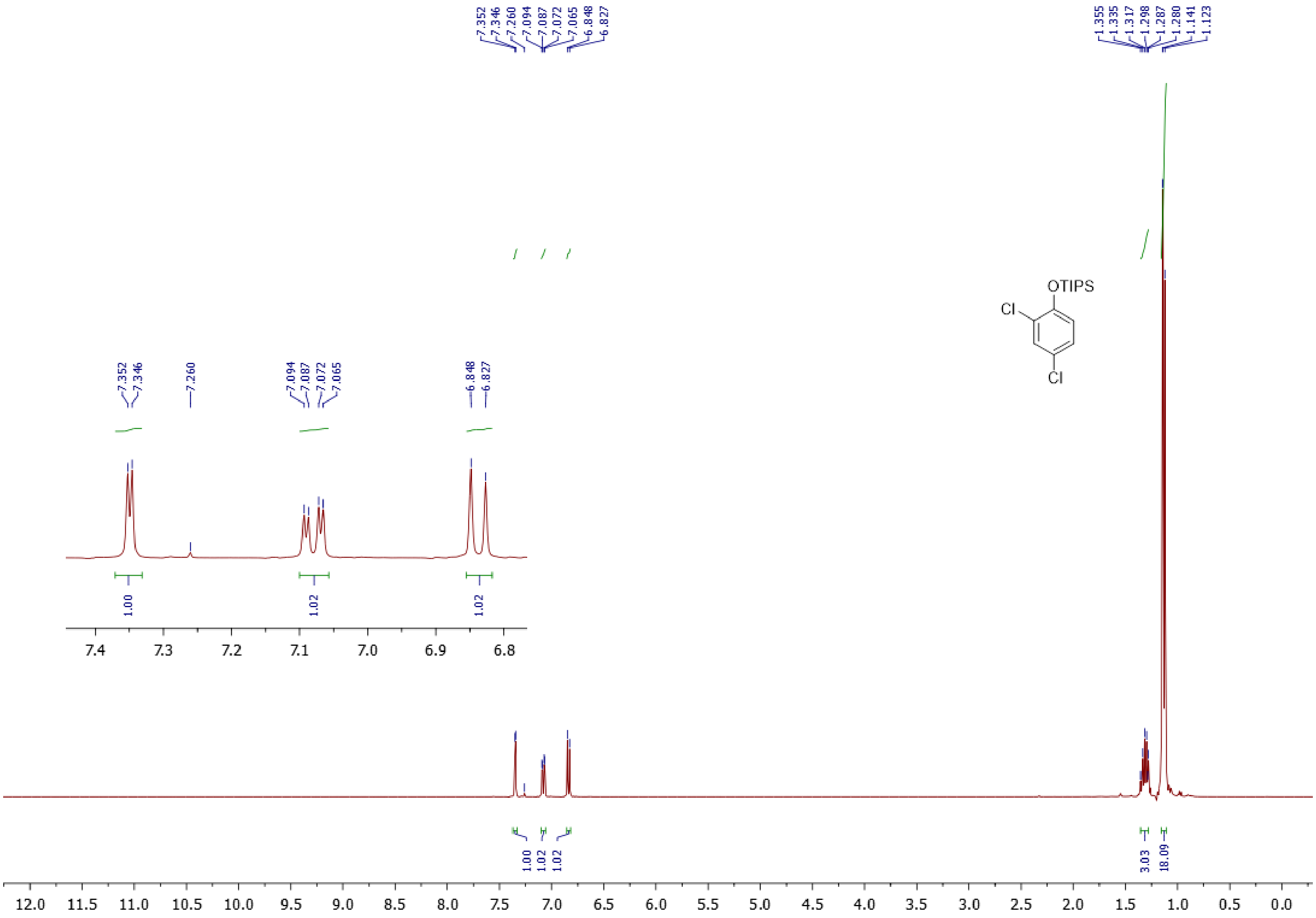

^{1}H-NMR spectra of **4z** (25 ^{o}C, 400 MHz, $CDCl_3$)

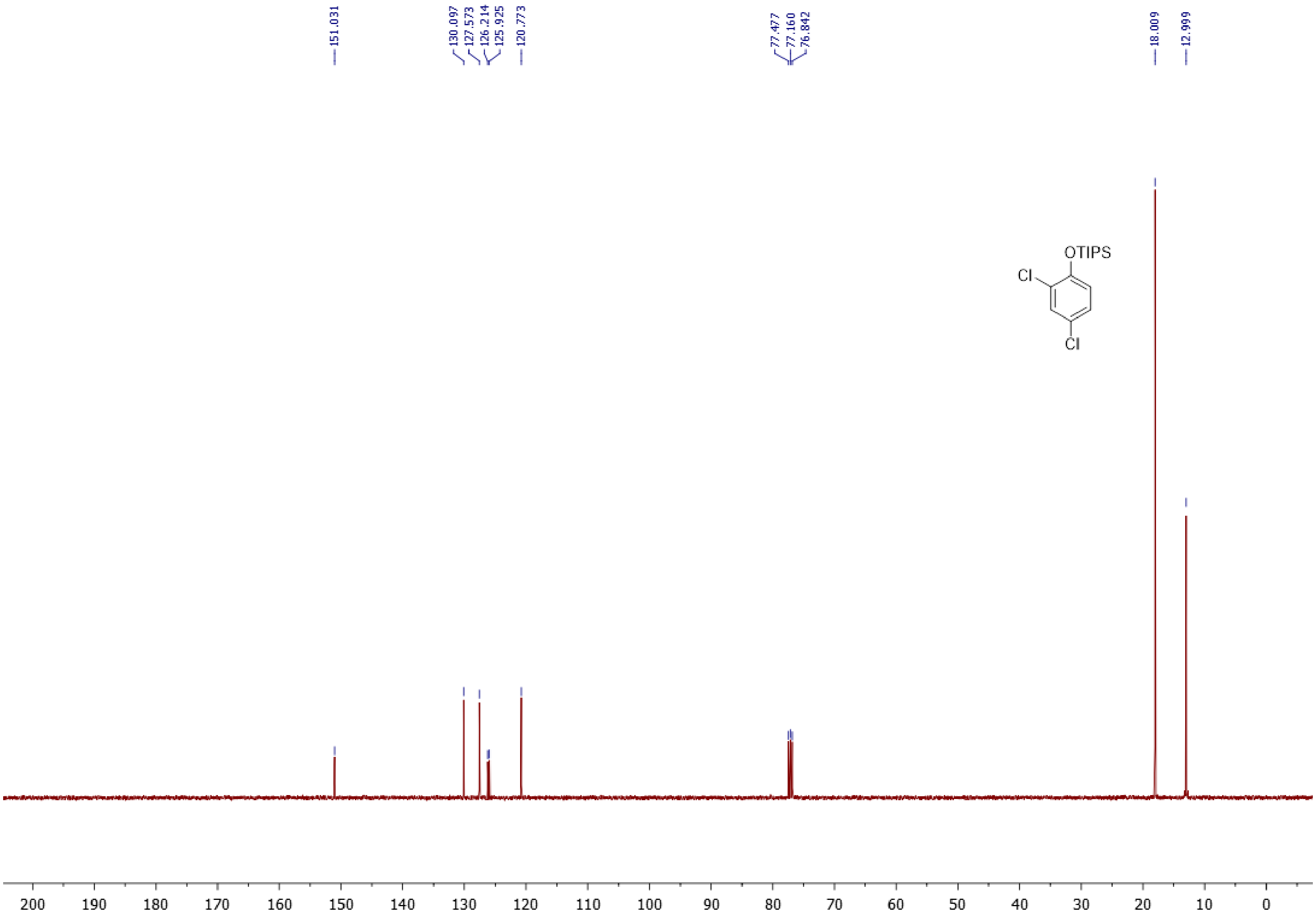

^{13}C-NMR spectra of **4z** (25 °C, 100 MHz, $CDCl_3$)

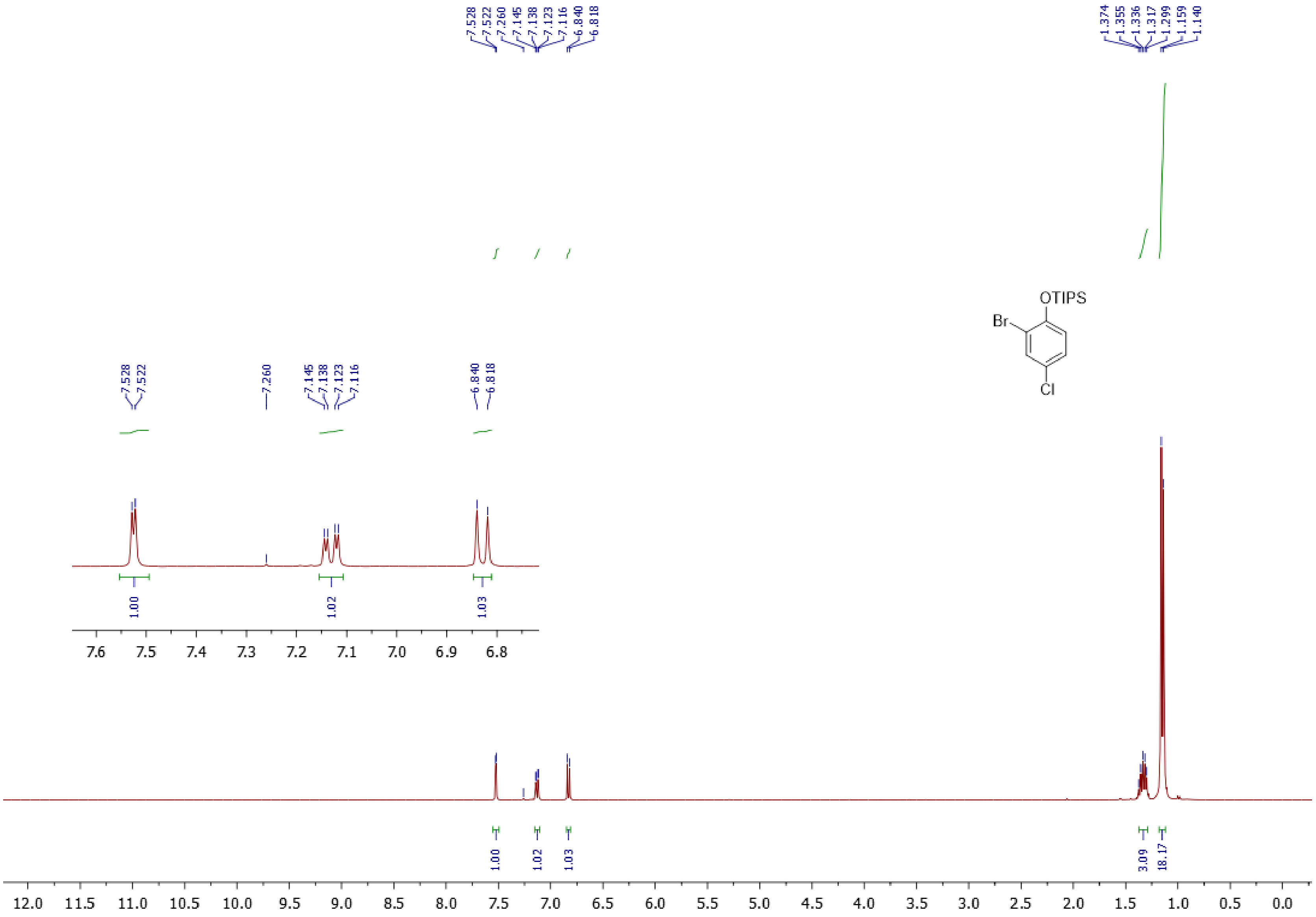

^{1}H-NMR spectra of **4aa** (25 ^{o}C, 400 MHz, $CDCl_3$)

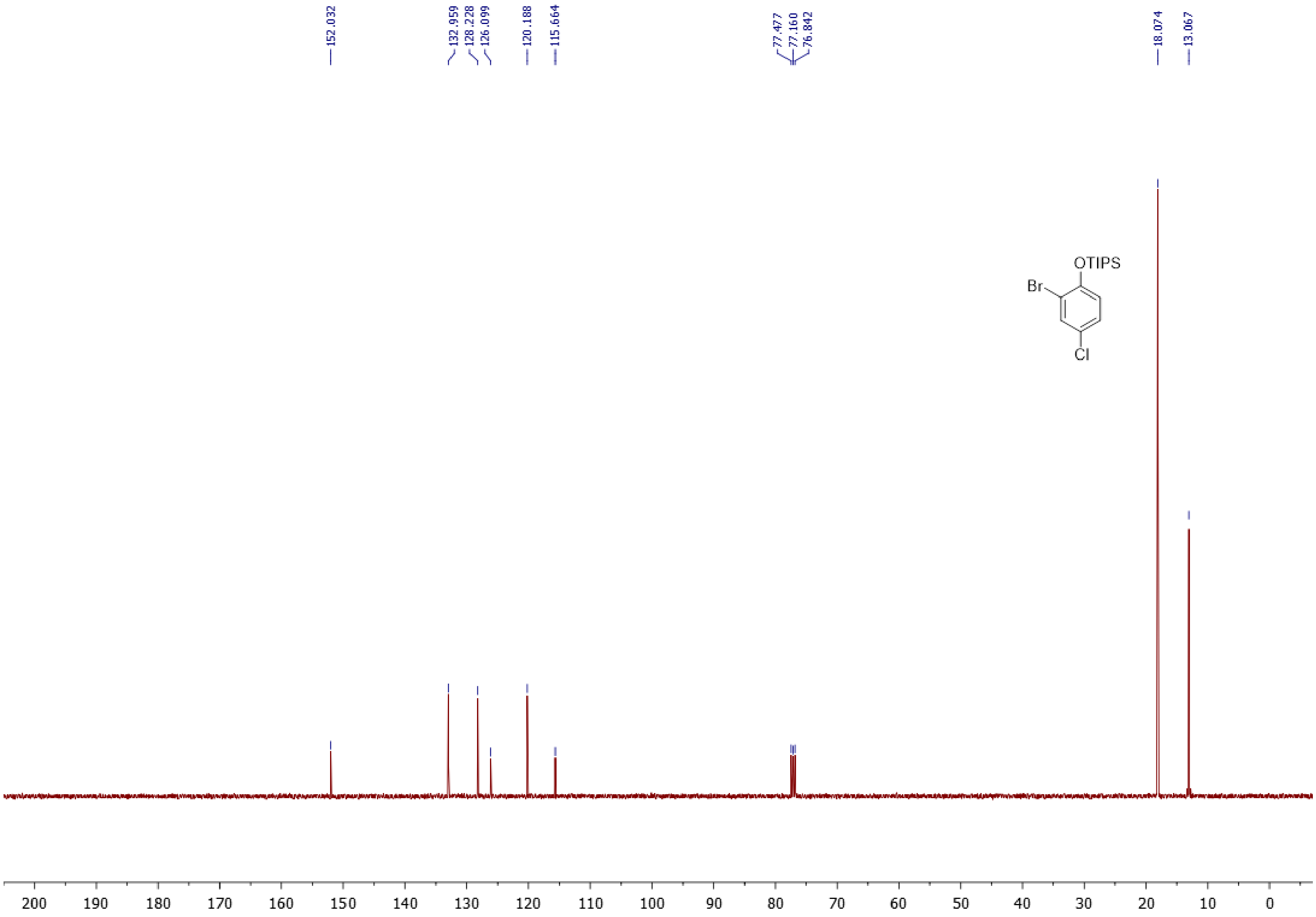

^{13}C-NMR spectra of **4aa** (25 ^{o}C, 100 MHz, $CDCl_3$)

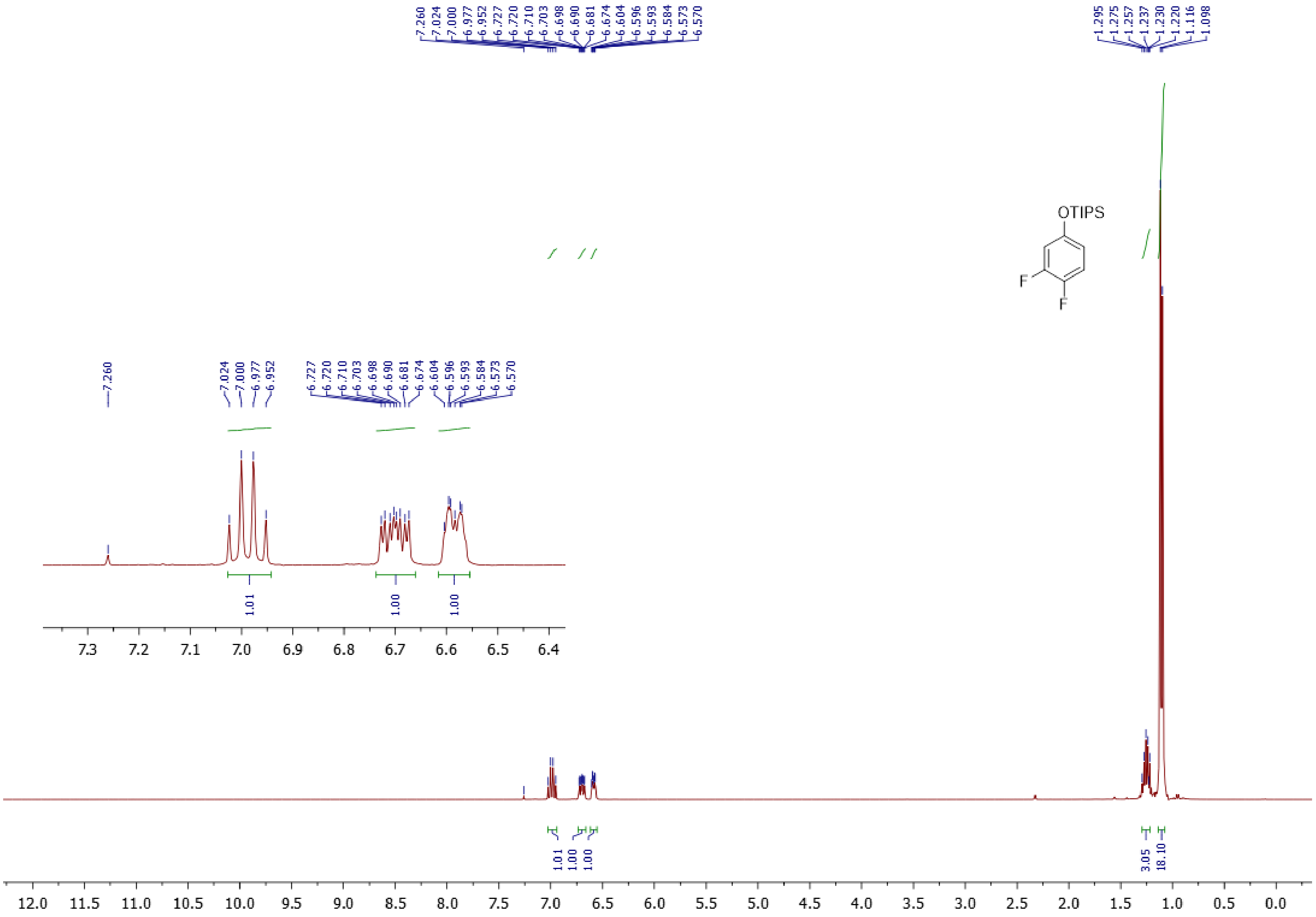

^{1}H-NMR spectra of **4ab** (25 °C, 400 MHz, $CDCl_3$)

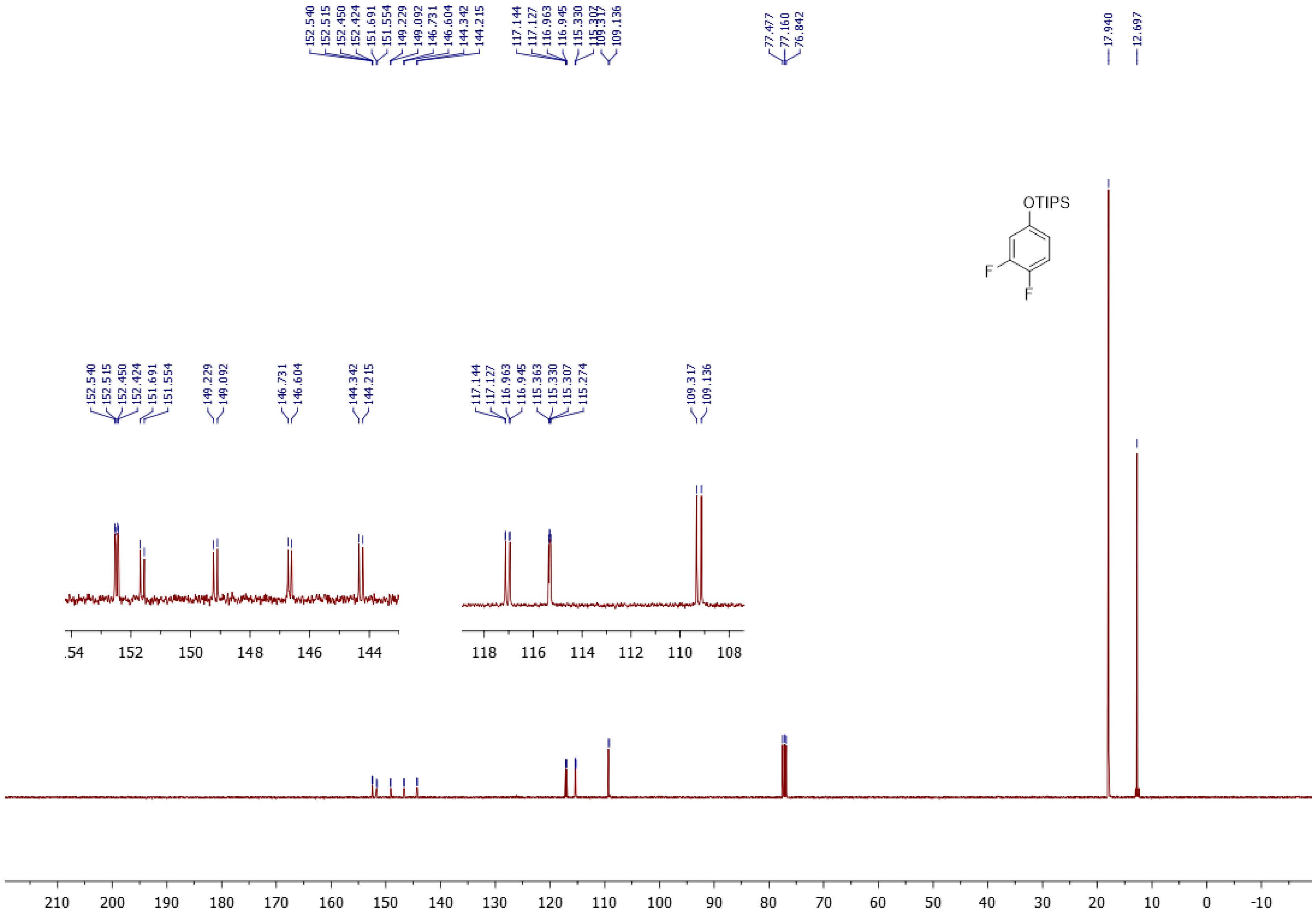

^{13}C-NMR spectra of **4ab** (25 ^{o}C, 100 MHz, $CDCl_3$)

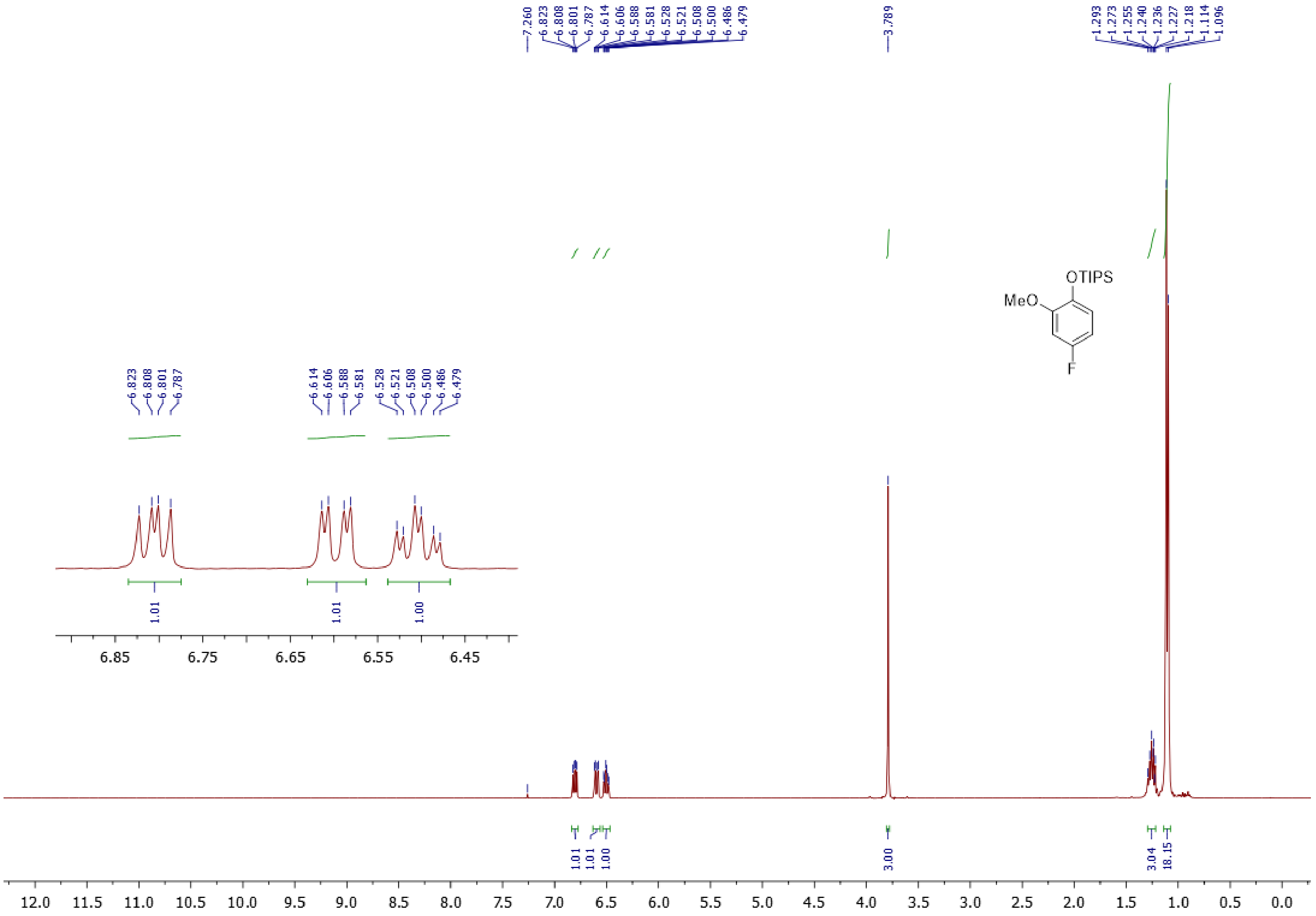

^{1}H-NMR spectra of **4ac** (25 °C, 400 MHz, $CDCl_3$)

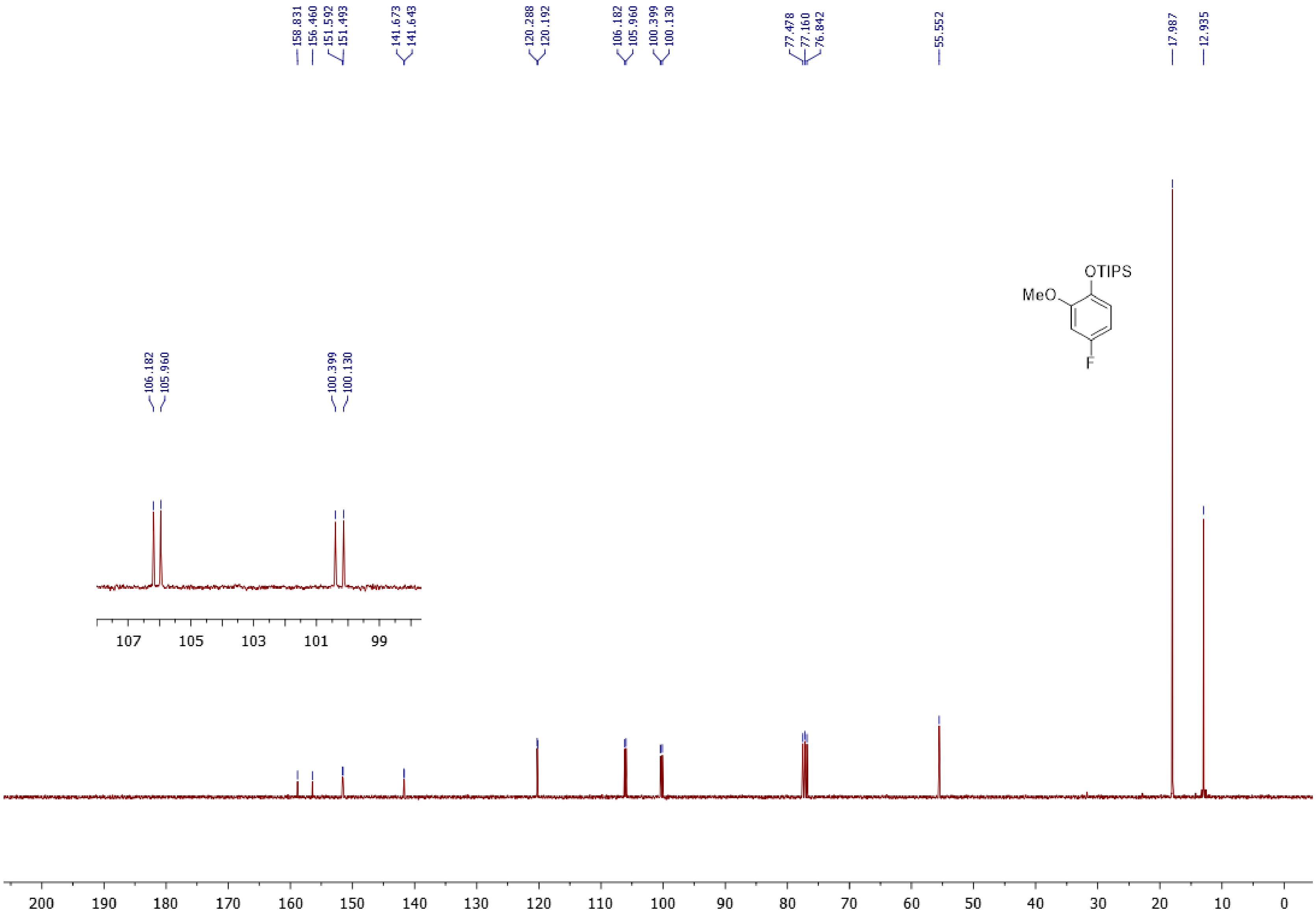

^{13}C-NMR spectra of **4ac** (25 °C, 100 MHz, $CDCl_3$)

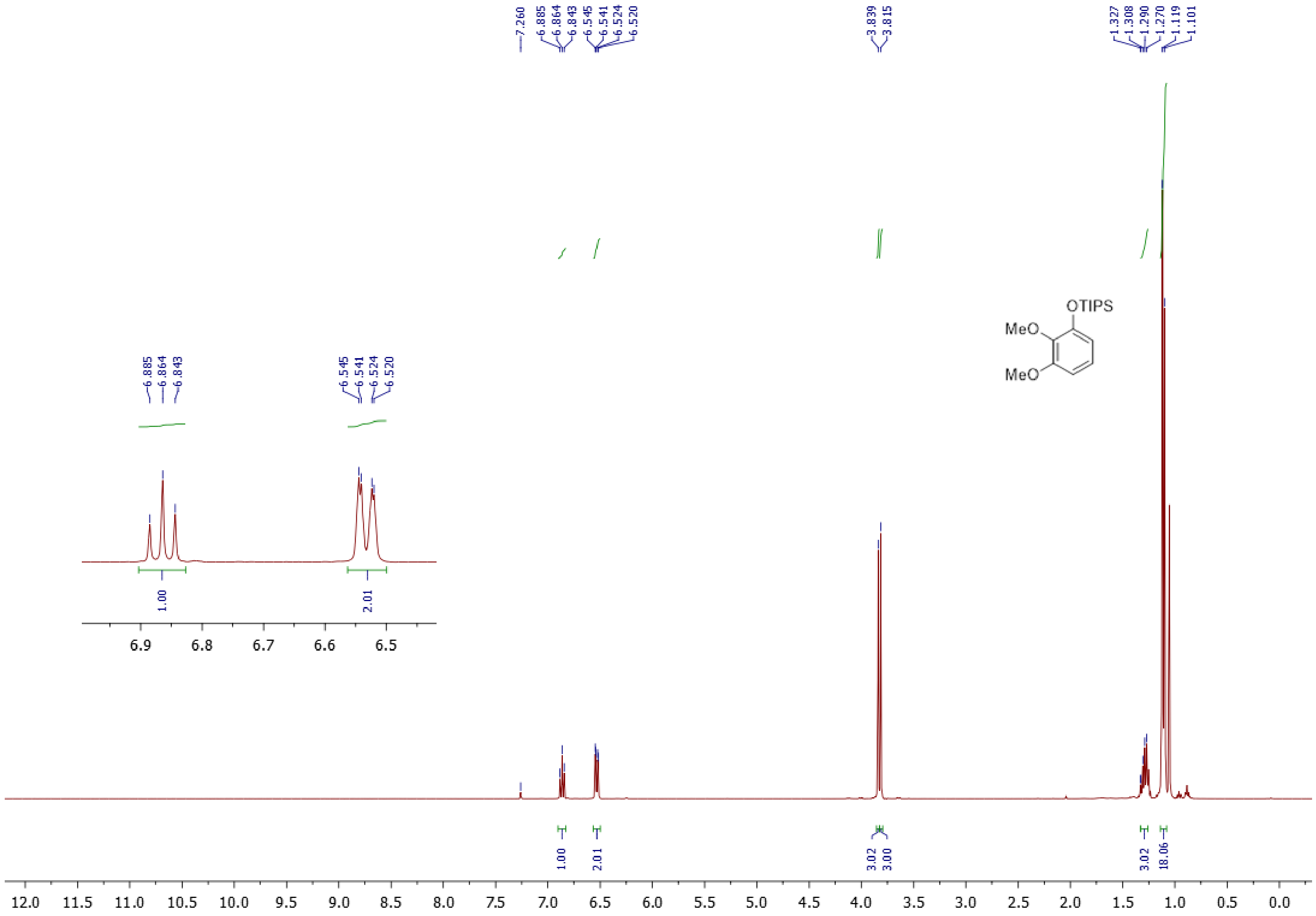

^{1}H-NMR spectra of **4ad** (25 °C, 400 MHz, $CDCl_3$)

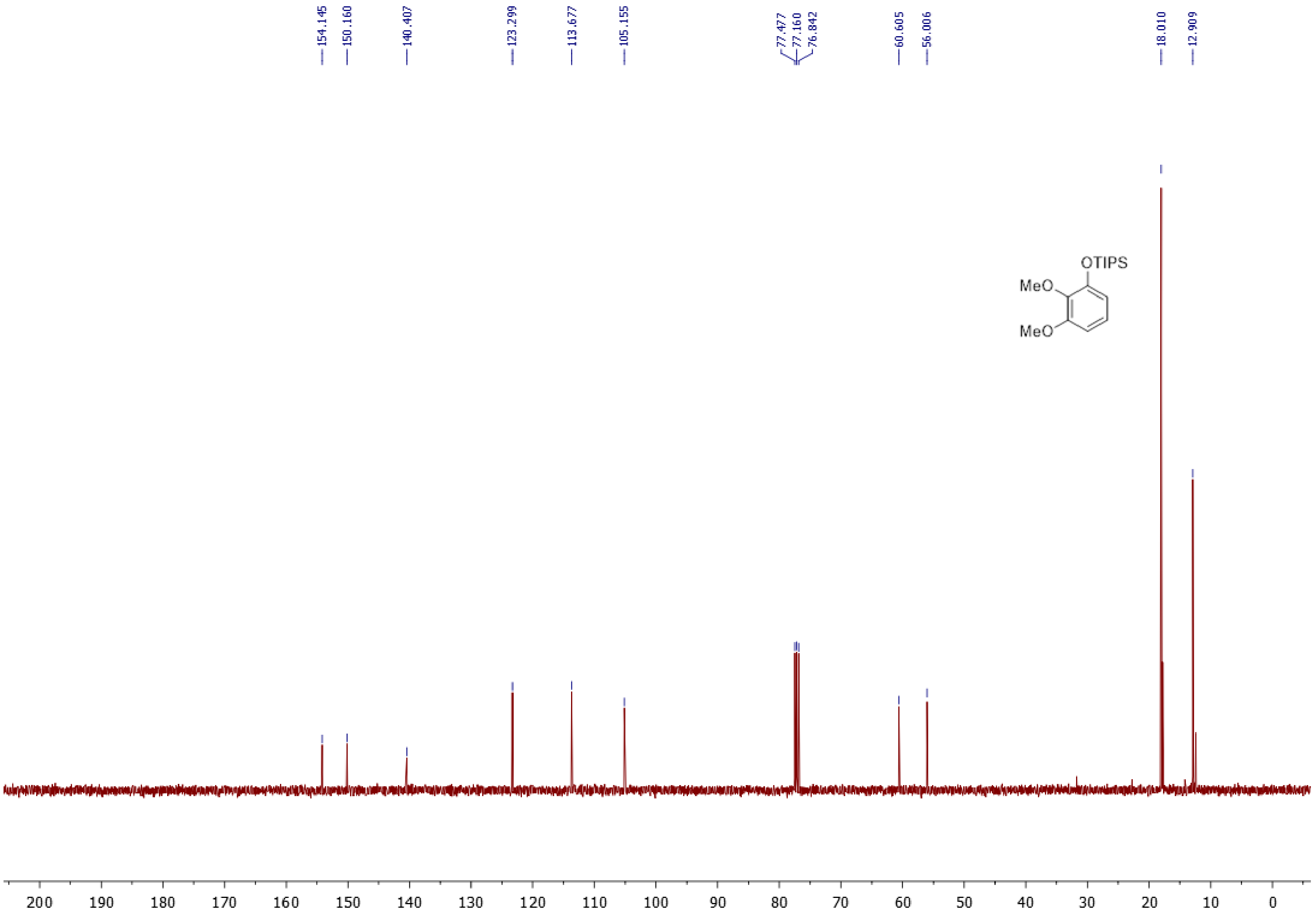

^{13}C-NMR spectra of **4ad** (25 ^{o}C, 100 MHz, $CDCl_3$)

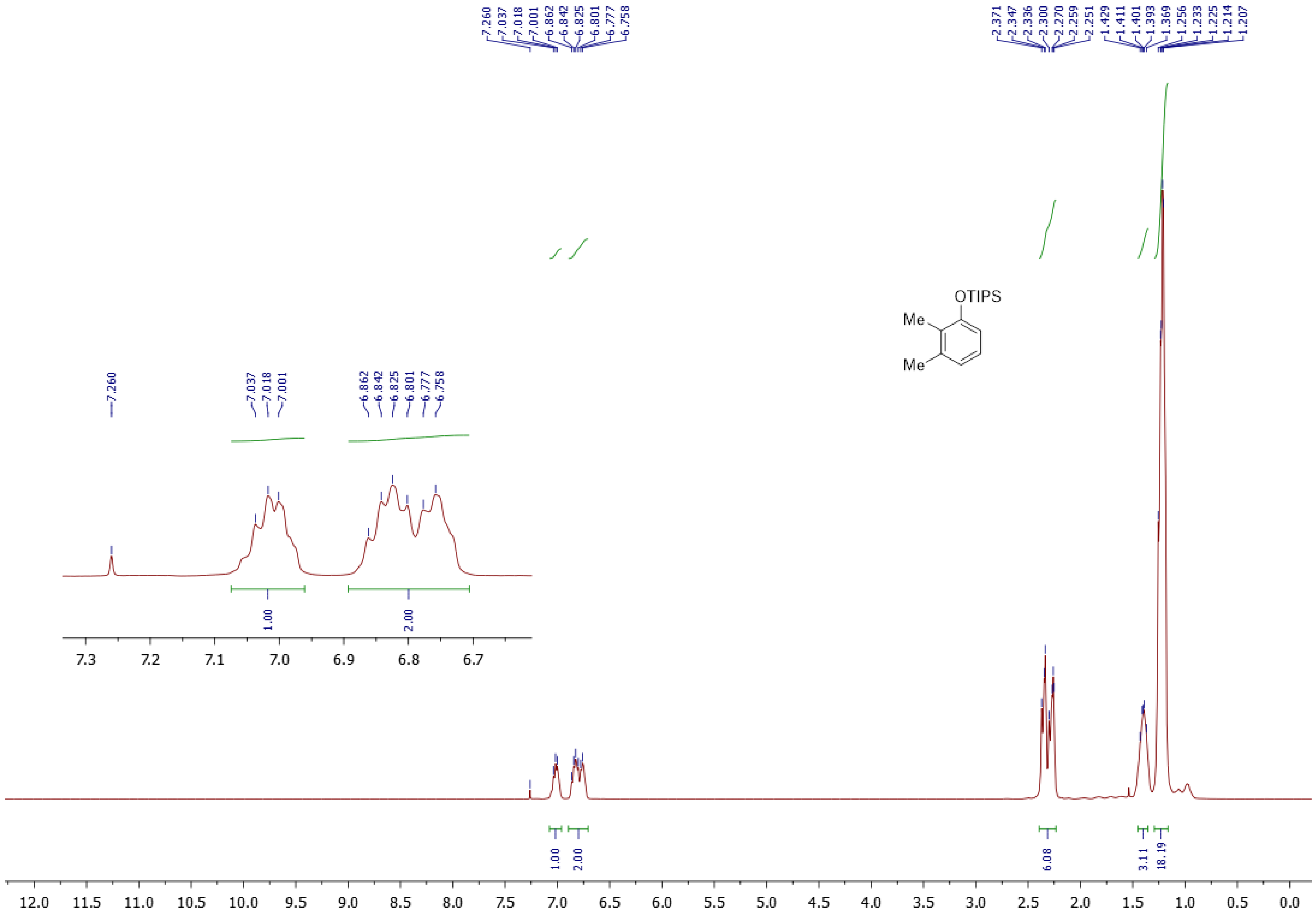

^{1}H-NMR spectra of **4ae** (25 °C, 400 MHz, $CDCl_3$)

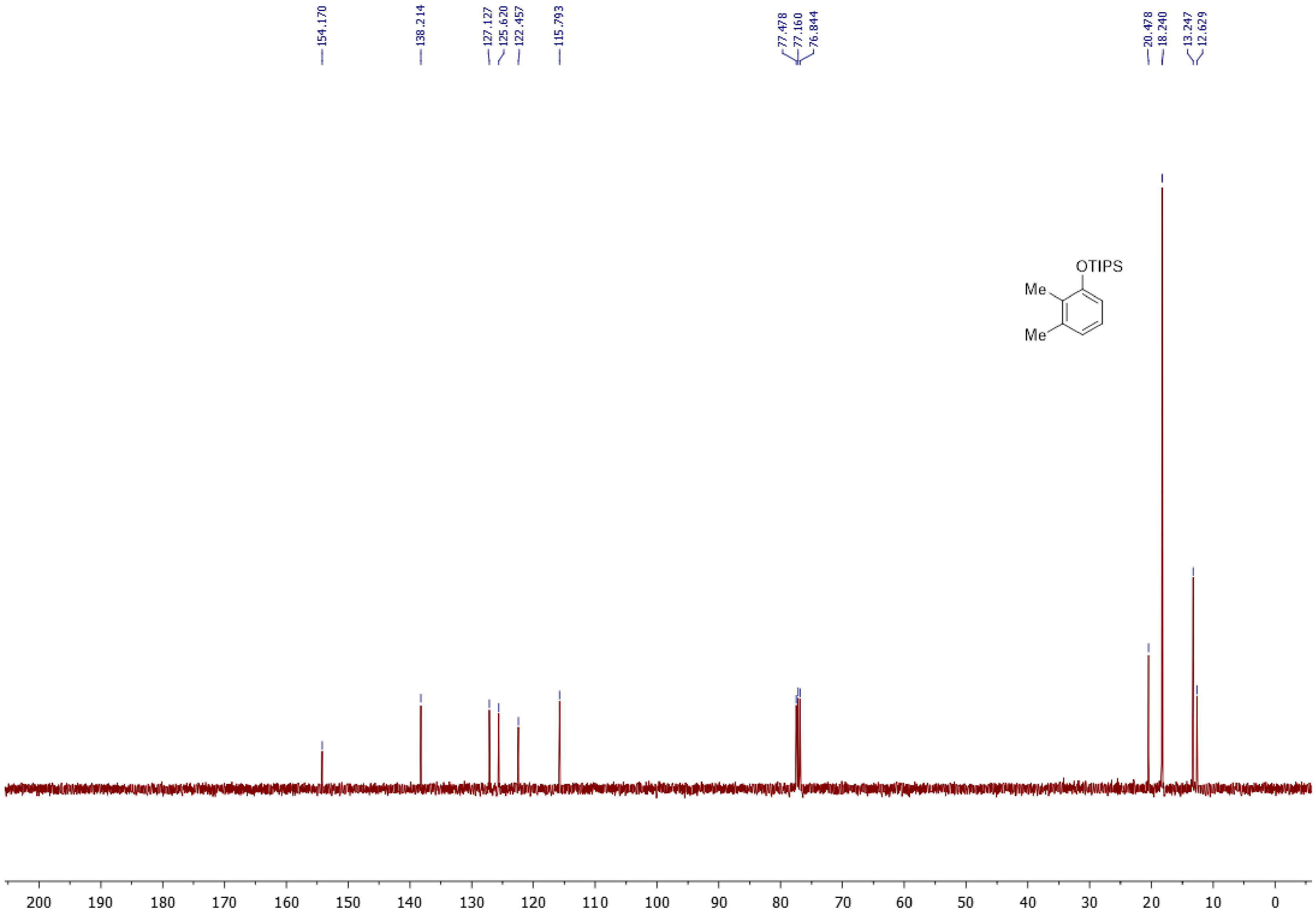

^{13}C-NMR spectra of **4ae** (25 ^{o}C, 100 MHz, $CDCl_3$)

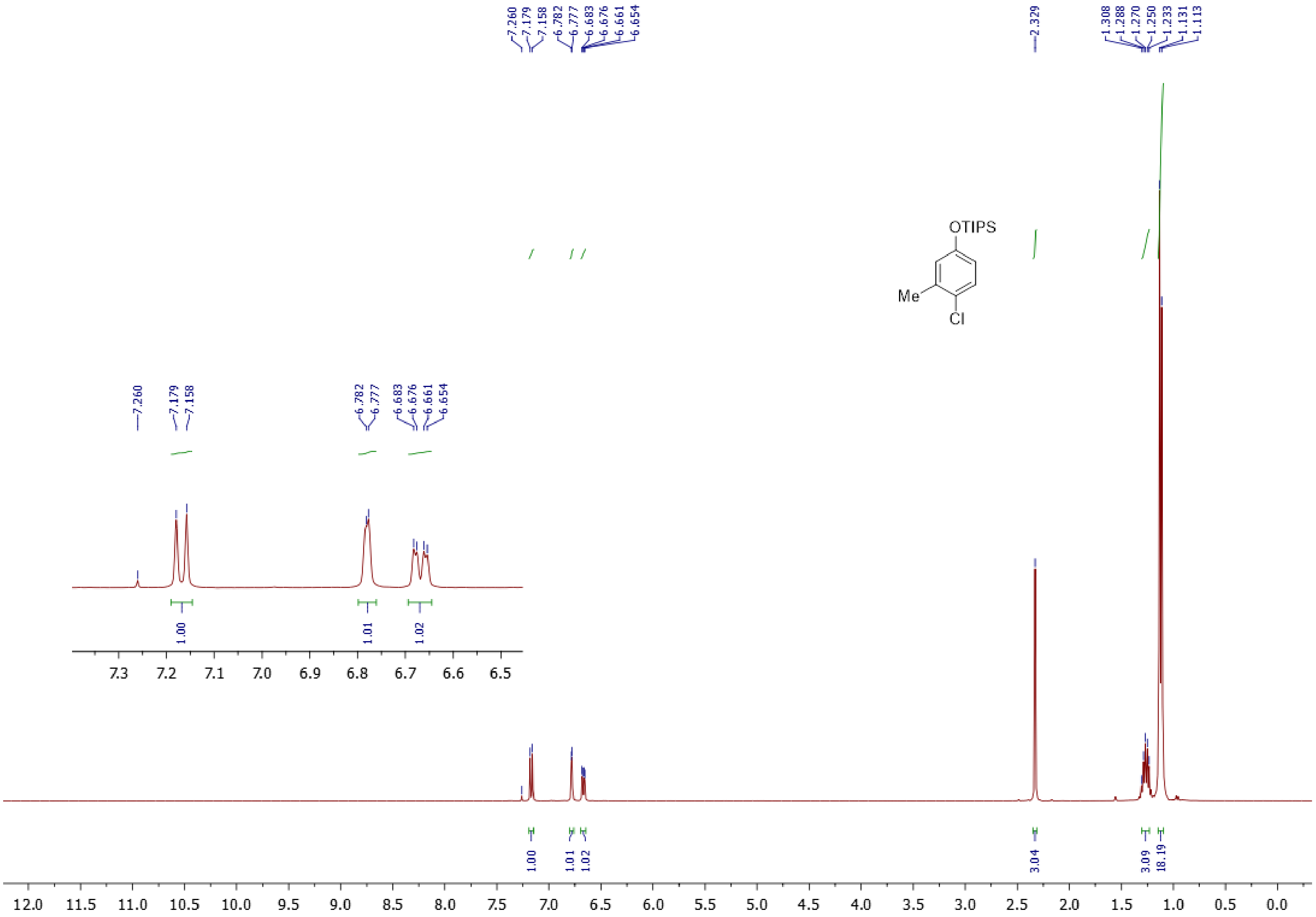

^{1}H-NMR spectra of **4af** (25 ºC, 400 MHz, $CDCl_3$)

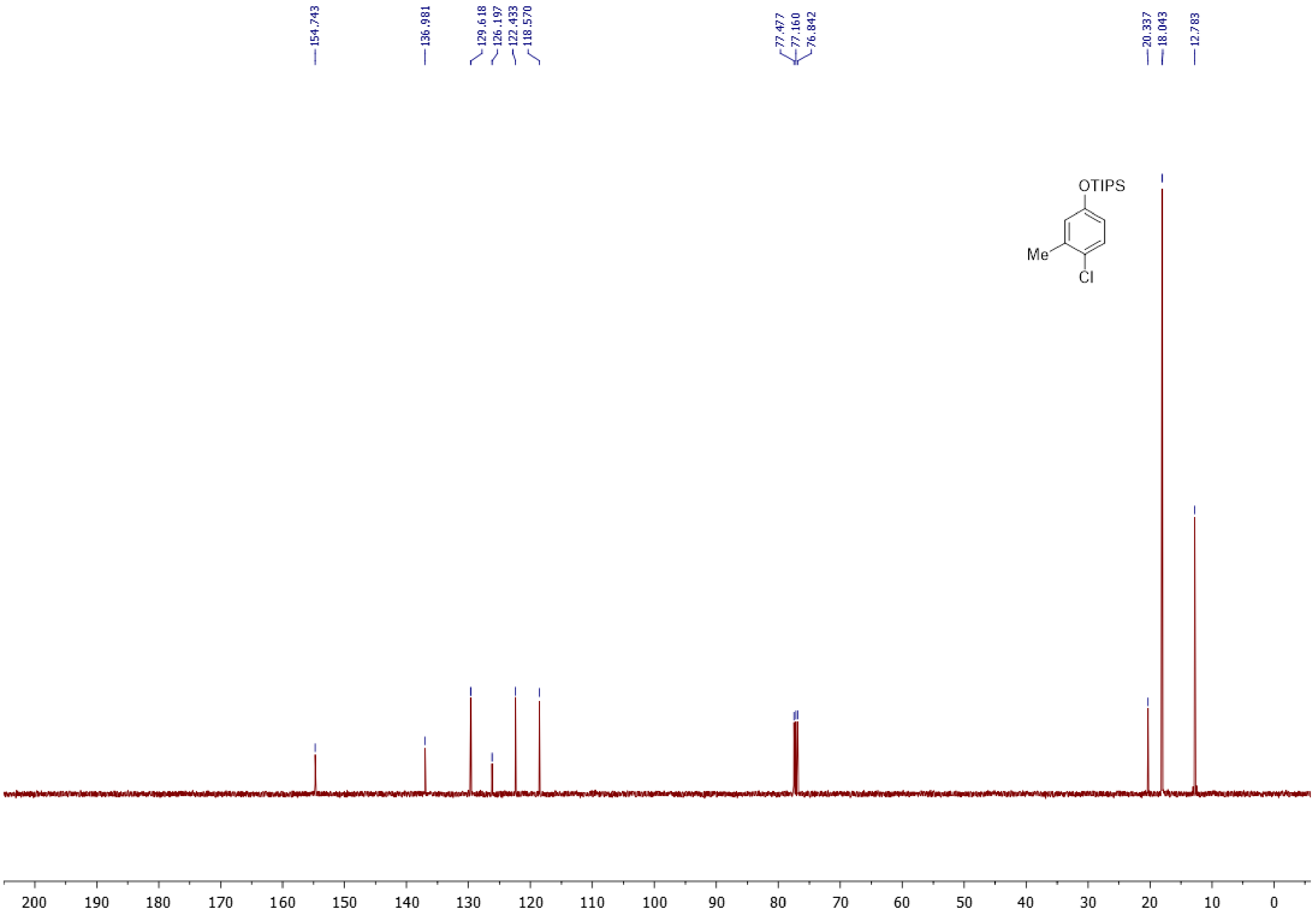

^{13}C-NMR spectra of **4af** (25 ^{o}C, 100 MHz, $CDCl_3$)

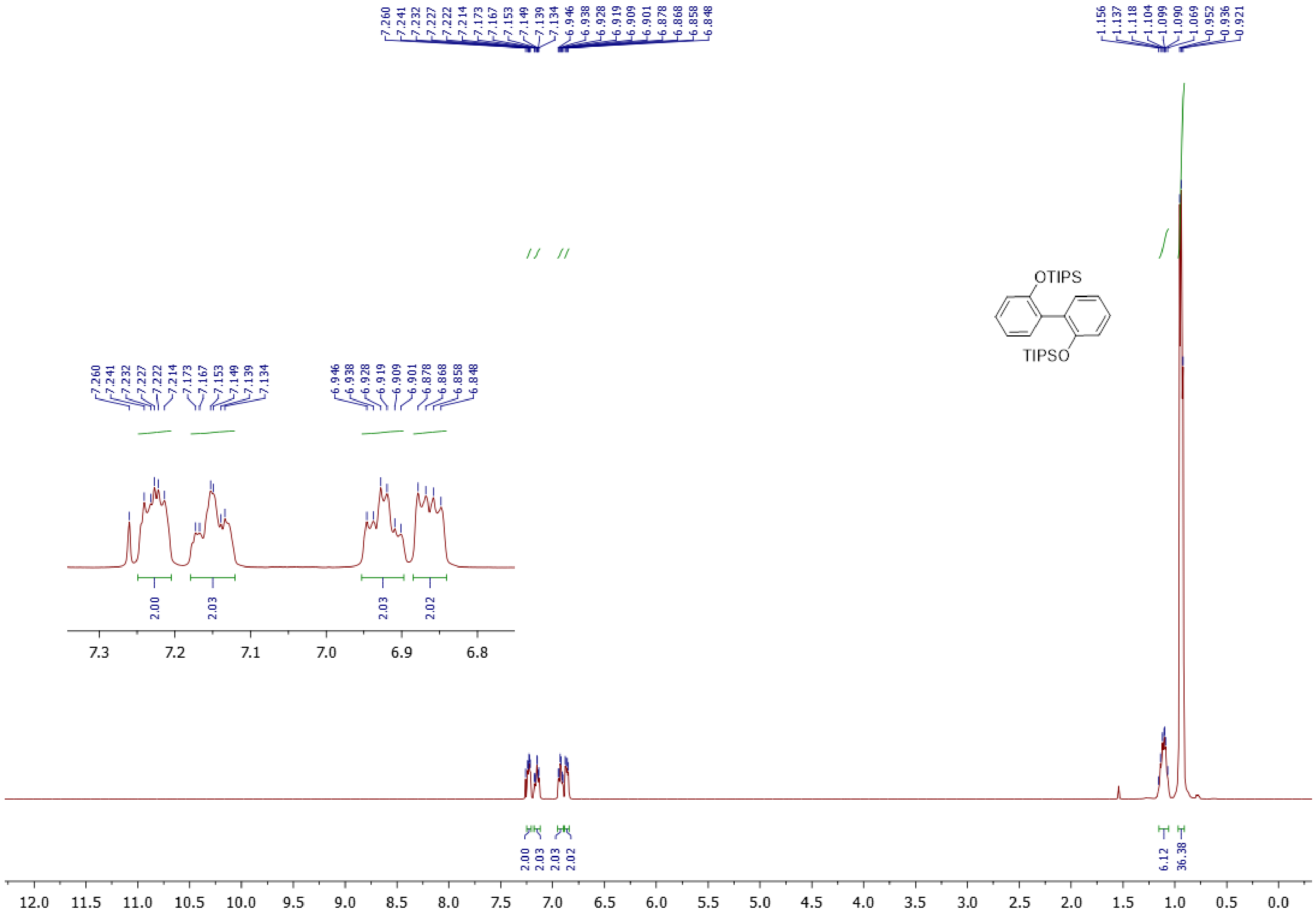

^{1}H-NMR spectra of **4ag** (25 ^{o}C, 400 MHz, $CDCl_3$)

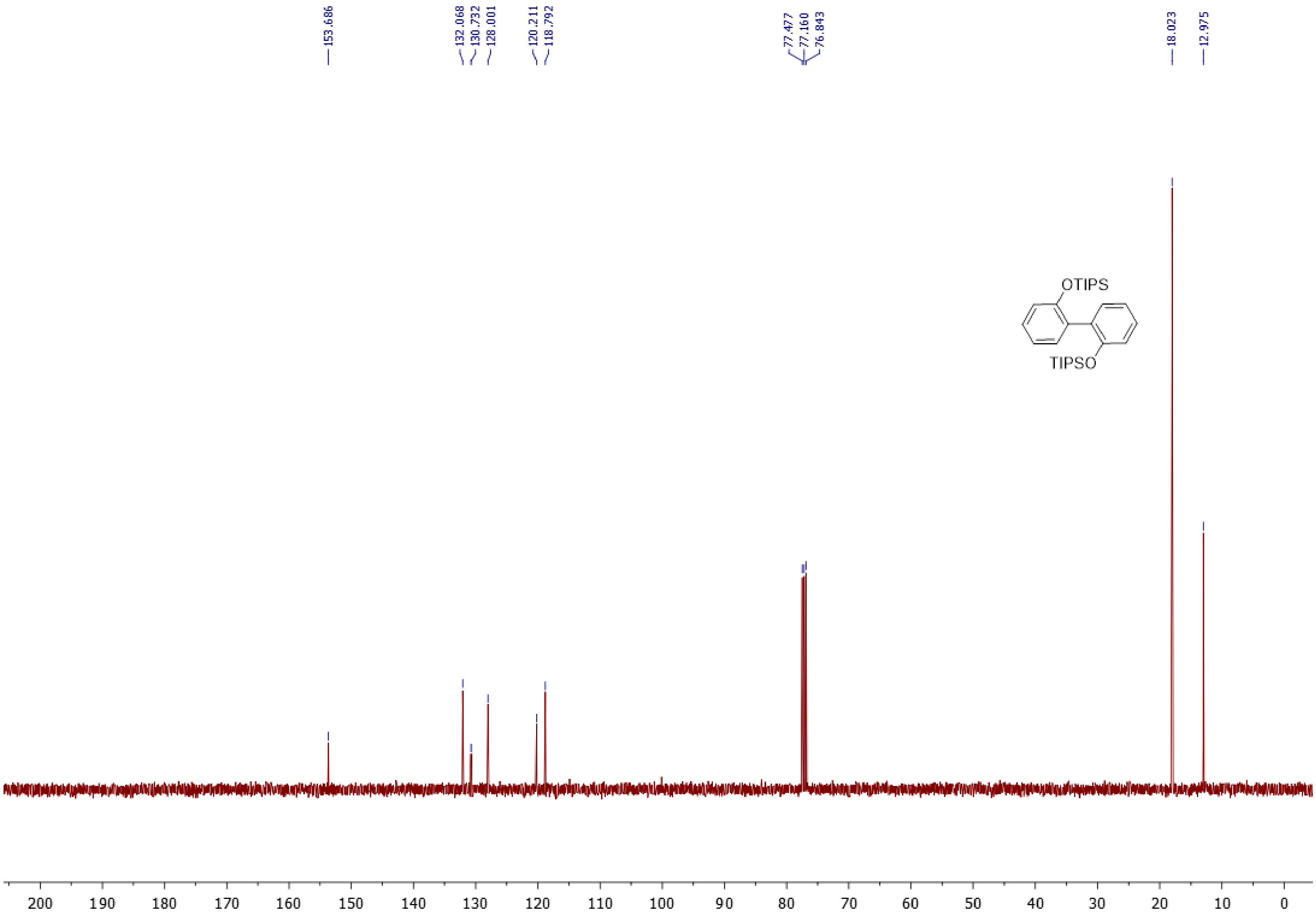

^{13}C-NMR spectra of **4ag** (25 °C, 100 MHz, $CDCl_3$)

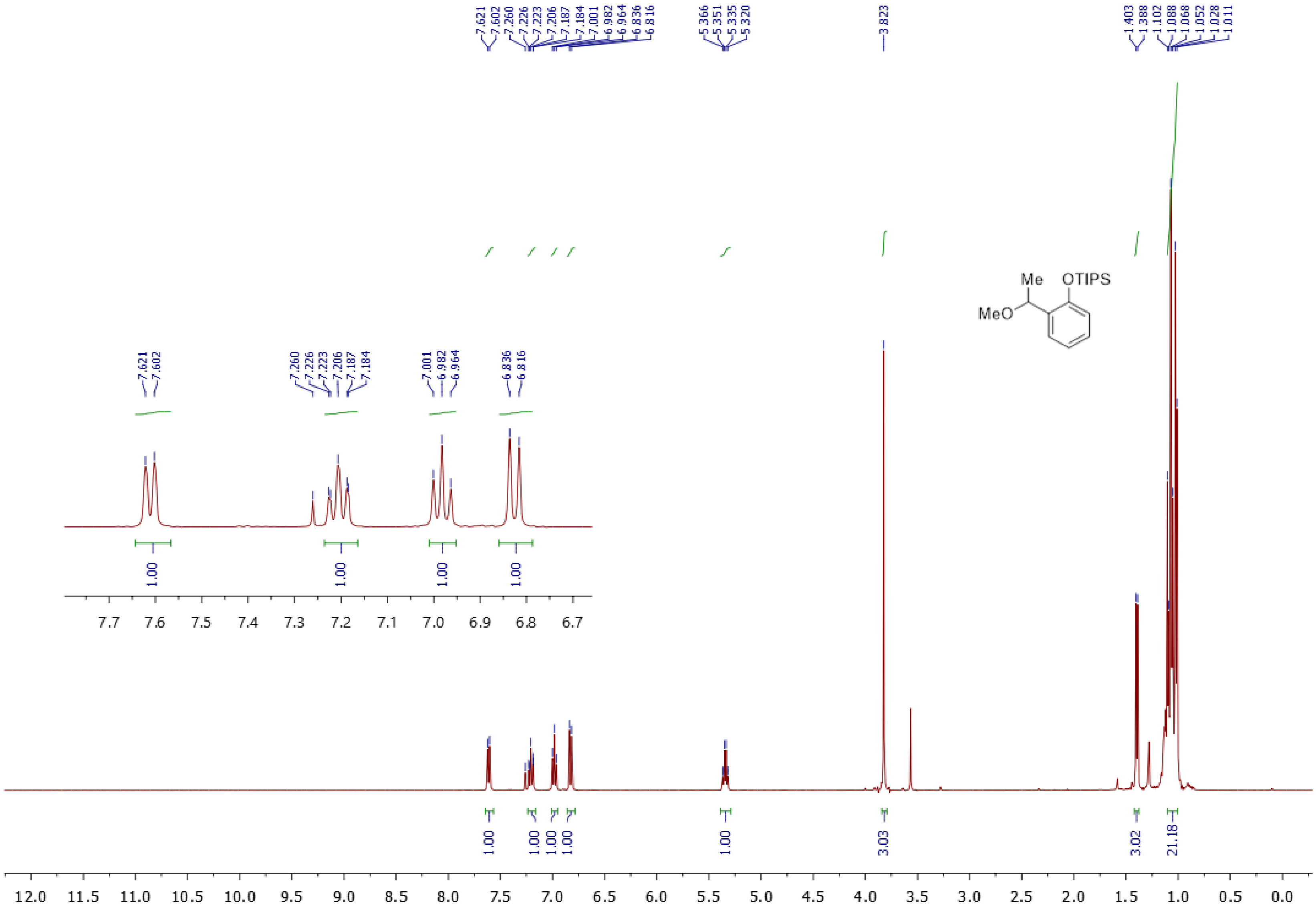

^{1}H-NMR spectra of **4ah** (25 °C, 400 MHz, $CDCl_3$)

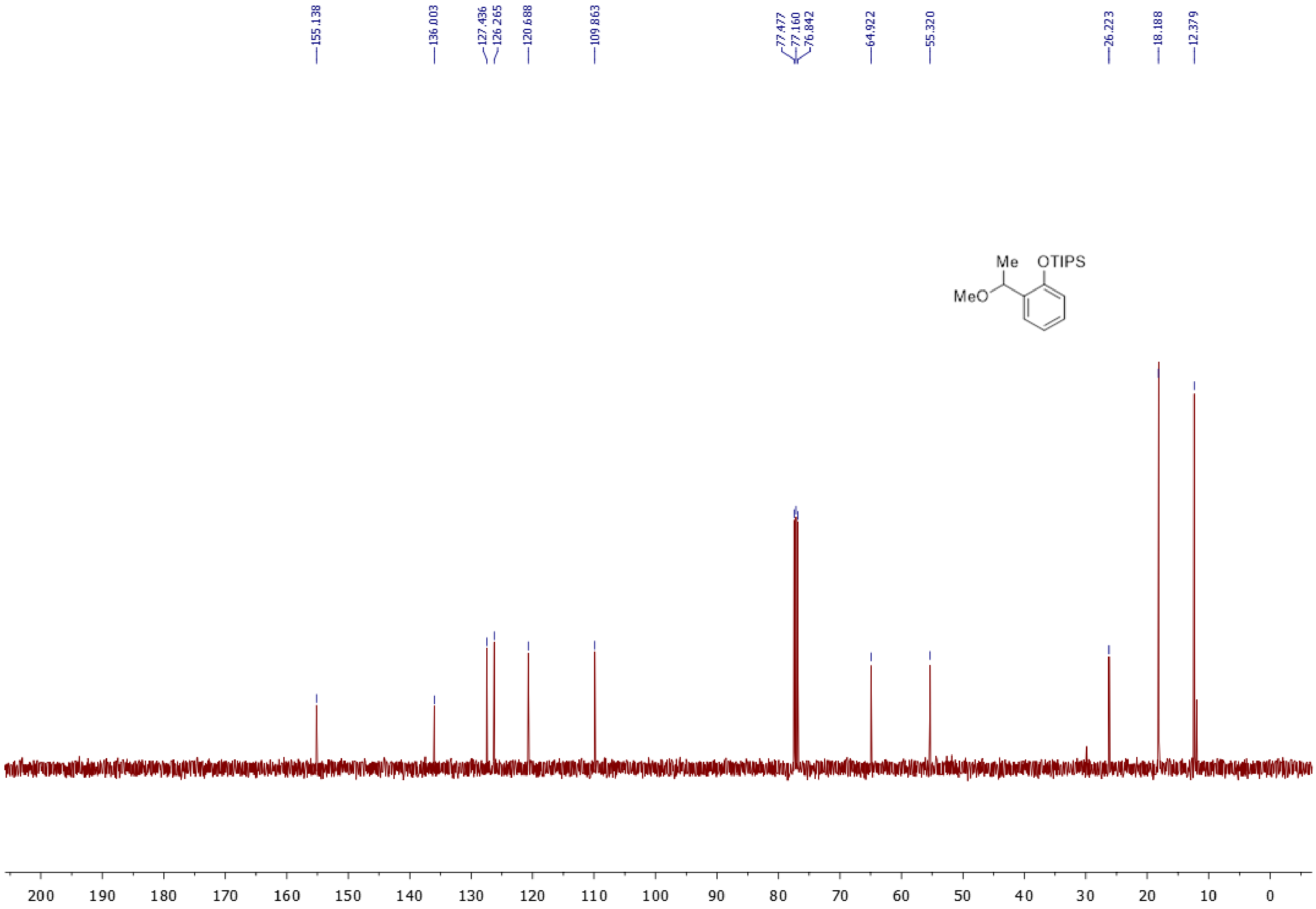

^{13}C-NMR spectra of **4ah** (25 ^{o}C, 100 MHz, $CDCl_3$)

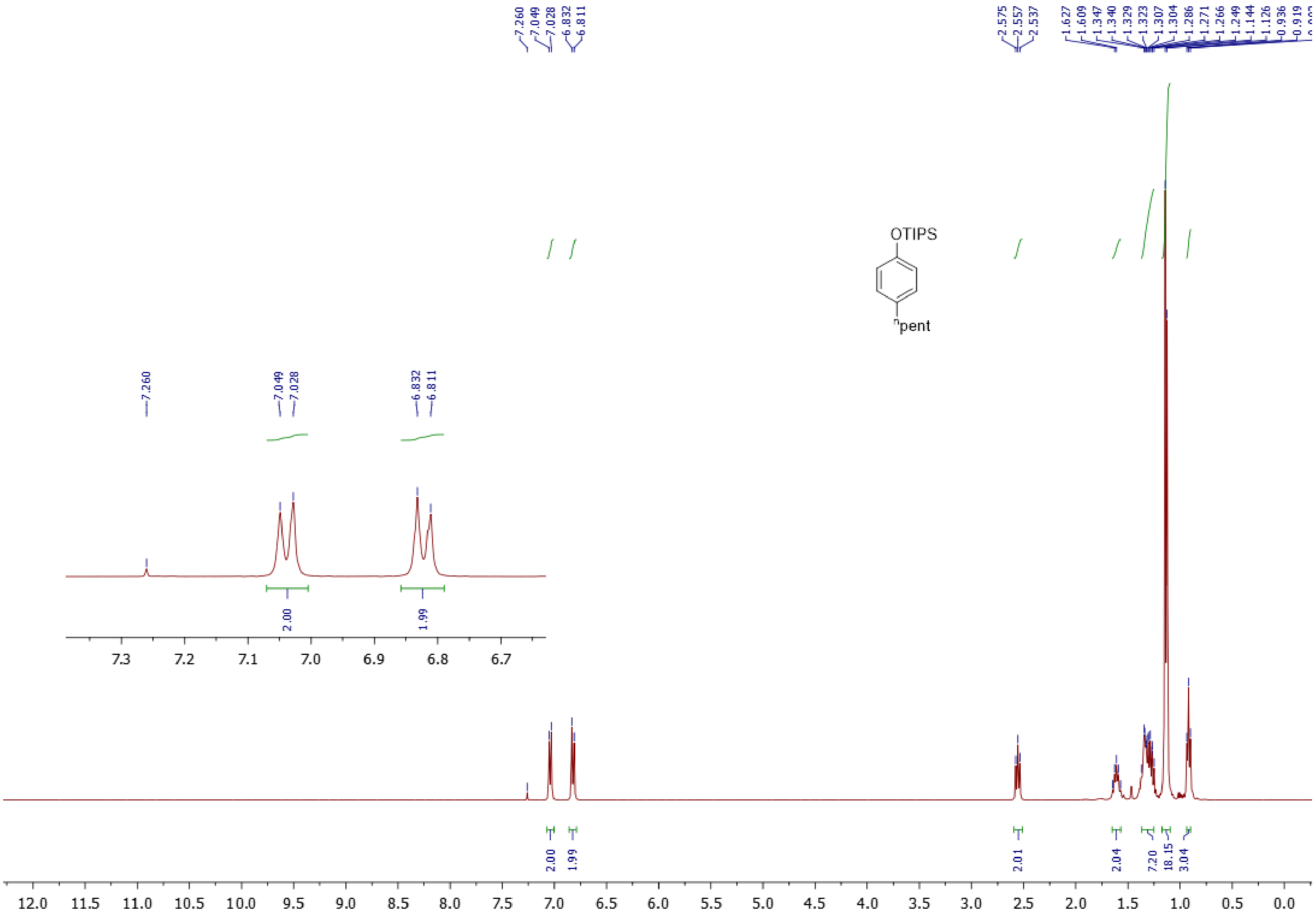

^{1}H-NMR spectra of **6c** (25 ^{o}C, 400 MHz, $CDCl_3$)

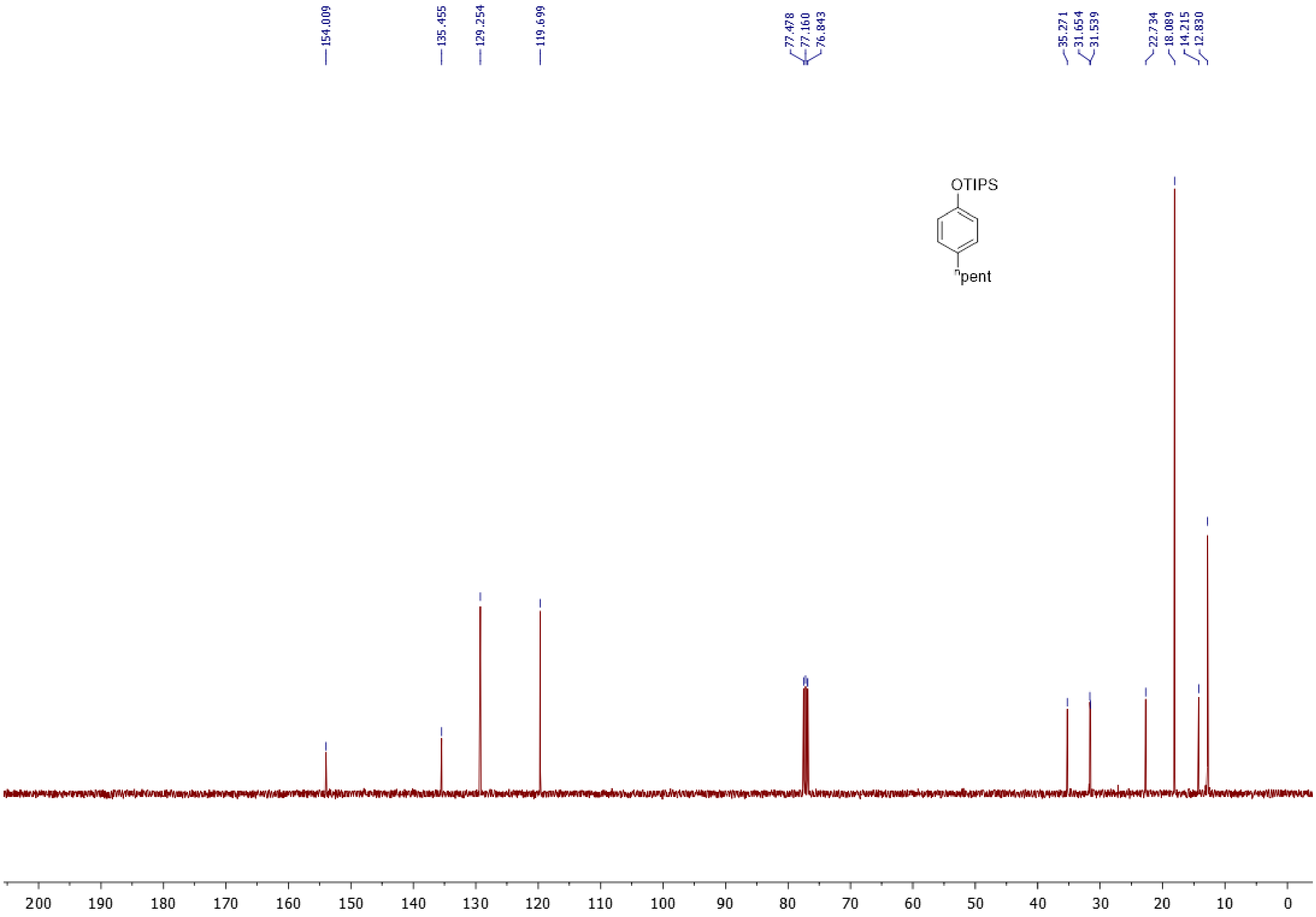

^{13}C-NMR spectra of **6c** (25 °C, 100 MHz, $CDCl_3$)

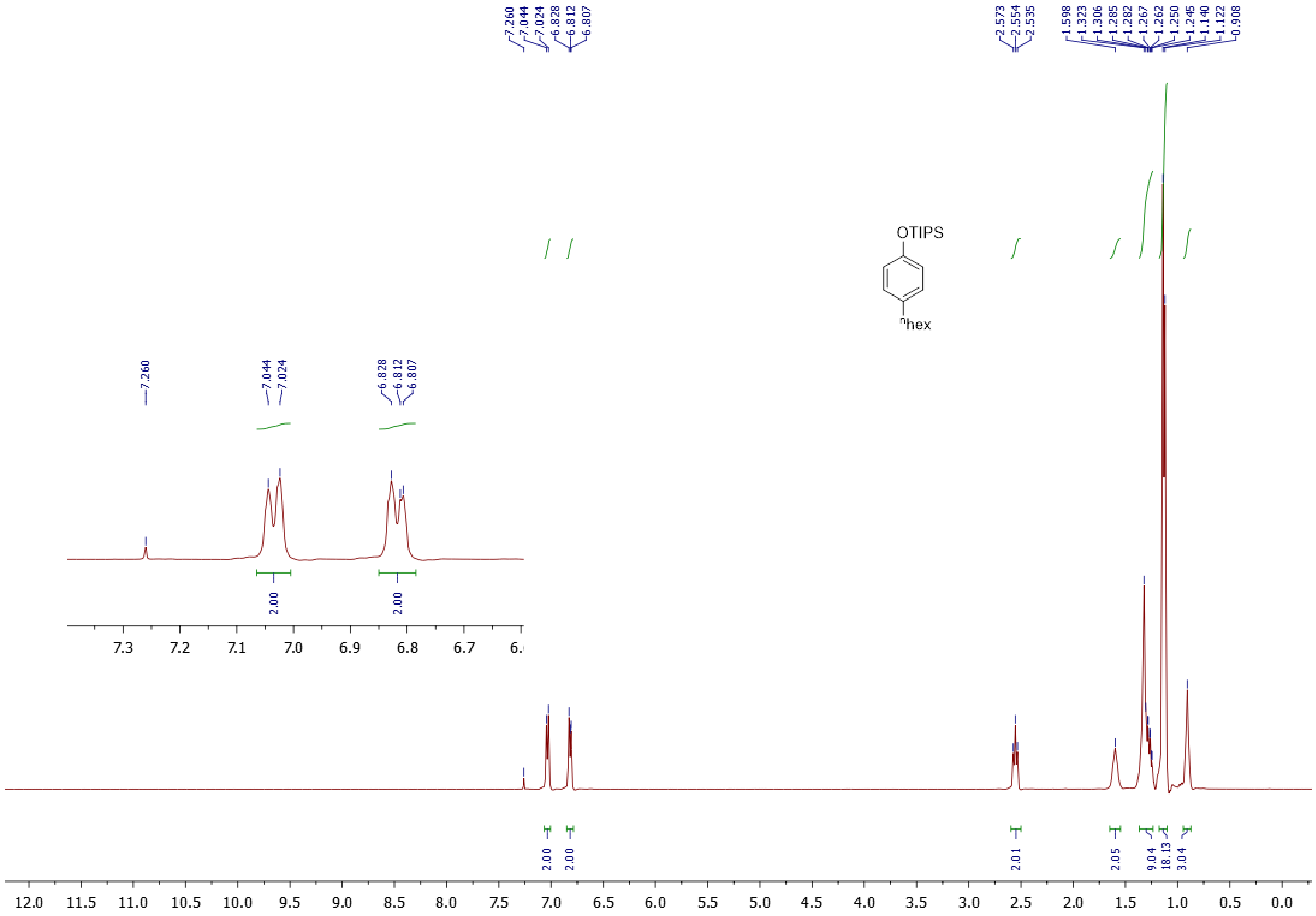

^{1}H-NMR spectra of **6d** (25 °C, 400 MHz, $CDCl_3$)

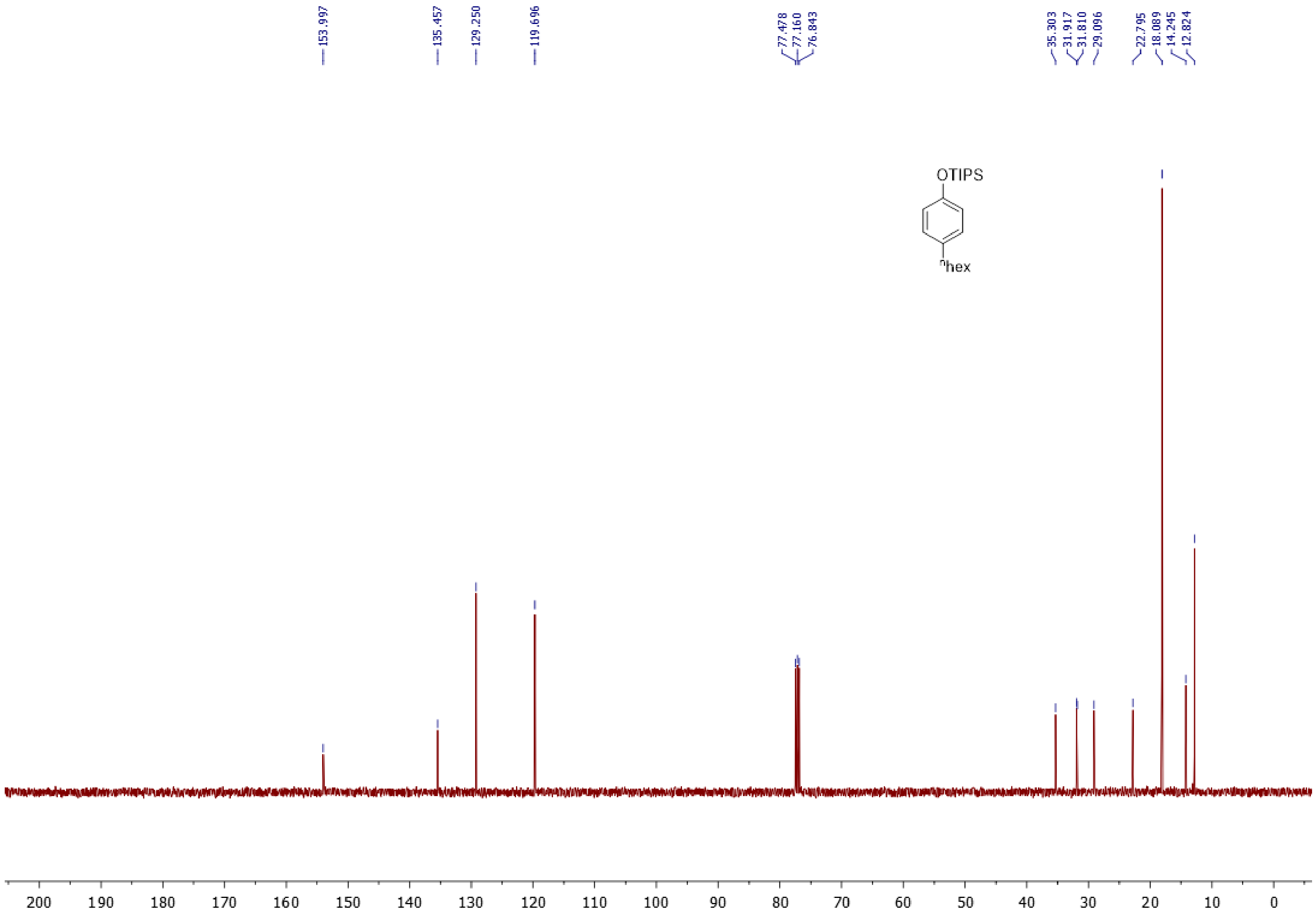

^{13}C-NMR spectra of **6d** (25 ^{o}C, 100 MHz, $CDCl_3$)

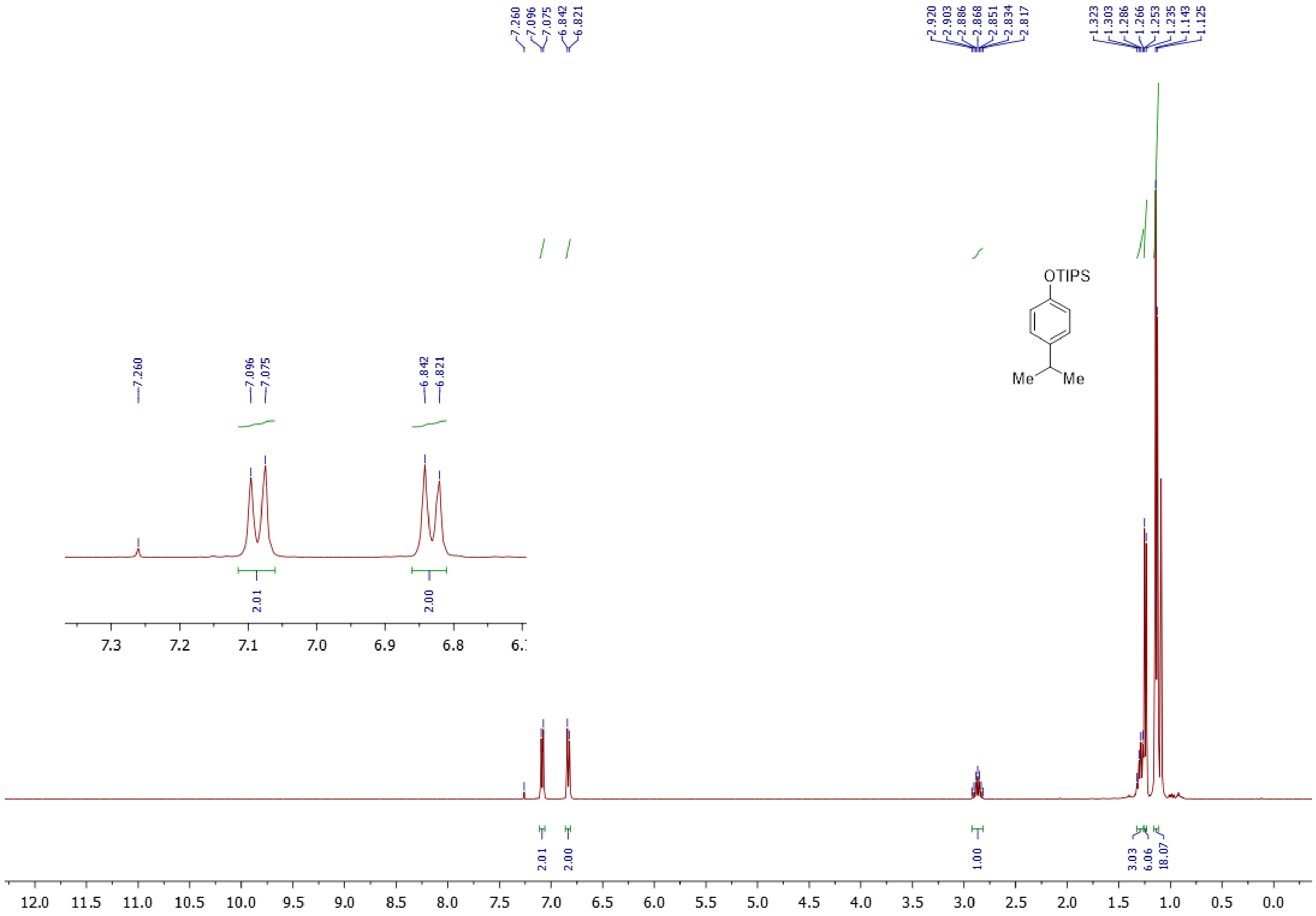

^{1}H-NMR spectra of **6e** (25 ^{o}C, 400 MHz, $CDCl_3$)

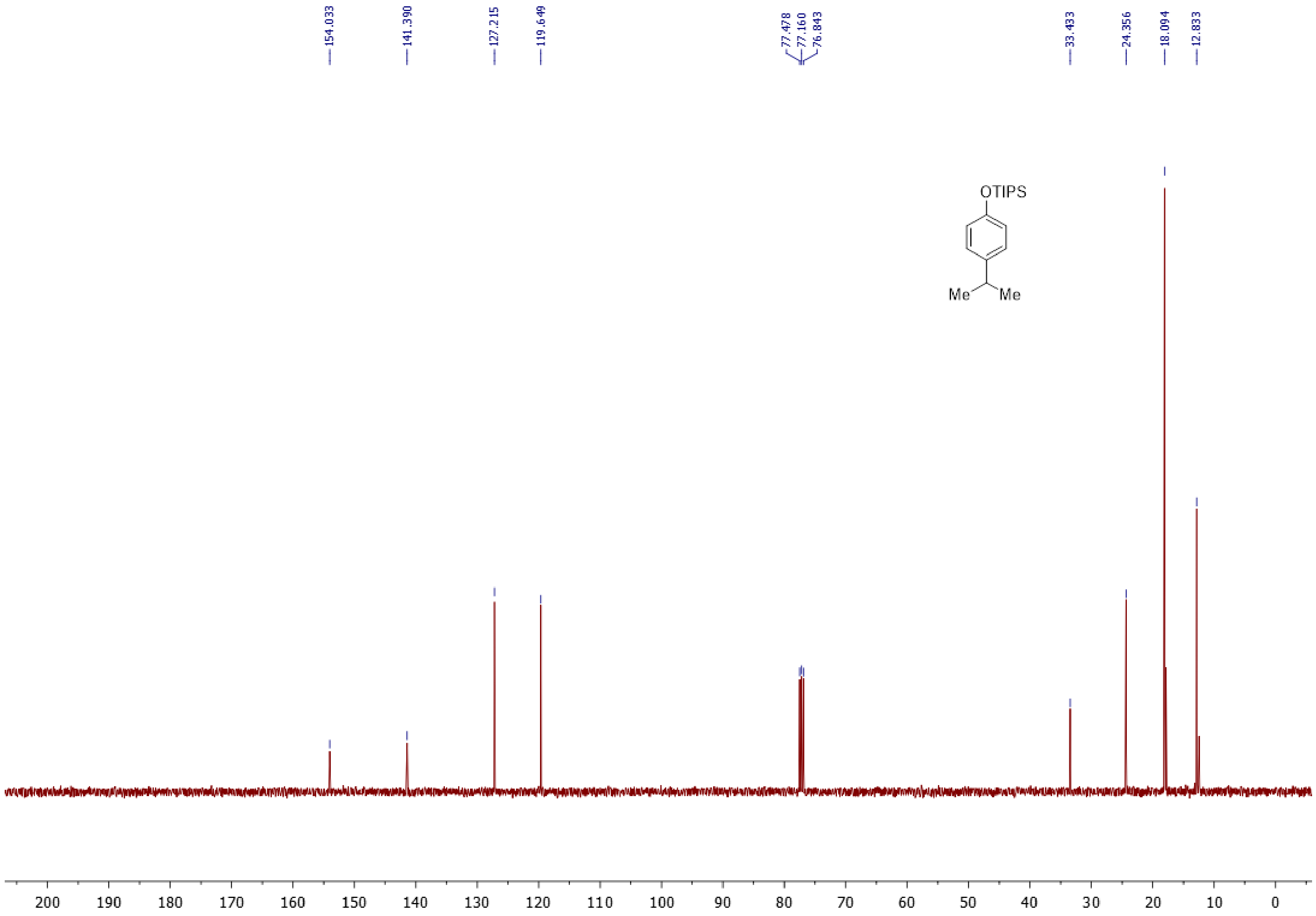

^{13}C-NMR spectra of **6e** (25 °C, 100 MHz, $CDCl_3$)

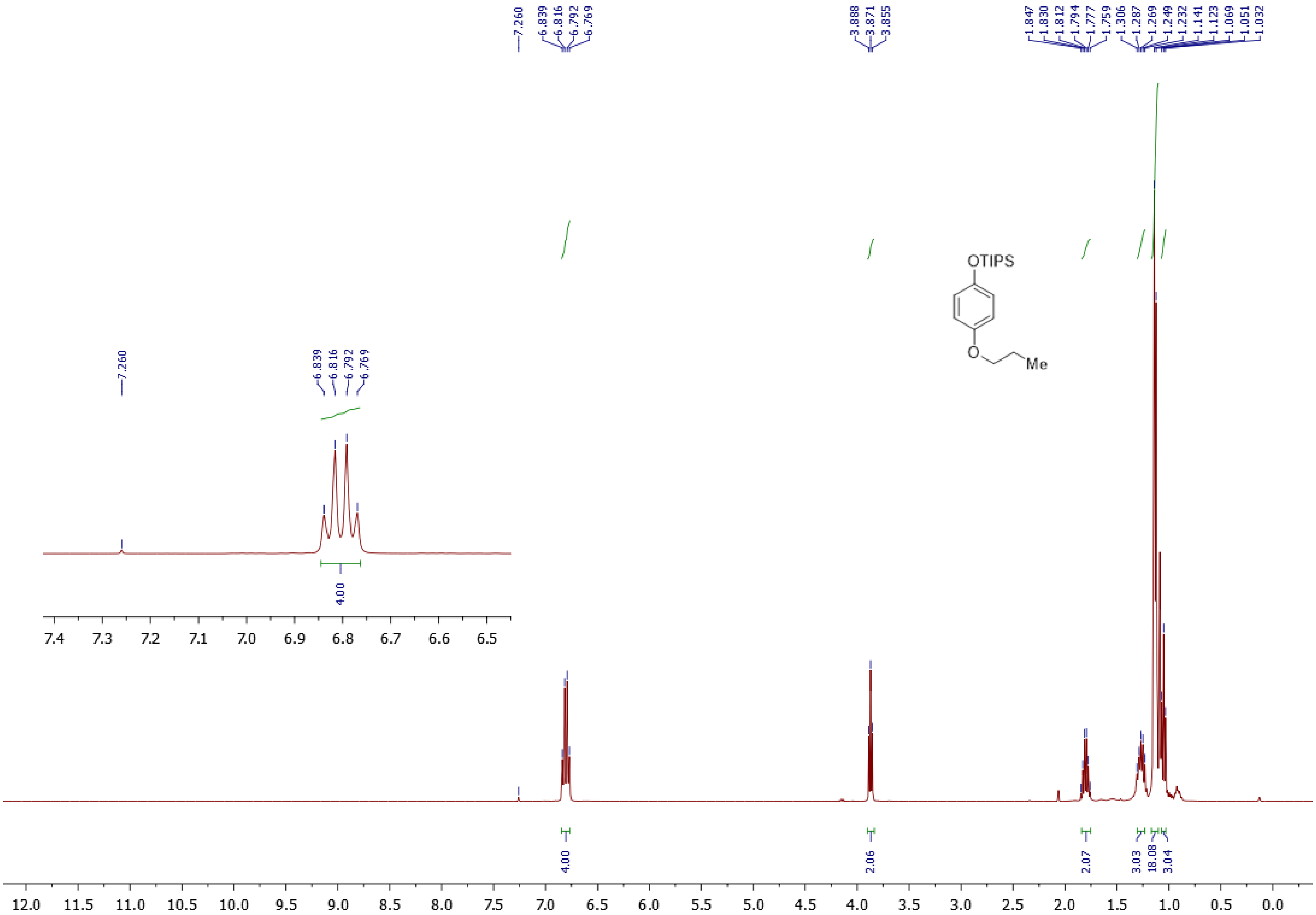

^{1}H-NMR spectra of **6g** (25 ^{o}C, 400 MHz, CDCl$_3$)

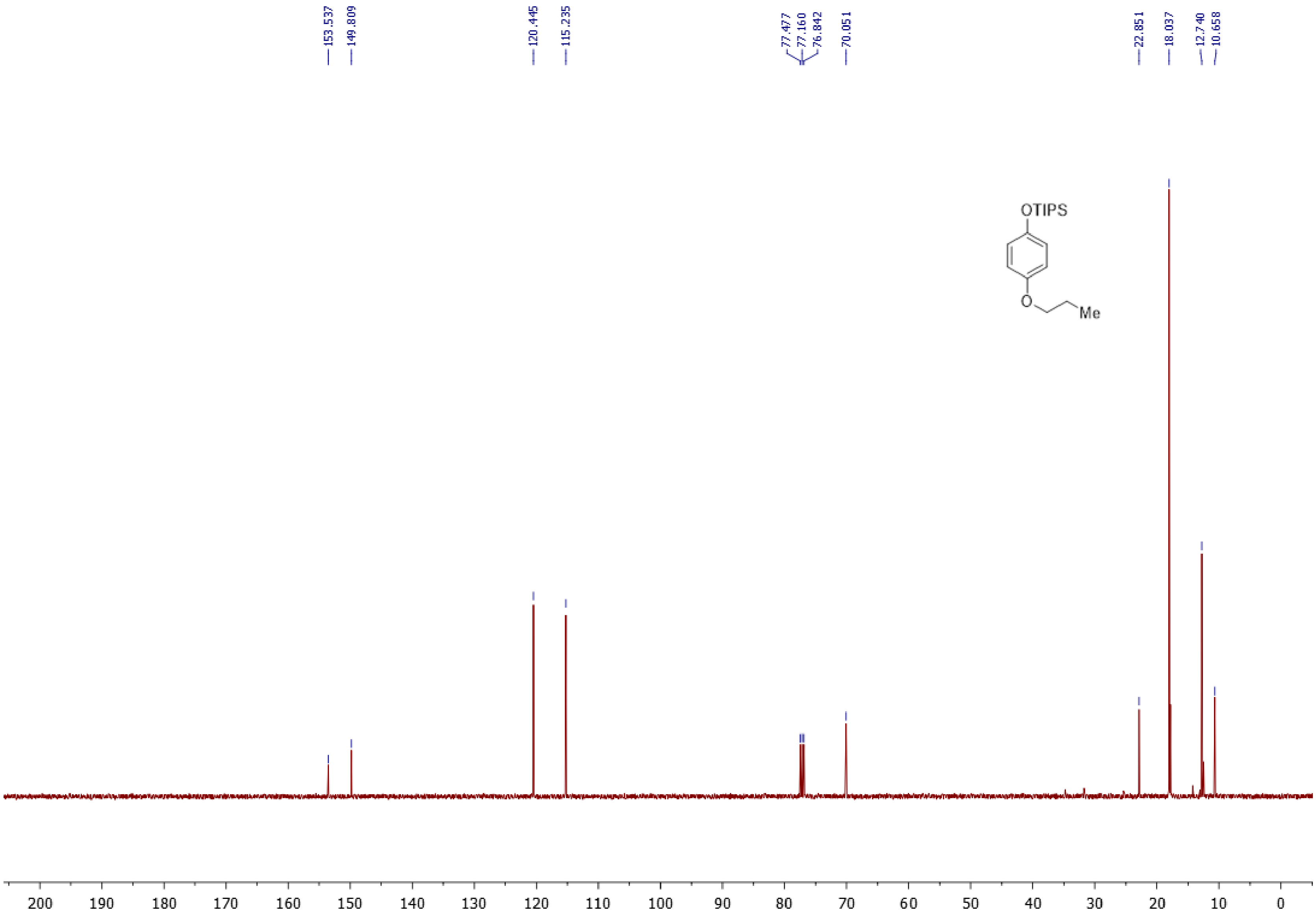

^{13}C-NMR spectra of **6g** (25 °C, 100 MHz, $CDCl_3$)

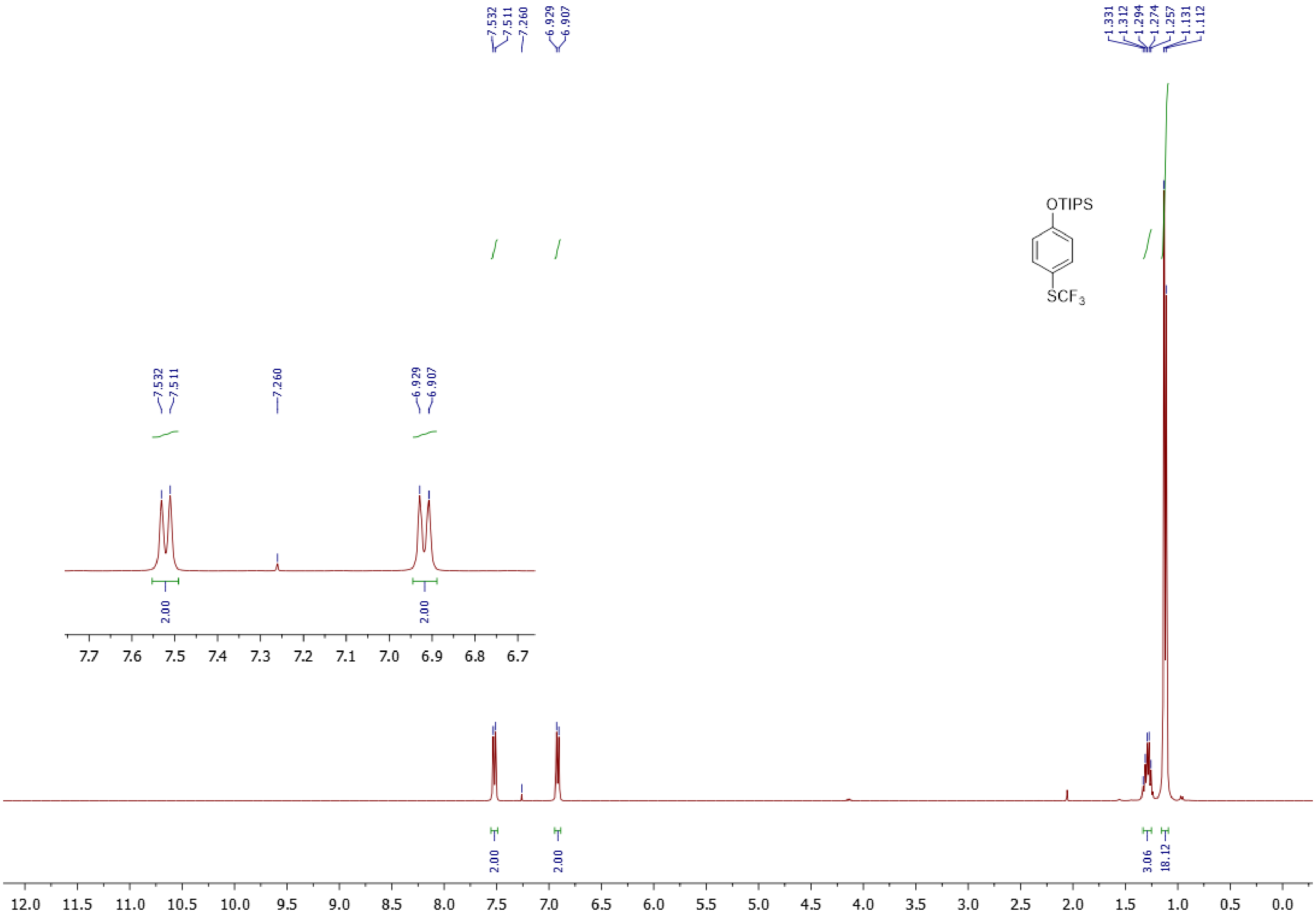

^{1}H-NMR spectra of **6i** (25 °C, 400 MHz, $CDCl_3$)

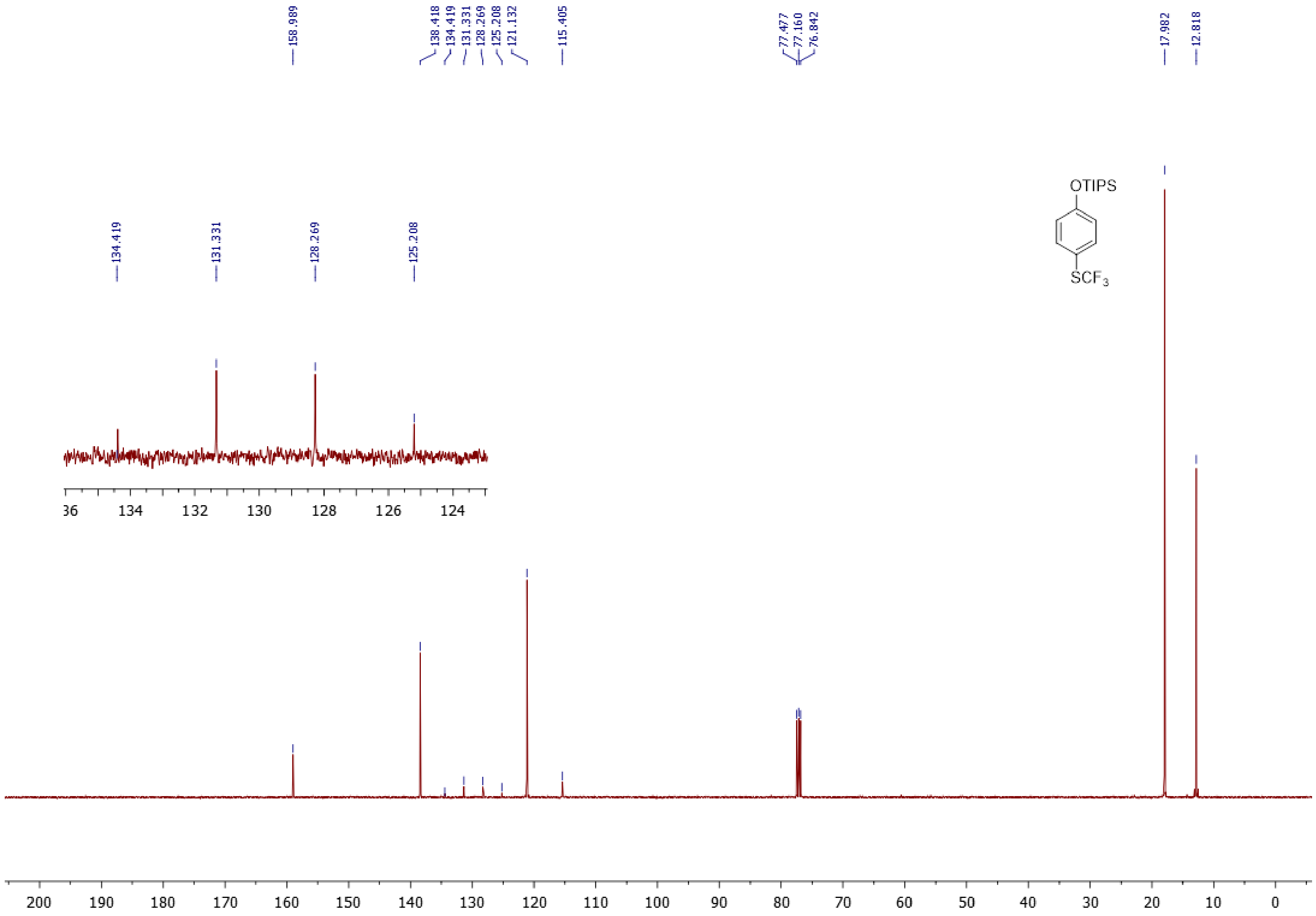

^{13}C-NMR spectra of **6i** (25 °C, 100 MHz, $CDCl_3$)

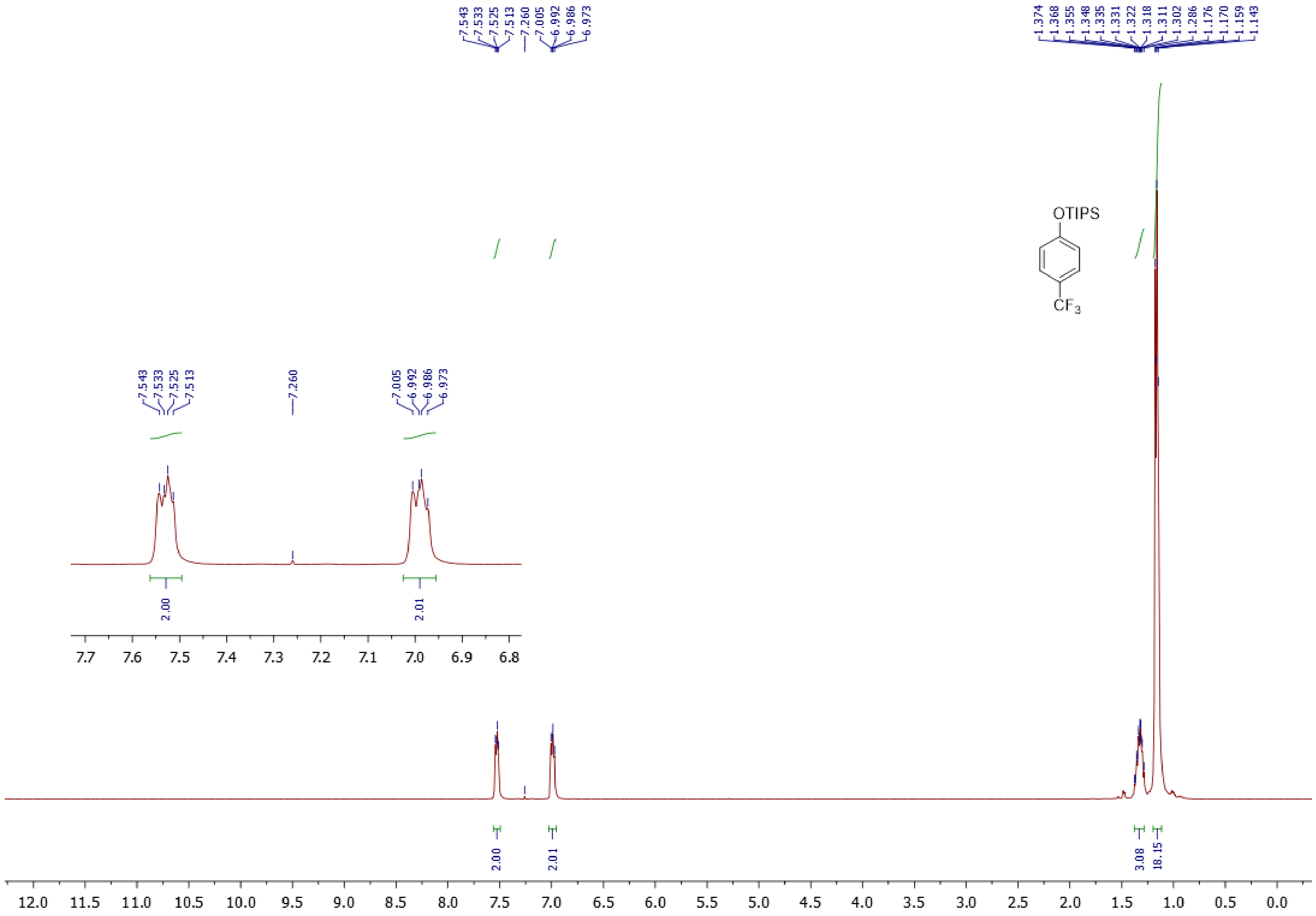

^{1}H-NMR spectra of **6l** (25 ^{o}C, 400 MHz, $CDCl_3$)

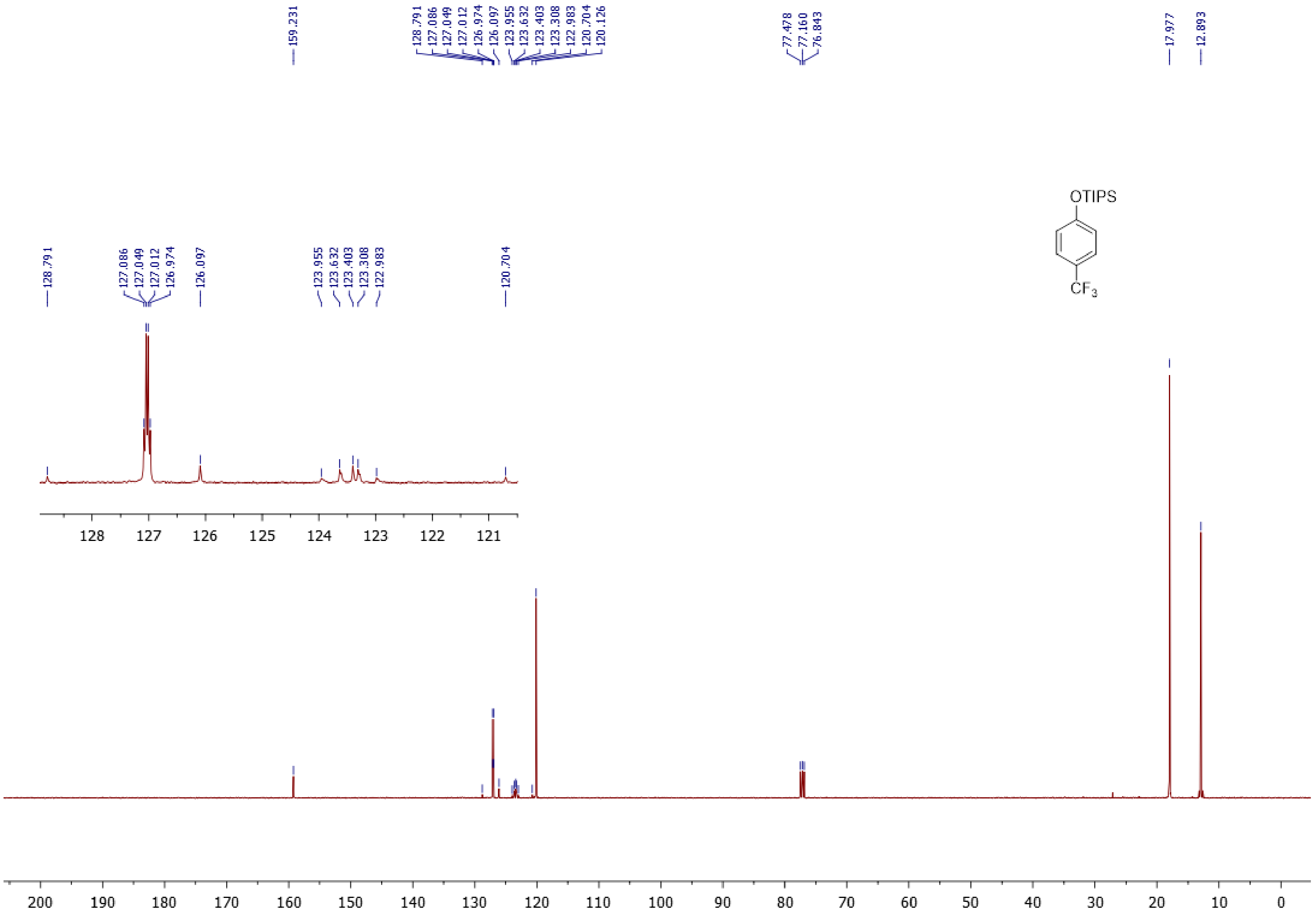

^{13}C-NMR spectra of **6l** (25 ^{o}C, 100 MHz, $CDCl_3$)

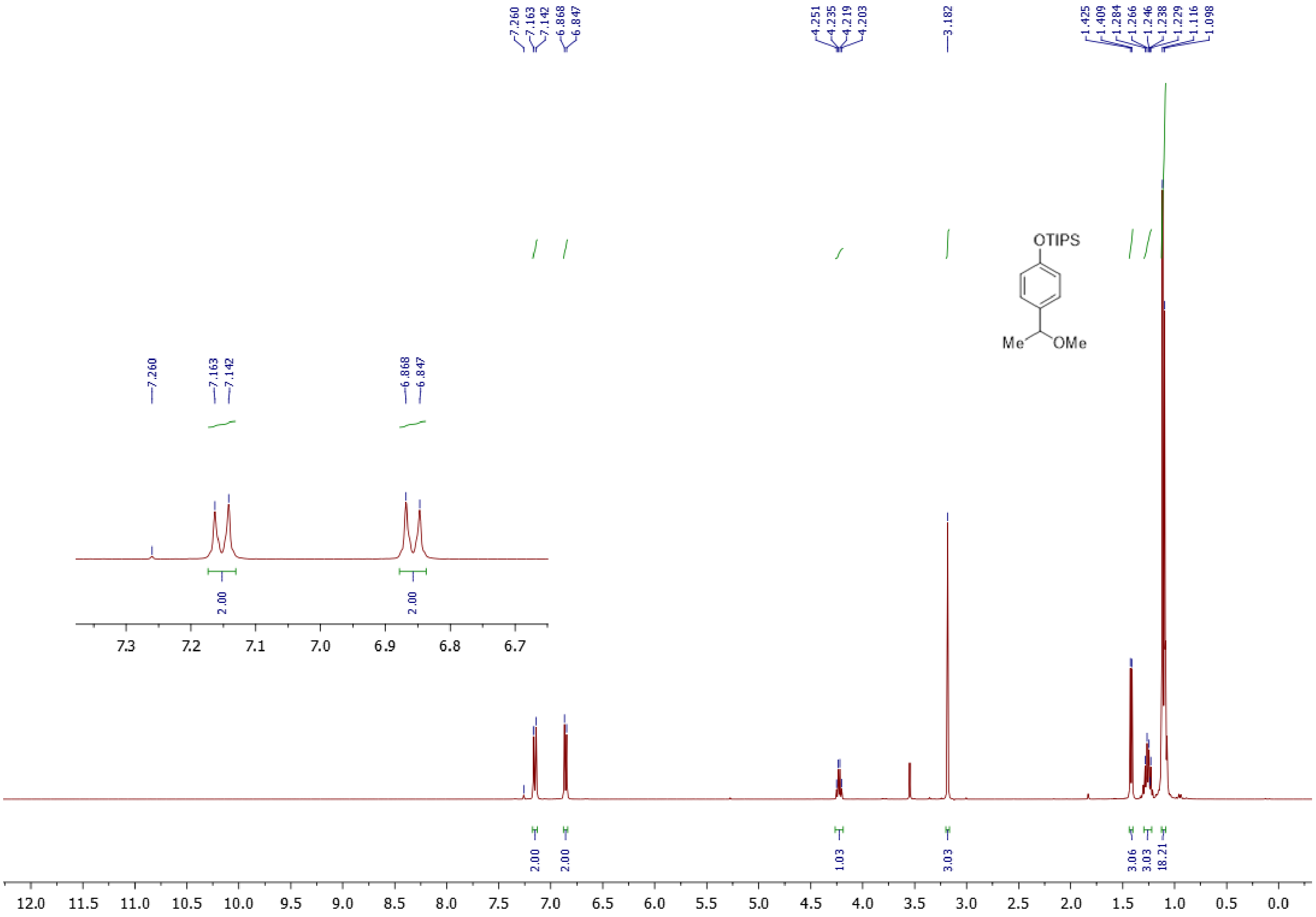

^{1}H-NMR spectra of **6o** (25 °C, 400 MHz, $CDCl_3$)

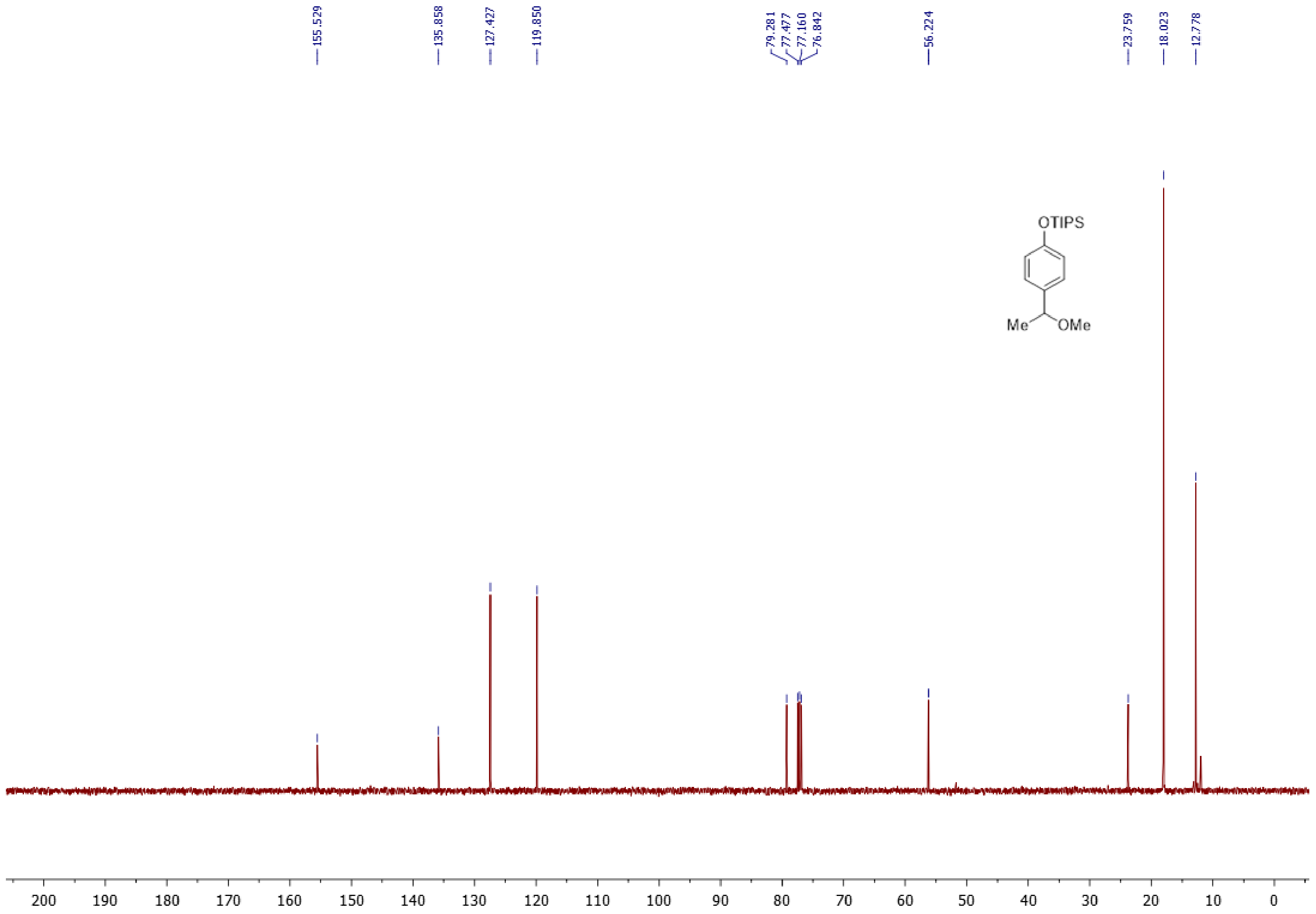

^{13}C-NMR spectra of **6o** (25 °C, 100 MHz, $CDCl_3$)

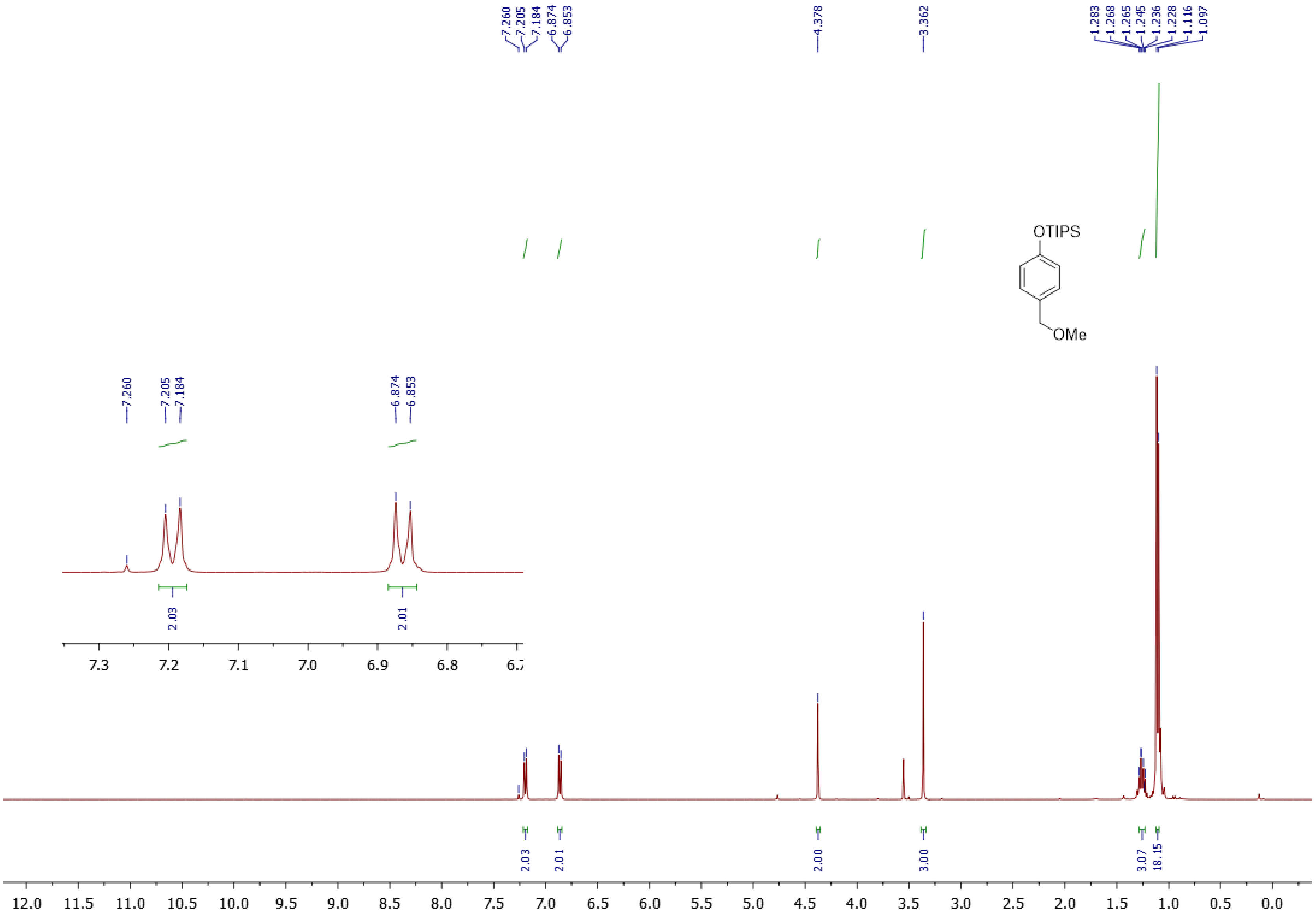

^{1}H-NMR spectra of **6p** (25 °C, 400 MHz, $CDCl_3$)

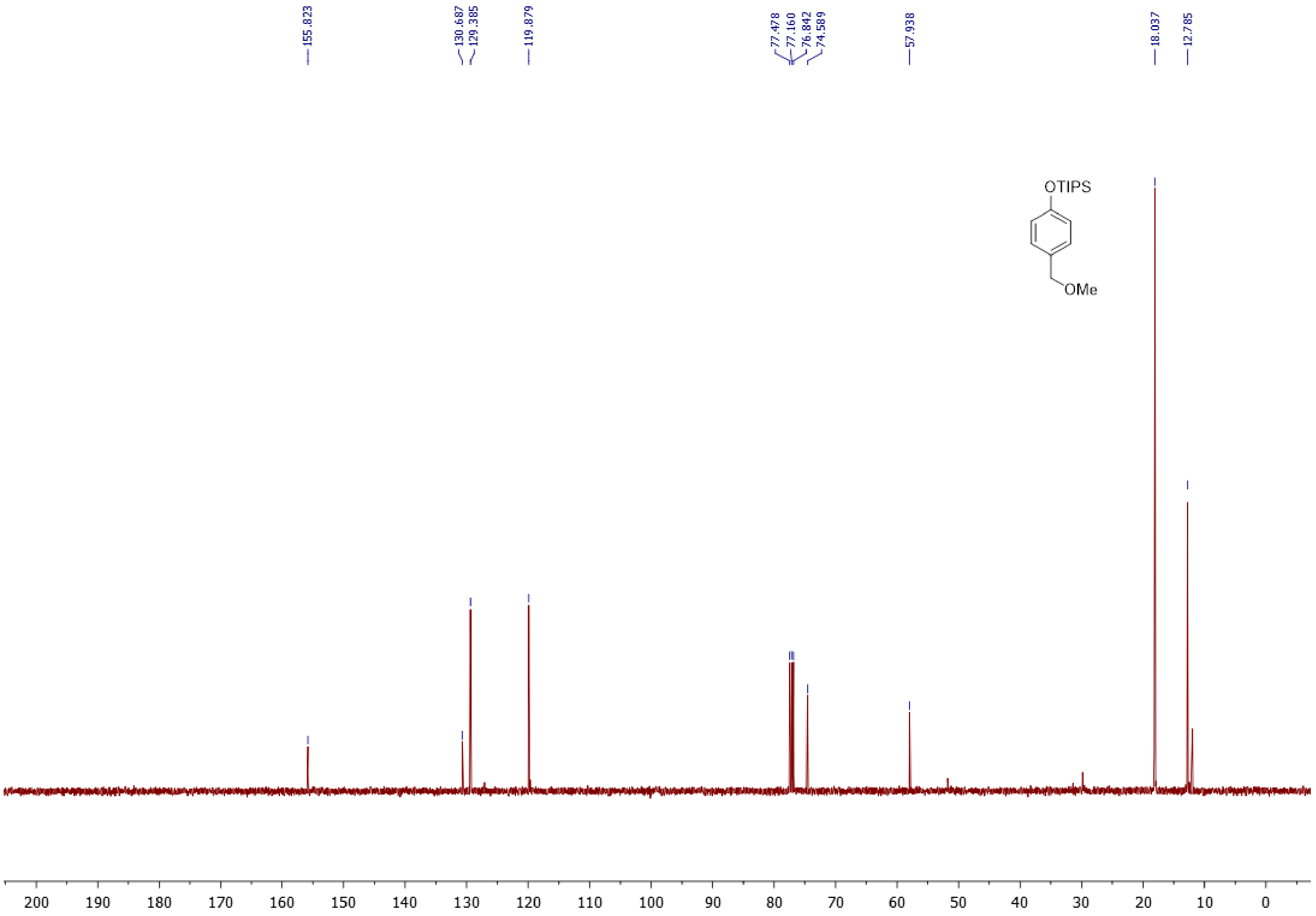

^{13}C-NMR spectra of **6p** (25 °C, 100 MHz, $CDCl_3$)

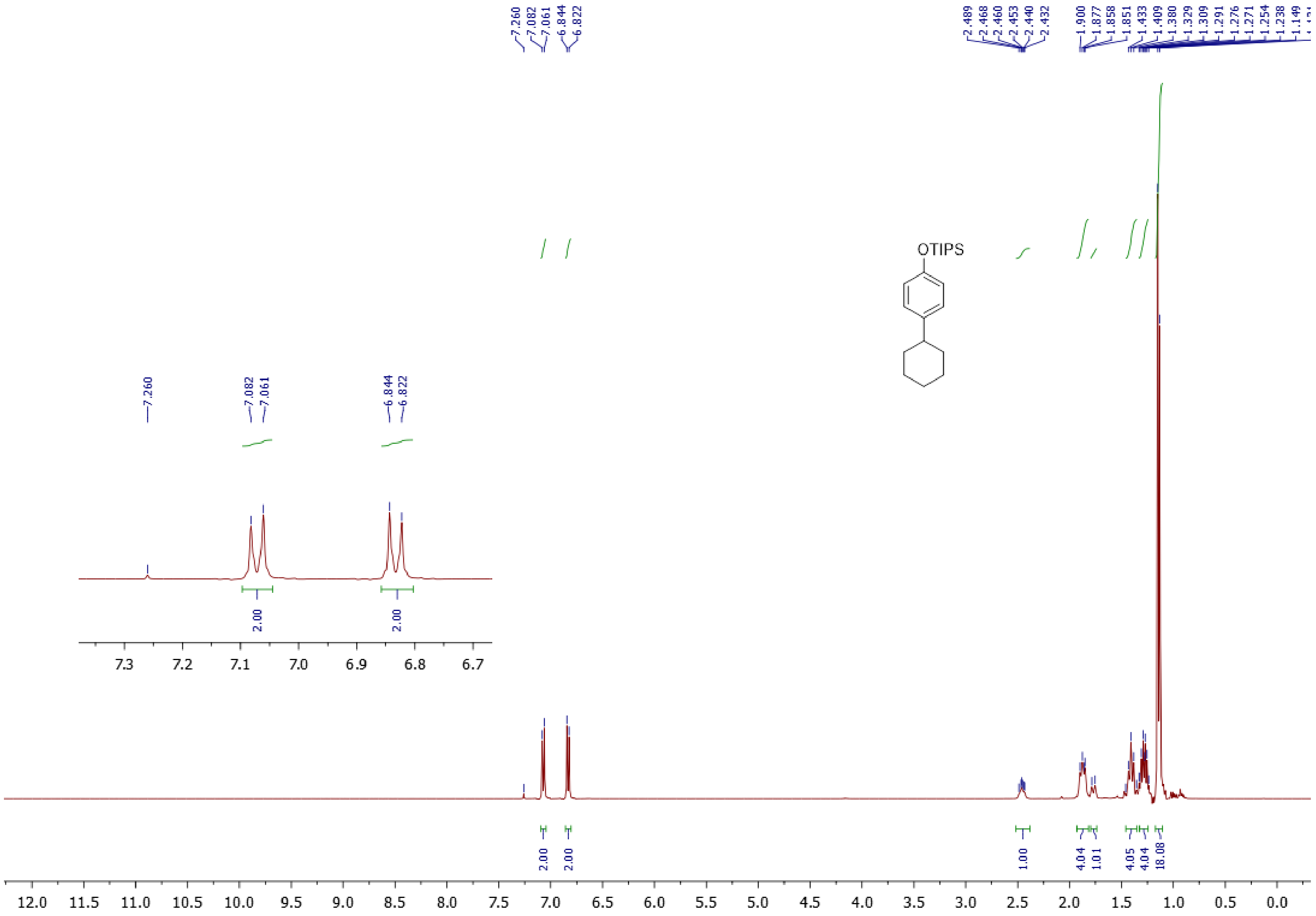

^{1}H-NMR spectra of **6r** (25 °C, 400 MHz, $CDCl_3$)

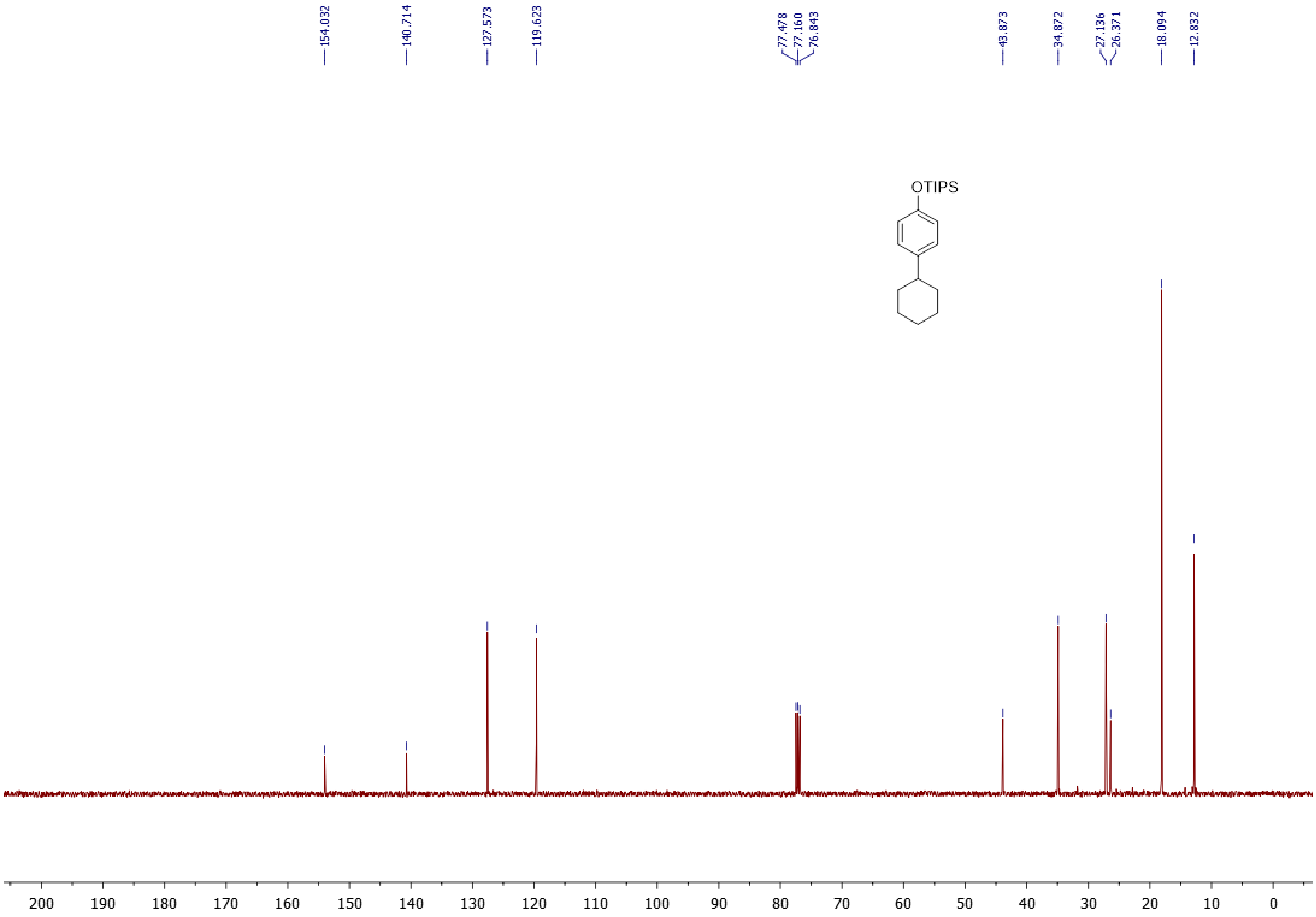

^{13}C-NMR spectra of **6r** (25 ^{o}C, 100 MHz, $CDCl_3$)

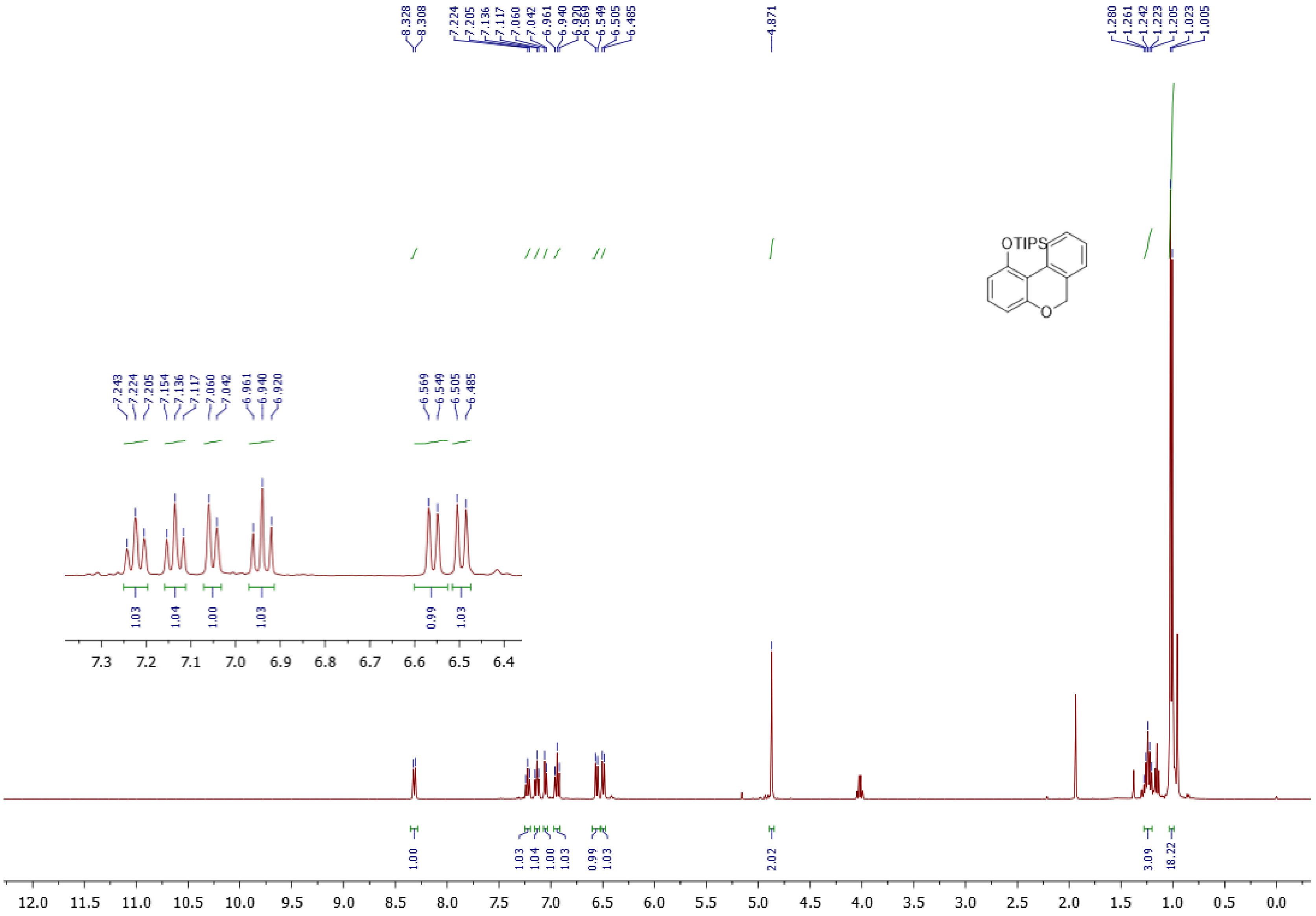

^{1}H-NMR spectra of **10a** (25 °C, 400 MHz, $CDCl_3$)

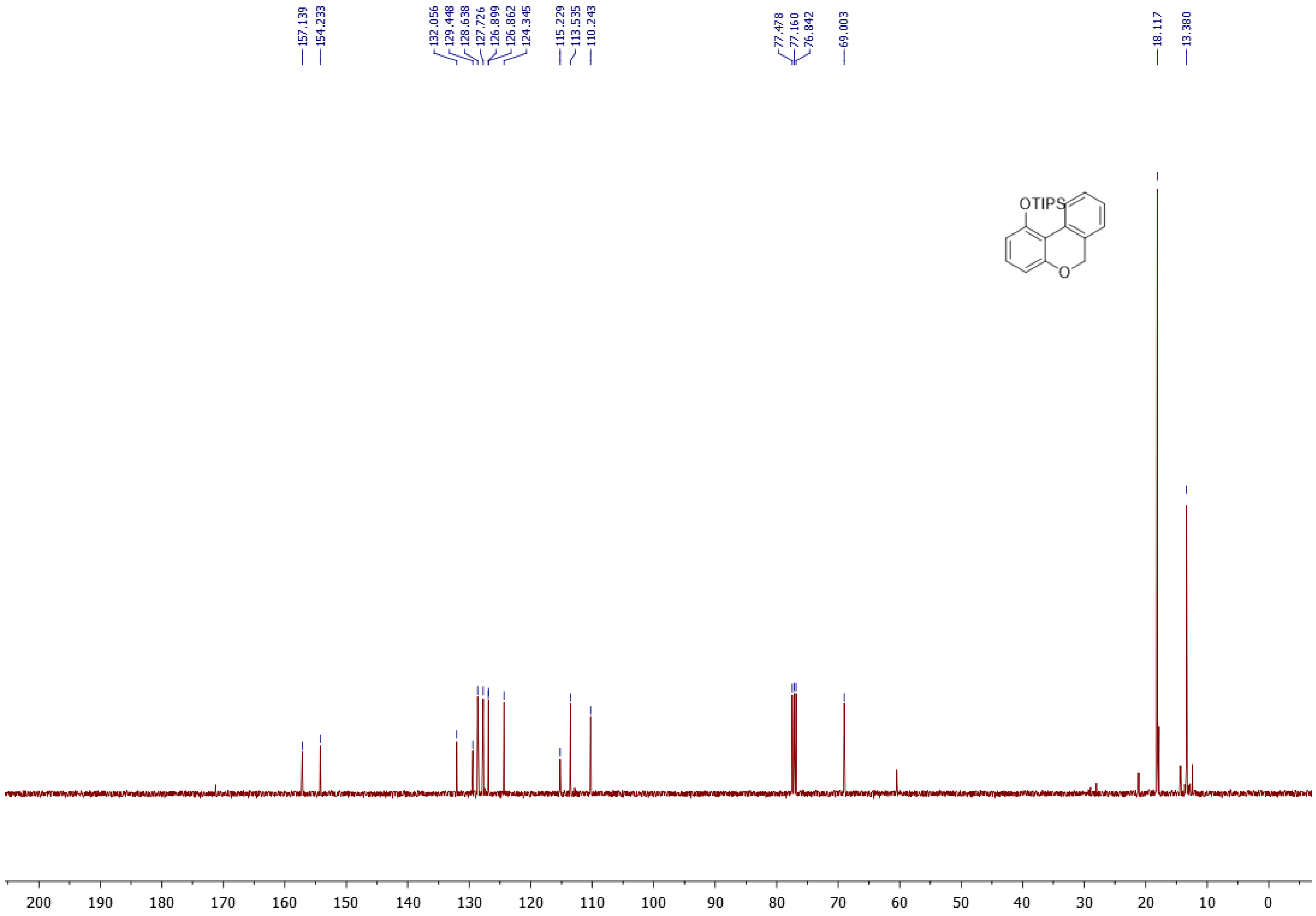

^{13}C-NMR spectra of **10a** (25 °C, 100 MHz, $CDCl_3$)

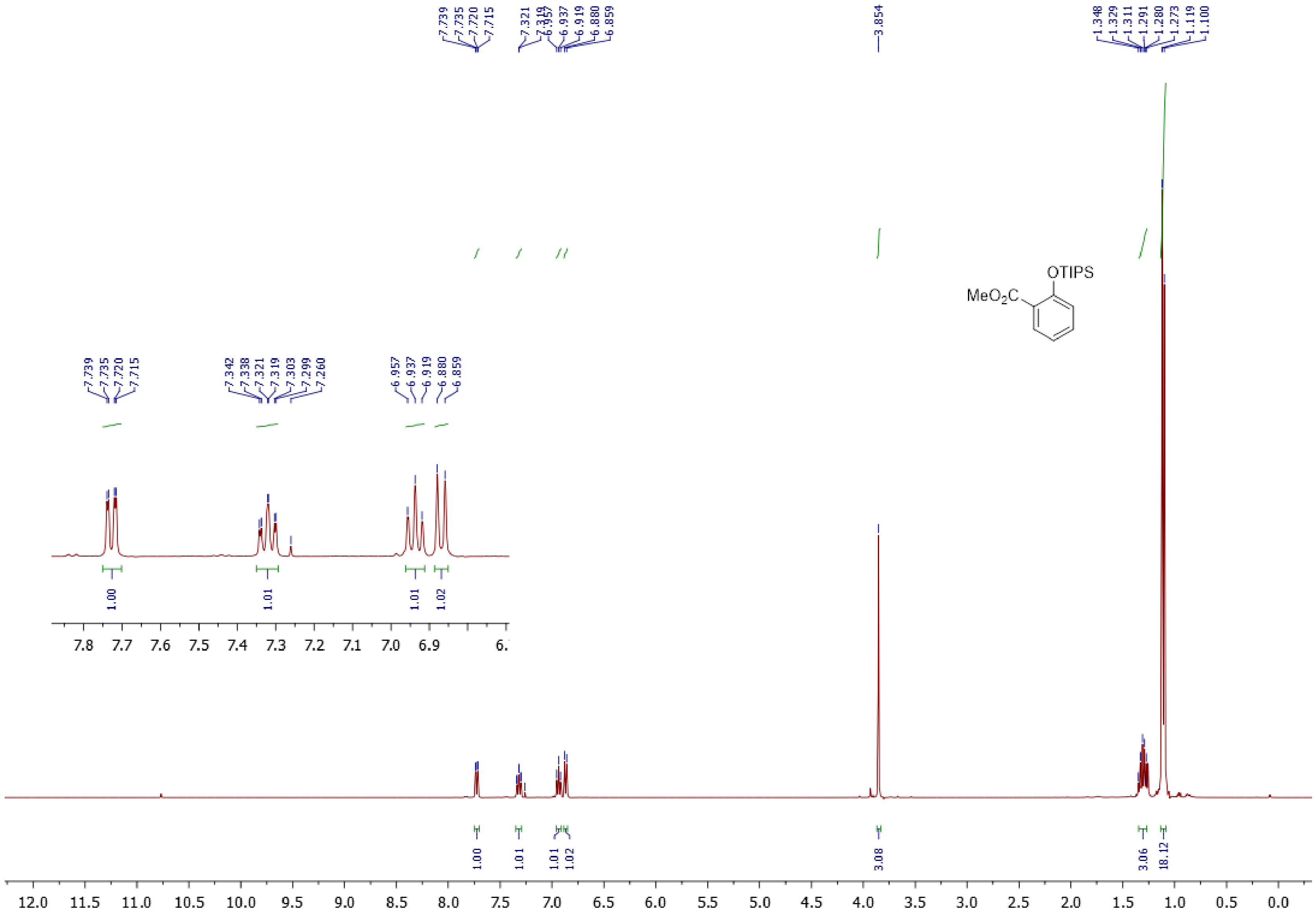

^{1}H-NMR spectra of **10b** (25 ^{o}C, 400 MHz, $CDCl_3$)

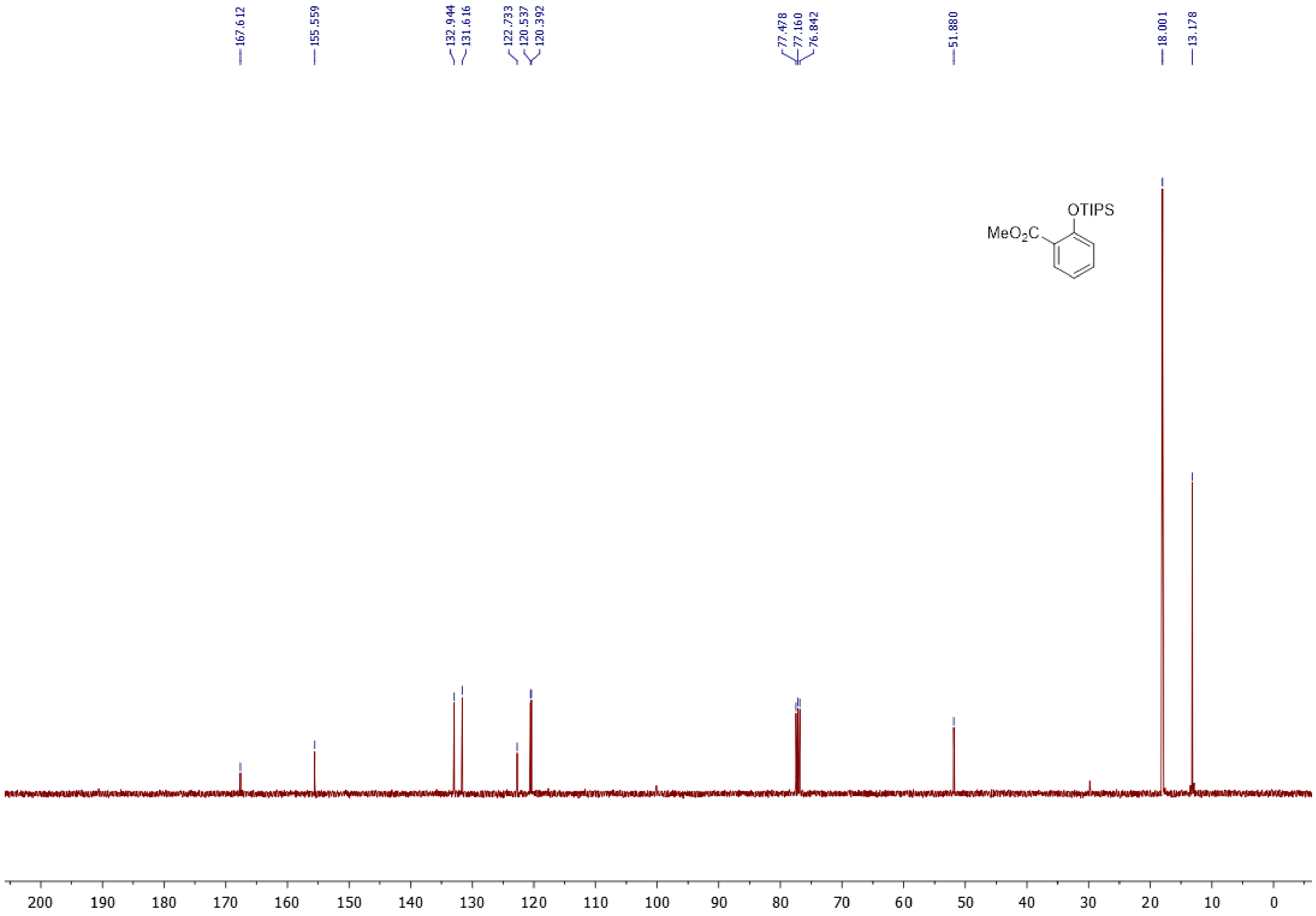

^{13}C-NMR spectra of **10b** (25 °C, 100 MHz, $CDCl_3$)

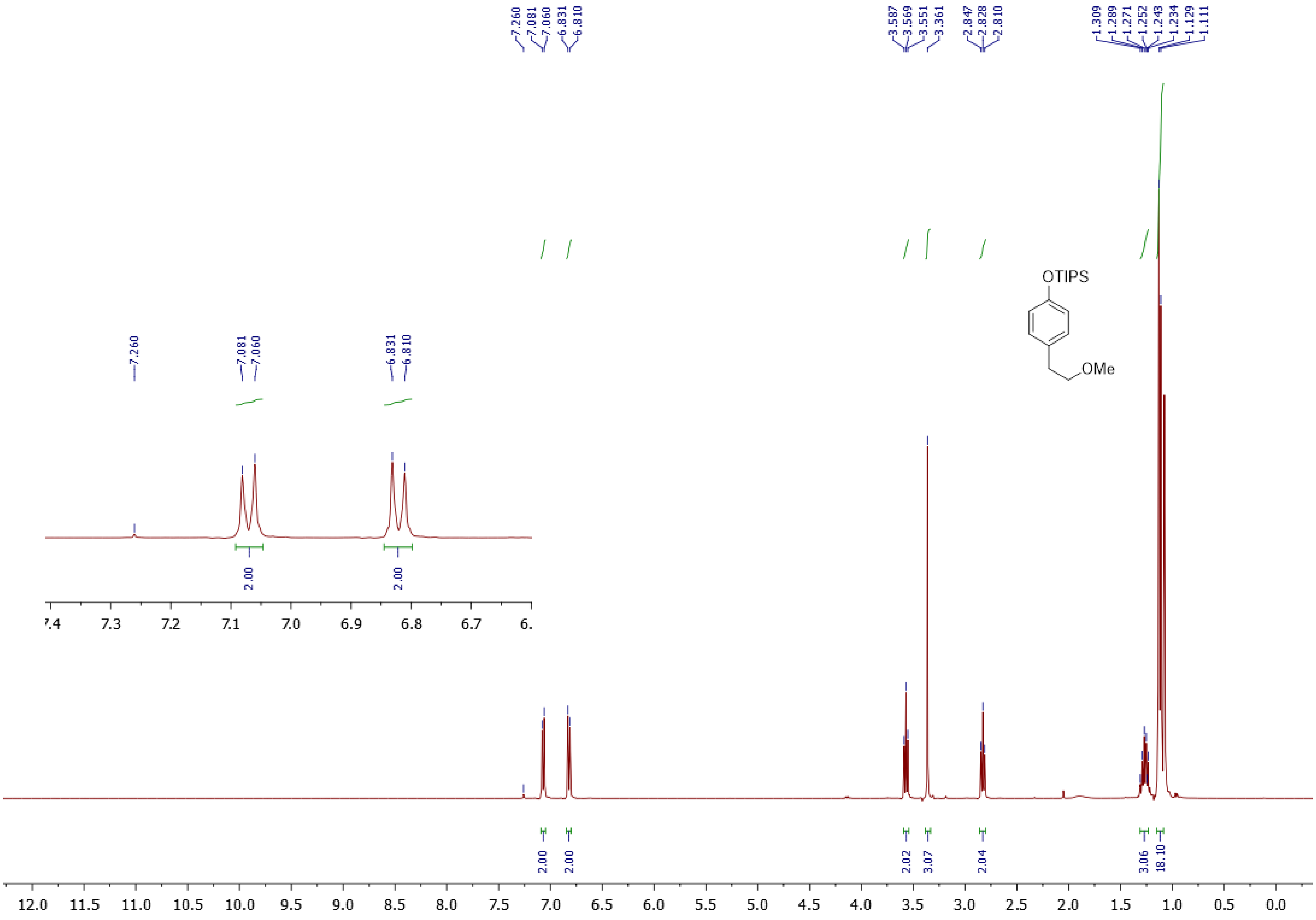

^{1}H-NMR spectra of **10c** (25 ^{o}C, 400 MHz, $CDCl_3$)

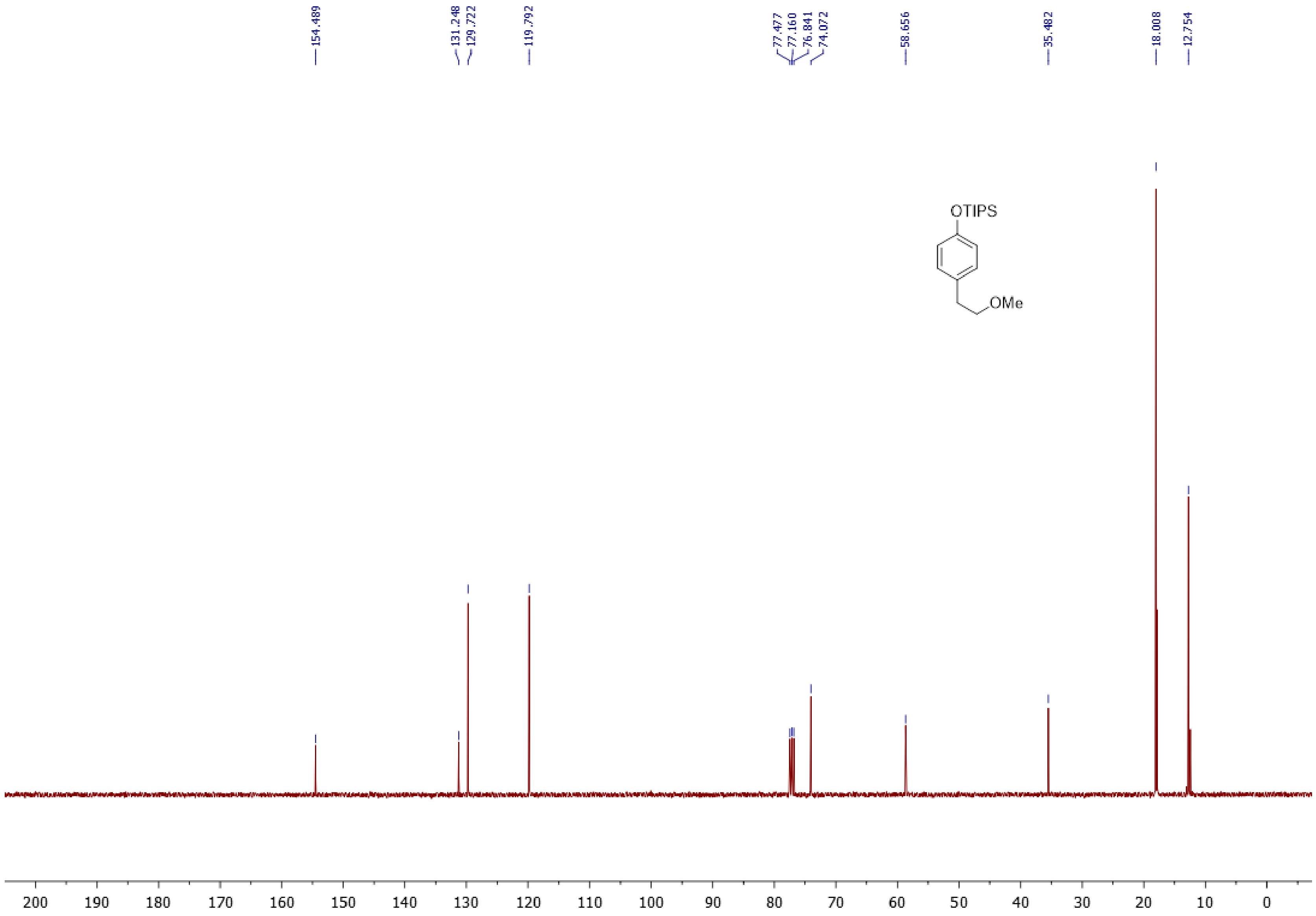

^{13}C-NMR spectra of **10c** (25 ^{o}C, 100 MHz, $CDCl_3$)

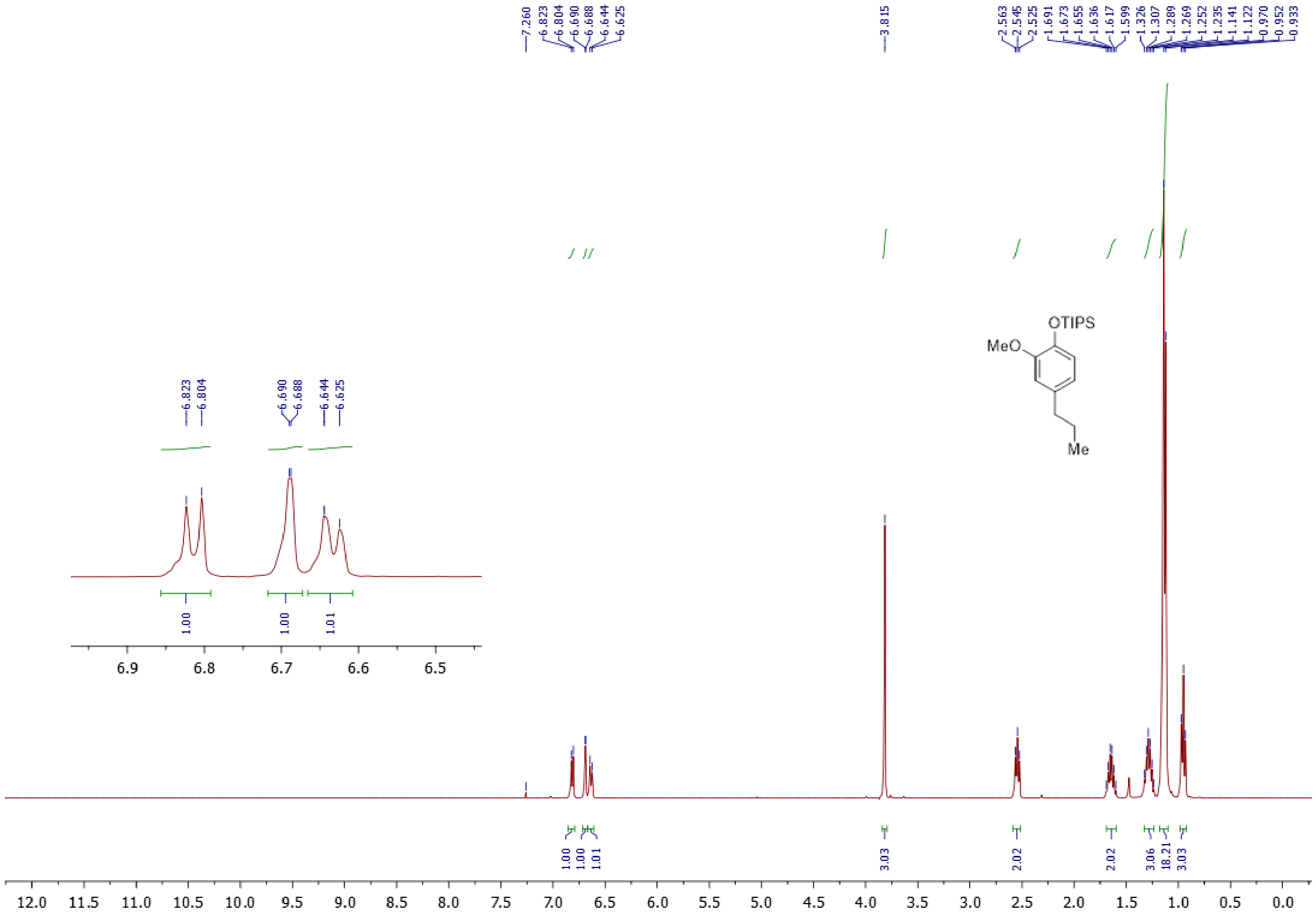

^{1}H-NMR spectra of **10d** (25 ^{o}C, 400 MHz, $CDCl_3$)

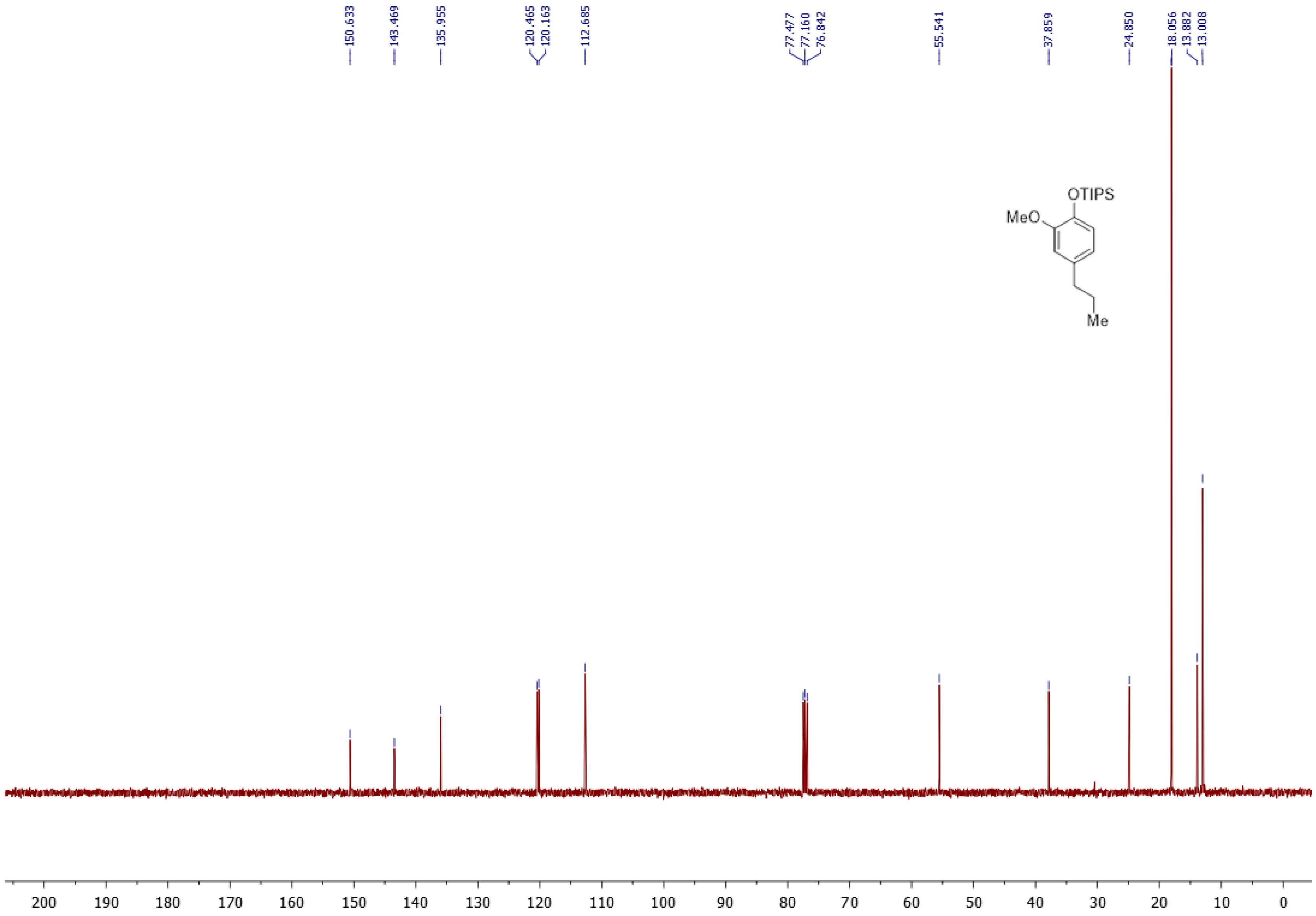

^{13}C-NMR spectra of **10d** (25 ^{o}C, 100 MHz, $CDCl_3$)

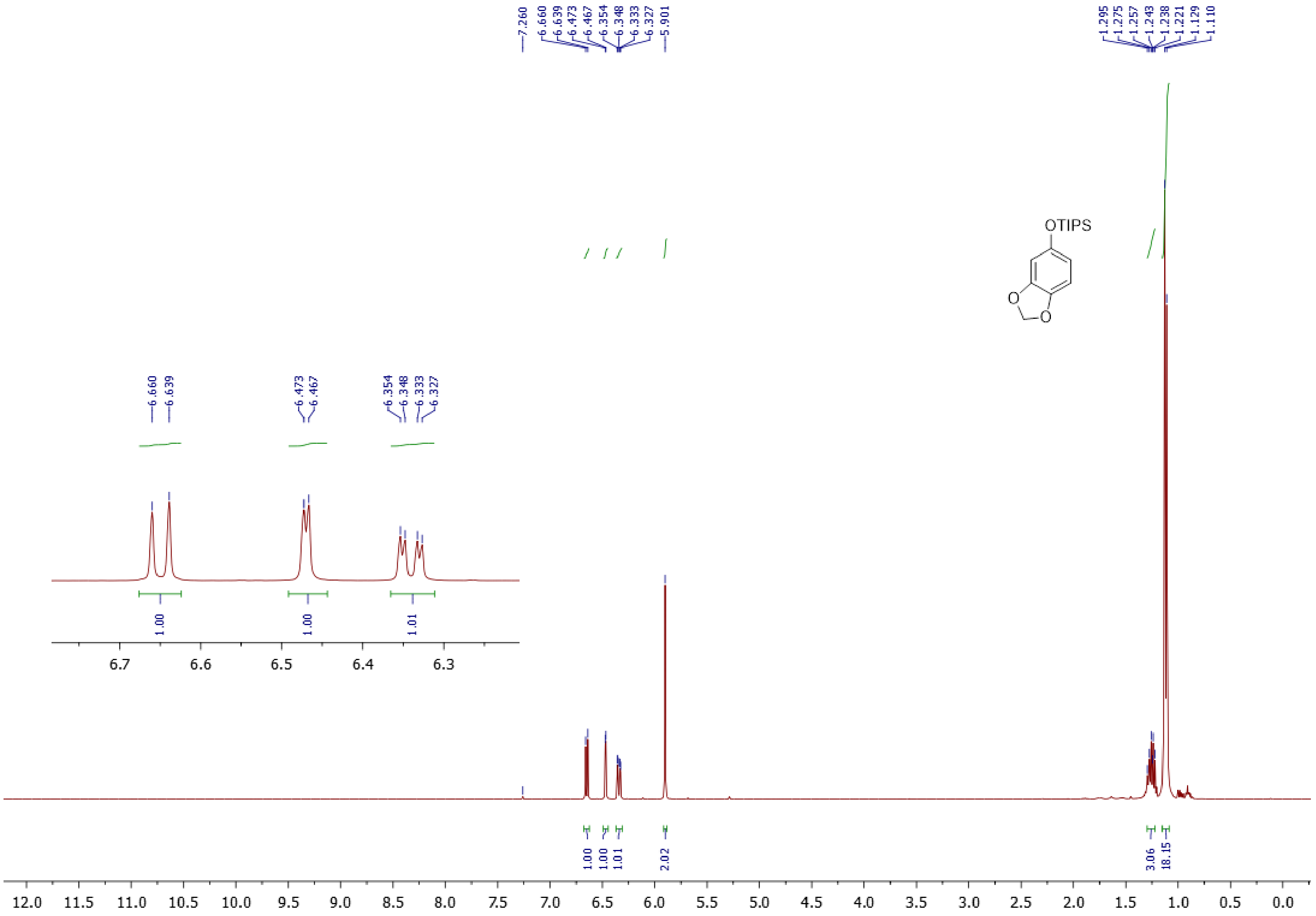

^{1}H-NMR spectra of **10e** (25 °C, 400 MHz, $CDCl_3$)

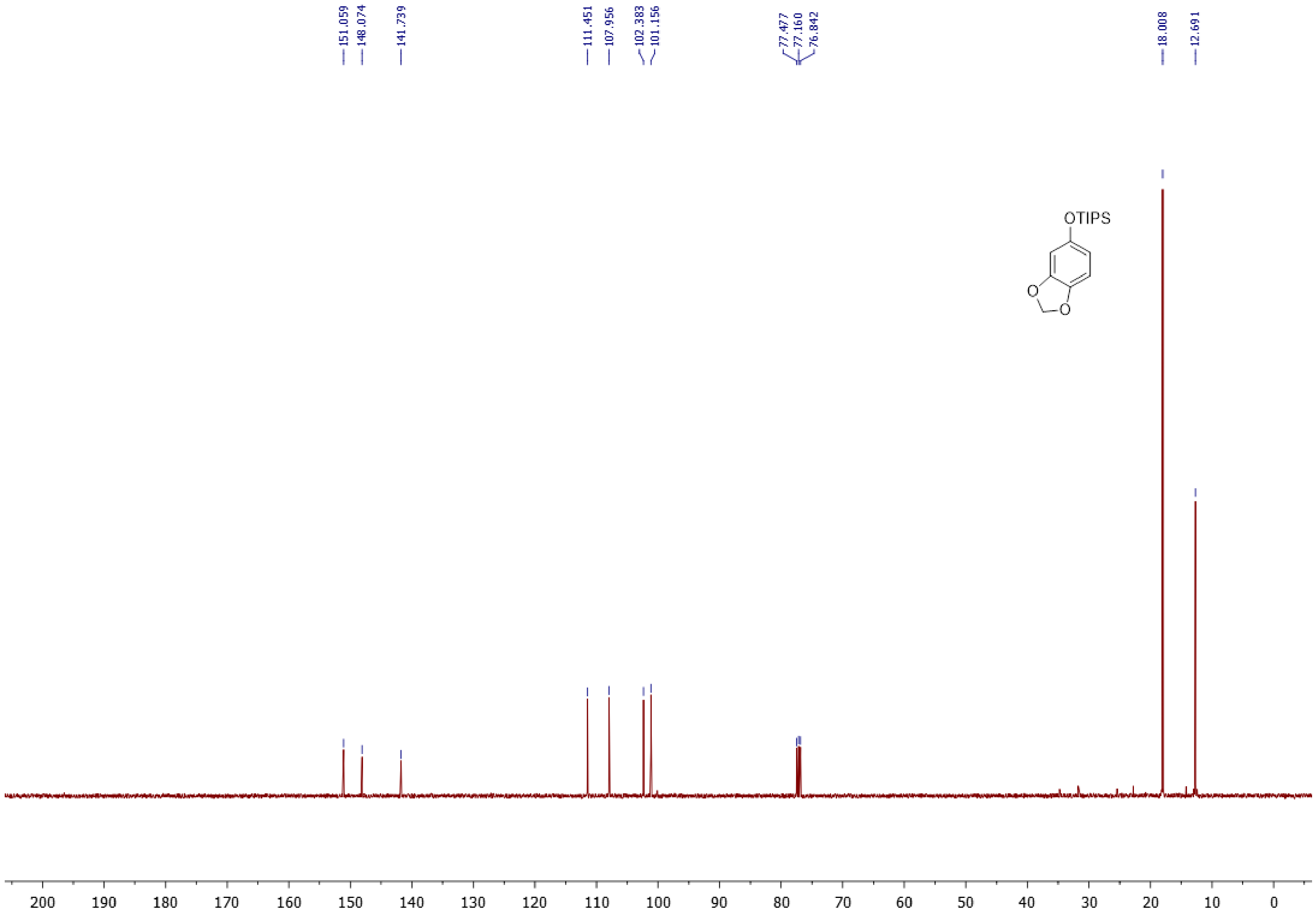

^{13}C-NMR spectra of **10e** (25 °C, 100 MHz, $CDCl_3$)

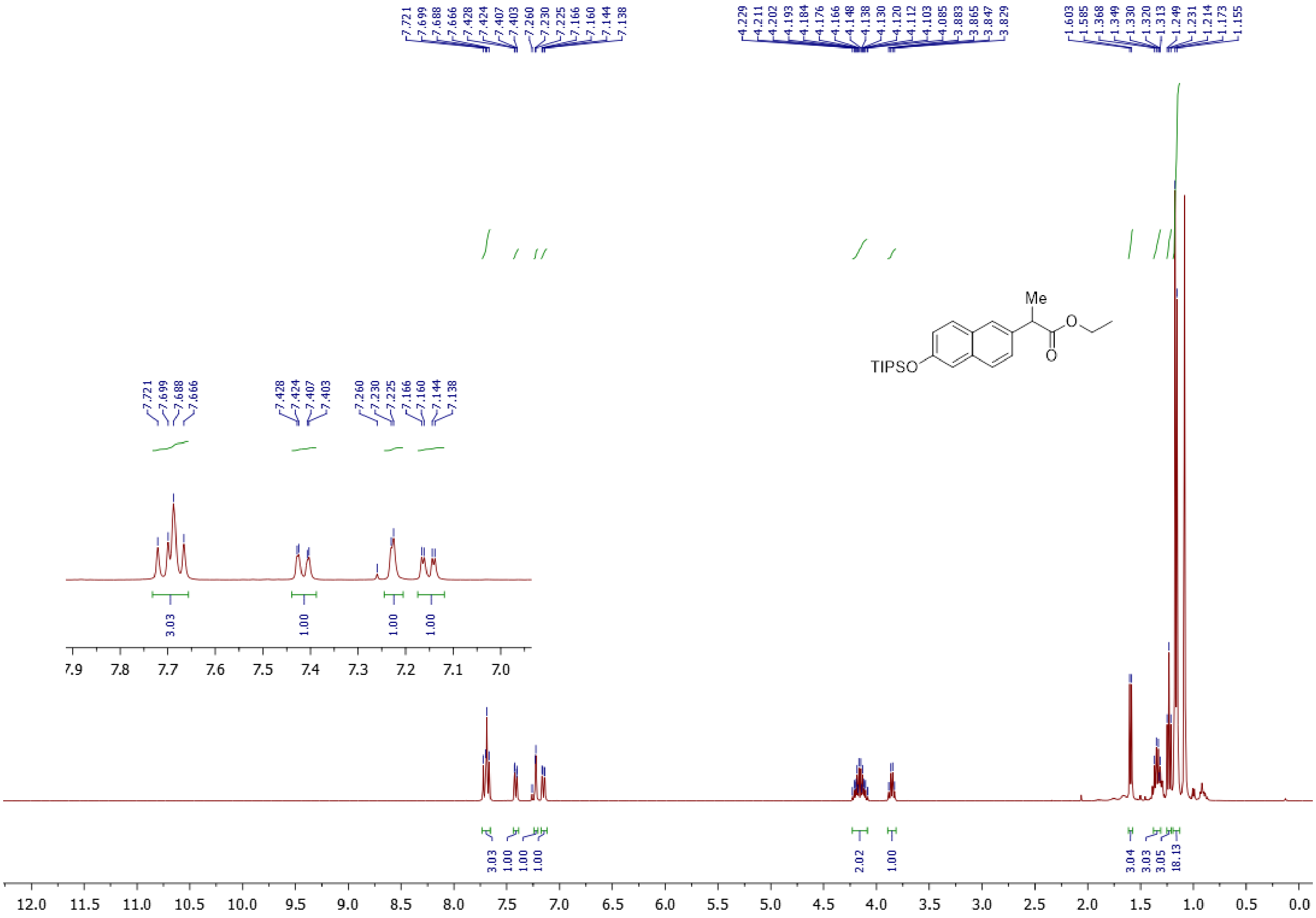

^{1}H-NMR spectra of **10f** (25 °C, 400 MHz, $CDCl_3$)

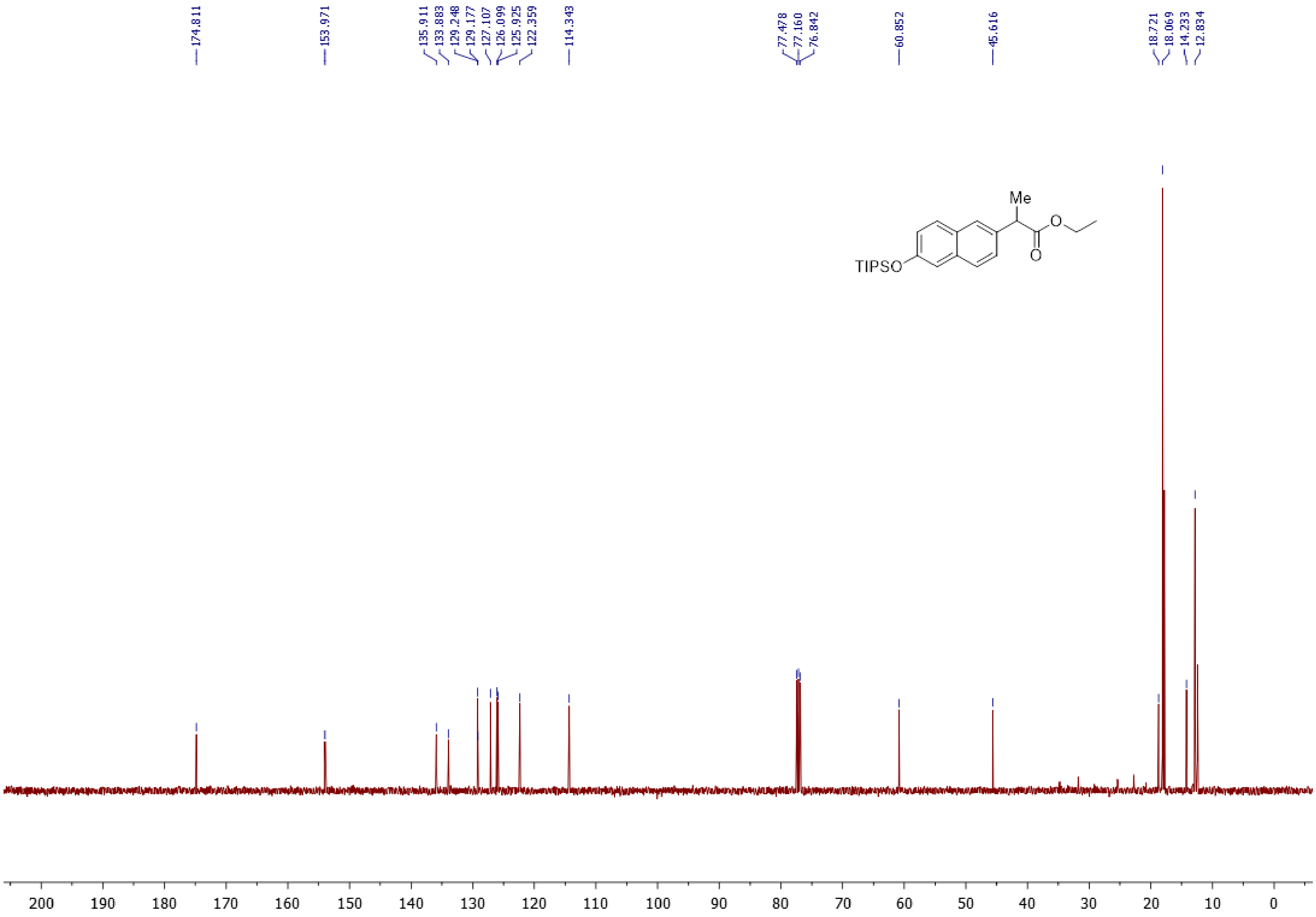

^{13}C-NMR spectra of **10f** (25 ^{o}C, 100 MHz, $CDCl_3$)

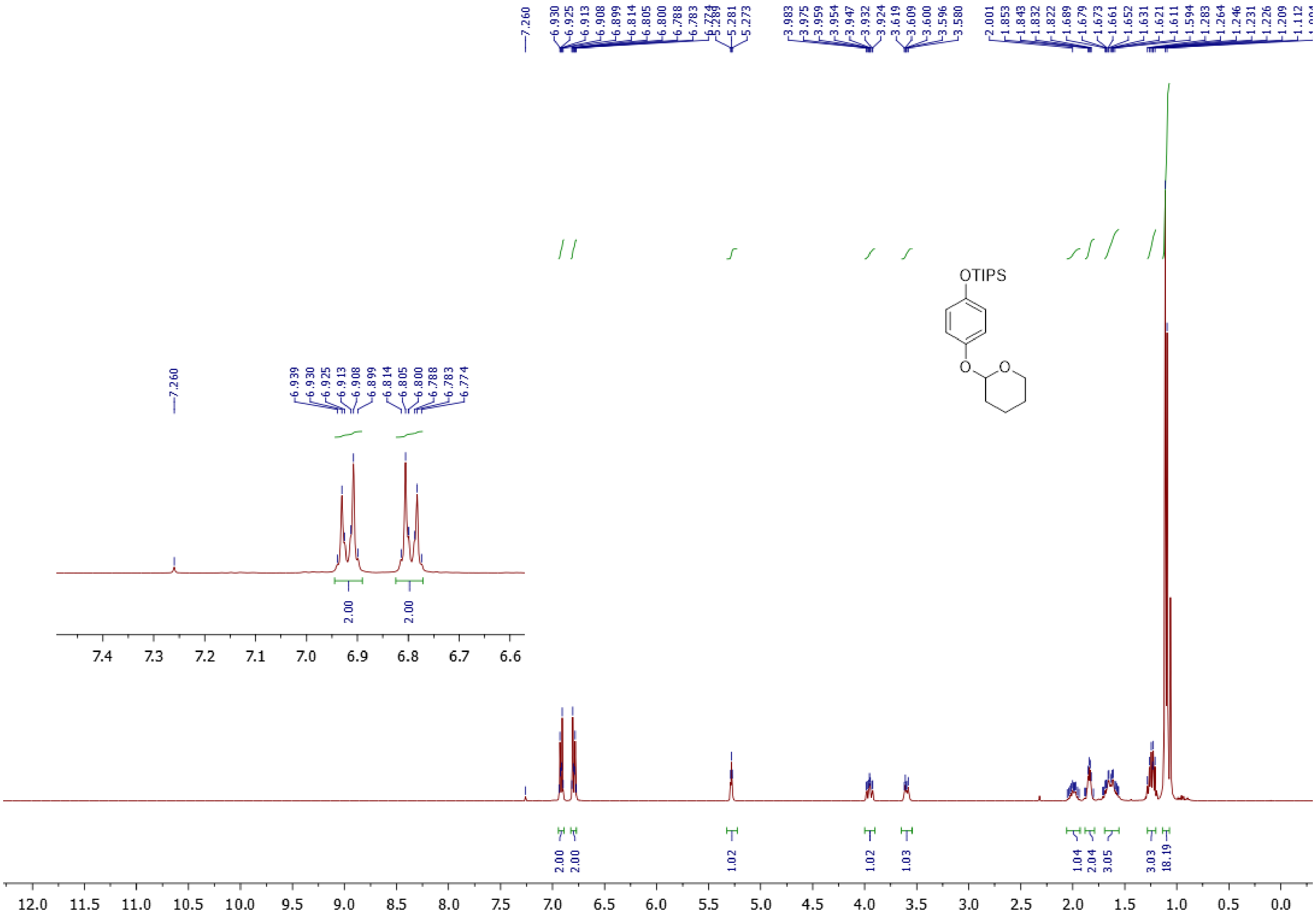

^{1}H-NMR spectra of **10g** (25 °C, 400 MHz, $CDCl_3$)

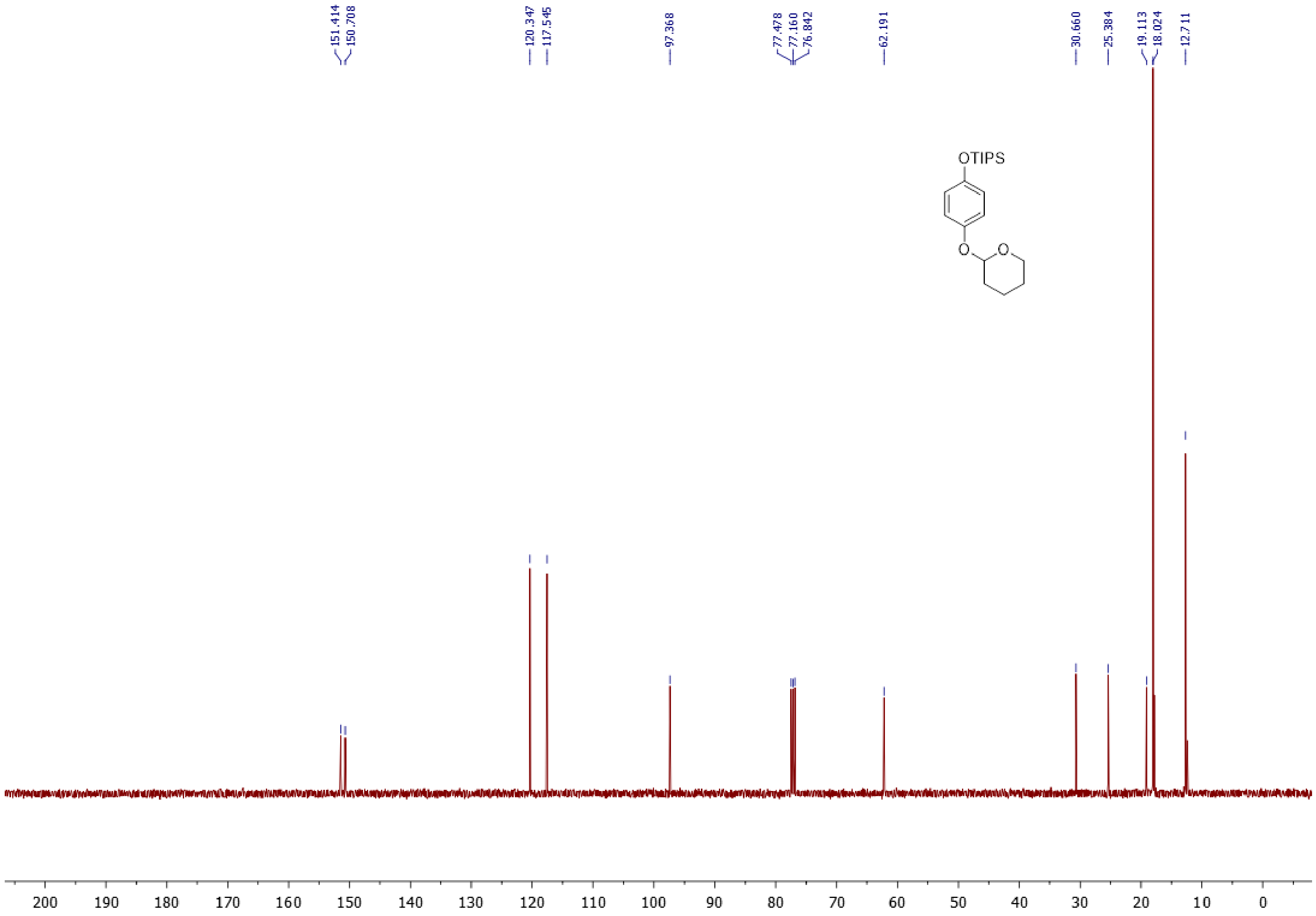

^{13}C-NMR spectra of **10g** (25 ^{o}C, 100 MHz, $CDCl_3$)

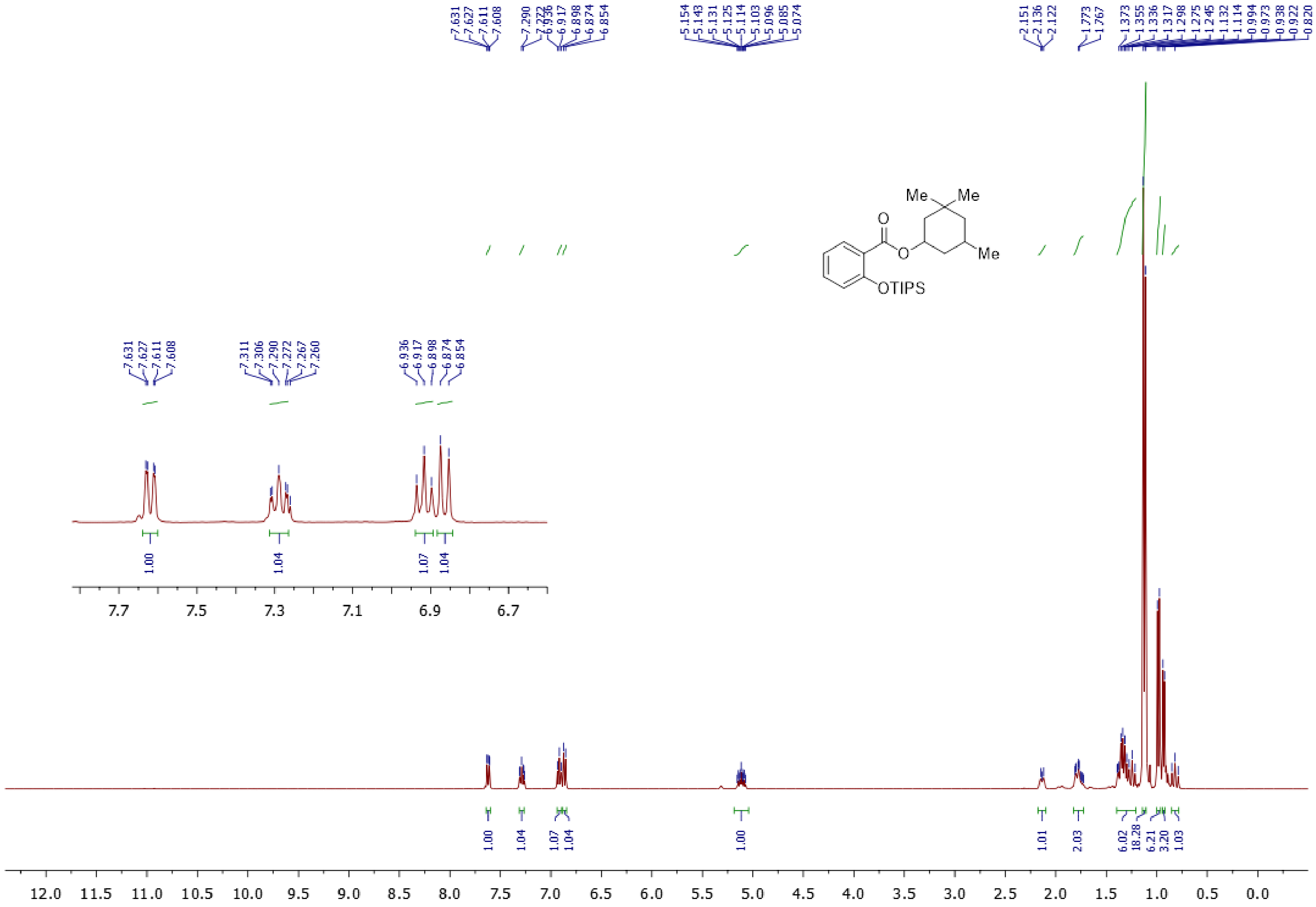

^{1}H-NMR spectra of **10h** (25 ^{o}C, 400 MHz, $CDCl_3$)

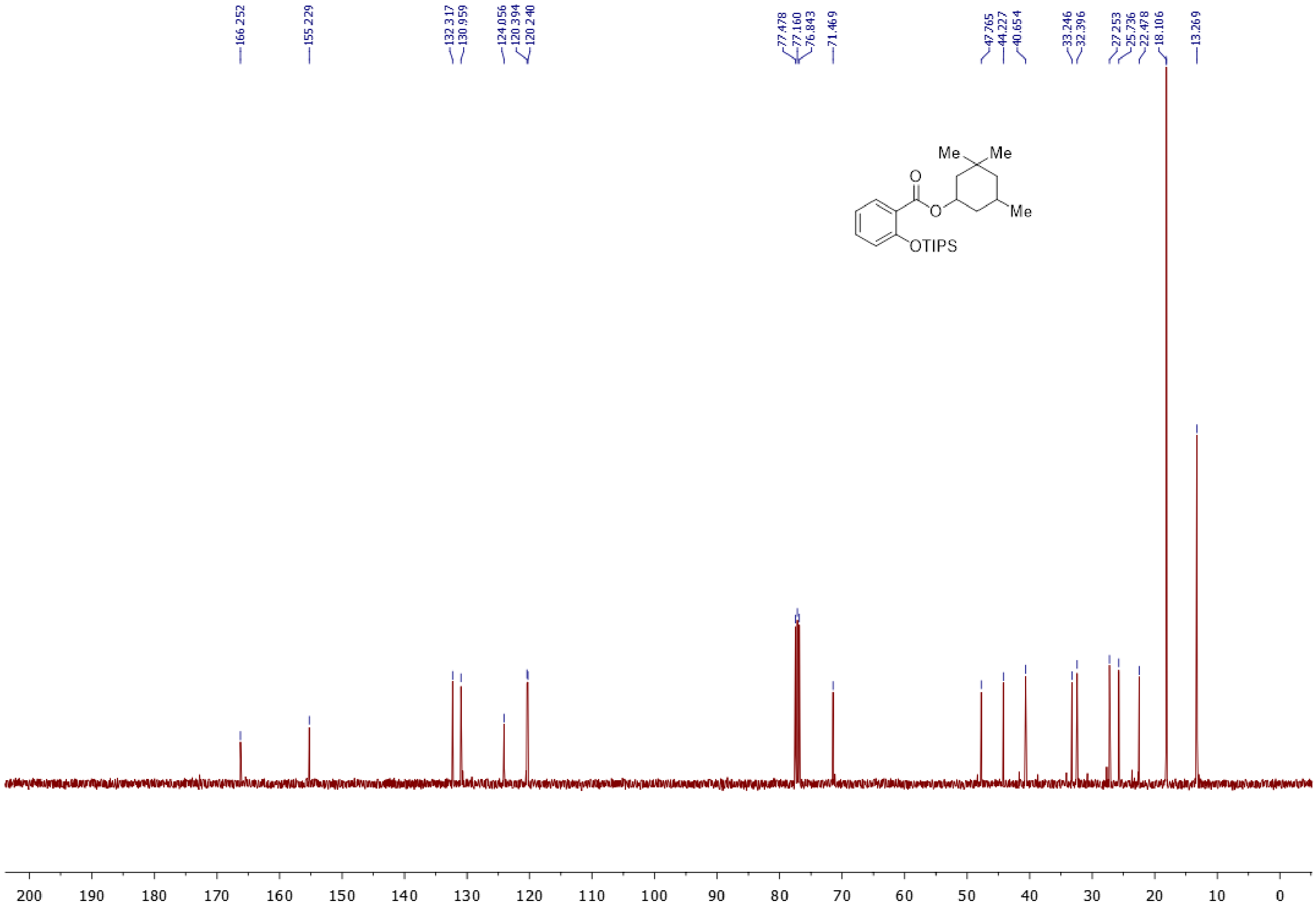

^{13}C-NMR spectra of **10h** (25 ^{o}C, 100 MHz, $CDCl_3$)

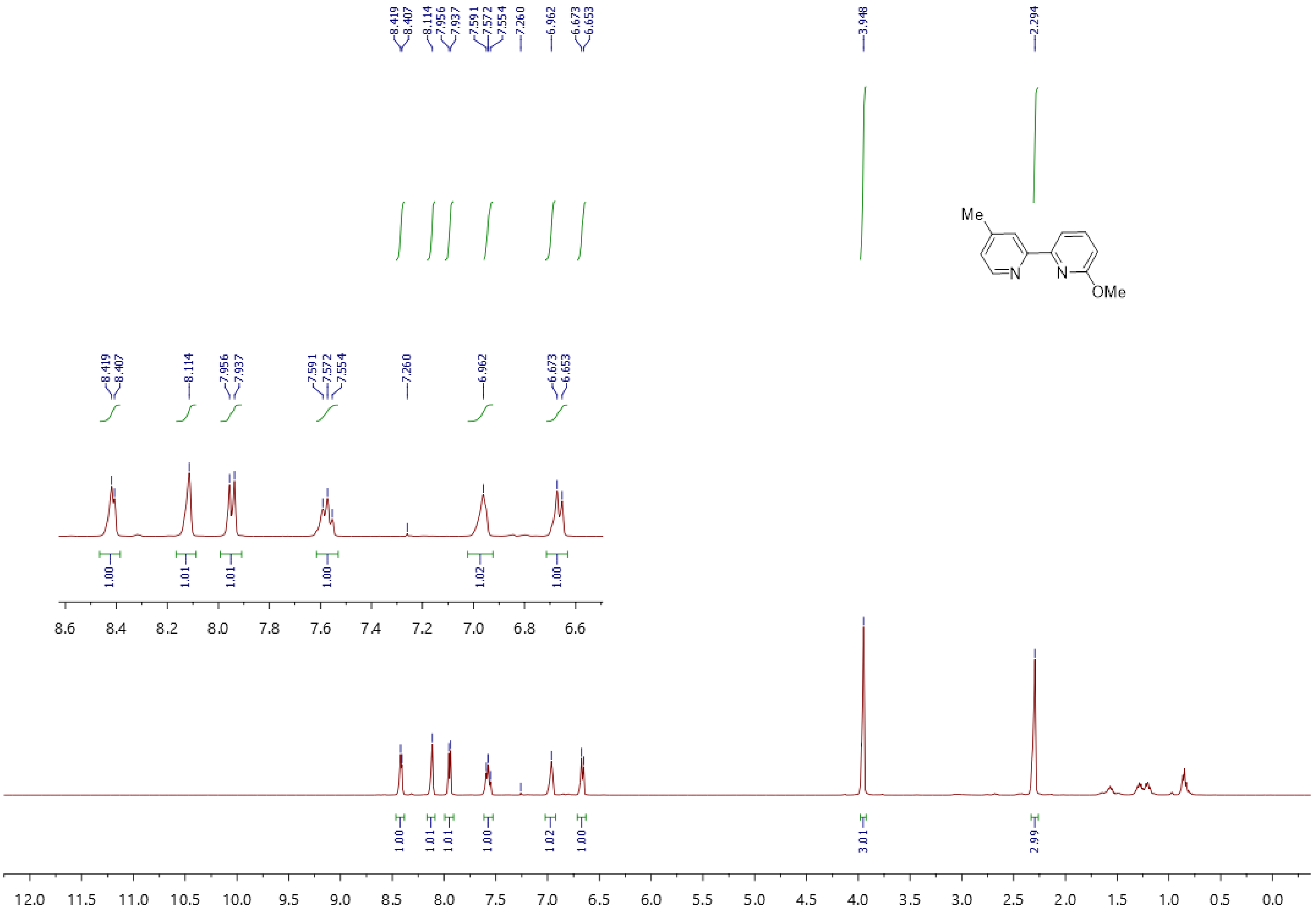

^{1}H-NMR spectra of **L8-I** (25 °C, 400 MHz, $CDCl_3$)

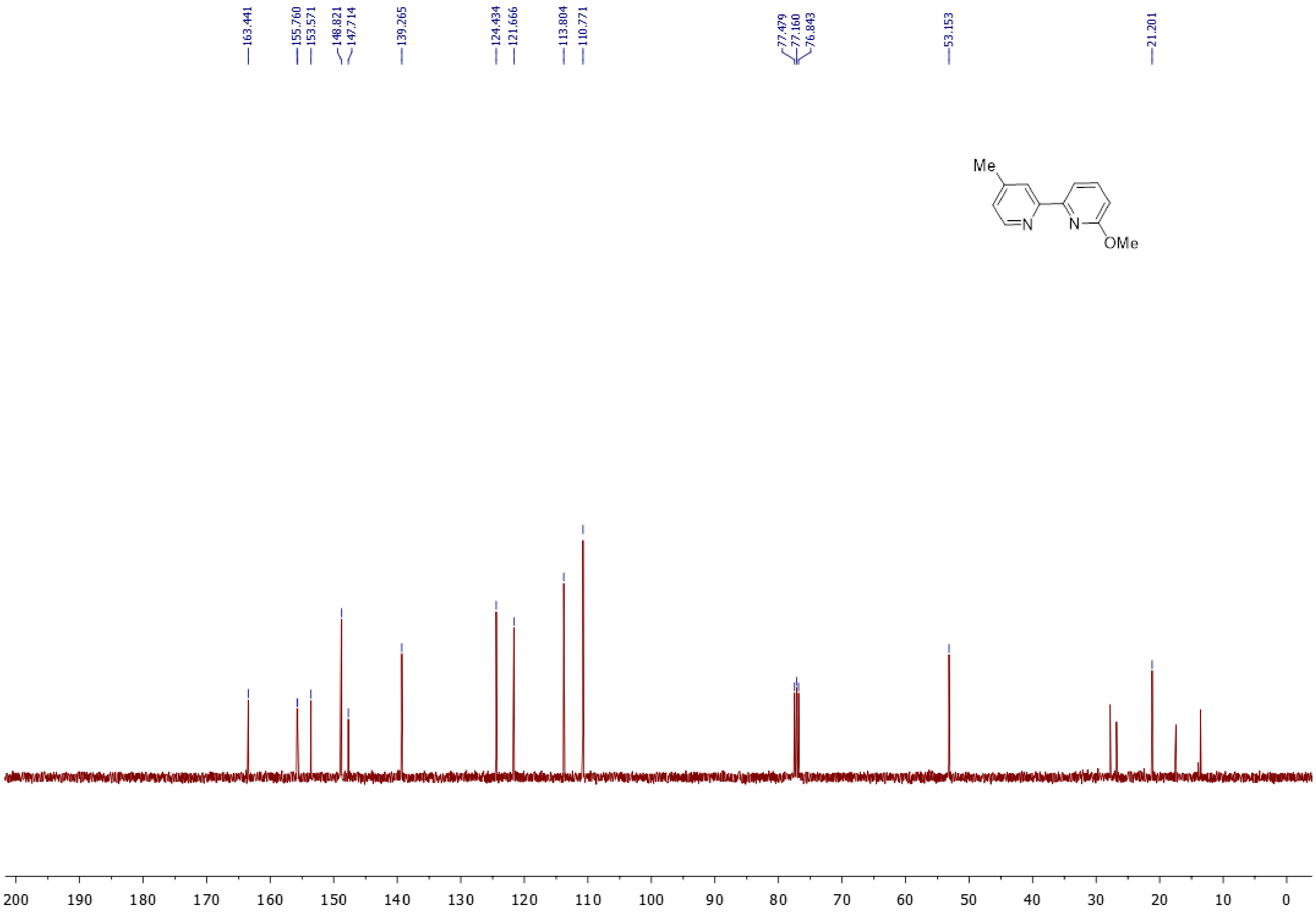

^{13}C-NMR spectra of **L8-I** (25 ^{o}C, 100 MHz, $CDCl_3$)

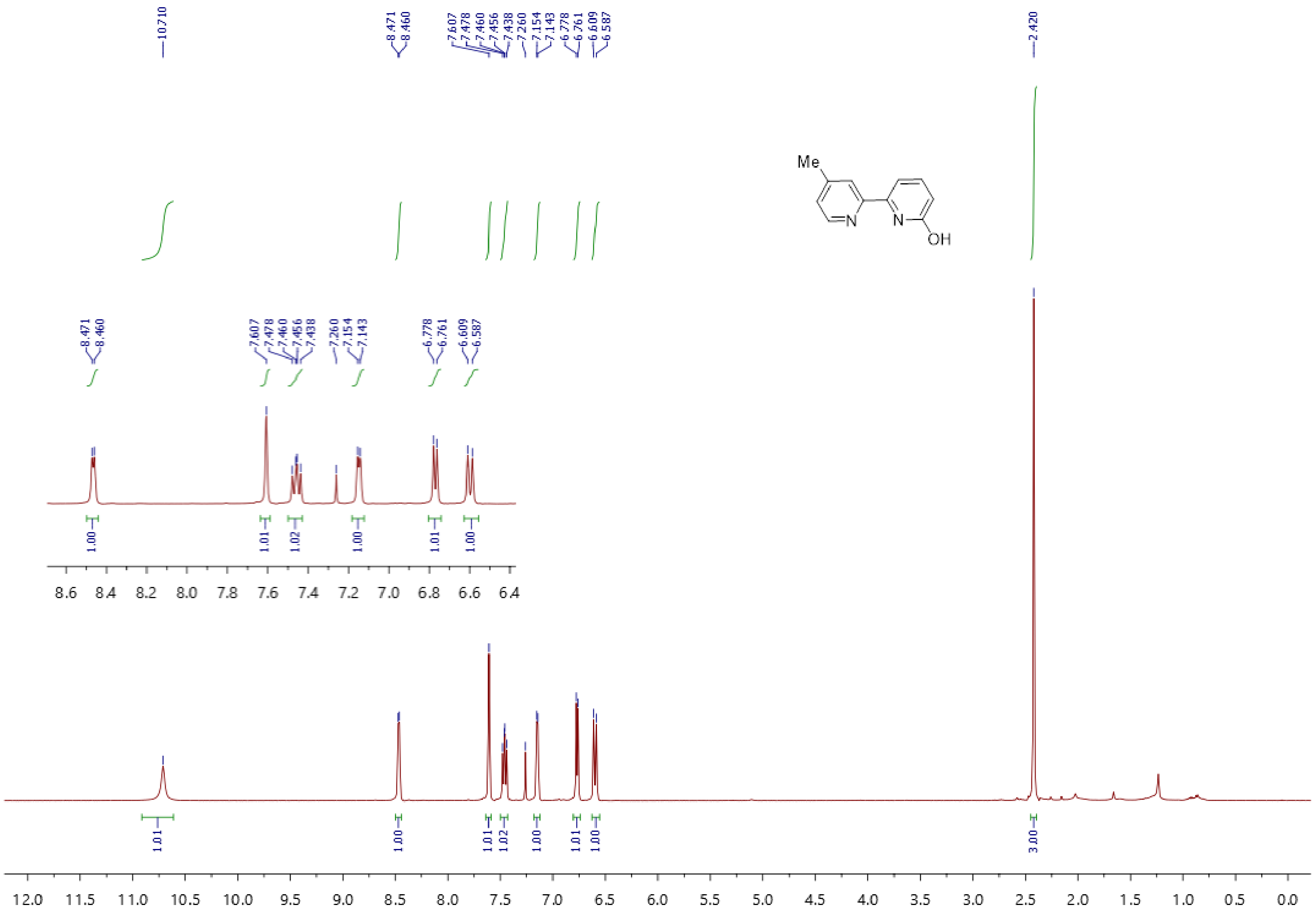

^{1}H-NMR spectra of **L8** (25 ºC, 400 MHz, $CDCl_3$)

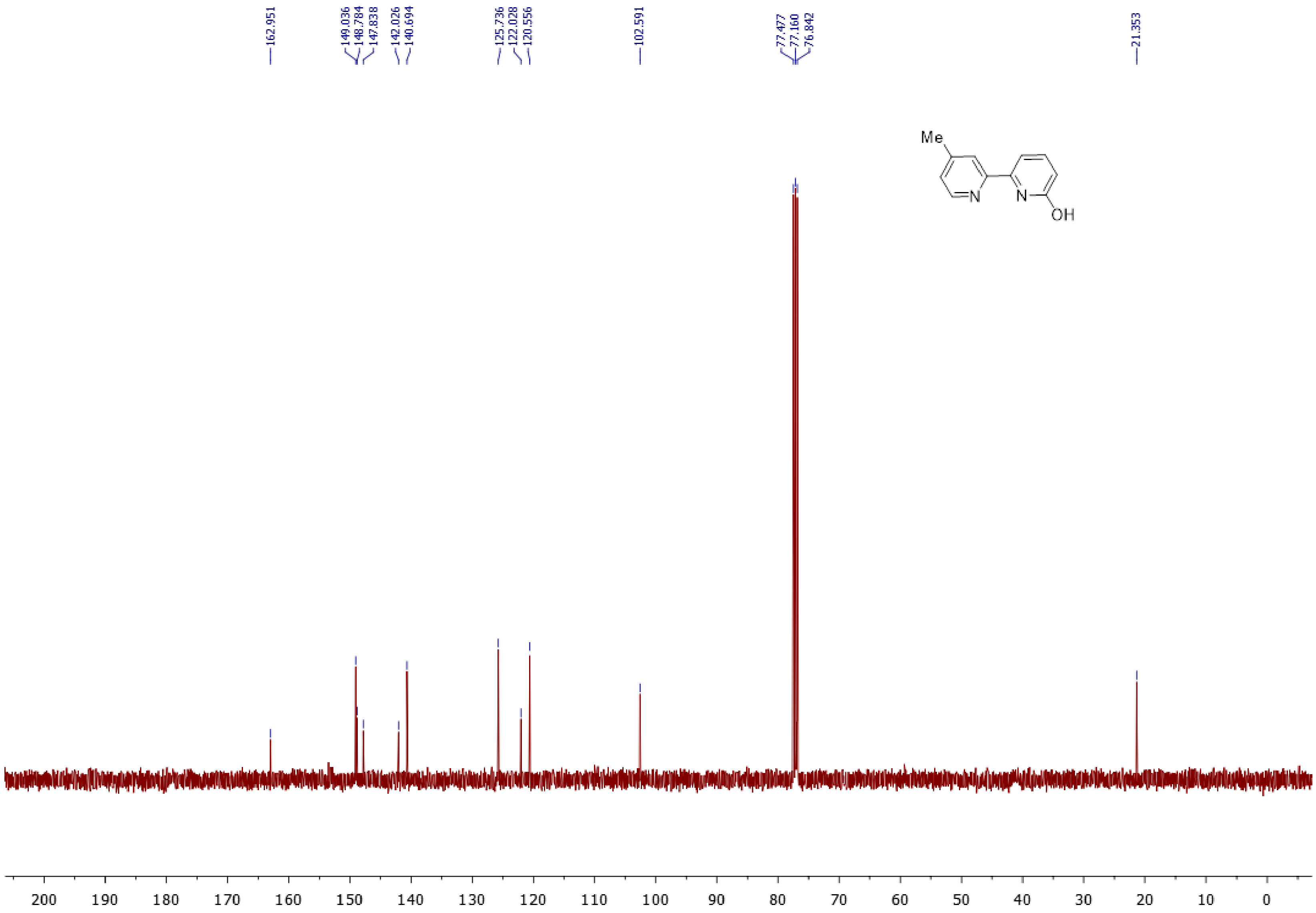

^{13}C-NMR spectra of **L8** (25 °C, 100 MHz, $CDCl_3$)

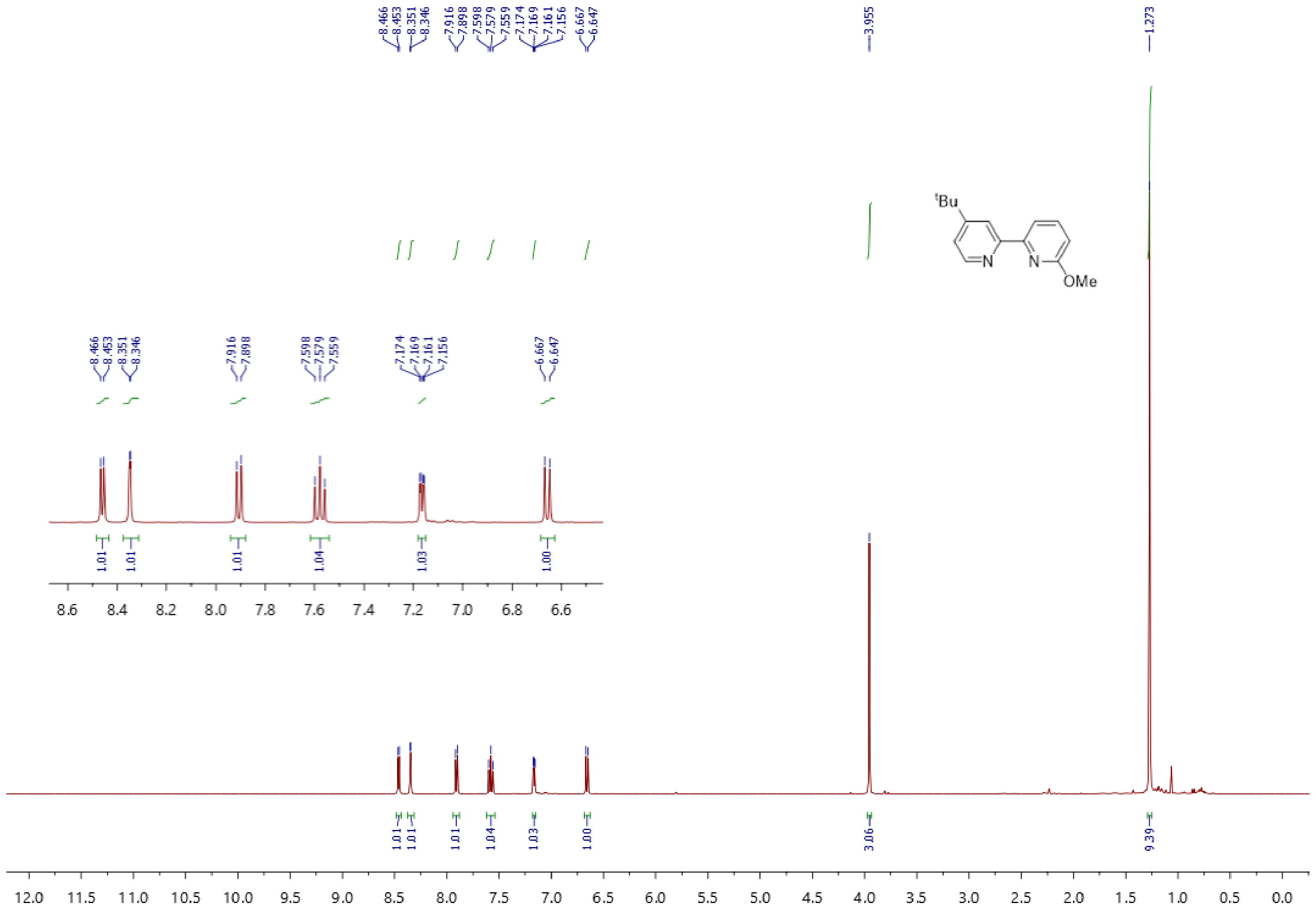

^{1}H-NMR spectra of **L9-I** (25 °C, 400 MHz, $CDCl_3$)

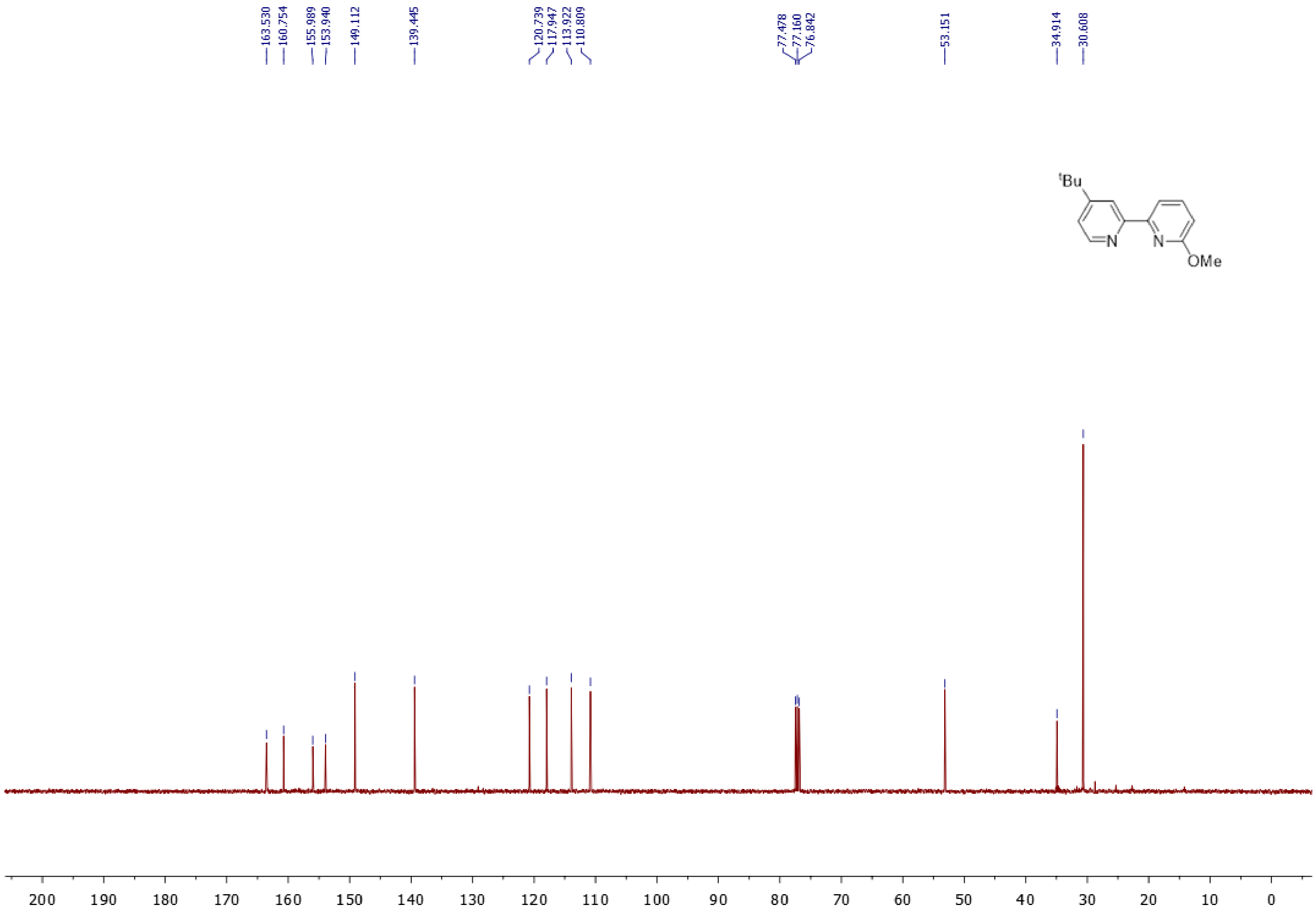

^{13}C-NMR spectra of **L9-I** (25 °C, 100 MHz, $CDCl_3$)

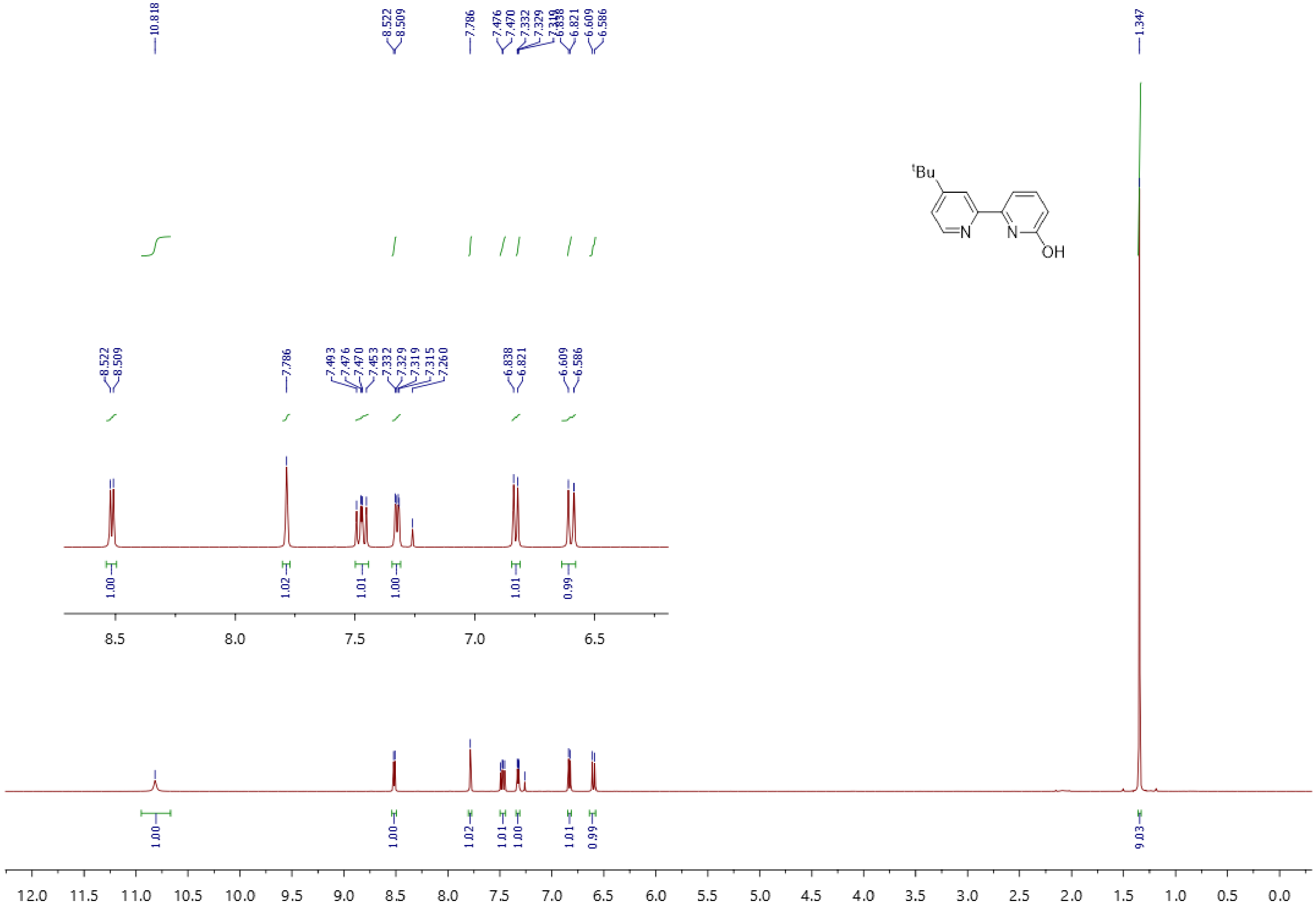

^{1}H-NMR spectra of **L9** (25 ^{o}C, 400 MHz, $CDCl_3$)

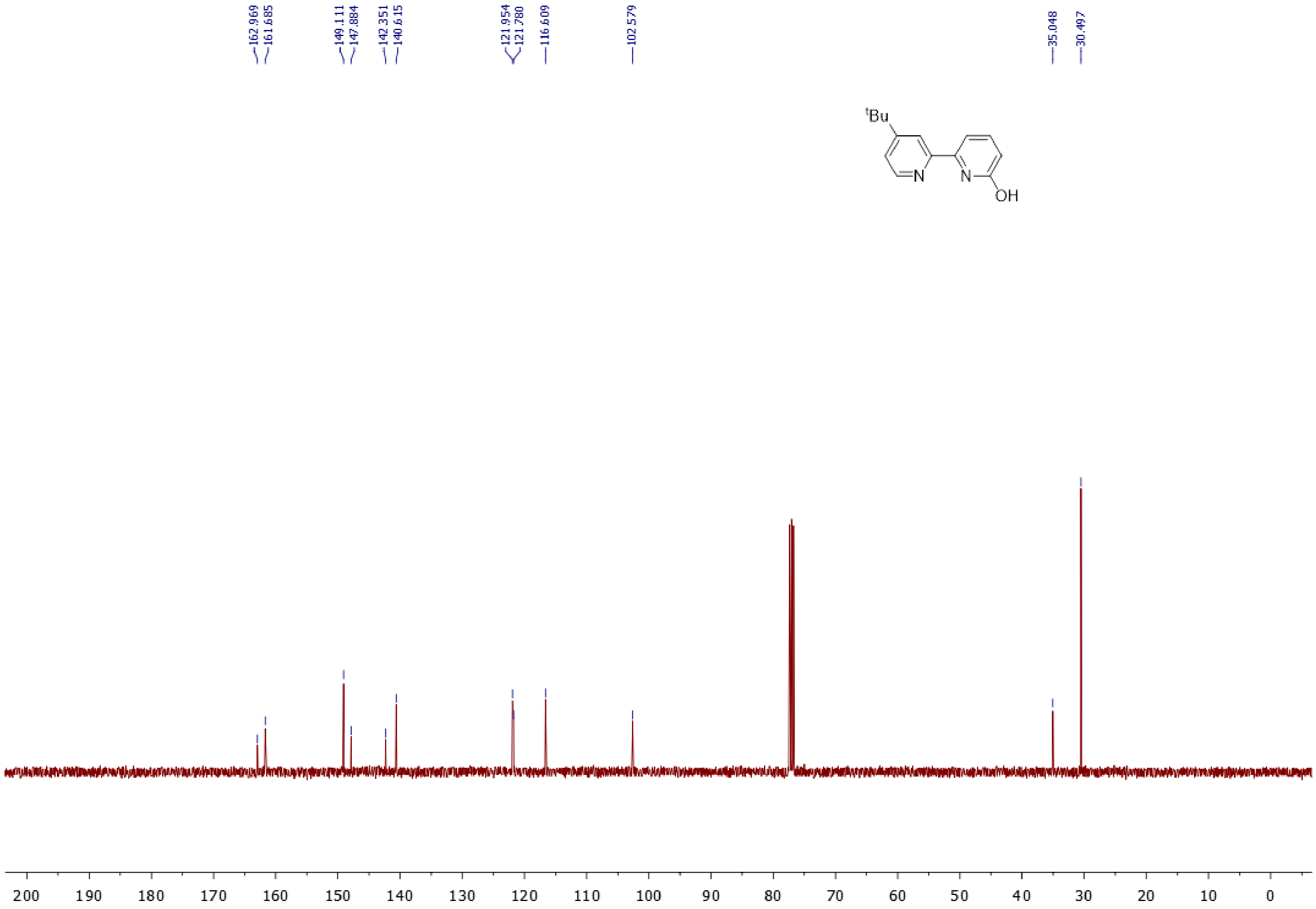

^{13}C-NMR spectra of **L9** (25 °C, 100 MHz, $CDCl_3$)

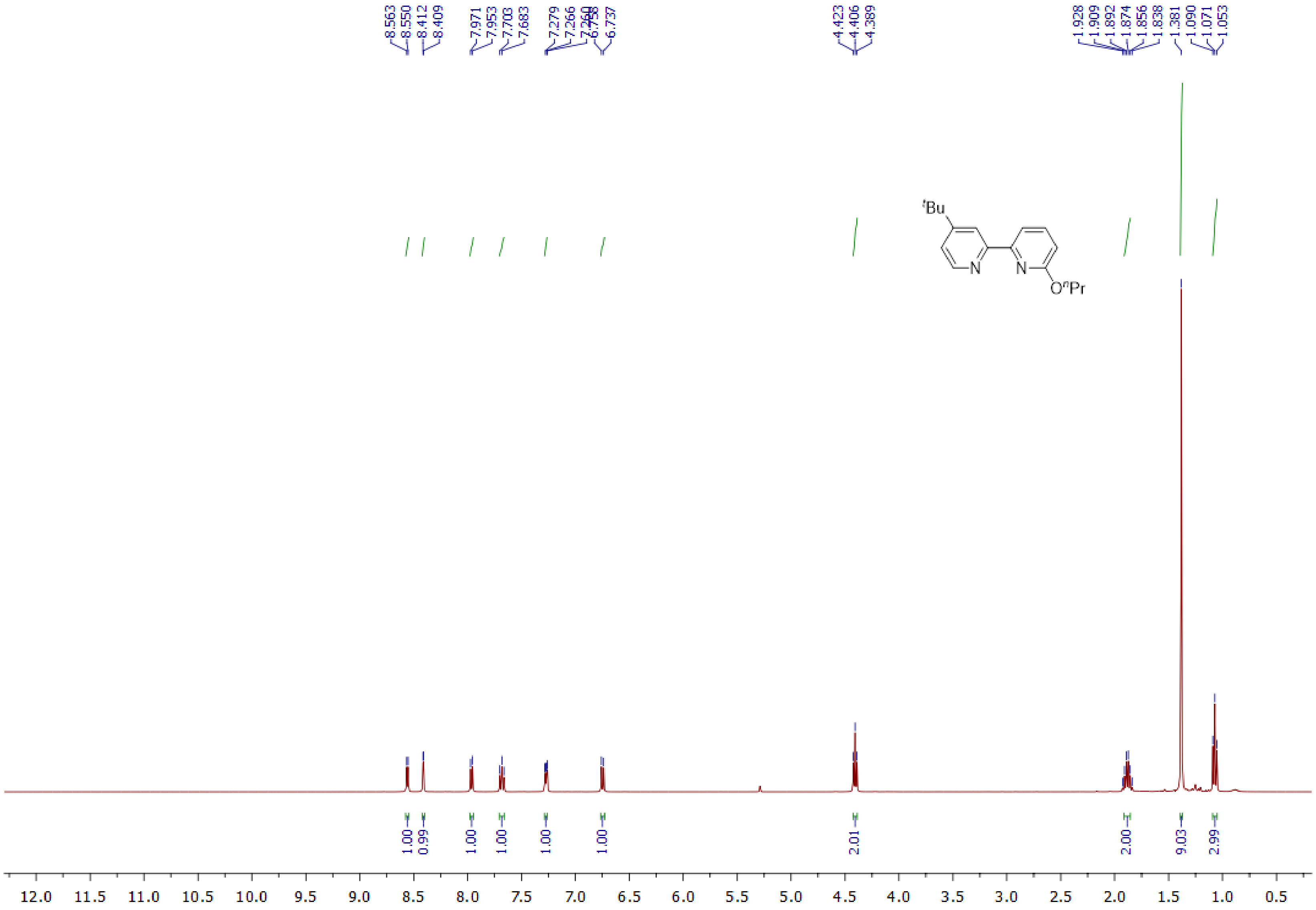

^{1}H-NMR spectra of **L14** (25 °C, 400 MHz, $CDCl_3$)

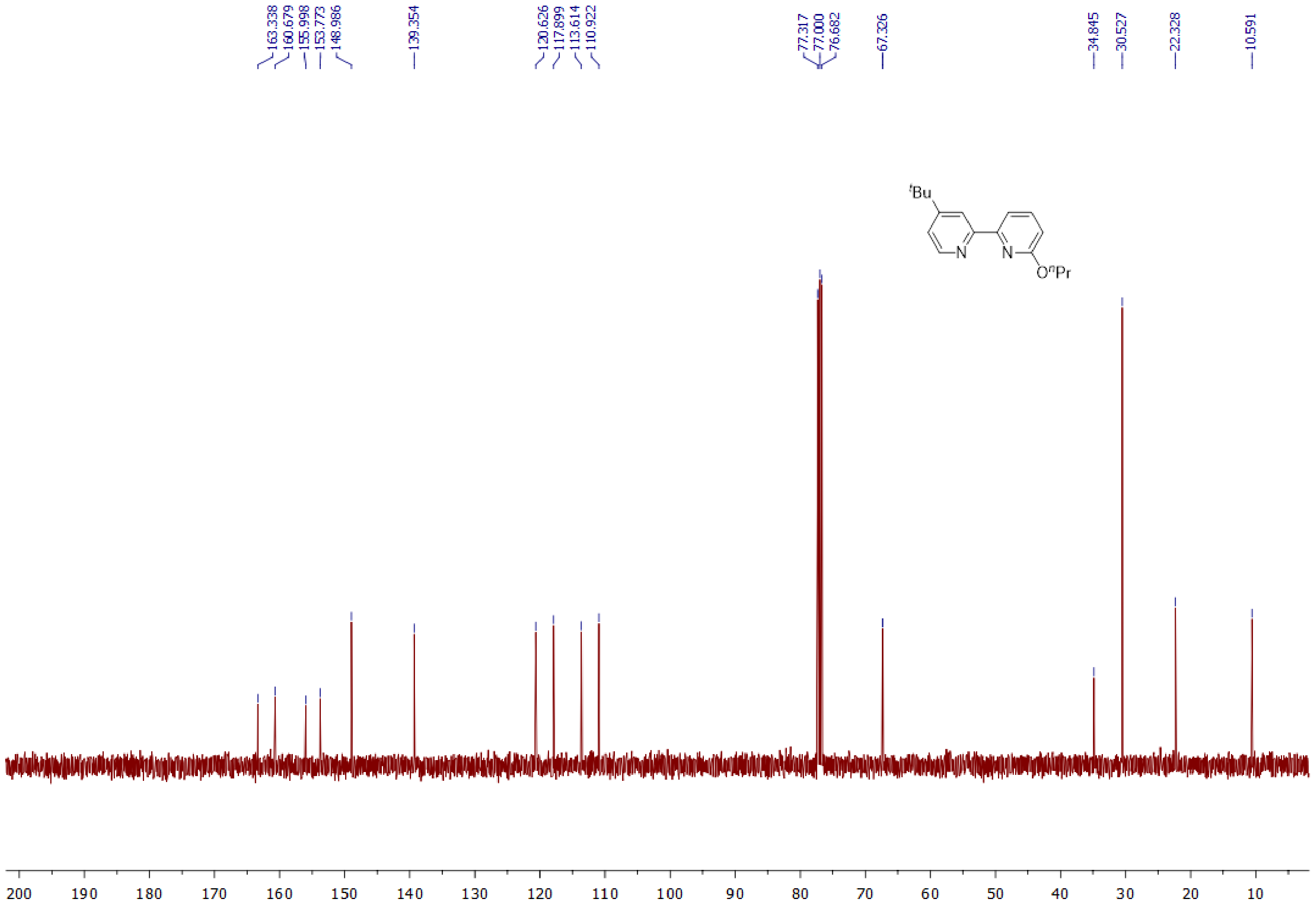

^{13}C-NMR spectra of **L14** (25 °C, 100 MHz, $CDCl_3$)

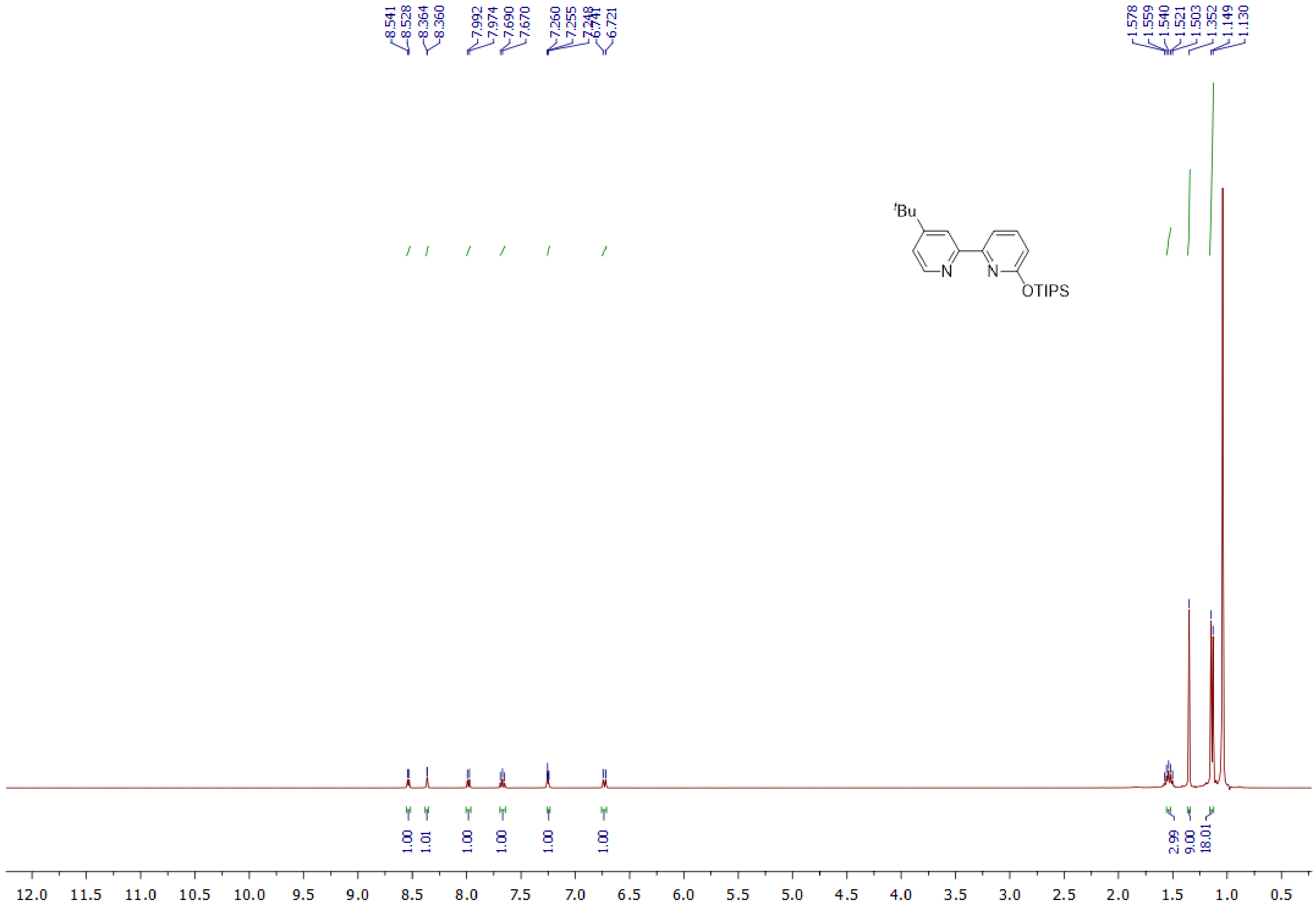

^{1}H-NMR spectra of **L15** (25 ºC, 400 MHz, $CDCl_3$)

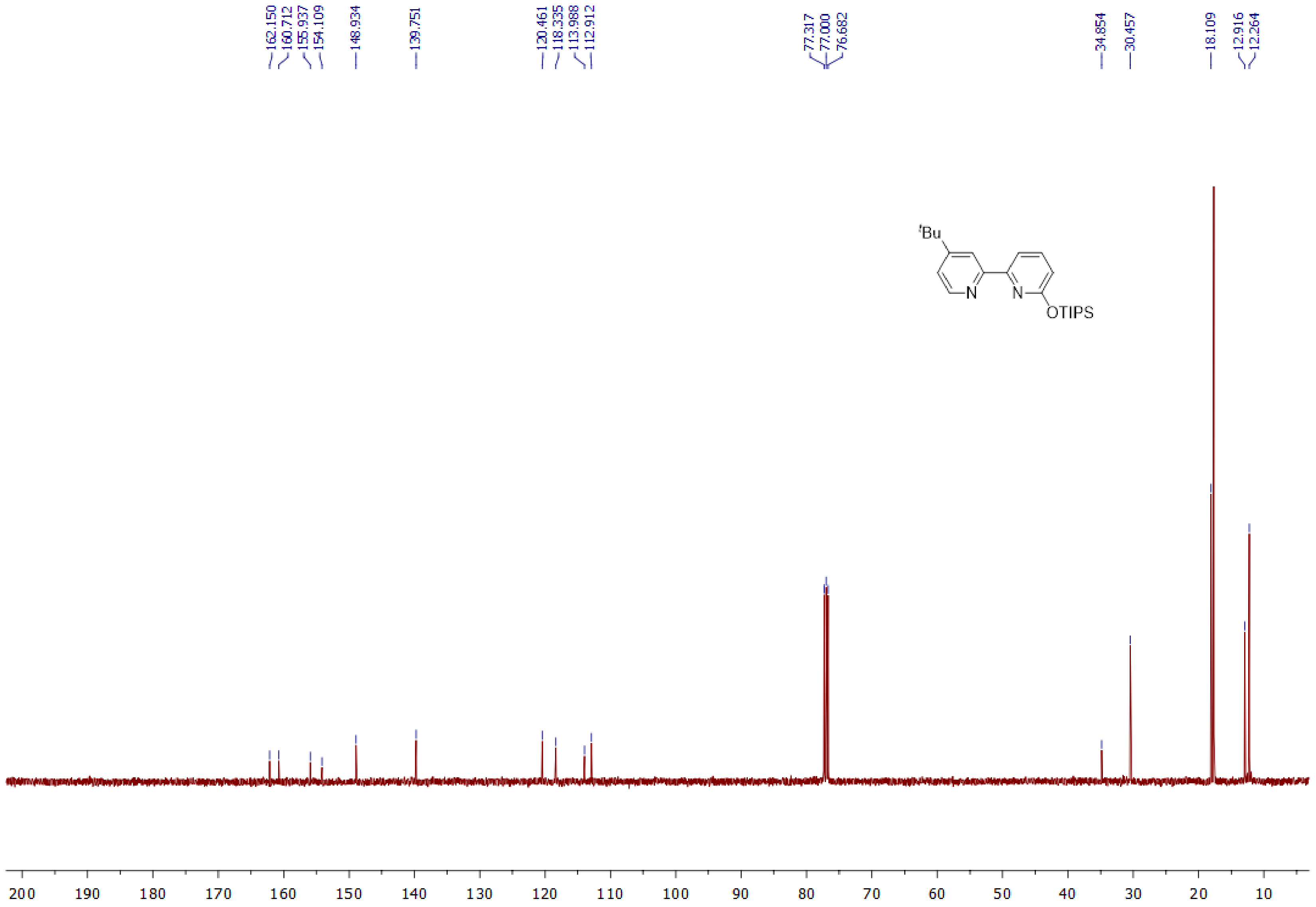

^{13}C-NMR spectra of **L15** (25 ºC, 100 MHz, $CDCl_3$)

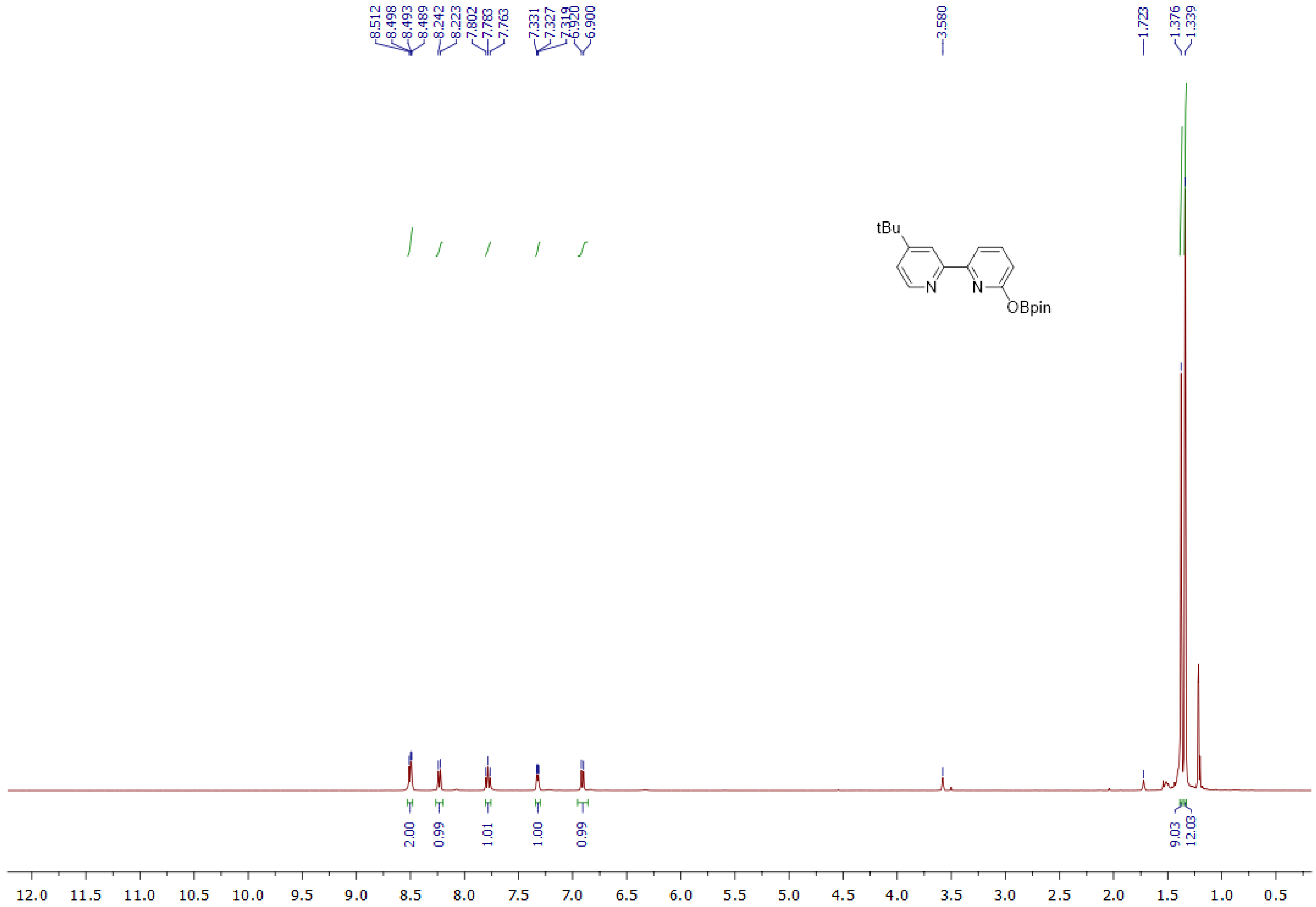

^{1}H-NMR spectra of **L10** (25 ^{o}C, 400 MHz, THF-d8)

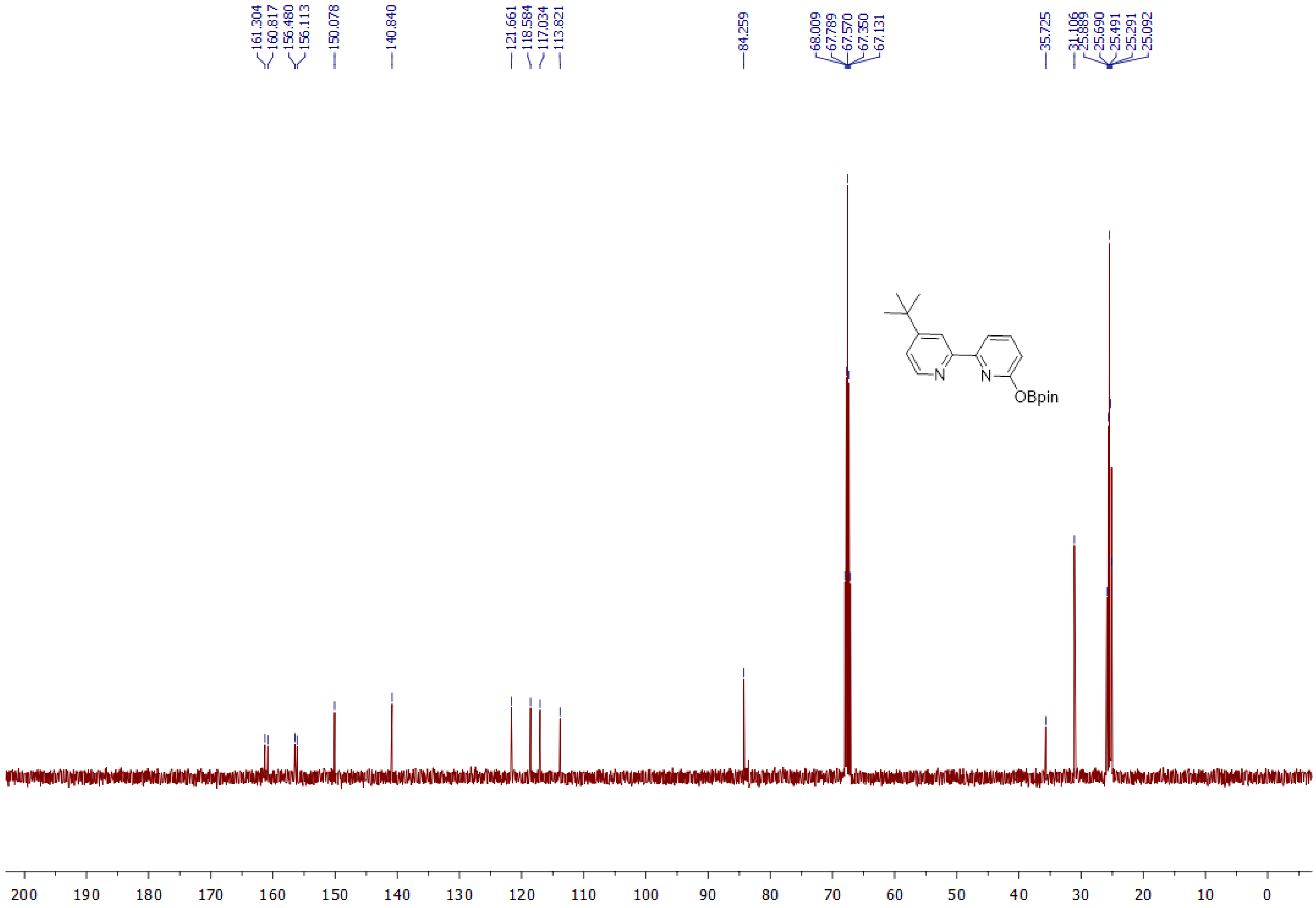

^{13}C-NMR spectra of **L10** (25 °C, 400 MHz, THF-d8)

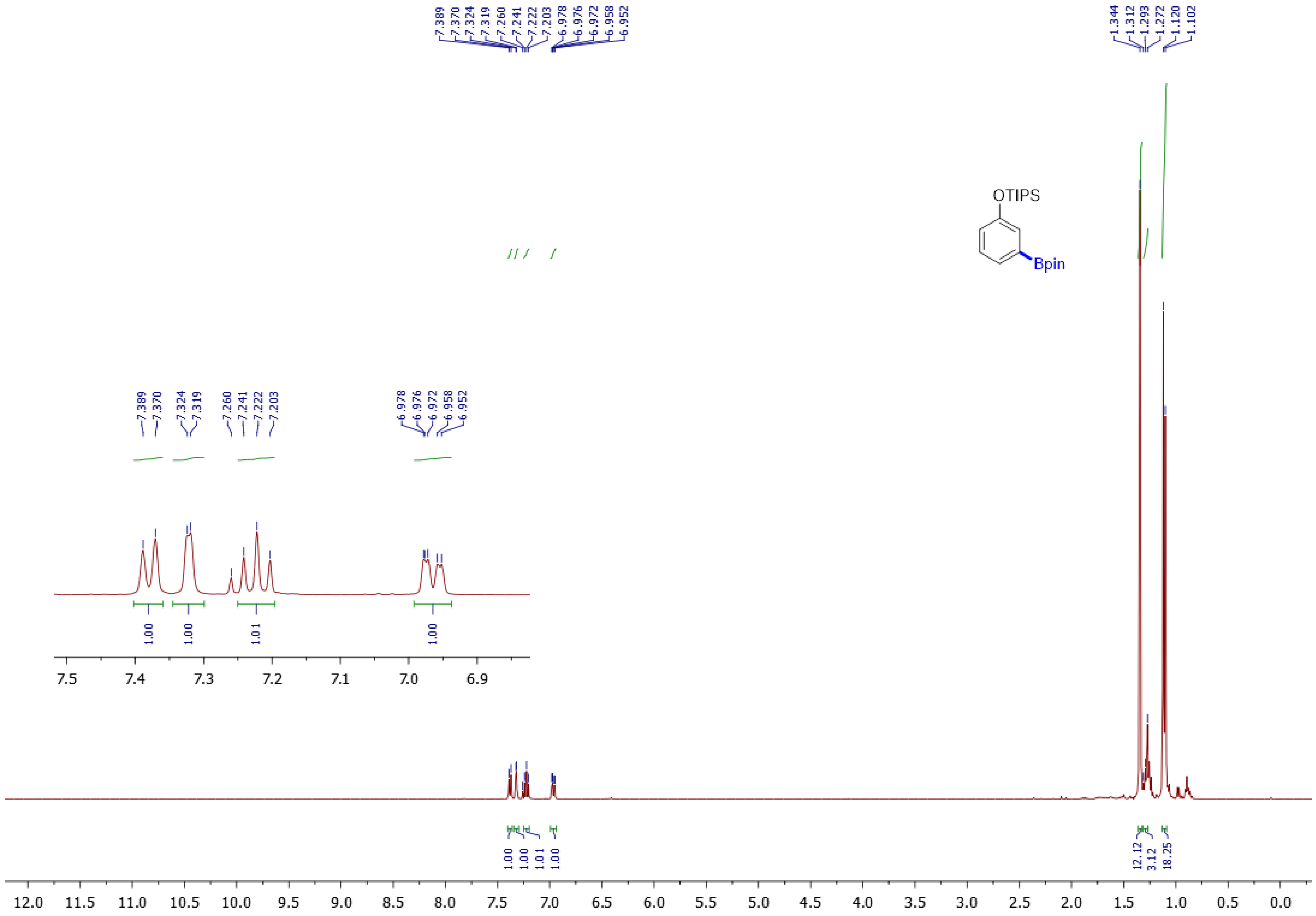

^{1}H-NMR spectra of **2l** (25 °C, 400 MHz, $CDCl_3$)

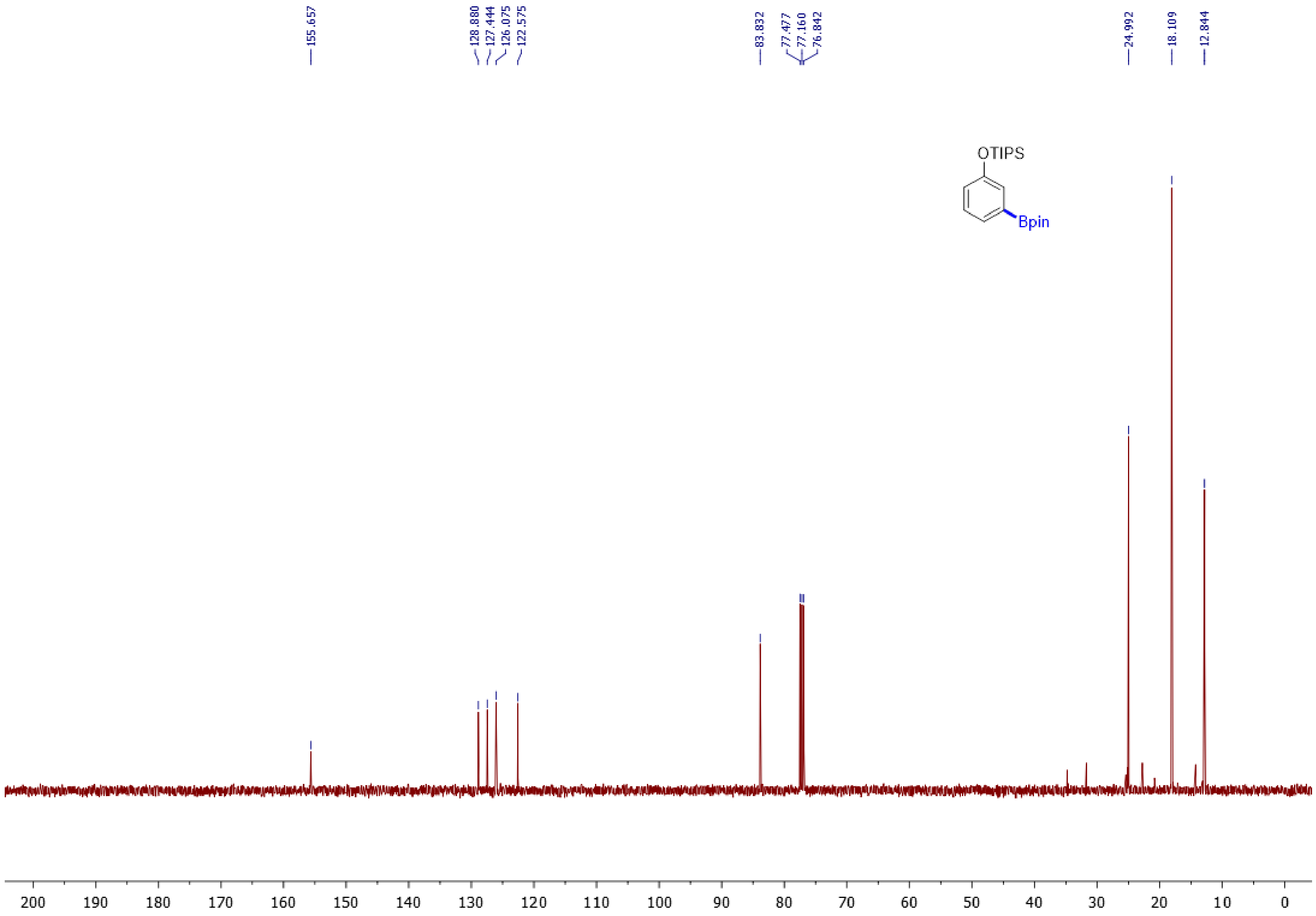

^{13}C-NMR spectra of **2l** (25 ºC, 100 MHz, $CDCl_3$)

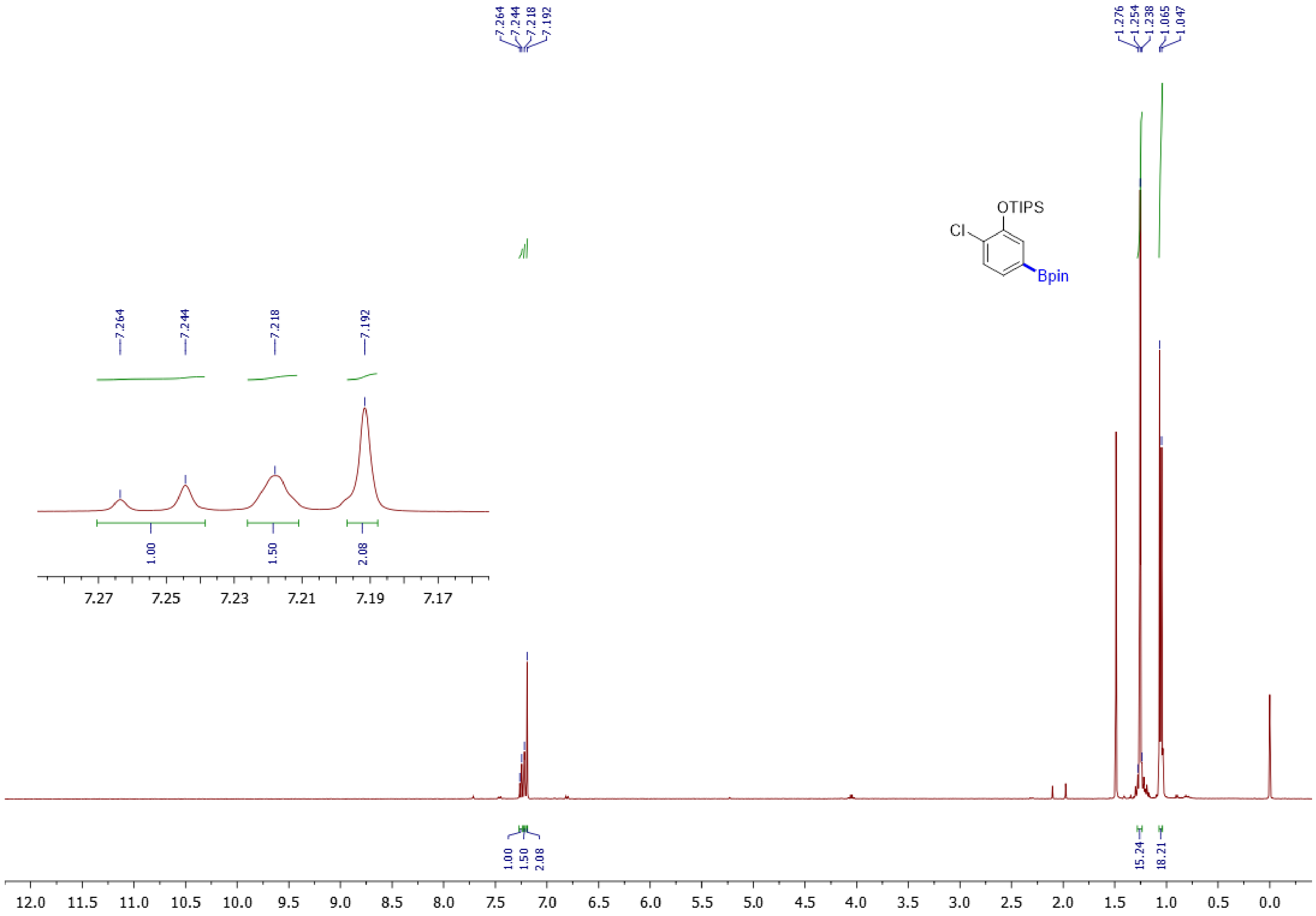

^{1}H-NMR spectra of **5a** (25 ^{o}C, 400 MHz, $CDCl_3$)

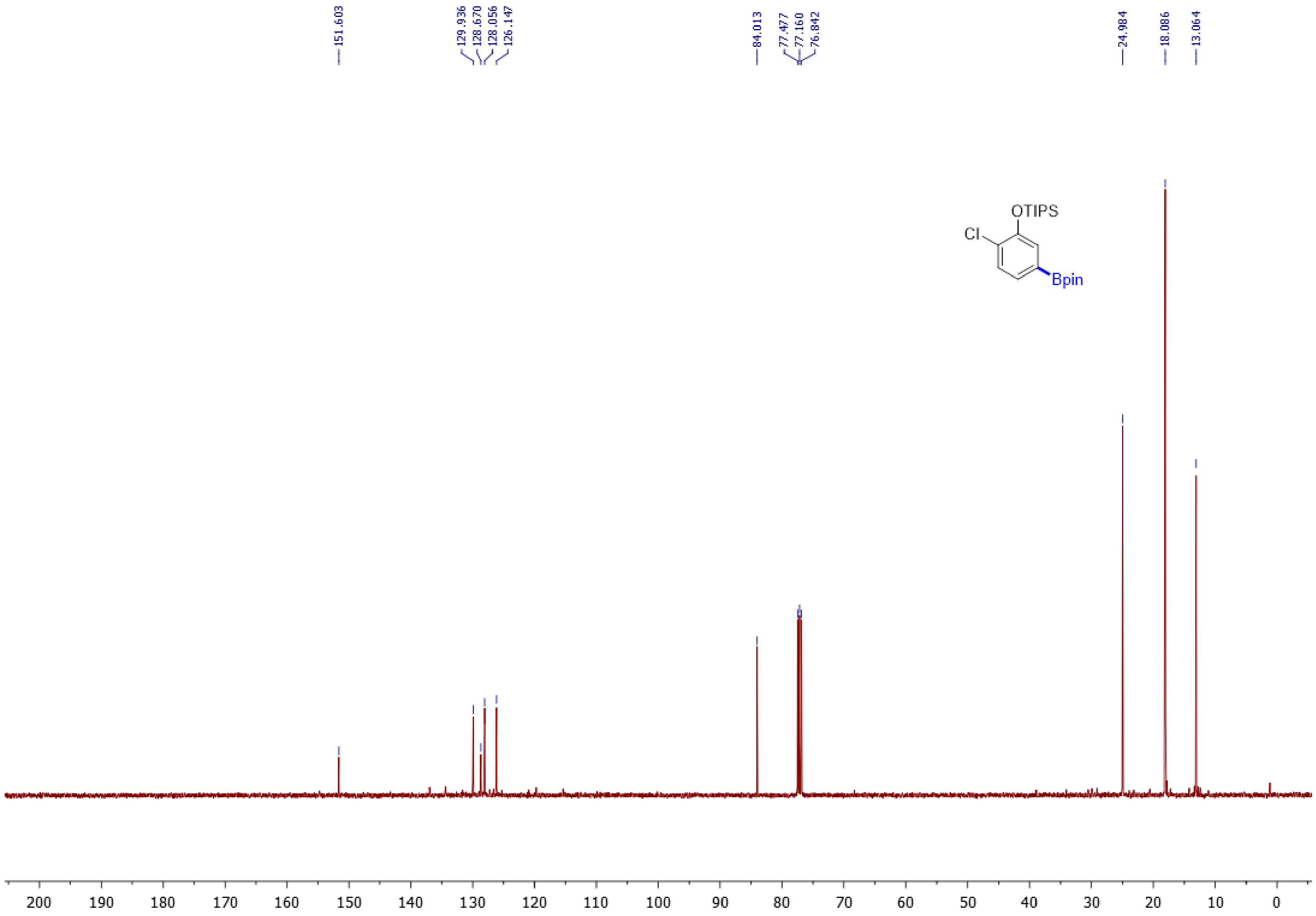

^{13}C-NMR spectra of **5a** (25 ^{o}C, 100 MHz, $CDCl_3$)

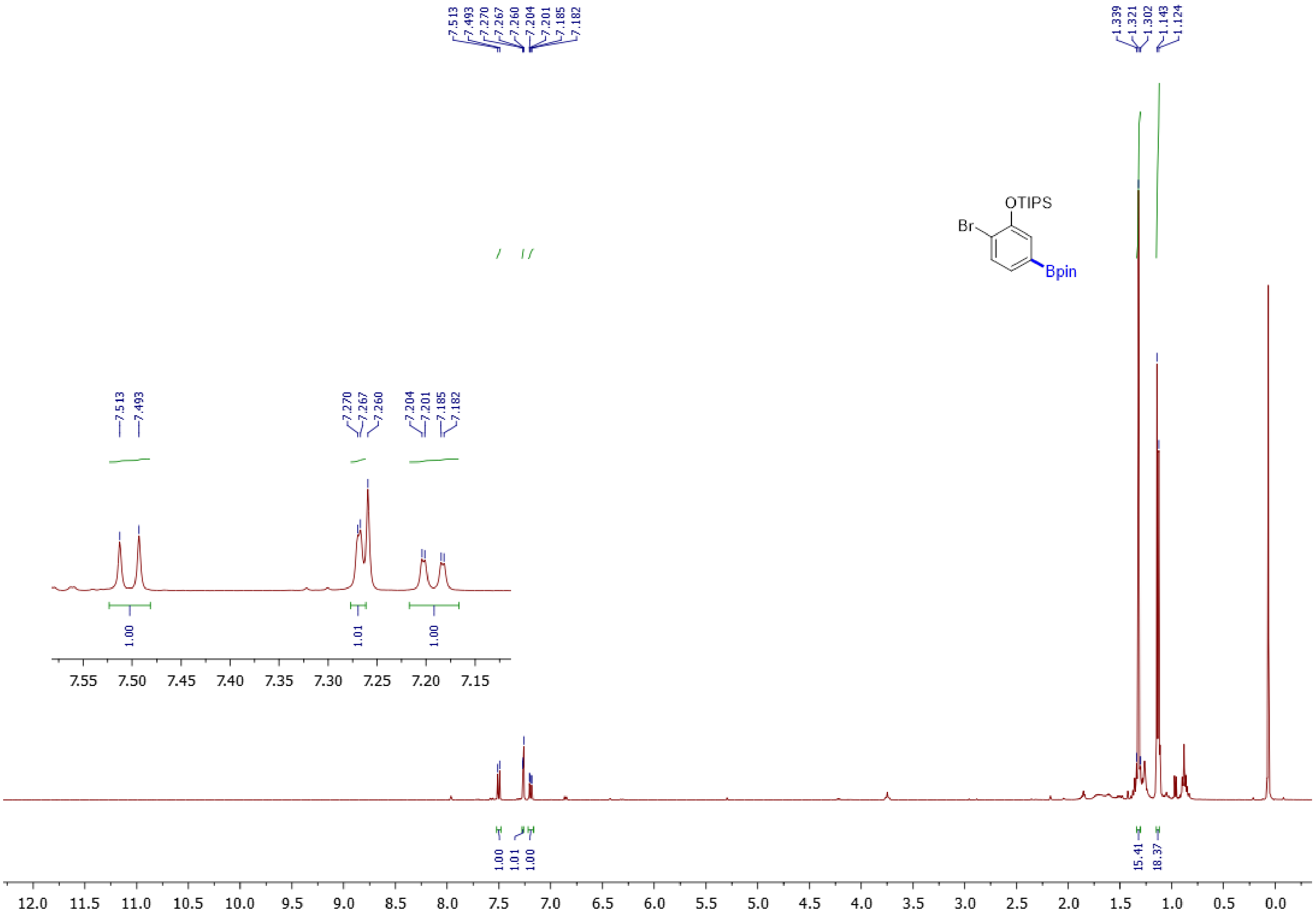

^{1}H-NMR spectra of **5b** (25 ^{o}C, 400 MHz, $CDCl_3$)

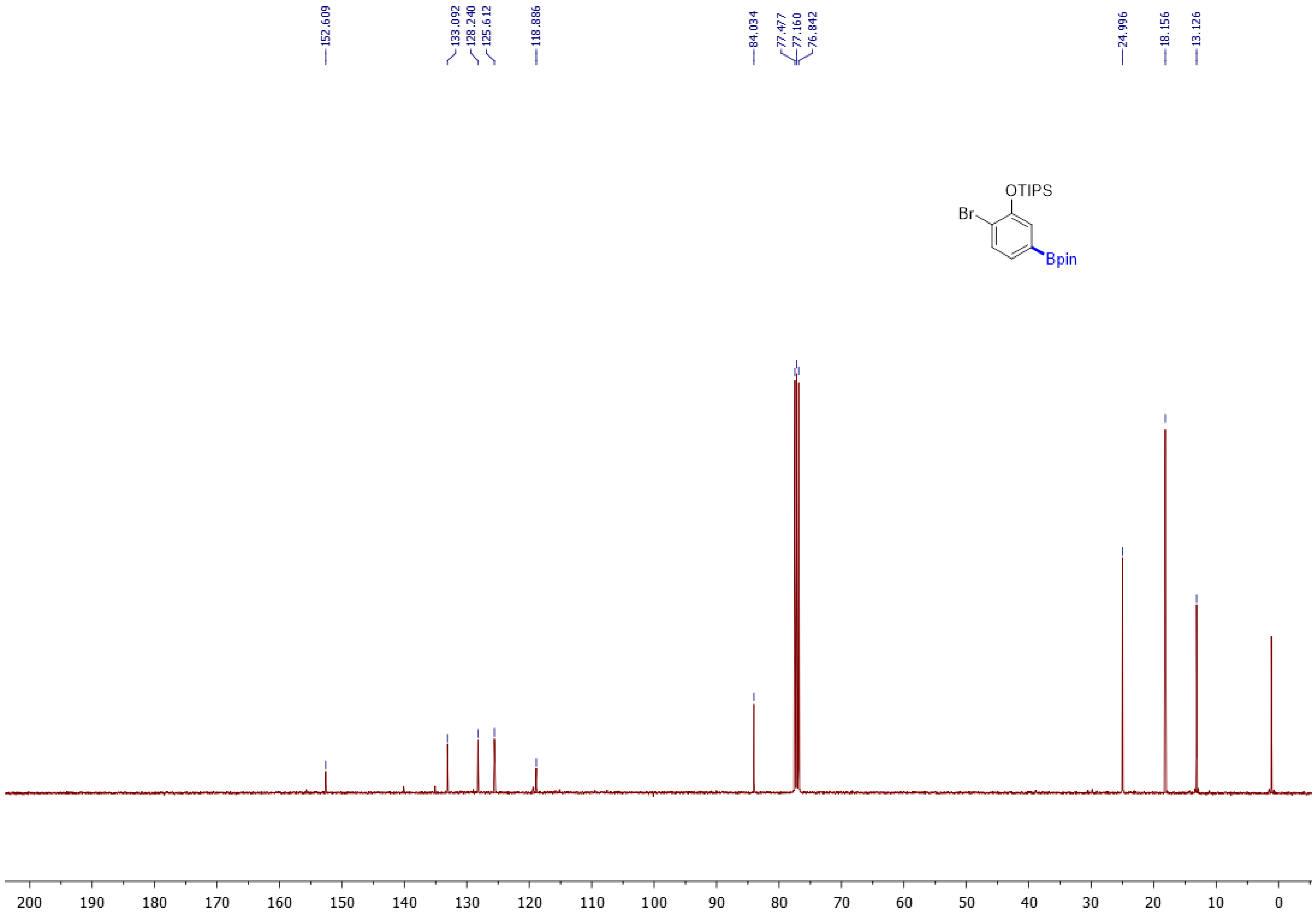

^{13}C-NMR spectra of **5b** (25 ºC, 100 MHz, $CDCl_3$)

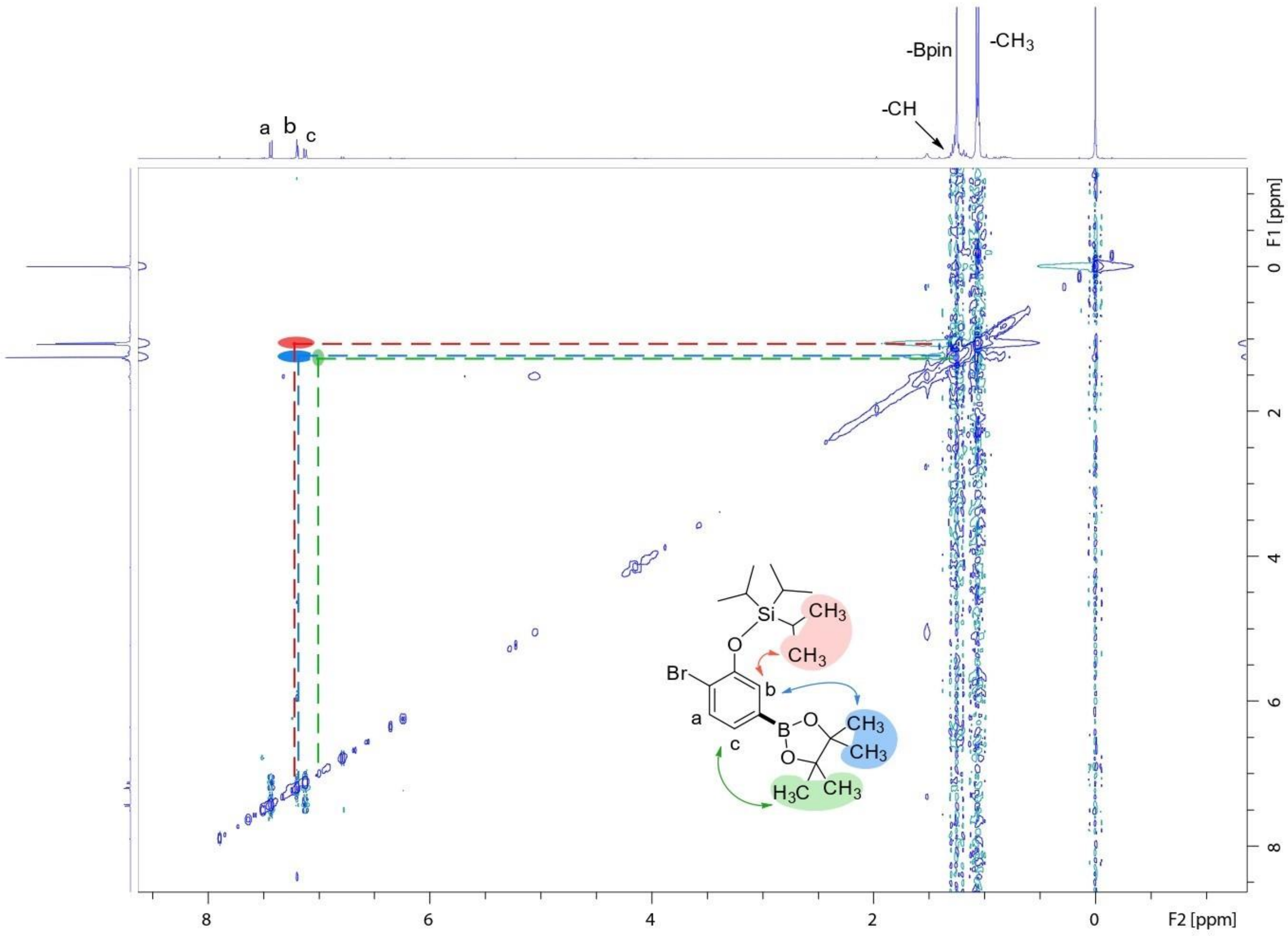

NOESY-NMR spectra of **5b** (25 ^{o}C, 100 MHz, $CDCl_3$)

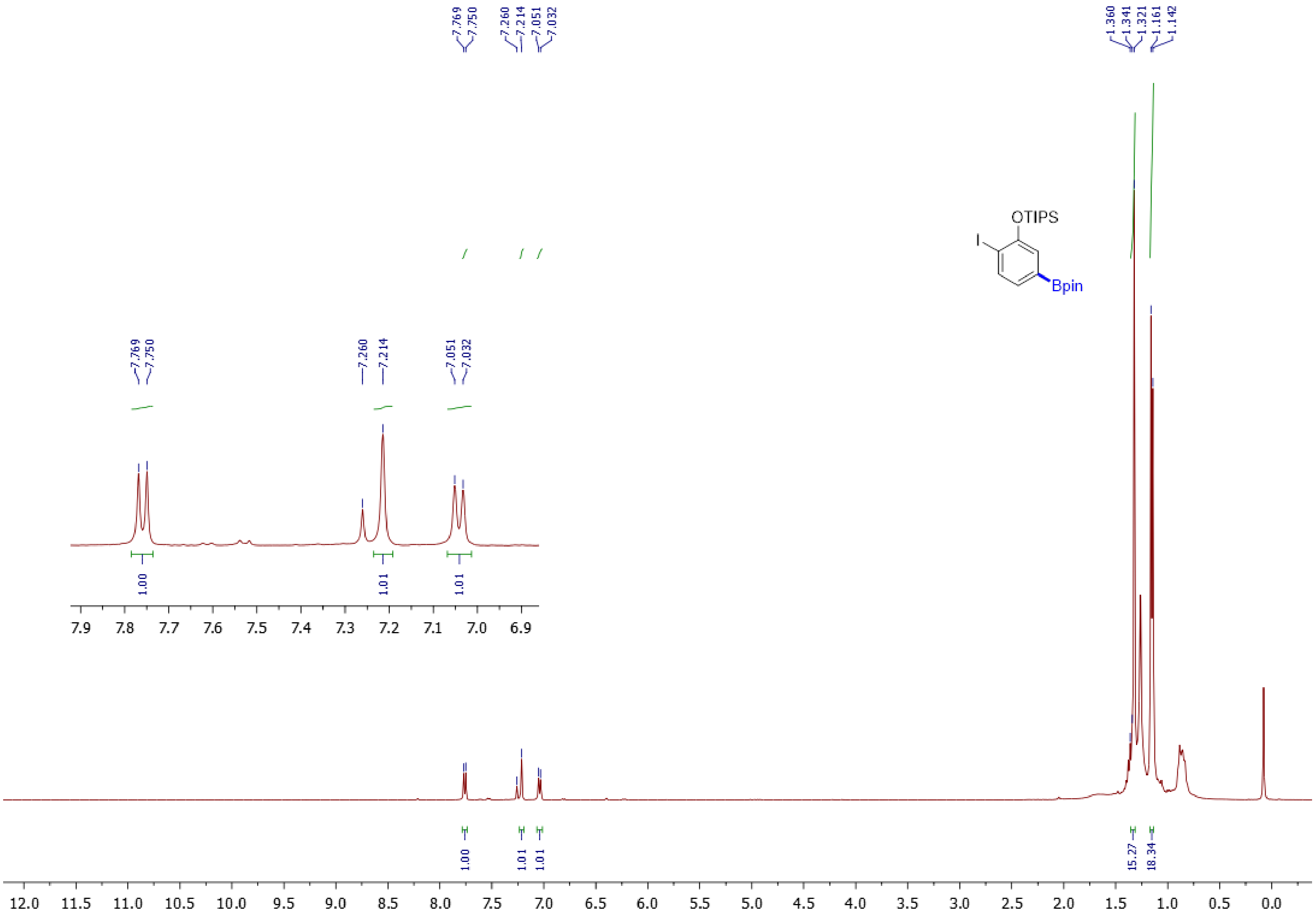

^{1}H-NMR spectra of **5c** (25 °C, 400 MHz, $CDCl_3$)

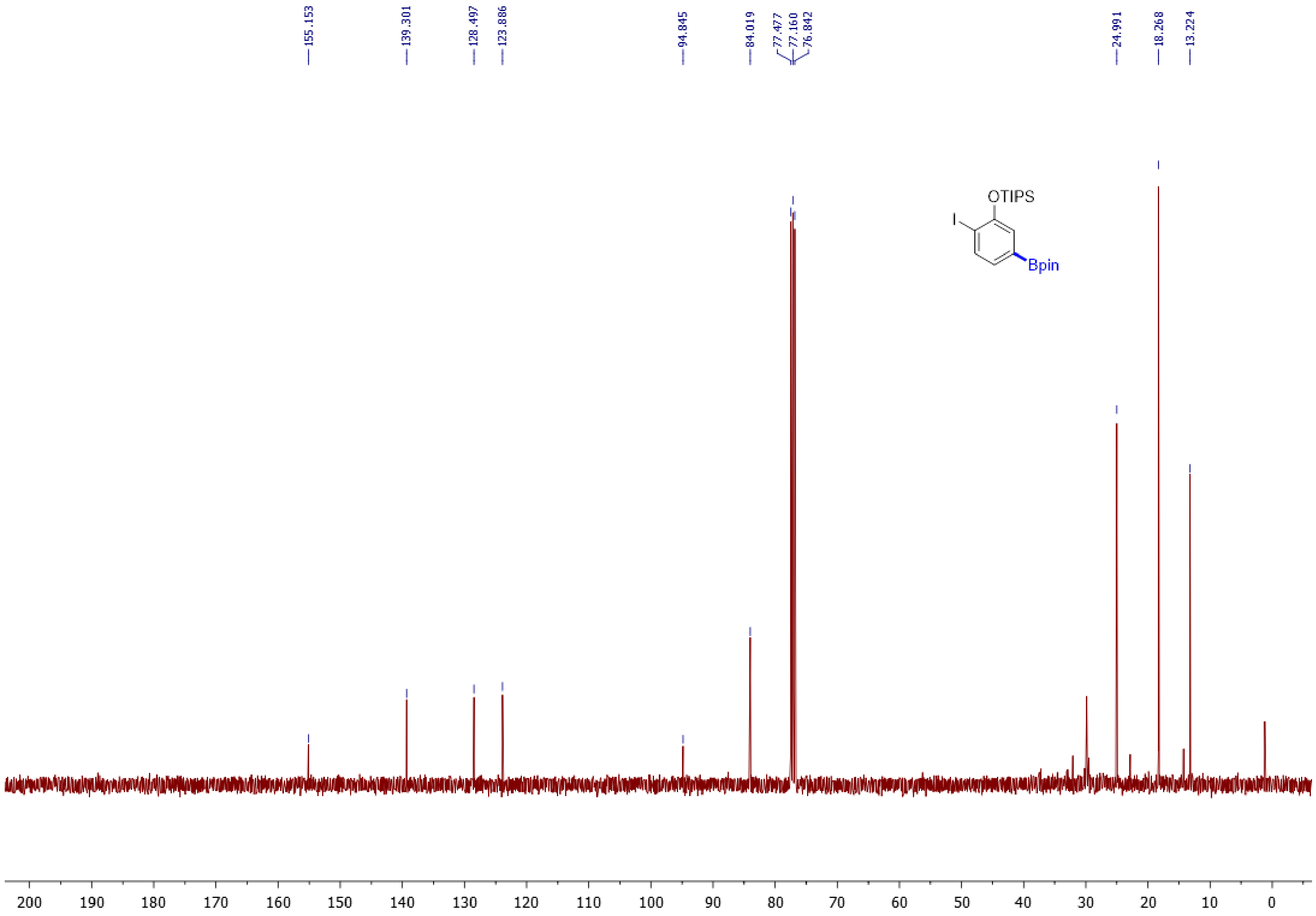

^{13}C-NMR spectra of **5c** (25 ºC, 100 MHz, $CDCl_3$)

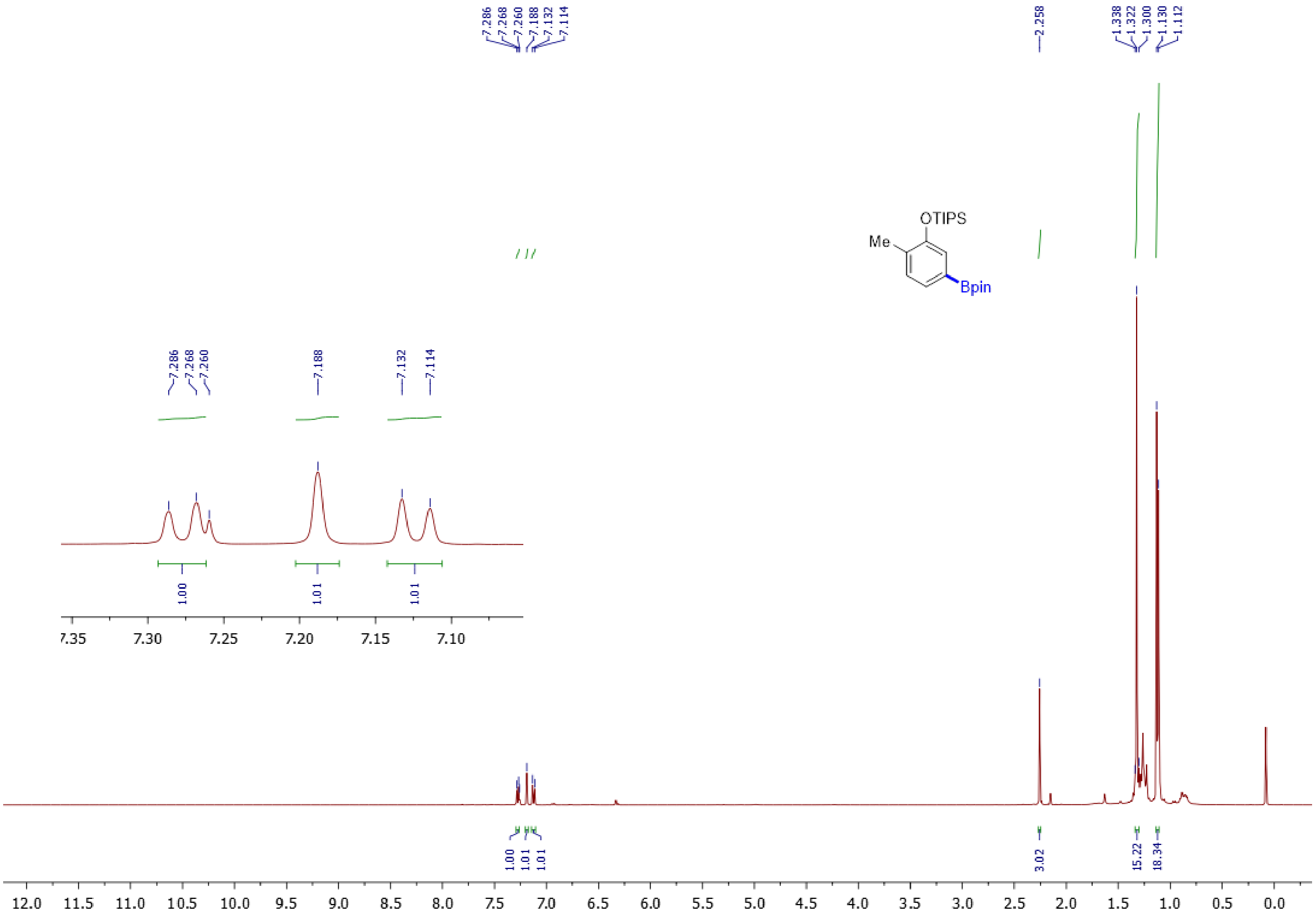

^{1}H-NMR spectra of **5d** (25 °C, 400 MHz, $CDCl_3$)

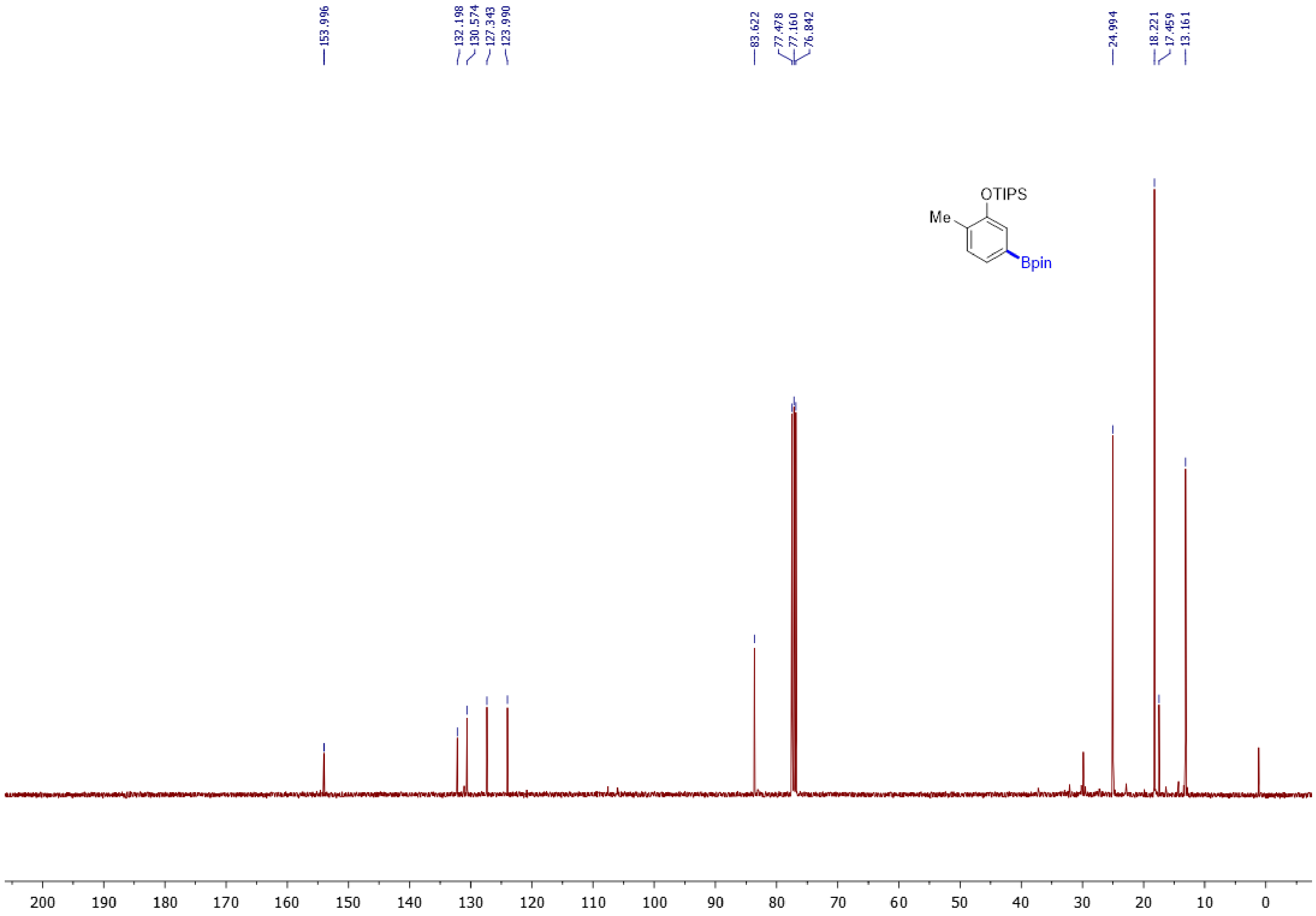

^{13}C-NMR spectra of **5d** (25 ^{o}C, 100 MHz, $CDCl_3$)

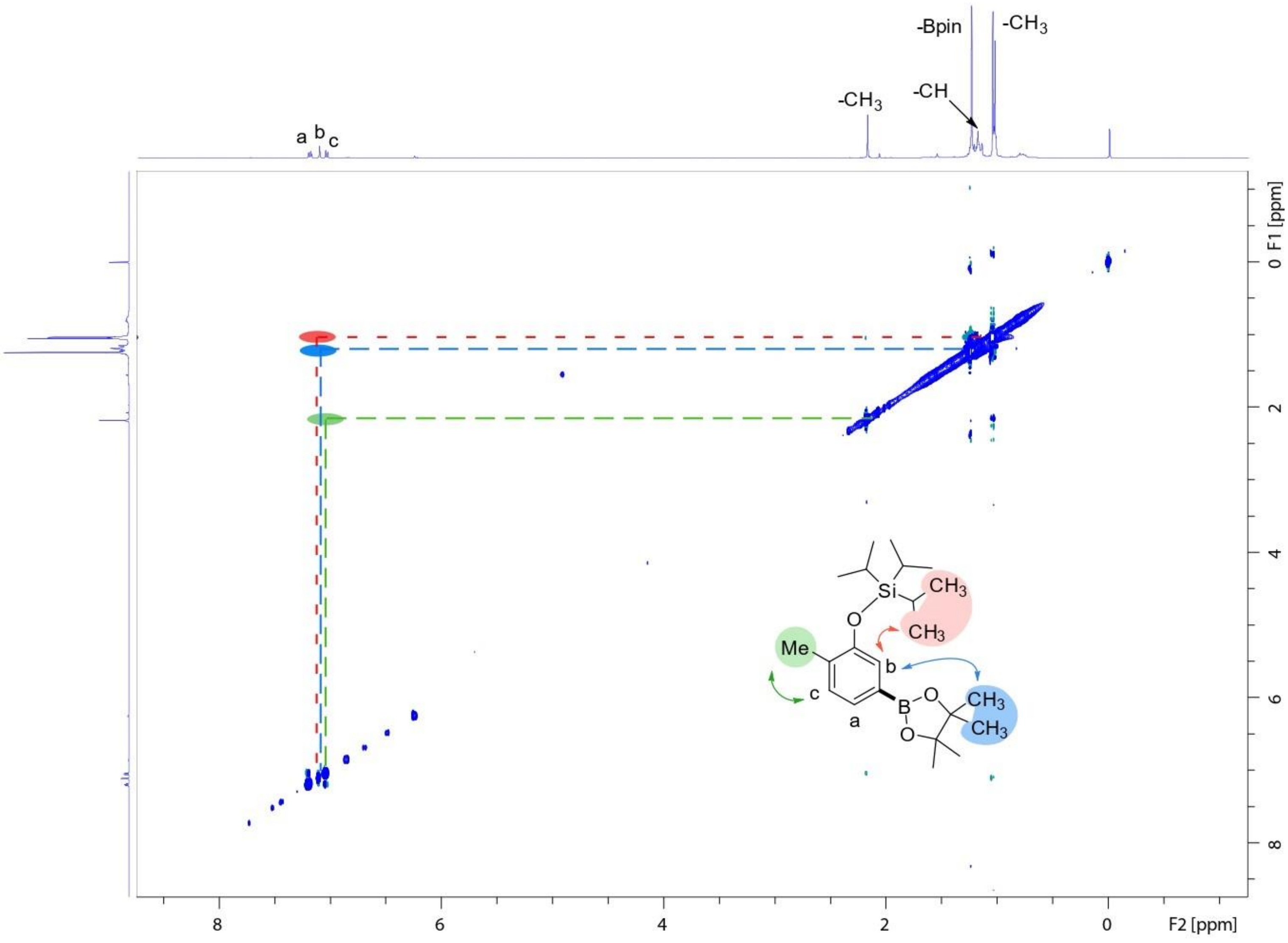

NOESY-NMR spectra of **5d** (25 ºC, 100 MHz, $CDCl_3$)

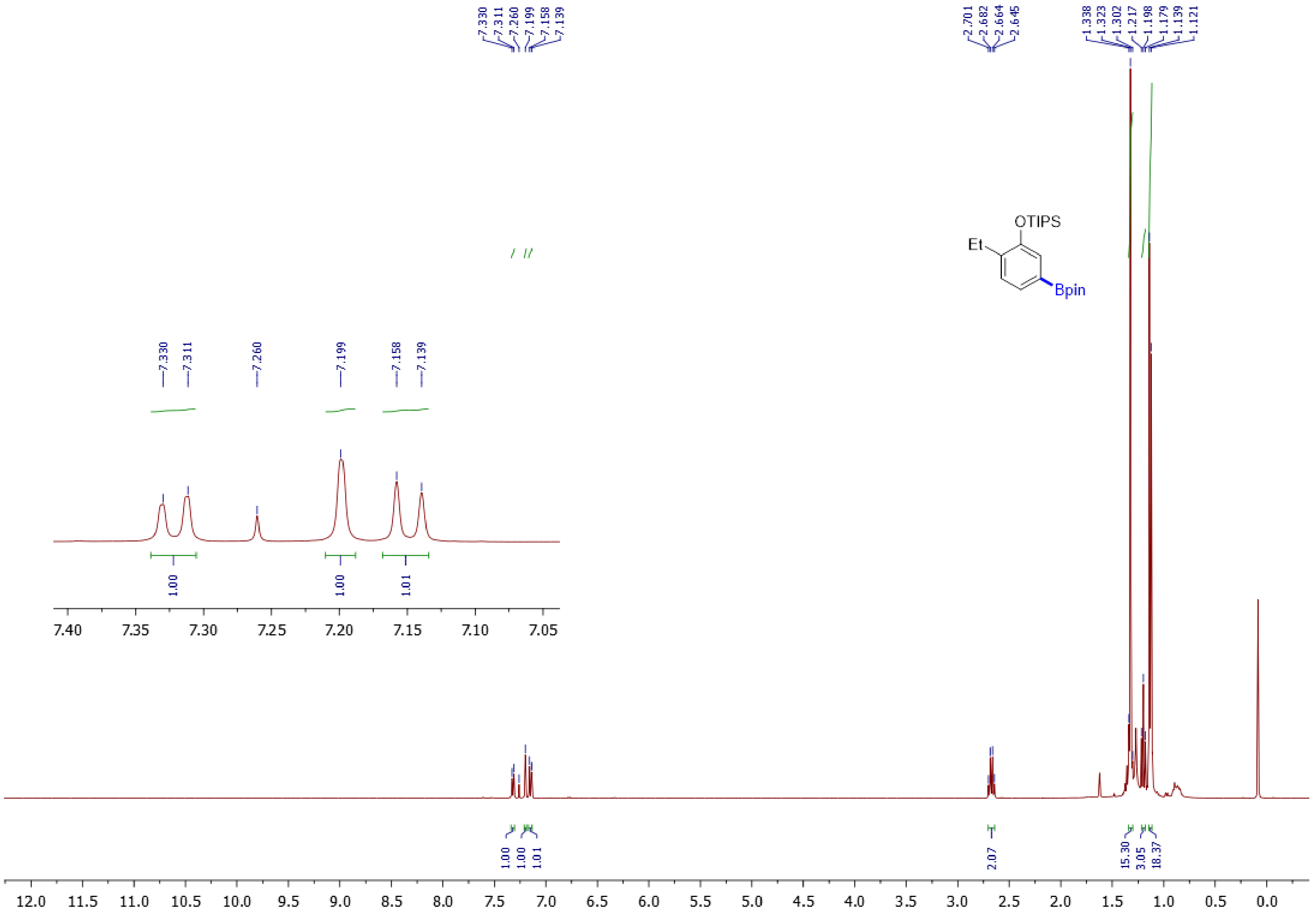

^{1}H-NMR spectra of **5e** (25 °C, 400 MHz, $CDCl_3$)

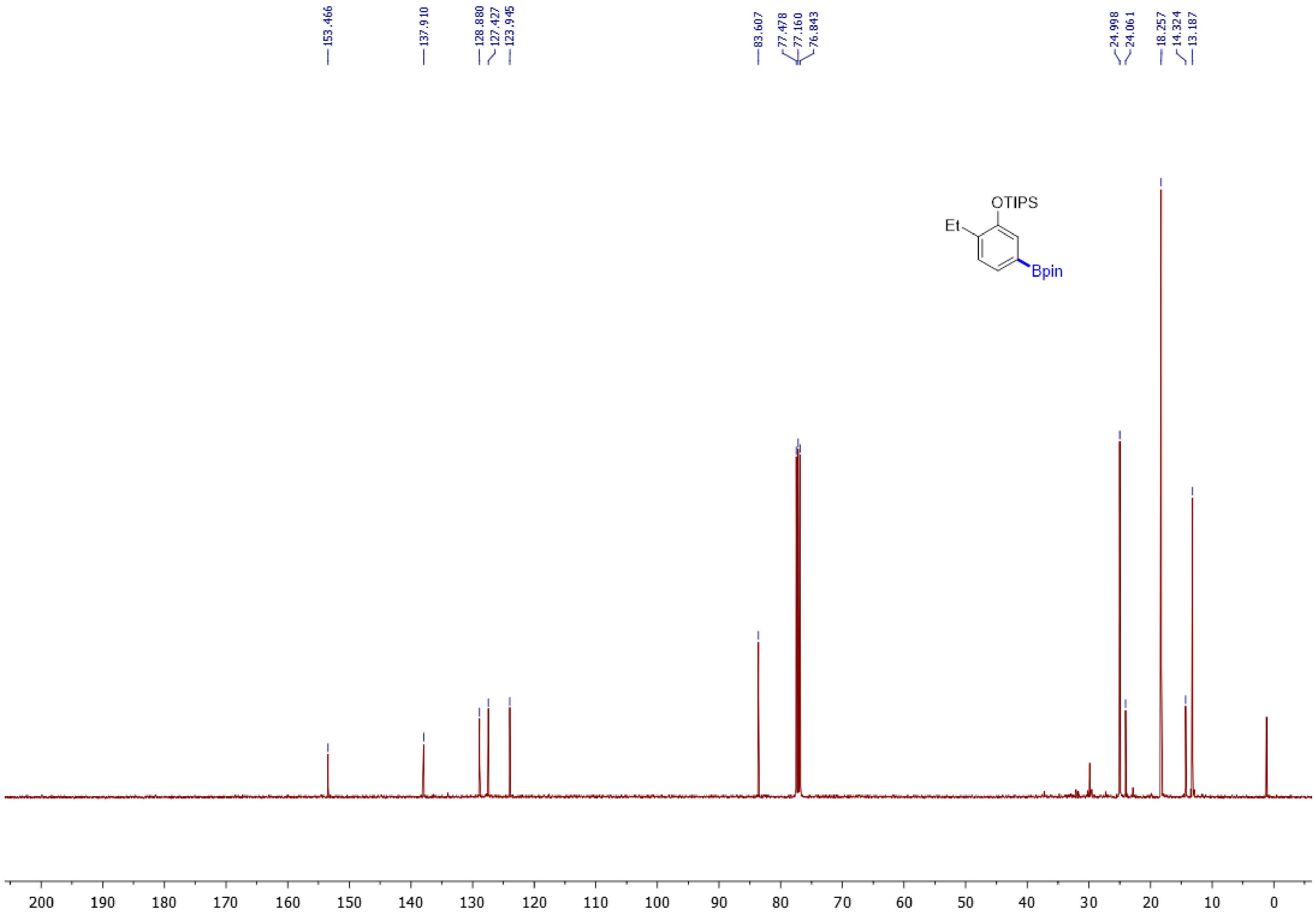

^{13}C-NMR spectra of **5e** (25 ºC, 100 MHz, $CDCl_3$)

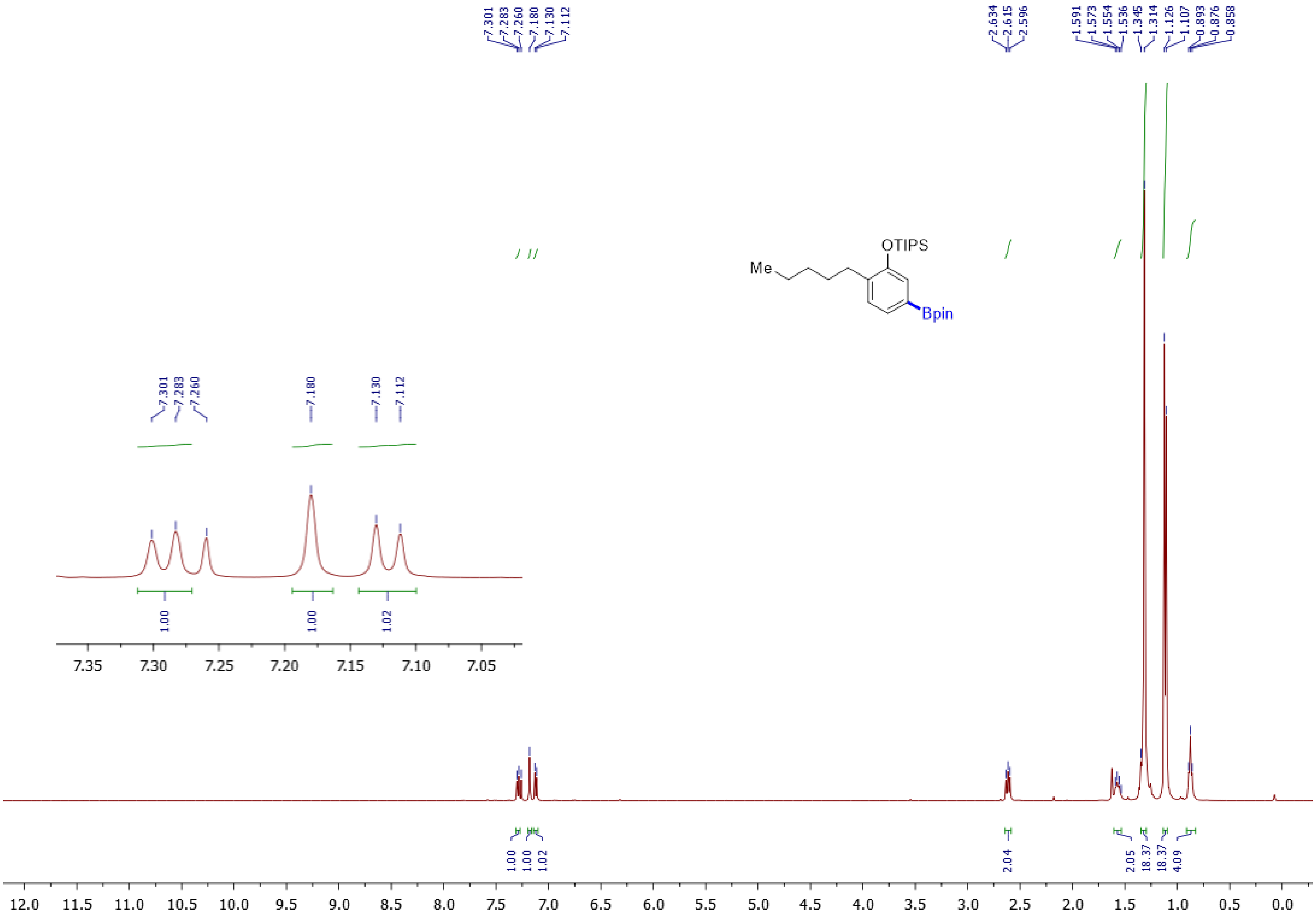

^{1}H-NMR spectra of **5f** (25 °C, 400 MHz, $CDCl_3$)

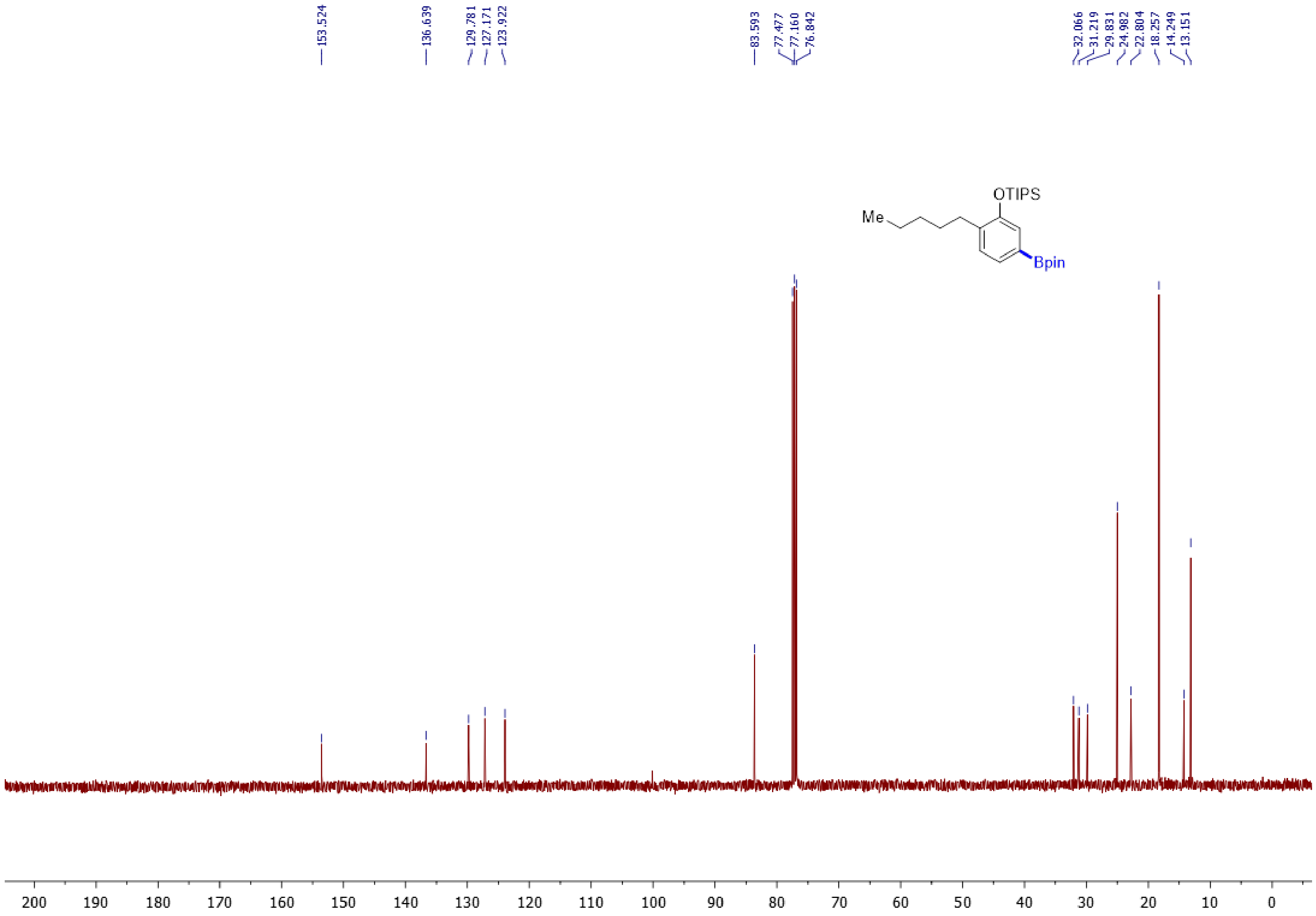

^{13}C-NMR spectra of **5f** (25 °C, 100 MHz, $CDCl_3$)

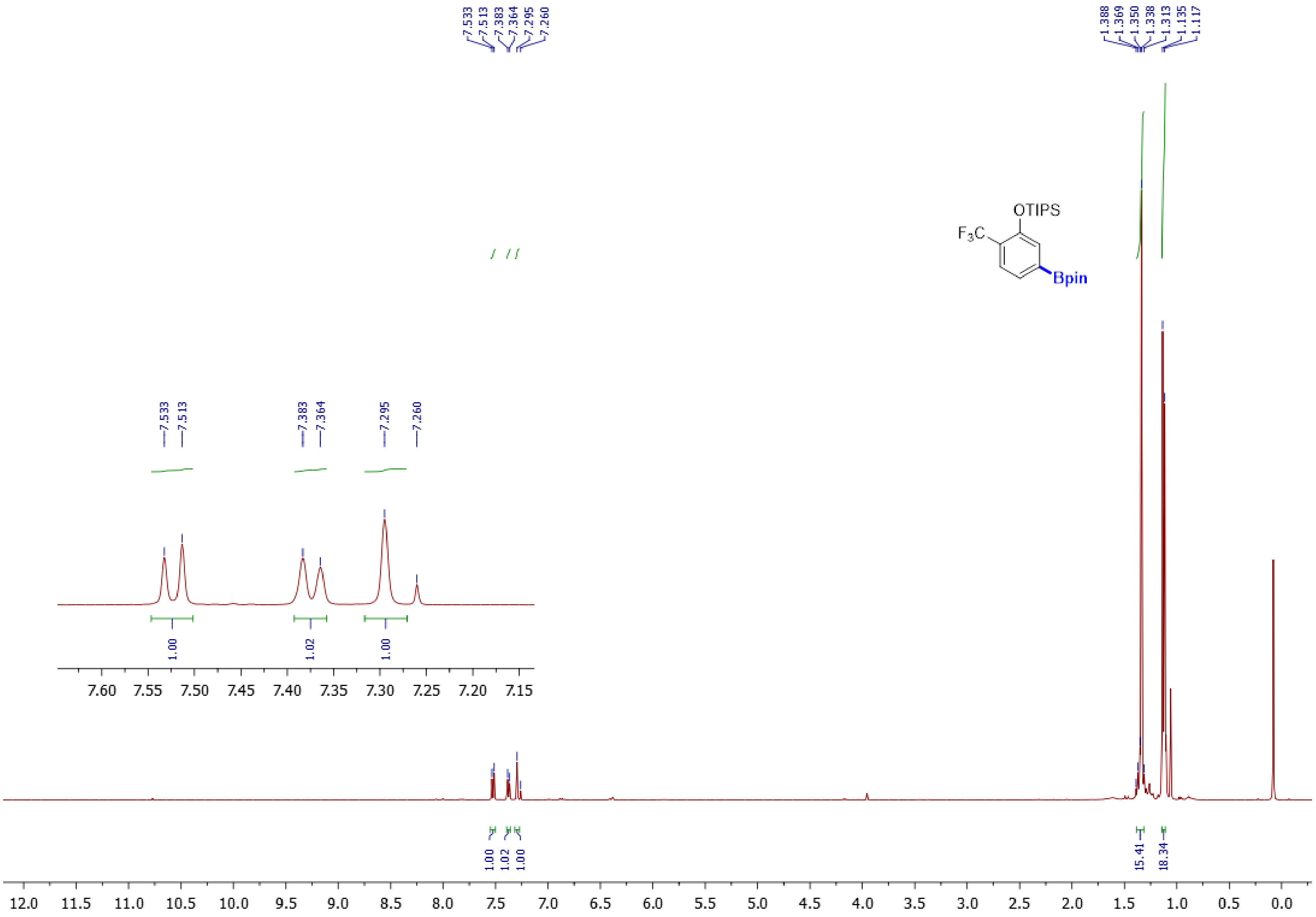

^{1}H-NMR spectra of **5g** (25 °C, 400 MHz, $CDCl_3$)

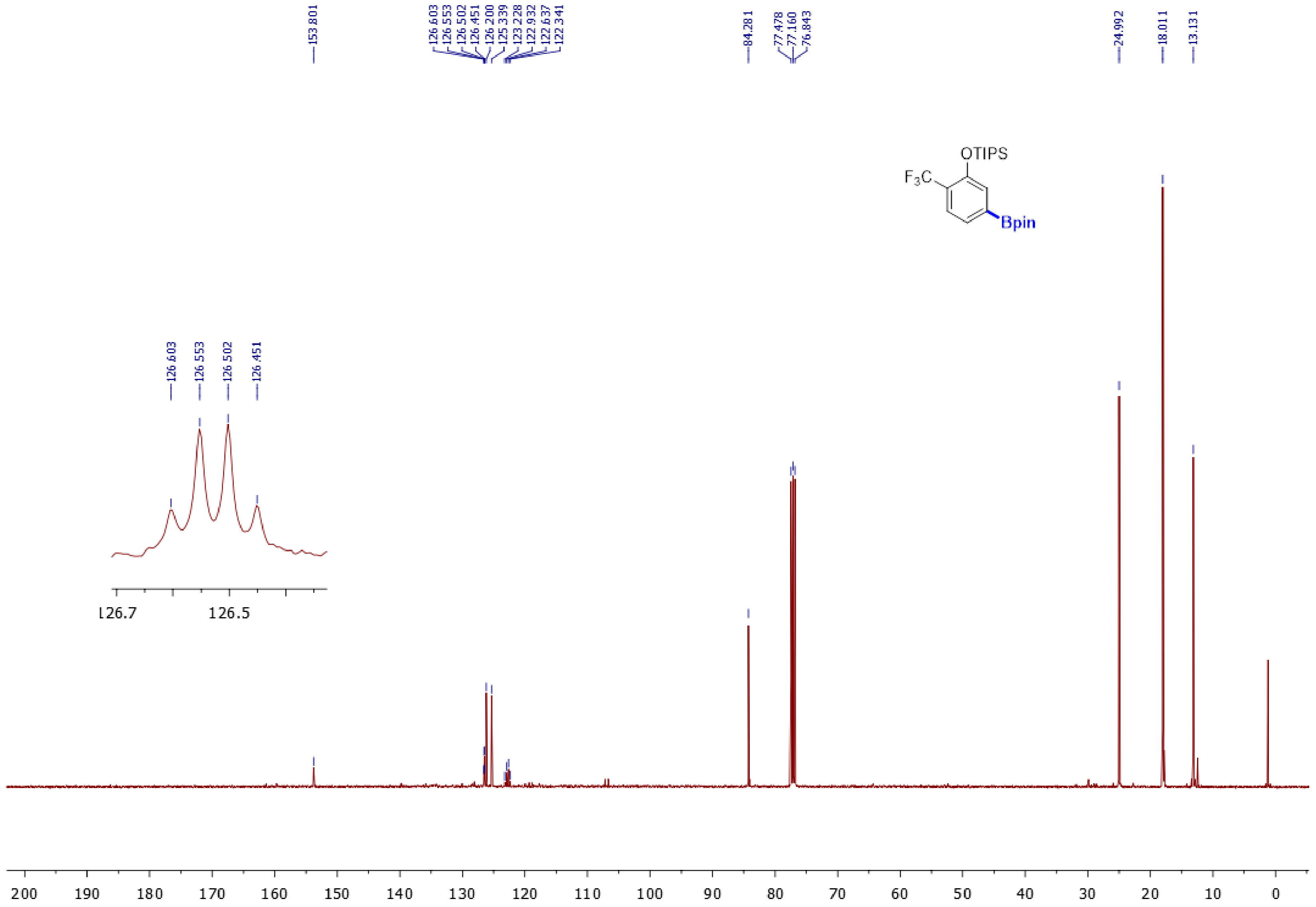

^{13}C-NMR spectra of **5g** (25 °C, 100 MHz, $CDCl_3$)

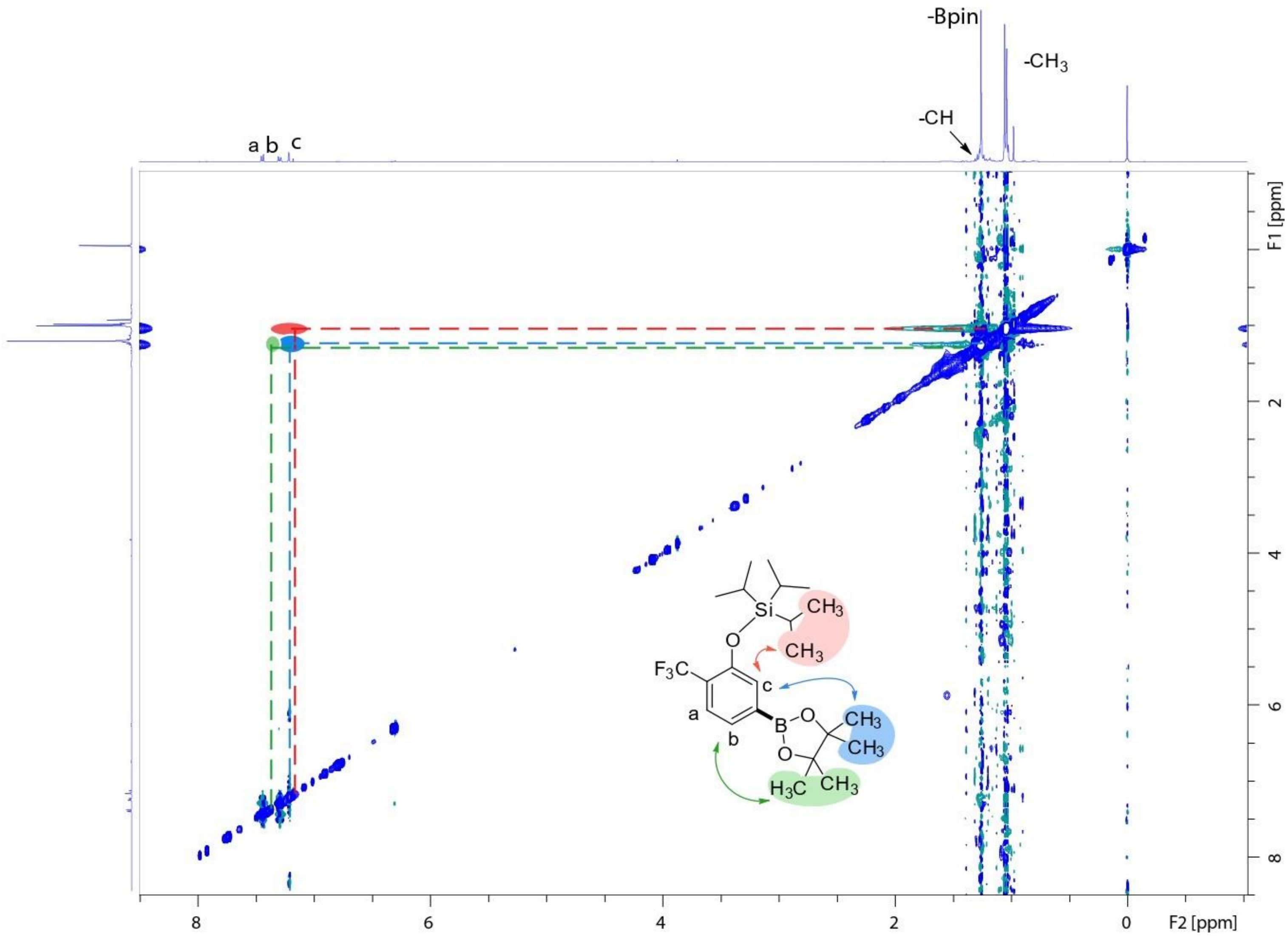

NOESY-NMR spectra of **5g** (25 °C, 100 MHz, CDCl$_3$)

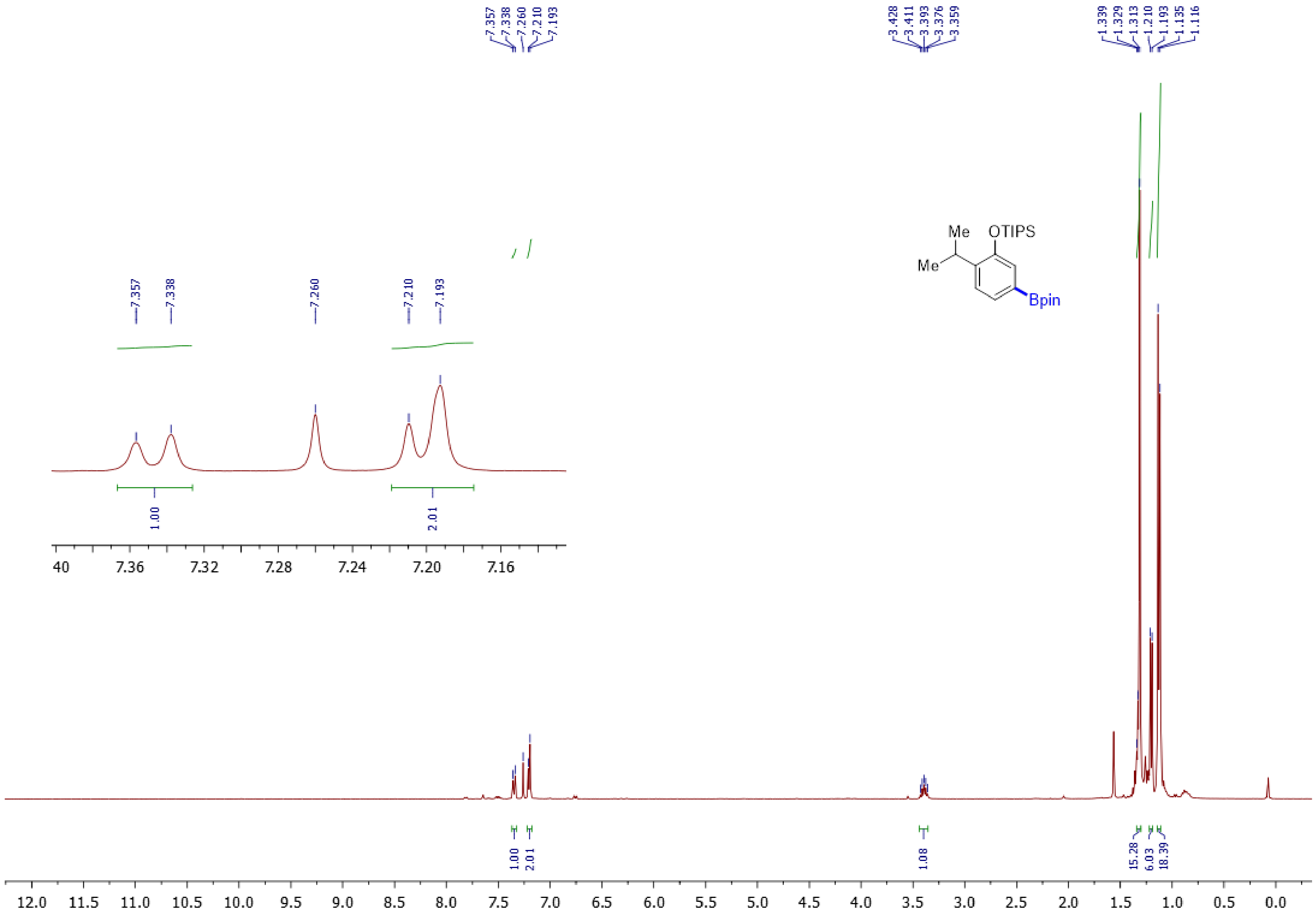

^{1}H-NMR spectra of **5h** (25 ^{o}C, 400 MHz, $CDCl_3$)

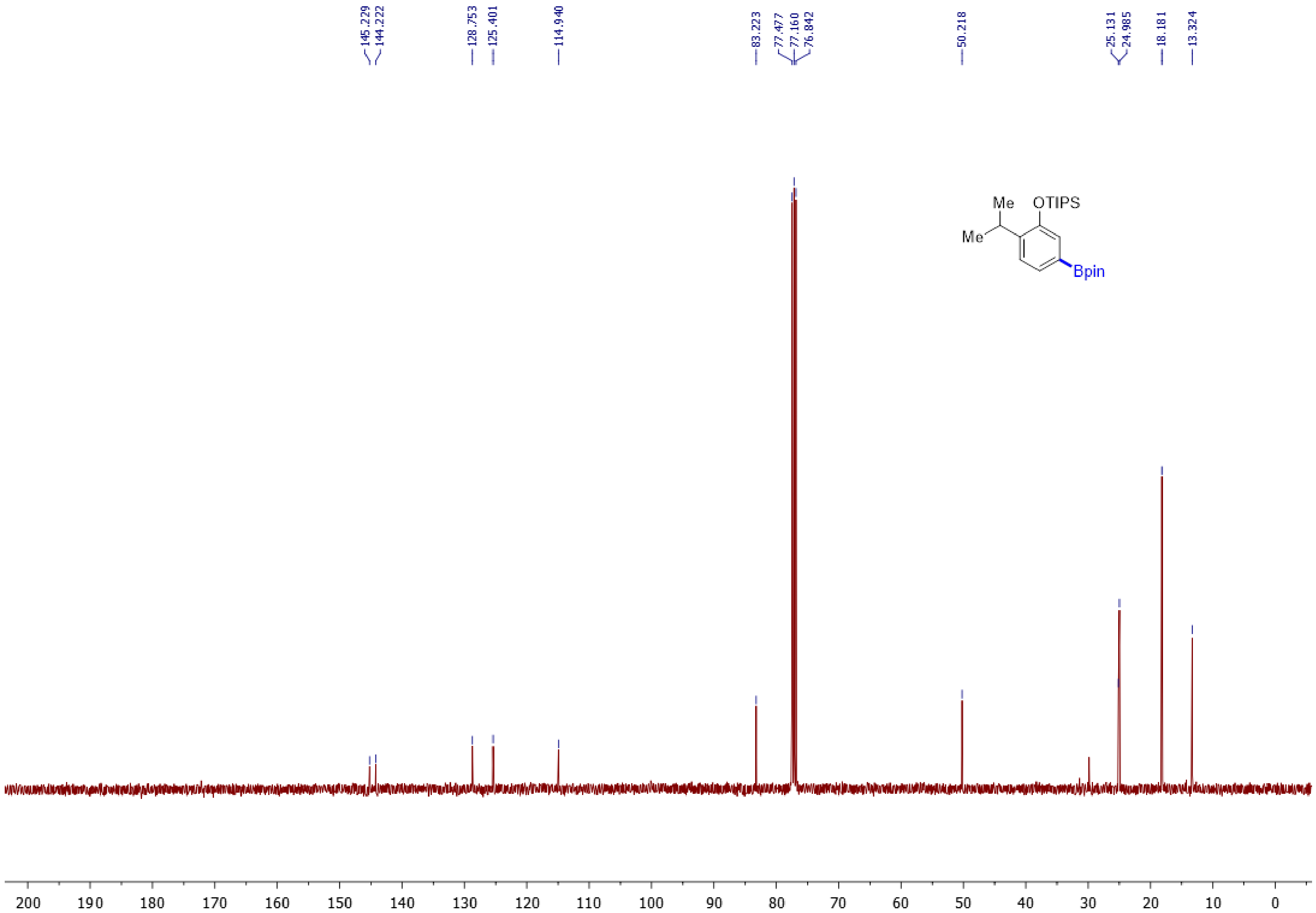

^{13}C-NMR spectra of **5h** (25 °C, 100 MHz, $CDCl_3$)

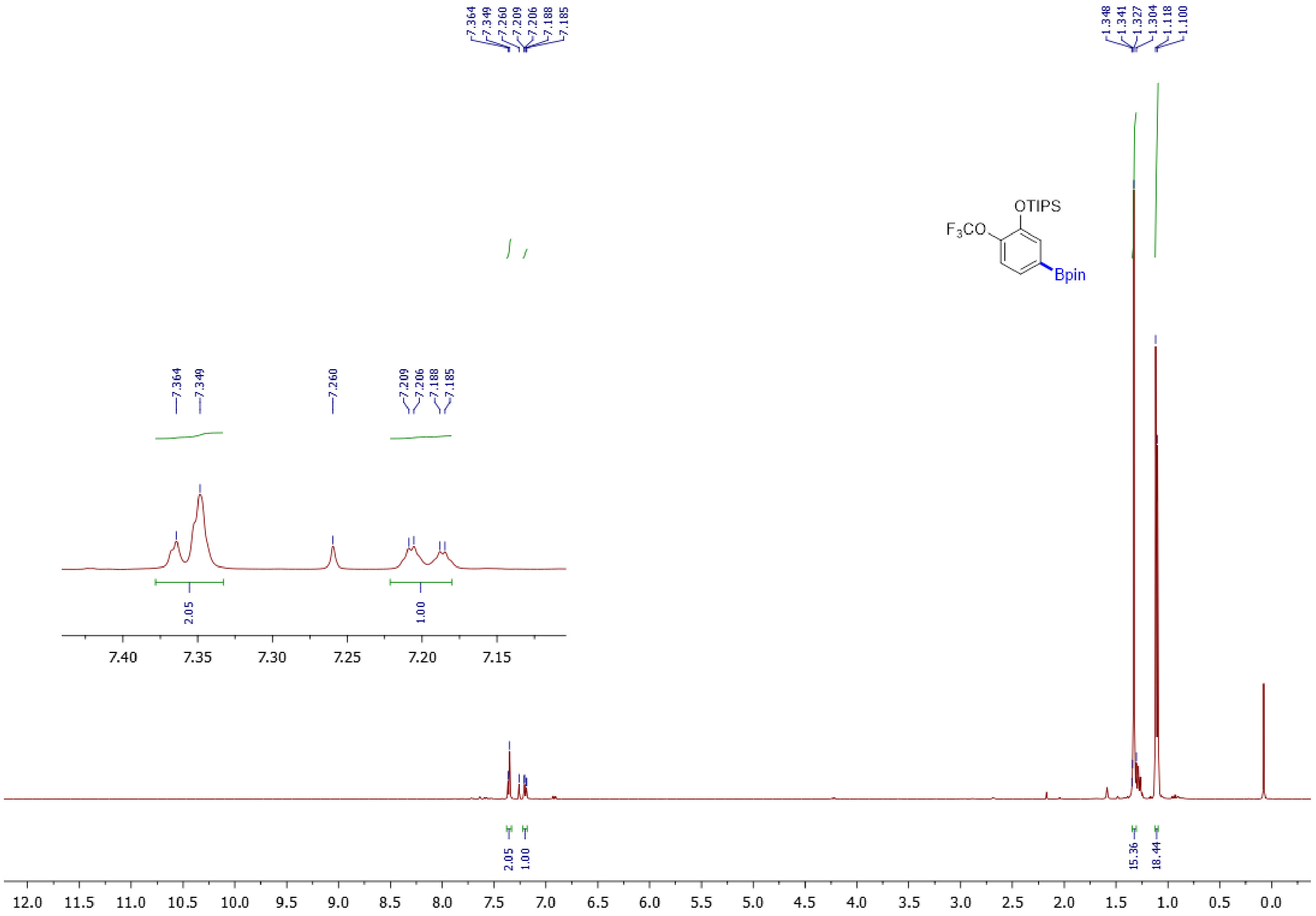

^{1}H-NMR spectra of **5i** (25 °C, 400 MHz, $CDCl_3$)

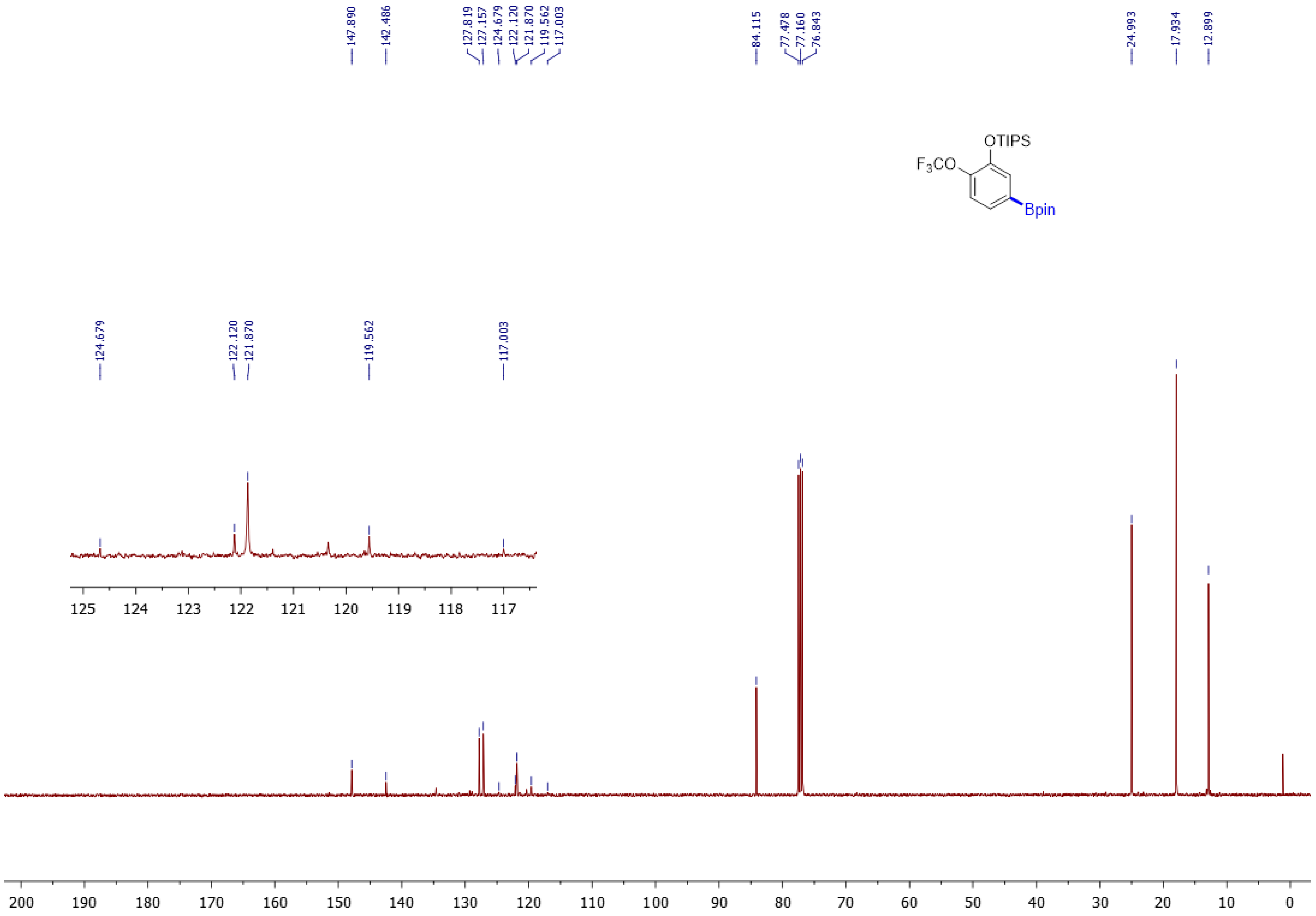

^{13}C-NMR spectra of **5i** (25 ^{o}C, 100 MHz, $CDCl_3$)

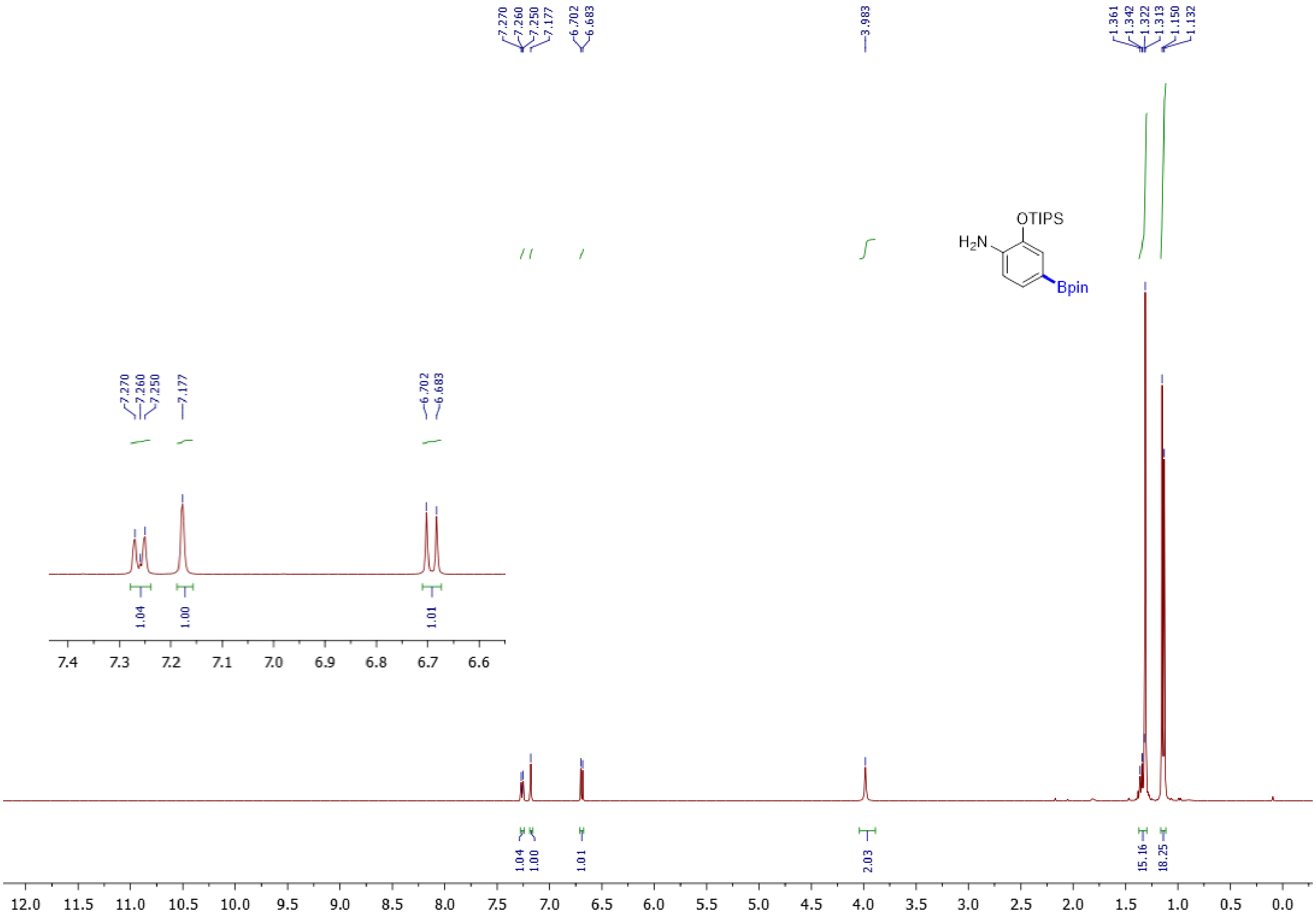

^{1}H-NMR spectra of **5j** (25 °C, 400 MHz, $CDCl_3$)

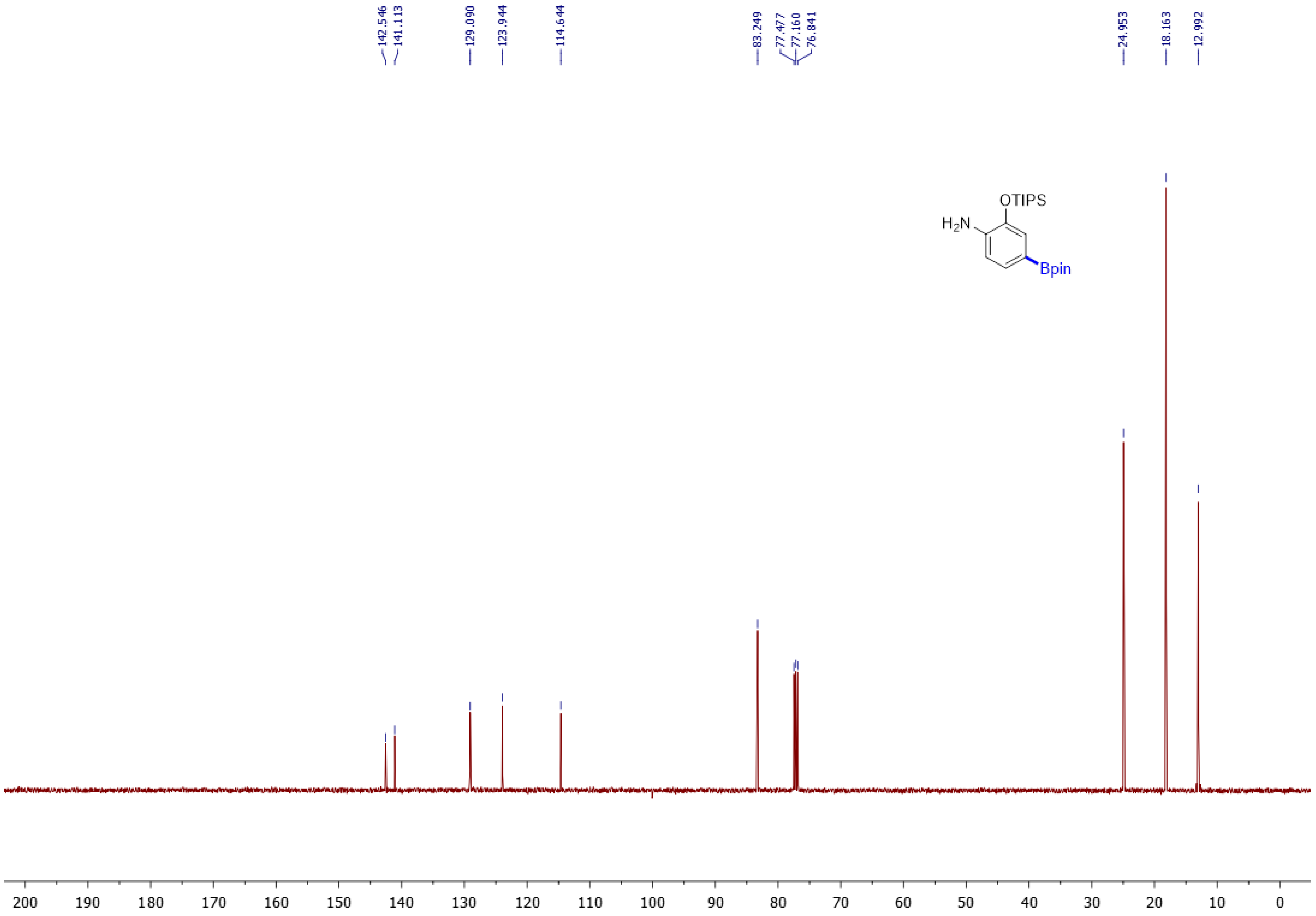

^{13}C-NMR spectra of **5j** (25 ^{o}C, 100 MHz, $CDCl_3$)

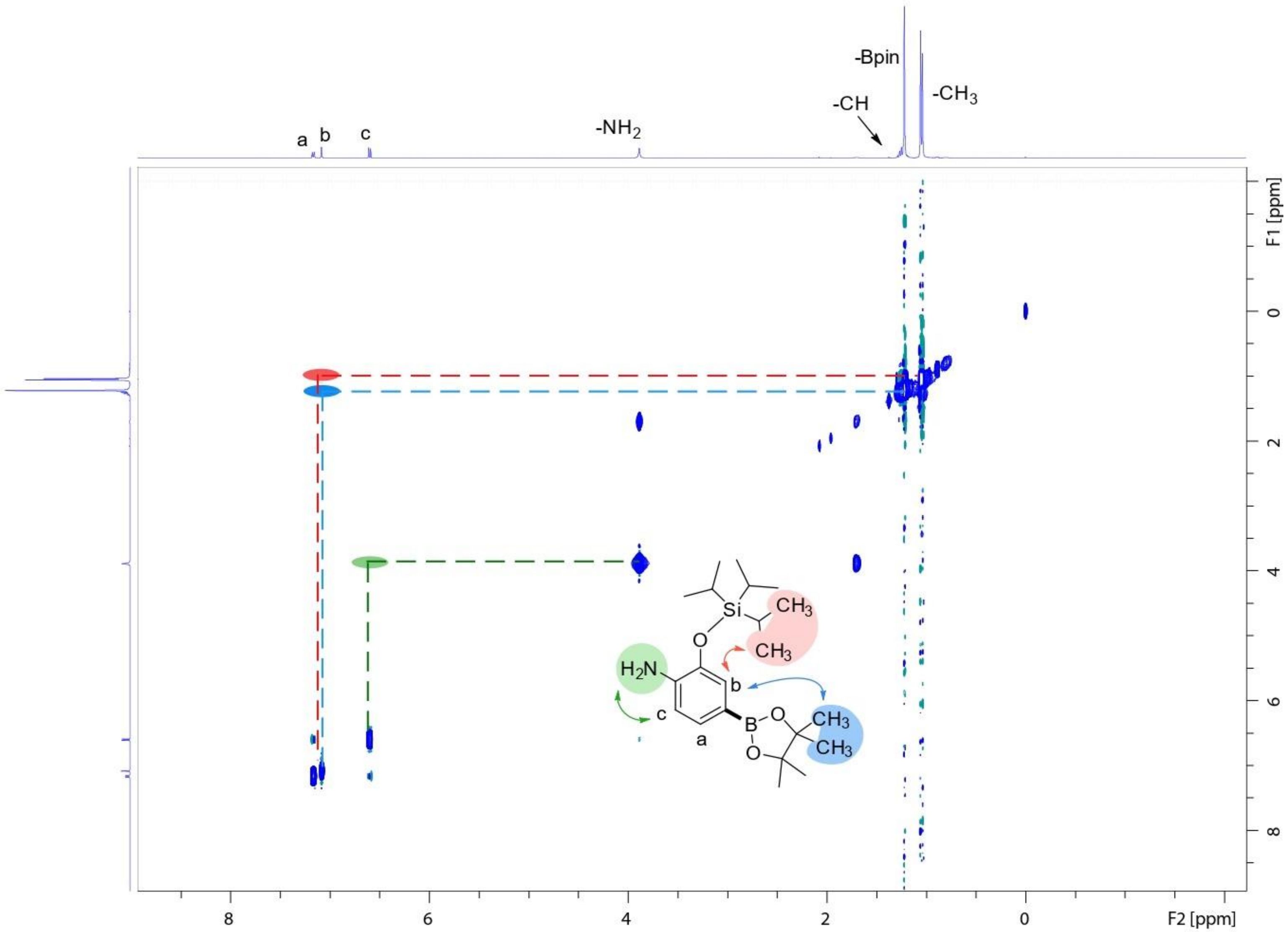

NOESY-NMR spectra of **5j** (25 ^{o}C, 100 MHz, $CDCl_3$)

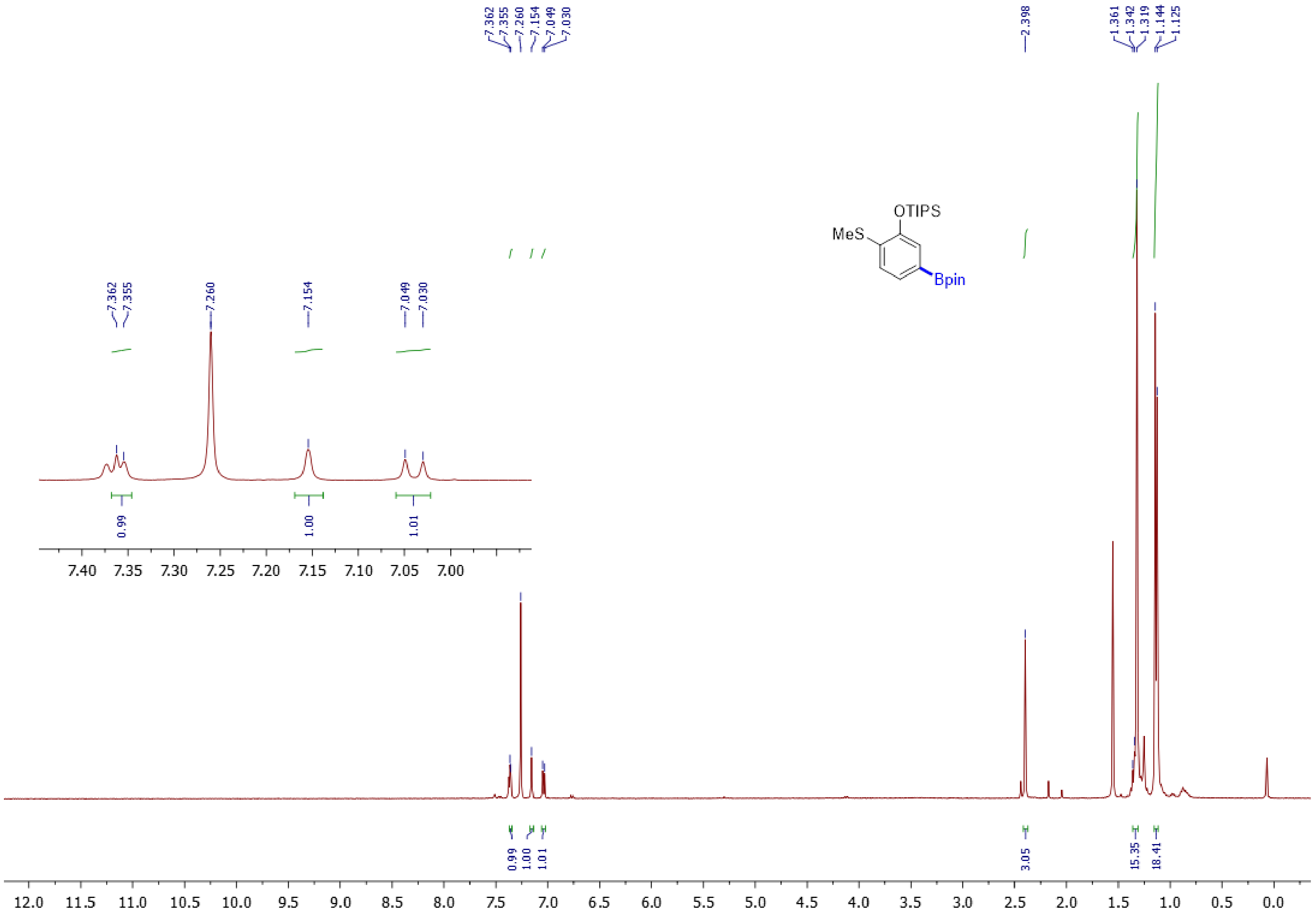

^{1}H-NMR spectra of **5k** (25 °C, 400 MHz, $CDCl_3$)

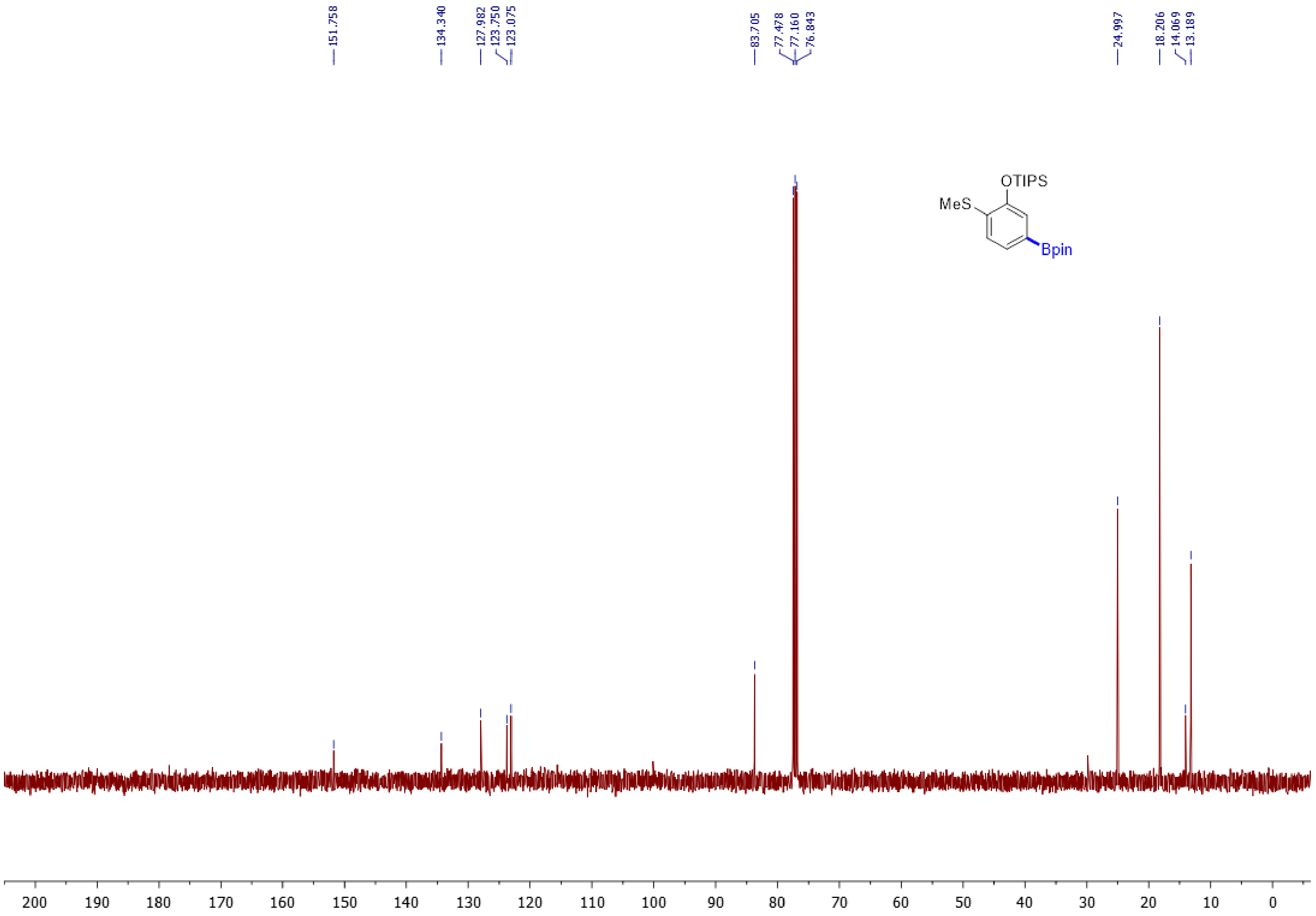

^{13}C-NMR spectra of **5k** (25 °C, 100 MHz, $CDCl_3$)

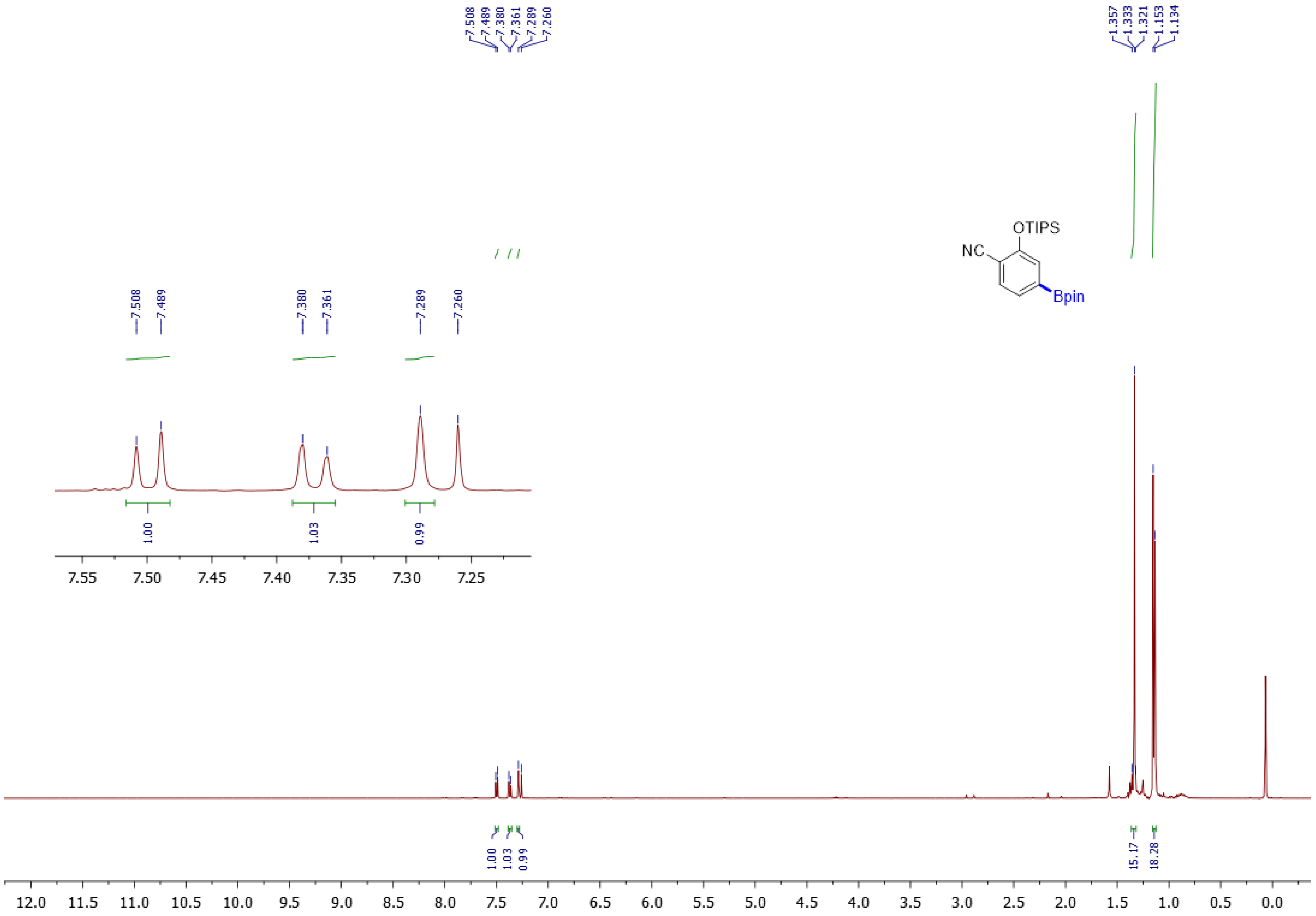

^{1}H-NMR spectra of **5l** (25 °C, 400 MHz, $CDCl_3$)

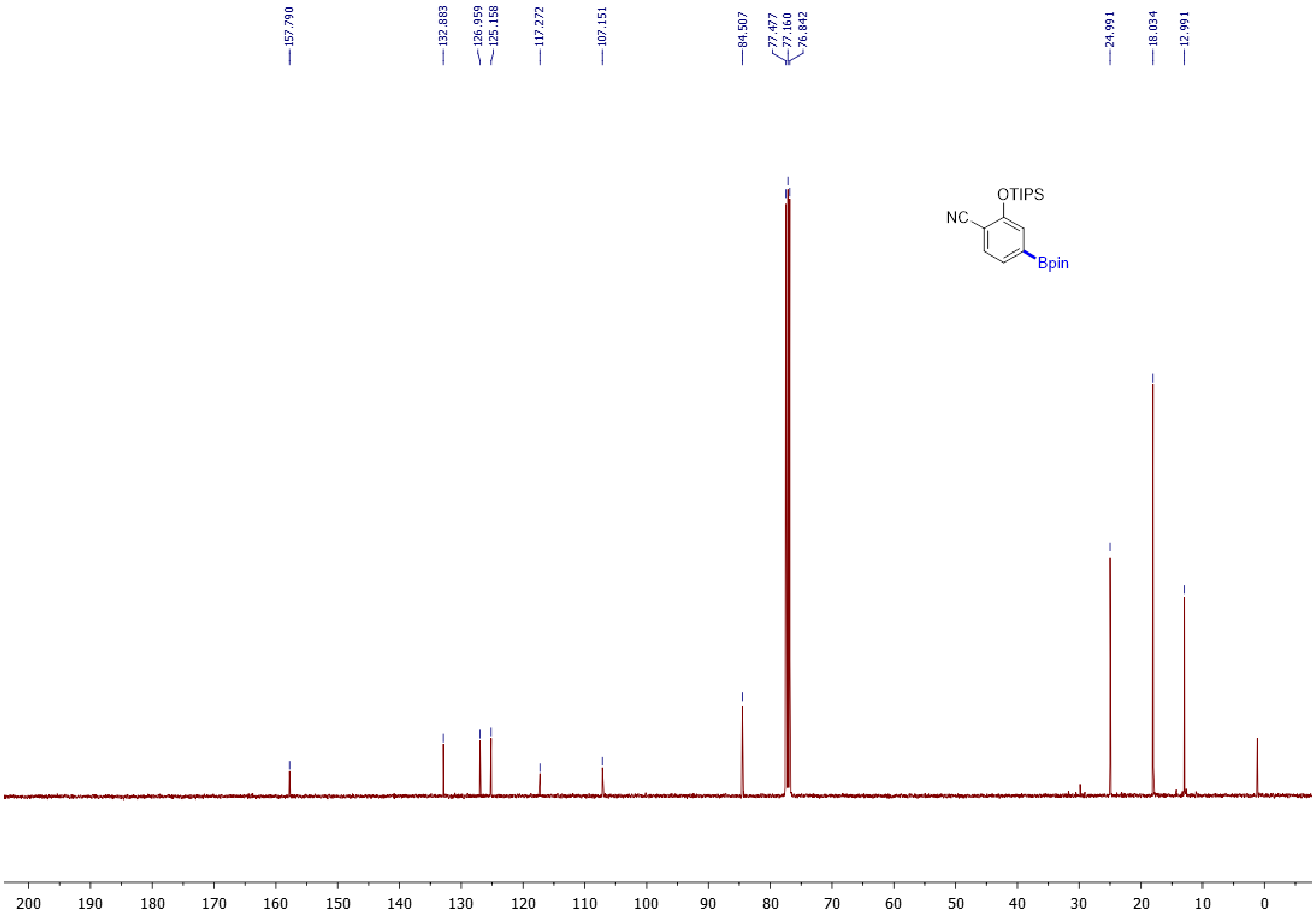

^{13}C-NMR spectra of **5l** (25 °C, 100 MHz, $CDCl_3$)

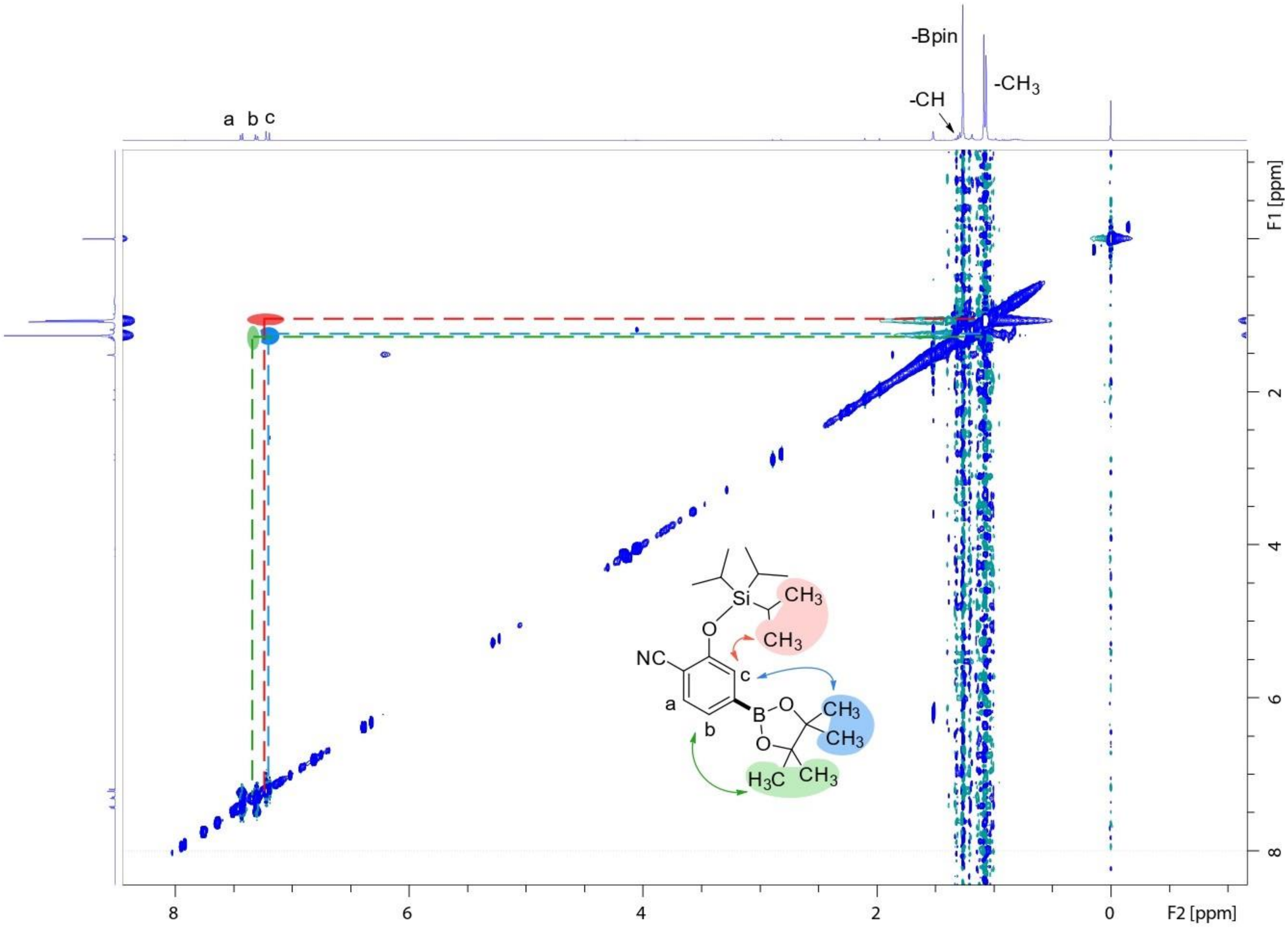

NOESY-NMR spectra of **5l** (25 ^{o}C, 100 MHz, $CDCl_3$)

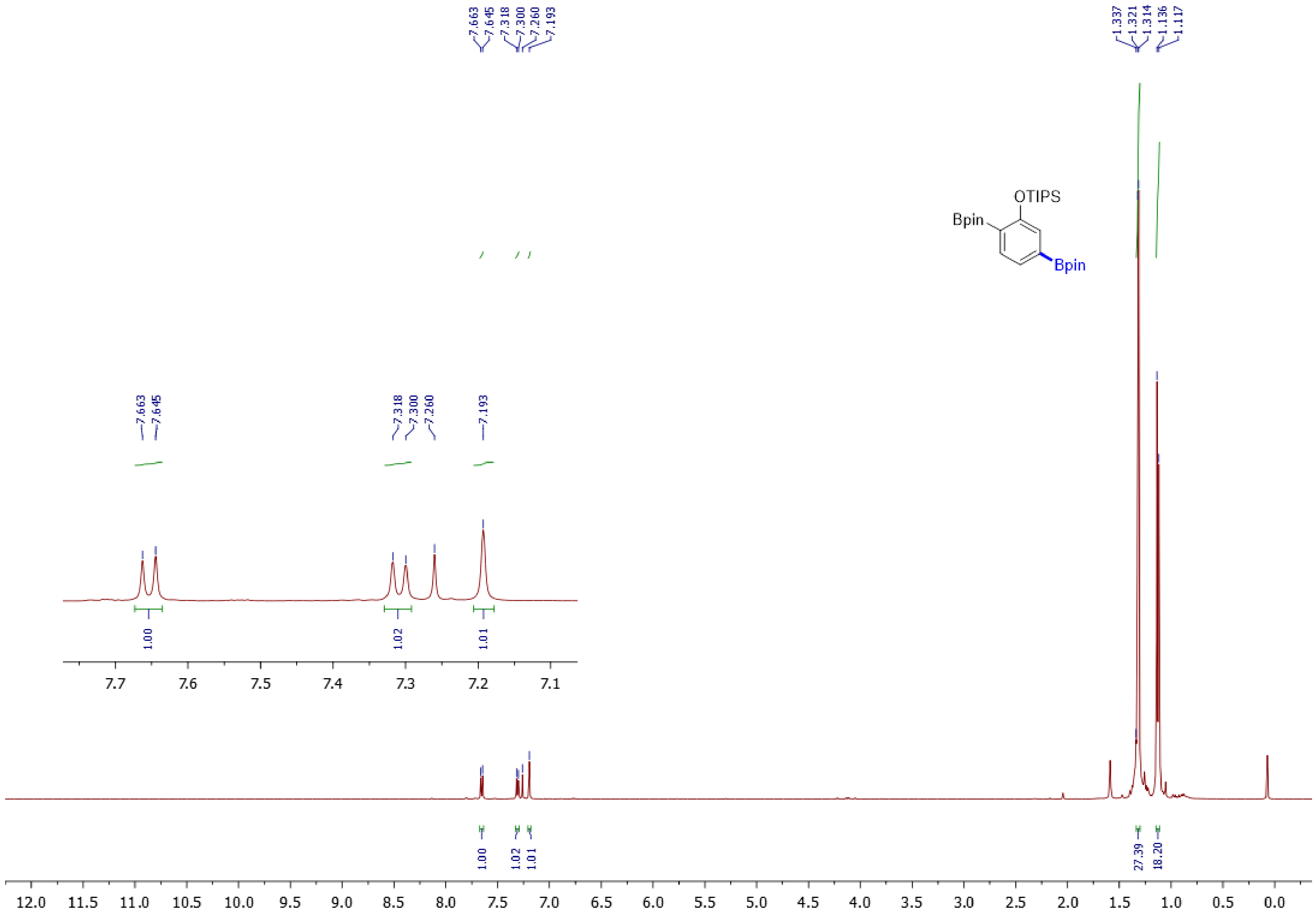

^{1}H-NMR spectra of **5m** (25 ^{o}C, 400 MHz, $CDCl_3$)

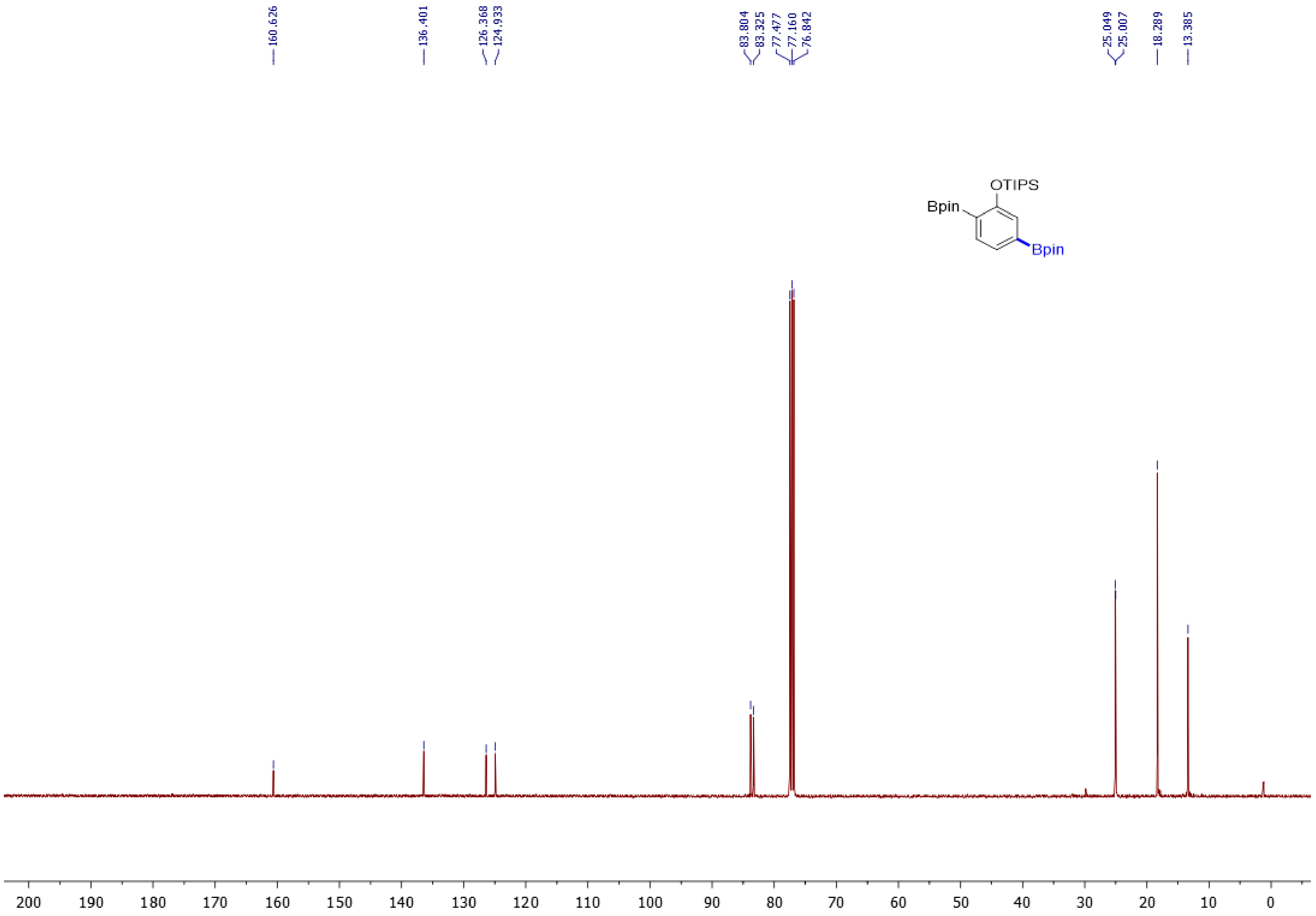

^{13}C-NMR spectra of **5m** (25 ^{o}C, 100 MHz, $CDCl_3$)

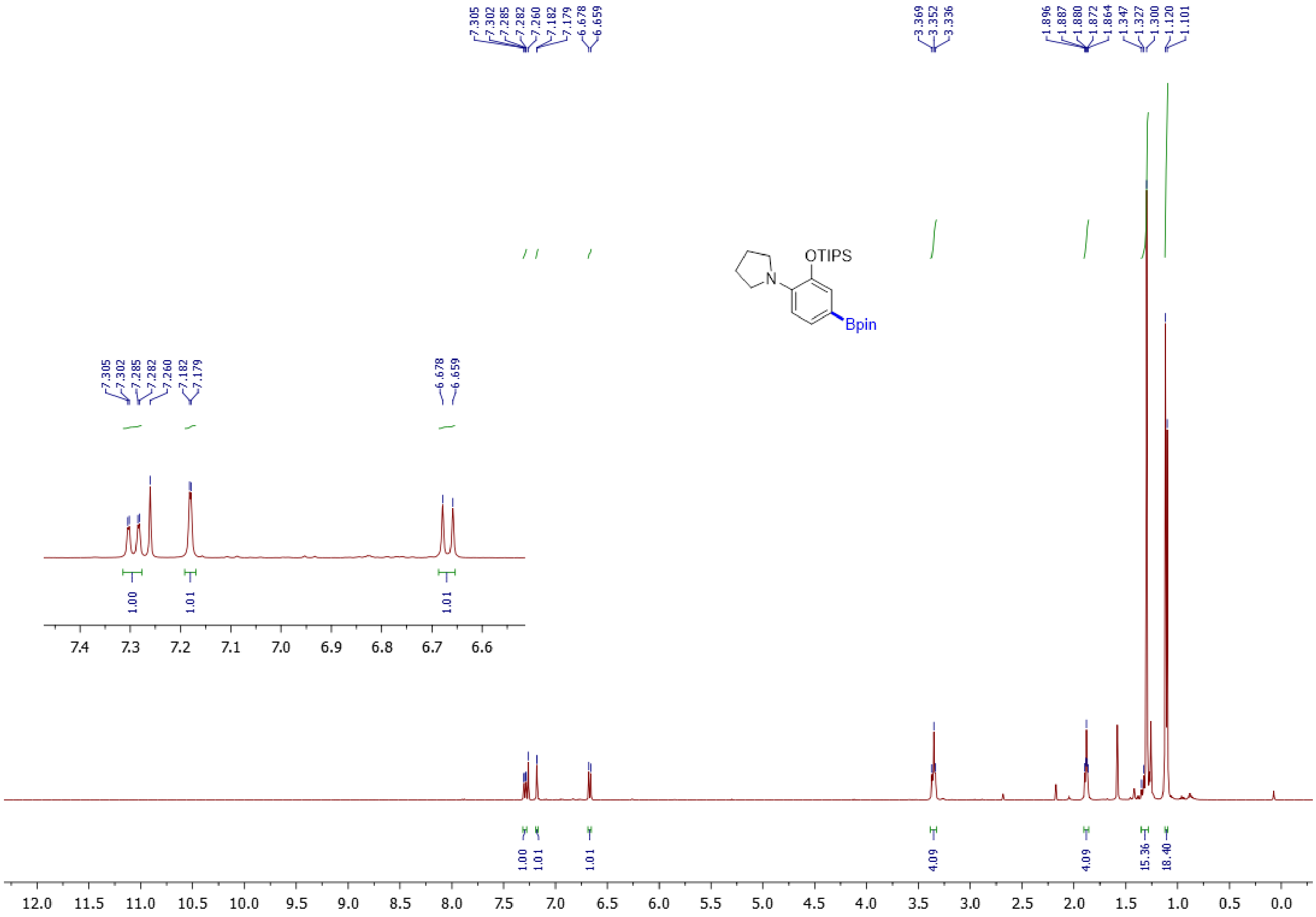

^{1}H-NMR spectra of **5n** (25 ^{o}C, 400 MHz, $CDCl_3$)

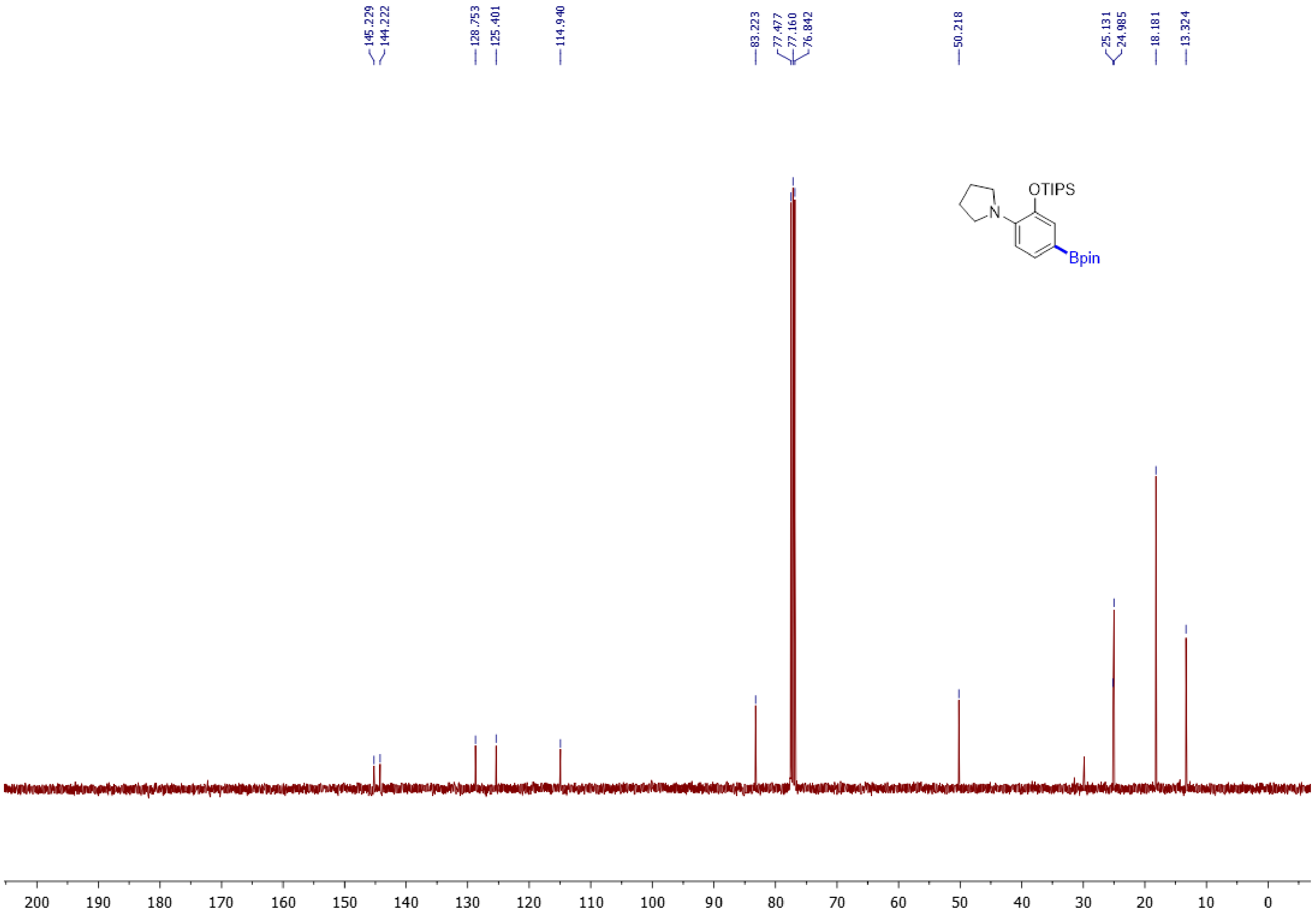

^{13}C-NMR spectra of **5n** (25 °C, 100 MHz, $CDCl_3$)

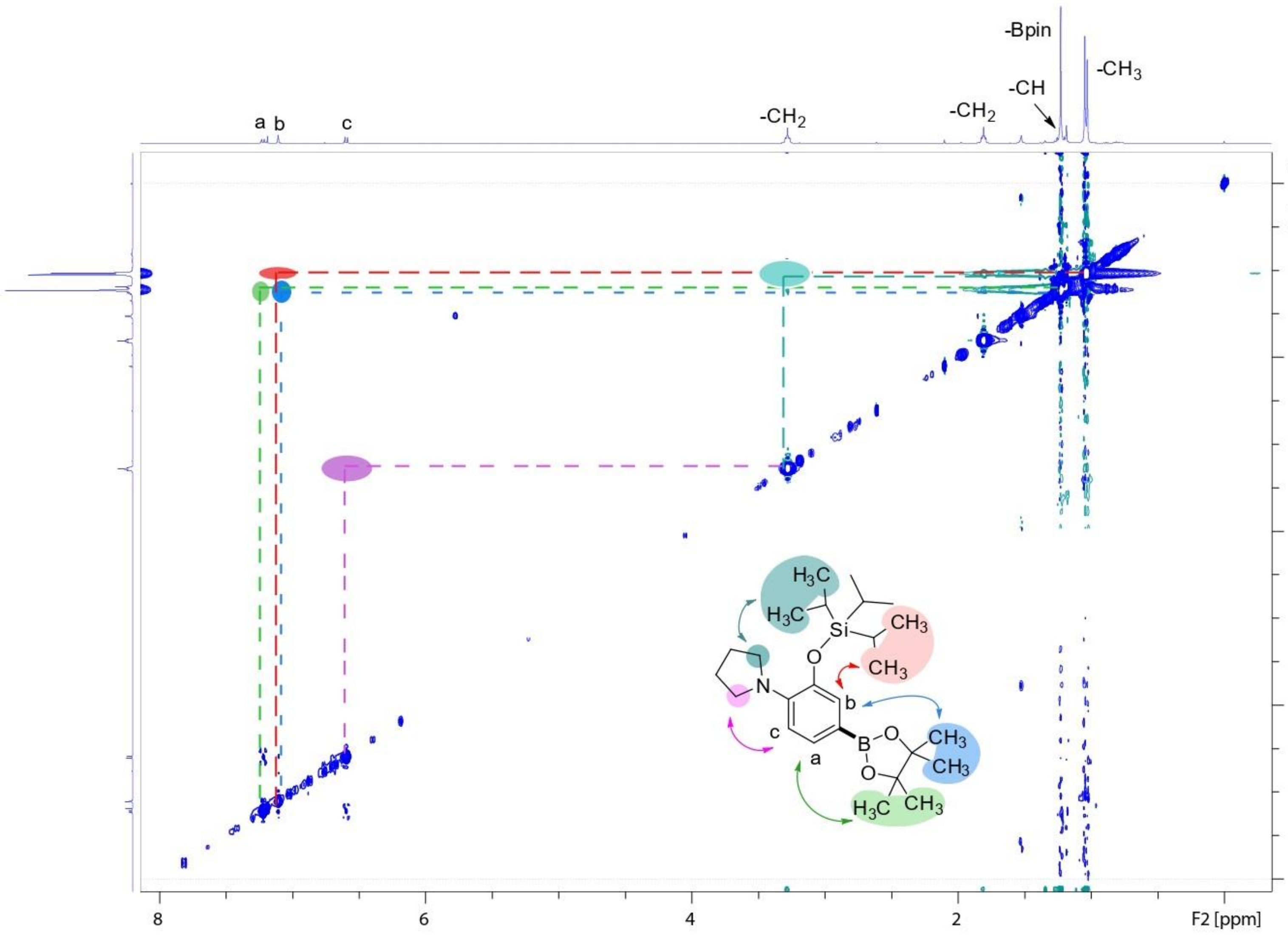

NOESY-NMR spectra of **5n** (25 ^{o}C, 100 MHz, $CDCl_3$)

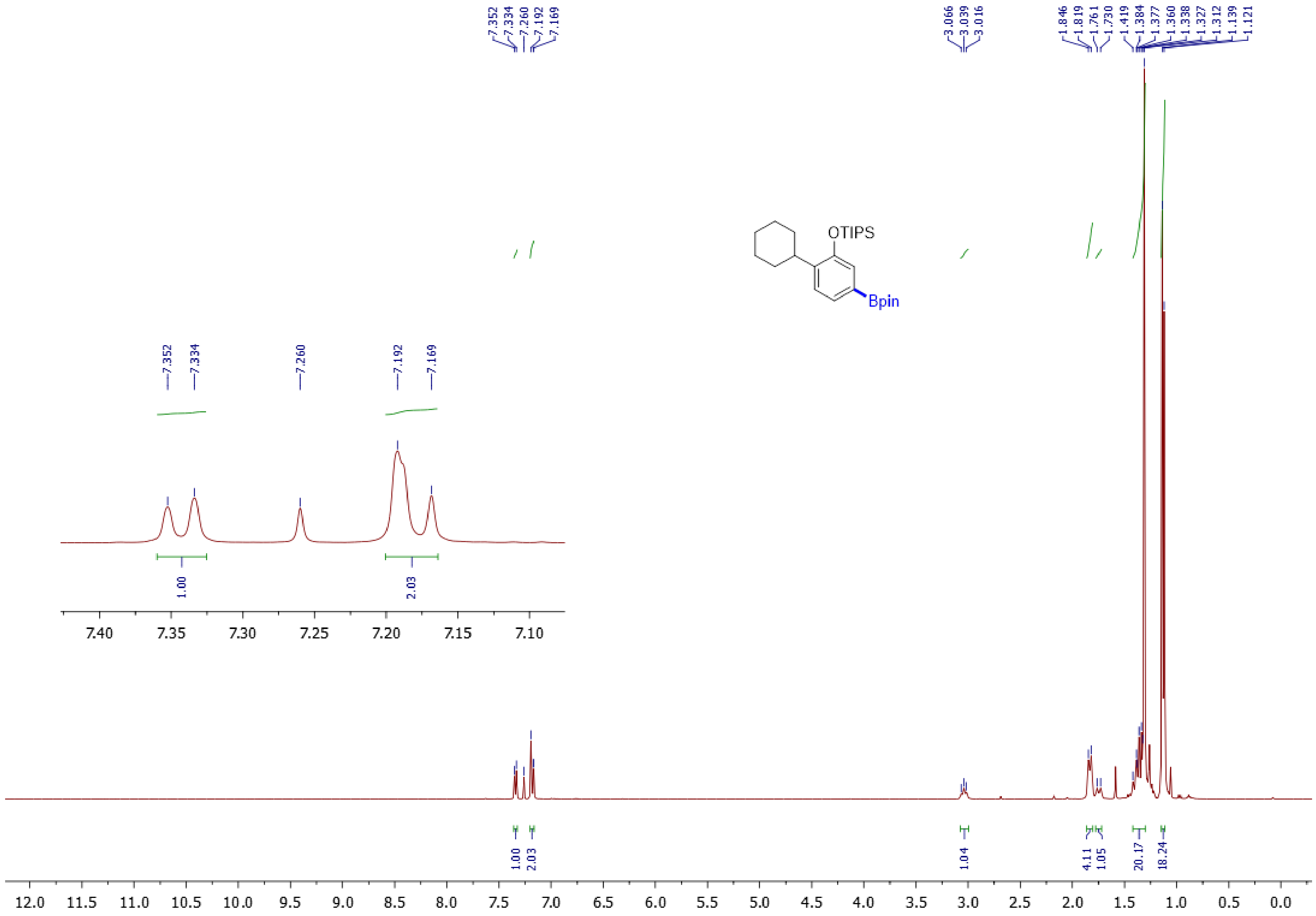

^{1}H-NMR spectra of **5o** (25 ^{o}C, 400 MHz, $CDCl_3$)

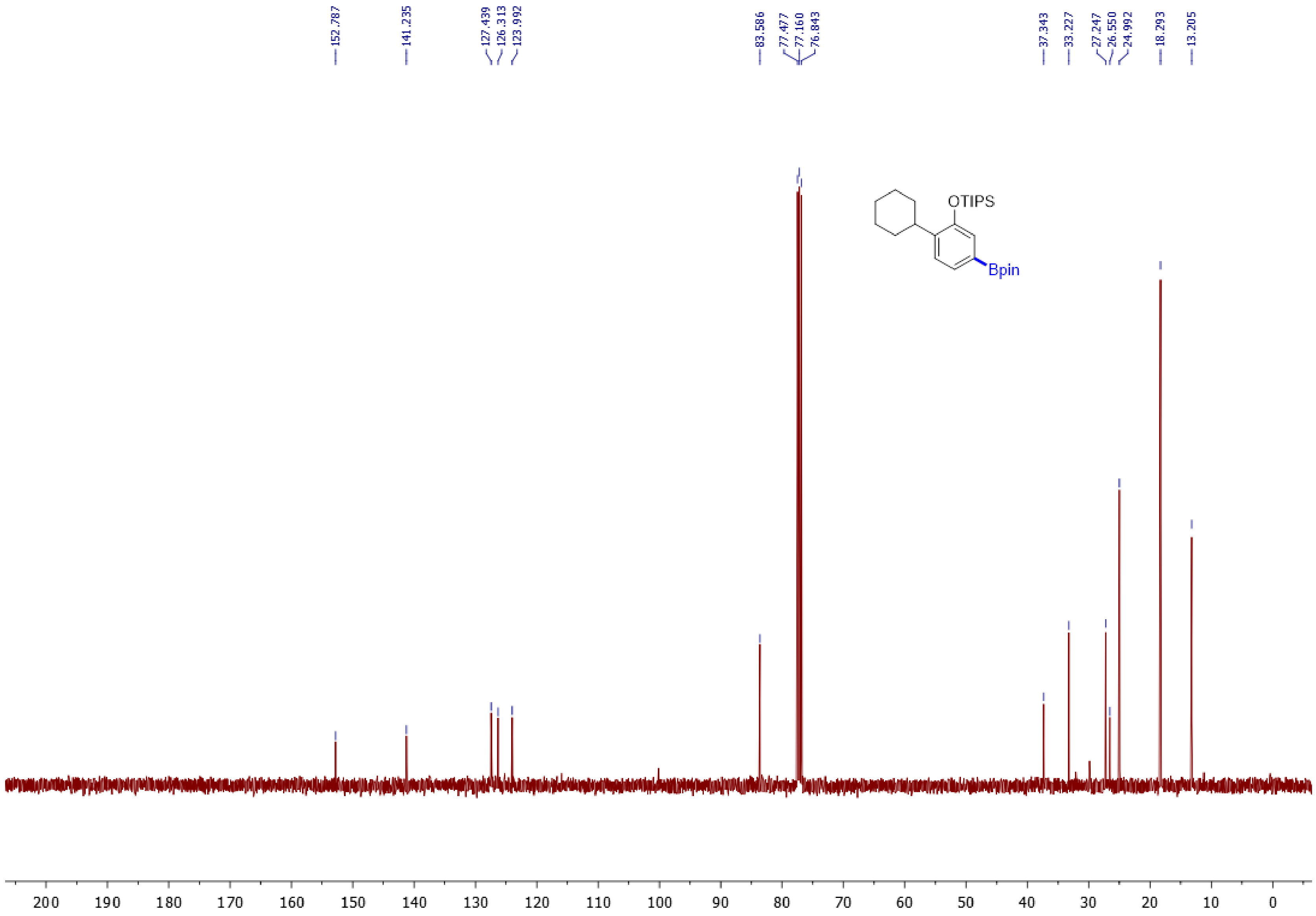

^{13}C-NMR spectra of **5o** (25 °C, 100 MHz, $CDCl_3$)

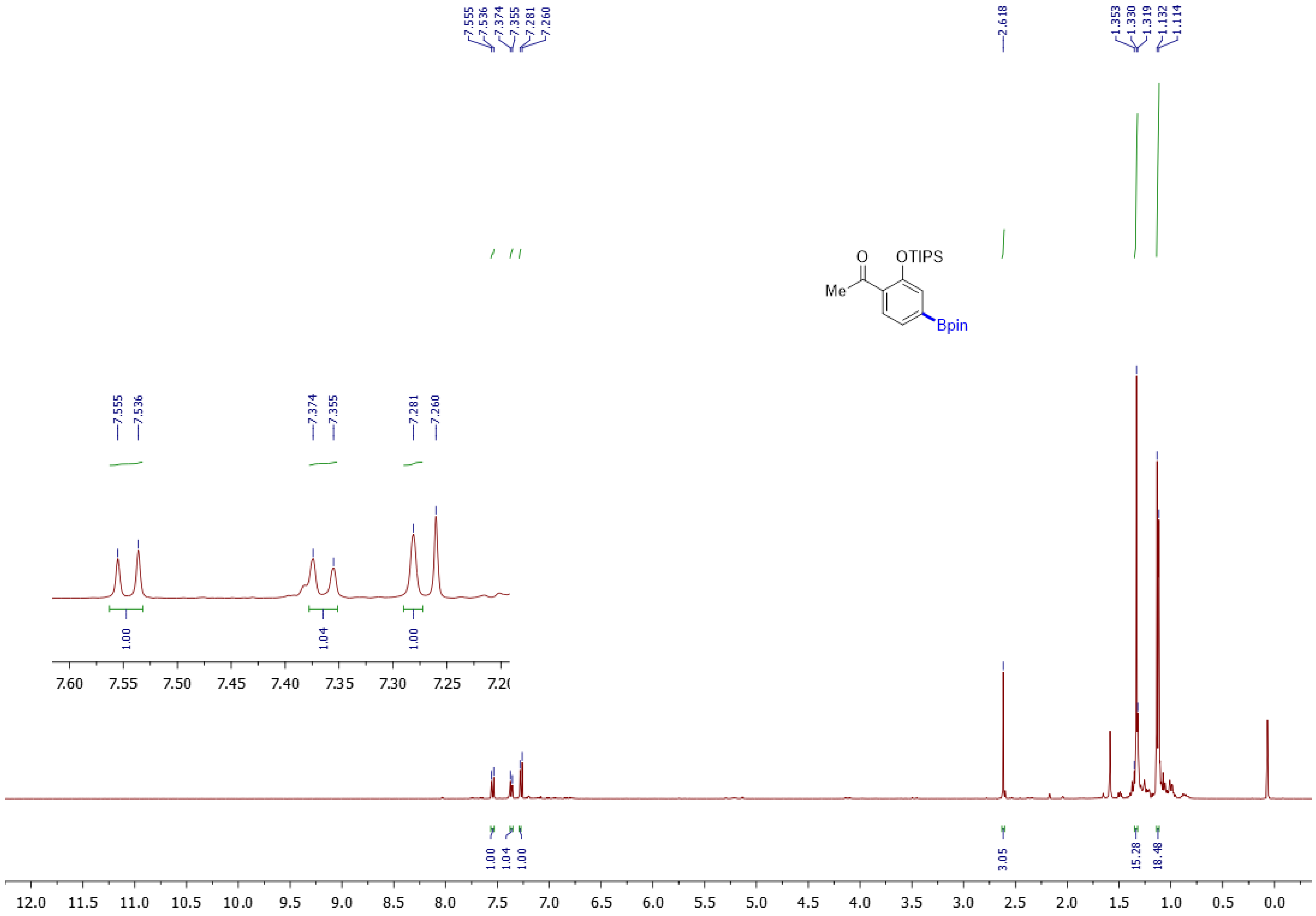

^{1}H-NMR spectra of **5p** (25 °C, 400 MHz, $CDCl_3$)

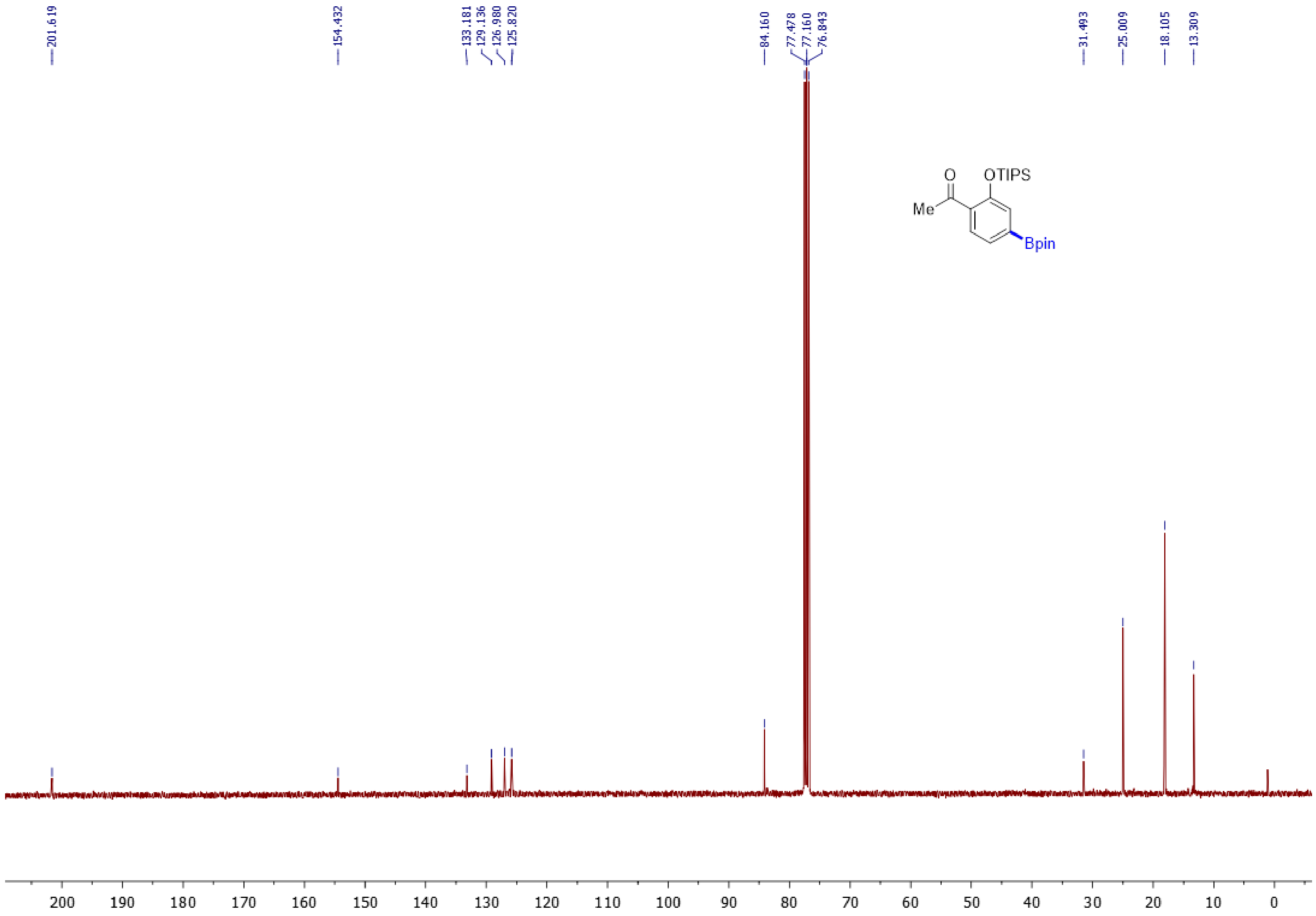

^{13}C-NMR spectra of **5p** (25 °C, 100 MHz, $CDCl_3$)

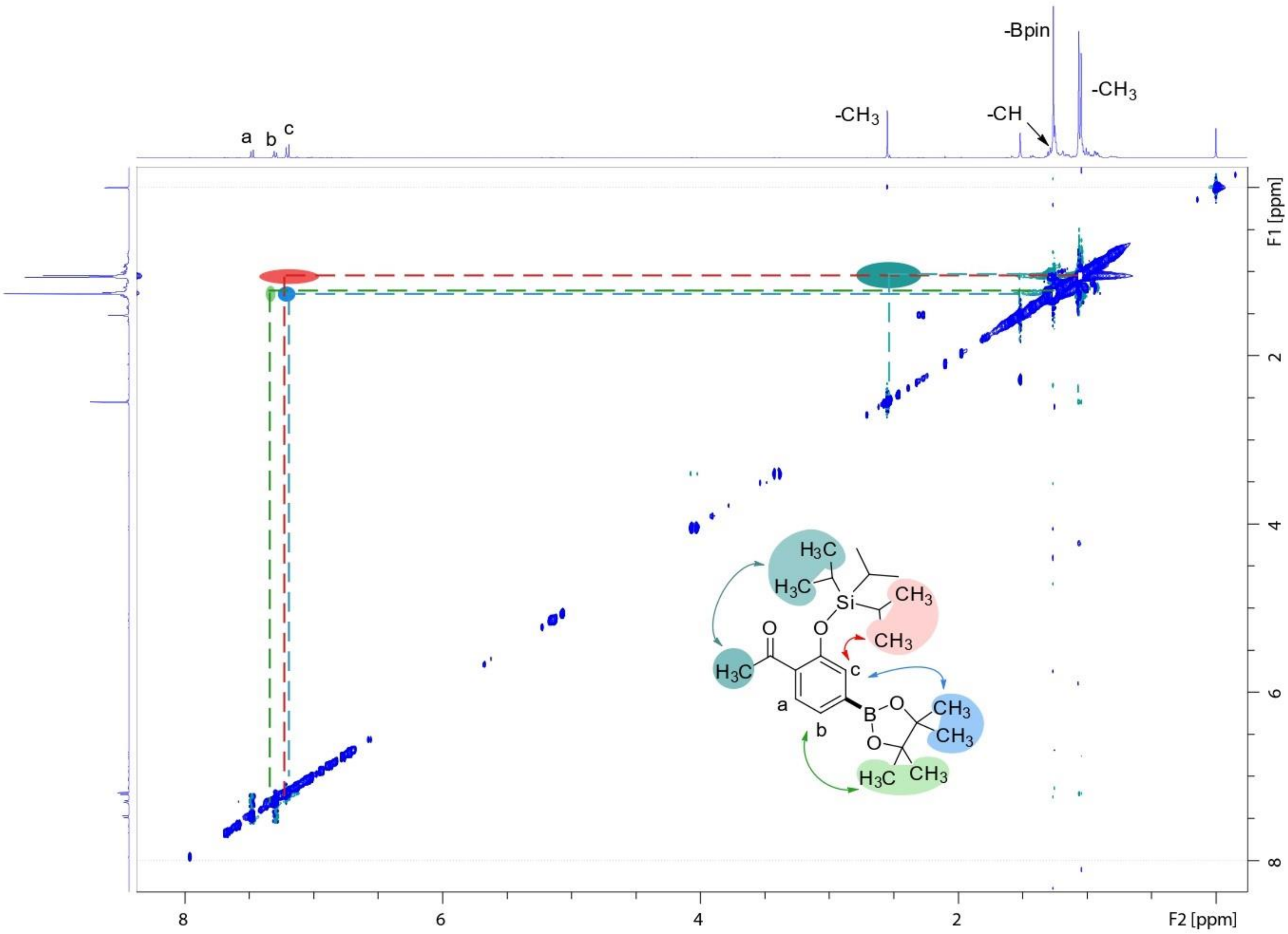

NOESY-NMR spectra of **5p** (25 ^{o}C, 100 MHz, $CDCl_3$)

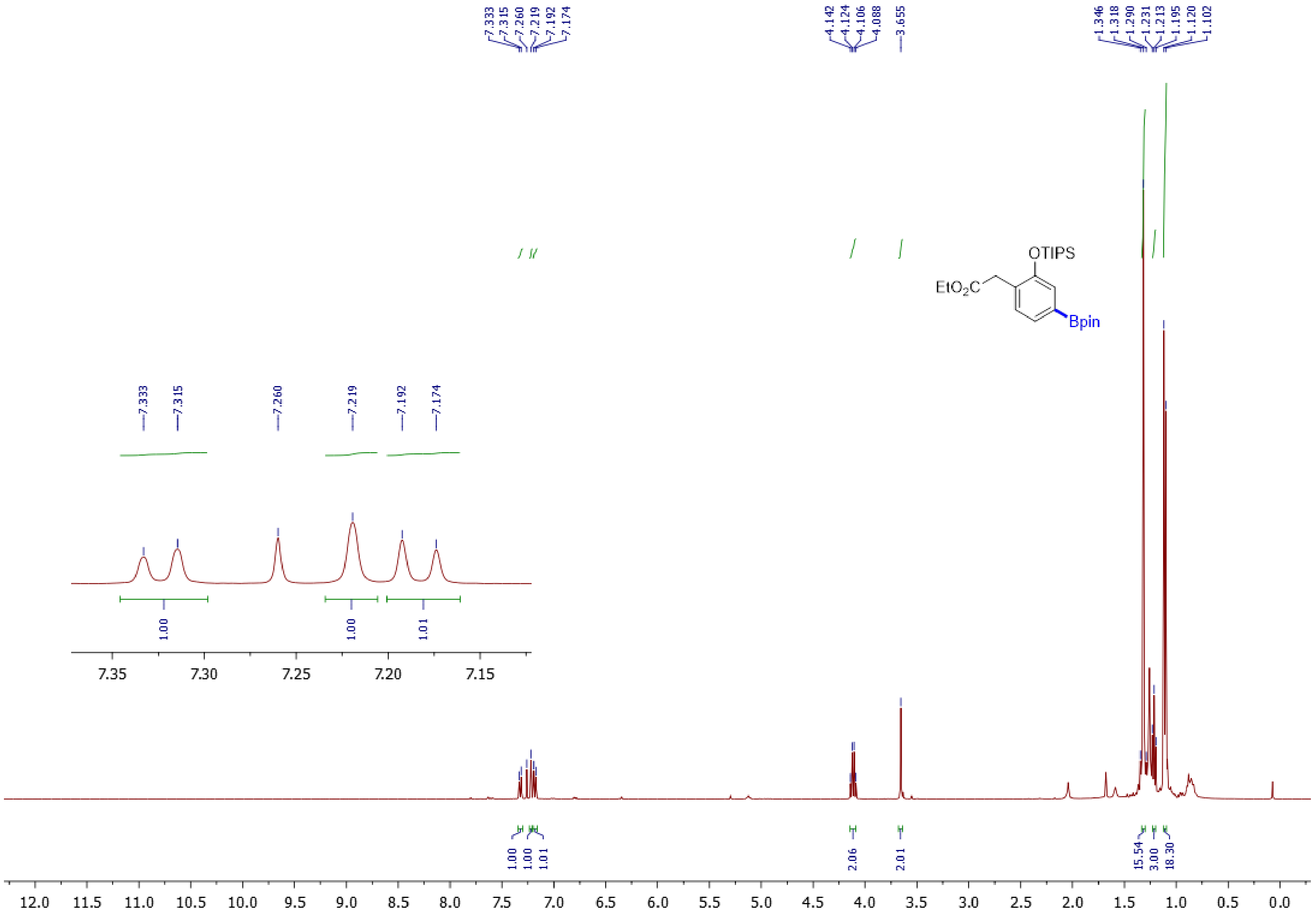

^{1}H-NMR spectra of **5q** (25 °C, 400 MHz, $CDCl_3$)

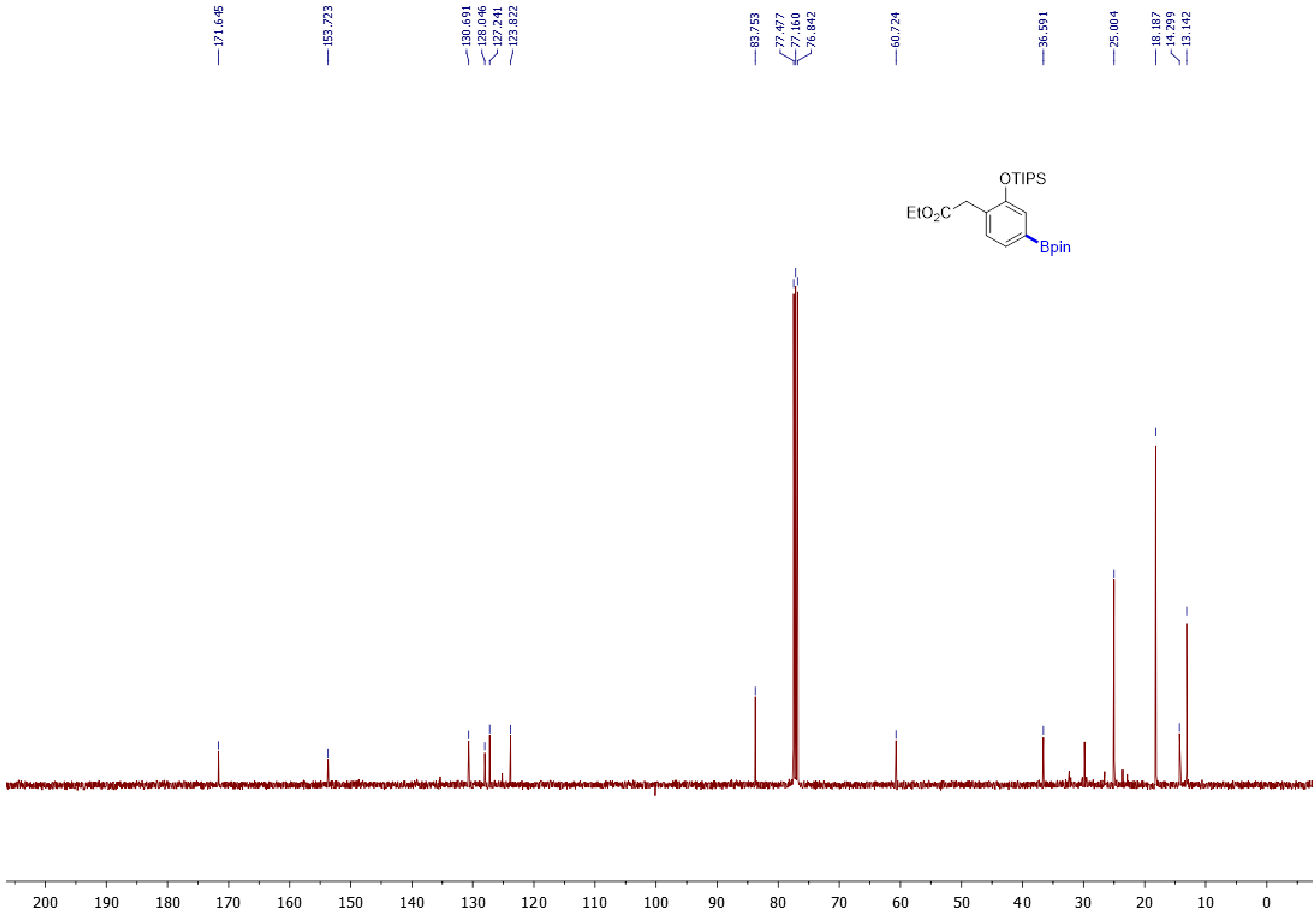

^{13}C-NMR spectra of **5q** (25 °C, 100 MHz, $CDCl_3$)

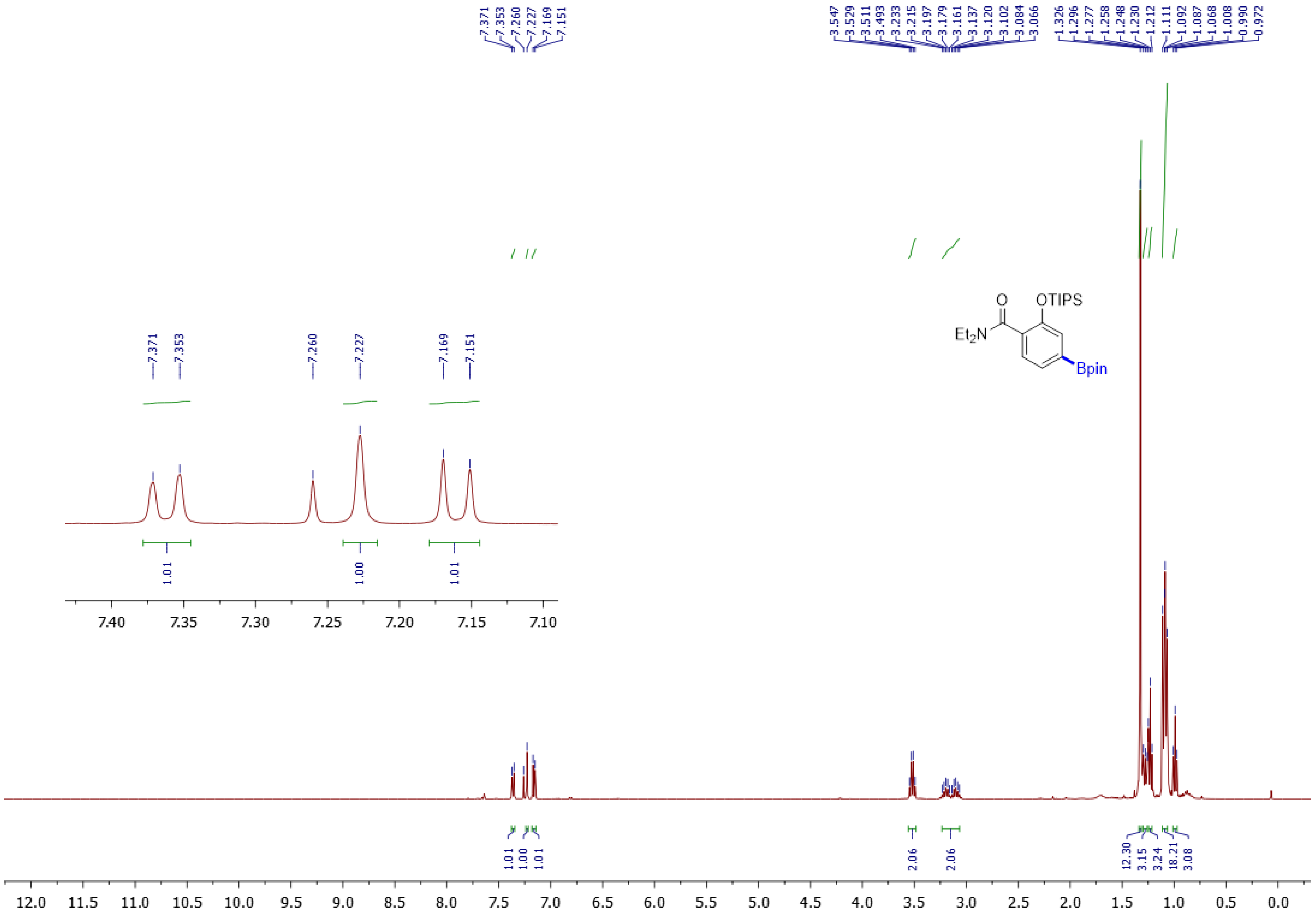

^{1}H-NMR spectra of **5r** (25 ^{o}C, 400 MHz, $CDCl_3$)

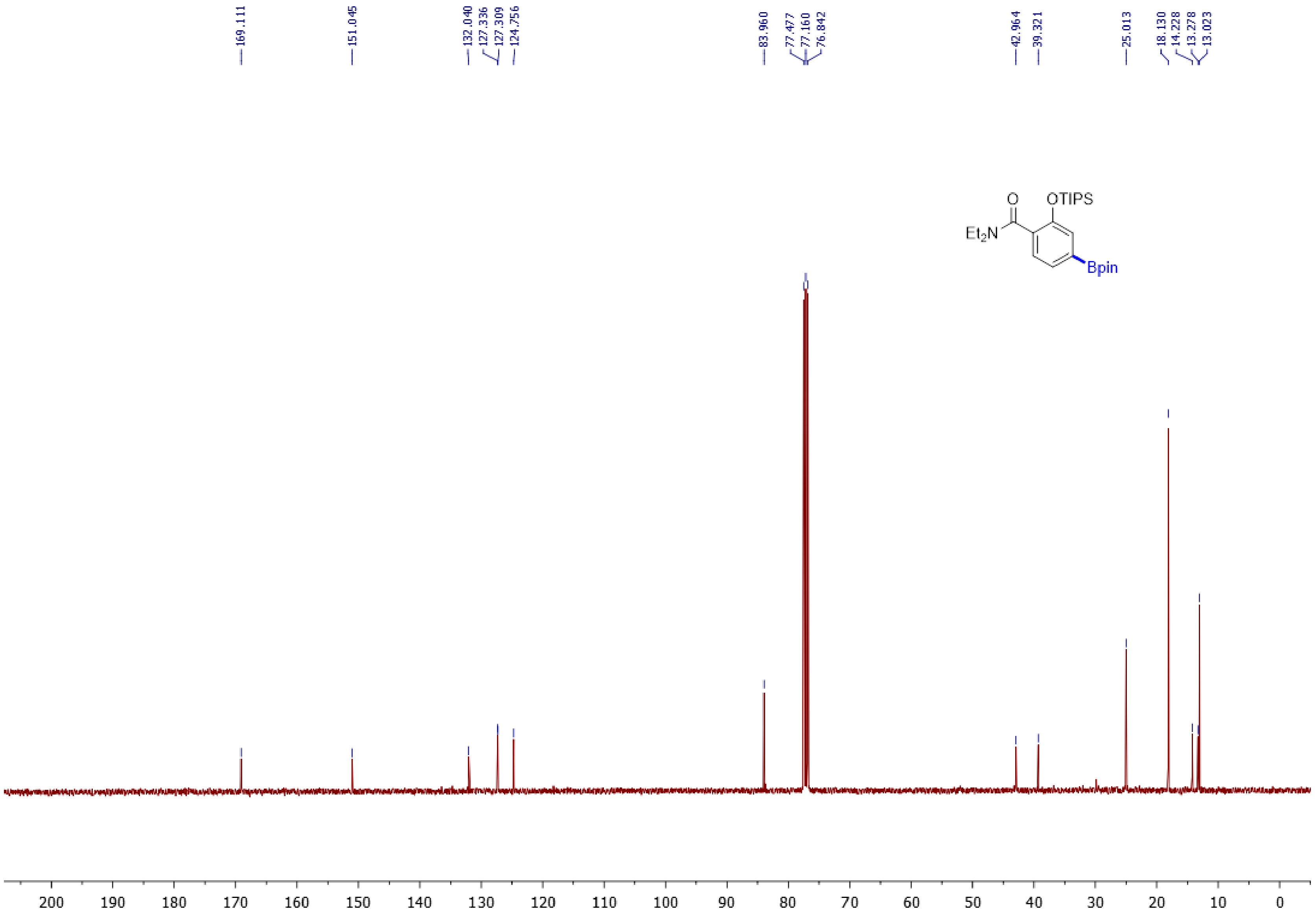

^{13}C-NMR spectra of **5r** (25 °C, 100 MHz, $CDCl_3$)

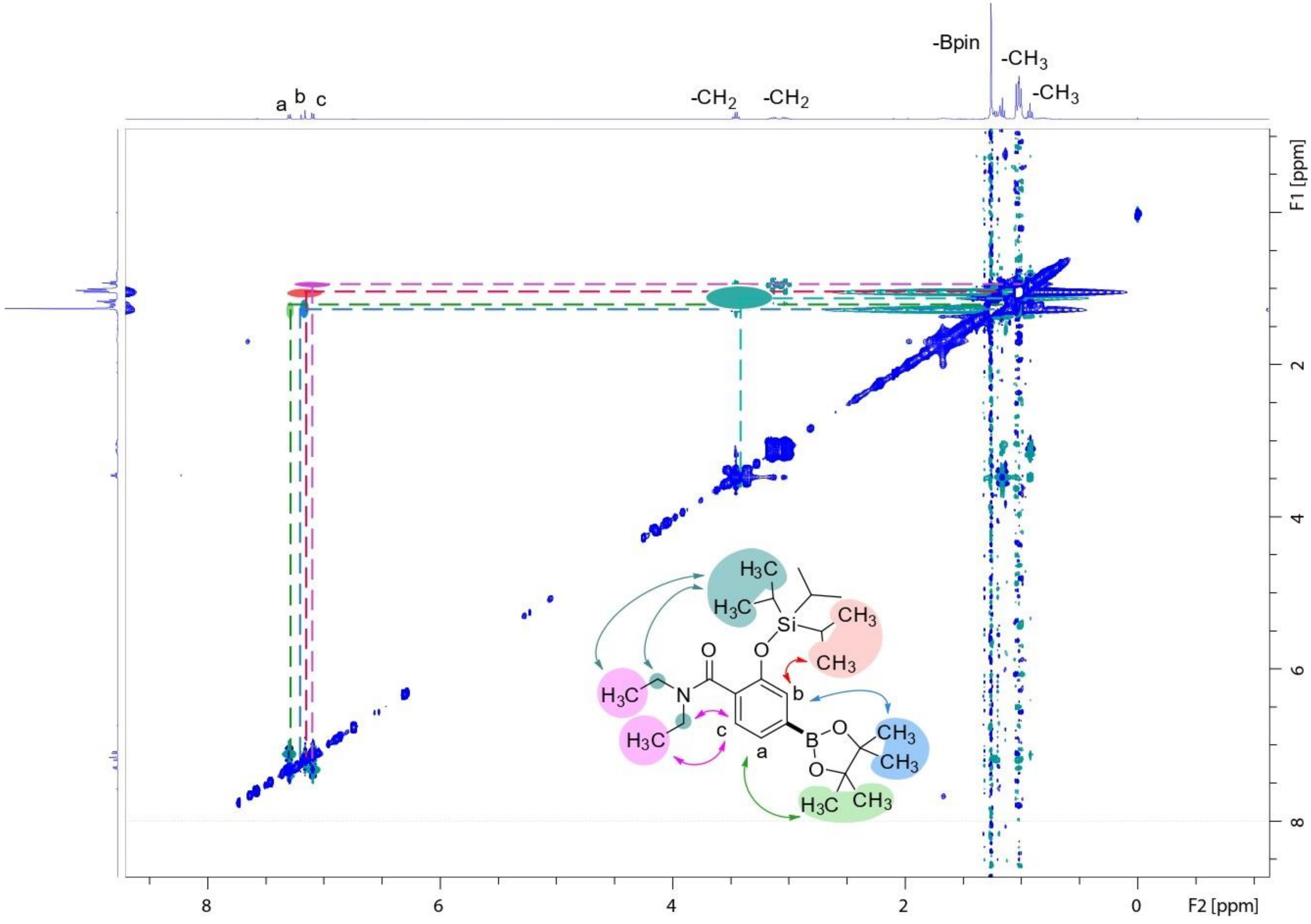

NOESY-NMR spectra of **5r** (25 ºC, 100 MHz, $CDCl_3$)

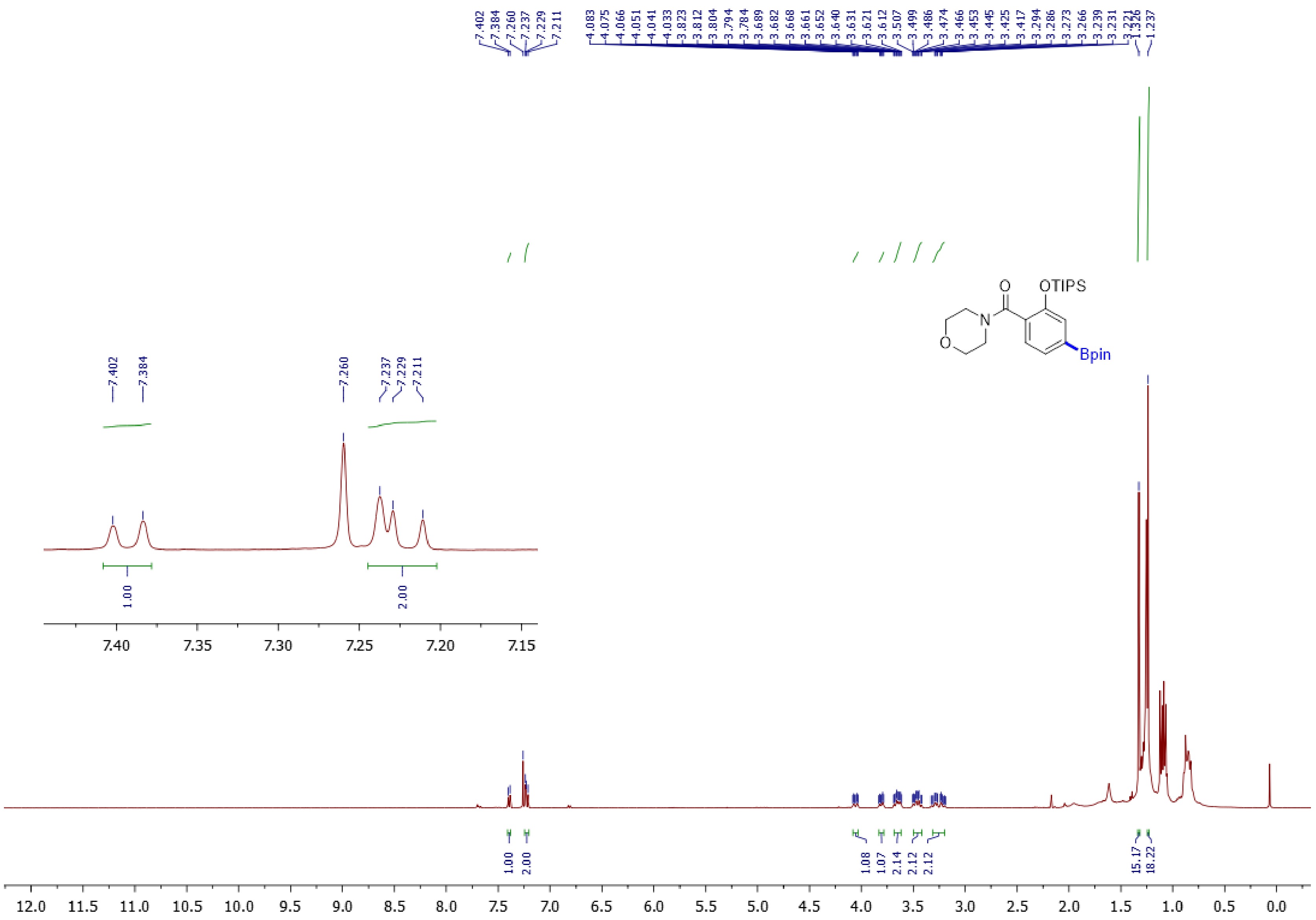

^{1}H-NMR spectra of **5s** (25 ^{o}C, 400 MHz, $CDCl_3$)

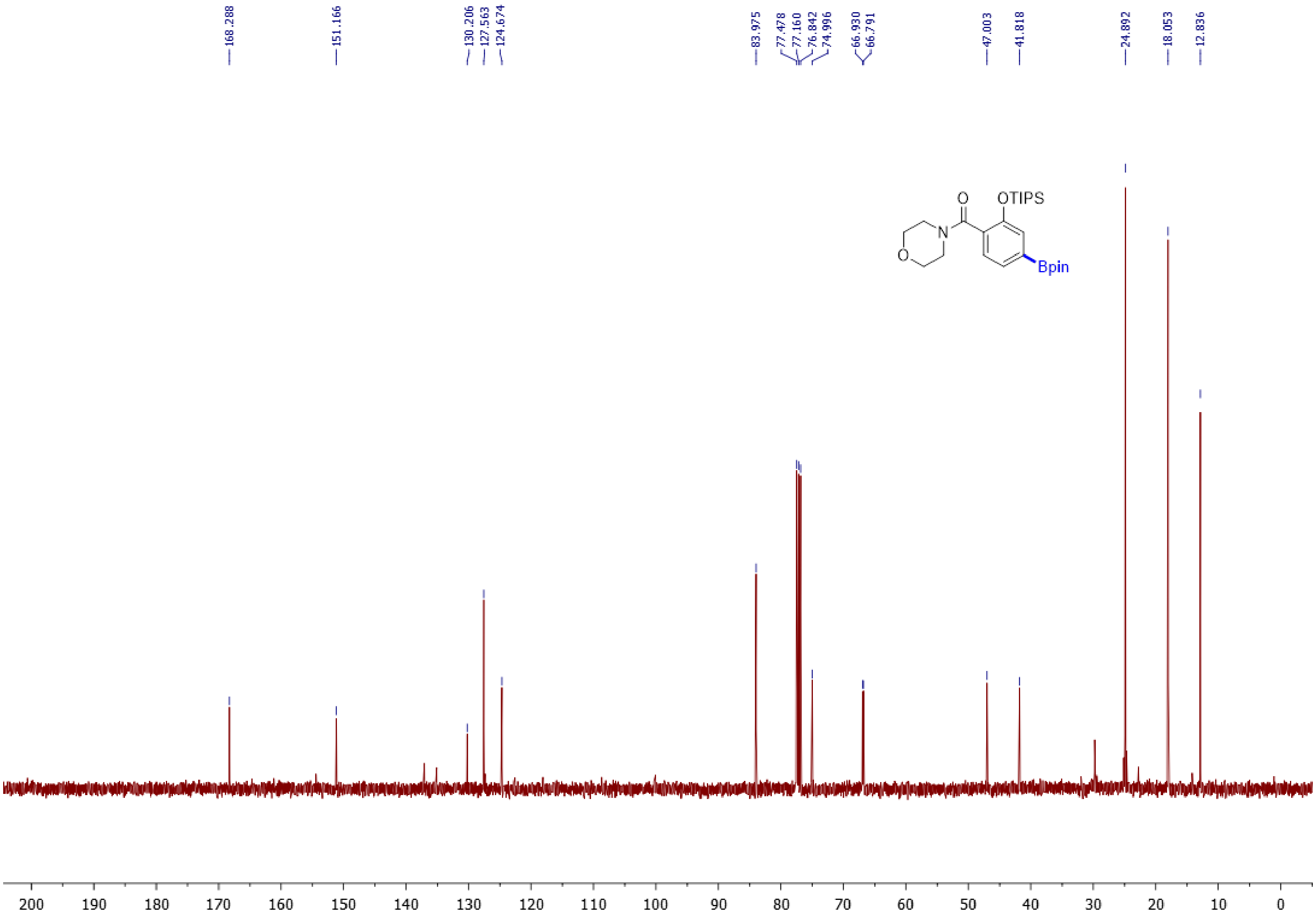

^{13}C-NMR spectra of **5s** (25 ^{o}C, 100 MHz, $CDCl_3$)

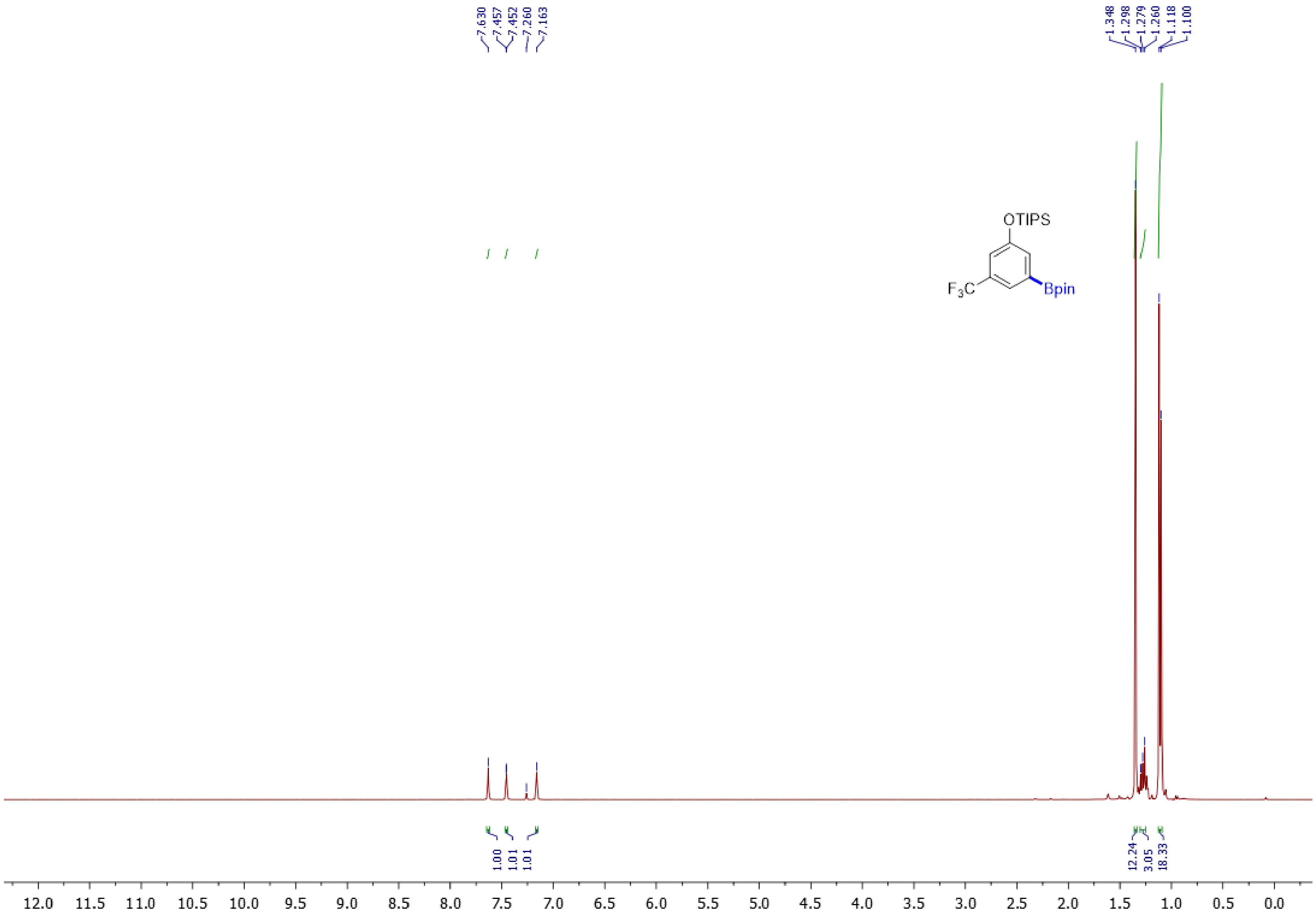

^{1}H-NMR spectra of **5t** (25 ^{o}C, 400 MHz, $CDCl_3$)

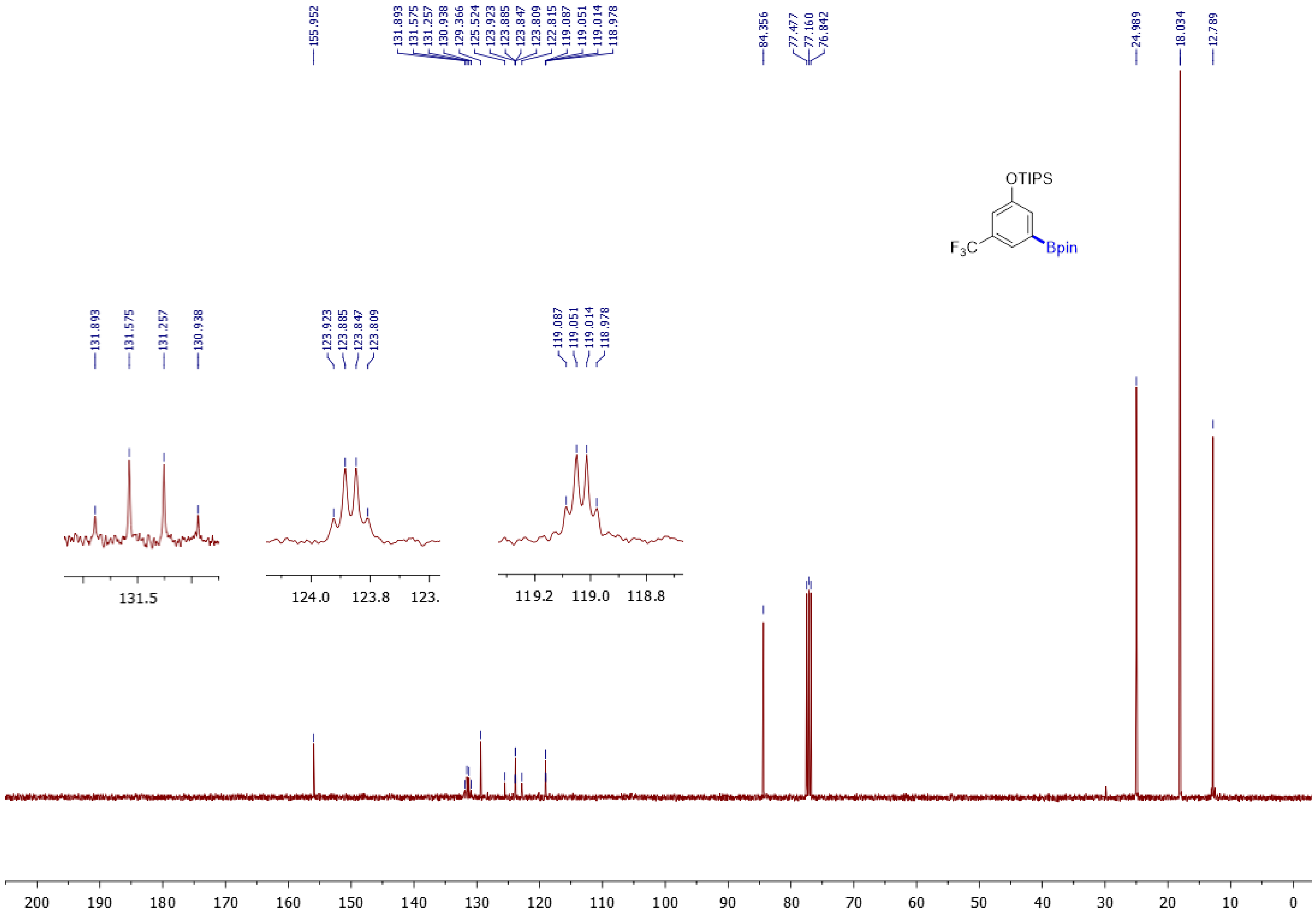

^{13}C-NMR spectra of **5t** (25 °C, 100 MHz, $CDCl_3$)

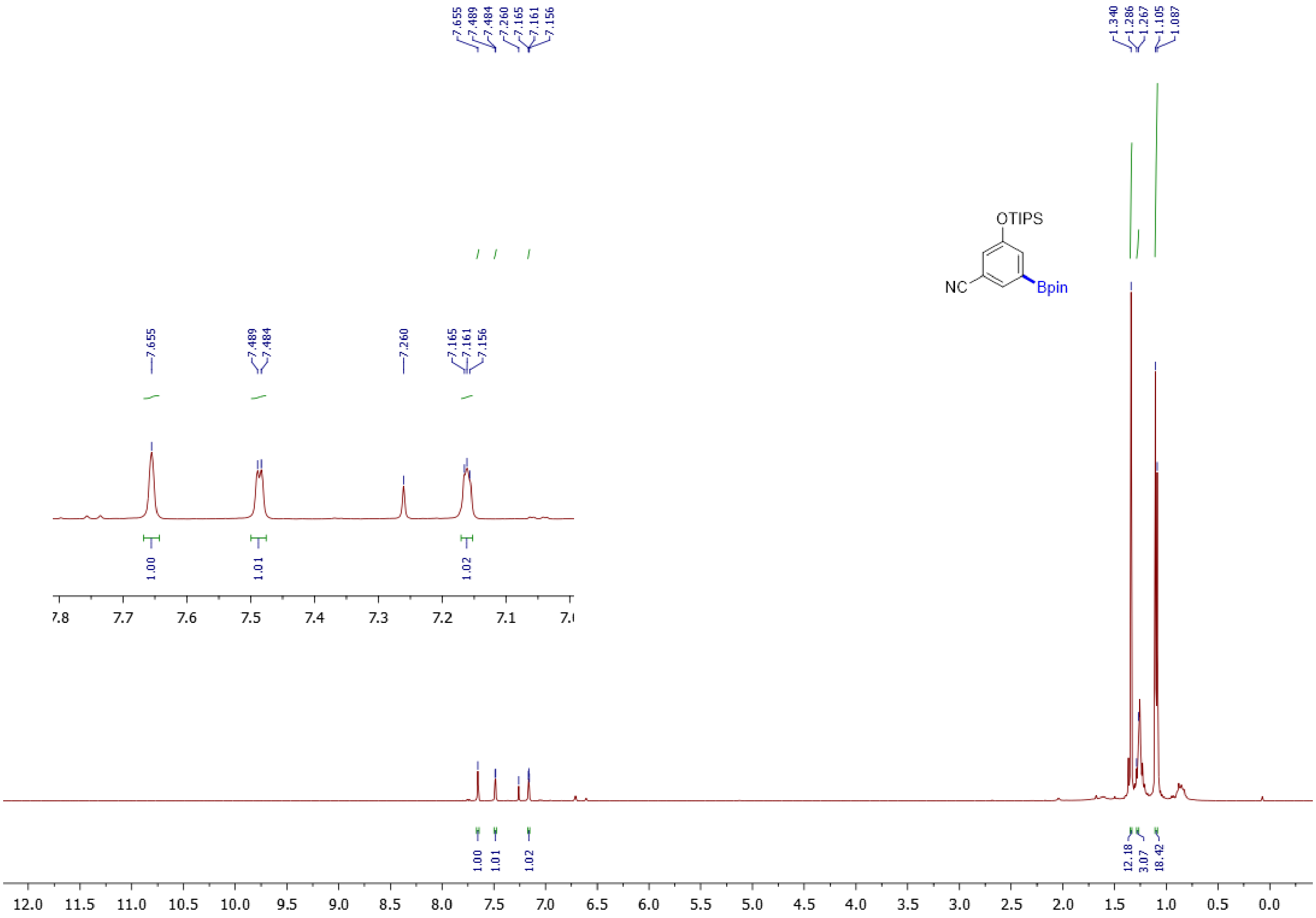

^{1}H-NMR spectra of **5u** (25 ^{o}C, 400 MHz, $CDCl_3$)

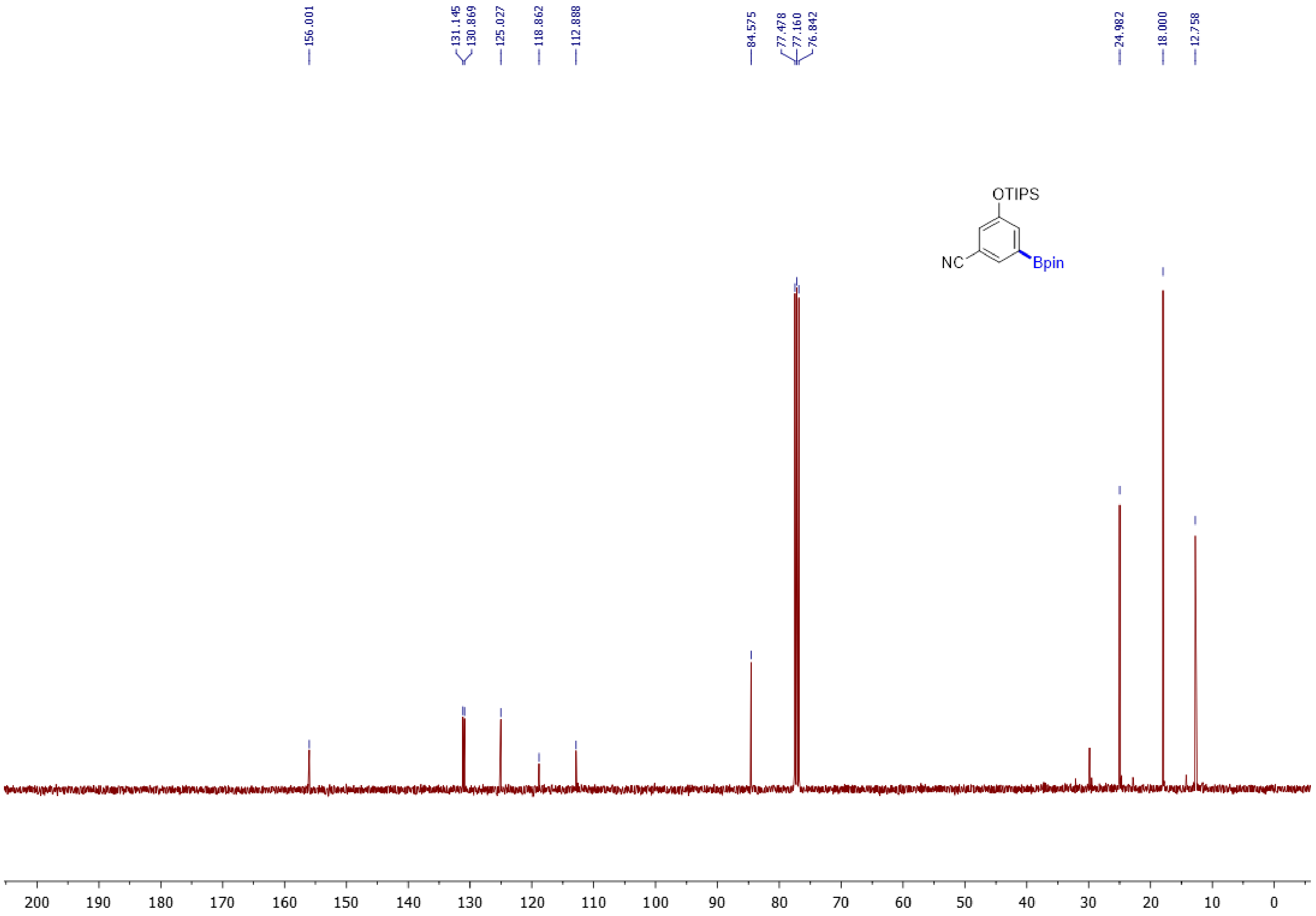

^{13}C-NMR spectra of **5u** (25 °C, 100 MHz, $CDCl_3$)

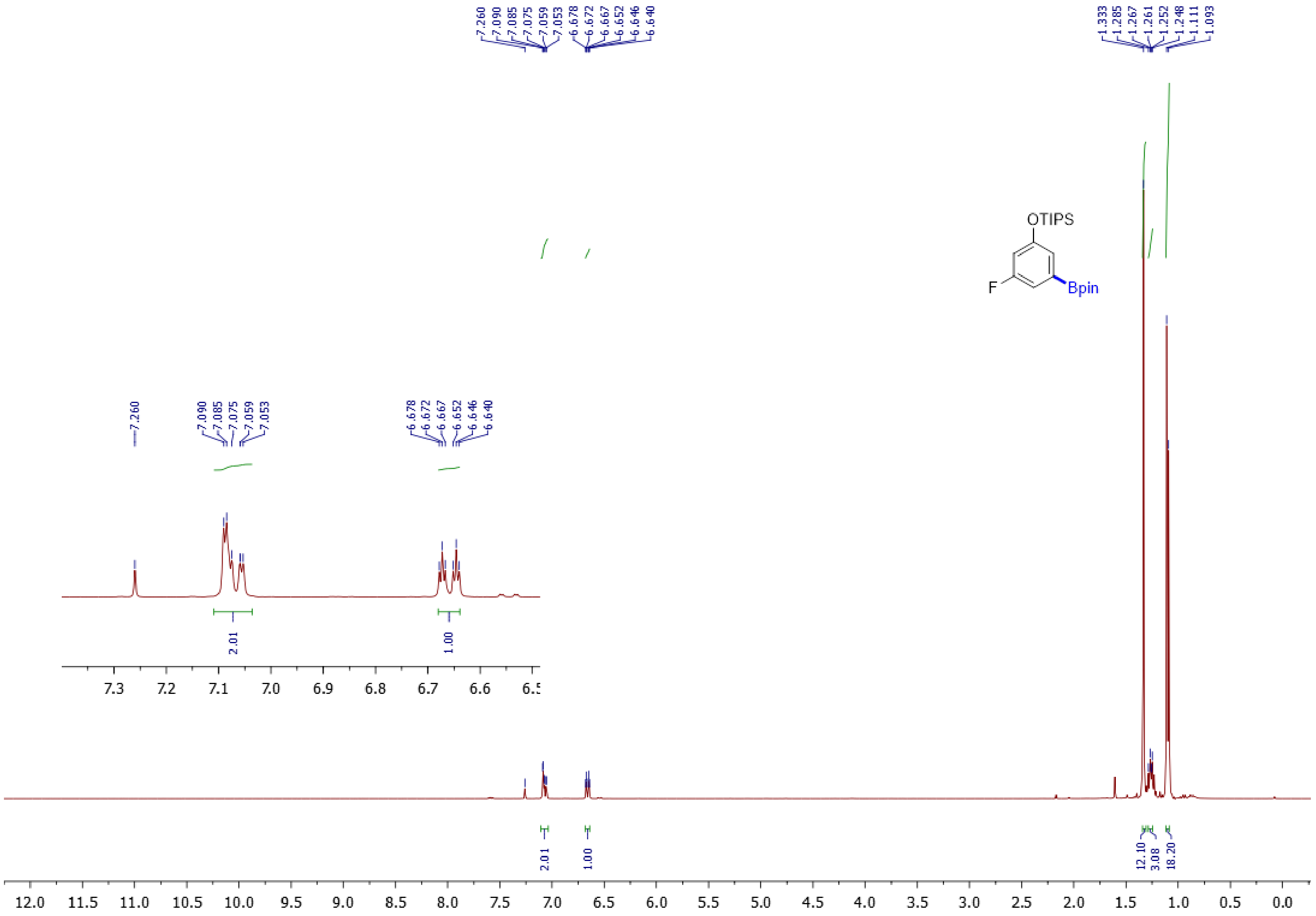

^{1}H-NMR spectra of **5v** (25 °C, 400 MHz, $CDCl_3$)

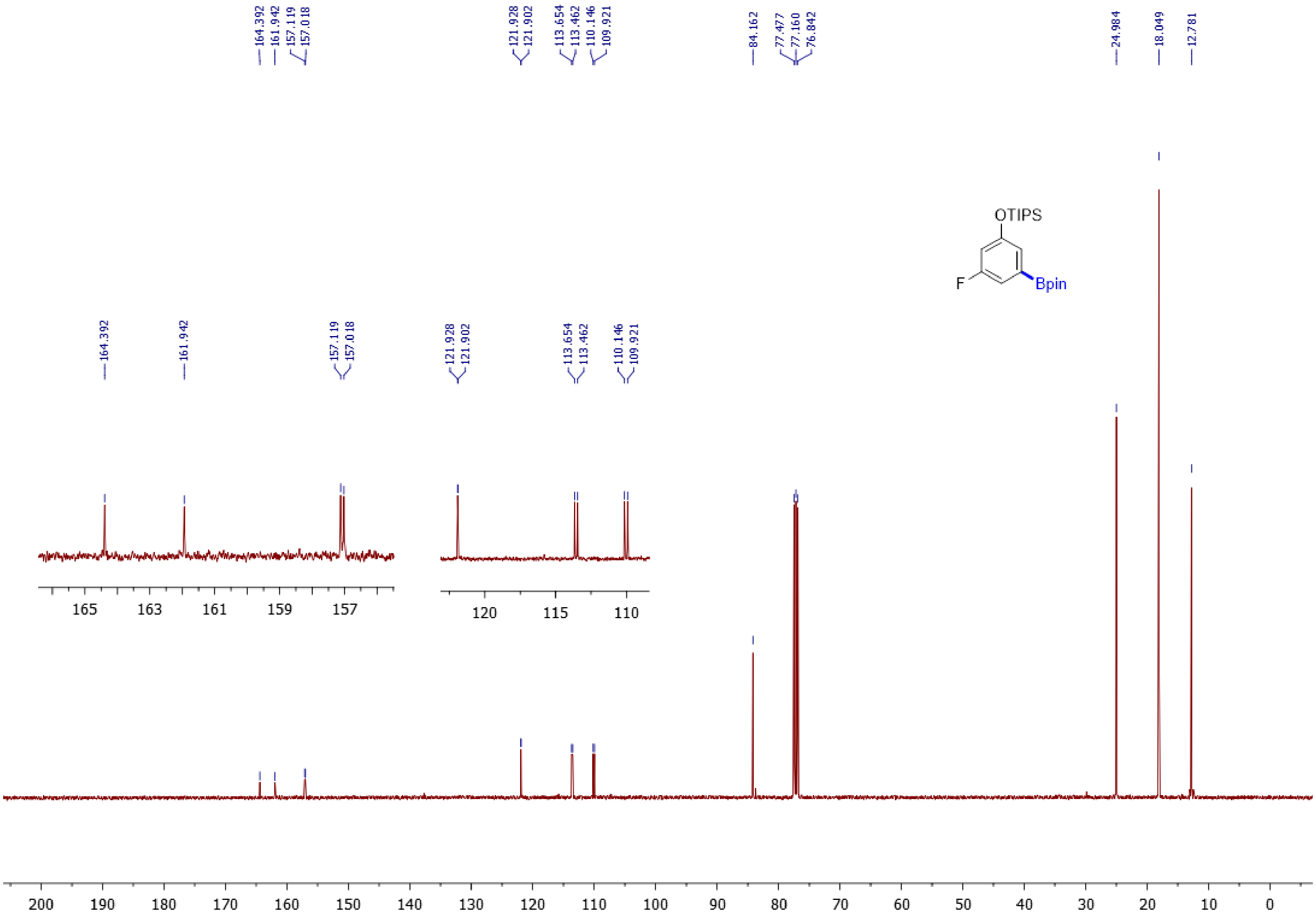

^{13}C-NMR spectra of **5v** (25 °C, 100 MHz, $CDCl_3$)

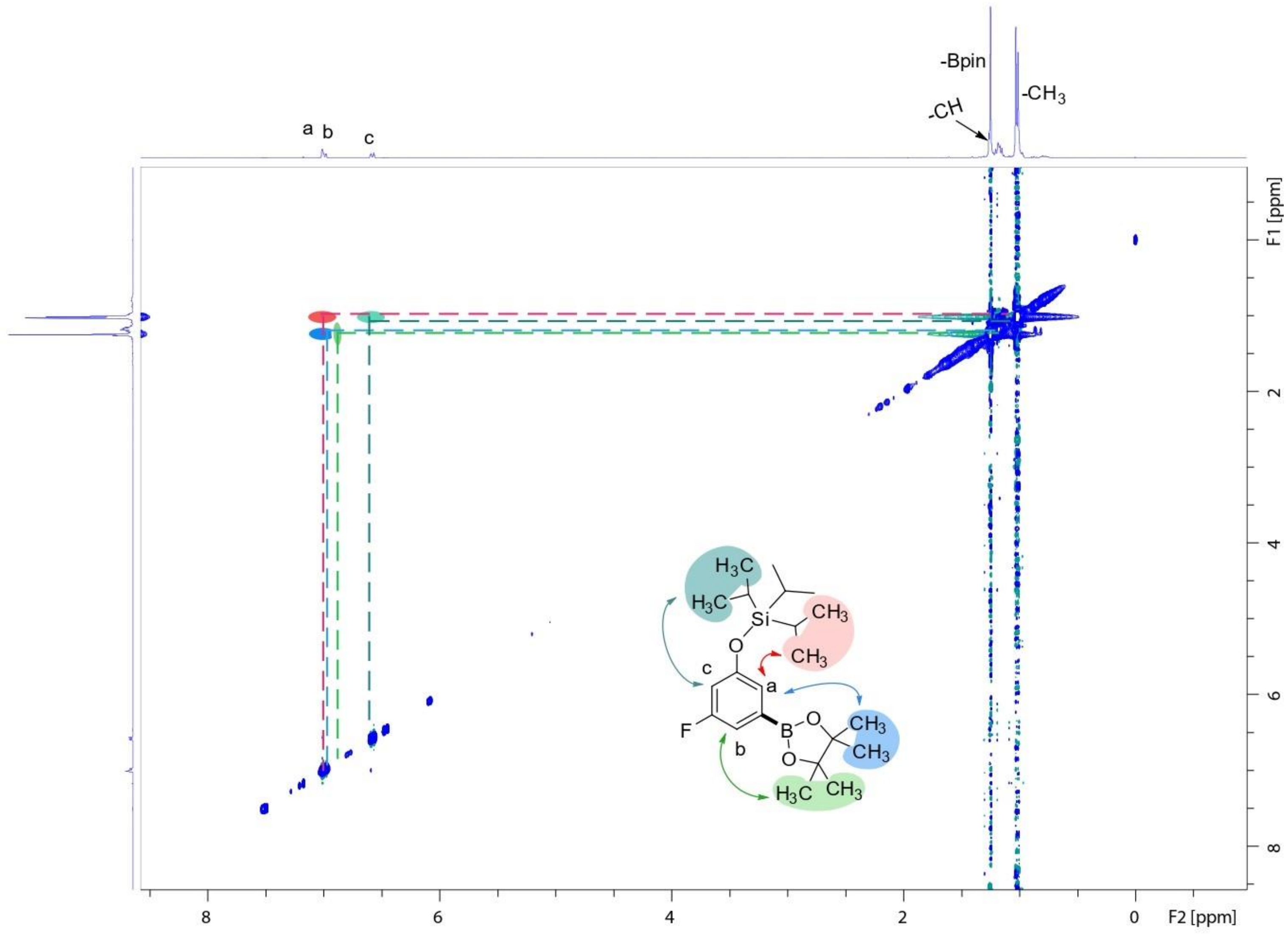

NOESY-NMR spectra of **5v** (25 °C, 100 MHz, $CDCl_3$)

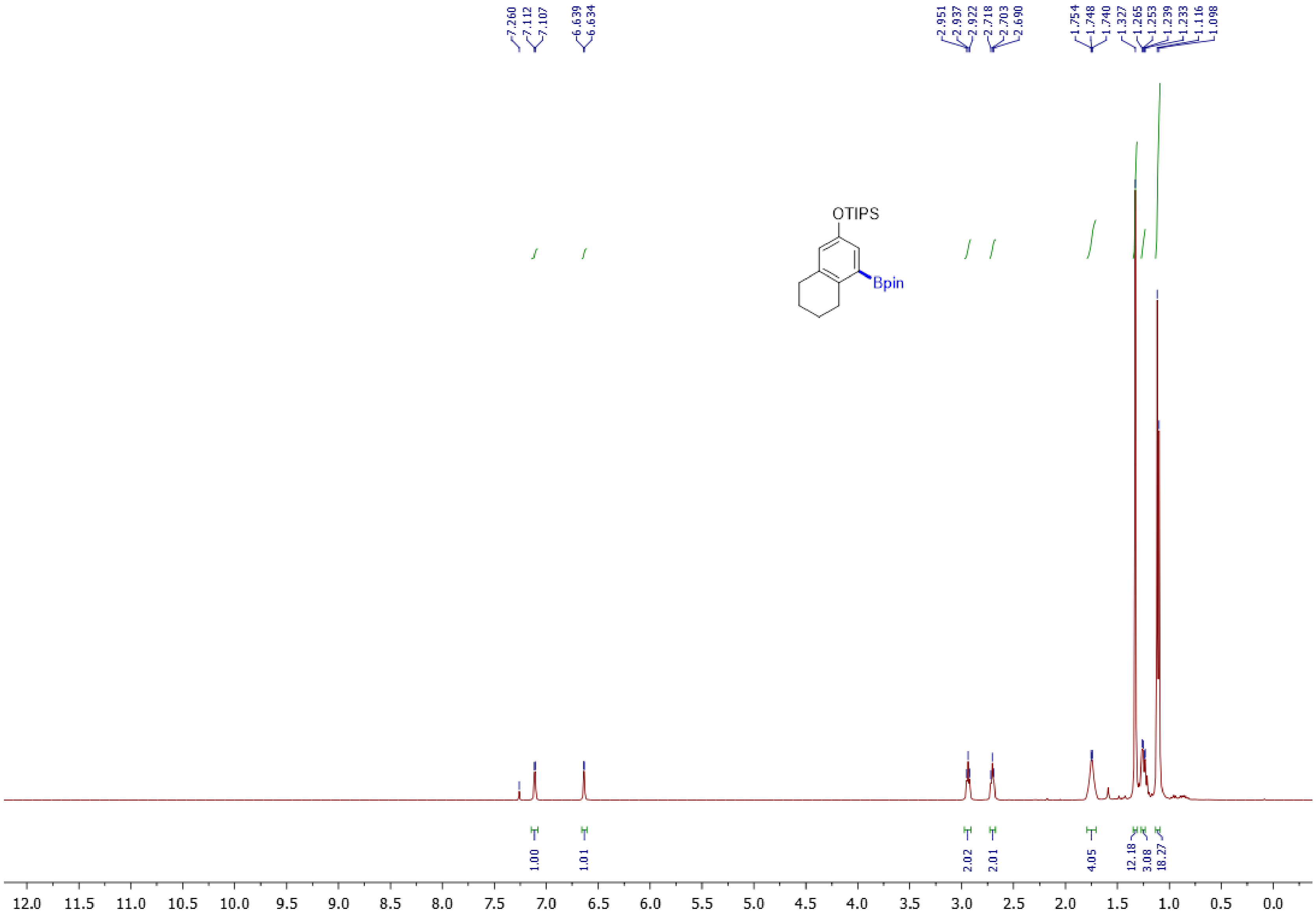

^{1}H-NMR spectra of **5w** (25 °C, 400 MHz, $CDCl_3$)

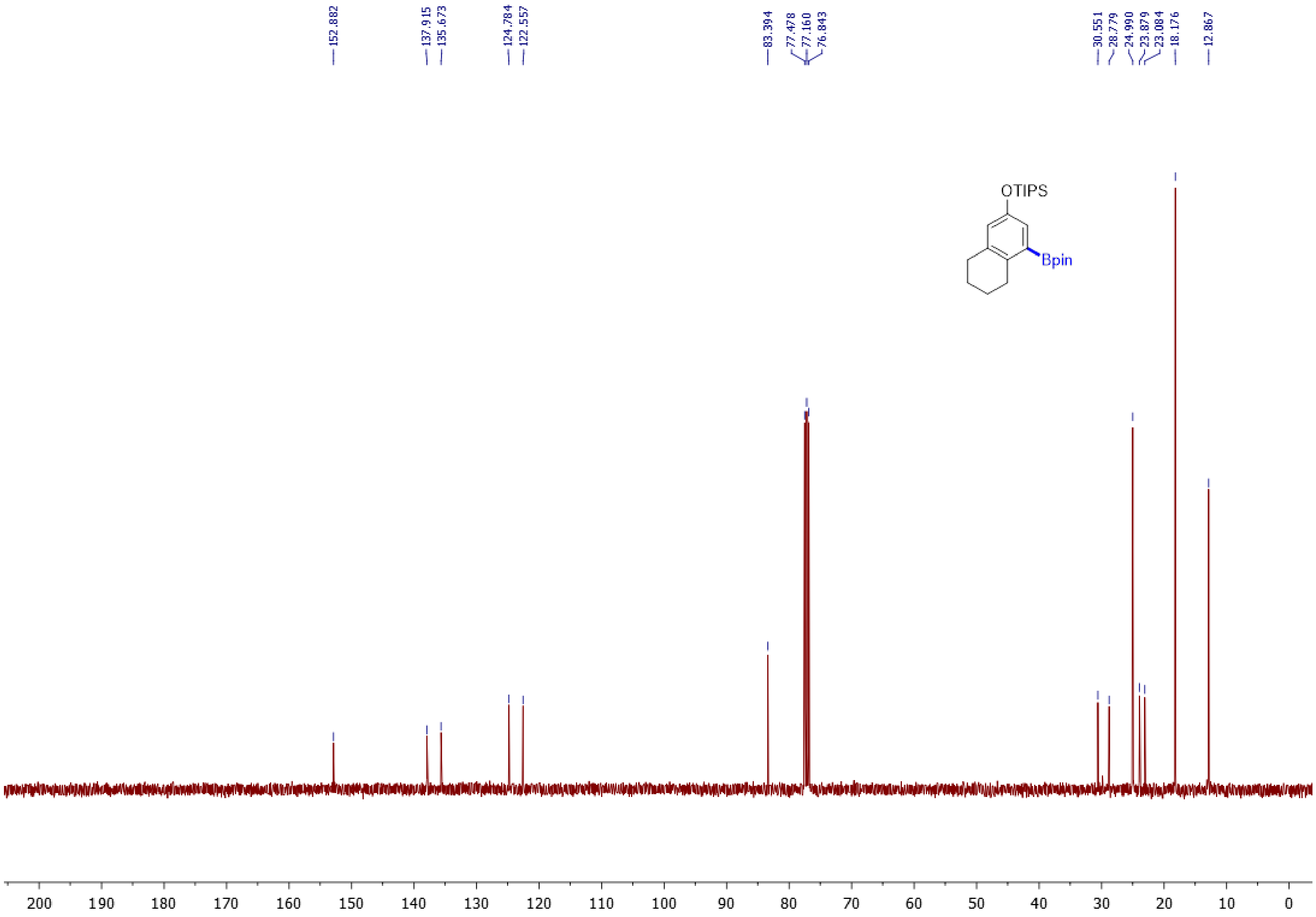

^{13}C-NMR spectra of **5w** (25 ^{o}C, 100 MHz, $CDCl_3$)

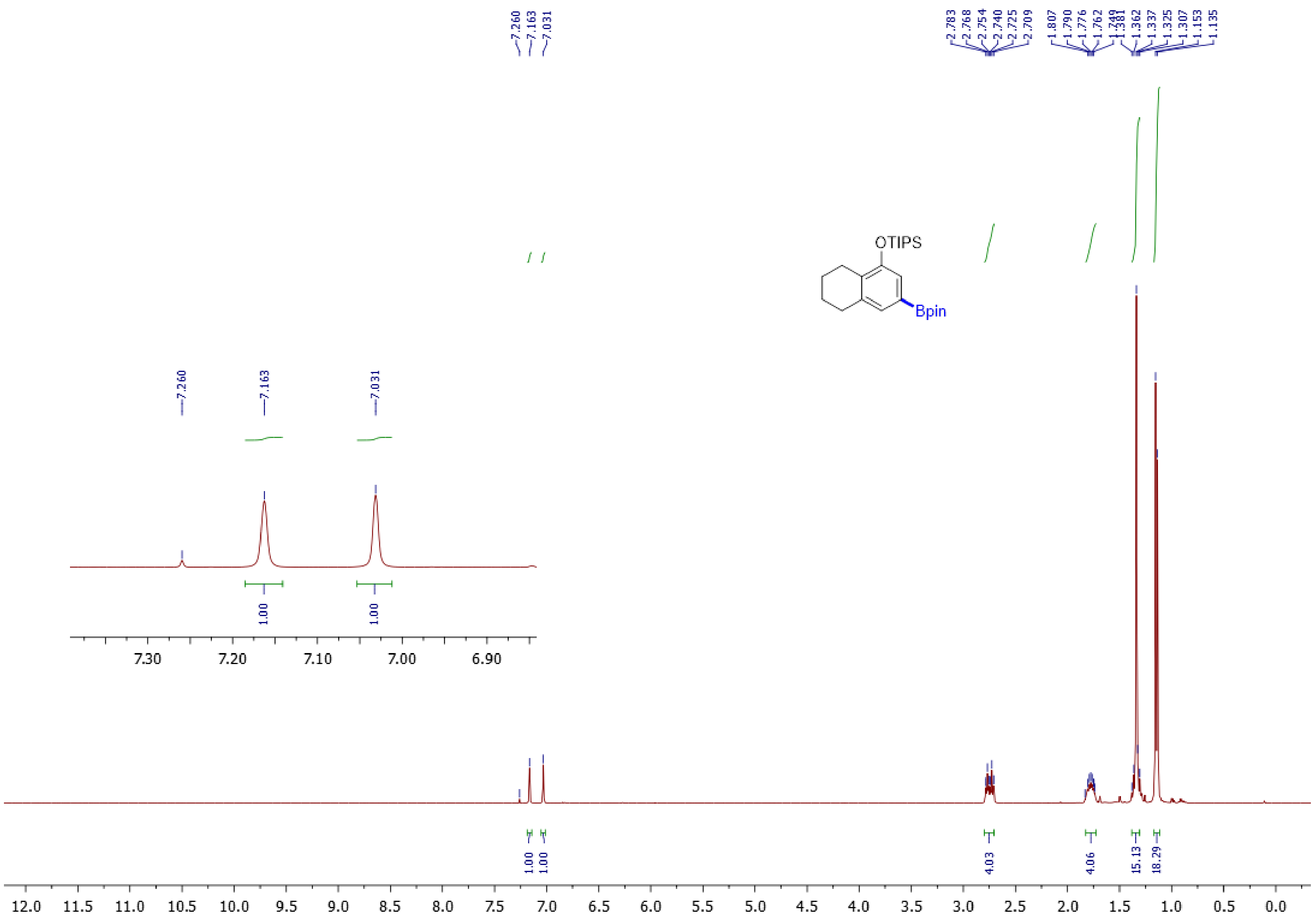

^{1}H-NMR spectra of **5x** (25 °C, 400 MHz, $CDCl_3$)

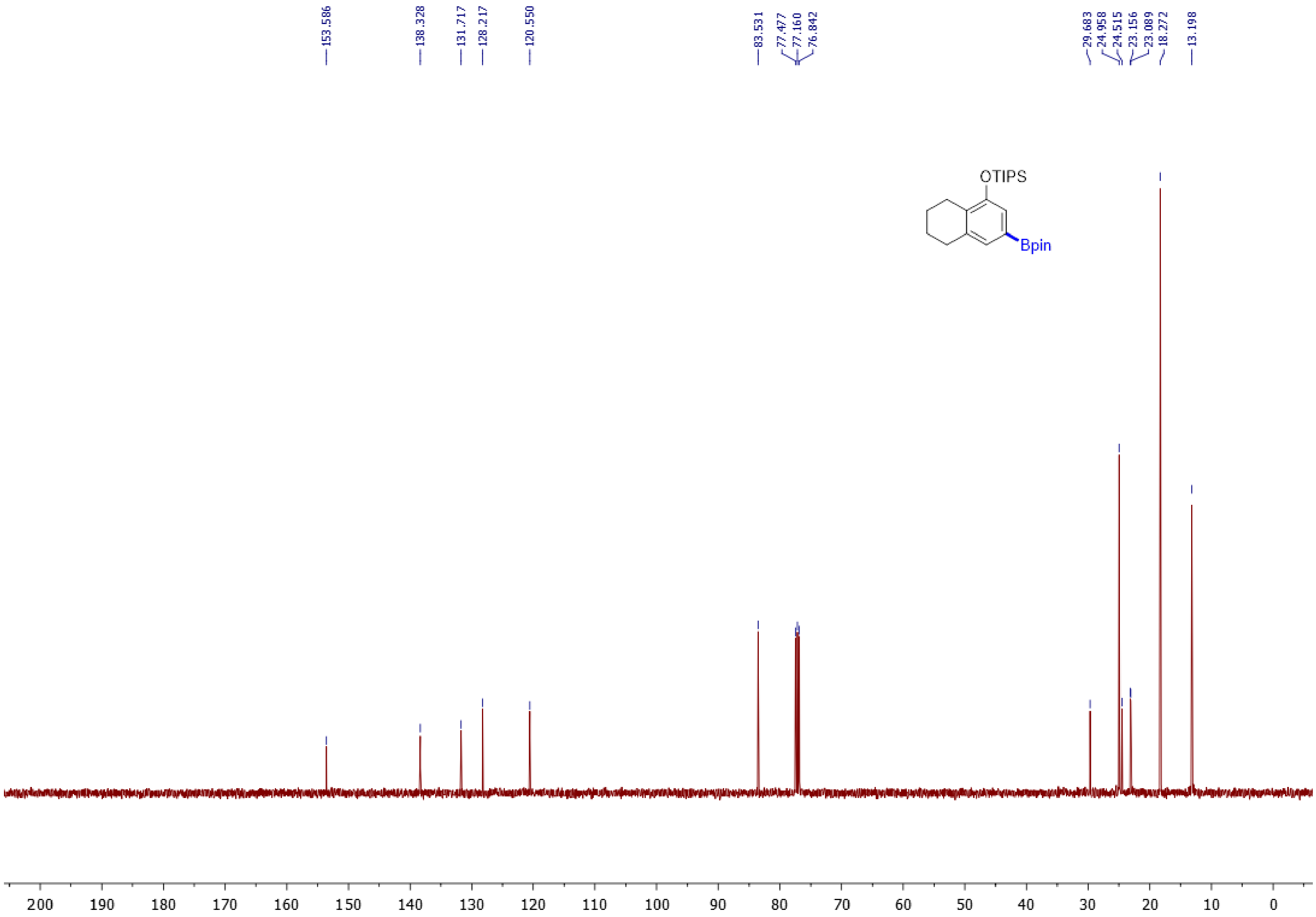

^{13}C-NMR spectra of **5x** (25 °C, 100 MHz, $CDCl_3$)

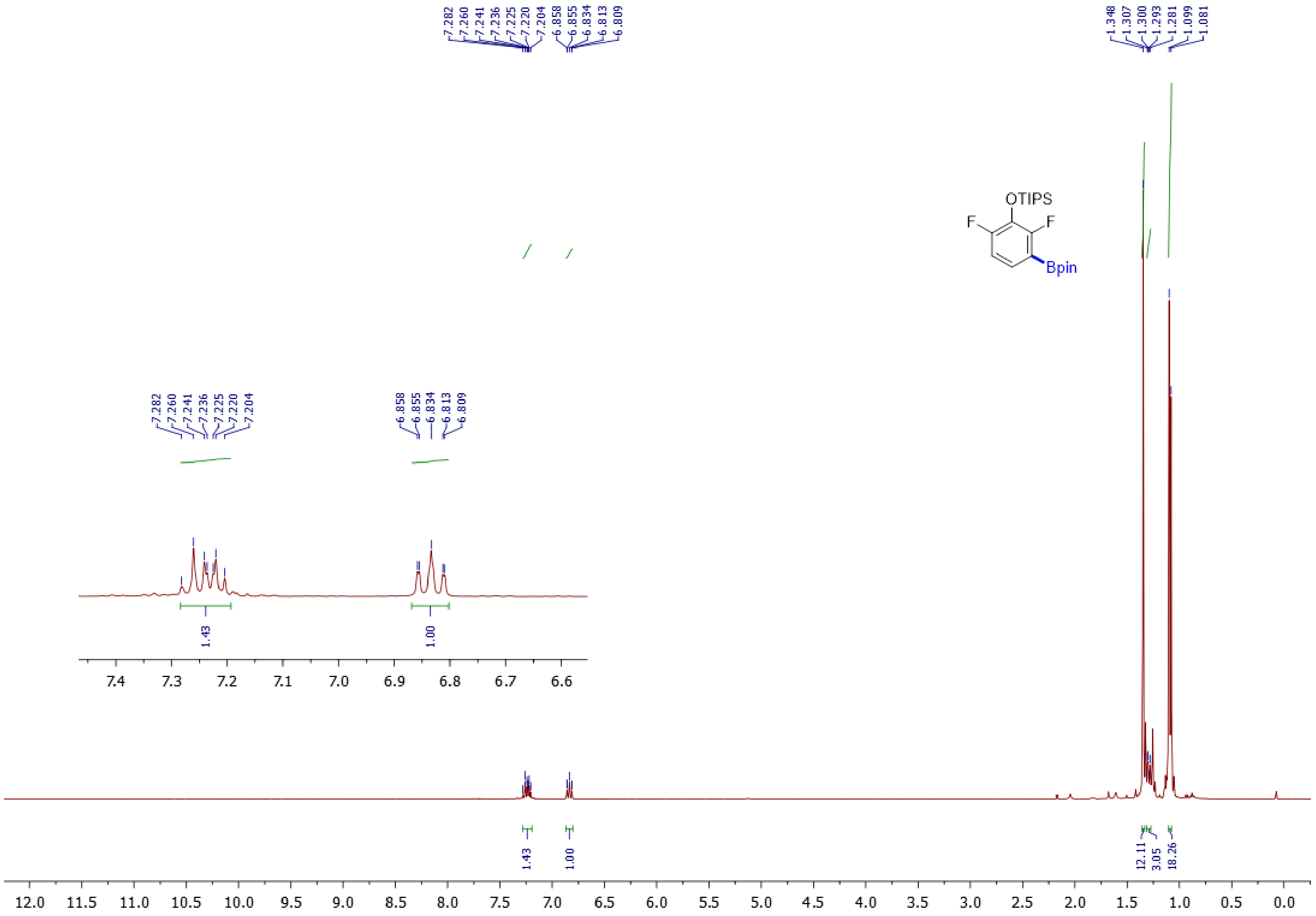

^{1}H-NMR spectra of **5y** (25 ^{o}C, 400 MHz, $CDCl_3$)

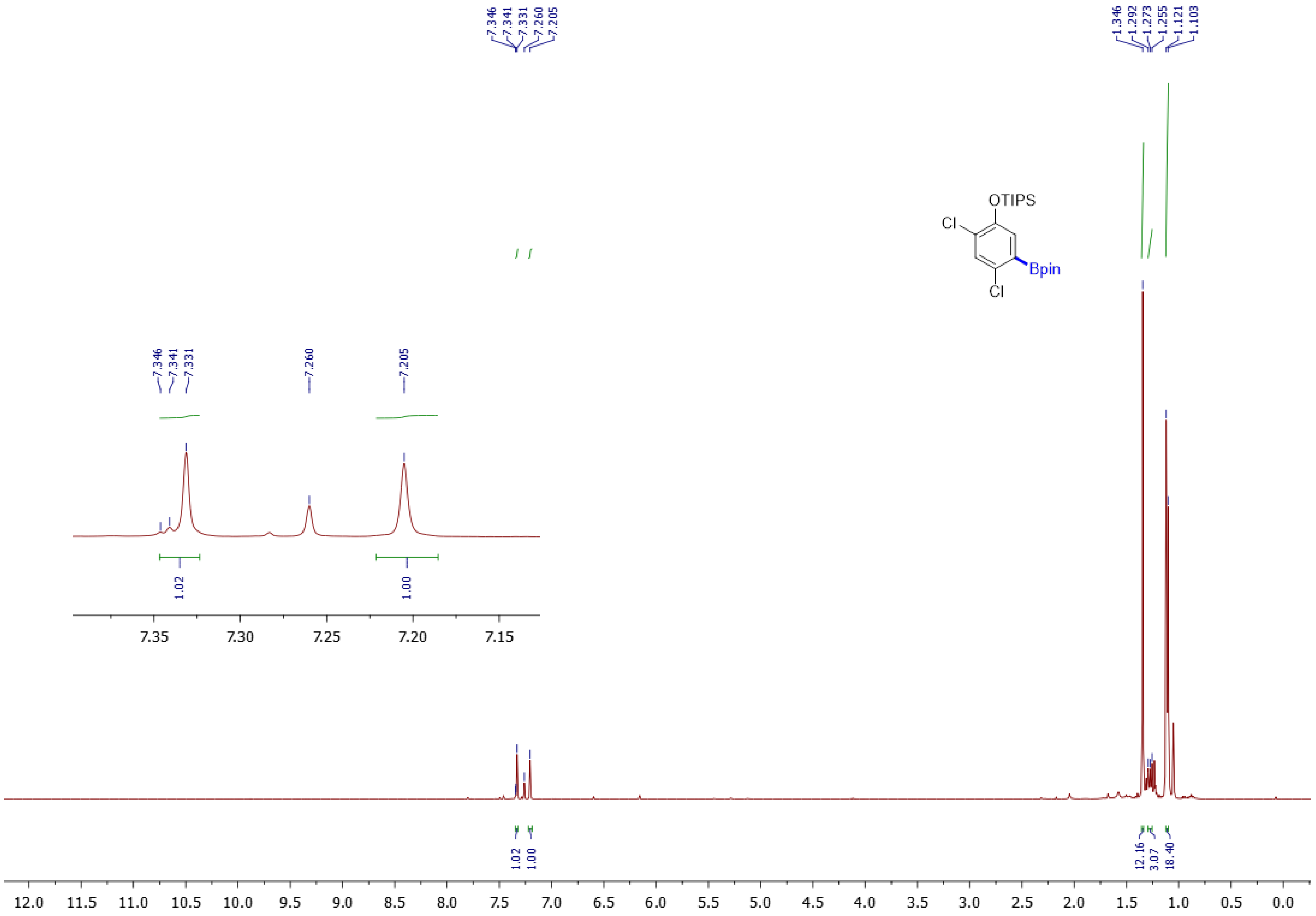

^{1}H-NMR spectra of **5z** (25 ^{o}C, 400 MHz, $CDCl_3$)

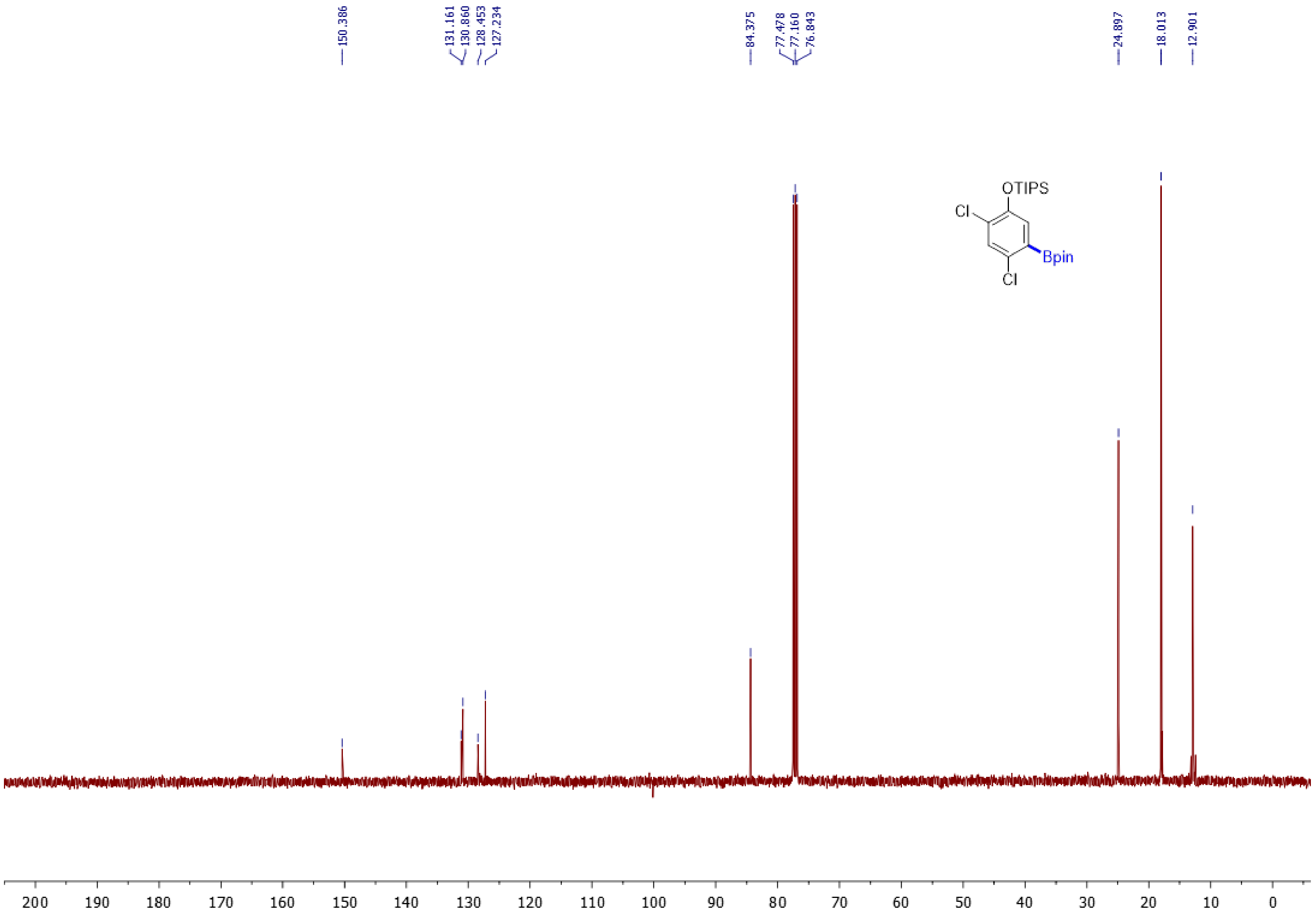

^{13}C-NMR spectra of **5z** (25 °C, 100 MHz, $CDCl_3$)

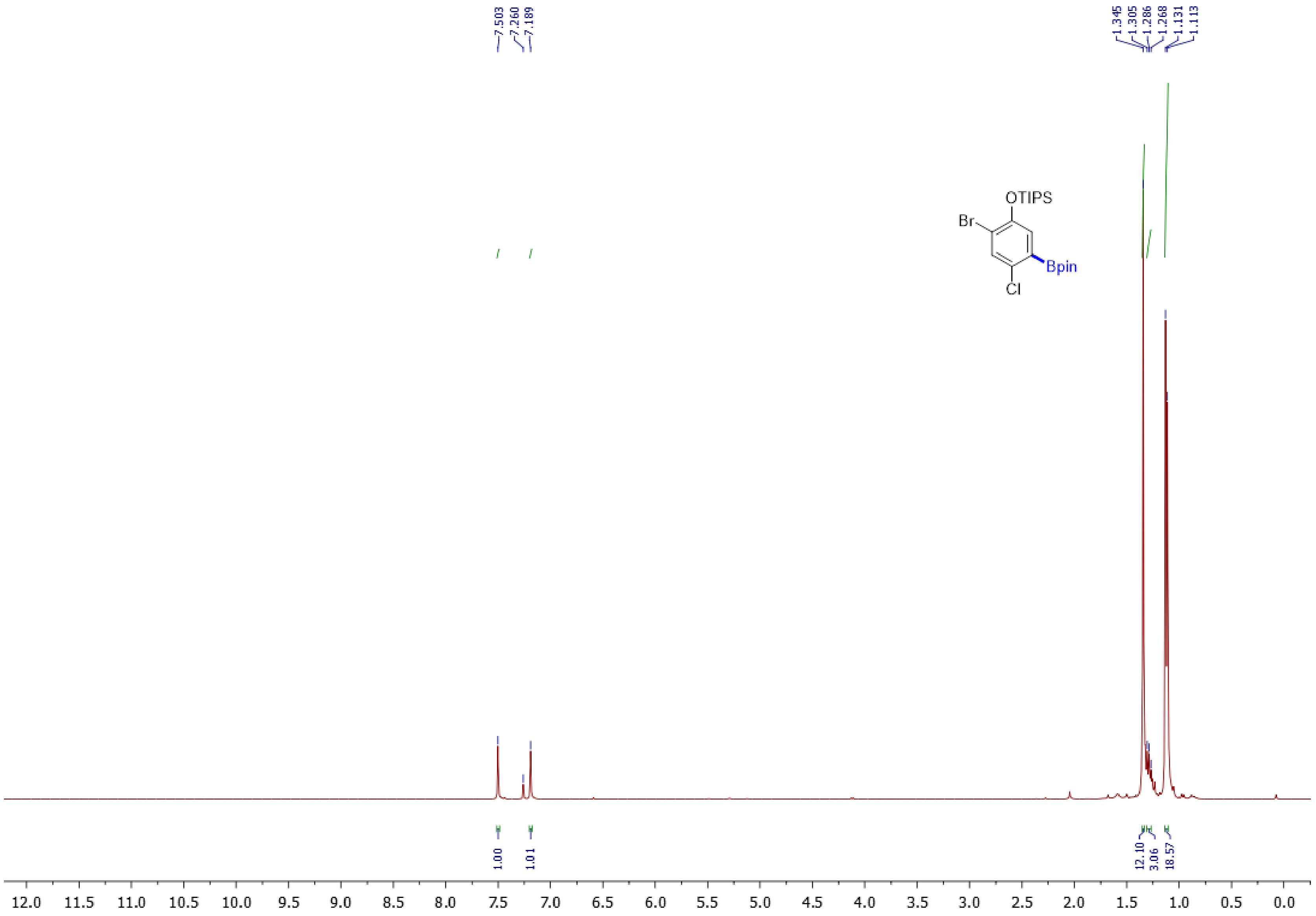

^{1}H-NMR spectra of **5aa** (25 °C, 400 MHz, $CDCl_3$)

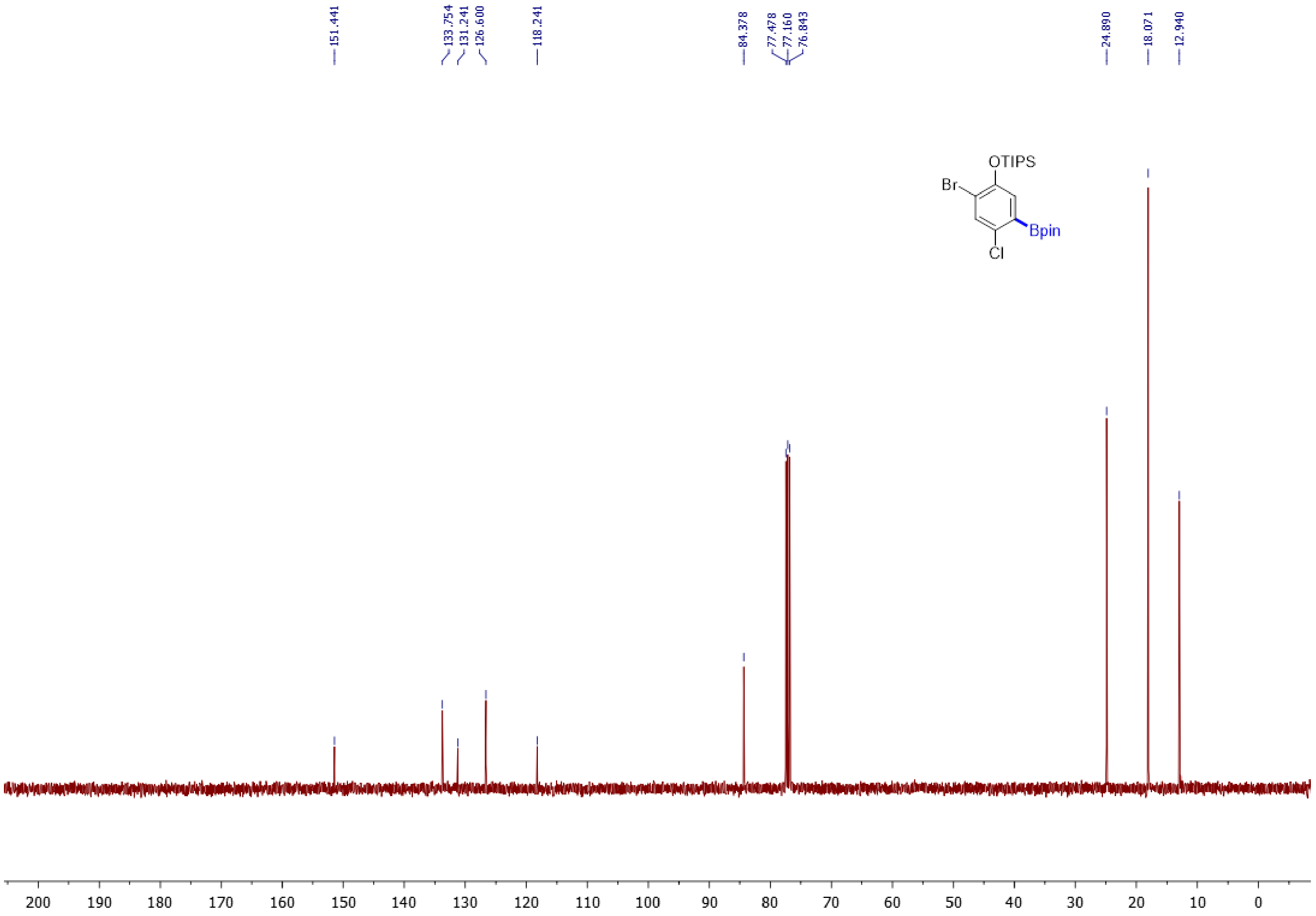

^{13}C-NMR spectra of **5aa** (25 °C, 100 MHz, $CDCl_3$)

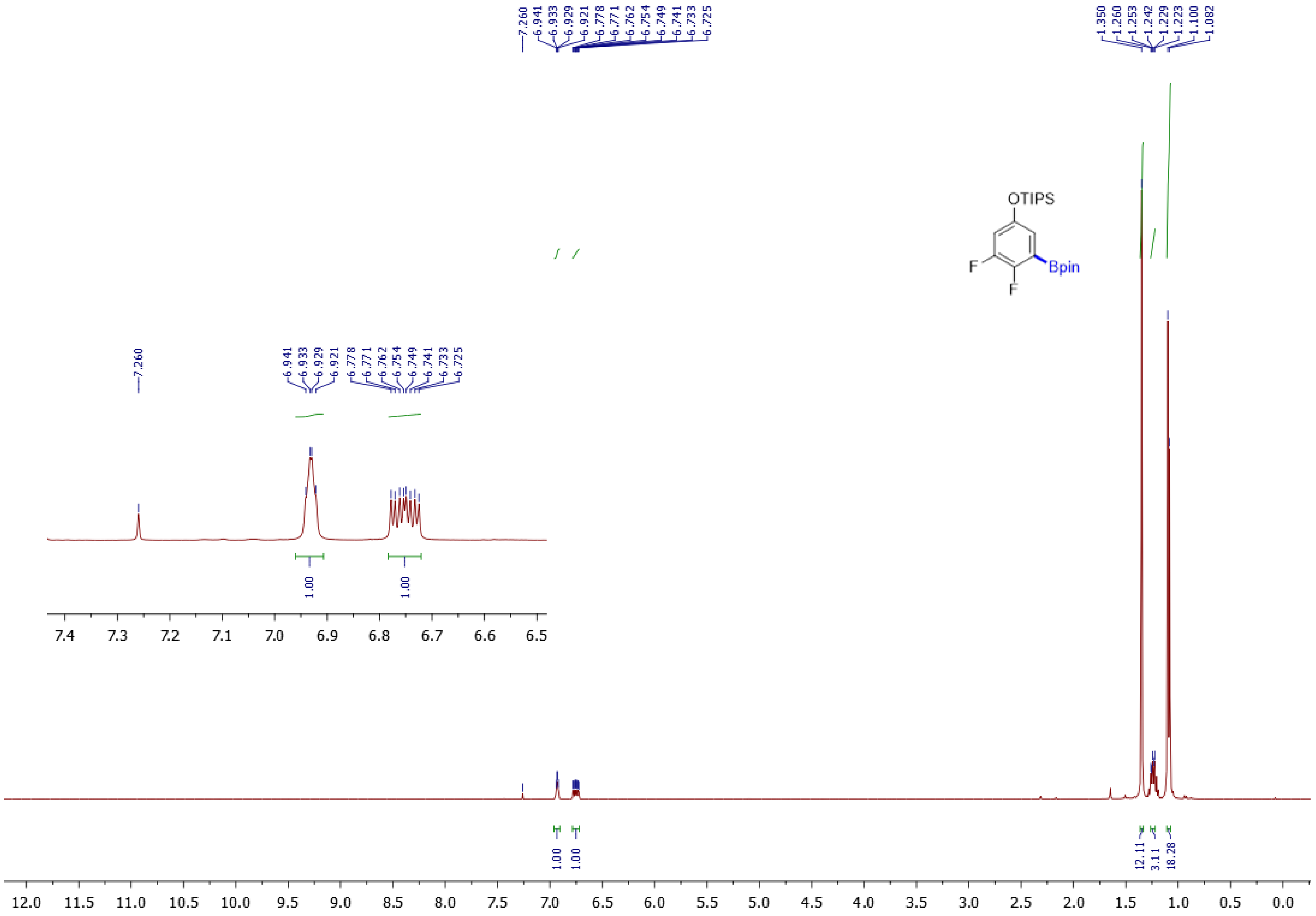

^{1}H-NMR spectra of **5ab** (25 °C, 400 MHz, $CDCl_3$)

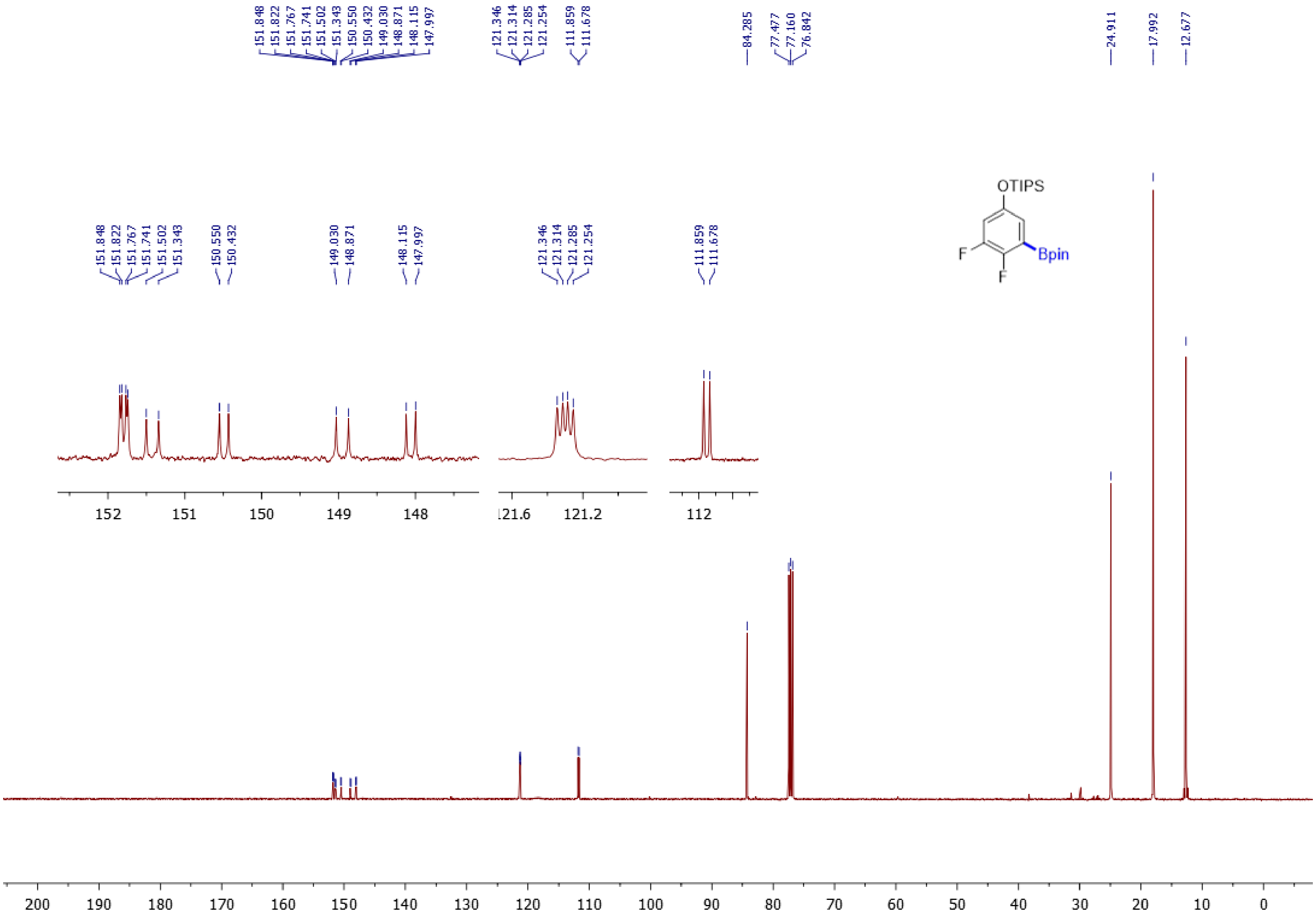

^{13}C-NMR spectra of **5ab** (25 ^{o}C, 100 MHz, $CDCl_3$)

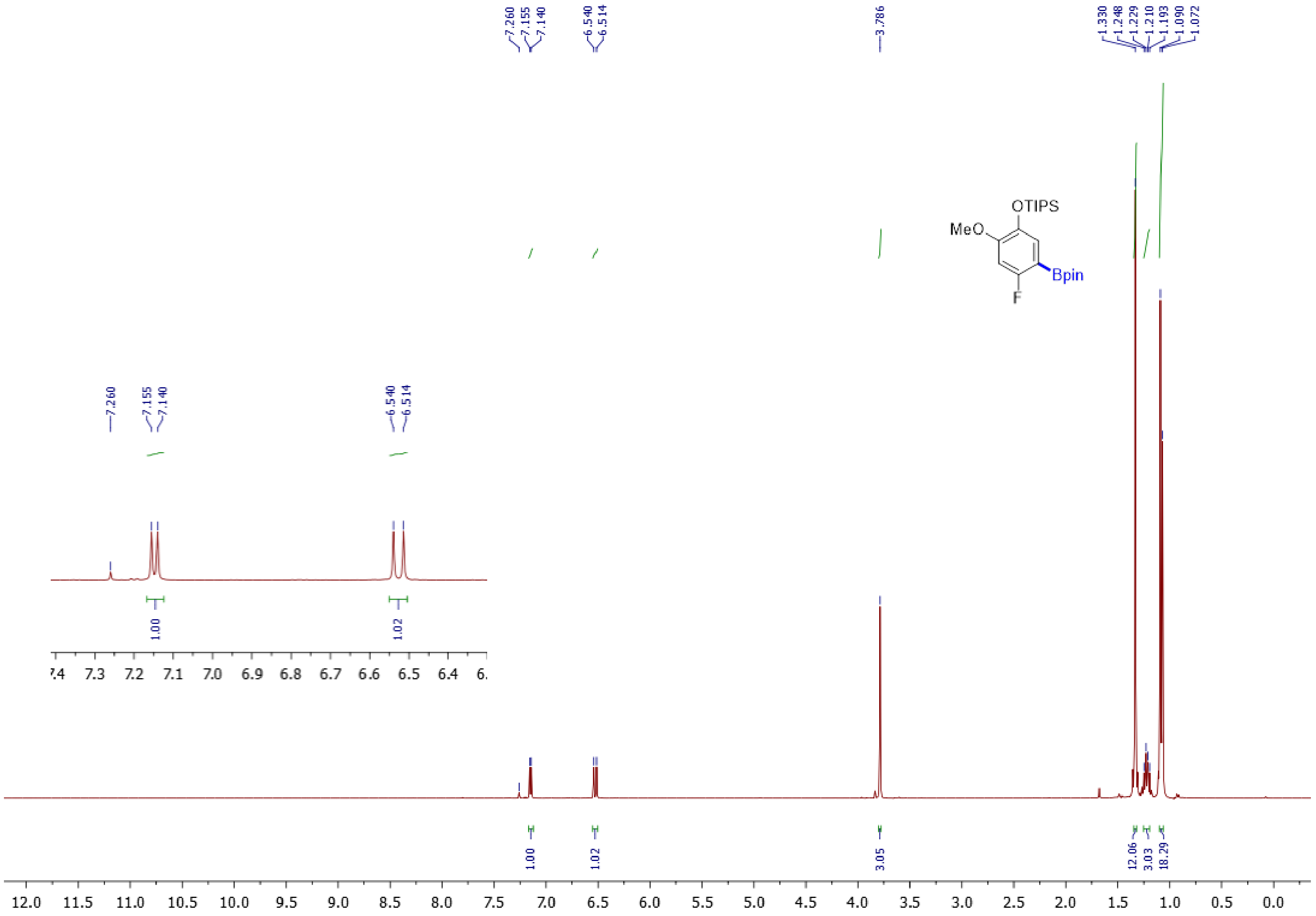

^{1}H-NMR spectra of **5ac** (25 ^{o}C, 400 MHz, $CDCl_3$)

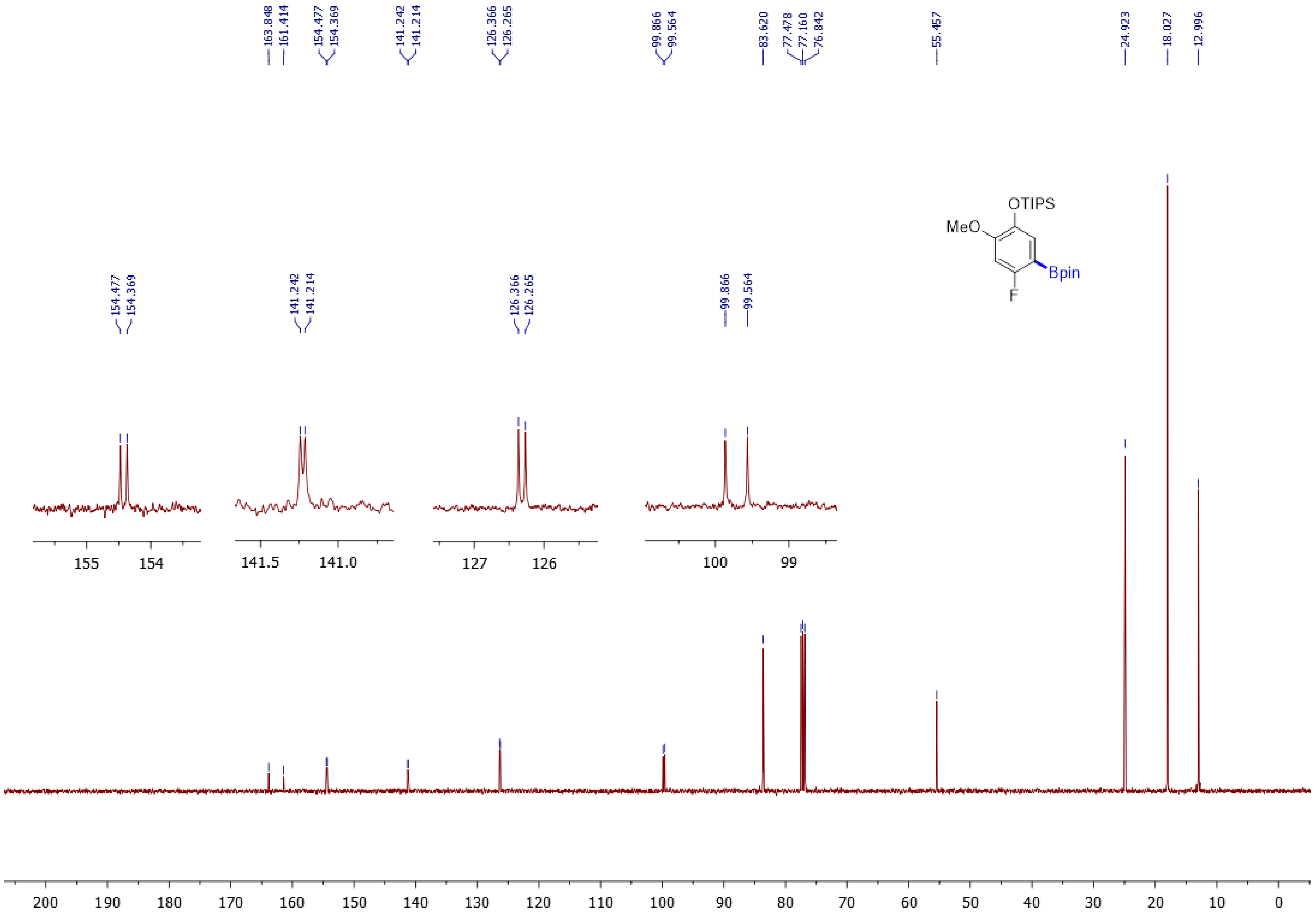

^{13}C-NMR spectra of **5ac** (25 ^{o}C, 100 MHz, $CDCl_3$)

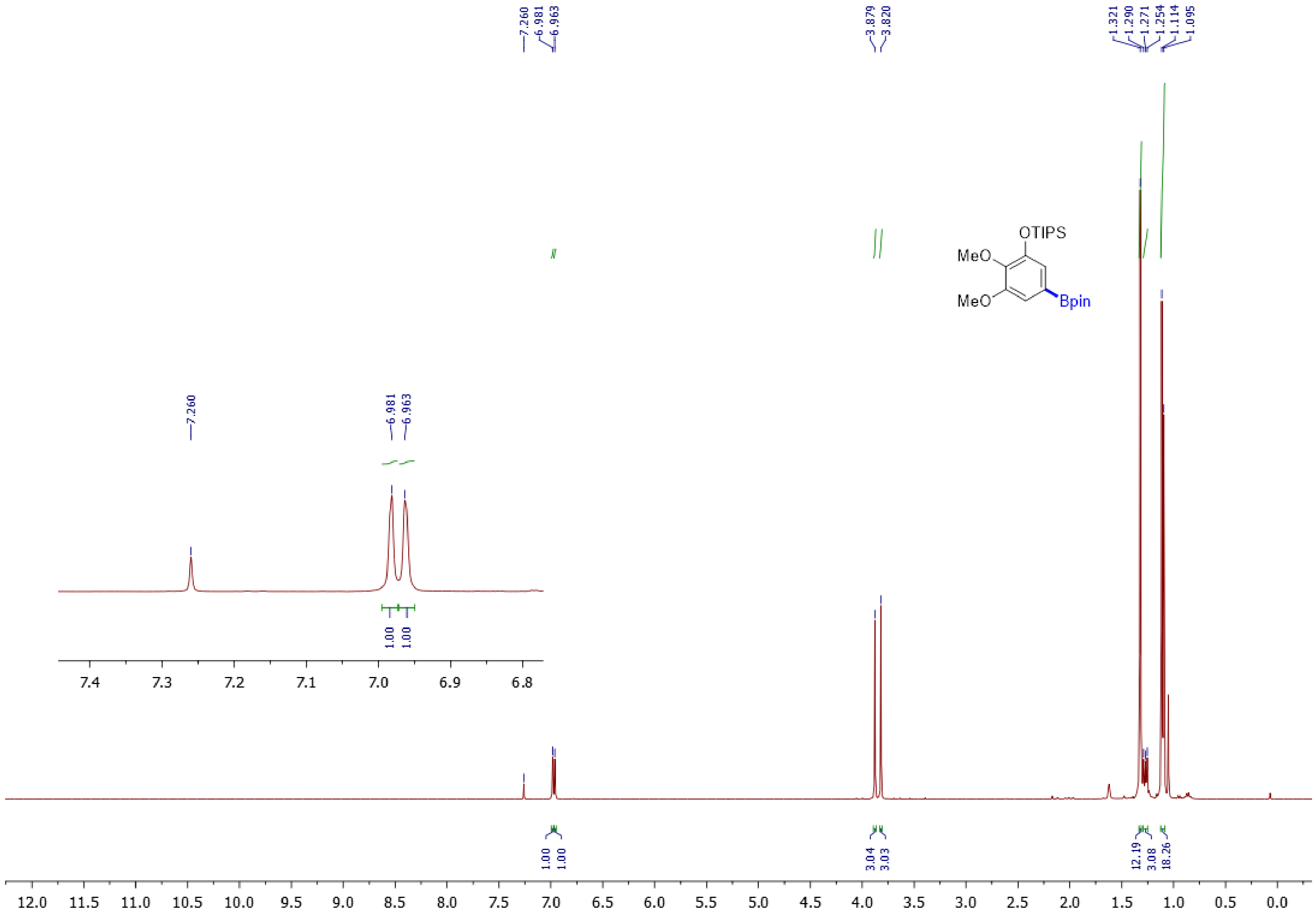

^{1}H-NMR spectra of **5ad** (25 ºC, 400 MHz, $CDCl_3$)

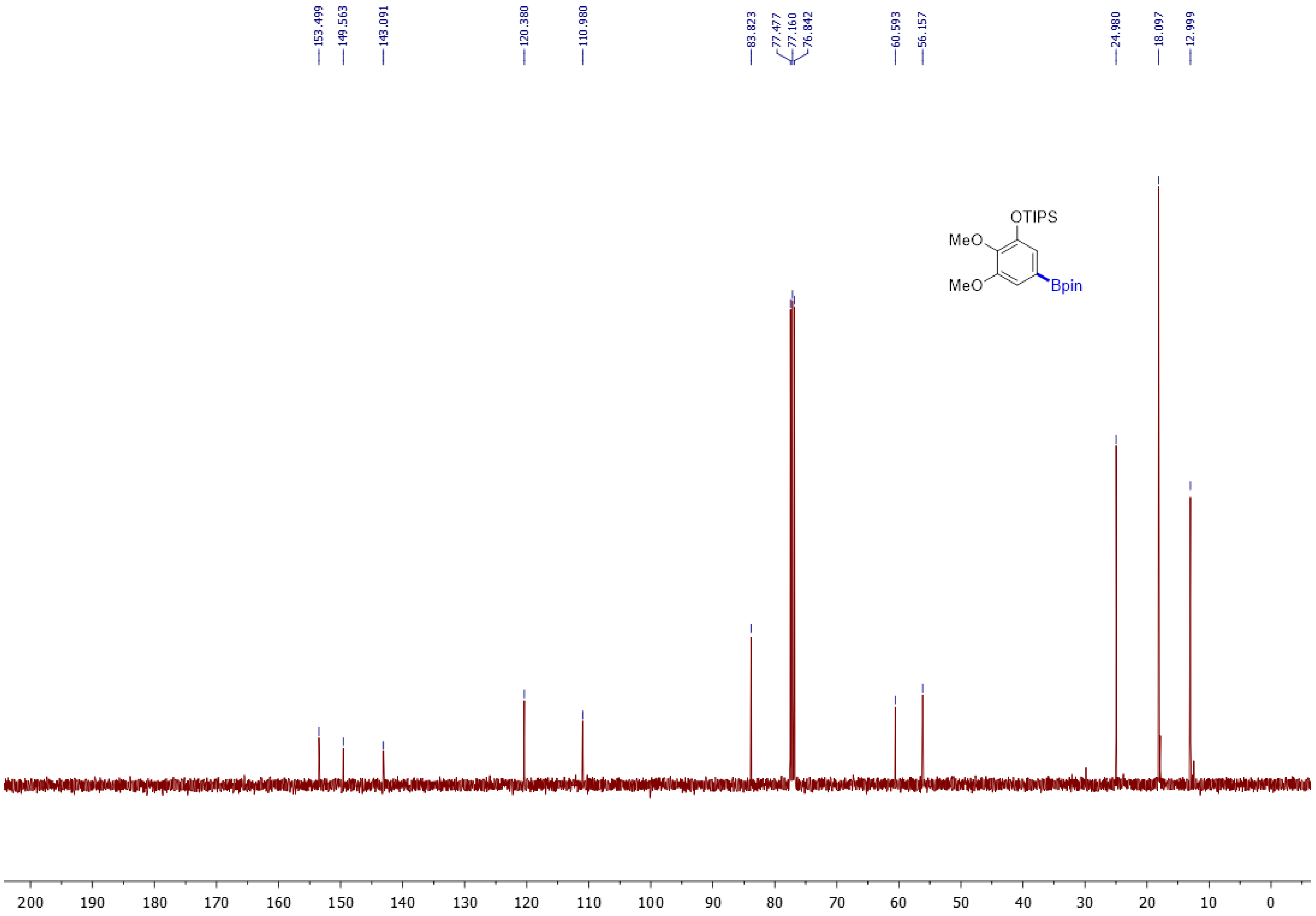

^{13}C-NMR spectra of **5ad** (25 °C, 100 MHz, $CDCl_3$)

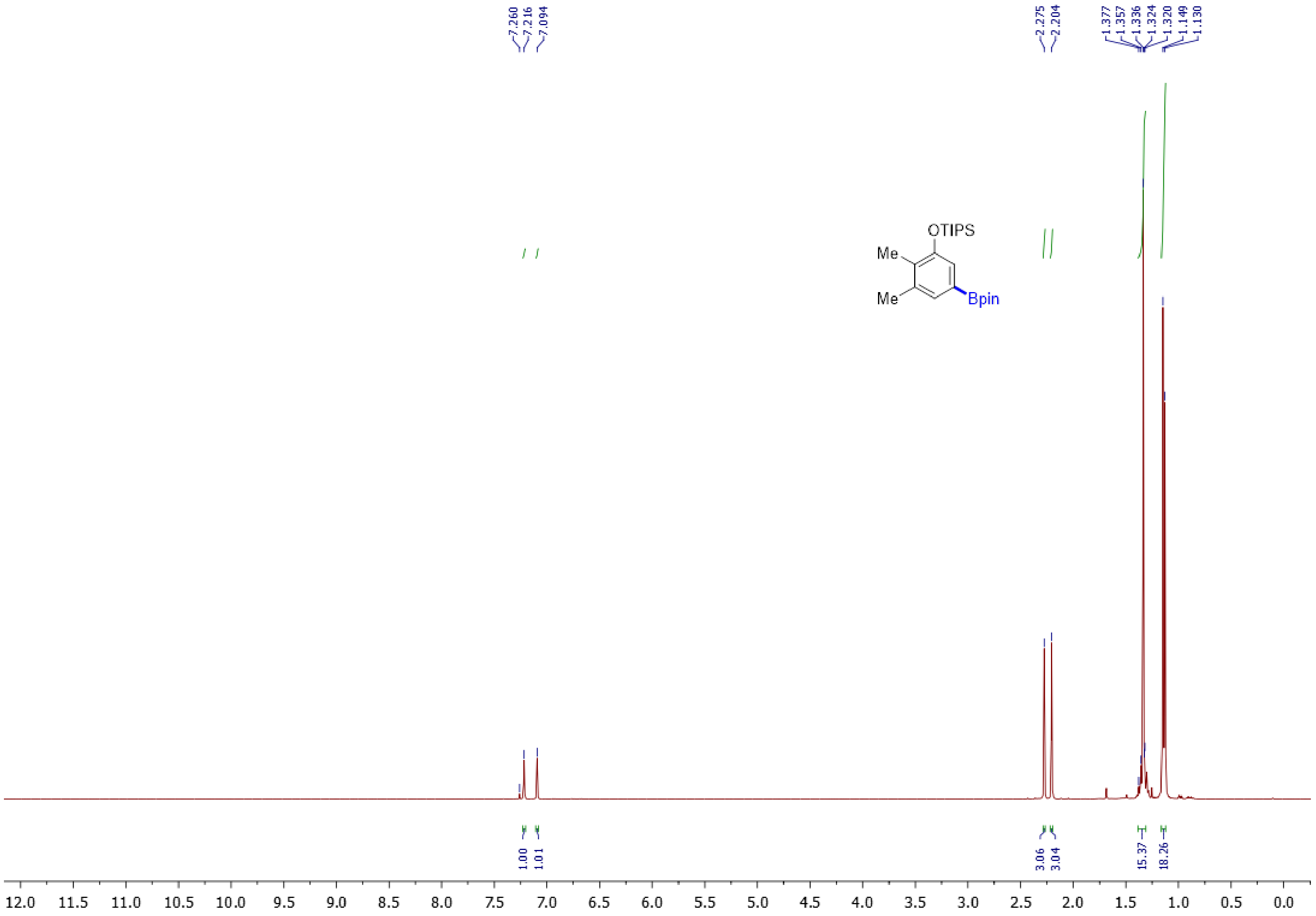

^{1}H-NMR spectra of **5ae** (25 °C, 400 MHz, $CDCl_3$)

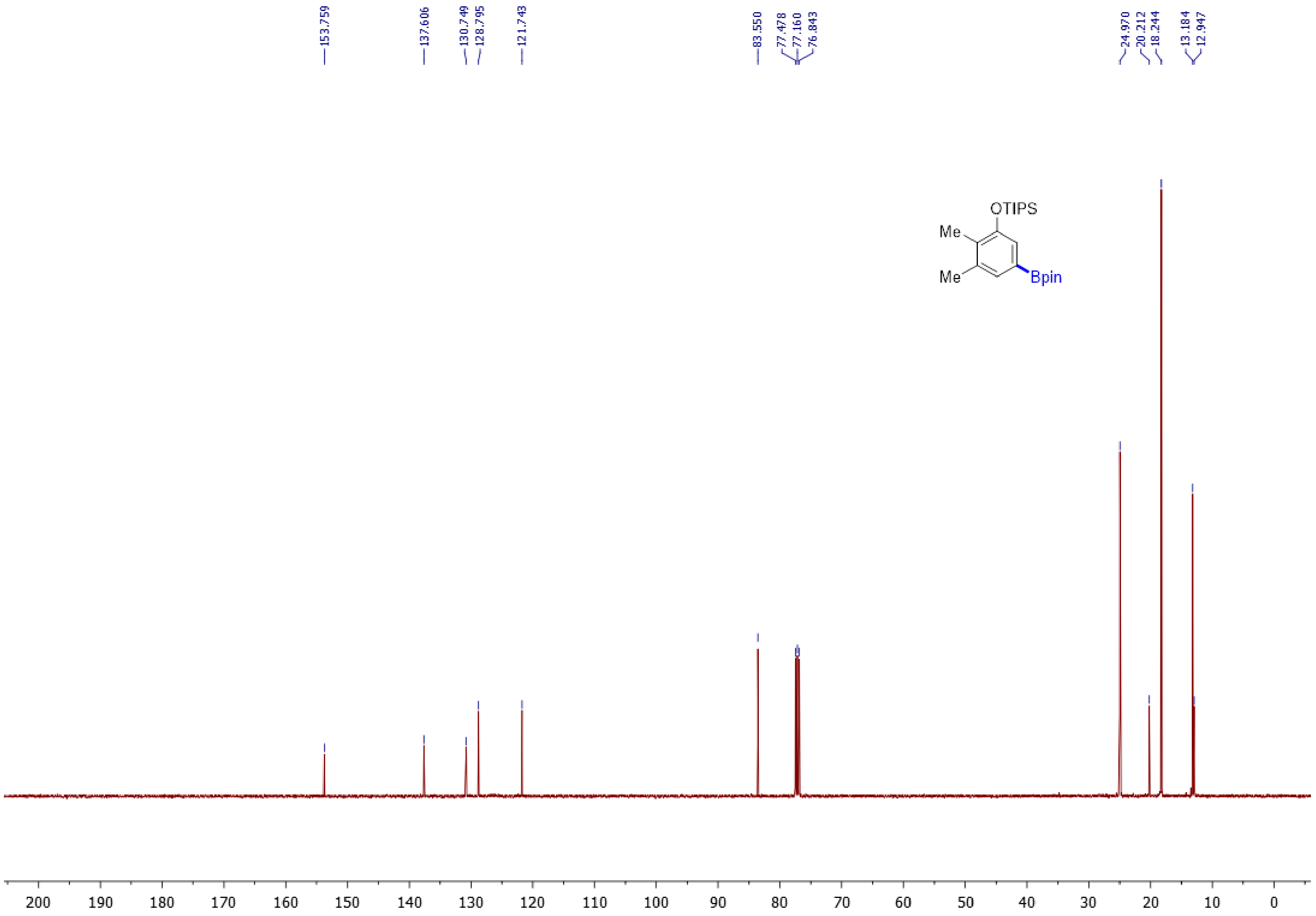

^{13}C-NMR spectra of **5ae** (25 ^{o}C, 100 MHz, $CDCl_3$)

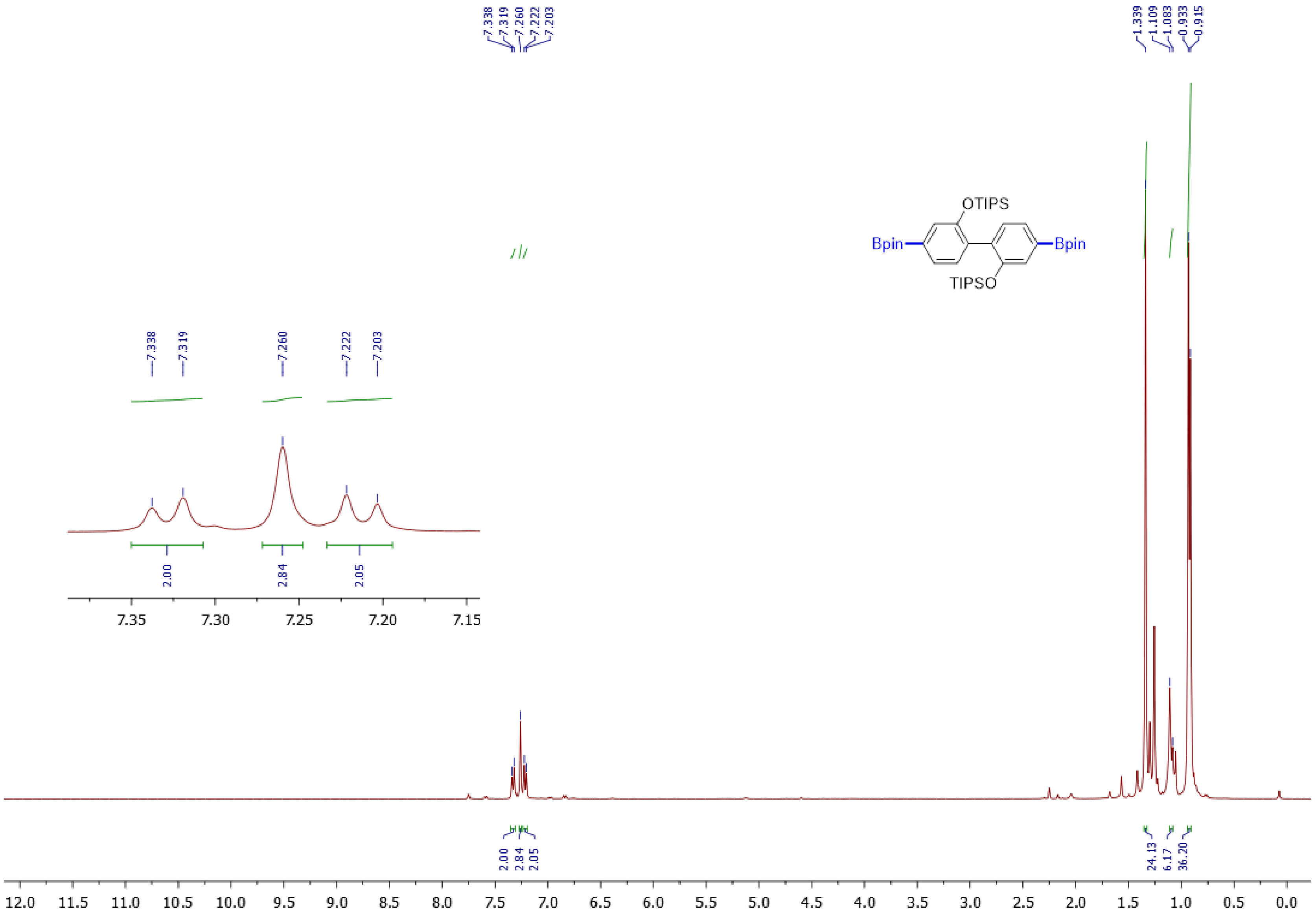

^{1}H-NMR spectra of **5ag** (25 ^{o}C, 400 MHz, $CDCl_3$)

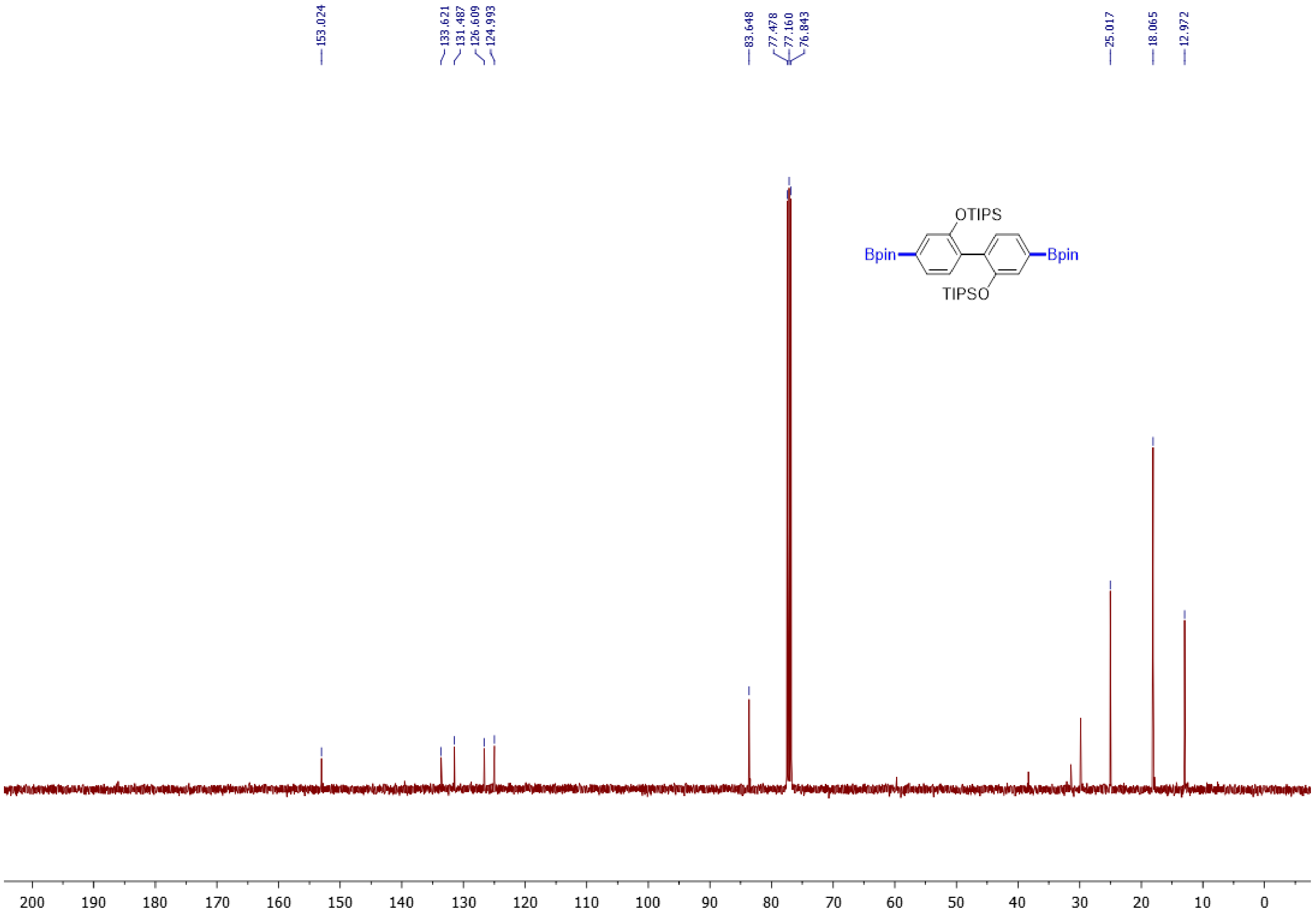

^{13}C-NMR spectra of **5ag** (25 ^{o}C, 100 MHz, $CDCl_3$)

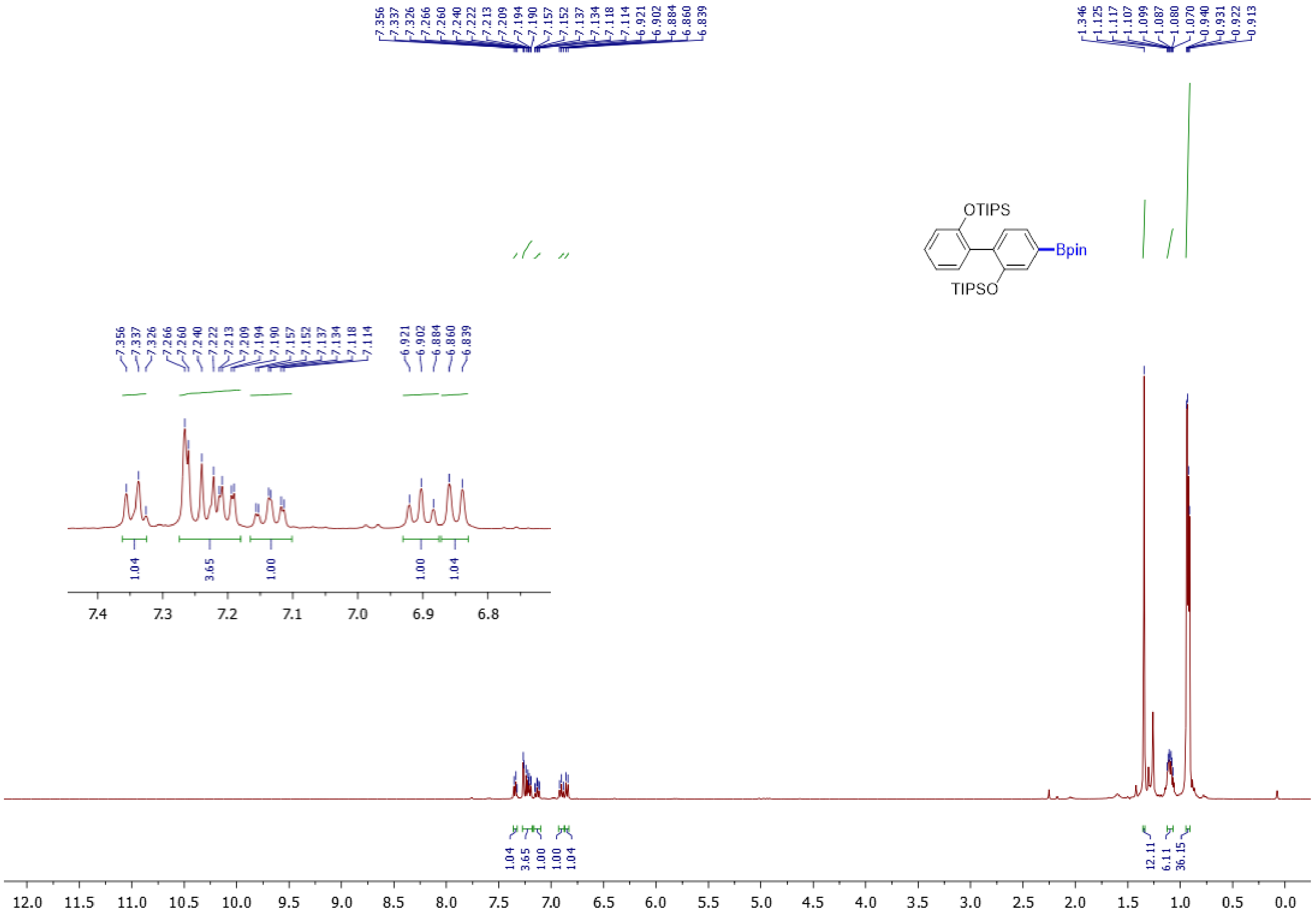

^{1}H-NMR spectra of **5ah** (25 ^oC, 400 MHz, $CDCl_3$)

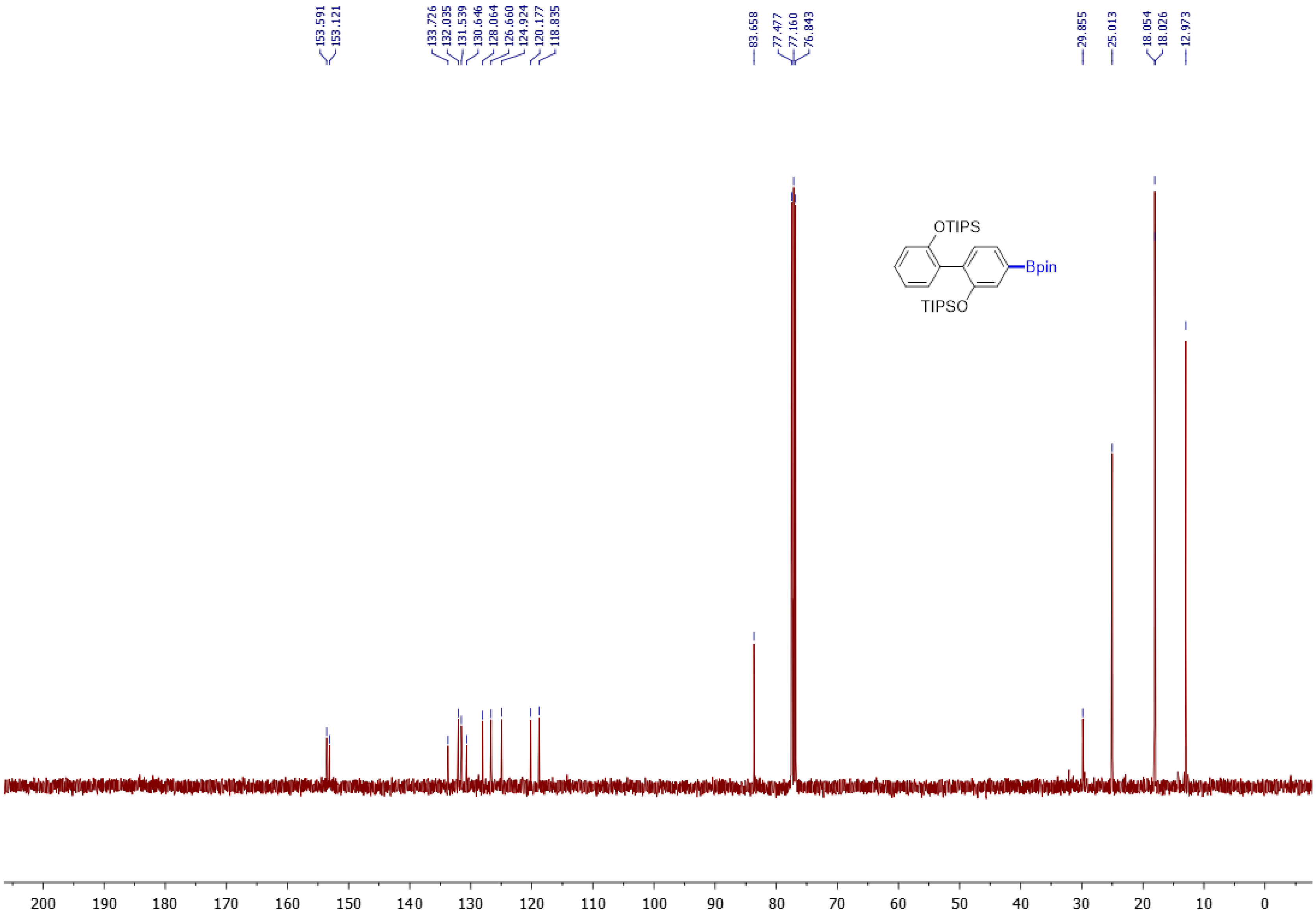

^{13}C-NMR spectra of **5ah** (25 °C, 100 MHz, $CDCl_3$)

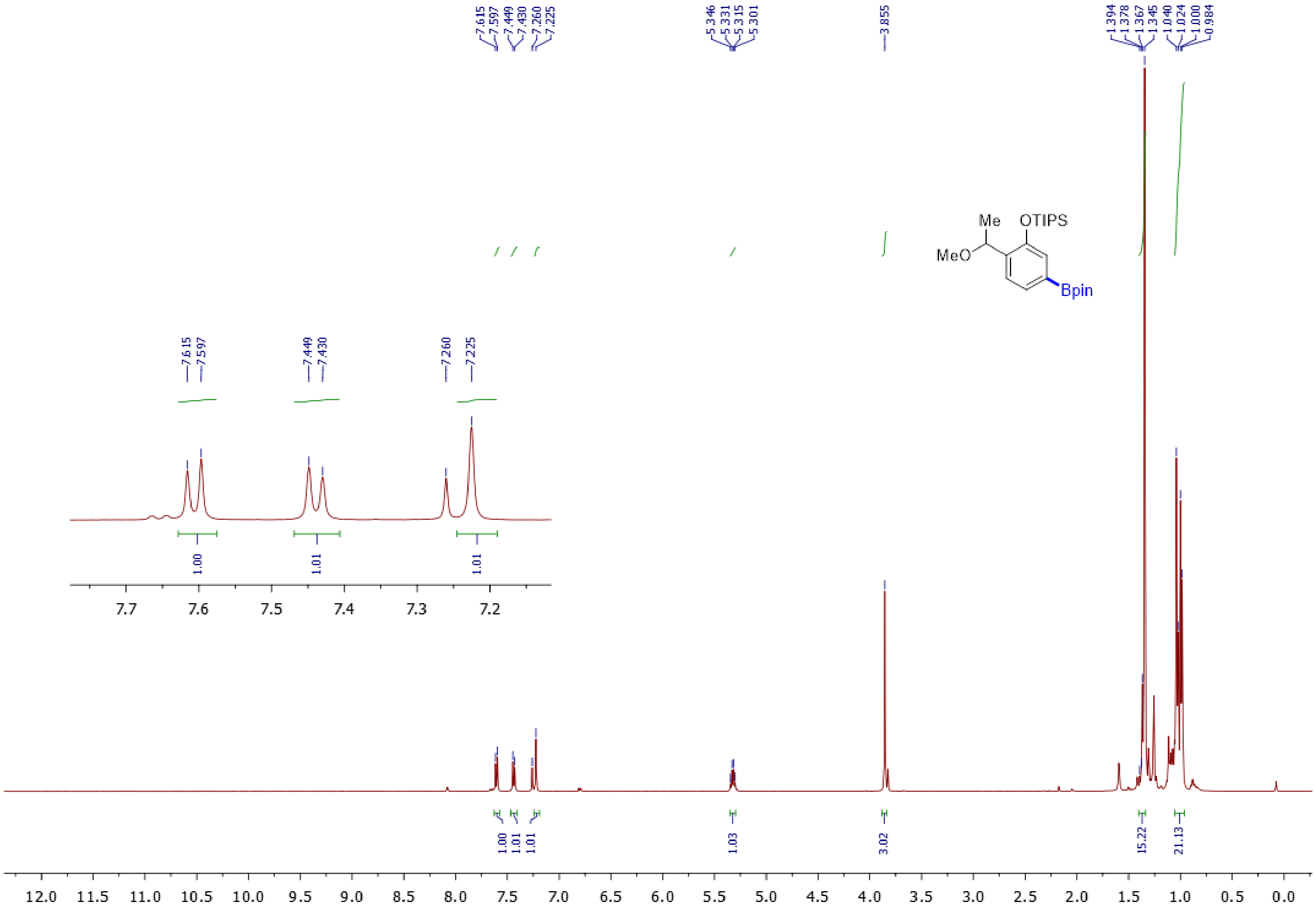

^{1}H-NMR spectra of **5ai** (25 °C, 400 MHz, $CDCl_3$)

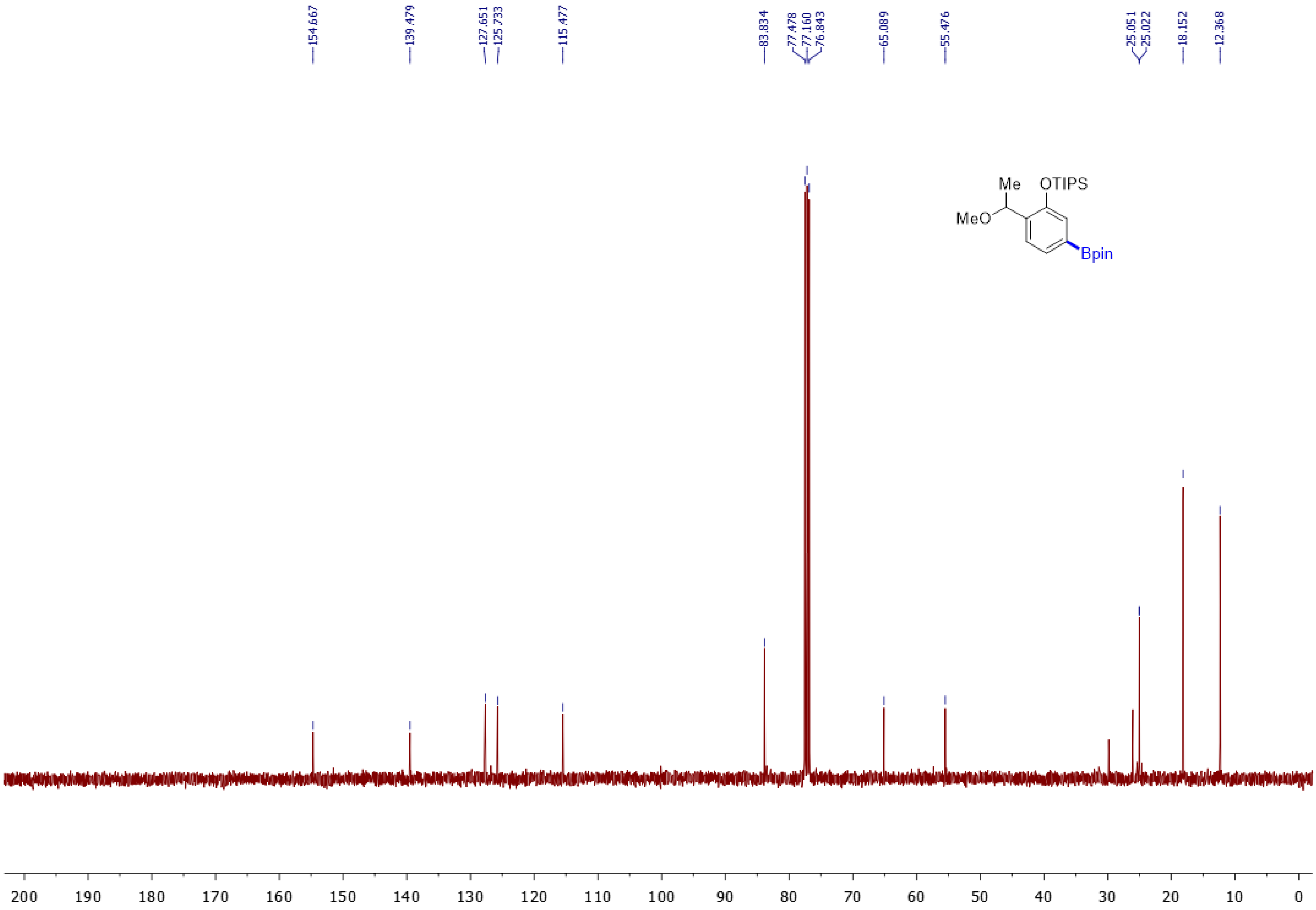

^{13}C-NMR spectra of **5ai** (25 ^{o}C, 100 MHz, $CDCl_3$)

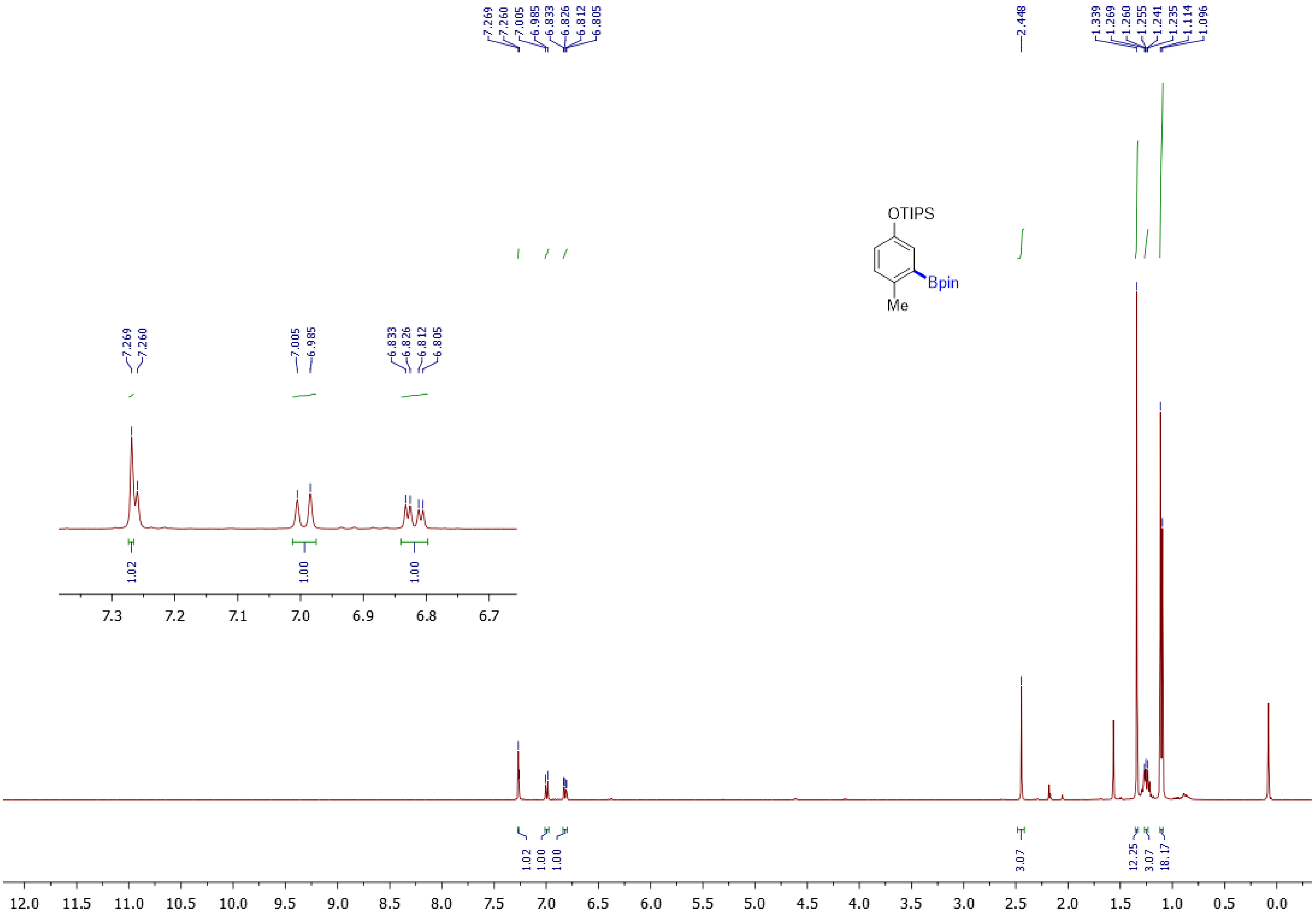

^{1}H-NMR spectra of **7a** (25 ^{o}C, 400 MHz, $CDCl_3$)

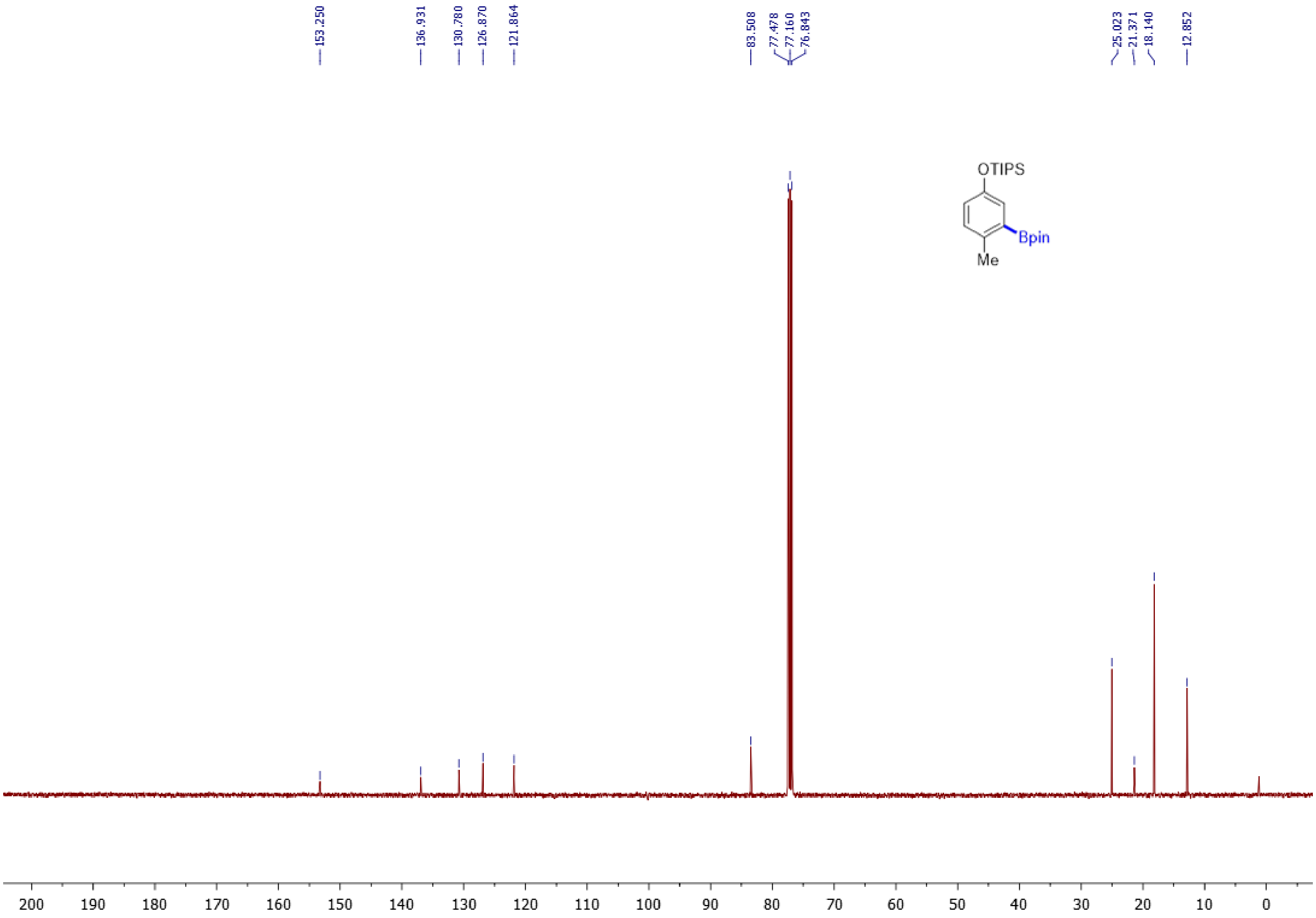

^{13}C-NMR spectra of **7a** (25 °C, 100 MHz, $CDCl_3$)

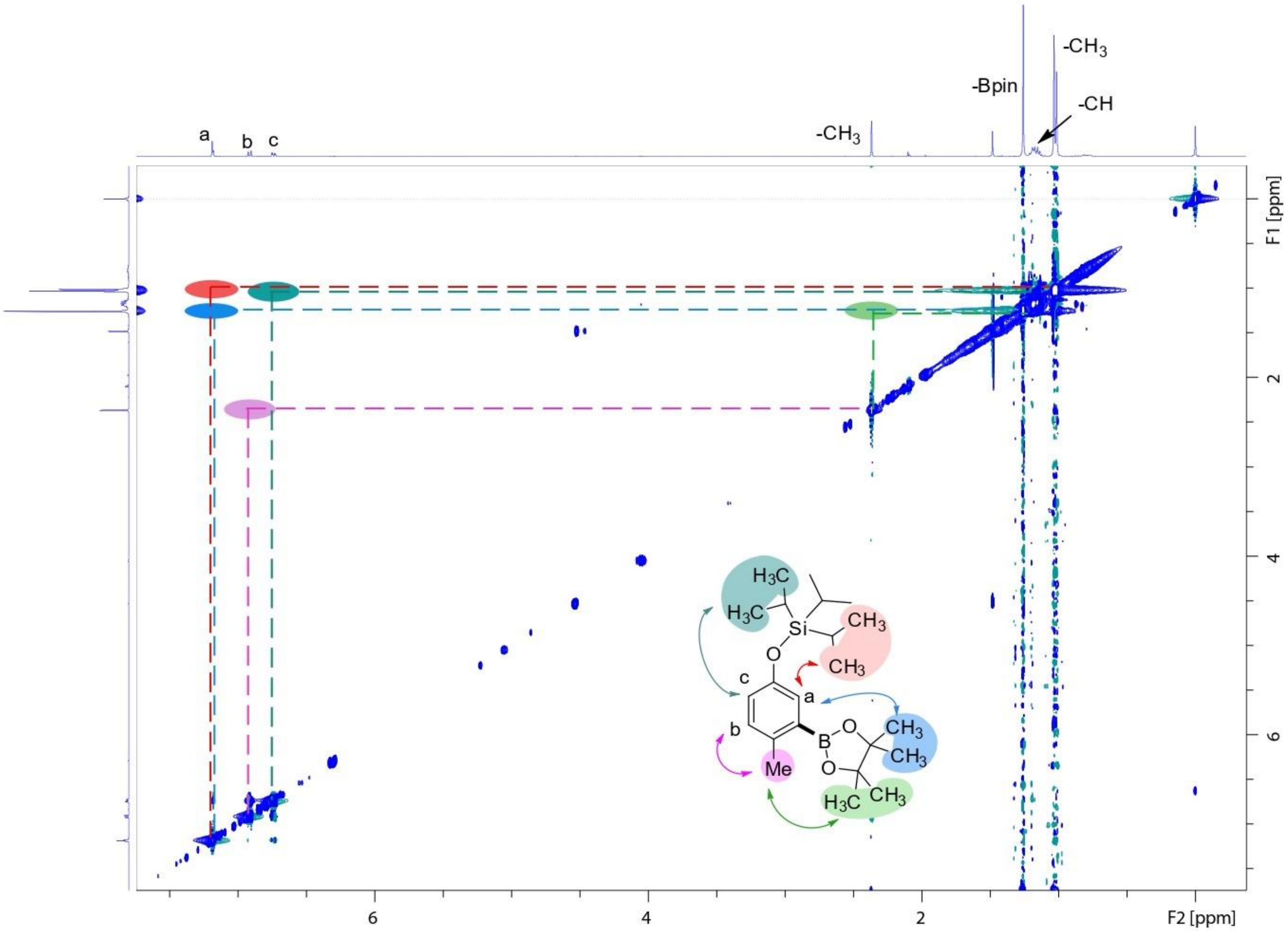

NOESY-NMR spectra of **7a** (25 ^{o}C, 100 MHz, $CDCl_3$)

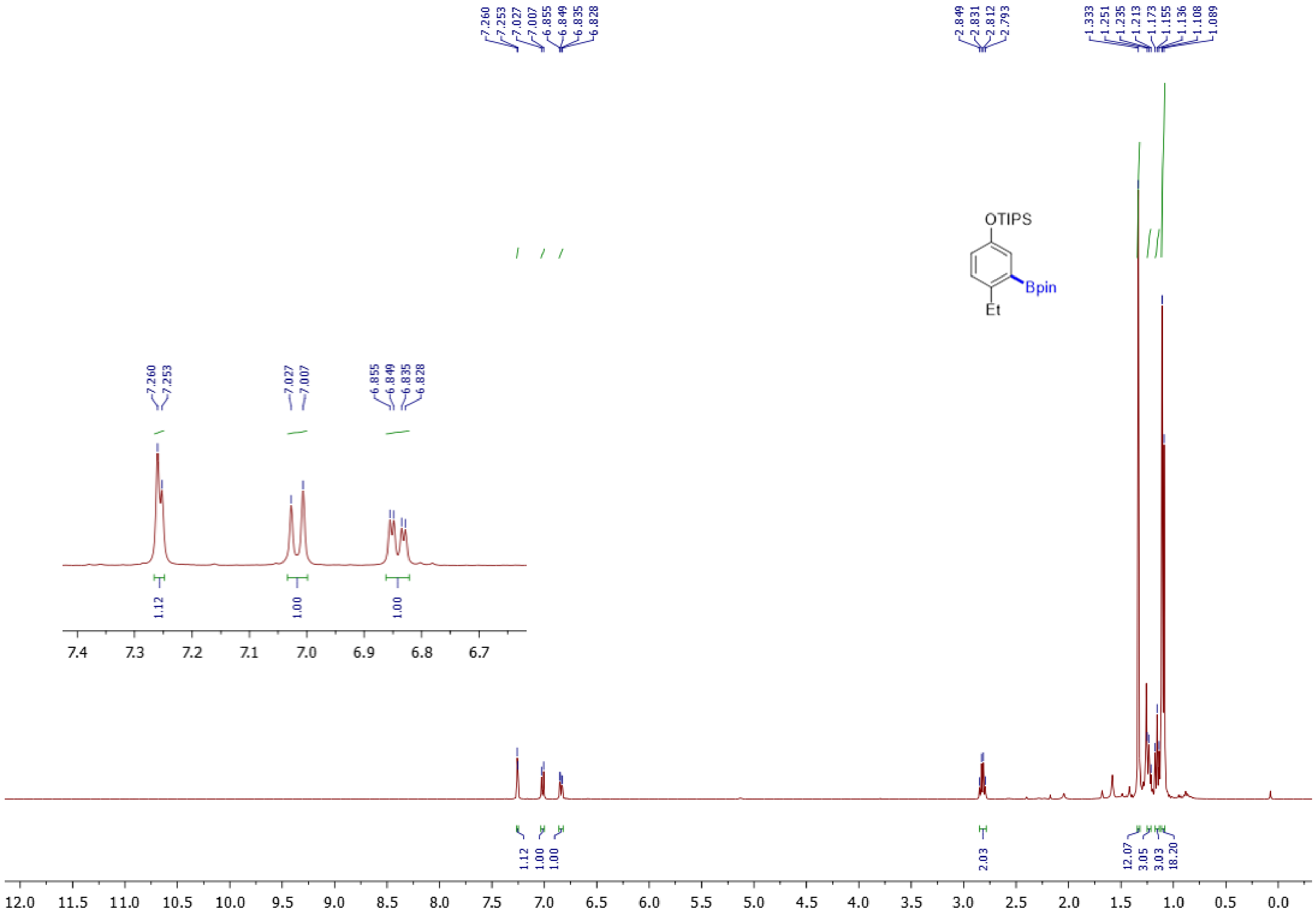

^{1}H-NMR spectra of **7b** (25 ºC, 400 MHz, $CDCl_3$)

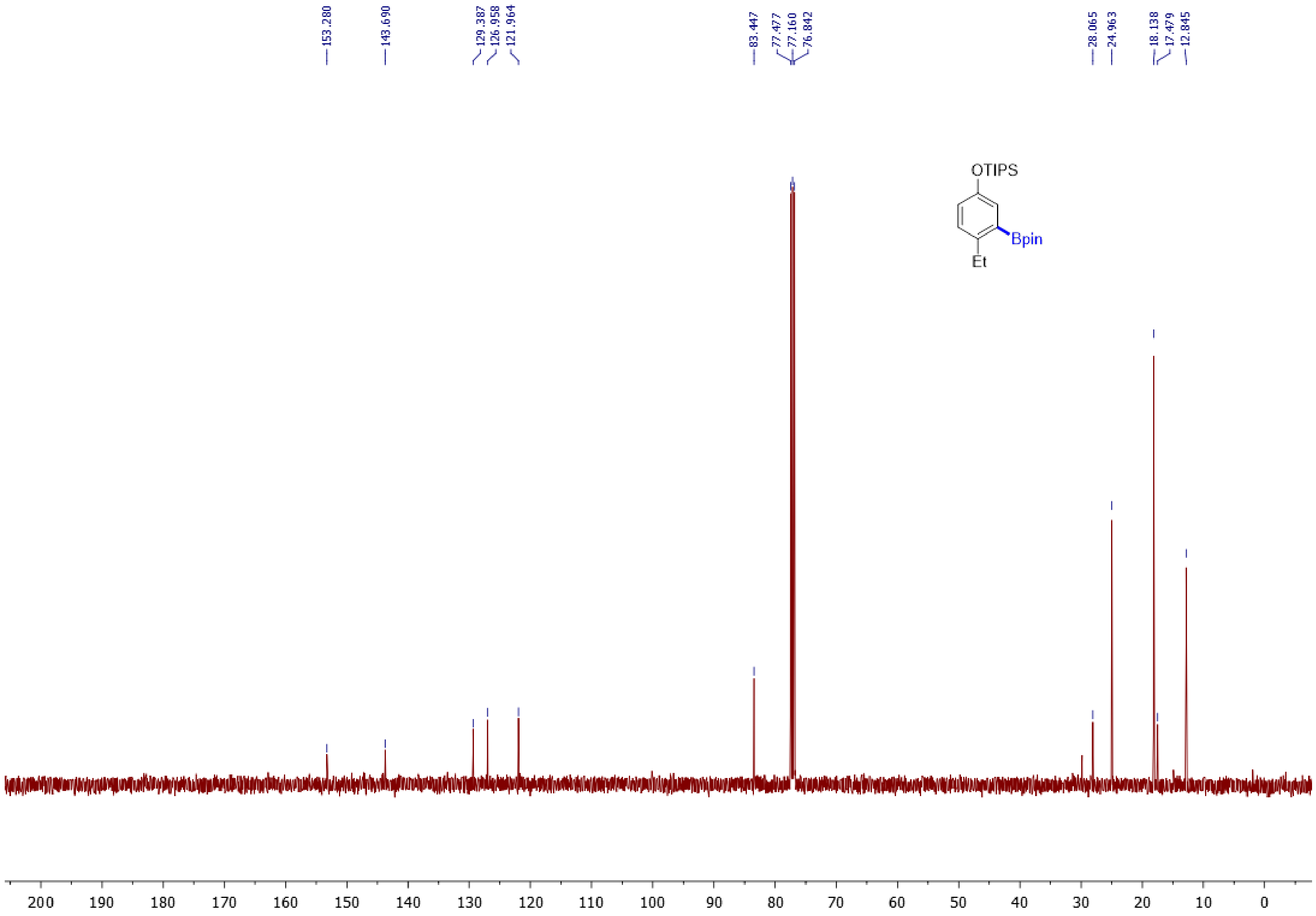

^{13}C-NMR spectra of **7b** (25 ºC, 100 MHz, $CDCl_3$)

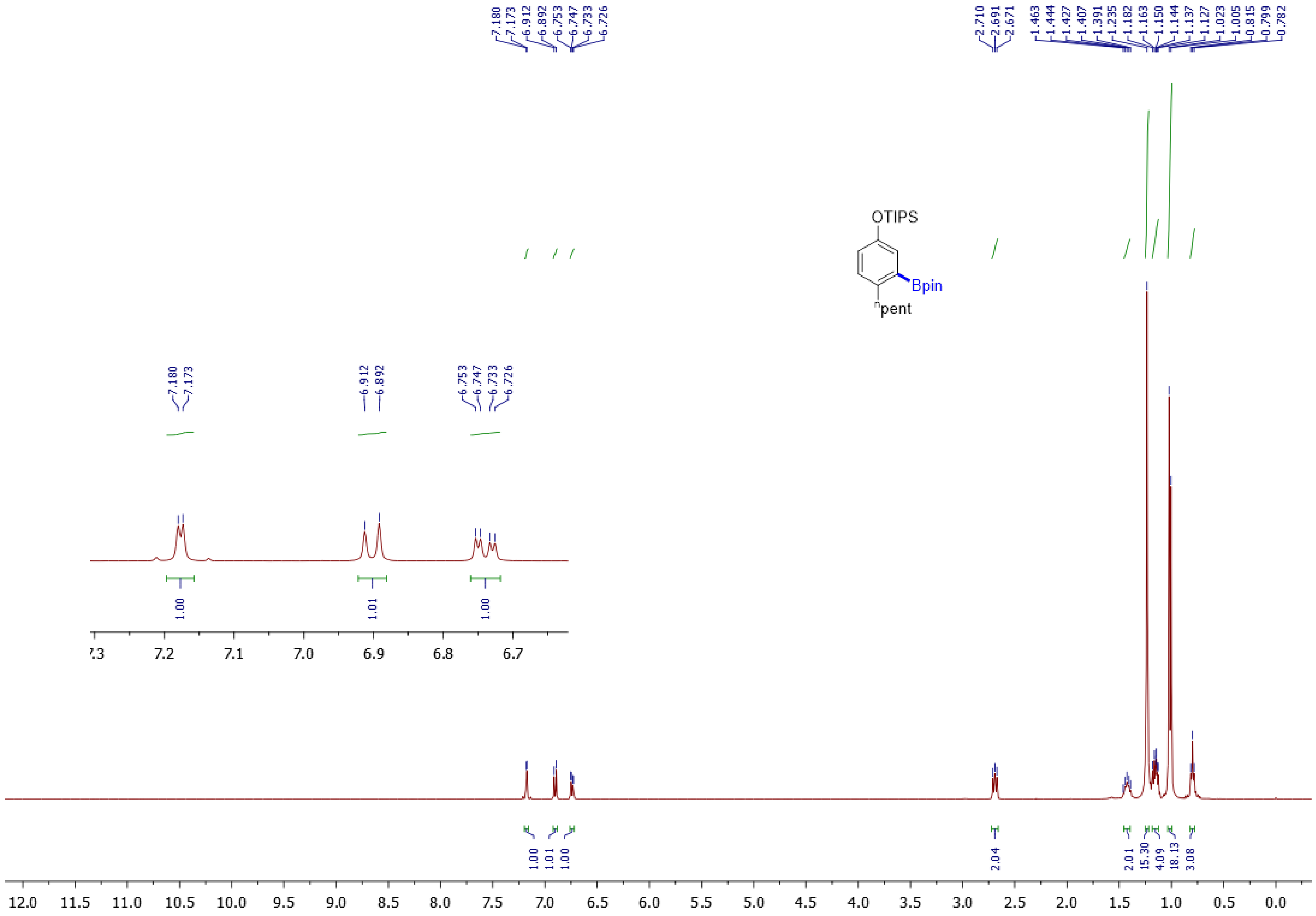

^{1}H-NMR spectra of **7c** (25 ^{o}C, 400 MHz, $CDCl_3$)

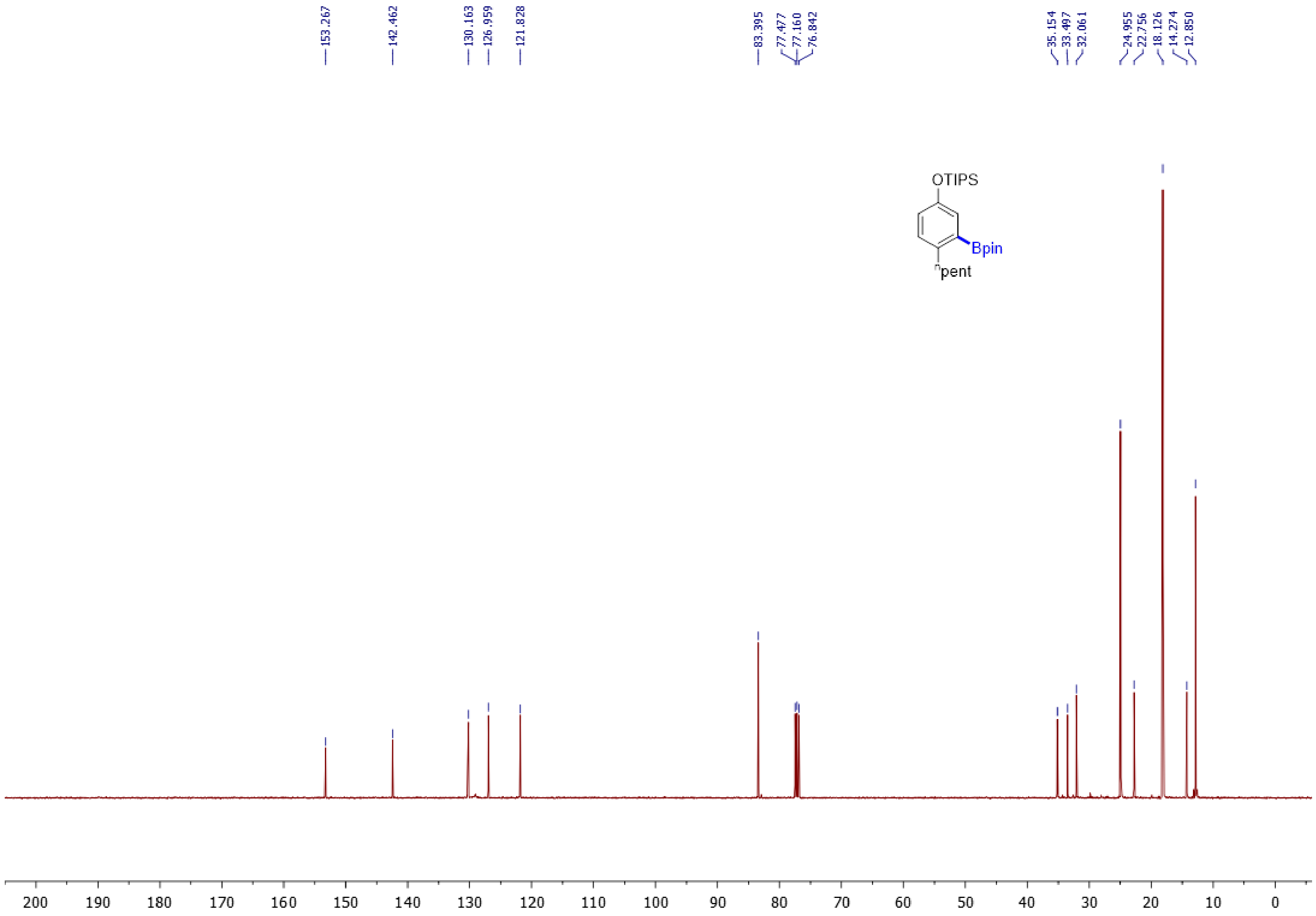

^{13}C-NMR spectra of **7c** (25 ºC, 100 MHz, $CDCl_3$)

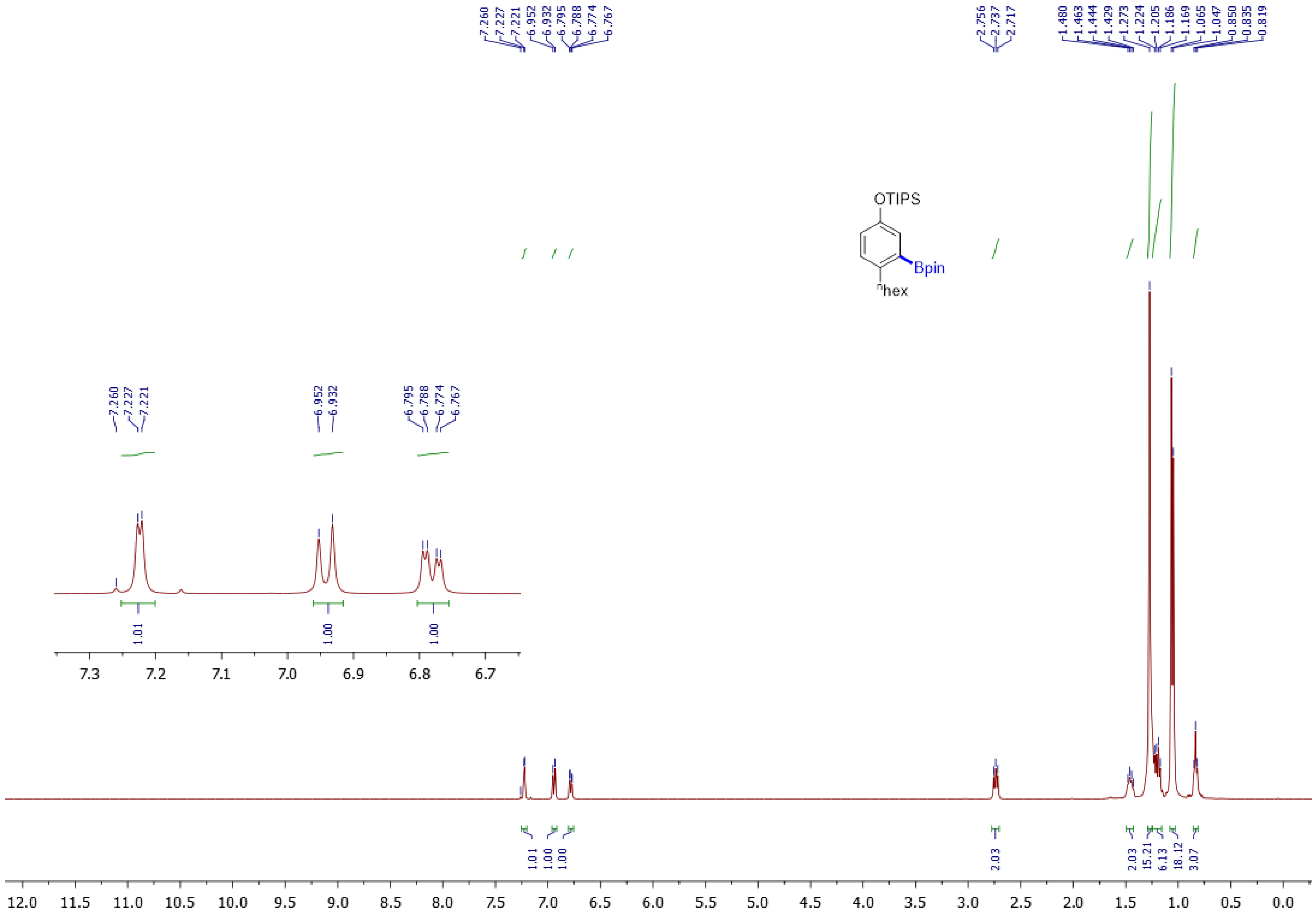

^{1}H-NMR spectra of **7d** (25 ^{o}C, 400 MHz, $CDCl_3$)

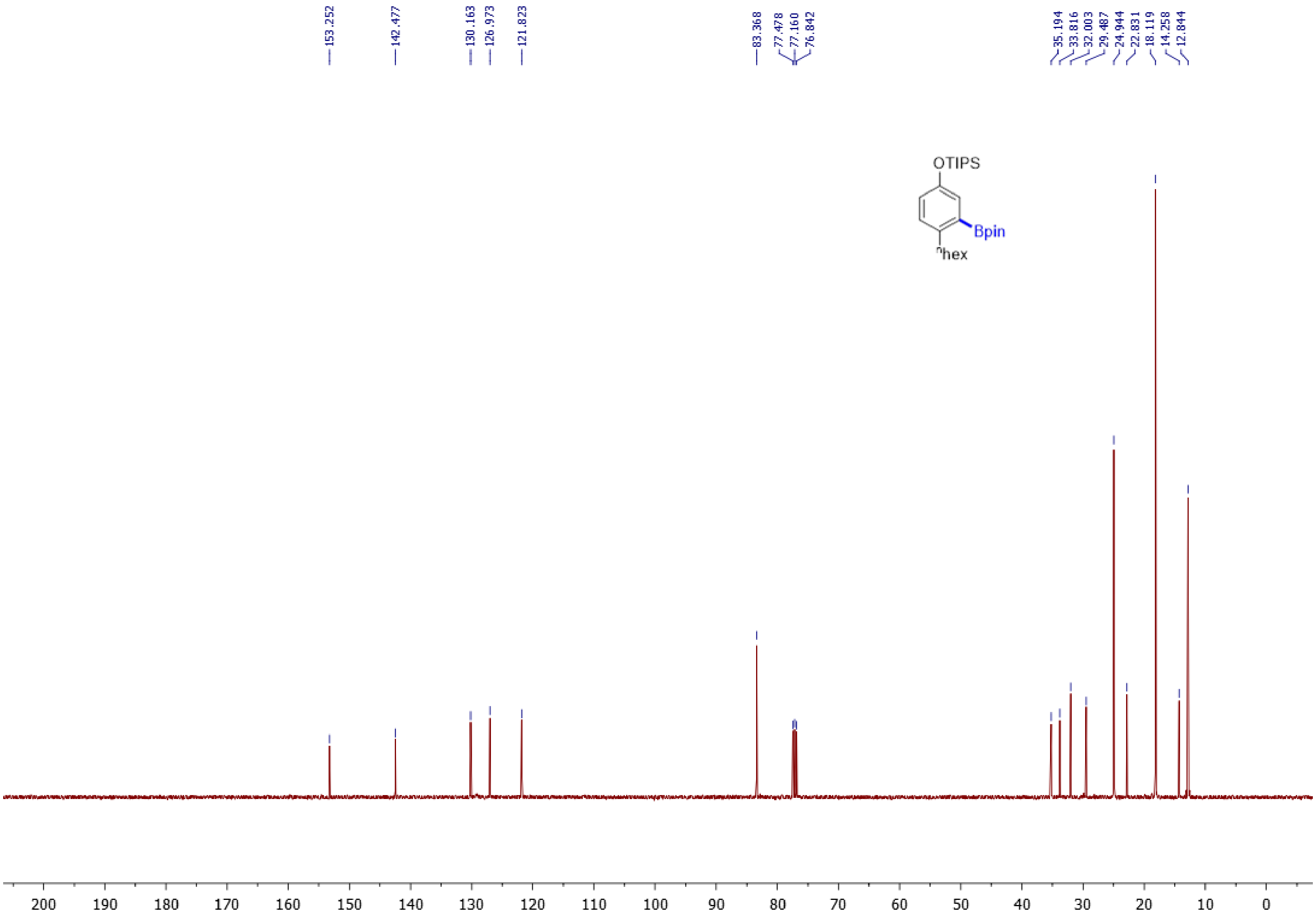

^{13}C-NMR spectra of **7d** (25 °C, 100 MHz, $CDCl_3$)

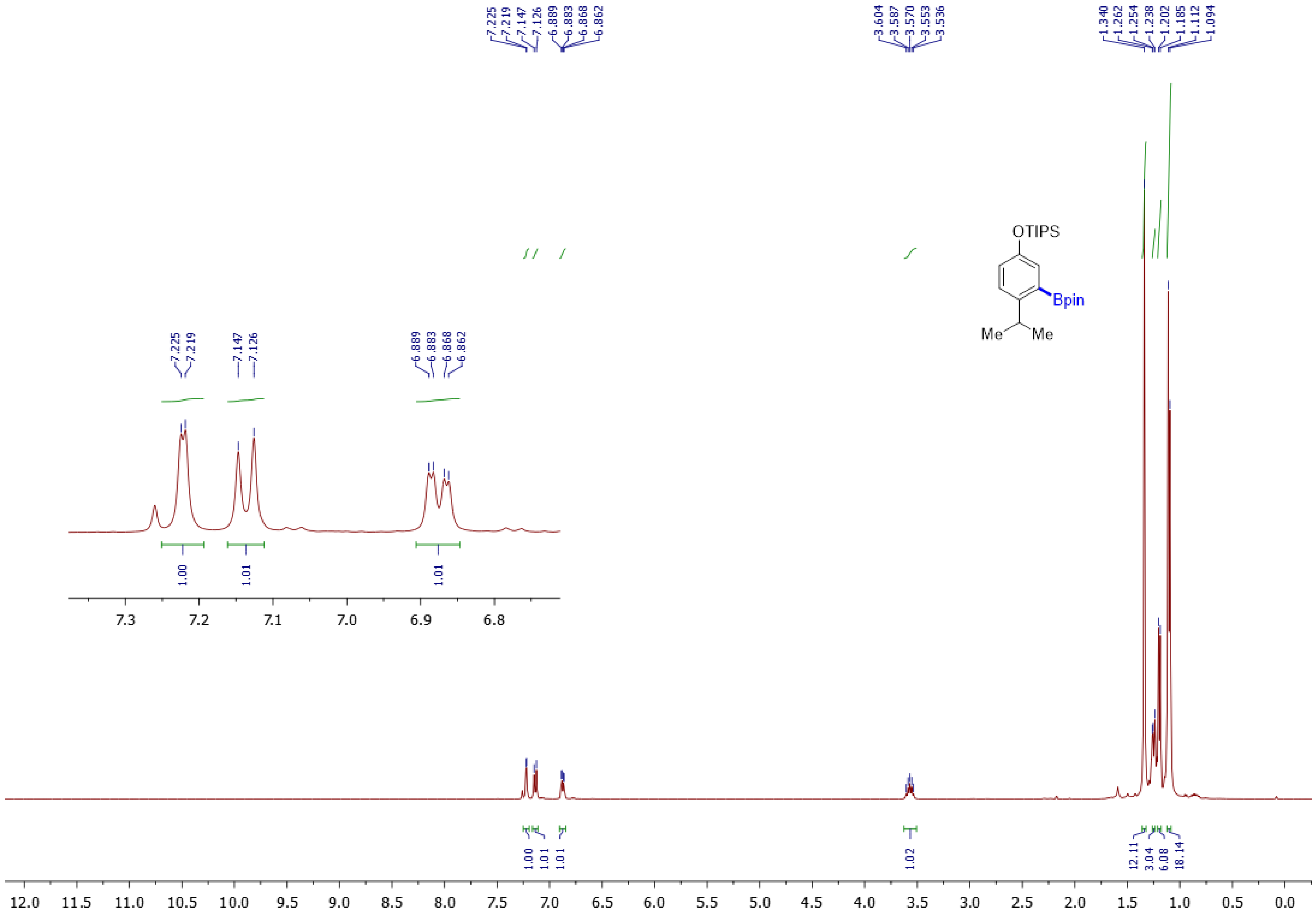

^{1}H-NMR spectra of **7e** (25 ^{o}C, 400 MHz, $CDCl_3$)

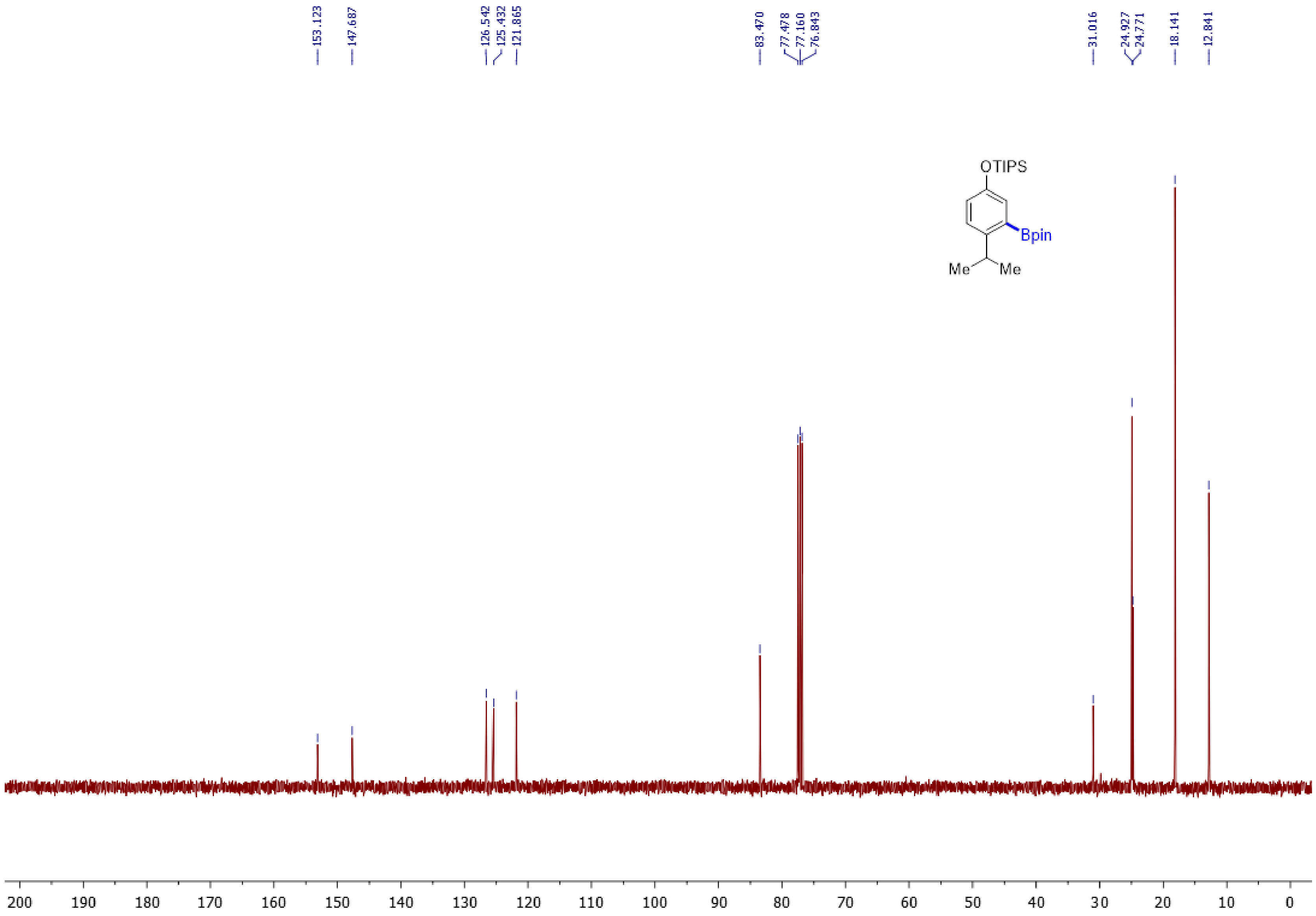

^{13}C-NMR spectra of **7e** (25 ºC, 100 MHz, $CDCl_3$)

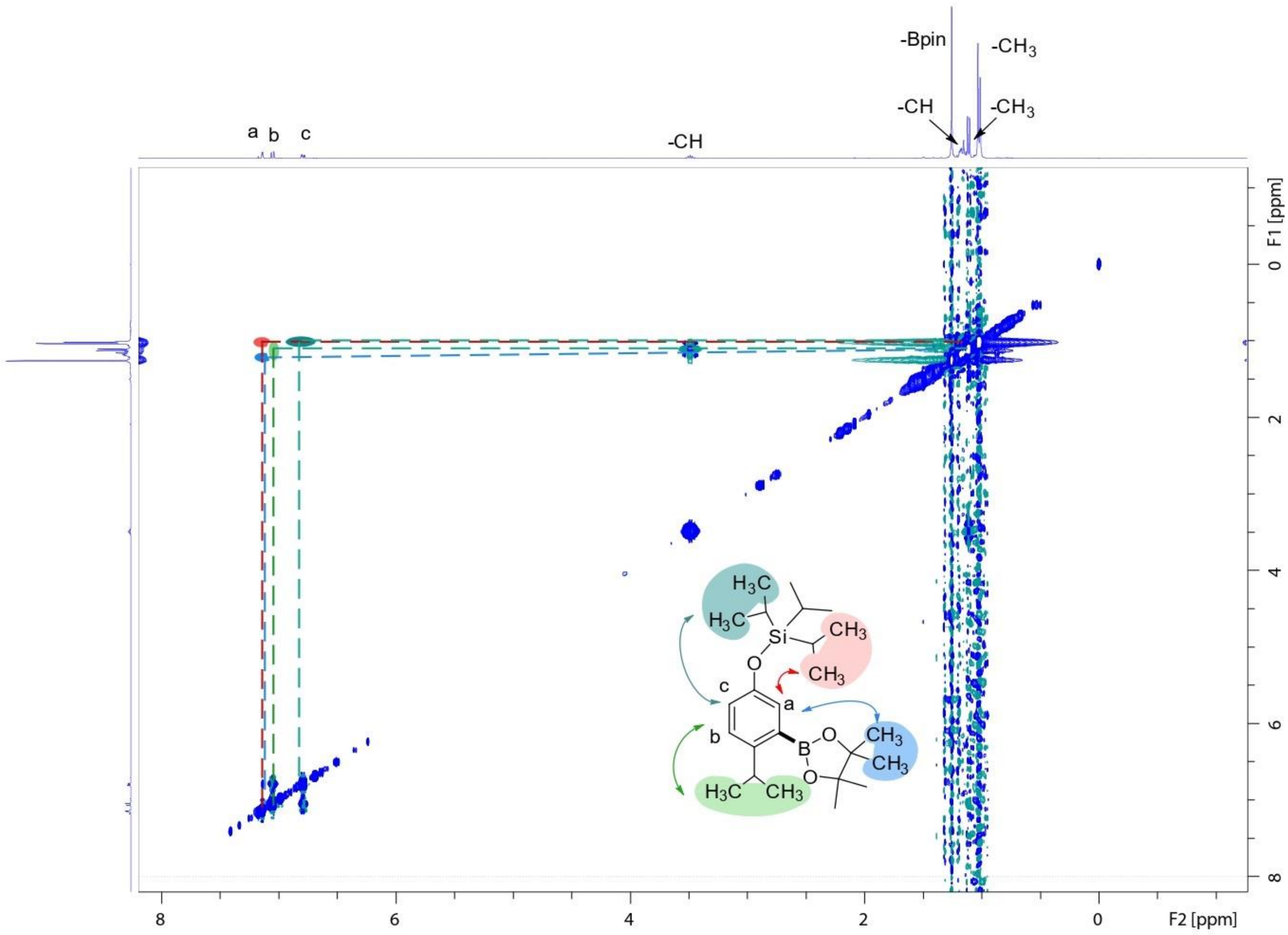

NOESY-NMR spectra of **7e** (25 ^{o}C, 100 MHz, $CDCl_3$)

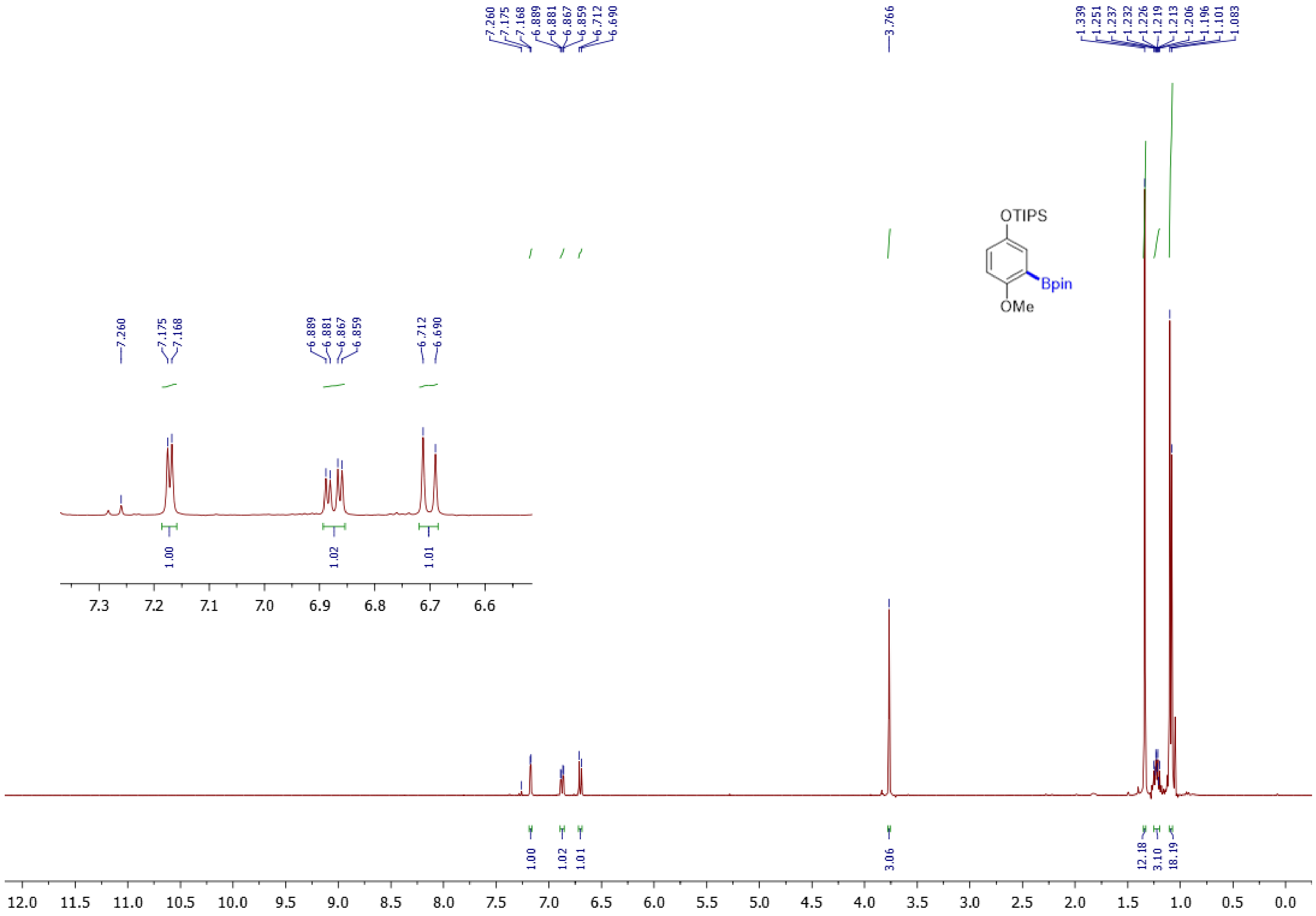

^{1}H-NMR spectra of **7f** (25 ^{o}C, 400 MHz, $CDCl_3$)

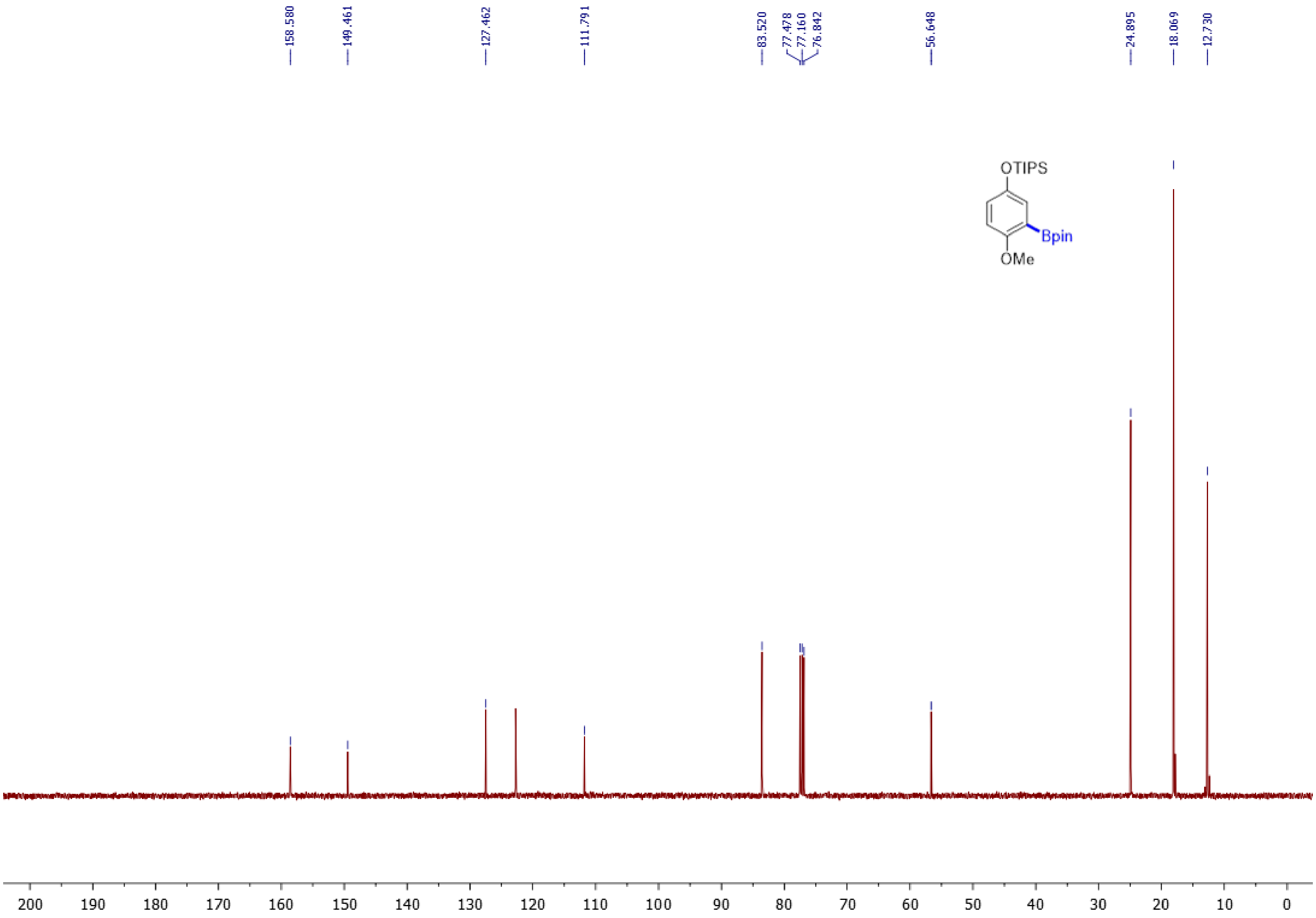

^{13}C-NMR spectra of **7f** (25 °C, 100 MHz, $CDCl_3$)

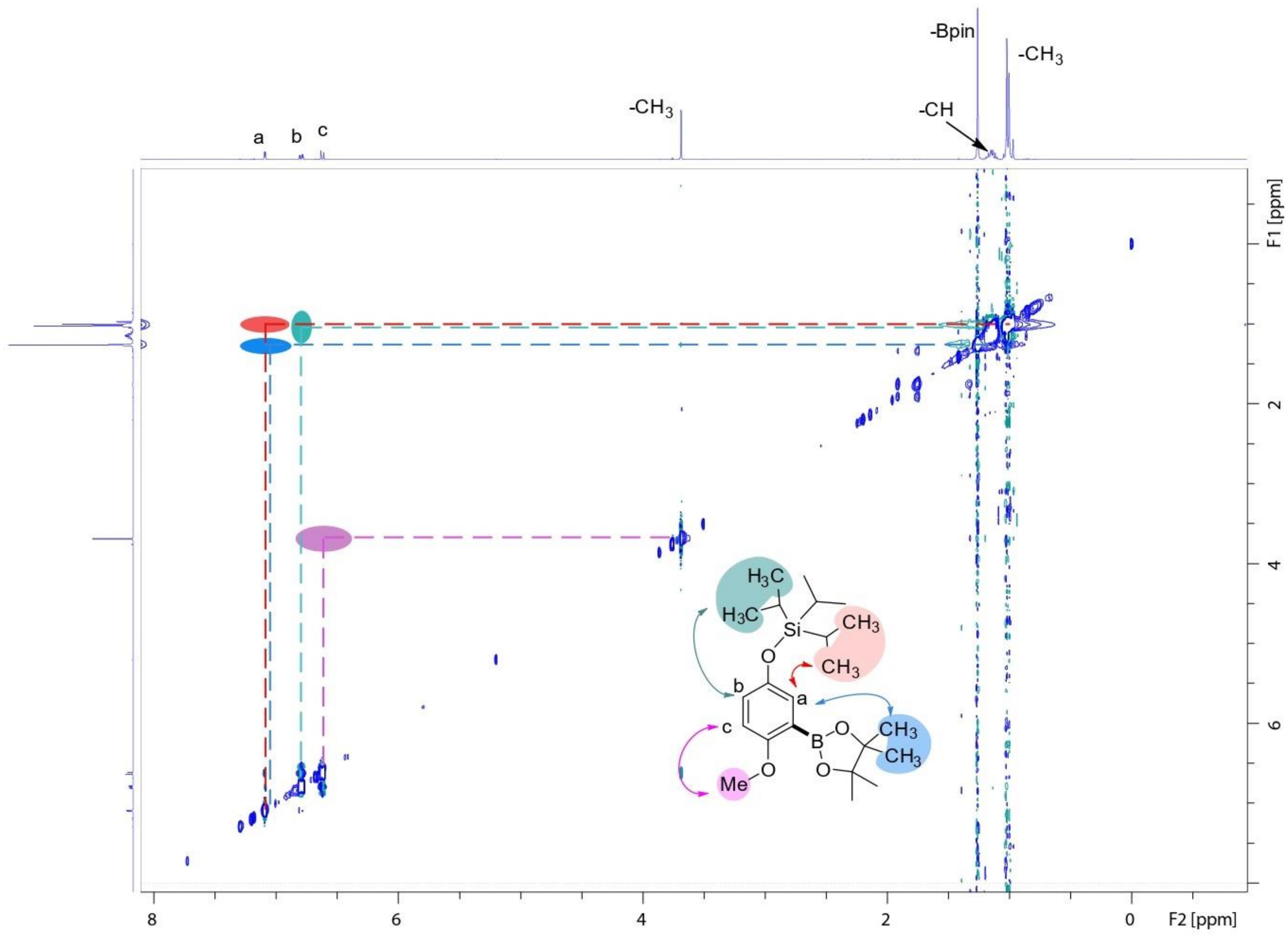

NOESY-NMR spectra of **7f** (25 ^{o}C, 100 MHz, $CDCl_3$)

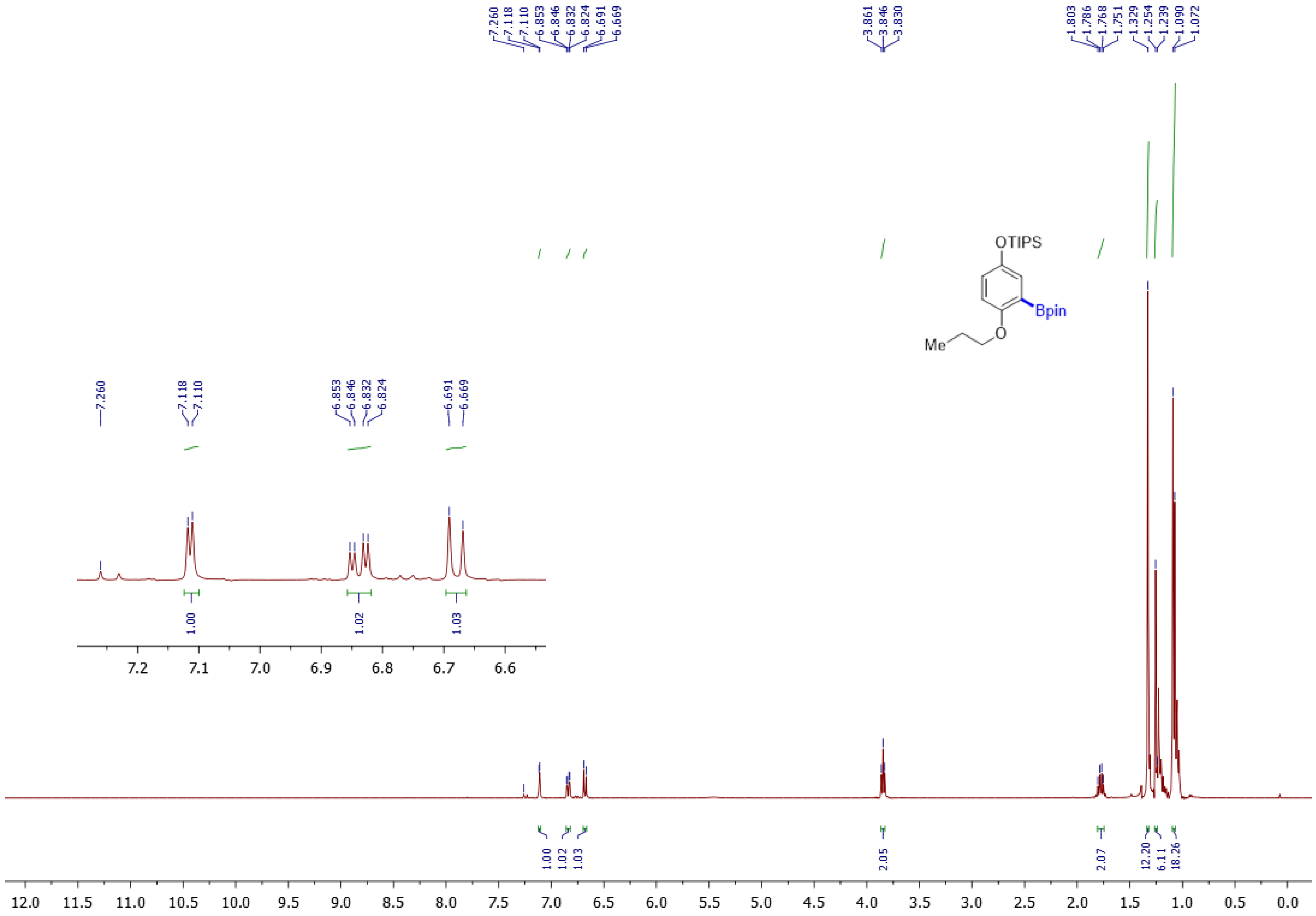

^{1}H-NMR spectra of **7g** (25 ^{o}C, 400 MHz, $CDCl_3$)

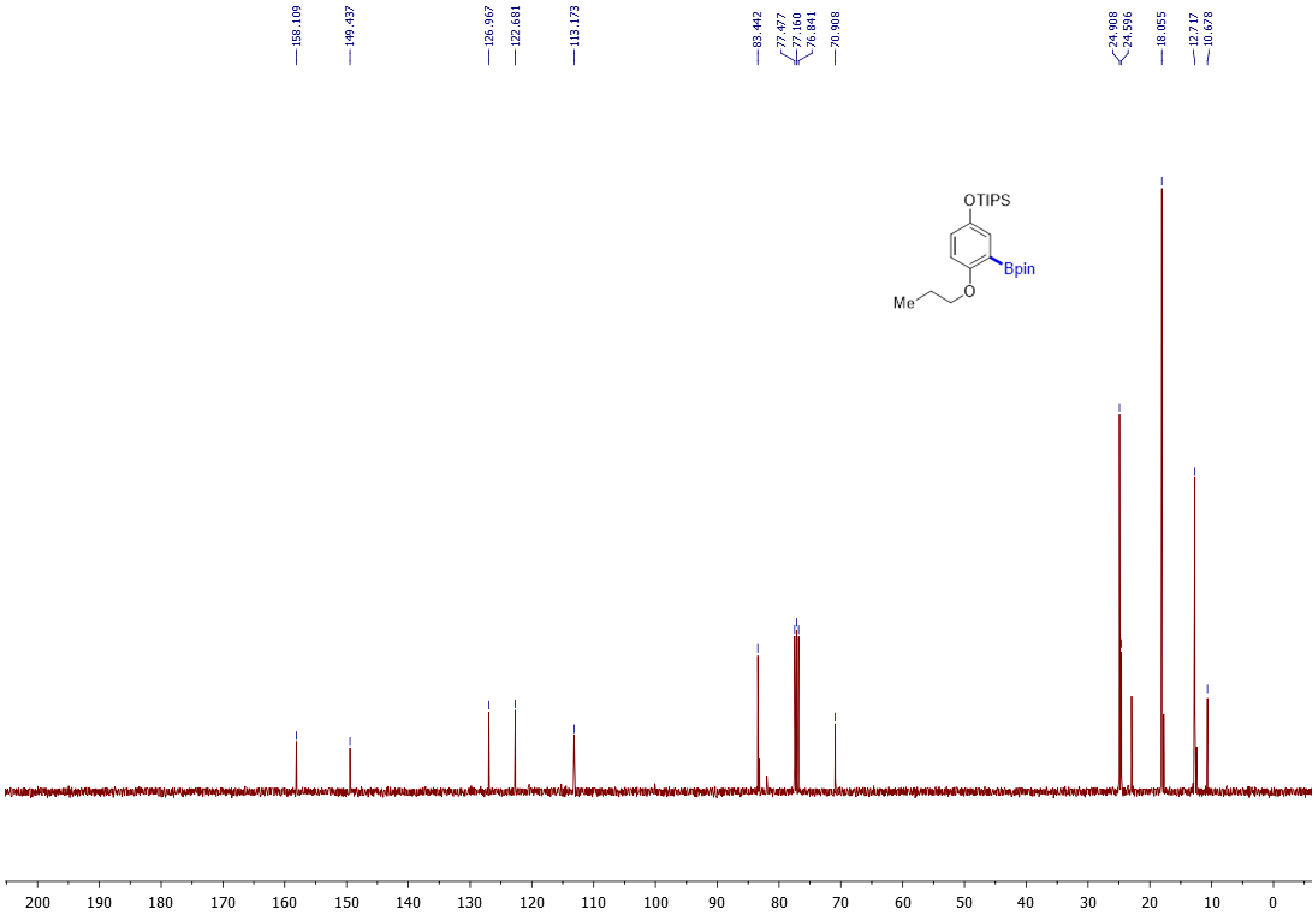

^{13}C-NMR spectra of **7g** (25 ^{o}C, 100 MHz, $CDCl_3$)

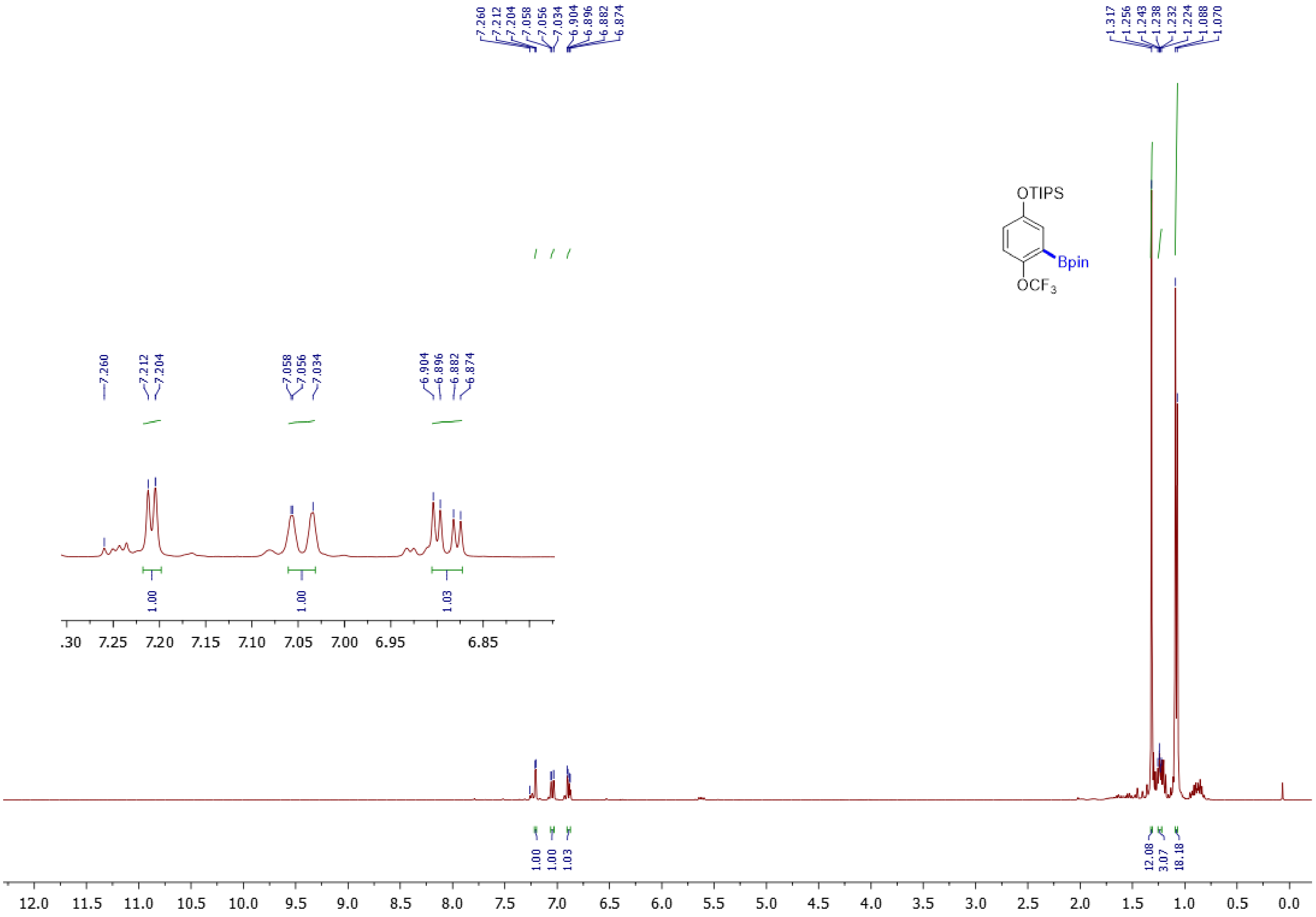

^{1}H-NMR spectra of **7h** (25 °C, 400 MHz, $CDCl_3$)

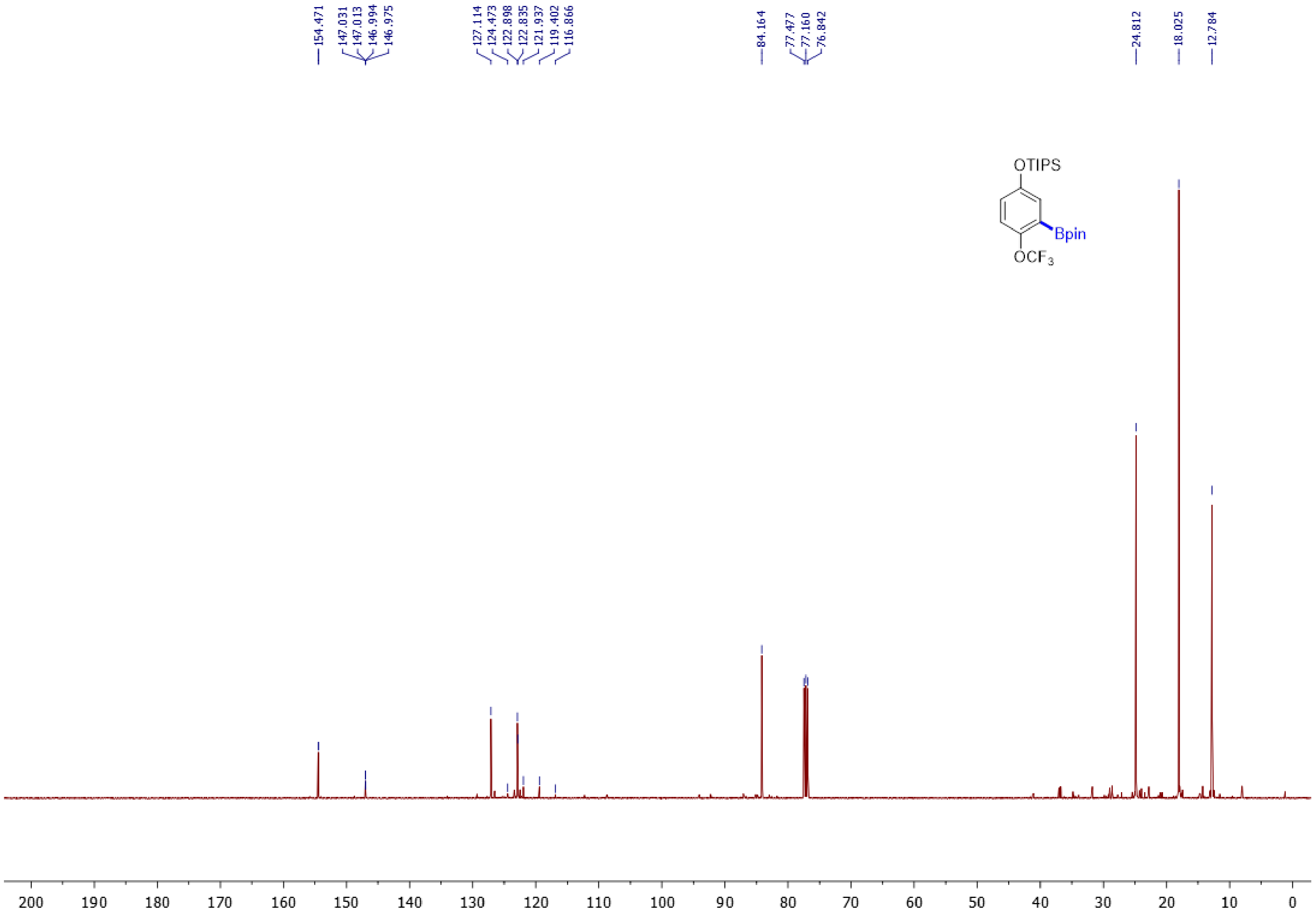

^{13}C-NMR spectra of **7h** (25 °C, 100 MHz, $CDCl_3$)

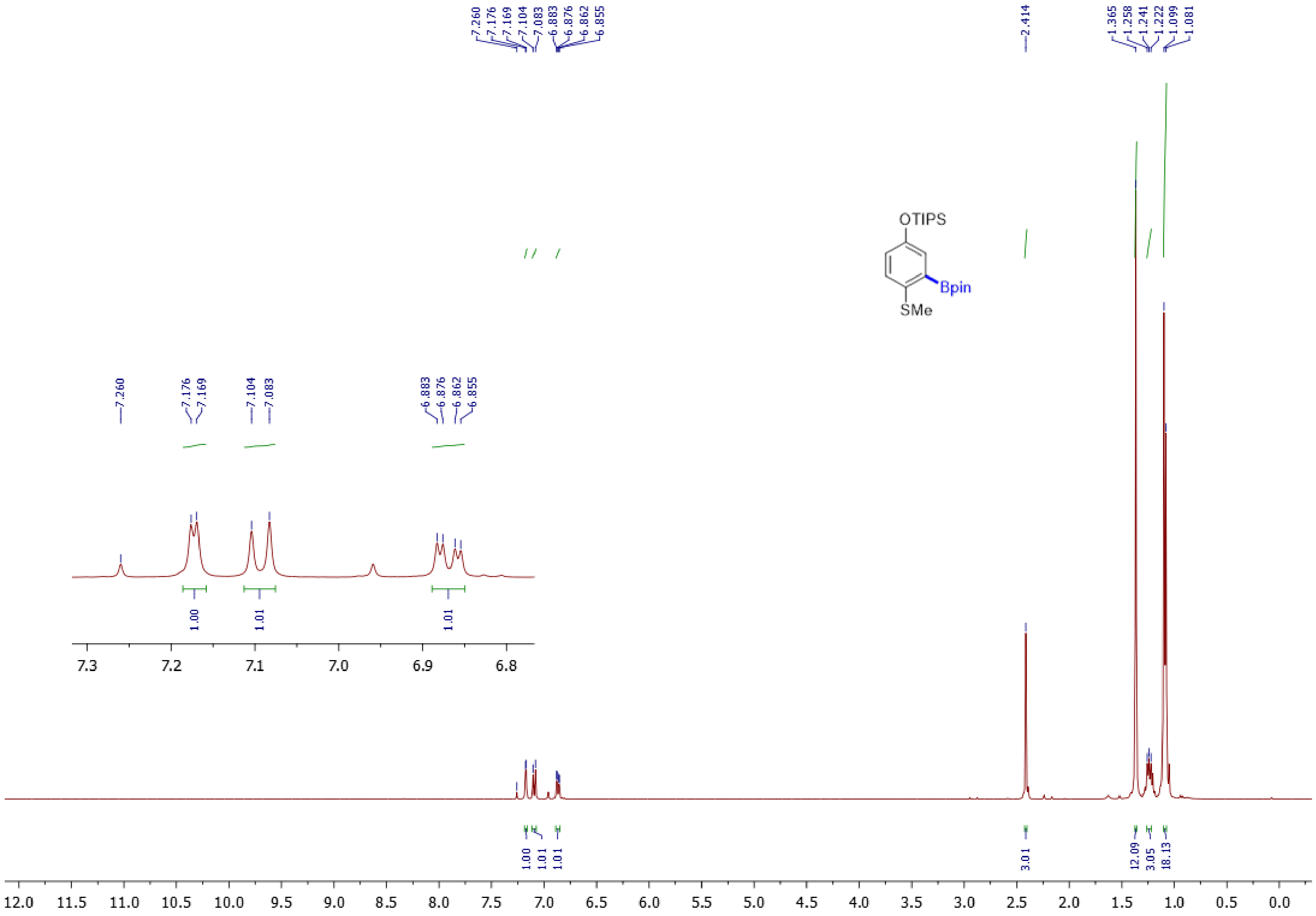

^{1}H-NMR spectra of **7j** (25 °C, 400 MHz, $CDCl_3$)

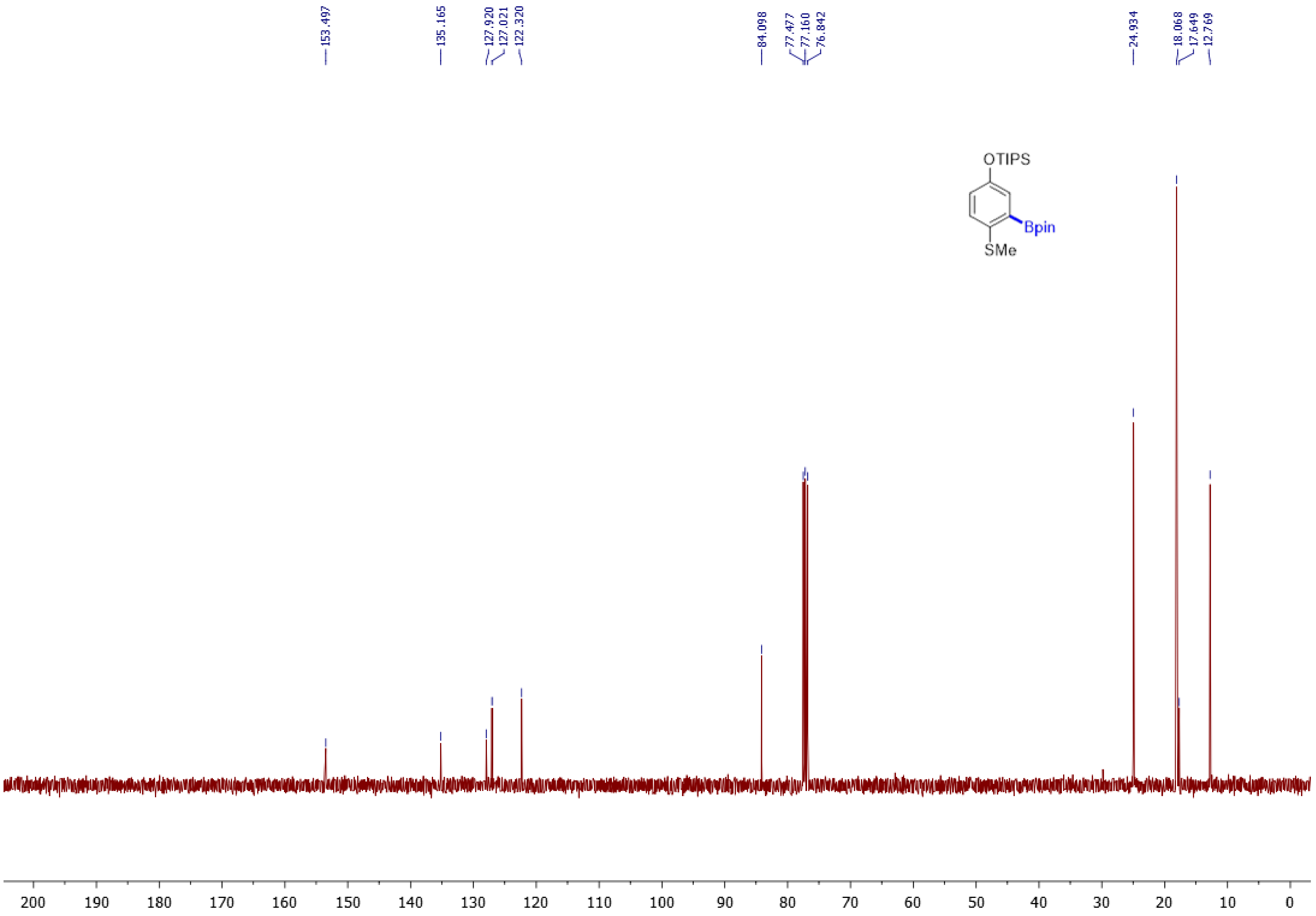

^{13}C-NMR spectra of **7j** (25 ^{o}C, 100 MHz, $CDCl_3$)

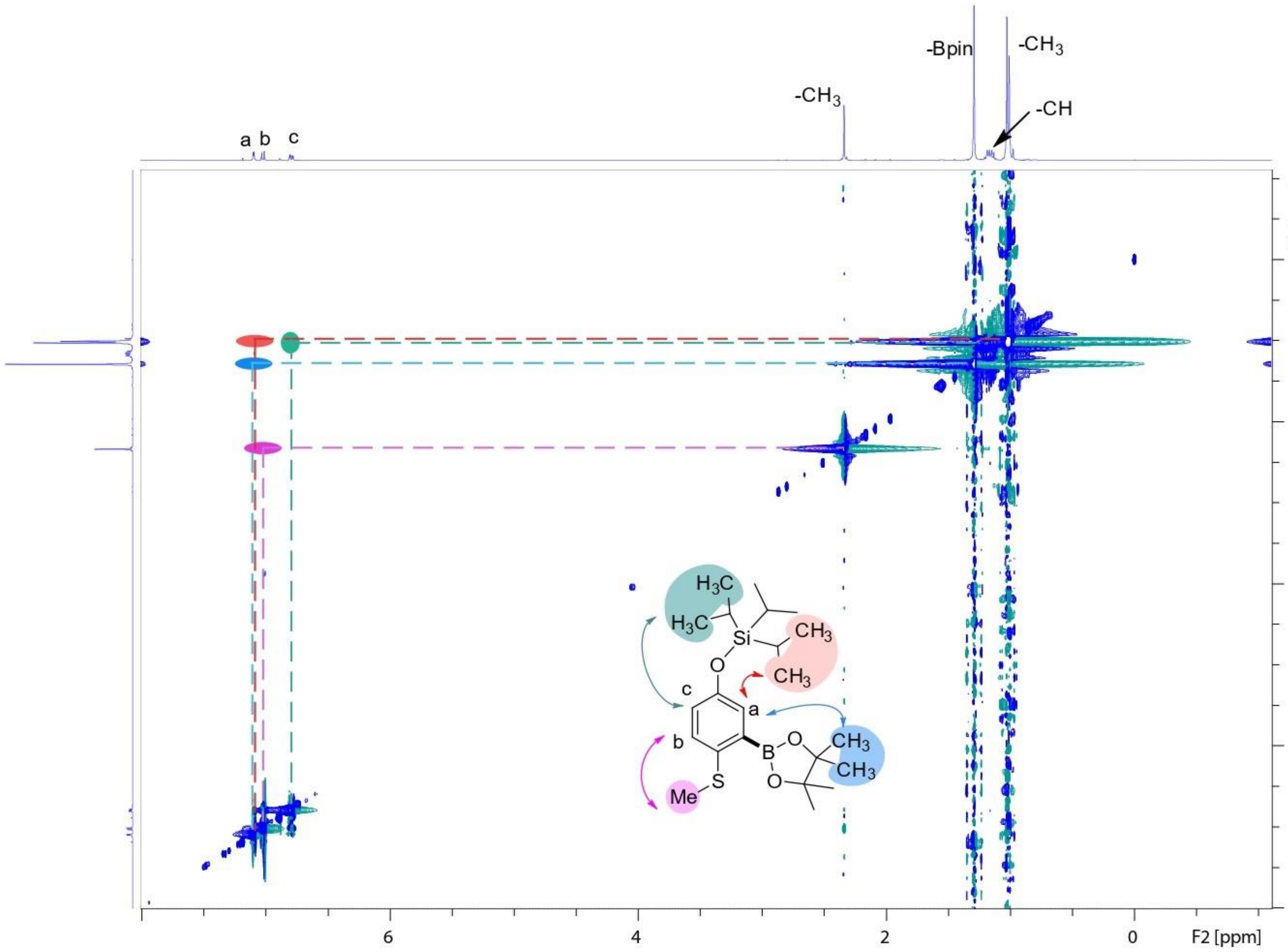

NOESY-NMR spectra of **7j** (25 °C, 100 MHz, $CDCl_3$)

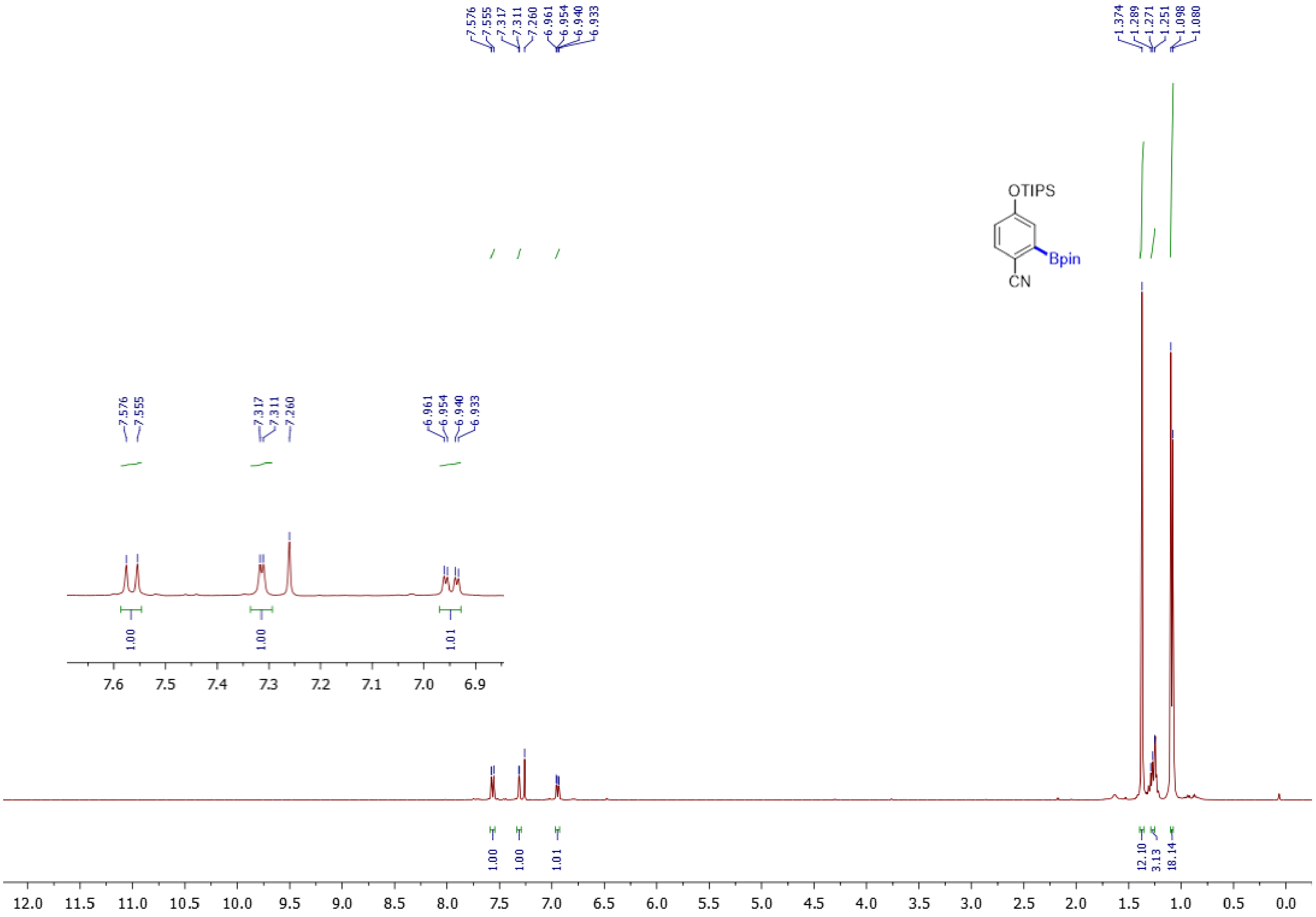

^{1}H-NMR spectra of **7k** (25 ^{o}C, 400 MHz, $CDCl_3$)

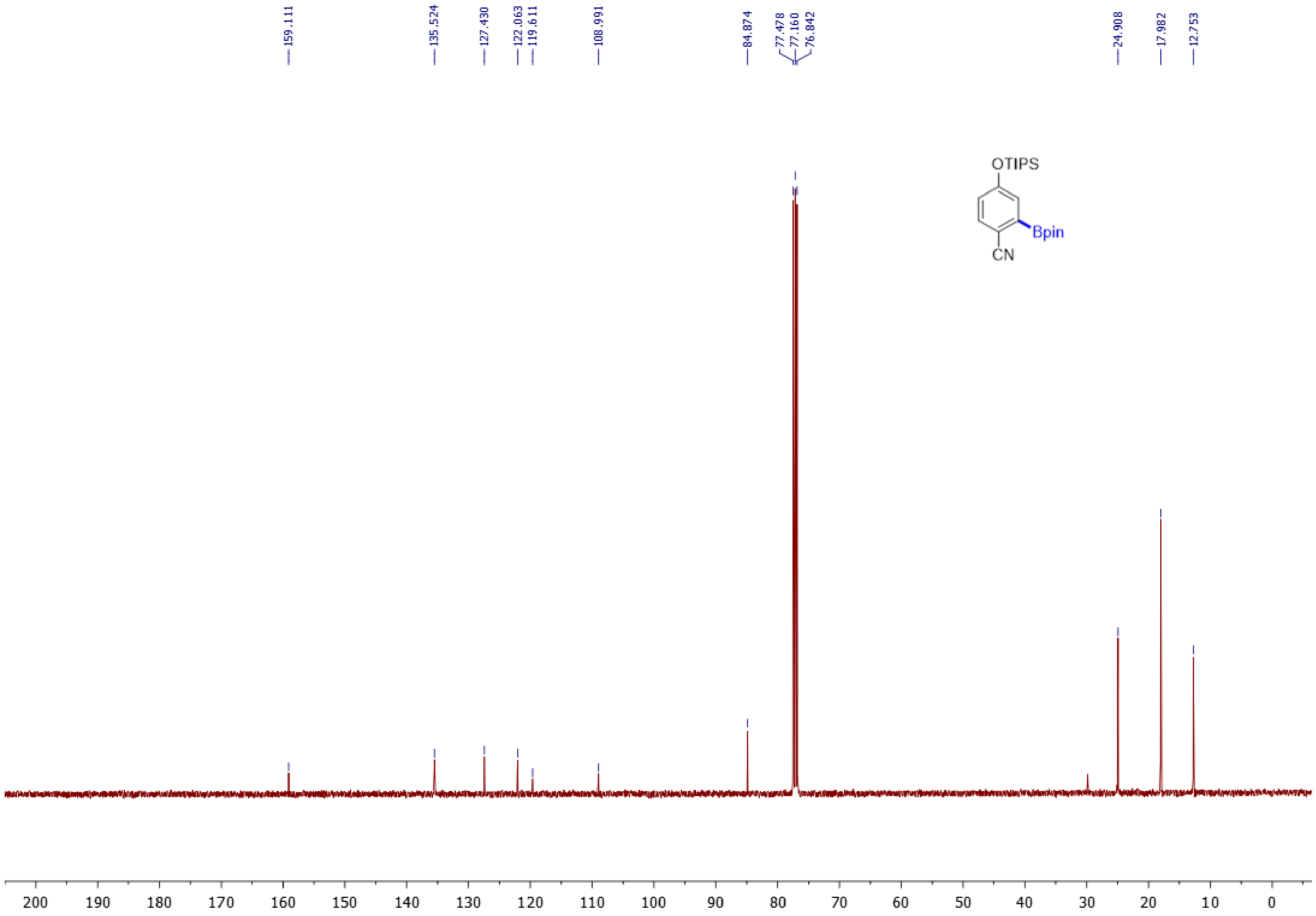

^{13}C-NMR spectra of **7k** (25 ^{o}C, 100 MHz, $CDCl_3$)

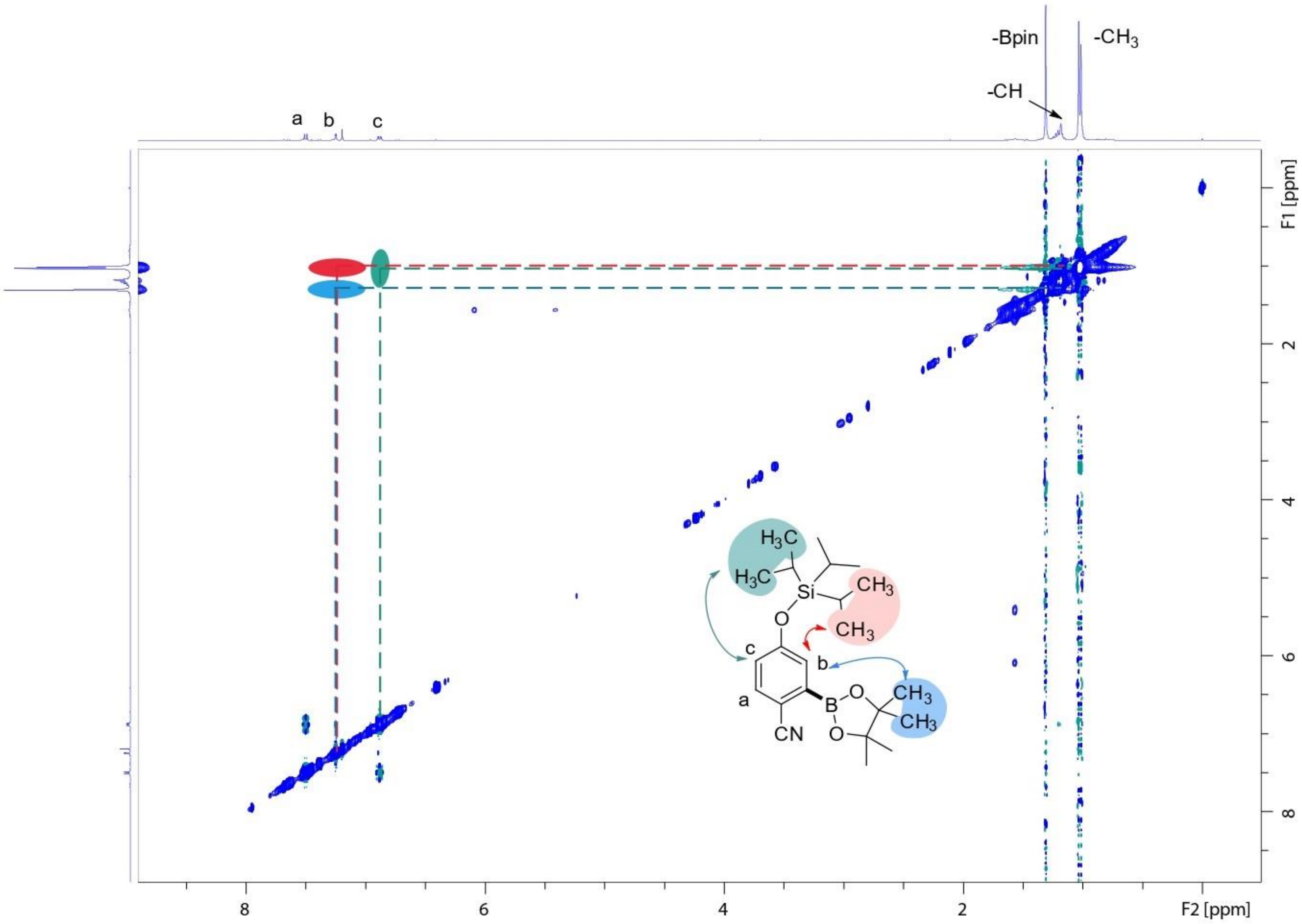

NOESY-NMR spectra of **7k** (25 ^{o}C, 100 MHz, $CDCl_3$)

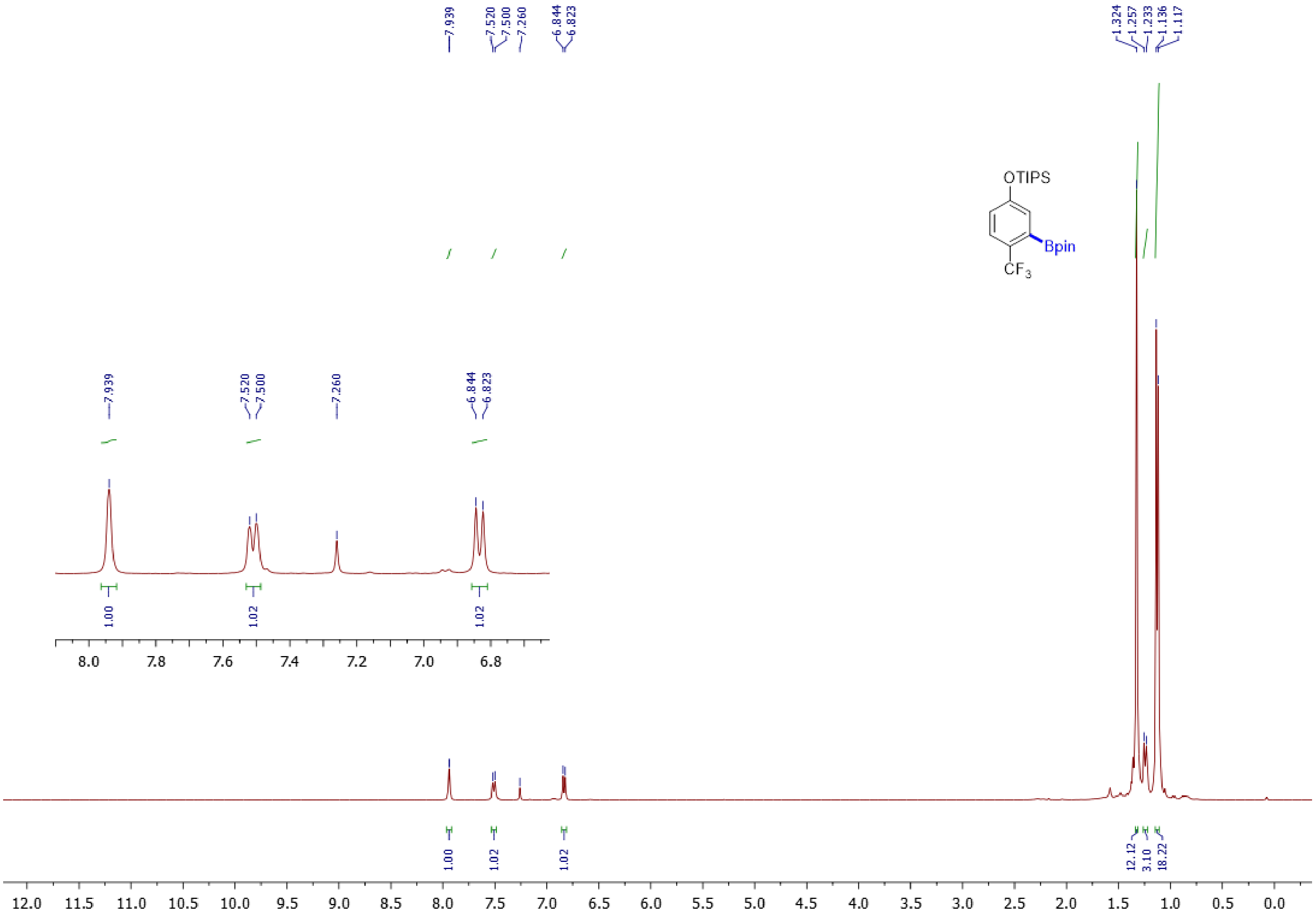

^{1}H-NMR spectra of **7l** (25 °C, 400 MHz, $CDCl_3$)

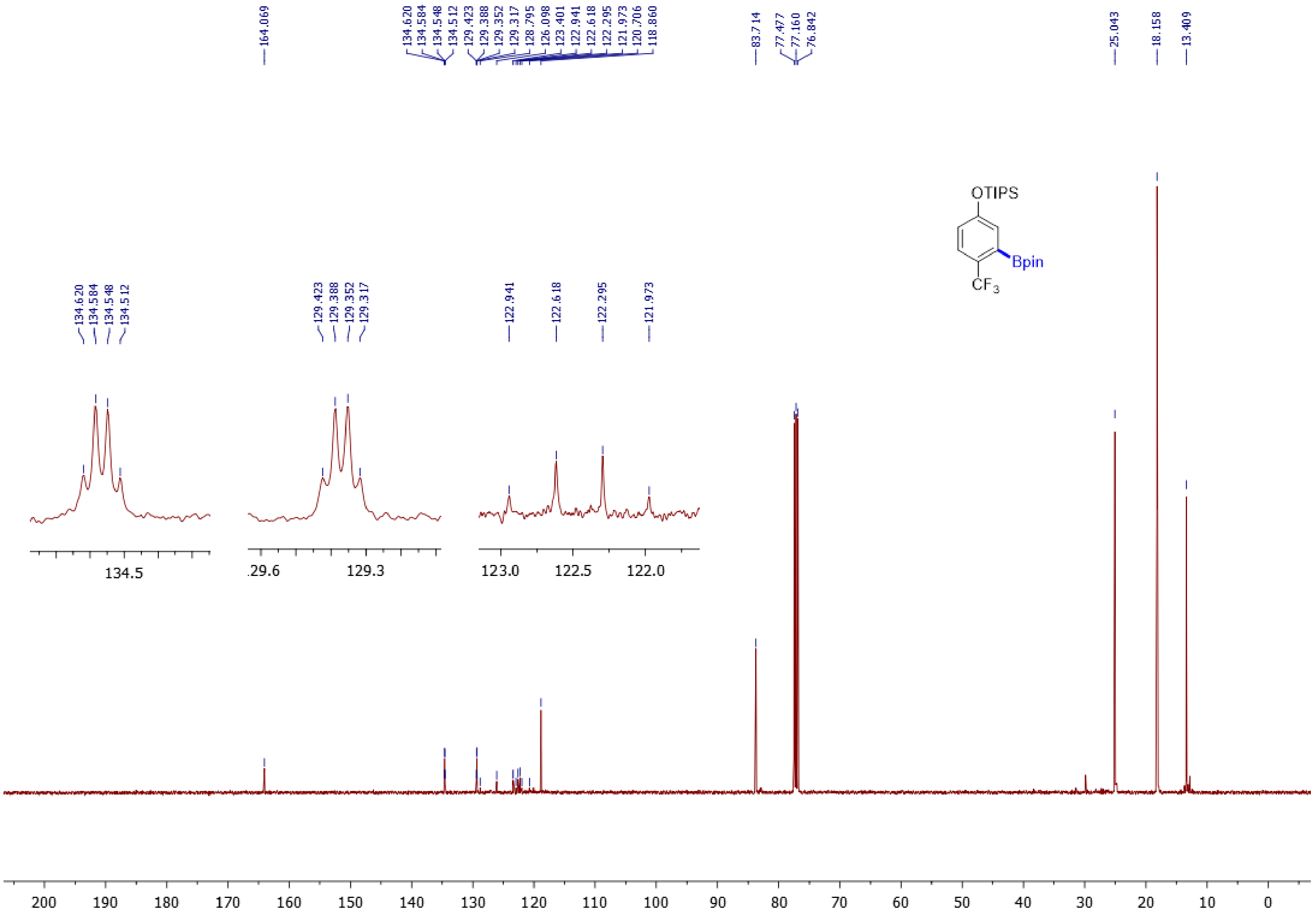

^{13}C-NMR spectra of **7l** (25 ^{o}C, 100 MHz, $CDCl_3$)

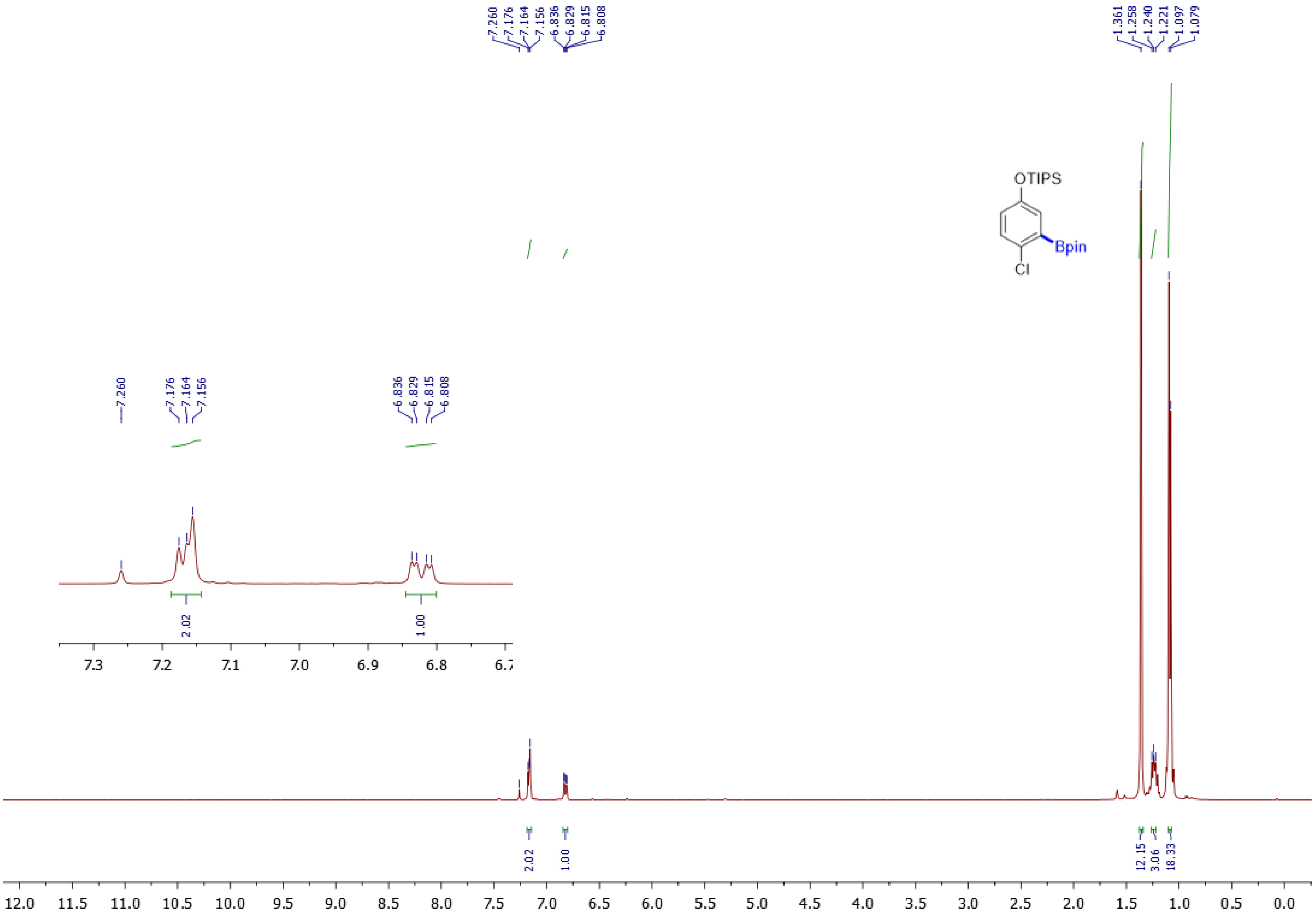

^{1}H-NMR spectra of **7m** (25 °C, 400 MHz, $CDCl_3$)

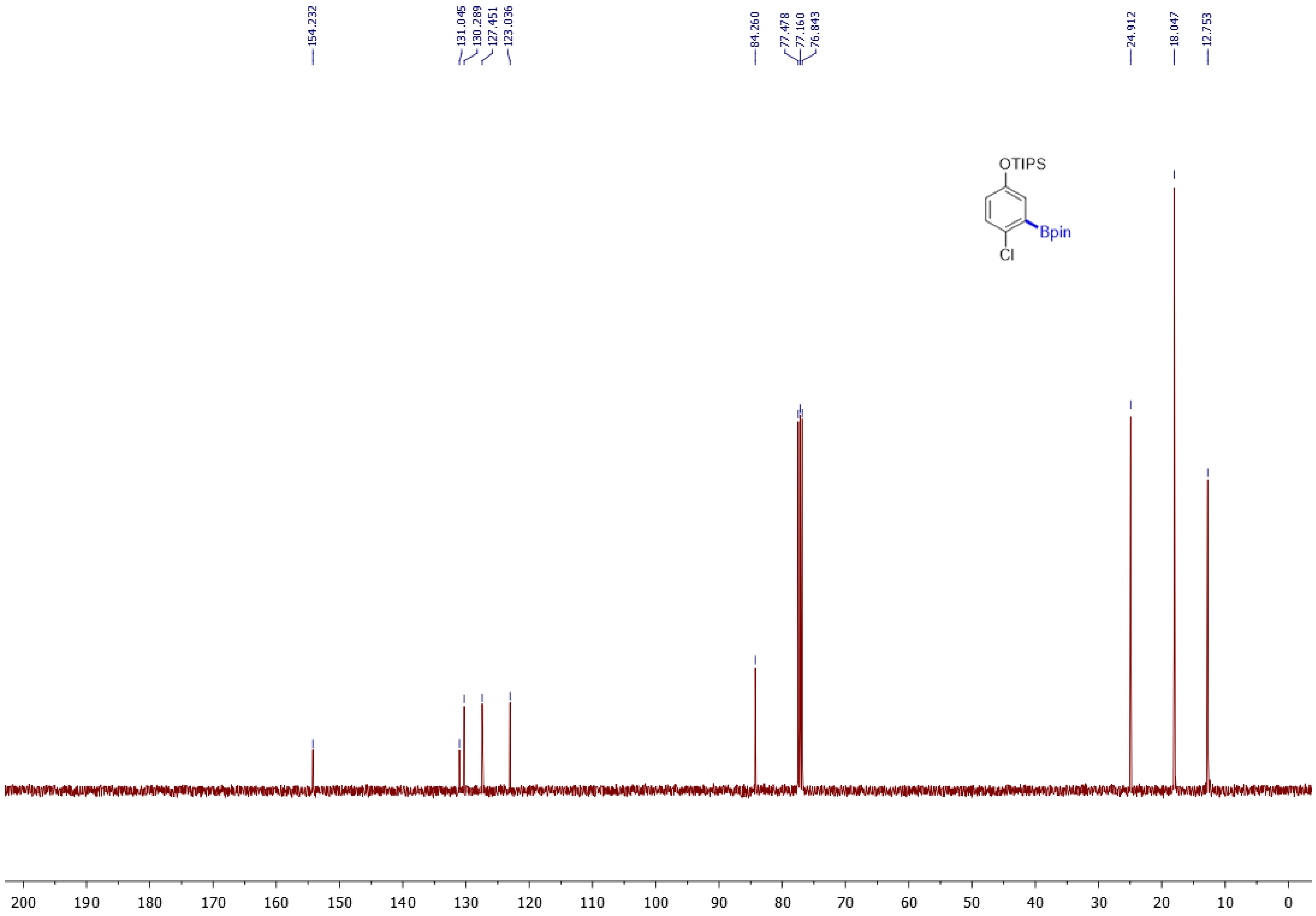

^{13}C-NMR spectra of **7m** (25 ^{o}C, 100 MHz, $CDCl_3$)

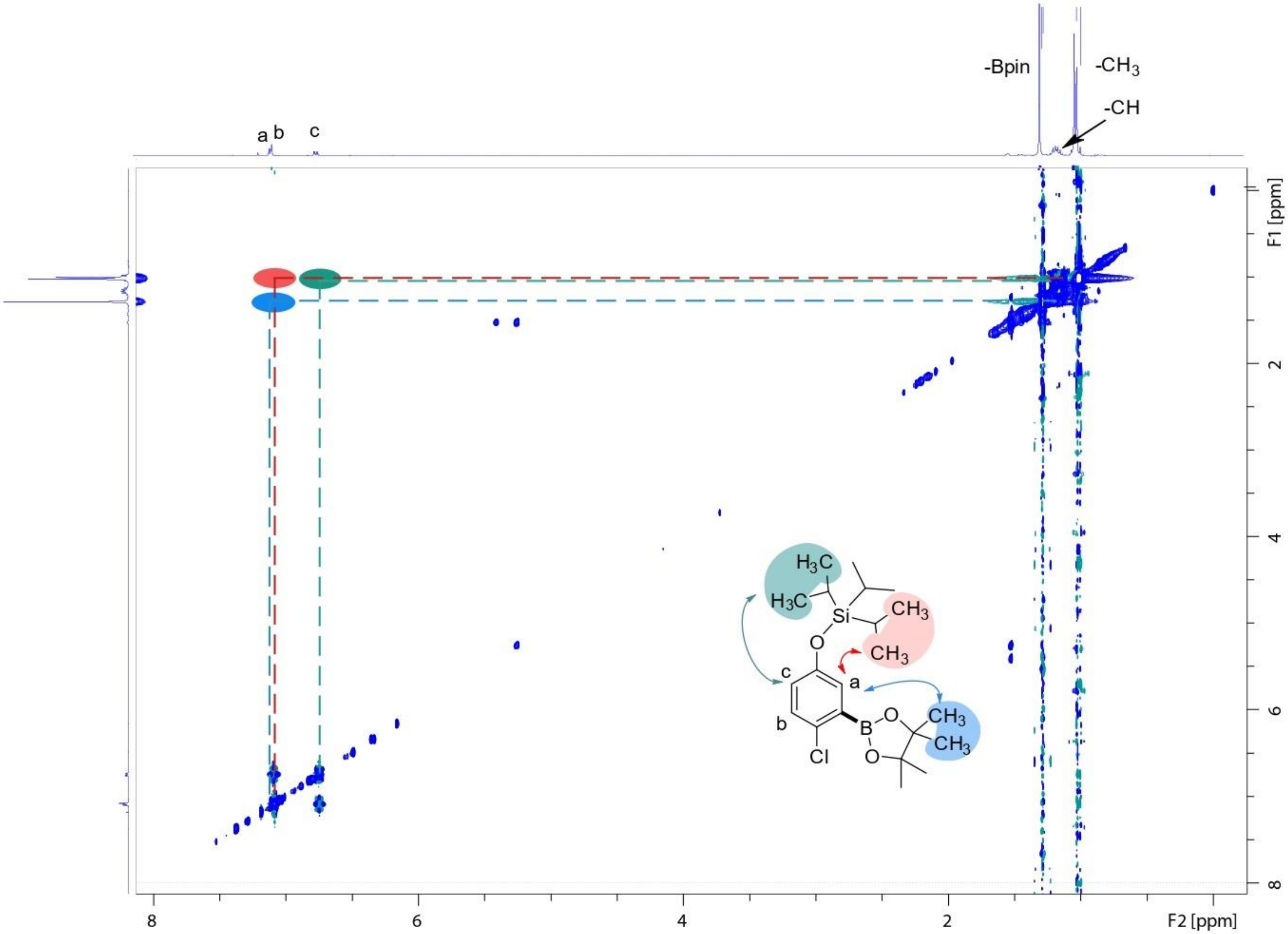

NOESY-NMR spectra of **7m** (25 ^{o}C, 100 MHz, $CDCl_3$)

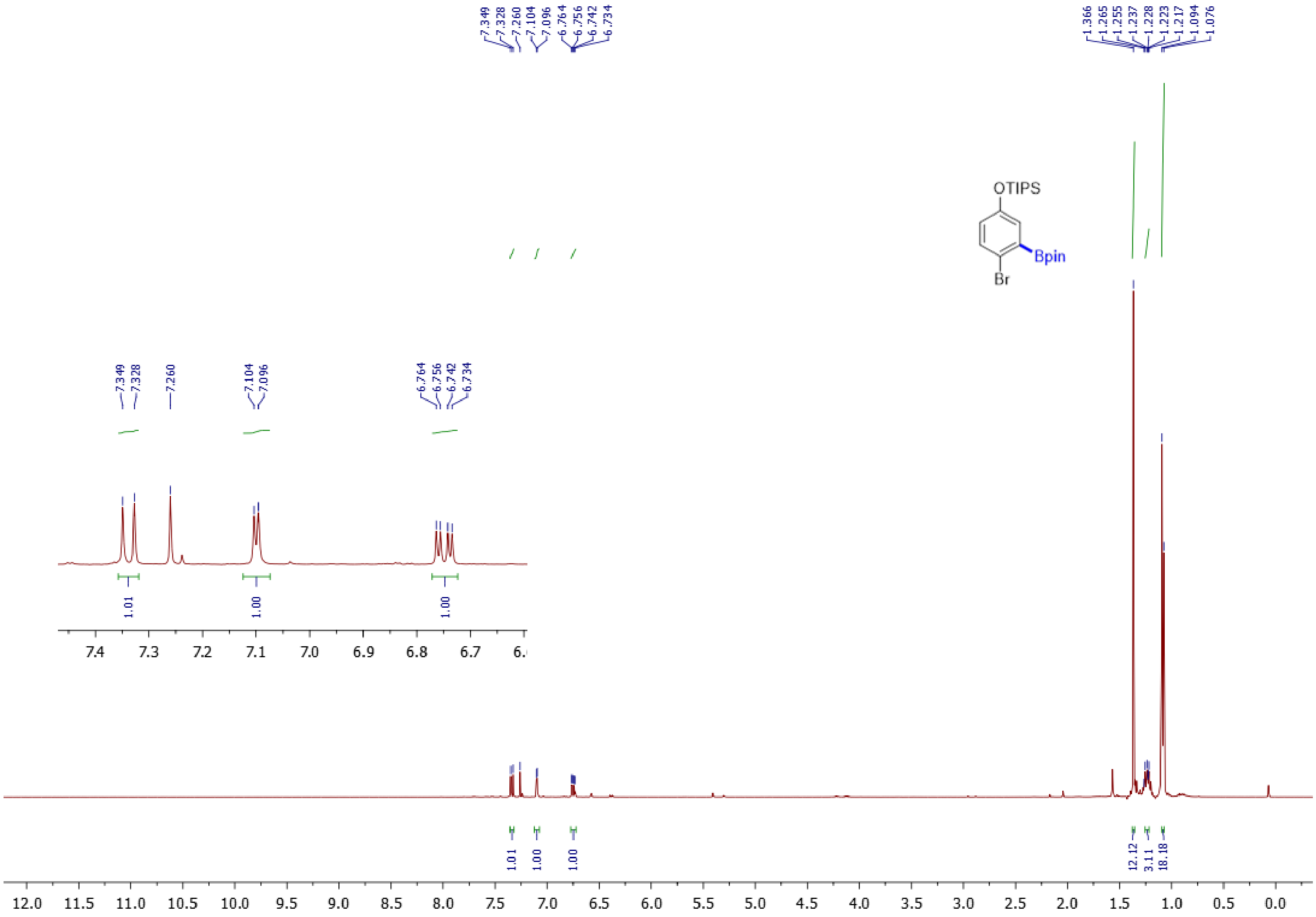

^{1}H-NMR spectra of **7n** (25 °C, 400 MHz, $CDCl_3$)

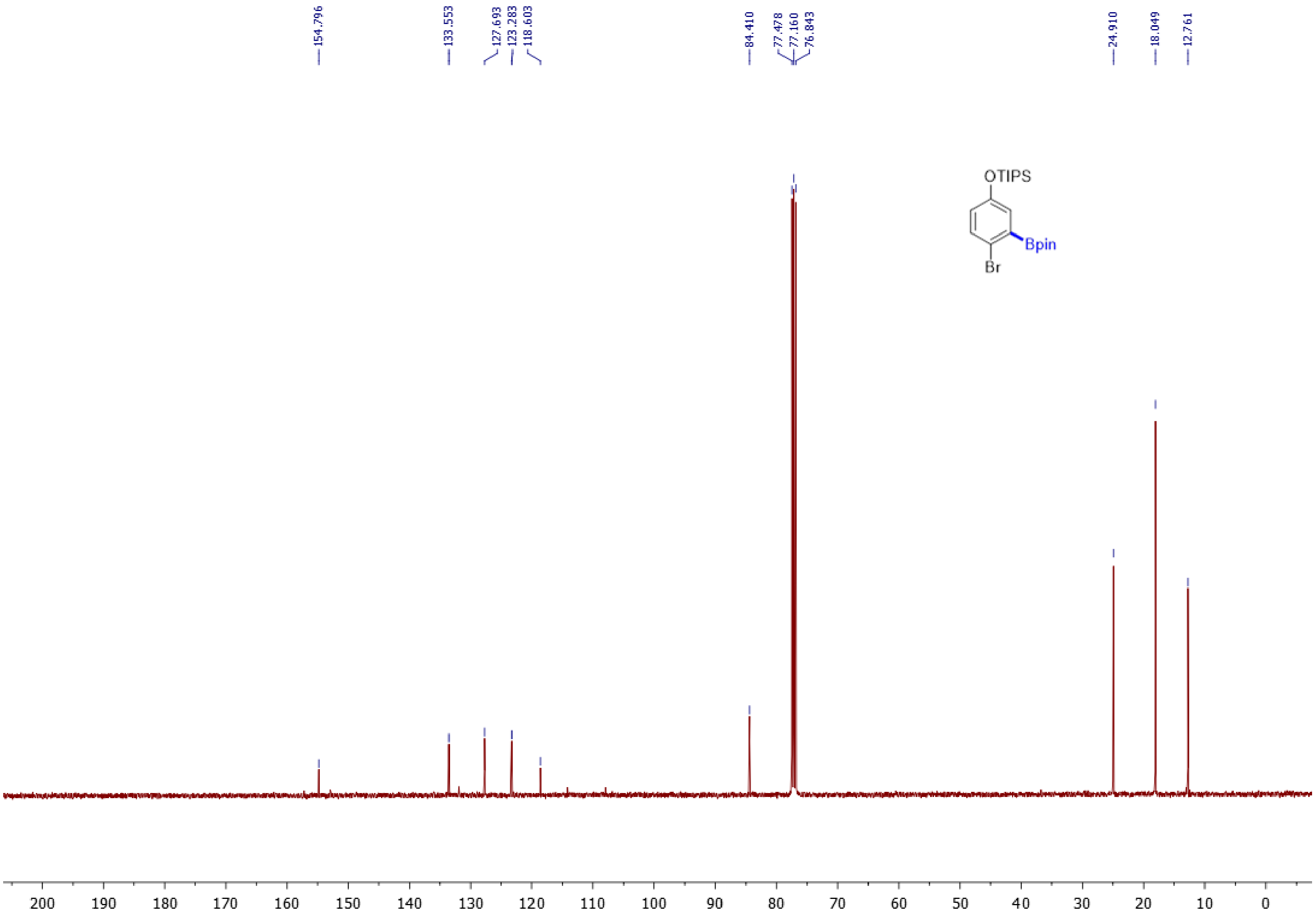

^{13}C-NMR spectra of **7n** (25 °C, 100 MHz, $CDCl_3$)

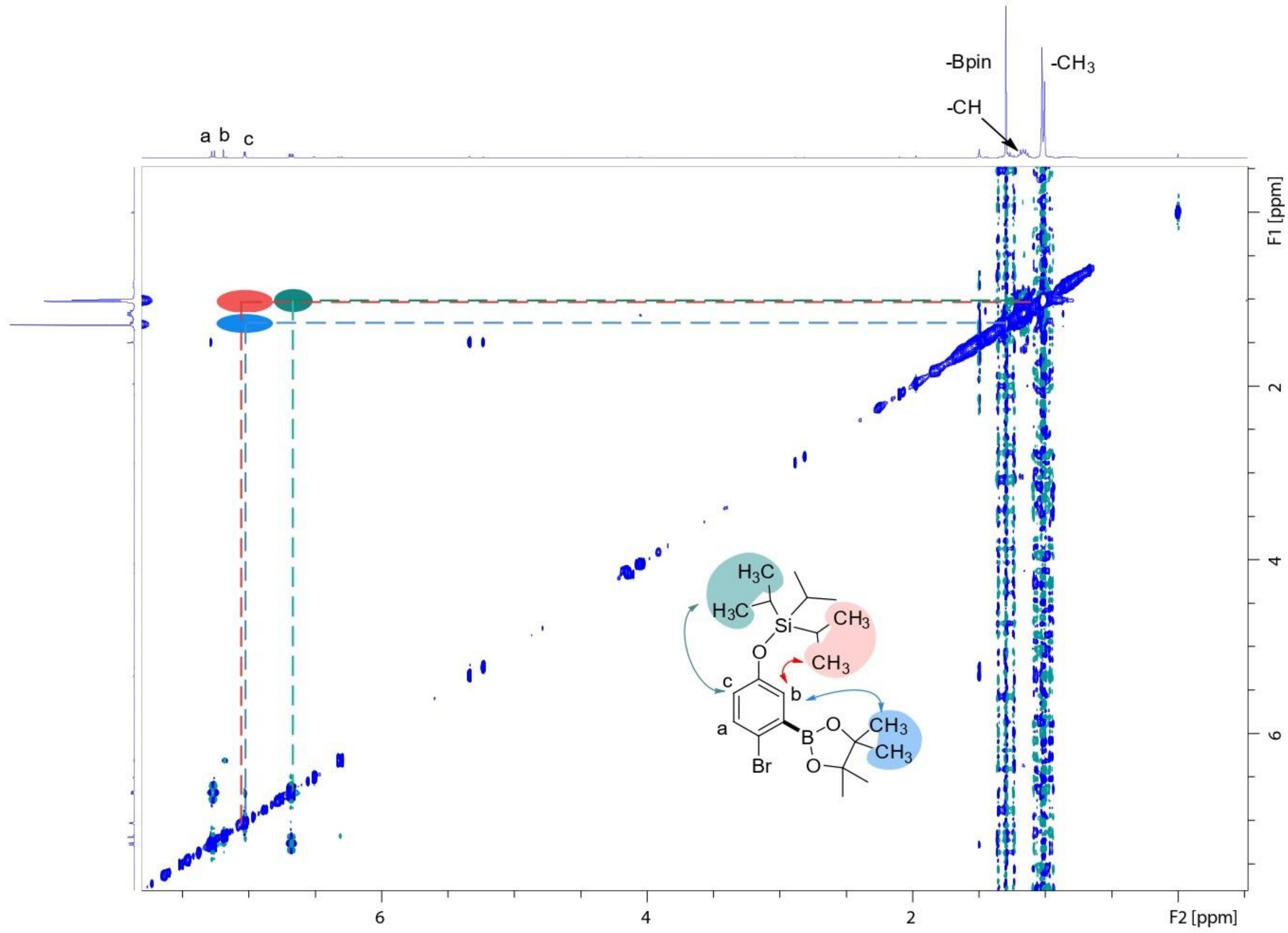

NOESY-NMR spectra of **7n** (25 ^{o}C, 100 MHz, $CDCl_3$)

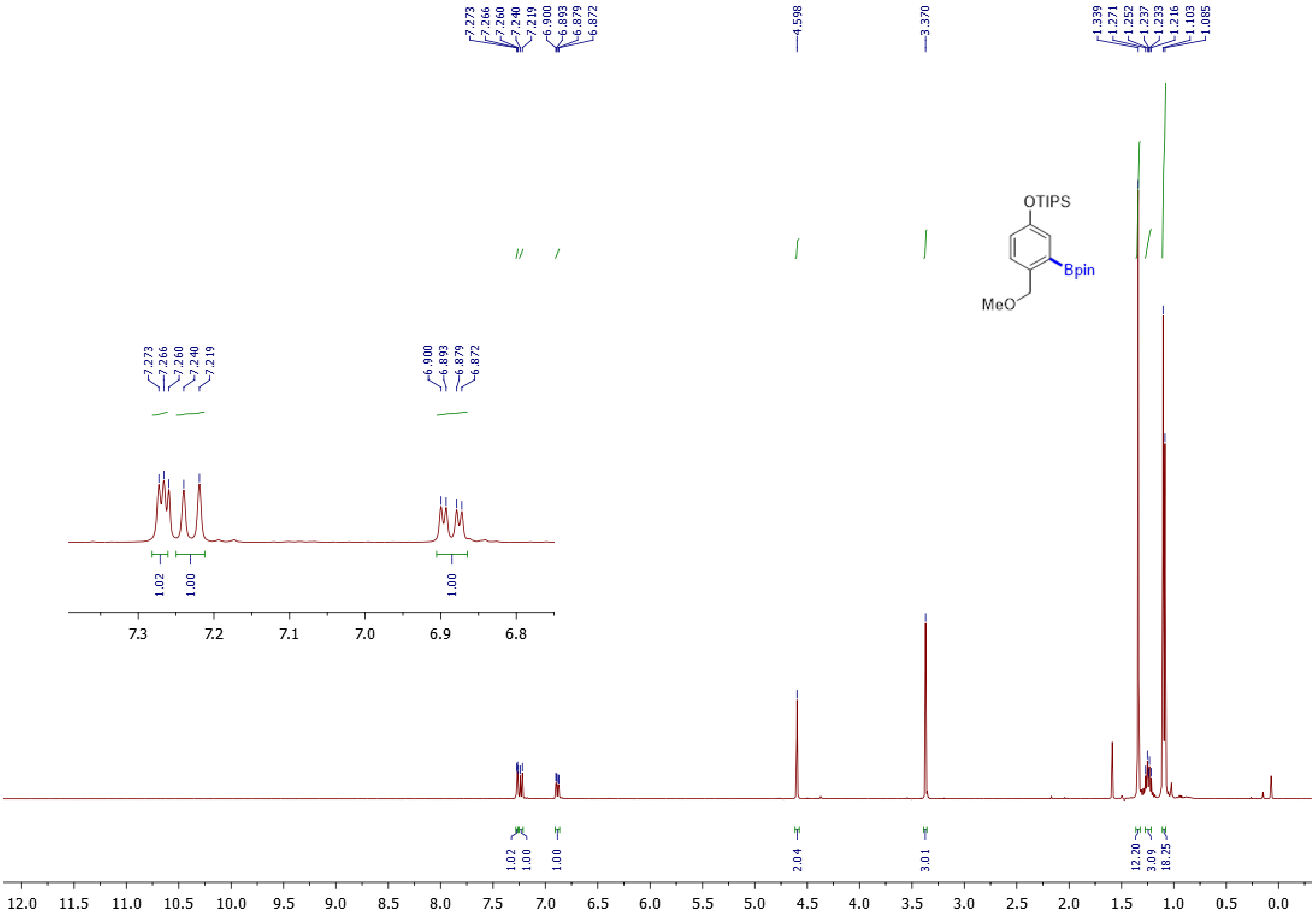

^{1}H-NMR spectra of **7p** (25 °C, 400 MHz, $CDCl_3$)

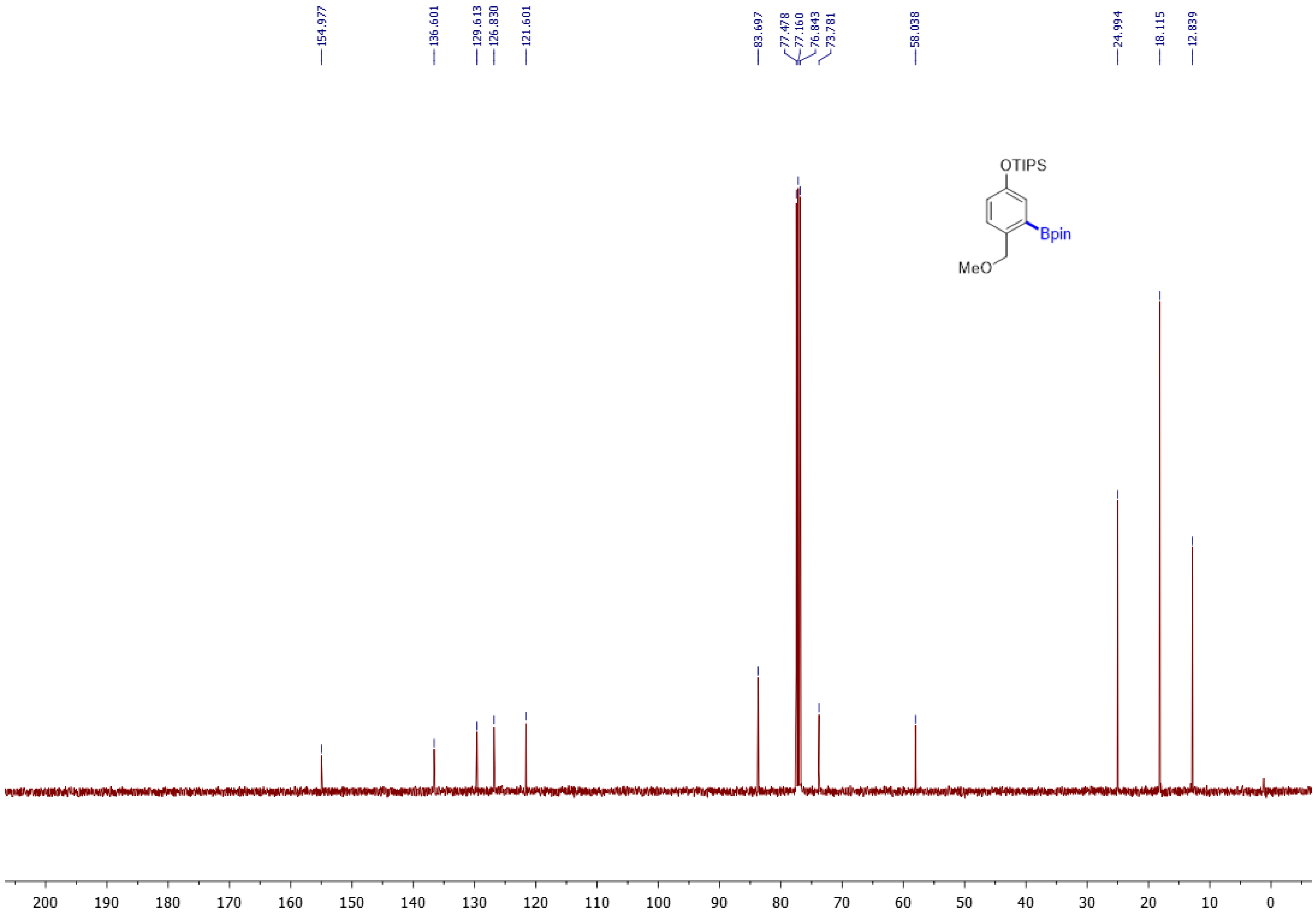

^{13}C-NMR spectra of **7p** (25 °C, 100 MHz, $CDCl_3$)

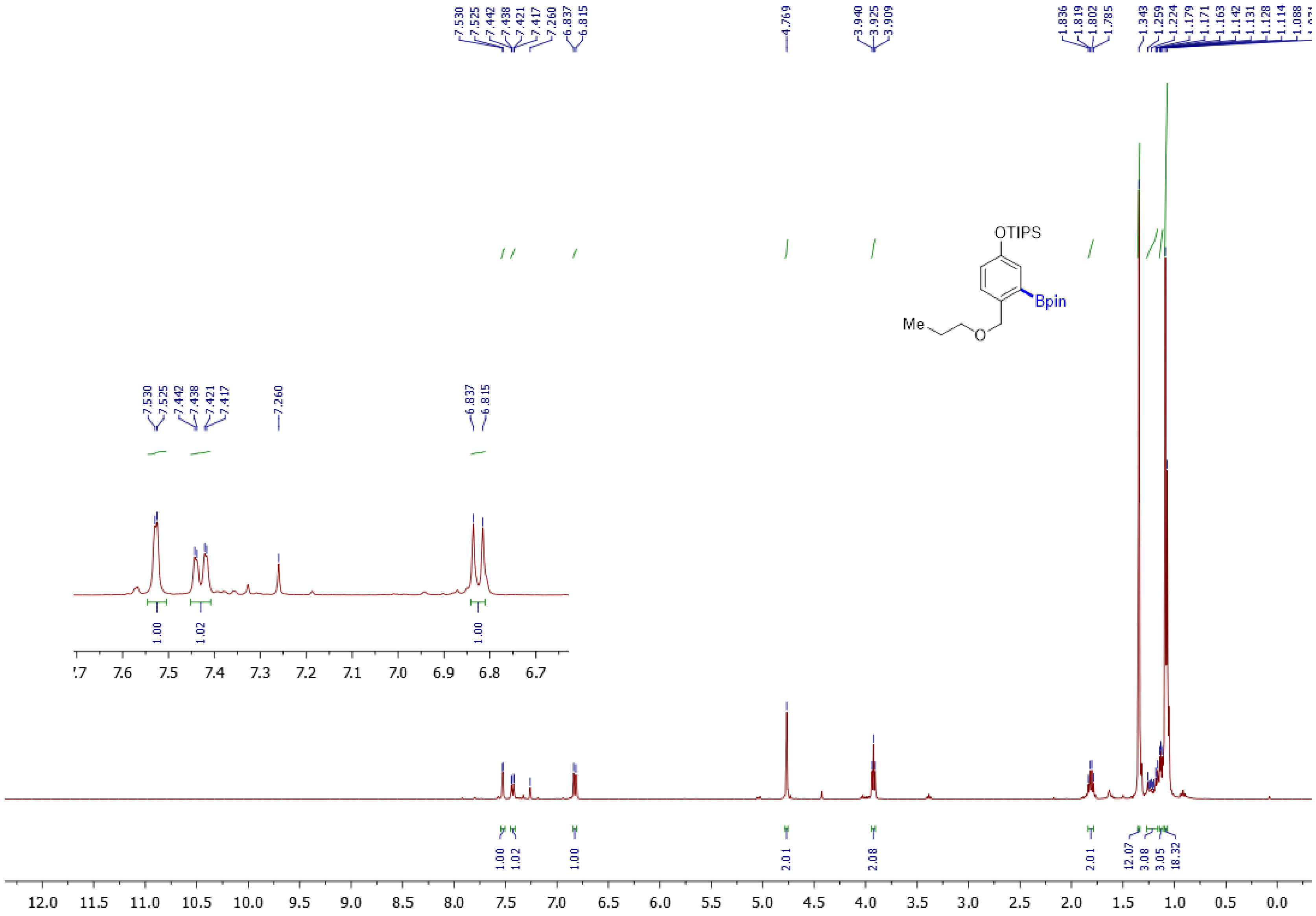

^{1}H-NMR spectra of **7q** (25 ^{o}C, 400 MHz, $CDCl_3$)

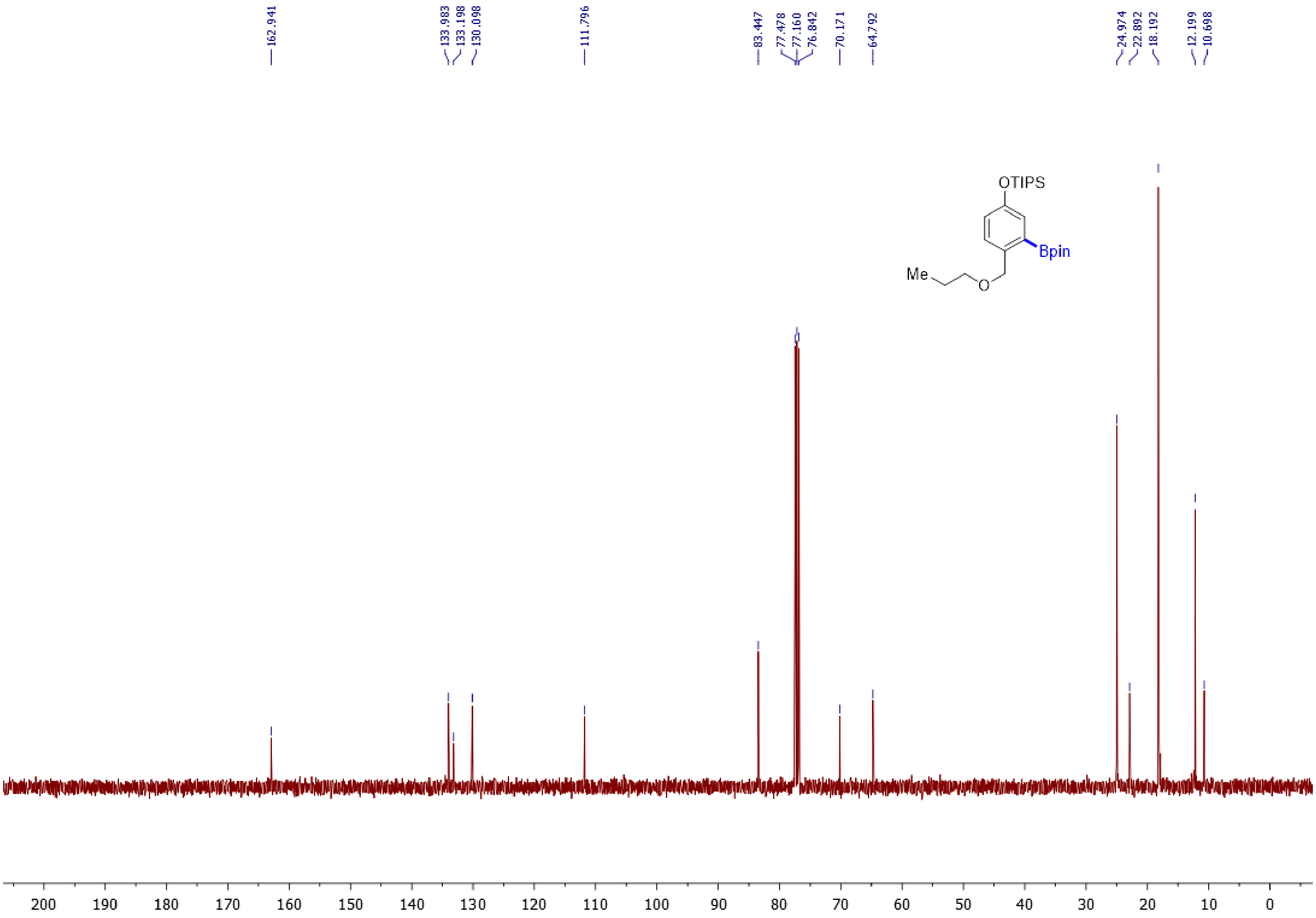

^{13}C-NMR spectra of **7q** (25 °C, 100 MHz, $CDCl_3$)

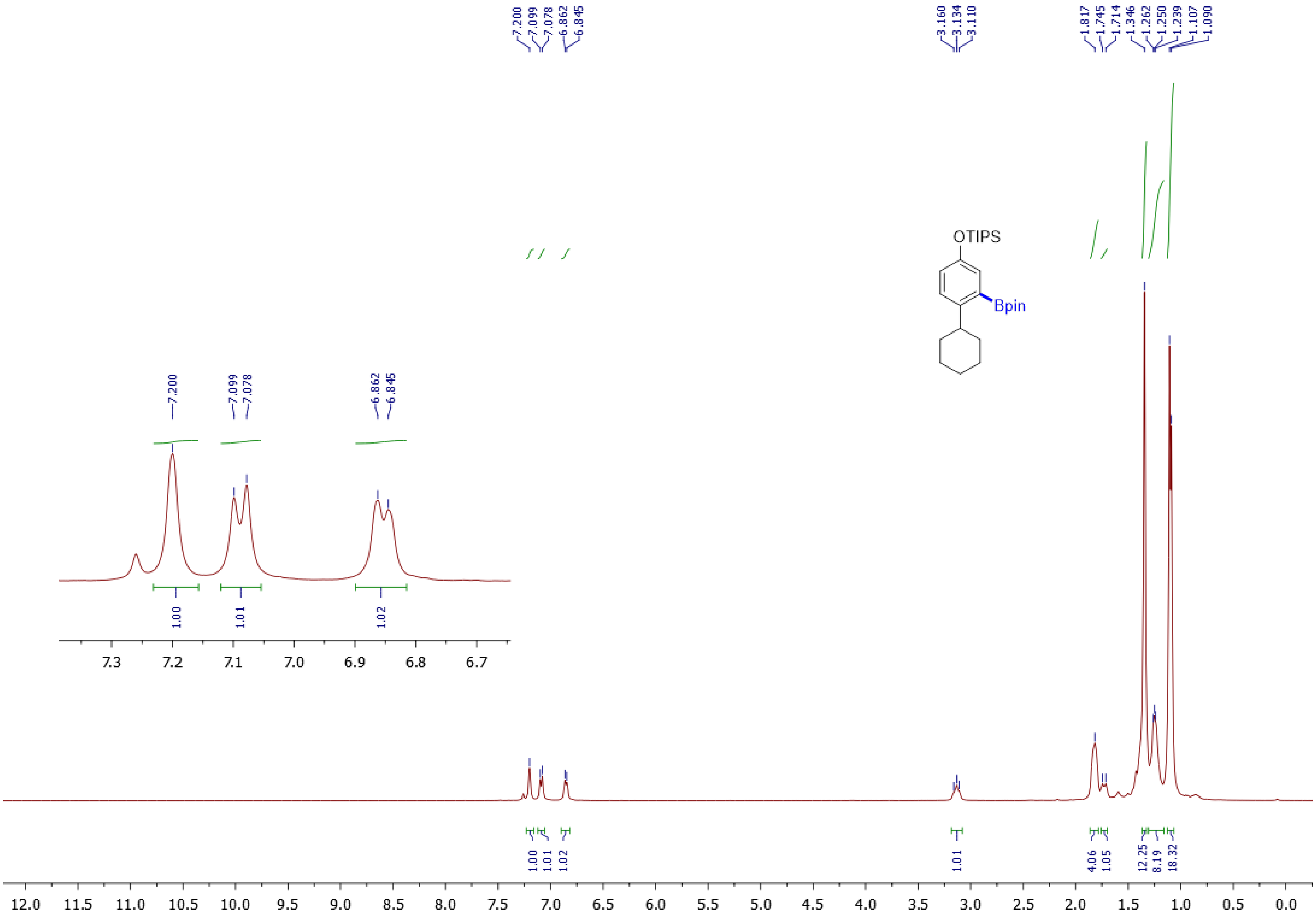

^{1}H-NMR spectra of **7r** (25 ^{o}C, 400 MHz, $CDCl_3$)

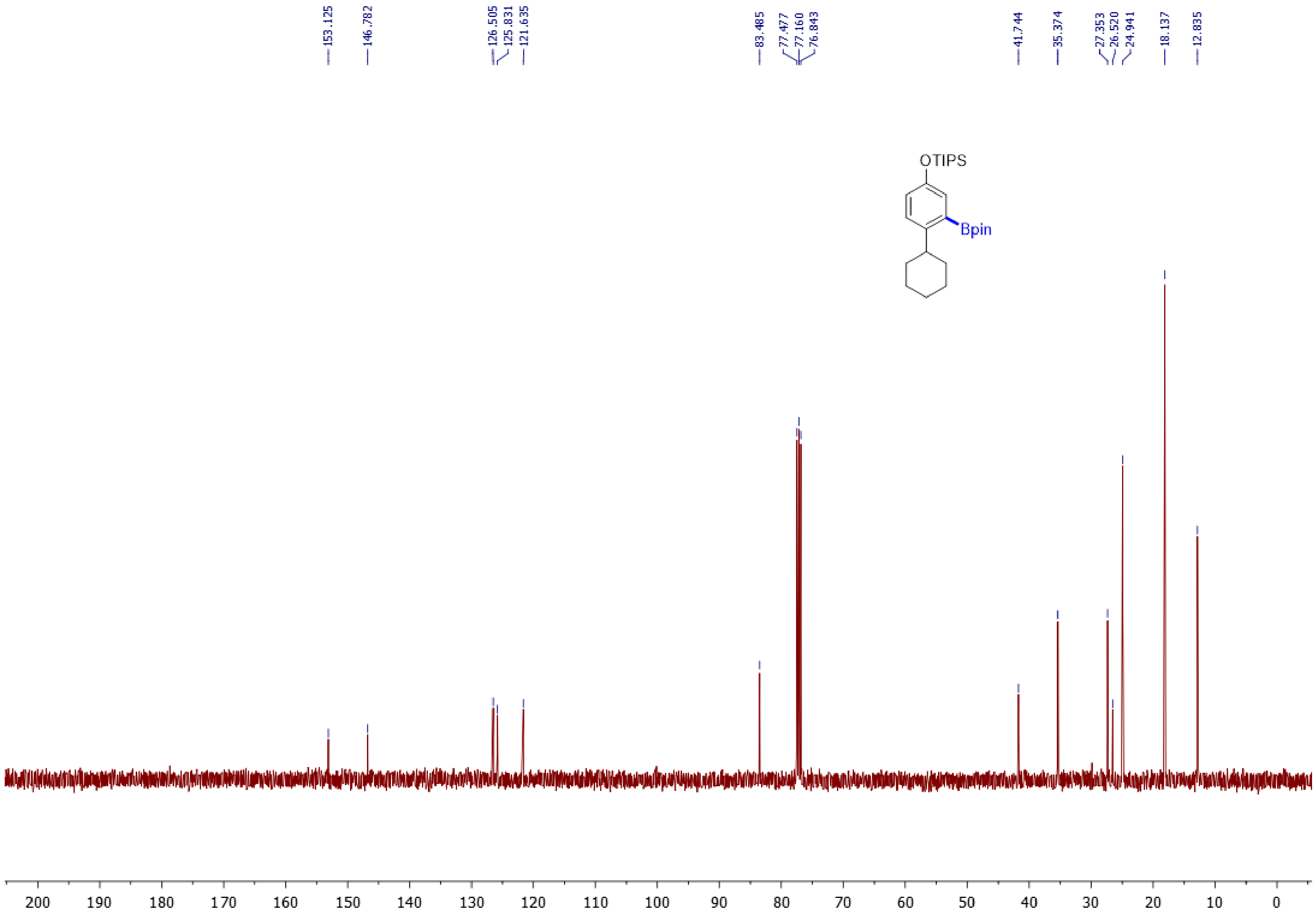

^{13}C-NMR spectra of **7r** (25 °C, 100 MHz, $CDCl_3$)

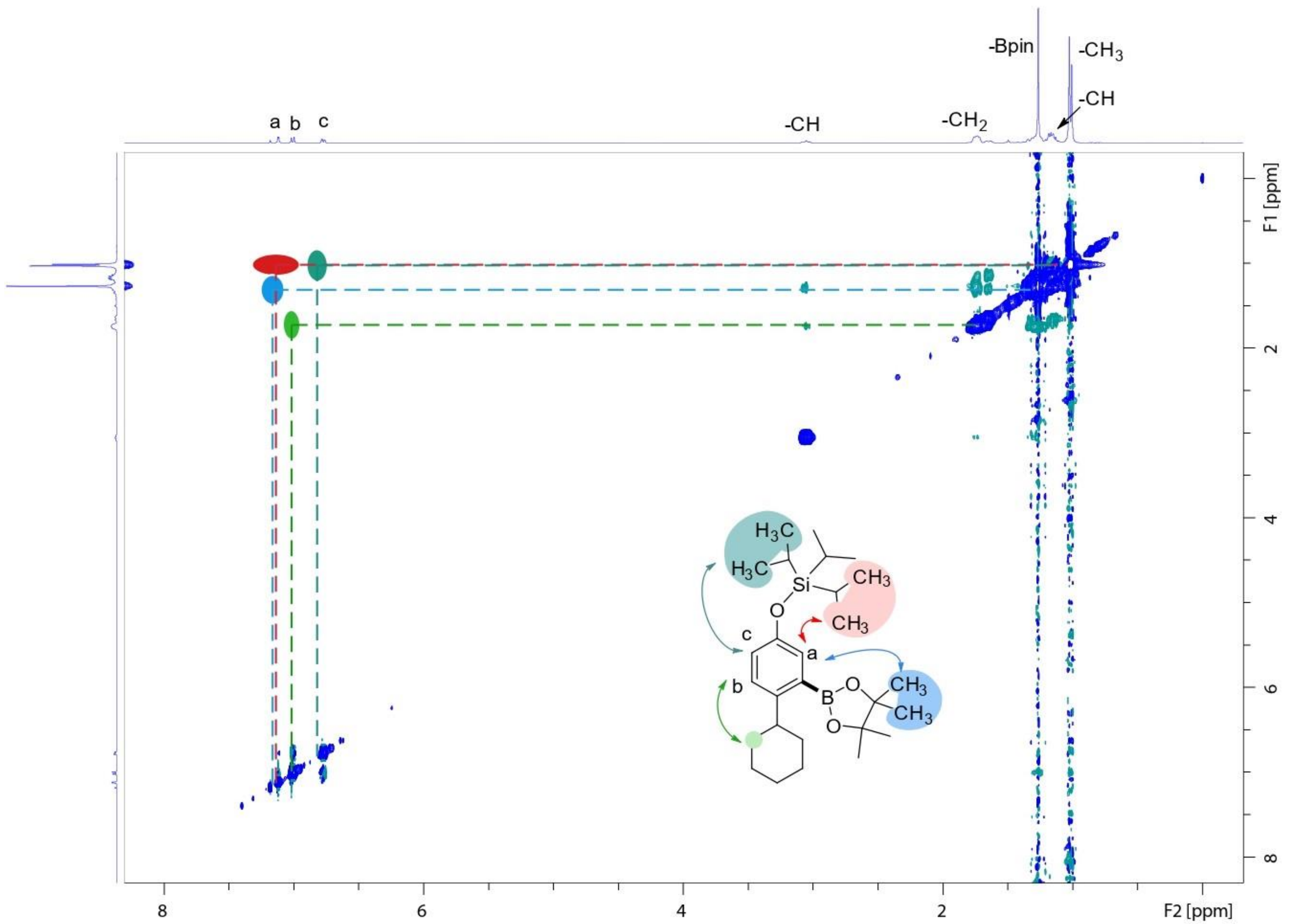

NOESY-NMR spectra of **7r** (25 ^{o}C, 100 MHz, $CDCl_3$)

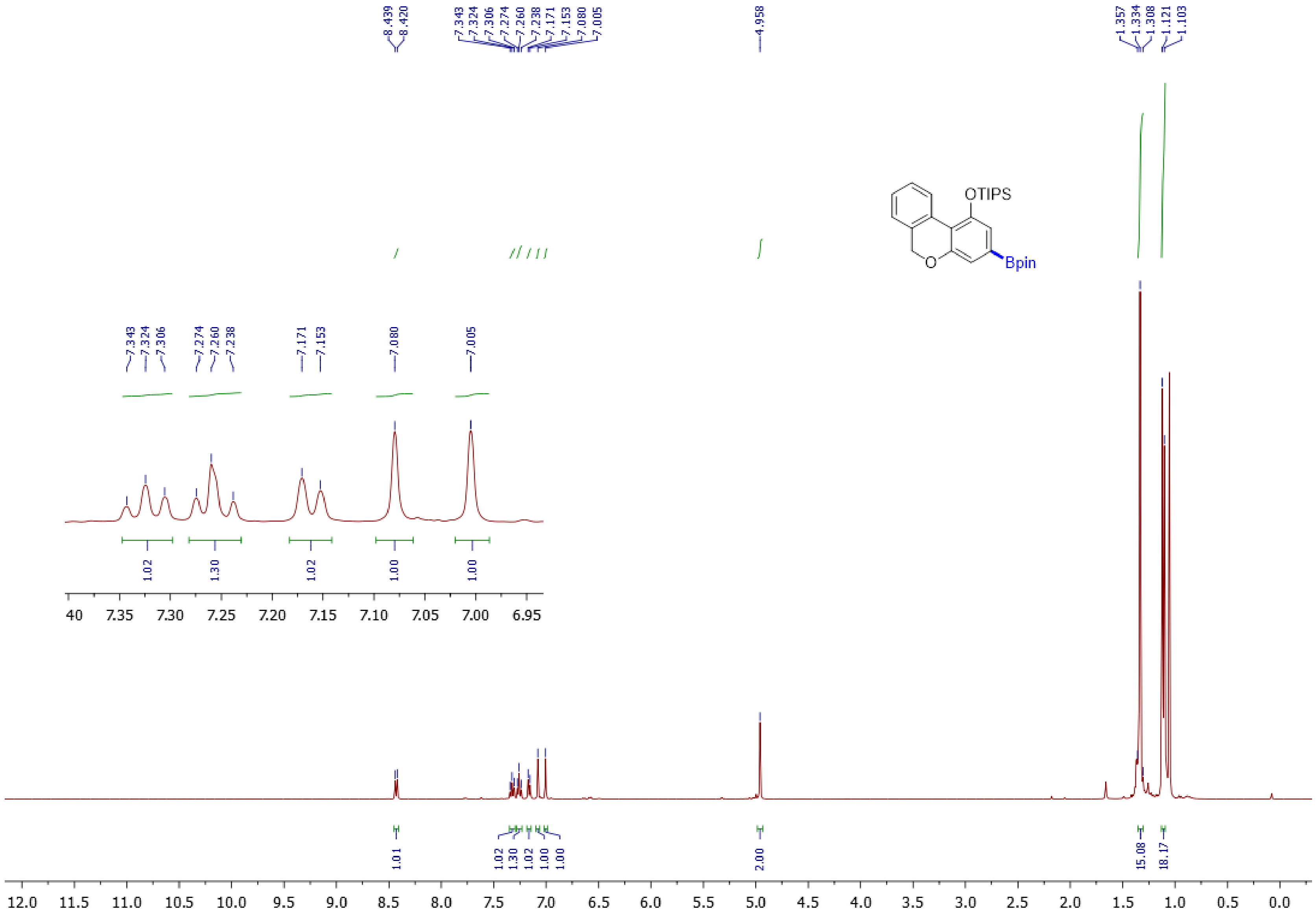

^{1}H-NMR spectra of **11a** (25 °C, 400 MHz, $CDCl_3$)

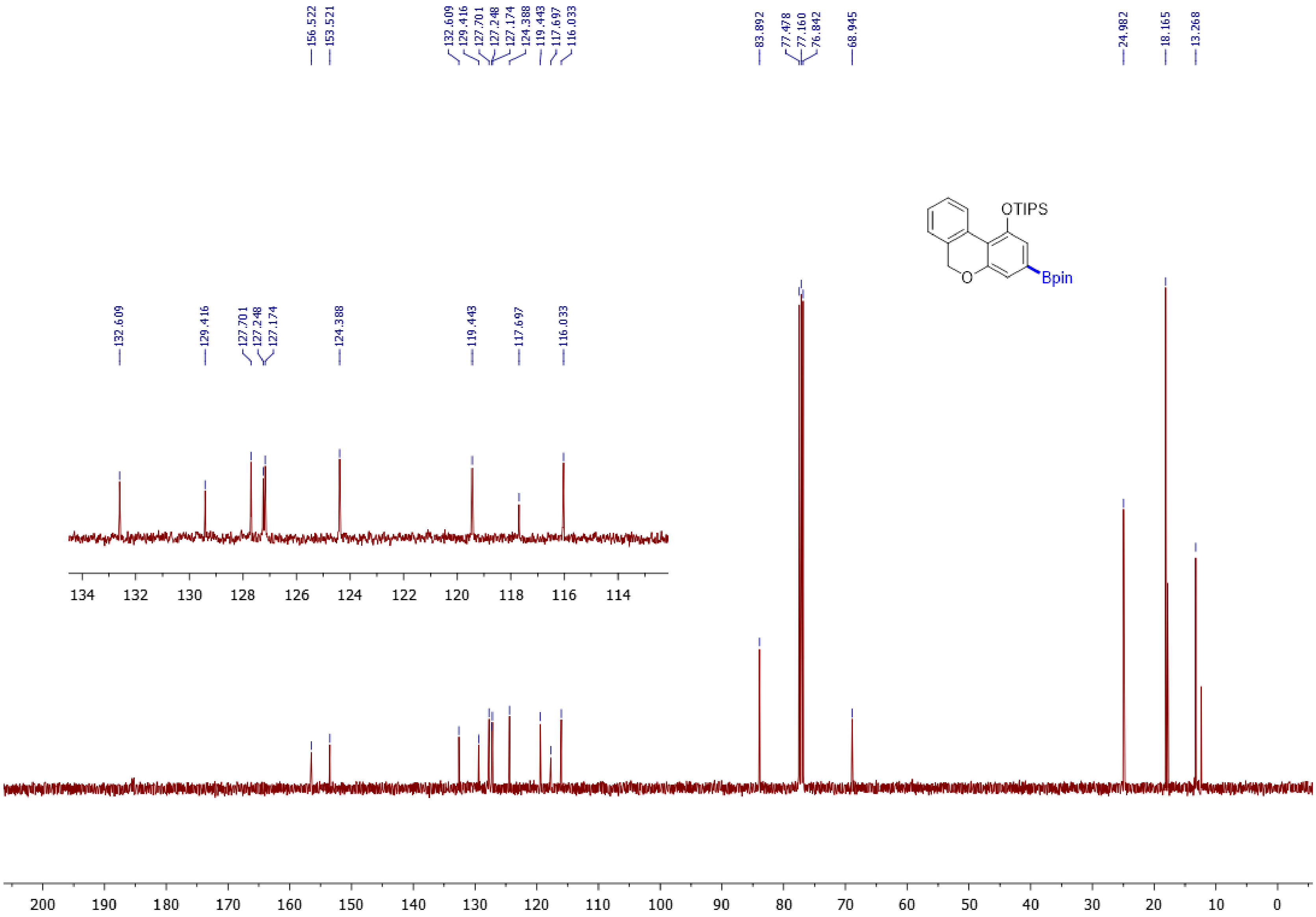

^{13}C-NMR spectra of **11a** (25 °C, 100 MHz, $CDCl_3$)

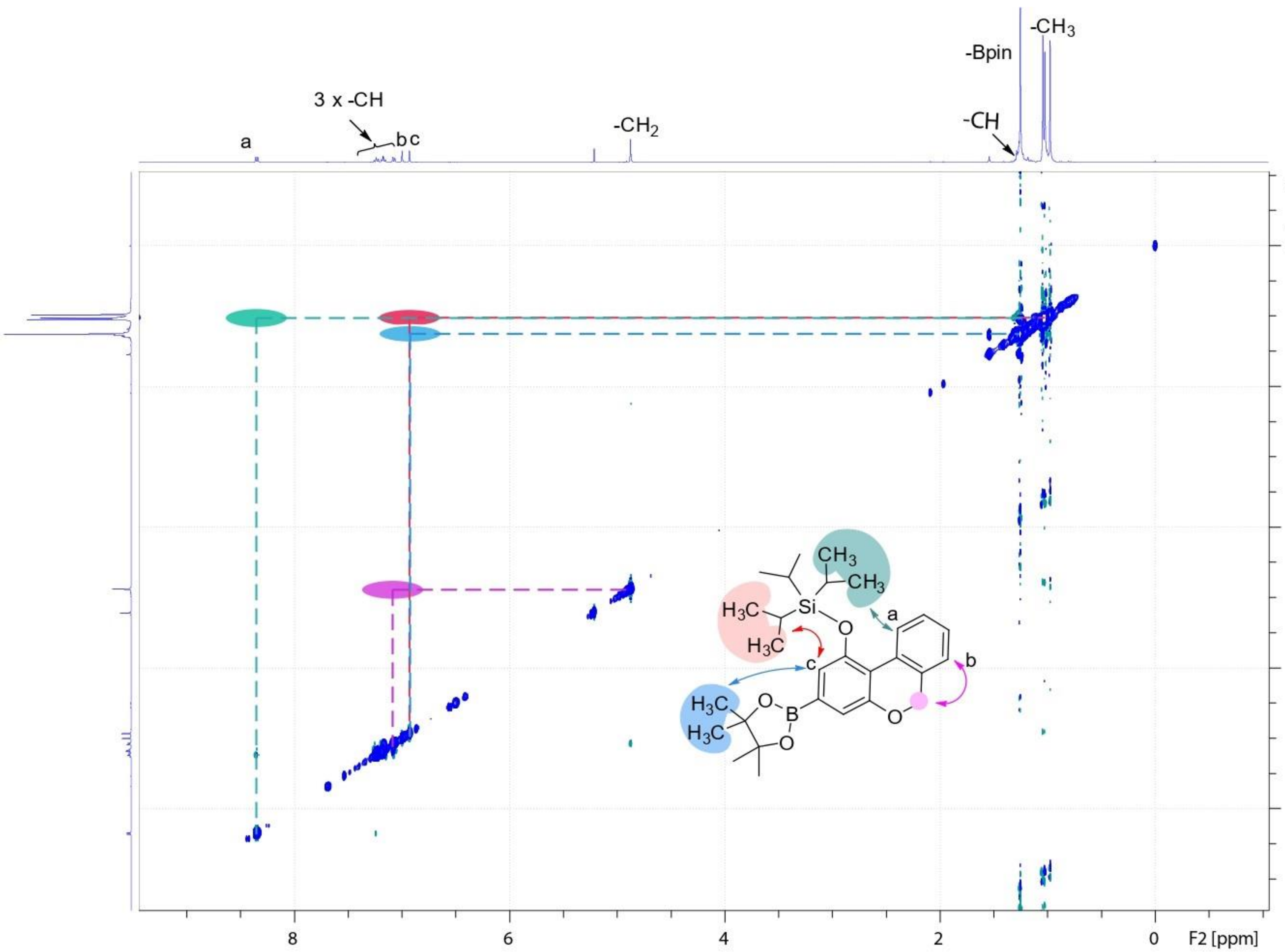

NOESY-NMR spectra of **11a** (25 °C, 100 MHz, $CDCl_3$)

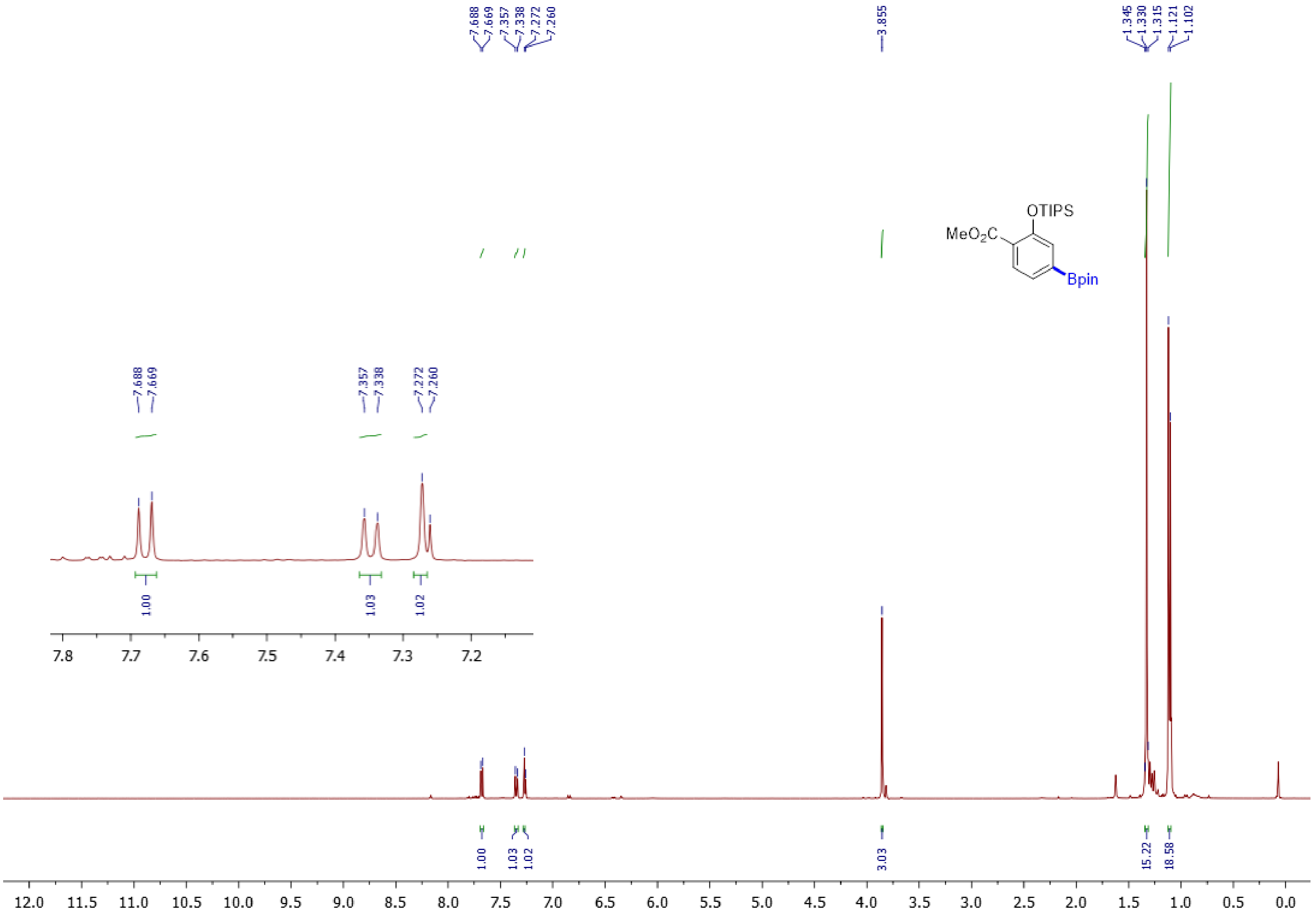

^{1}H-NMR spectra of **11b** (25 °C, 400 MHz, $CDCl_3$)

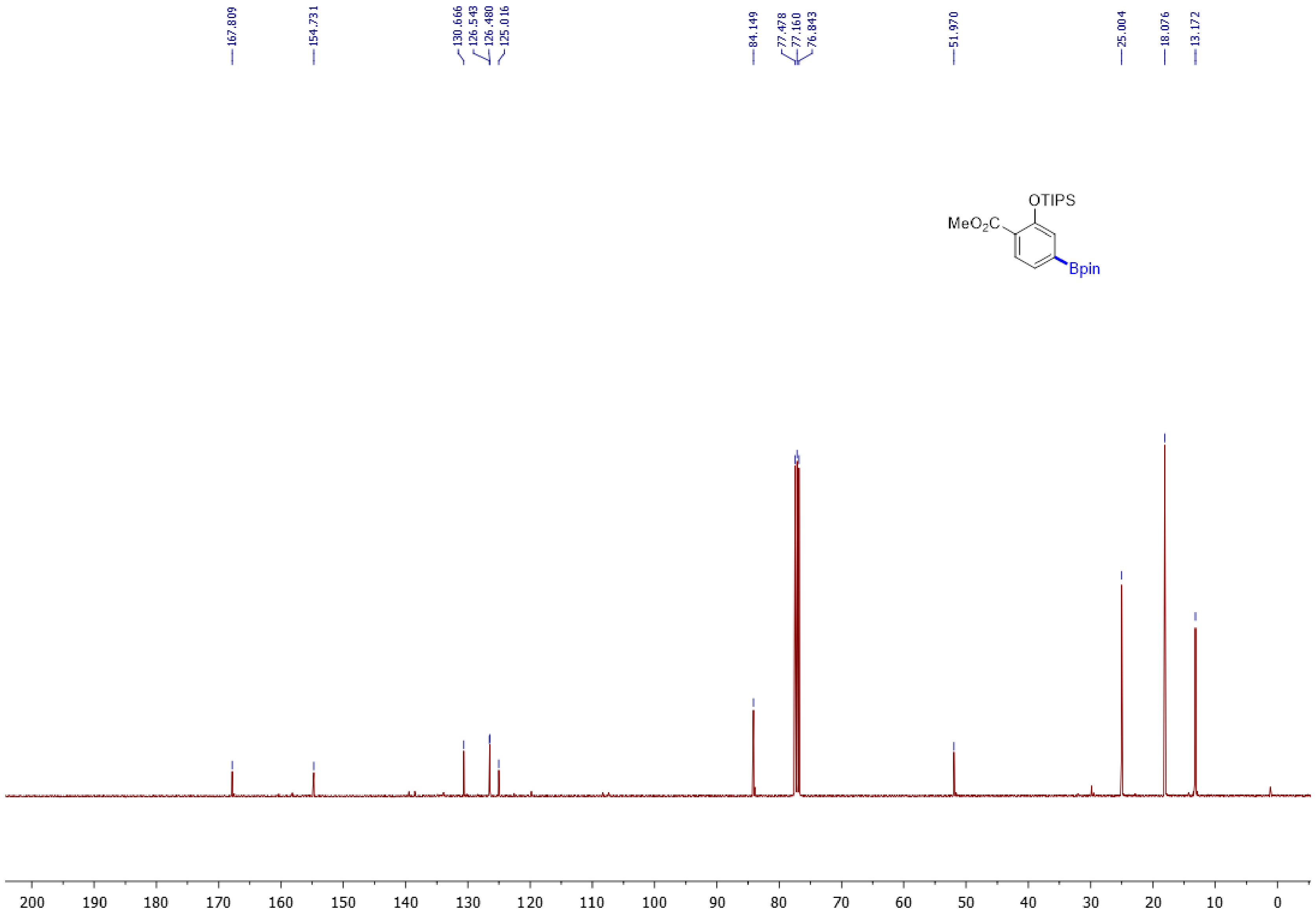

^{13}C-NMR spectra of **11b** (25 ^{o}C, 100 MHz, $CDCl_3$)

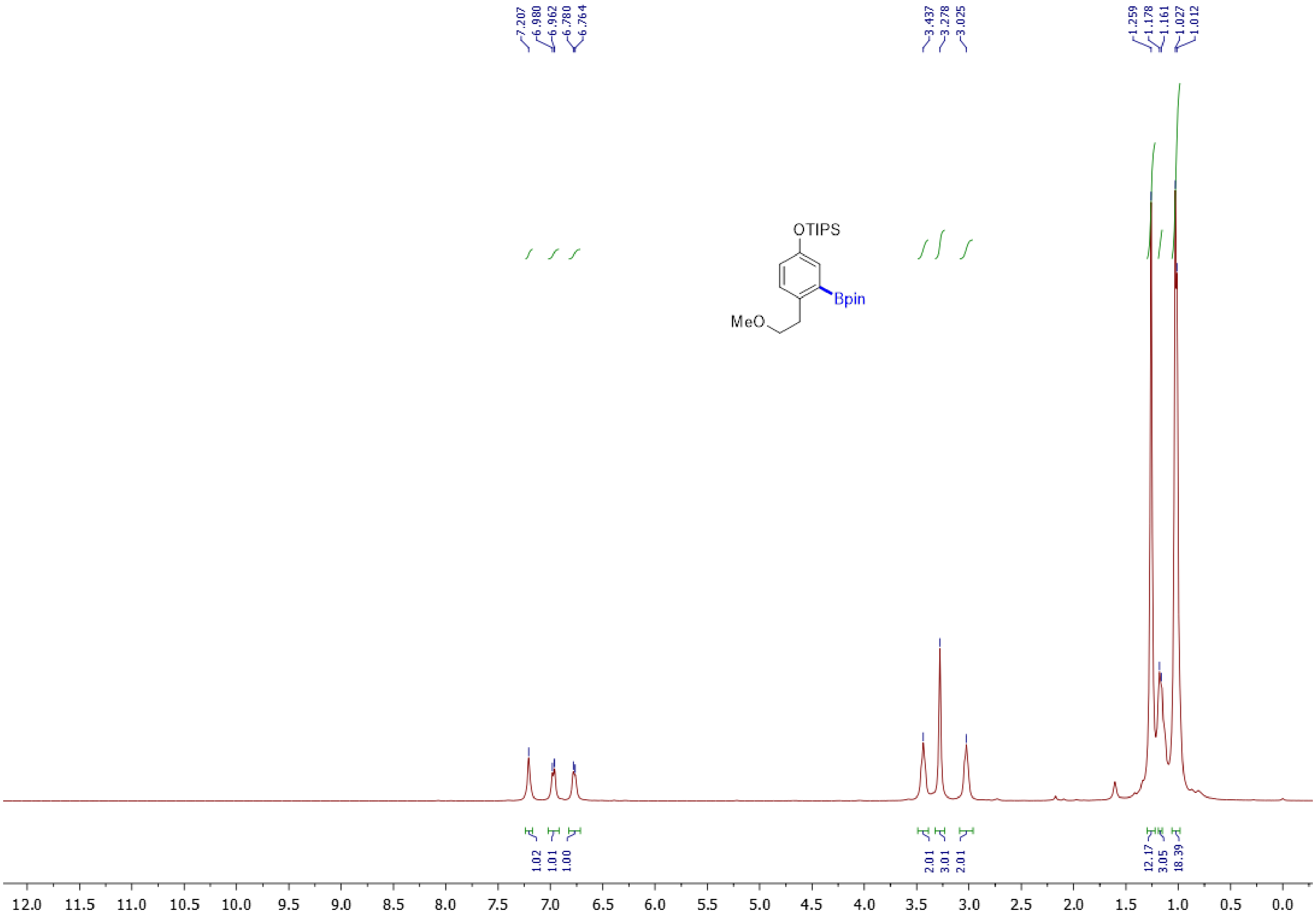

^{1}H-NMR spectra of **11c** (25 °C, 400 MHz, $CDCl_3$)

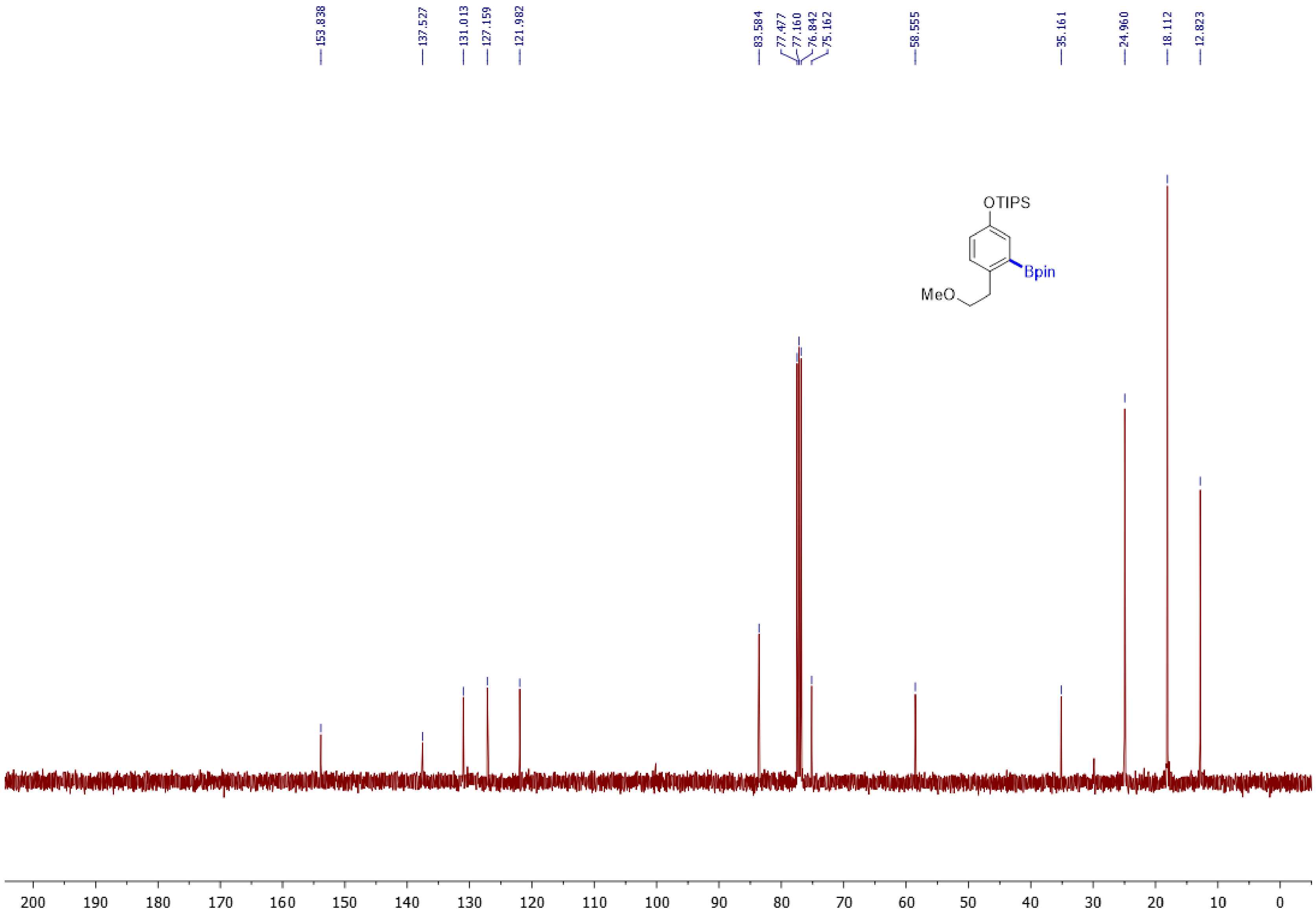

^{13}C-NMR spectra of **11c** (25 ^{o}C, 100 MHz, $CDCl_3$)

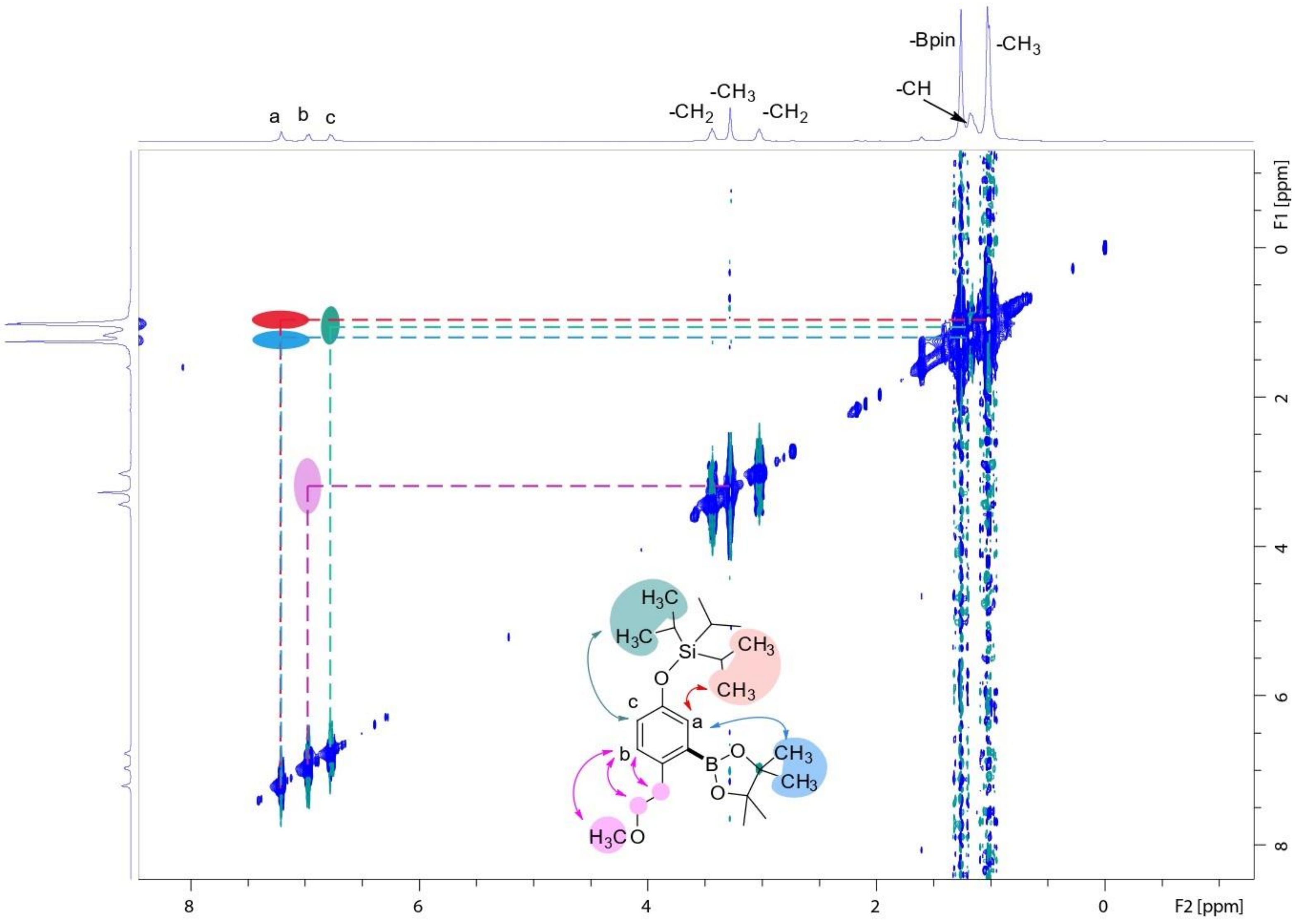

NOESY-NMR spectra of **11c** (25 ^{o}C, 100 MHz, $CDCl_3$)

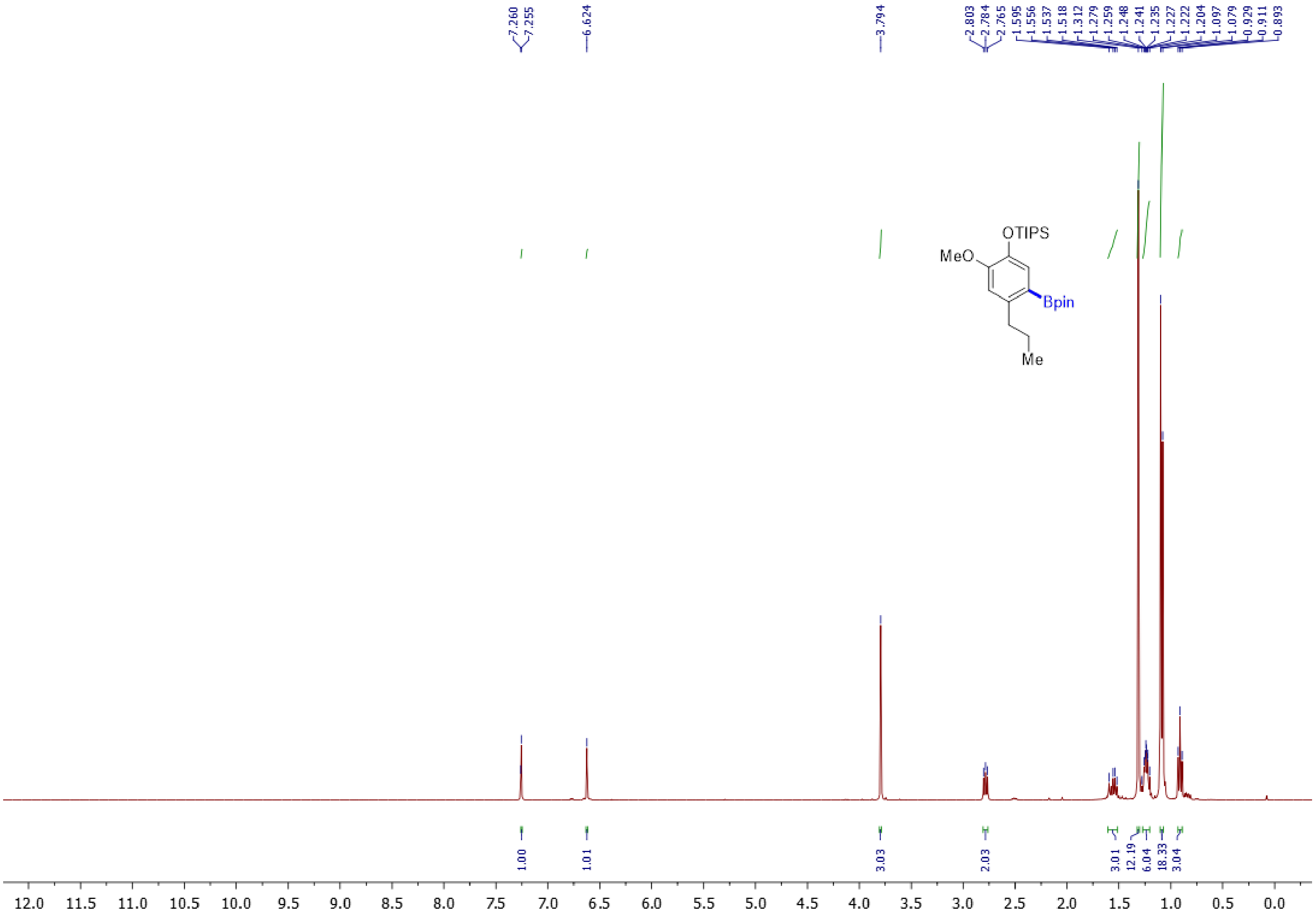

^{1}H-NMR spectra of **11d** (25 °C, 400 MHz, $CDCl_3$)

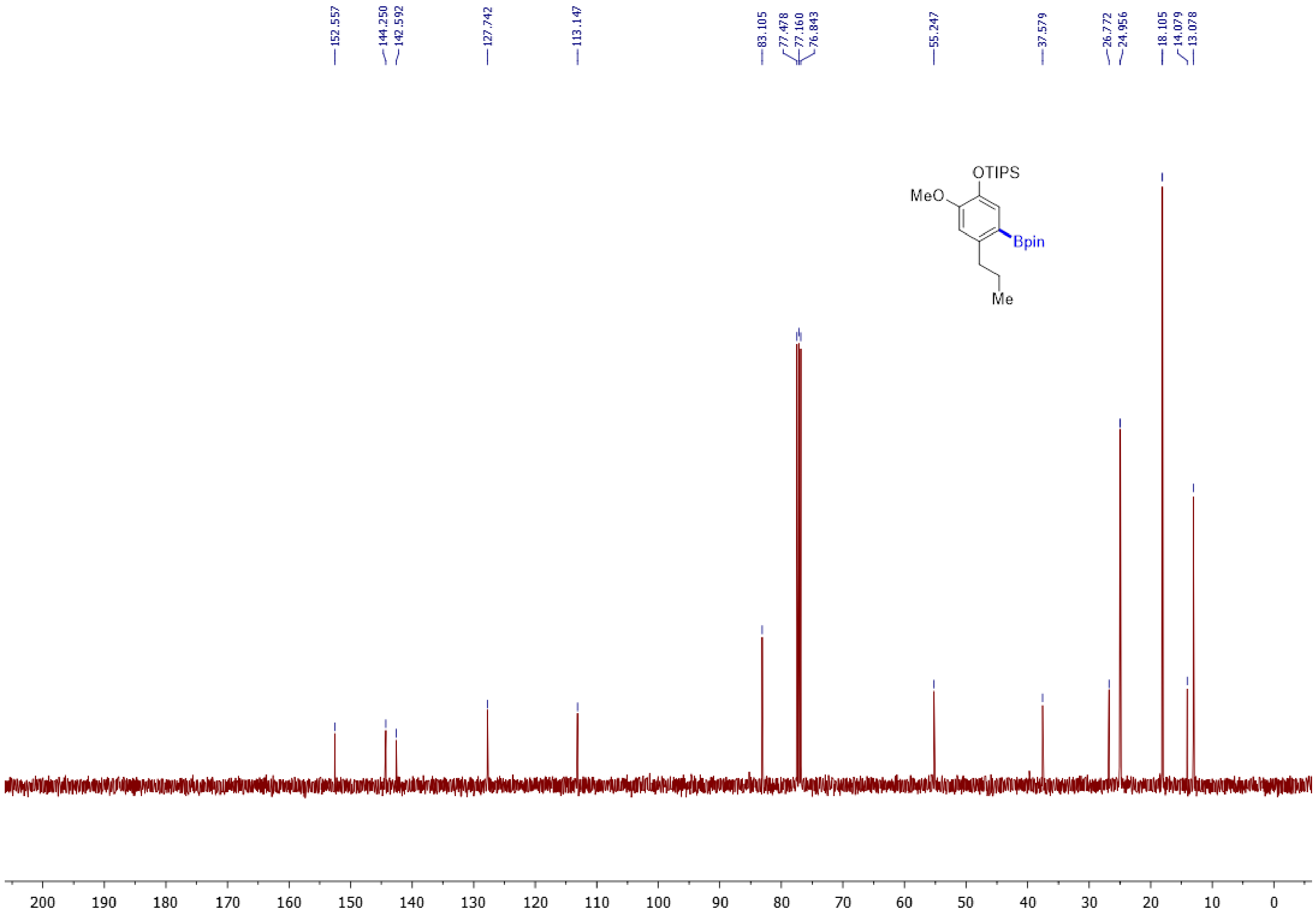

^{13}C-NMR spectra of **11d** (25 ^{o}C, 100 MHz, $CDCl_3$)

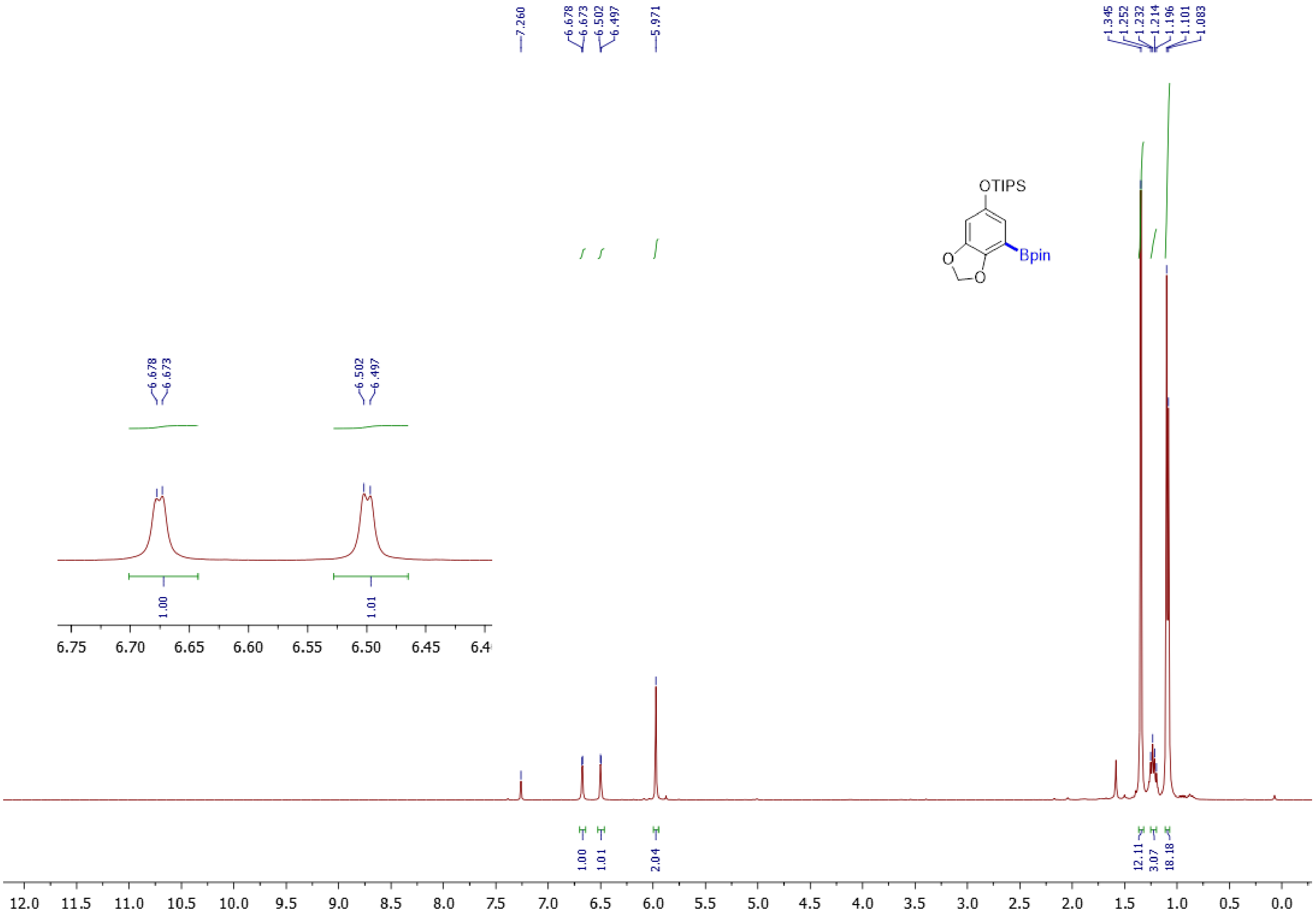

^{1}H-NMR spectra of **11e** (25 °C, 400 MHz, $CDCl_3$)

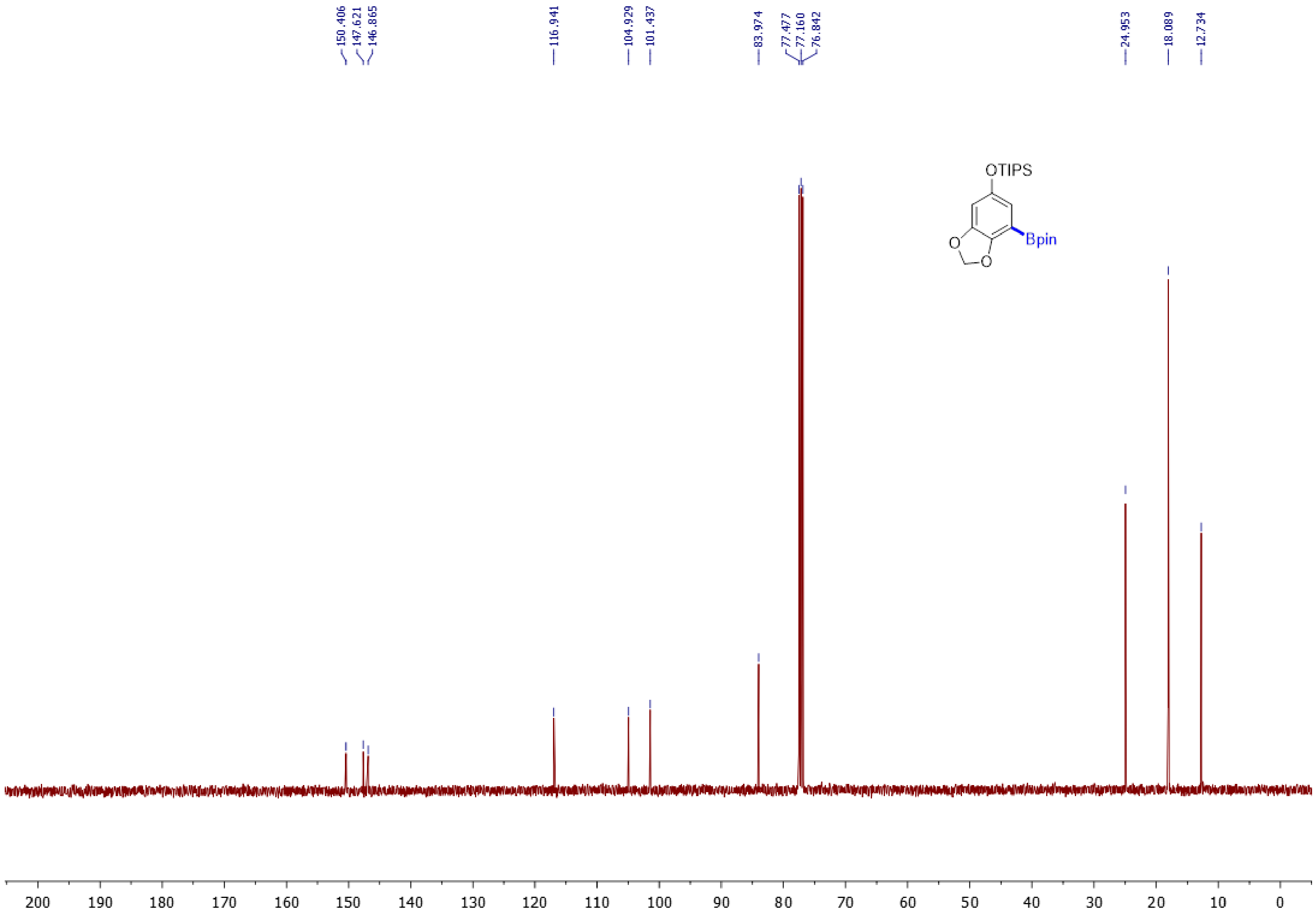

^{13}C-NMR spectra of **11e** (25 °C, 100 MHz, $CDCl_3$)

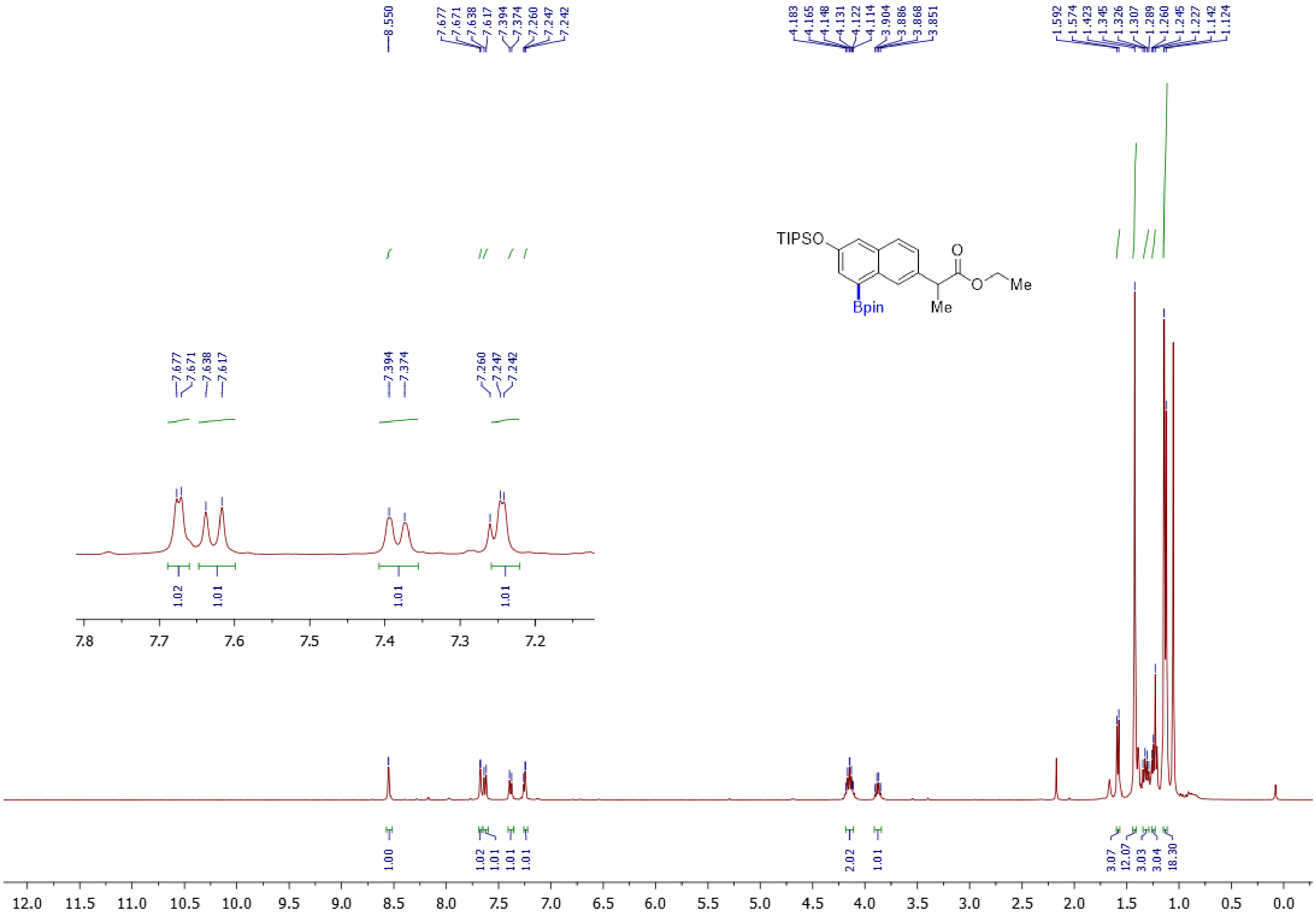

^{1}H-NMR spectra of **11f** (25 °C, 400 MHz, $CDCl_3$)

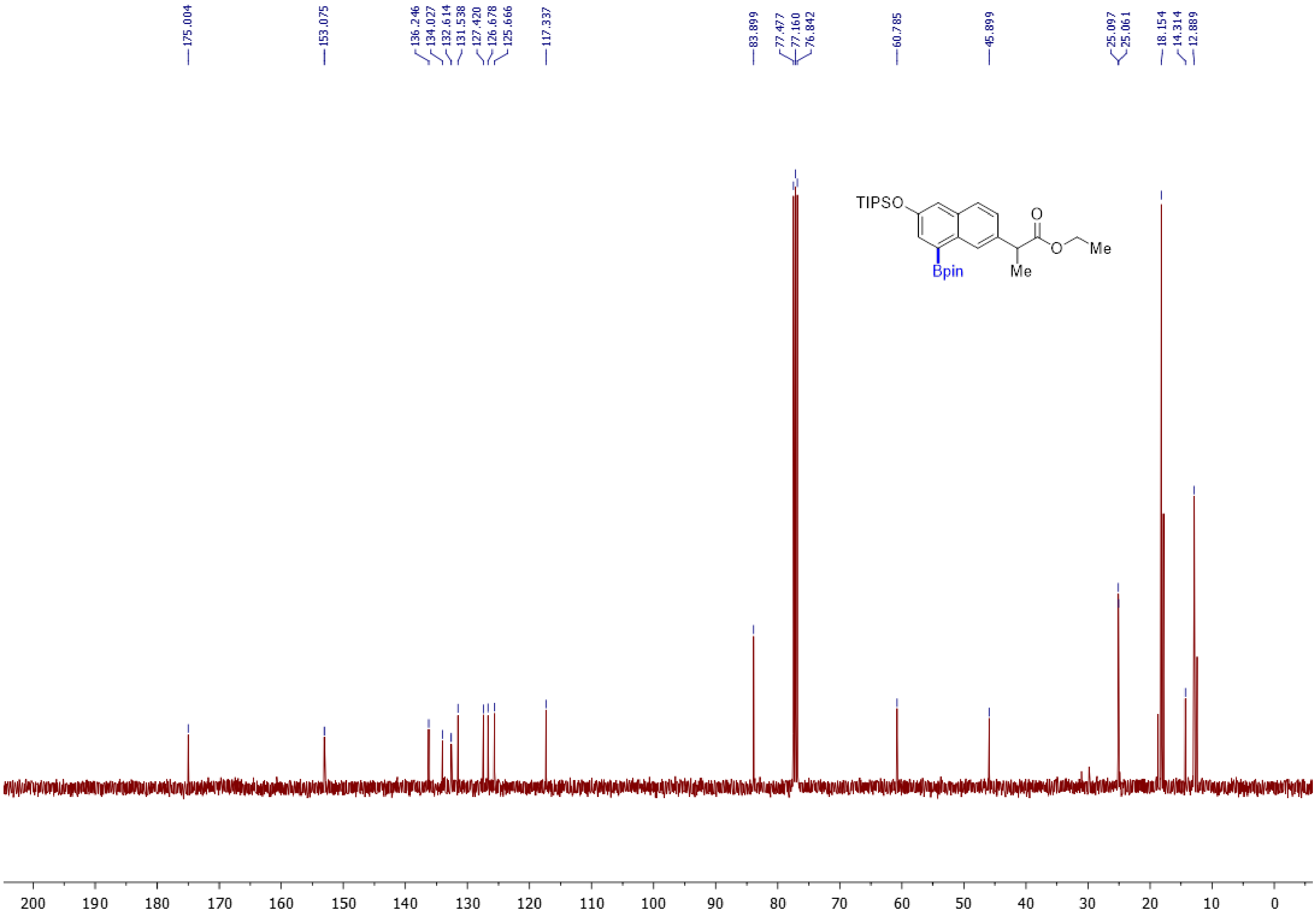

^{13}C-NMR spectra of **11f** (25 ^{o}C, 100 MHz, $CDCl_3$)

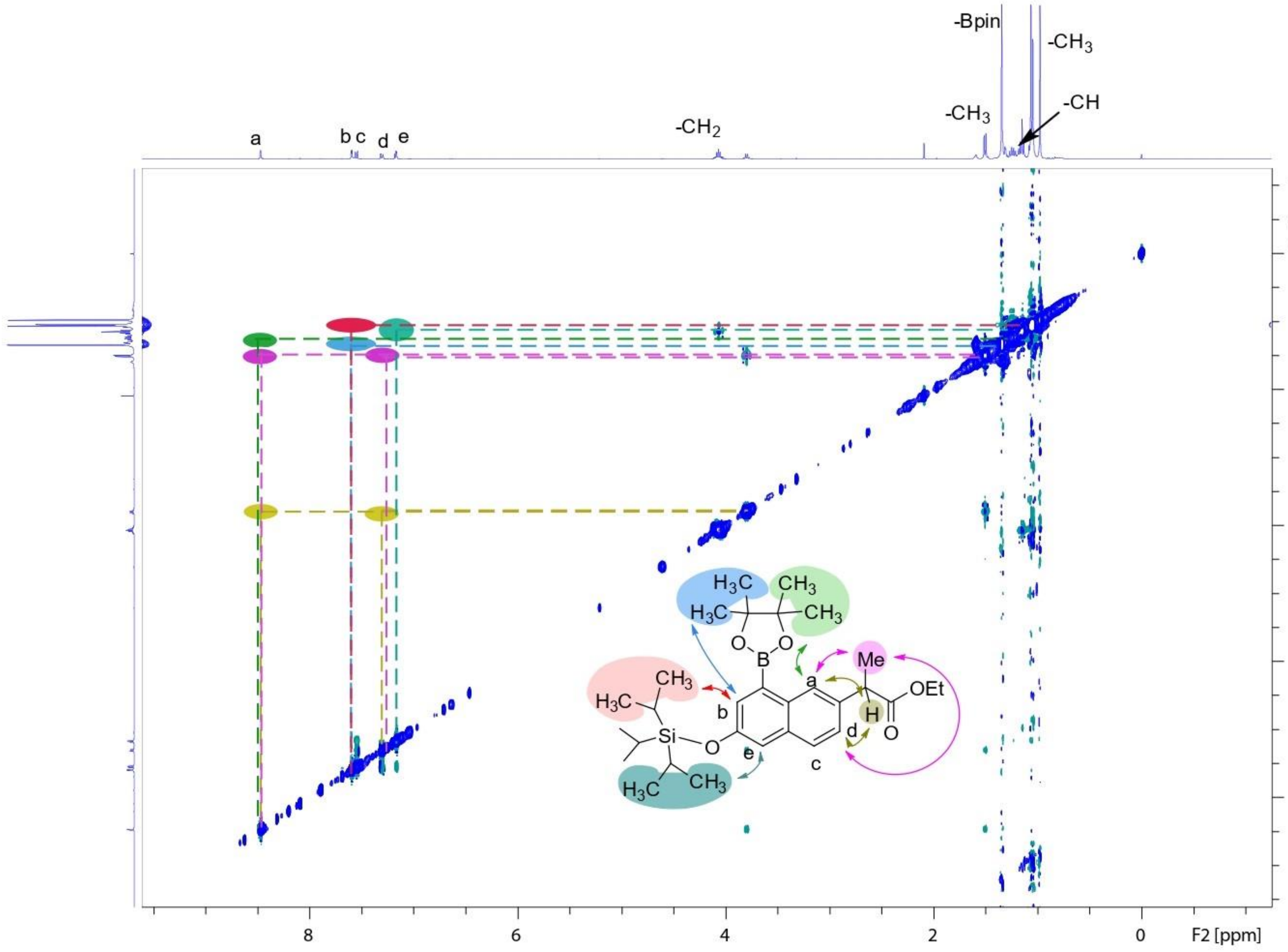

NOESY-NMR spectra of **11f** (25 °C, 100 MHz, $CDCl_3$)

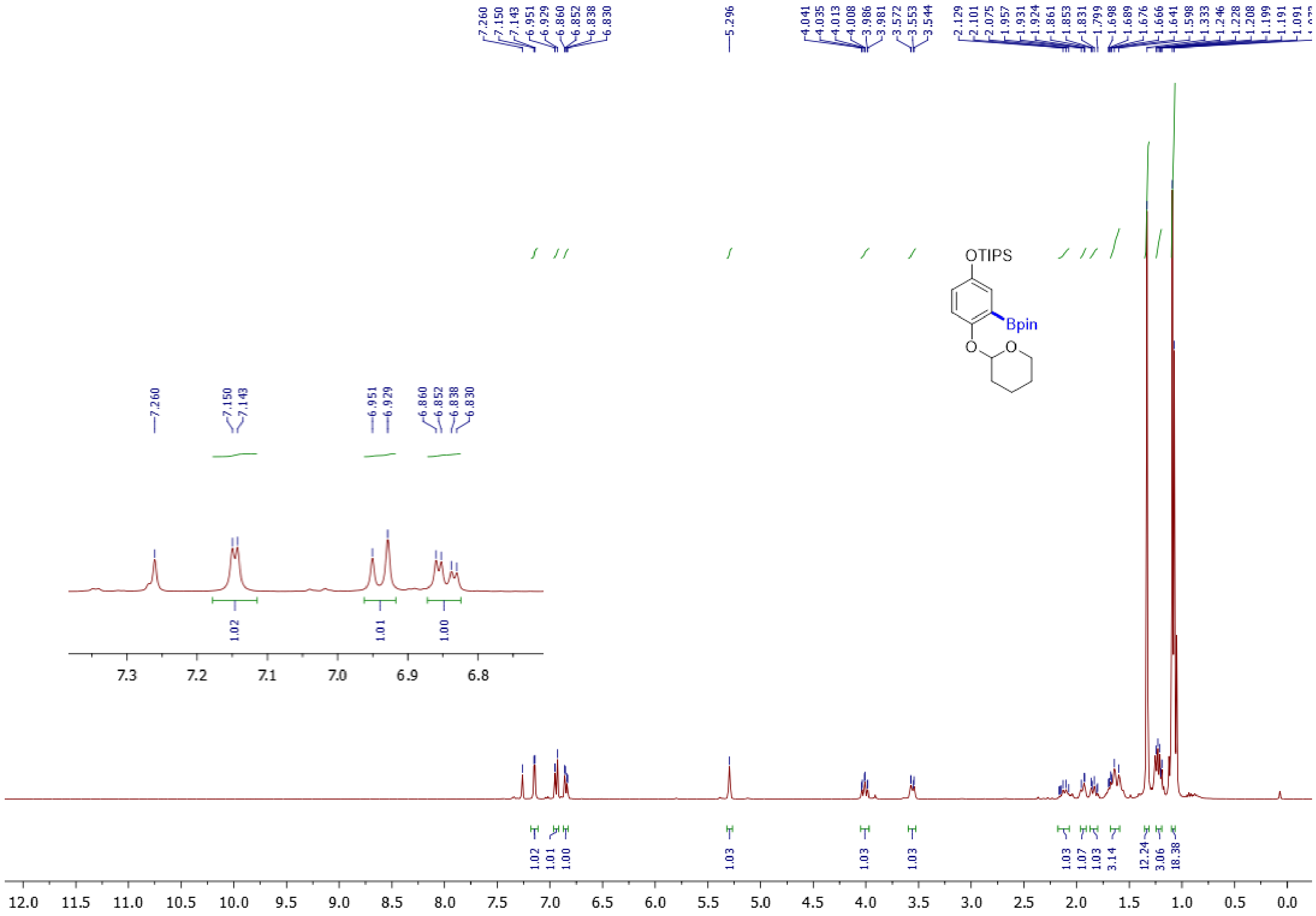

^{1}H-NMR spectra of **11g** (25 °C, 400 MHz, $CDCl_3$)

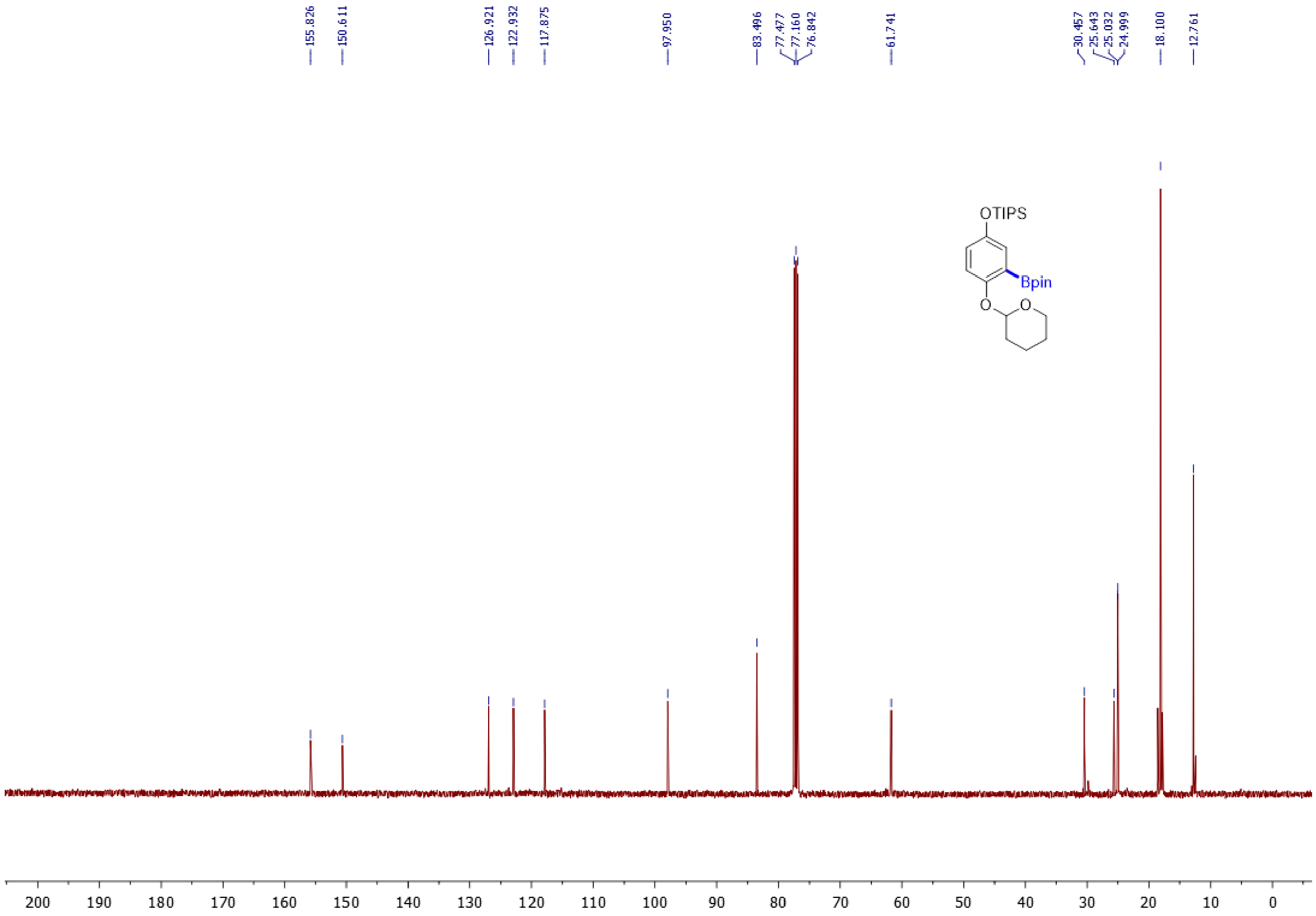

^{13}C-NMR spectra of **11g** (25 ^{o}C, 100 MHz, $CDCl_3$)

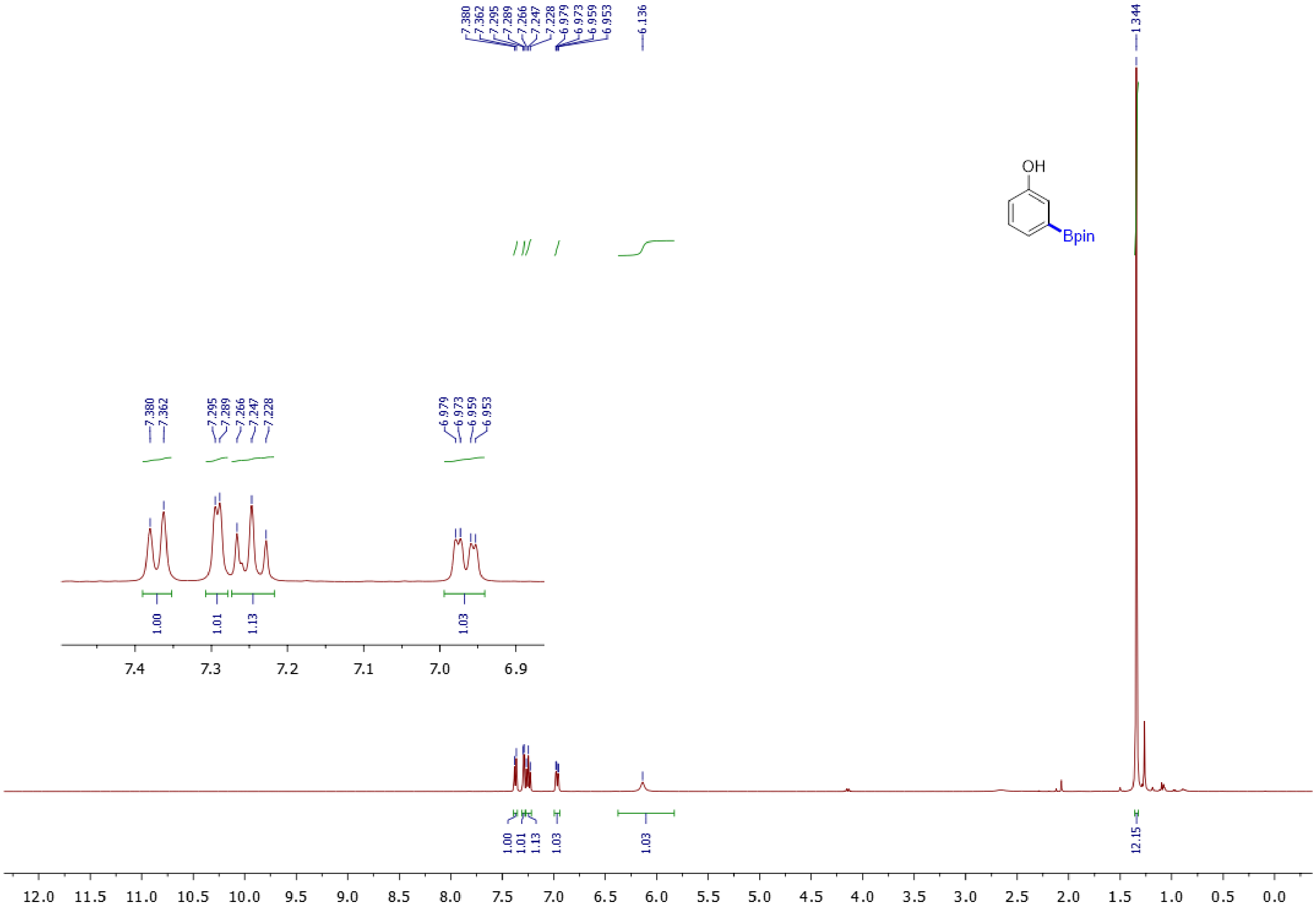

^{1}H-NMR spectra of **12** (25 °C, 400 MHz, $CDCl_3$)

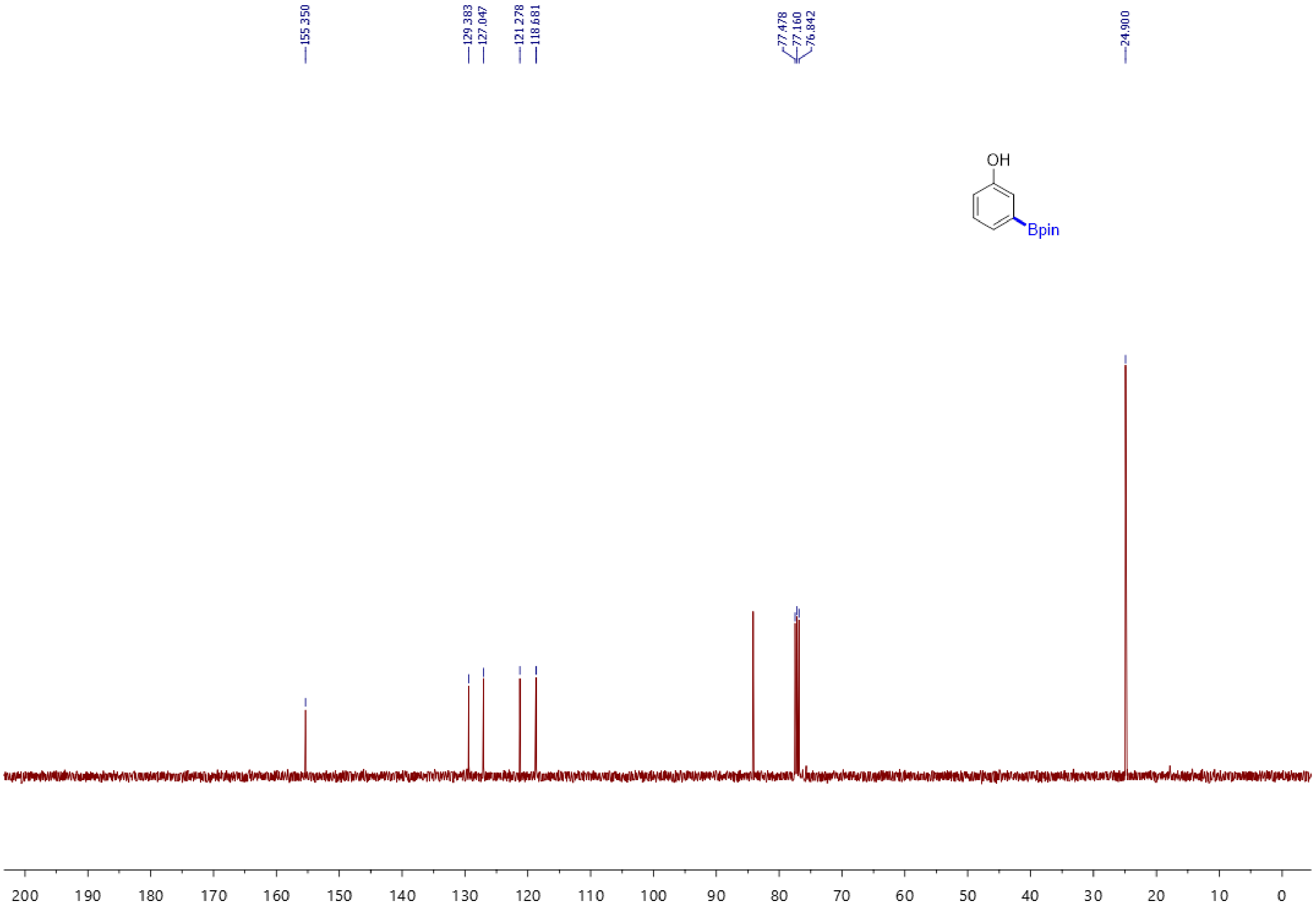

^{13}C-NMR spectra of **12** (25 °C, 100 MHz, $CDCl_3$)

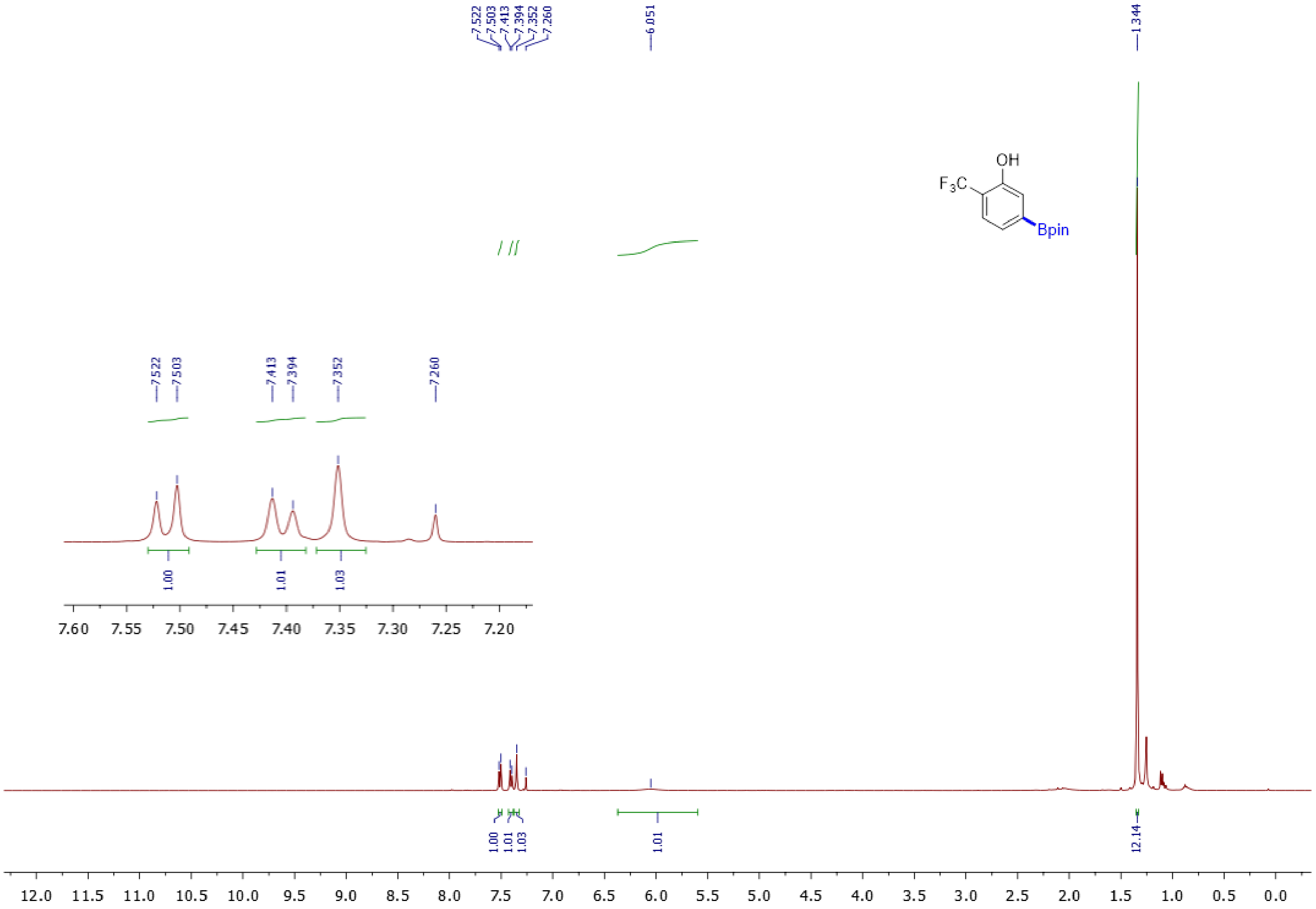

^{1}H-NMR spectra of **13** (25 ^{o}C, 400 MHz, $CDCl_3$)

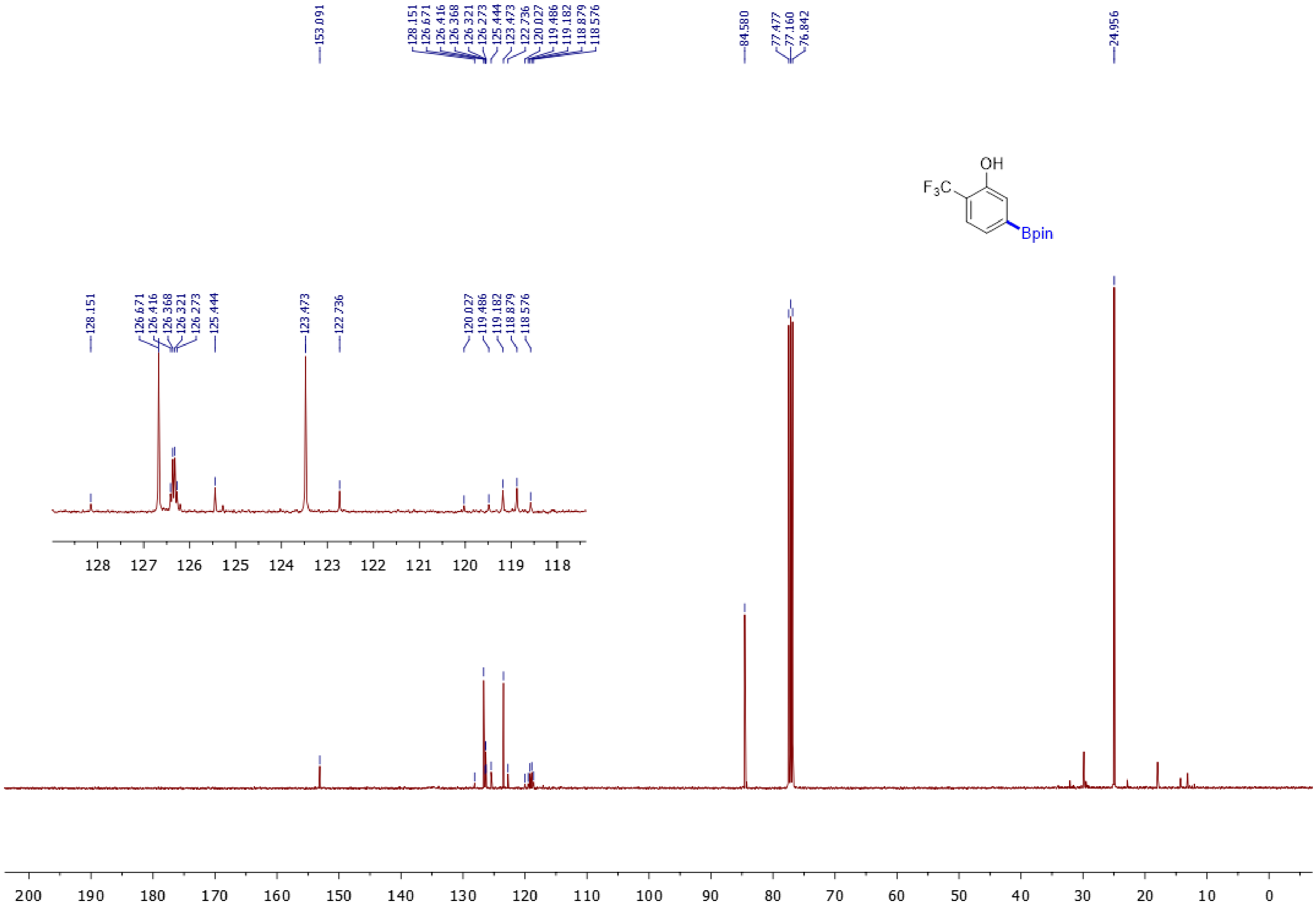

^{13}C-NMR spectra of **13** (25 °C, 100 MHz, $CDCl_3$)

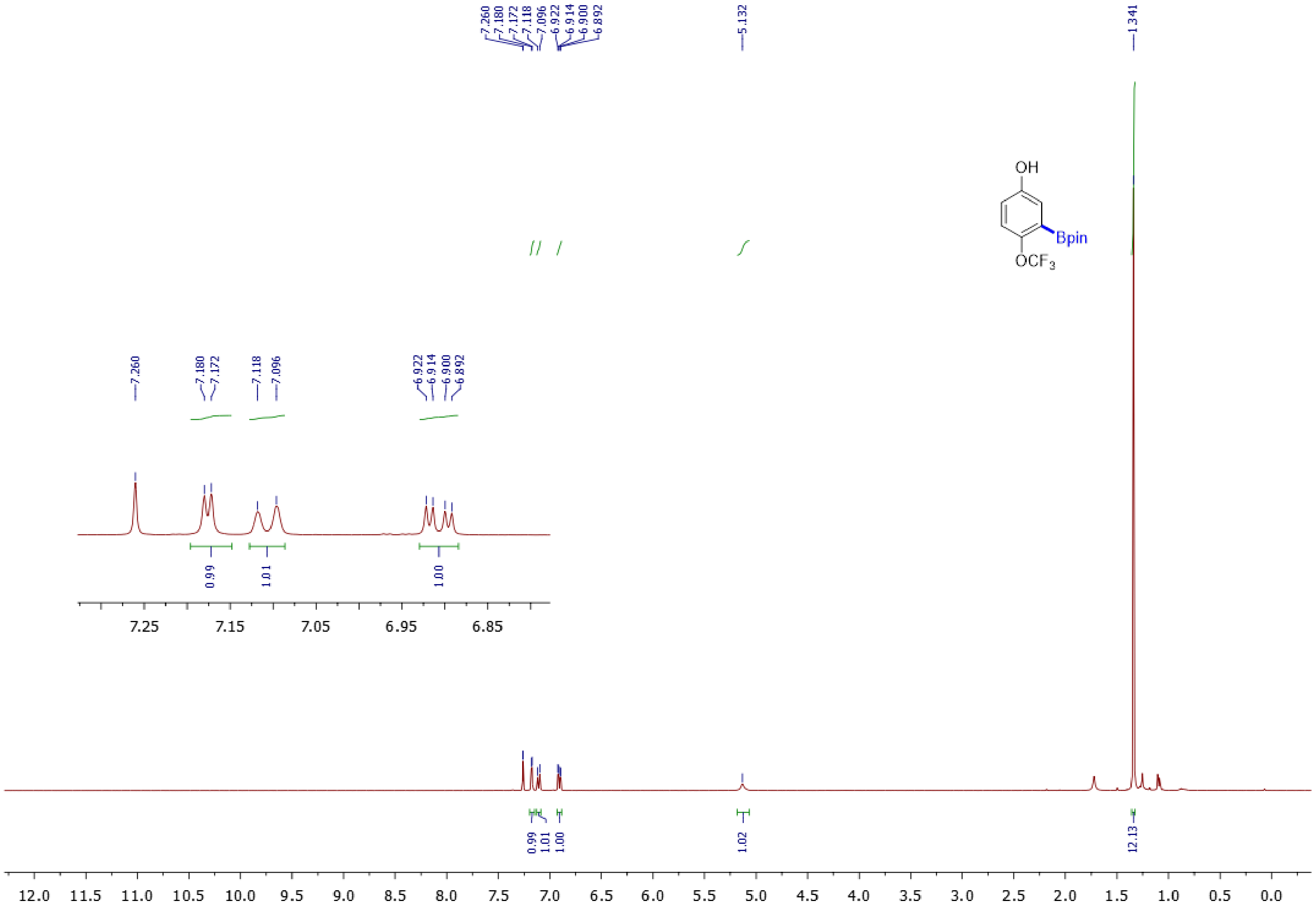

^{1}H-NMR spectra of **14** (25 ^{o}C, 400 MHz, $CDCl_3$)

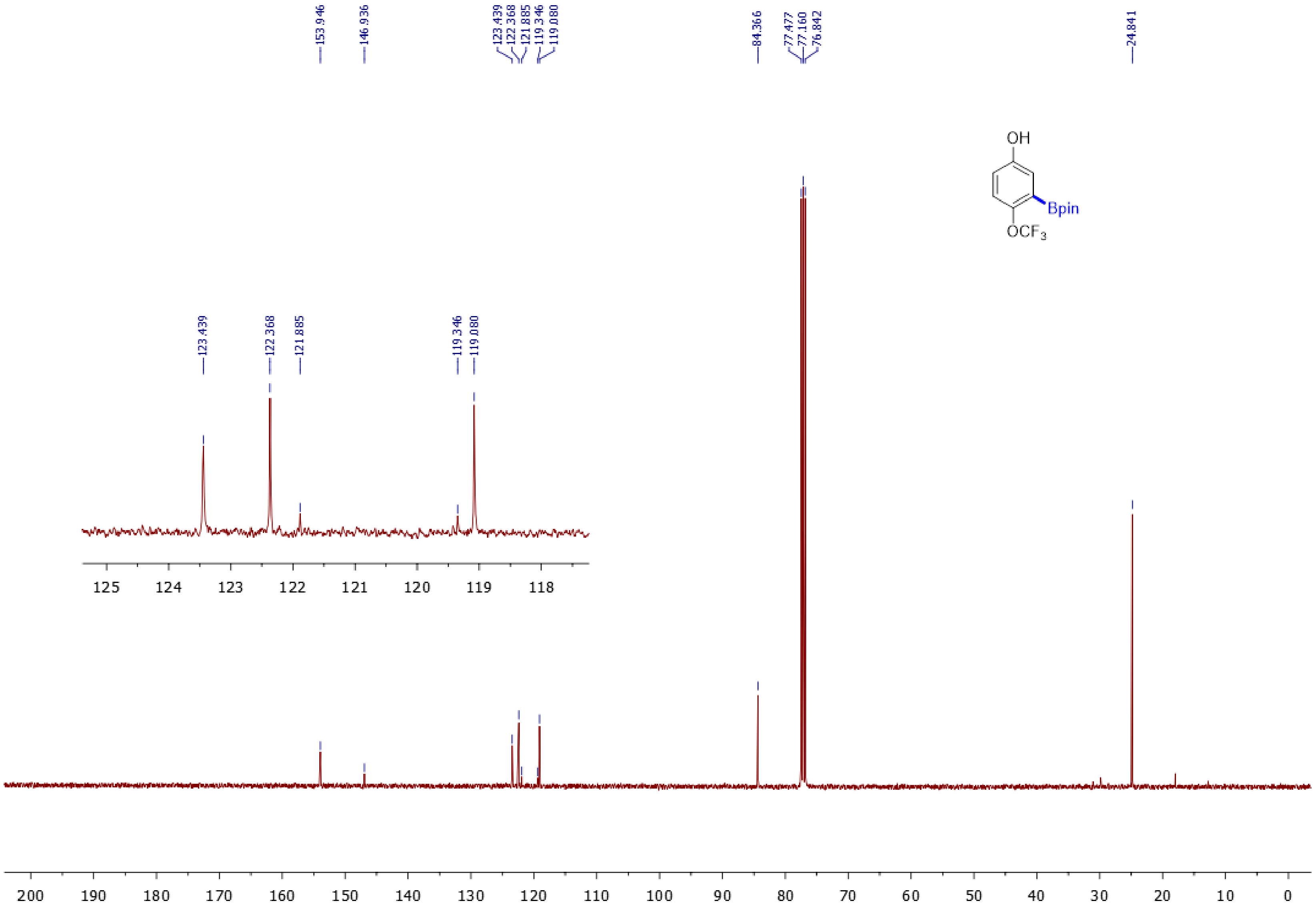

^{13}C-NMR spectra of **14** (25 °C, 100 MHz, $CDCl_3$)

References:

1. Velappan, A. B. et. al. 2-Methoxyphenyl isocyanate: a chemoselective multitasking reagent for an amine protection/deprotection sequence. *Org. Chem. Front*., **6**, 2360-2364 (2019).

2. Aukland, M.H., Šiaučiulis, M., West, A. et al. Metal-free photoredox-catalysed formal C–H/C–H coupling of arenes enabled by interrupted Pummerer activation. *Nat. Catal*., **3**, 163–169 (2020).

3. Procopio, A. et. al. An eco-sustainable erbium(iii)-catalyzed method for formation/cleavage of O-tert-butoxy carbonates. *Green Chem.*, **13**, 436-443 (2011).

4. Bartoli, G. et. al. A New, Mild, General and Efficient Route to Aryl Ethyl Carbonates in Solvent-Free Conditions Promoted by Magnesium Perchlorate. *Eur. J. Org. Chem*., **2006**, 4429-4434 (2006).

5. Lo, H. J., Lin, C. Y., Tseng, M. C., Chein, R. J. Lithiation of a Silyl Ether: Formation of an ortho-Fries Hydroxyketone. *Angew. Chem. Int. Ed*., **53**, 9026-9029 (2014).

6. Olivito, F., Costanzo, P., Di Gioia, M. L., Nardi, M., Oliverio M. & Procopio, A. Efficient synthesis of organic thioacetates in water. *Org. Biomol. Chem*., **16**, 7753-7759 (2018).

7. Alyssa F.J., van den Boom, Subramaniam, M. & Zuilhof, H. Sulfur-Phenolate Exchange As a Fluorine-Free Approach to S(VI) Exchange Chemistry on Sulfonyl Moieties. *Org. Lett.*, **24**, 8621–8626 (2022).

8. DeLucia, N. A., Das, N. & Vannucci, A. K. Mild synthesis of silyl ethers via potassium carbonate catalyzed reactions between alcohols and hydrosilanes. *Org. Biomol. Chem*., **16**, 3415-3418 (2018).

9. Seyferth, D., Annarelli, D. C., Shannon, M. L., Escudie, J. & Duncan, D. P. Hexamethylsilirane: II. Ring-opening reactions with some simple reagents. *Journal of Organometallic Chemistry*, **225**, 177-192 (1982).

10. Mehta, M. & Goicoechea, J. M. Nitrenium Salts in Lewis Acid Catalysis. *Angew. Chem. Int. Ed*., **59**, 2715-2719 (2019).

11. Allouche, E. M. D., Al-Saleha, A., & Charette, A. B. Iron-catalyzed synthesis of cyclopropanes by in situ generation and decomposition of electronically diversified diazo compounds. *Chem. Commun.*, **54**, 13256-13259 (2018).

12. Bartoli, G. et. al. Alcohols and Di-tert-butyl Dicarbonate: How the Nature of the Lewis Acid Catalyst May Address the Reaction to the Synthesis of tert-Butyl Ethers. J. Org. Chem. 71, 9580–9588 (2006).

13. Marzi, E. & Schlosser, M. The site-selective functionalization of halogen-bearing phenols: an exercise in diversity-oriented organometallic synthesis. *Tetrahedron*, **61**, 3393-3401 (2005).

14. Lavery, C. B., McDonald, R. & Stradiotto, M. Efficient palladium-catalyzed synthesis of substituted indoles employing a new (silanyloxyphenyl)phosphine ligand. *Chem. Commun*., **48**, 7277-7279 (2012).

15. Csekei, M., Novak, Z. & Kotschy, A. Development of a one-pot sequential Sonogashira coupling for the synthesis of benzofurans. *Tetrahedron*, **64**, 8992-8996 (2008).

16. Komeyama, K., Yamahata, Y. & Osaka, I. Nickel and Nucleophilic Cobalt-Catalyzed Trideuteriomethylation of Aryl Halides Using Trideuteriomethyl p-Toluenesulfonate. *Org. Lett*., **20**, 4375-4378 (2018).

17. Tao, L., Guo, X., Li, J., Li, R., Lin, Z. & Zhao, W. Rhodium-Catalyzed Deoxygenation and Borylation of Ketones: A Combined Experimental and Theoretical Investigation. *J. Am. Chem. Soc*., **142**, 18118-18127 (2020).

10. Liu, X., Jia, J. & Rueping, M. Nickel-Catalyzed C–O Bond-Cleaving Alkylation of Esters: Direct Replacement of the Ester Moiety by Functionalized Alkyl Chains. *ACS Catal*., **7**, 4491-4496 (2017).

19. Yu, J., Li, C. J. & Zeng, H. Dearomatization-Rearomatization Strategy for ortho -Selective Alkylation of Phenols with Primary Alcohols. *Angew. Chem. Int. Ed*., **60**, 4043-4048 (2020).

20. Saga, Y. et. al. Catalytic Asymmetric Synthesis of R207910. *J. Am. Chem. Soc.*, **132**, 7905-7907 (2010).

21. Varjosaari, S. E. et. al. Stereoelectronics of silyloxybenzoic acids. *Tetrahedron Letters*, **56**, 642-645 (2015).

22. Zernickel, A. et. al. Bedford-Type Palladacycle-Catalyzed Miyaura Borylation of Aryl Halides with Tetrahydroxydiboron in Water. *J. Org. Chem*., **83**, 1842-1851 (2018).

23. Shaikh, A. k. & Varvounis, G. Novel Synthesis of 3-Substituted 2,3-Dihydrobenzofurans via ortho-Quinone Methide Intermediates Generated in Situ. *Org. Lett.*, **16**, 1478-1481 (2014).

24. Tezuka, N. et. al. Direct Hydroxylation and Amination of Arenes via Deprotonative Cupration. *J. Am. Chem. Soc.*, **138**, 9166-9171 (2016).

25. Prasad, K. R., Suresh, P., Ravikumar, B., Reddy, N. V. & Reddy, K. R. Synthesis of functionalized carbamates and quinones via sequential oxidation of salicylaldehydes using TBHP as the oxidant. *Tetrahedron Letters*, **55**, 6307-6310 (2015).
26. Davies, J. S., Higginbotham, C. L., Tremeer, E. J., Brown, C. & Treadgold, R. C. Protection of hydroxy groups by silylation: use in peptide synthesis and as lipophilicity modifiers for peptides. *J. Chem. Soc., Perkin Trans. 1*, 3043-3048 (1992).
27. Lee, B. J., DeGlopper, K. S. & Yoon, T. P. Site-Selective Alkoxylation of Benzylic C−H Bonds by Photoredox Catalysis, *Angew. Chem. Int. Ed*., **59**, 197-202 (2020).
28. Chauvier, C., Godou, T. & Cantat, T. Silylation of O–H bonds by catalytic dehydrogenative and decarboxylative coupling of alcohols with silyl formats. *Chem. Commun.*, **53**, 11697-11700 (2017).
29. Ilangovan, A., Saravanakumar, S., Malayappasamy, S. & Manickam, G. A convenient approach for the deprotection and scavenging of the PMB group using POCl3. *RSC Adv*., **3**, 14814-14828 (2013).
30. Uetake, Y., Niwa, T. & Hosoya, T. Rhodium-Catalyzed ipso-Borylation of Alkylthioarenes via C–S Bond Cleavage. *Org. Lett.,* **18**, 2758-2761 (2016).
31. Firouzabadi, H., Iranpoor, N. & Shaterian, H. R. Effective silylation of carboxylic acids under solvent-free conditions with tert-butyldimethylsilyl chloride (TBDMSCl) and triisopropylsilyl chloride (TIPSCl). *Phosphorus, Sulfur and Silicon and the Related Elements*, **166,** 71-81 (2000).
32. Pilzak, G. S. et. al. The synthesis of 13C6-labeled l-thyronine, 3,5-diiodothyronine, 3,3′,5-triiodothyroacetic acid and 3,3′,5,5′-tetraiodothyroacetic acid. *Tetrahedron*, **76**, 131352 (2020).
33. Correia, A. R., Weyel, X. M. M. & Heckel, A. Four Levels of Wavelength-Selective Uncaging for Oligonucleotides. *Org. Lett.*, **15**, 21, 5500-5503 (2013).
34. Lu, L., Siu, J. C., Lai, Y. & Lin, S. An Electroreductive Approach to Radical Silylation via the Activation of Strong Si–Cl Bond. *J. Am. Chem. Soc.*, **142**, 21272–21278 (2020).
35. Feng, Y., Holte, D., Zoller, J., Umemiya, S., Simke, L. R. & Baran, P. S. Total Synthesis of Verruculogen and Fumitremorgin A Enabled by Ligand-Controlled C–H Borylation. *J. Am. Chem. Soc*. **137**, 10160–10163 (2015).
36. Bajracharya, G. B. & Daugulis, O. Direct Transition-Metal-Free Intramolecular Arylation of Phenols. *Org. Lett*., **10**, 4625–4628 (2008).

37. Alonso, F., Riente, P. & M. Yus, Transfer hydrogenation of olefins catalysed by nickel nanoparticles. *Tetrahedron*, **65**, 10637-10643 (2009).
38. Amo del, V., McGlone, A. P., Soriano, J. M. & Davis, A. P. Two-colour screening in combinatorial chemistry: prospecting for enantioselectivity in a library of steroid-based receptors. Tetrahedron, **65**, 6370-6381 (2009).
39. Yurino, T., Ueda, Y., Shimizu, Y., Tanaka, S., Nishiyama, H., Tsurugi, H., Sato, K., Mashima, K. Salt-Free Reduction of Nonprecious Transition-Metal Compounds: Generation of Amorphous Ni Nanoparticles for Catalytic C–C Bond Formation. *Angew. Chem.* **127**, 14645-14649 (2015).
40. Sakashita, S., Takizawa, M., Sugai, J., Ito, H. & Yamamoto, Y. Tetrabutylammonium 2-Pyridyltriolborate Salts for Suzuki–Miyaura Cross-Coupling Reactions with Aryl Chlorides. *Org. Lett*., **15**, 4308–4311 (2013).
41. Donohoe, T. J., Fishlock, L. P. & Procopiou, P. A. A Metathesis-Based Approach to the Synthesis of 2-Pyridones and Pyridines. *Org. Lett.*, **10**, 285–288 (2008).
42. Manandhar, S., Singh, R. P., Eggers, G. V. & Shreeve, J. M. Electrophilic Fluorinating Reagent Mediated Synthesis of Fluorinated α-Keto Ethers, Benzil, and 6,6‘-Dialkoxy-2,2‘-bipyridines. *J. Org. Chem.*, **67**, 6415–6420 (2002).
43. Yao, W. et. al. Reinvestigating Catalytic Alcohol Dehydrogenation with an Iridium Dihydroxybipyridine Catalyst. *Organometallics*, **39**, 3656–3662 (2020).
44. Liao, L. Y., Kong, X. R. & Duan, X. F. Reductive Couplings of 2-Halopyridines without External Ligand: Phosphine-Free Nickel-Catalyzed Synthesis of Symmetrical and Unsymmetrical 2,2′-Bipyridines. *J. Org. Chem.*, **79**, 777–782 (2014).
45. Shimoda, T. et. al. Photocatalytic CO_2 reduction by trigonal-bipyramidal cobalt (II) polypyridyl complexes: The nature of cobalt(I) and cobalt (0) complexes upon their reactions with CO_2, CO, or proton. *Inorg. Chem*., **57**, 5486–5498 (2018).
46. Lu, J., Pan, Q., Zhu, S., Liu, R. & Zhu, H. Ligand-Mediated Photophysics Adjustability in Bis-tridentate Ir(III) Complexes and Their Application in Efficient Optical Limiting Materials. *Inorg. Chem.*, **60**, 12835–12486 (2021).
47. Frisch, M. J., Trucks, G. W., Schlegel, H. B., Scuseria, G. E., Robb, M. A., Cheeseman, J. R., Scalmani, G., Barone, V., Mennucci, B., Petersson, G. A., Nakatsuji, H., Caricato, M., Li, X., Hratchian, H. P., Izmaylov, A. F., Bloino, J., Zheng, G., Sonnenberg, J. L., Hada, M., Ehara, M., Toyota, K., Fukuda, R., Hasegawa, J., Ishida, M., Nakajima, T., Honda, Y., Kitao, O., Nakai, H.,

Vreven, T., Montgomery, J. A. Jr., Peralta, J. E., Ogliaro, F., Bearpark, M., Heyd, J. J., Brothers, E., Kudin, K. N., Staroverov, V. N., Keith, T., Kobayashi, R., Normand, J., Raghavachari, K., Rendell, A., Burant, J. C., Iyengar, S. S., Tomasi, J., Cossi, M., Rega, N., Millam, J. M., Klene, M., Knox, J. E., Cross, J. B., Bakken, V., Adamo, C., Jaramillo, J., Gomperts, R., Stratmann, R. E., Yazyev, O., Austin, A. J., Cammi, R., Pomelli, C., Ochterski, J. W., Martin, R. L., Morokuma, K., Zakrzewski, V. G., Voth, G. A., Salvador, P., Dannenberg, J. J., Dapprich, S., Daniels, A. D., Farkas, O., Foresman, J. B., Ortiz, J. V., Cioslowski, J. & Fox, D. J. Gaussian 09, revision E.01, Gaussian, Inc., Wallingford CT, 2013.

48. Becke, A. D. Density-functional thermochemistry. III. the role of exact exchange. *J. Chem. Phys.* **98**, 5648-5652 (1993).

49. Lee, C., Yang, W. & Parr, R. G. Development of the Colle-Salvetti correlation-energy formula into a functional of the electron density. *Phys. Rev. B* **37**, 785-789 (1988).

50. Hay, P. J. & Wadt, W. R. Ab initio effective core potentials for molecular calculations - potentials for the transition-metal atoms Sc to Hg. *J. Chem. Phys.* **82**, 270-283 (1985).

51. Fukui, K. The path of chemical reactions - the IRC approach. *Acc. Chem. Res.* **14**, 363-368 (1981).

52. Zhao, Y. & Truhlar, D. G. The M06 suite of density functionals for main group thermochemistry, thermochemical kinetics, noncovalent interactions, excited states, and transition elements: two new functionals and systematic testing of four M06-class functionals and 12 other functionals. *Theor. Chem. Acc.* **120**, 215-241 (2008).

53. Andrae, D., Haussermann, U., Dolg, M., Stoll, H. & Preuss, H. Energy-adjusted ab initio pseudopotentials for the 2nd and 3rd row transition-elements. *Theor. Chim. Acta.* **77**, 123-141 (1990).

54. Grimme, S., Antony, J., Ehrlich, S. & Krieg, H. A consistent and accurate ab initio parametrization of density functional dispersion correction (DFT-D) for the 94 elements H-Pu. *J. Chem. Phys.* **132**, 154104 (2010).

55. Grimme, S., Ehrlich, S. & Goerigk, L. Effect of the Damping Function in Dispersion Corrected Density Functional Theory. *J. Comput. Chem.* **32**, 1456-1465 (2011).

56. Chai, J.-D. & Head-Gordon, M. Long-range corrected hybrid density functionals with damped atom-atom dispersion corrections. *Phys. Chem. Chem. Phys.* **10**, 6615-6620 (2008).

57. Marenich, A. V., Cramer, C. J. & Truhlar, D. G. Universal solvation model based on solute electron density and on a continuum model of the solvent defined by the bulk dielectric constant and atomic surface tensions. *J. Phys. Chem. B* **113**, 6378-6396 (2009).
58. Legault, C. Y. CYLview, 1.0b. Université de Sherbrooke, 2009 (http://www.cylview.org).
